# Draft genome of *Glyptosternon maculatum*, an endemic fish from Tibet Plateau

**DOI:** 10.1093/gigascience/giy104

**Published:** 2018-08-14

**Authors:** Haiping Liu, Qiyong Liu, Zhiqiang Chen, Yanchao Liu, Chaowei Zhou, Qiqi Liang, Caixia Ma, Jianshe Zhou, Yingzi Pan, Meiqun Chen, Wangjiu, Wenkai Jiang, Shijun Xiao, Zhenbo Mou

**Affiliations:** 1 Institute of Fisheries Science, Tibet Academy of Agricultural and Animal Husbandry Sciences, 130 Jinzhu West Road, Lhasa 850002, Tibet, China; 2 Novogene Bioinformatics Institute, Zone A10 jiuxianqiao North Road, Chaoyang District, Beijing 100000, China; 3 School of Computer Science and Technology, Wuhan University of Technology, 122 Luoshi Road, Wuhan 430070, Hubei, China

**Keywords:** *Glyptosternon maculatum*, genome assembly, annotation, phylogeny

## Abstract

**Background:**

Mechanisms for high-altitude adaption have attracted widespread interest among evolutionary biologists. Several genome-wide studies have been carried out for endemic vertebrates in Tibet, including mammals, birds, and amphibians. However, little information is available about the adaptive evolution of highland fishes. *Glyptosternon maculatum* (Regan 1905), also known as Regan or barkley and endemic to the Tibetan Plateau, belongs to the Sisoridae family, order Siluriformes (catfishes). This species lives at an elevation ranging from roughly 2,800 m to 4,200 m. Hence, a high-quality reference genome of *G. maculatum* provides an opportunity to investigate high-altitude adaption mechanisms of fishes.

**Findings:**

To obtain a high-quality reference genome sequence of *G. maculatum*, we combined Pacific Bioscience single-molecule real-time sequencing, Illumina paired-end sequencing, 10X Genomics linked-reads, and BioNano optical map techniques. In total, 603.99 Gb sequencing data were generated. The assembled genome was about 662.34 Mb with scaffold and contig N50 sizes of 20.90 Mb and 993.67 kb, respectively, which captured 83% complete and 3.9% partial vertebrate Benchmarking Universal Single-Copy Orthologs. Repetitive elements account for 35.88% of the genome, and  22,066 protein-coding genes were predicted from the genome, of which 91.7% have been functionally annotated.

**Conclusions:**

We present the first comprehensive *de novo* genome of *G. maculatum*. This genetic resource is fundamental for investigating the origin of *G. maculatum* and will improve our understanding of high-altitude adaption of fishes. The assembled genome can also be used as reference for future population genetic studies of *G. maculatum*.

## Data Description

### Background information on *Glyptosternon maculatum*

**Figure 1:**
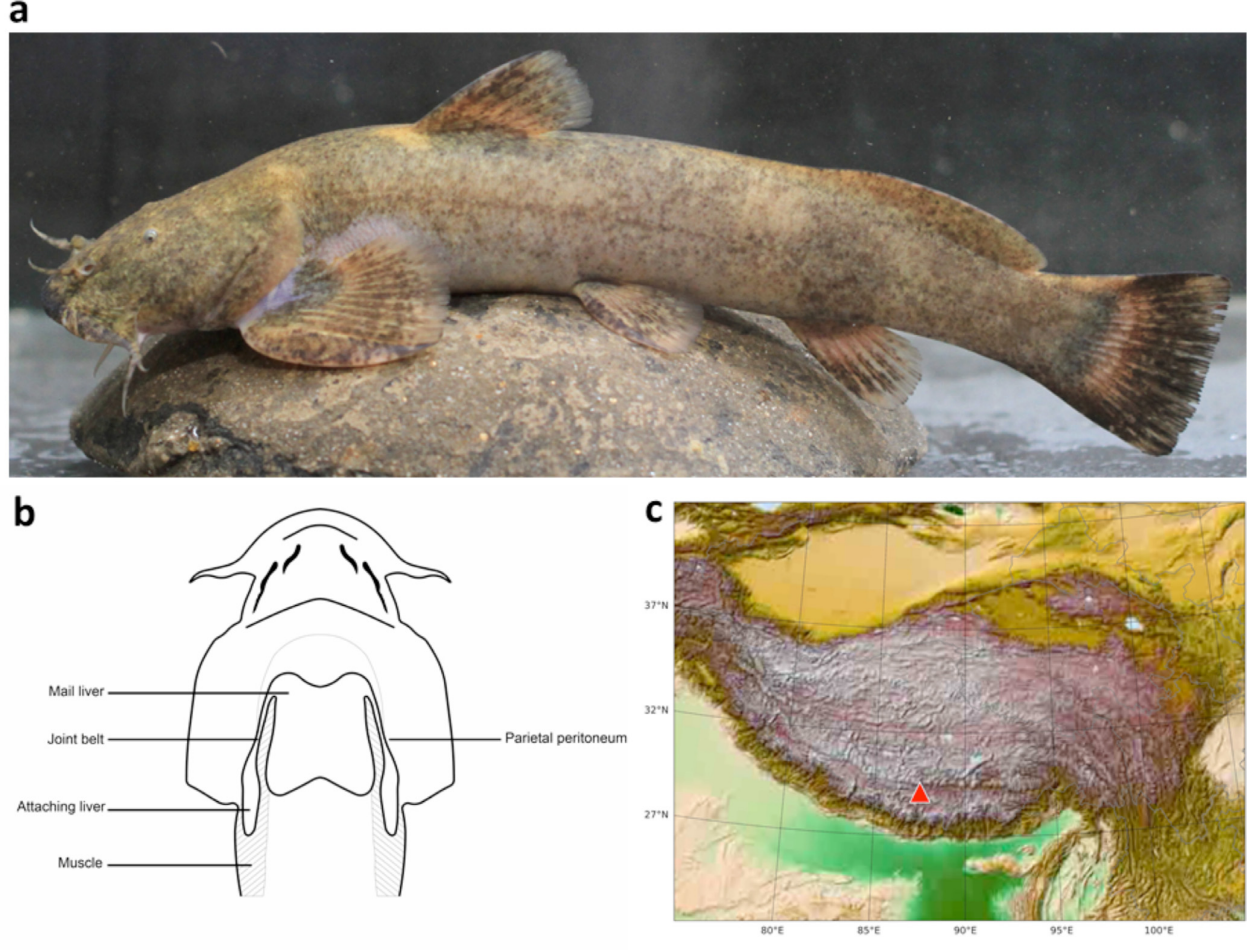
**(a)** The appearance of *G. maculatum*. **(b)** The liver of *G. maculatum* was divided into two parts, one placed outside the abdominal cavity (attaching liver), connected to another part that is located inside the cavity (mail liver). **(c)** Distributed localization (red triangle) of *G. maculatum* for sequencing. (Figure schematic drawings (ventral view) of *G. maculatum* (imaged from Zhang [9]).

*Glyptosternon maculatum* (Regan 1905; Fishbase ID: 24838, National Center for Biotechnology Information [NCBI] Taxon ID: 175778), also called barkley in Tibetan language, is a species in the genus *Glyptosternum* (family Sisoridae, order Siluriformes, infraclass Teleostei; Fig. 1a, 1b). The Sisoridaes are the largest family of catfishes (Siluriformes) in China, consisting of 44 species divided into two natural groups, Glyptosternoids and non-Glyptosternoids [1, 2]. There are eight Sisorids distributed in the Yarlung Tsangpo (Brahmaputra) River. Of them, *G. maculatum* is the only species that is distributed at the middle section. Specifically, it is distributed at Niyang tributary, the Tangjia to Zhaxue segment of the Lhasa tributary and the Xietongmen segment of Yarlung Tsangpo, across an elevation ranging from roughly 2,800 m to 4,200 m [3].

The karyotype of *G. maculatum* is a debated topic. Ren and Cui [4] reported a result of 2n = 48 = 28m+12sm+8st, NF = 88, based on specimens sampled at Quxur, and speculated that it may be the most specialized karyotype among all sisoridaes. Conversely, Wu et al. [5] reported a karyotype of 2n = 48 = 22m+12sm+10st+6t, NF = 80 sampled at Xigaze, while karyotypes of 2n = 44 and 2n = 42 were also found. They compared it to other Sisorid karyotypes and concluded that the karyotype of *G. maculatum* was not the most specialized. The genome assembly of *G. maculatum* might provide a route to resolve these debates.

Fishes from the Glyptosternoid group are distributed broadly at the south and southeast drainages of the Tibetan Plateau, providing a good model to study the speciation process caused by the up-shift of the Tibetan Plateau. He et al. [1] conducted a cladistic analysis of the Glyptosternoid group based on 60 bone features and found that Glyptosternoids formed a monophyletic group, of which the *Glyptosternum* were the most primitive clade. He et al. [6] further analyzed the phylogeny of Glyptosternoids using 19 species distributed in four genera by their bone features, in combination with biogeographical analysis. They postulated the rise of the Tibetan Plateau had a direct influence on the diversification of Glyptosternoids, with *Glyptosternum* (particularly *G. maculatum*) as the most primitive clade, which was consistent with the conclusion of Hora and Silas [2]. Peng et al. [7] sequenced mitochondrial cytochrome b from 13 Glyptosternoids, also supporting them to be a monophyletic group, with *Glyptosternum* and *Exostoma* as relatively primitive clades. We thus chose *G. manulatum* to represent the fishes of the Glyptosternoid group. Its whole genome sequence can provide a foundation to explore the adaptive evolution process of highland fishes and also be used as a starting point to study speciation mechanisms caused by the rapid rising of the Tibetan Plateau.

*Glyptosternon maculatum* has a specialized liver that can be divided into two parts, one placed outside the abdominal cavity, connected to another part located inside the cavity [8] (Fig. 1b). Several studies reported similar ectopic livers in other Sisords, suggesting that this specialized organ might be the result of adaptive evolution [9]. The genesis of the liver in *G. maculatum* occurs in three stages: the ectopic liver is not present from the beginning until the end of the larva's exit from the egg envelope; a “bump” then develops, starting from day 17 until day 22; the ectopic liver appears on day 22 [9]. Zhang [9] pointed out that expression of copper-zinc superoxide dismutase (SOD), manganese SOD, and catalase (CAT) mRNA e all higher in the primary liver relative to the secondary liver, suggesting that the two livers have different physiological roles in *G. maculatum*. However, the molecular mechanisms of liver development and their physiological functions in adaptive evolution are not fully understood. The genome assembly also provides a solid foundation for the investigation of liver biology in this species.

### Sample collection and sequencing

The female fish individual used for genome sequencing originated from Angren, Xizang Province (Fig. 1c). Total genomic DNA was extracted from muscular tissue and kept at Novogene Bioinformatics Institute.

A combination of four technologies was applied: Pacific Bioscience's single-molecule real-time sequencing, Illumina's paired-end sequencing, 10X Genomics link-reads, and BioNano optical maps. Two paired-end Illumina sequence libraries were constructed with an insert size of 250 bp, and sequencing was carried out on the Illumina HiSeq 4000 platform according to the manufacturer's instructions; 147.16 Gb (191x coverage) sequencing data were produced. In addition, one 10X Genomics linked-read library was constructed and sequencing was performed on Illumina HiSeq 4000 platform, which produced 157 Gb (203.5x coverage) sequencing data. Raw sequence data generated by the Illumina platform were filtered by the following criteria: filtered reads with adapters, filtered reads with N bases more than 10%, and filtered reads with low-quality bases (≤ 5) more than 50%. PacBio reads were sequenced by the Sequel platform, which gained 106.3 Gb (145.2x coverage) sequencing data. For the PacBio data, subreads were filtered with the default parameters. Finally, we obtained 106.32 Gb of long reads (polymerase reads) data. The average and the N50 length of long subreads reached 8.04 kb and 13.26 kb, respectively. An optical map was also constructed from Irys platform (BioNano Genomics), of which 191.3 Gb (248x coverage) data were generated. All sequence data are summarized in Table 1.

**Table 1:** Sequencing data used for the *G. maculatum* genome assembly

Pair-end libraries	Insert size (bp)	Raw data (Gb)	Clean data (Gb)	Read length (bp)	Sequence coverage (X)
Illumina reads	250 bp	148.16	147.16	150	191
PacBio reads	20 Kb	106.32	106.05	11 745	145.2
10X Genomics	500 bp	157.21	157.02	150	203.5
BioNano	–	192.30	191.30	–	248
Total	–	603.99	601.53	–	787.7

The coverage was calculated using an estimated genome size of 771.19 Mb.

### *De novo* assembly of *G. maculatum* genome

The genome size was estimated based on the *k*-mer spectrum: G = (K_total_– K_error_)/D, where K_total_ is the total count of *k*-mers, K_error_ is the total count of low-frequency (frequency ≤3) *k*-mers that were probably caused by sequencing errors, G is the genome size, and D is the *k*-mer depth. Using Jellyfish [10] (v2.1.3), 17-mers were counted as 54 ,676  ,846, 244 from short clean reads. The total count of error *k*-mers was  1,980 ,028 ,579 and the *k*-mer depth was 69 (Supplementary Fig. S1). Therefore, the genome size of *G. maculatum* was estimated to be ∼763.7 Mb.

The contig assembly of the *G. maculatum* genome was carried out using the FALCON assembler [11], followed by two rounds of polishing with Quiver [12]. FALCON implements a hierarchical assembly process that include the following steps: (1) subread error correction through aligning all reads to each other using daligner [13], the overlap data were then processed to generate error-corrected consensus reads; after error correction, we obtained 28 Gb (35x coverage) of error-corrected reads; (2) second round of overlap detection using error-corrected reads; (3) construction of a directed string graph from overlap data; and (4) resolving contig path from the string graph. After FALCON assembly, the genome was polished by Quiver. Initial assembly of the PacBio data alone resulted in a contig N50 (the minimum length of contigs accounting for half of the haploid genome size) of 697.4 Kb. Then, PacBio contigs were first scaffolded using optical map data, and the resulting scaffolds were further connected to super-scaffolds by 10X Genomics linked-read data using the fragScaff software [14]. Finally, we used Illumina-derived short reads to correct any remaining errors by pilon [15]. These processes yielded a final draft *G. maculatum* genome assembly with a total length of 662.34 Mb, contig N50 of 993.67 kb, and scaffold N50 of 20.90 Mb (Table 2).

**Table 2:** Assembly statistics of *G. maculatum*

Sample ID	Length		Number	
	Contig^a^ (bp)	Scaffold (bp)	Contig^a^	Scaffold
Total	637,133,884	662,339,741	3,281	531
Max	5,772,991	47,179,384	-	-
Number ≥ 2000	-	-	3,161	531
N50	993,673	20,902,354	161	11
N60	668,112	17,328,106	239	14
N70	418,057	12,288,896	359	19
N80	211,596	6,320,921	575	27
N90	77,392	1,017,220	1,067	50

^a^Contig after scaffolding.

To evaluate the accuracy of the genome at the single base level, we mapped short sequence reads generated by Illumina platform to the *G. maculatum* genome with BWA (BWA, RRID:SCR_010910) [16] and performed variant calling with SAMtools (SAMTools, RRID:SCR_002105) [17]. We obtained 3,632 homozygous single-nucleotide polymorphisms (Supplementary Table S2), reflecting a low homozygous rate (0.0007%) and a high accuracy of genome assembly at the single base level.

To assess the completeness of the assembled *G. maculatum* genome, we performed Benchmarking Universal Single-Copy Orthologs (BUSCO) (BUSCO, RRID:SCR_015008) analysis [18] by searching against the vertebrate BUSCO (version 3.0). Overall, 83% complete and 3.9% partial of the 970 vertebrate BUSCOs were identified in the assembled genome. We also assessed the completeness of *G. maculatum* genome by the Core Eukaryotic Genes Mapping Approach (CEGMA) (RRID:SCR_015055) [19]. According to CEGMA, 211 (85.08%) conserved genes were identified in the *G. maculatum* genome.

The muscle transcriptome *de novo* assembled by Trinity (Trinity, RRID:SCR_013048) [20] was also mapped to the genome assembly using the Basic Local Alignment Search Tool-like alignment tool [21] with default parameters, showing that the alignment coverage of expressed sequences ranged from 75% to 99% in the genome assembly. To answer the question of why some contigs have a low coverage (85%) on genome sequence alignment. We first searched mRNA sequencing reads to NT database and found that the top five hits ware all from the closely related fish species, such as *Ictalurus punctatus* and *Danio rerio* (Supplementary Table 7). Therefore, the probability for external contamination was ruled out. We therefore attributed the low coverage of some trinity contigs to two reasons: first, the potential chimeric transcript generated during the transcriptome assembly using trinity, especially for genes with various alternative splices, and, second, the fragments of genomic contig sequences.

### Annotation of repetitive sequences in *G. maculatum* genome

The repetitive sequences in the *G. maculatum* genome were identified by a combination of homology searching and *ab initio* prediction. For homology-based prediction, we used RepeatMasker (RepeatMasker, RRID:SCR_012954) [22] and RepeatProteinMask to search against Repbase. For *ab initio* prediction, we used Tandem Repeats Finder [23], LTR_FINDER (LTR_FINDER, RRID:SCR_015247) [24], PILER [25], and RepeatScout (RepeatScout, RRID:SCR_014653) [26] with default parameters. We found that 33.96% of the *G. maculatum* assembly was composed of repetitive elements (Supplementary Table S3 and Figure S2). Additionally, we predicted miniature inverted–repeat transposable elements (MITEs) through the genome using MITE-digger [27] with default parameters. As a result, we identified 2962 MITEs accounting for 0.185% of the whole genome (Supplemental File MITE).

### Protein coding gene prediction and ncRNA prediction

Gene prediction was conducted through a combination of homology-based prediction, *ab initio* prediction, and transcriptome-based prediction methods. Protein repertoires of vertebrates including *Takifugu rubripes* (Tru, GCF_000180615.1), *Ctenopharyngodon idellus* (Cid) [28], *Cyprinus carpio* (Cca, GCF_000951615.1), *Danio rerio* (Dre, GCF_000002035.5), *Sinocyclocheilus graham* (Sga, GCF_001515645.1), channel catfish (Ipu, GCF_001660625.1), *Homo sapiens* (Hom, GCF_000001405.37), and *Mus musculus* (Mmu, GCF_000001635.26) were used as queries to search against the *G. maculatum* genome using TBLASTN (TBLASTN, RRID:SCR_011822) [29]. The Basic Local Alignment Search Tool (BLAST) hits were conjoined by Solar software [30]. GeneWise (GeneWise, RRID:SCR_015054) [31] was used to predict the exact gene structure of the corresponding genomic region on each BLAST hit. Homology predictions were denoted as “Homology-set” (Supplementary Table S4). RNA-sequencing (RNA-seq) data derived from 10 tissues that obtained about 77.29 Gb clean data were assembled by Trinity [20]. The Trinity assembly included 572, 416 contigs with an average length of 1,075 bp. These assembled sequences were aligned against the *G. maculatum* genome by Program to Assemble Spliced Alignment (PASA). Valid transcript alignments were clustered based on genome mapping location and assembled into gene structures. Gene models created by PASA [32] were denoted as PASA-T-set (PASA Trinity set).In addition, RNA-seq reads were directly mapped to the genome using Tophat (Tophat, RRID:SCR_013035) [33] to identify putative exon regions and splice junctions; Cufflinks (Cufflinks, RRID:SCR_014597) [34] was then used to assemble the mapped reads into gene models (Cufflinks-set). Augustus (Augustus, RRID:SCR_008417) [35], GeneID [36], GeneScan [37], GlimmerHMM (GlimmerHMM, RRID:SCR_002654) [38], and SNAP [39] were also used to predict coding regions in the repeat-masked genome. Of these, Augustus, SNAP, and GlimmerHMM were trained by PASA-H-set gene models. Gene models generated from all the methods were integrated by EvidenceModeler [40]. Weights for each type of evidence were set as follows: PASA-T-set > Homology-set > Cufflinks-set > Augustus > GeneID = SNAP = GlimmerHMM = GeneScan. The gene models were further updated by PASA2 to generate untranslated regions, alternative splicing variation information. We have identified  22,066 protein-coding genes with a mean of 8.5 exons per gene (Table 3). The lengths of genes, coding sequence, introns, and exons in *G. maculatum* were comparable to those of closely related genomes (Supplementary Table S4 and Figure S3). In addition, we predicted noncoding RNA genes in the *G. maculatum* genome. The rRNA fragments were predicted by searching against Human rRNA database using BLAST with an E-value of 1E-10. The tRNA genes were identified by tRNAscan-SE (tRNAscan-SE, RRID:SCR_010835) software [41]. The miRNA and snRNA genes were predicted by INFERNAL (INFERNAL, RRID:SCR_011809) [42] using Rfam database [43]. We found 3,117 ribosomal RNA (rRNA), 3,512 transfer RNA (tRNA), 1,235 microRNAs (miRNA), and 781 snRNA genes in the *G. maculatum* genome (Supplementary Table S5).

**Table 3:** General statistics of predicted protein-coding genes

Gene set		Number	Average transcript length (bp)	Average coding sequence length (bp)	Average exons per gene	Average exon length (bp)	Average intron length (bp)
*De novo*	Augustus	14,910	9,534	1,241	6.93	179	1,399
	GlimmerHMM	73,320	7,896	574	3.87	148	2,551
	SNAP	43,247	15,950	847	6.04	140	2,996
	Geneid	23,523	16,924	1,323	6.29	210	2,948
	Genscan	24,037	19,024	1,514	8.14	186	2,451
	*Sga*	32,364	6,413	1,142	5.12	223	1,279
	*Cca*	27,208	6,326	1,252	5.36	234	1,165
	*Cid*	30,336	5,615	1,048	4.87	215	1,181
	*Dre*	19,458	9,935	1,507	7.58	199	1,280
Homolog	*Hom*	16,090	10,844	1,432	7.83	183	1,379
	*Tru*	23,120	8,191	1,225	6.12	200	1,362
	*Mmu*	16,164	10,803	1,417	7.74	183	1,392
	*Ipu*	37,610	6,704	1,155	5.22	221	1,315
RNA-seq	PASA	97,309	9,419	1,201	7.09	169	1,348
	Cufflinks	92,180	19,478	4,707	10.13	465	1,618
EvidenceModeler	25,365	11,517	1,323	7.66	173	1,531	
PASA-update*	38,086	13,009	1,521	8.79	173	1,475	
Final set*	22,066	12,913	1,458	8.48	172	1,531	

### Functional annotation of protein-coding genes

Gene functions of protein-coding genes were annotated by searching functional motifs, domains, and the possible biological process of genes to known databases such as SwissProt [44], Pfam [45], NR database (from NCBI), Gene Ontology [46], and Kyoto Encyclopedia of Genes and Genomes [47]. In total,  20,234 protein-coding genes (91.7%) were successfully annotated for at least one function terms (Supplementary Table S6, Figure S4).

### Phylogenetic analysis and species divergence time estimation

To investigate the phylogenic position of *G. maculatum*, we retrieved nucleotide and protein data for *Cyprinus carpio* (GCF_000951615.1), *Sinocyclocheilus rhinocerous* (GCF_001515625.1), *Sinocyclocheilus anshuiensis* (GCF_001515605.1), *Astyanax mexicanus* (GCF_000372685.2), *Pygocentrus nattereri* (GCF_001682695.1), *Sinocyclocheilus grahami* (GCF_001515645.1), *Ictalurus punctatus* (GCF_001660625.1), *Danio rerio* (GCF_000002035.6), *Amazon molly* (GCF_000485575.1), *Oreochromis niloticus* (GCF_001858045.1), *Takifugu rubripes* (GCF_000180615.1), and *Ctenopharyngodon idellus* [28] from public databases. To remove redundancy caused by alternative splicing variations, we retained only gene models at each gene locus that encoded the longest protein sequence. To exclude putative fragmented genes, genes encoding protein sequences shorter than 50 amino acids were filtered out. All-against-all BLASTP (BLASTP, RRID:SCR_001010) [29] was employed to identity the similarities among filtered protein sequences in these species with an E-value cutoff of 1e^−7^. The OrthoMCL (OrthoMCL, RRID:SCR_007839) [48] method was used to cluster genes from these different species into gene families with the parameter of “-inflation 1.5.”

A total of  26,588 gene family clusters were constructed. There were 101 gene families and 228 genes in *G. maculatum* without significant homologous hits to other teleosts. We further searched the 228 genes to NCBI NR database by BLASTP and found that 142 genes hit to database with e-value of 1e-5 and that 86 genes still failed to hit any protein sequences in the database. The function of those genes lacking significant homology is an interesting topic in the following studies.

**Figure 2:**
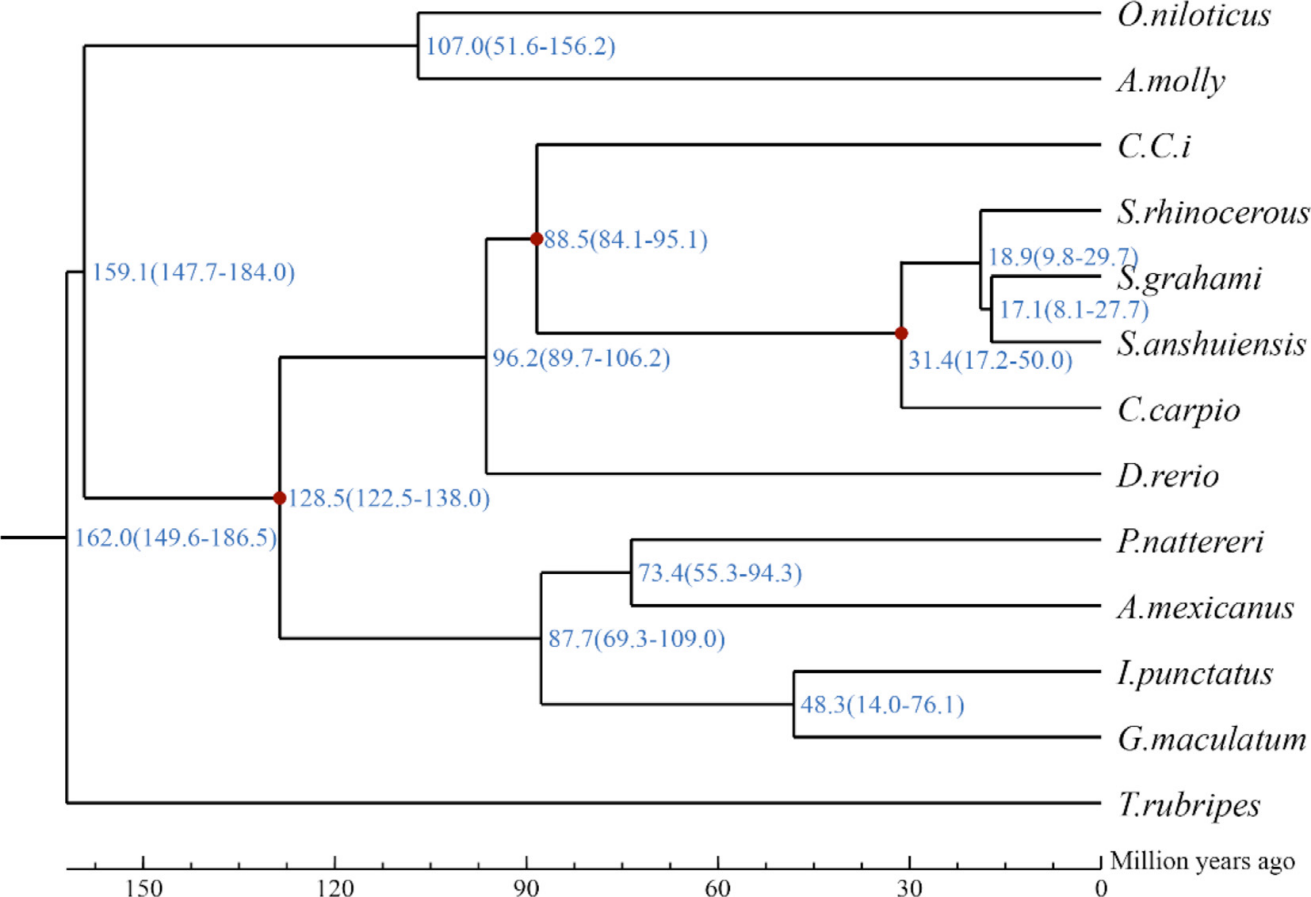
Divergence time estimated between *G. maculatum* and other species.

Protein sequences from 247 single-copy gene families were used for phylogenetic tree reconstruction. MUSCLE (MUSCLE, RRID:SCR_011812) [49] was used to generate multiple sequence alignments for protein sequences in each single-copy family with default parameters. Then, the alignments of each family were concatenated to a super alignment matrix. The super alignment matrix was used for phylogenetic tree reconstruction through maximum likelihood methods. Divergence time between species was estimated using MCMCtree in PAML [50] with the options “correlated molecular clock” and “JC69” model. A Markov chain Monte Carlo analysis was run for  20,000 generations using a burn-in of 1,000 iterations. Divergence time for the common ancestor of *C. idellus*, *S. rhinocerous*, and *P. nattereri* obtained from the TimeTree database (http://www.timetree.org/) was used as the calibrate point. These phylogenetic analyses indicated that *G. maculatum* diverged from the common ancestral of *I. punctatus* approximately 48.3 million years ago (Fig. 2) [51].

## Conclusion

We have constructed a *de novo* assembly of the *G. maculatum* genome and describe its genetic attributes. To our knowledge, this is the first *de novo* genome for Glyptosternoids group of fishes. The *G. maculatum* genome will support investigations concerning the origin and evolutionary history of Glyptosternoid. This resource will be important for the future conservation of this endangered plateau species. In addition, the *G. maculatum* genome laid a solid foundation to investigate molecular mechanism of high-altitude adaption of fishes and the speciation process during the rising of the Tibetan Plateau.

## Availability of supporting data

The raw sequencing data are available via NCBI under SRA accessions SRR7279473-SRR7279474, SRR7268130-SRR7268162, SRR7350914-SRR7350921, SRR7351269-SRR7351265, SRR7403445-SRR7403454 (BioProject accession number PRJNA447978). Supporting data, including also the genome assembly, annotations, BUSCO results, phylogenetic trees, and scripts are available via the *GigaScience* database GigaDB [51]. Raw and physical mapping data were also deposited at the National Omics Data Encyclopedia (NODE) with the project ID OEP000007. All supplementary figures and tables are provided as Additional File.

## Additional files

Supplemental_file.docx.

Supplemental_file_MITE.docx.

## Abbreviations

BLAST: Basic Local Alignment Search Tool; BUSCO: Benchmarking Universal Single-Copy Orthologs; CEGMA: Core Eukaryotic Genes Mapping Approach; MITE: miniature inverted–repeat transposable elements; NCBI: National Center for Biotechnology Information; PacBio: Pacific Biosciences; PASA: Program to Assemble Spliced Alignment; PASA-T-set: PASA Trinity set; RNA-seq: RNA sequen

## Competing interests

All authors declare that they have no competing interest.

## Funding

This work was supported by the special finance of Tibet Autonomous Region (grant 2017CZZX003) and the National Natural Science Foundation of China (grants 31560144 and 31602207).

## Author contributions

H.L., W.J., and Z.M. conceived the study. H.L. and W.J. designed the scientific objectives. Q.L. and Z.M. managed the project. Y.L. and C.Z. collected the samples and extracted the genomic DNA. Z.C. estimated the genome size and assembled the genome. Q.L. and C.M. assessed the assembly quality. J.Z. and Y.P. carried out the repeat annotation and gene annotation. Z.C. carried out comparative genomics analysis. H.L., S.X., Z.C., and W.J. wrote the manuscript. All authors read, edited, and approved the final manuscript.

## Supplementary Material

GIGA-D-18-00128_(Original_Submission).pdf

GIGA-D-18-00128_Revision_1.pdf

GIGA-D-18-00128_Revision_2.pdf

GIGA-D-18-00128_Revision_3.pdf

Response_to_Reviewer_Comments_Original_Submission.pdf

Response_to_Reviewer_Comments_Revision_1.pdf

Response_to_Reviewer_Comments_Revision_2.pdf

Reviewer_1_Report_(Original_Submission) -- Geoffrey Waldbieser 05-08-2018 Reviewed

Reviewer_2_Report_(Original_Submission) -- Jean-Nicolas Volff 5/18/2018 Reviewed

Supplemental Files

## References

[1] He S The phylogeny of the glyptosternoid fishes (Teleostei: Siluriformes, Sisoridae). Cybium. 1996;20(2):115–59.

[2] Hora SL, Silas EG Evolution and distribution of Glyptosternoid fishes of the family Sisoridae (Order: Siluroidea). Proc Nat Inst Sci of India. 1952;18(4):309–22.

[3] Cheng QT, Zheng BS Systematic synopsis of Chinese Fishes. Beijing: Science Press, 1987, 289–296.

[4] Ren X The karyotype and haploidy NOR of *Glyptosternum maculatum*. Hereditas. 1992; 14(6):10-11.

[5] Wu Y-f, Kang B, Men Q, Chromosome diversity of Tibetan fishes. Zoological Research. 1999;20(4):258–64.

[6] He S, Cao W, Chen Y The uplift of Qinghai-Xizang (Tibet) Plateau and the vicariance speciation of glyptosternoid fishes (Siluriformes: Sisoridae). Science China. 2001;44(6):644. 10.1007/BF02879359 18763106

[7] Peng Z, He S, Zhang Y Phylogenetic relationships of glyptosternoid fishes (Siluriformes: Sisoridae) inferred from mitochondrial cytochrome b gene sequences. Molecular Phylogenetics & Evolution. 2004;31(3):979–87. 15120395 10.1016/j.ympev.2003.10.023

[8] Li H, Xie X, Li D, Exo-celiac Liever in Glyptosternum Maculatum, Progress in Natural Science: Materials International. 2017, 17((9):):1109–1113.

[9] Huijuan Z Genesis of Liver in *Glyptosternum maculatum* and Related Bioadaptive Studies, in Library of Huazhong Agricultural University. Huazhong Agricultural University: WuHan, China, 2011.

[10] Marcais G, Kingsford C A fast, lock-free approach for efficient parallel counting of occurrences of k-mers. Bioinformatics. 2011;27(6):764–70. 21217122 10.1093/bioinformatics/btr011 PMC3051319

[11] Pendleton M, Sebra R, Pang AW Assembly and diploid architecture of an individual human genome via single-molecule technologies. Nat Methods. 2015;12(8):780–6. 26121404 10.1038/nmeth.3454 PMC4646949

[12] Chin CS, Alexander DH, Marks P, Nonhybrid, finished microbial genome assemblies from long-read SMRT sequencing data. Nat Methods. 2013;10(6):563–9. 23644548 10.1038/nmeth.2474

[13] Myers G Efficient local alignment discovery amongst noisy long reads. In: Algorithms in Bioinformatics. Berlin, Heidelberg: Springer Berlin Heidelberg 2014.

[14] Adey A, Kitzman JO, Burton JN, In vitro, long-range sequence information for de novo genome assembly via transposase contiguity. Genome Res. 2014;24(12):2041–9. 25327137 10.1101/gr.178319.114 PMC4248320

[15] Walker BJ, Abeel T, Shea T, Pilon: an integrated tool for comprehensive microbial variant detection and genome assembly improvement. PLoS One. 2014;9(11):e112963 25409509 10.1371/journal.pone.0112963 PMC4237348

[16] Li H, Durbin R Fast and accurate short read alignment with Burrows-Wheeler transform, Bioinformatics. 2009, 25((14):):1754–1760. 10.1093/bioinformatics/btp324 PMC2705234 19451168

[17] Li H A stical framework for SNP calling, mutation discovery, association mapping and population genetical parameter estimation from sequencing data. Bioinformatics. 2011;27(21):2987–93. 21903627 10.1093/bioinformatics/btr509 PMC3198575

[18] Simao FA, Waterhouse RM, Ioannidis P BUSCO: assessing genome assembly and annotation completeness with single-copy orthologs. Bioinformatics. 2015;31(19):3210–2. 26059717 10.1093/bioinformatics/btv351

[19] Parra G, Bradnam K, Korf I CEGMA: a pipeline to accurately annotate core genes in eukaryotic genomes. Bioinformatics. 2007;23(9):1061–7. 17332020 10.1093/bioinformatics/btm071

[20] Grabherr MG, Haas BJ, Yassour M, Full-length transcriptome assembly from RNA-Seq data without a reference genome. Nat Biotechnol. 2011;29(7):644–52. 21572440 10.1038/nbt.1883 PMC3571712

[21] Kent WJ BLAT–the BLAST-like alignment tool. Genome Res. 2002;12(4):656–64. 11932250 10.1101/gr.229202 PMC187518

[22] Bergman CM, Quesneville H Discovering and detecting transposable elements in genome sequences. Brief Bioinform. 2007;8(6):382–92. 17932080 10.1093/bib/bbm048

[23] Benson G Tandem repeats finder: a program to analyze DNA sequences. Nucleic Acids Res. 1999;27(2):573–80. 9862982 10.1093/nar/27.2.573 PMC148217

[24] Xu Z, Wang H LTR_FINDER: an efficient tool for the prediction of full-length LTR retrotransposons. Nucleic Acids Res. 2007;35(Web Server issue):W265–8. 17485477 10.1093/nar/gkm286 PMC1933203

[25] Edgar RC, Myers EW PILER: identification and classification of genomic repeats. Bioinformatics. 2005;21 Suppl 1:i152–8. 15961452 10.1093/bioinformatics/bti1003

[26] Price AL, Jones NC, Pevzner PA De novo identification of repeat families in large genomes. Bioinformatics. 2005;21 Suppl 1:i351–8. 15961478 10.1093/bioinformatics/bti1018

[27] Yang G Digger, an efficient and accurate algorithm for genome wide discovery of miniature inverted repeat transposable elements. BMC Bioinformatics. 2013;14:186. 23758809 10.1186/1471-2105-14-186 PMC3680318

[28] Wang Y, Lu Y, Zhang Y, The draft genome of the grass carp (*Ctenopharyngodon idellus*) provides insights into its evolution and vegetarian adaptation. Nat Genet. 2015;47(6):625–31. 25938946 10.1038/ng.3280

[29] Altschul SF, Gish W, Miller W, Basic local alignment search tool. J Mol Biol. 1990;215(3):403–10. 2231712 10.1016/S0022-2836(05)80360-2

[30] Yu XJ, Zheng HK, Wang J, Detecting lineage-specific adaptive evolution of brain-expressed genes in human using rhesus macaque as outgroup. Genomics. 2006;88(6):745–51. 16857340 10.1016/j.ygeno.2006.05.008

[31] Birney E, Clamp M, Durbin R GeneWise and Genomewise. Genome Res. 2004;14(5):988–95. 15123596 10.1101/gr.1865504 PMC479130

[32] Haas BJ, Delcher AL, Mount SM, Improving the Arabidopsis genome annotation using maximal transcript alignment assemblies. Nucleic Acids Res. 2003;31(19):5654–66. 14500829 10.1093/nar/gkg770 PMC206470

[33] Kim D, Pertea G, Trapnell C TopHat2: accurate alignment of transcriptomes in the presence of insertions, deletions and gene fusions. Genome Biol. 2013;14(4):R36. 23618408 10.1186/gb-2013-14-4-r36 PMC4053844

[34] Trapnell C, Roberts A, Goff L Differential gene and transcript expression analysis of RNA-seq experiments with TopHat and Cufflinks. Nat Protoc. 2012;7(3):562–78. 22383036 10.1038/nprot.2012.016 PMC3334321

[35] Stanke M, Waack S Gene prediction with a hidden Markov model and a new intron submodel. Bioinformatics. 2003;19 Suppl 2:ii215–25. 14534192 10.1093/bioinformatics/btg1080

[36] Guigo R Assembling genes from predicted exons in linear time with dynamic programming. J Comput Biol. 1998;5(4):681–702. 10072084 10.1089/cmb.1998.5.681

[37] Burge C, Karlin S Prediction of complete gene structures in human genomic DNA. J Mol Biol. 1997;268(1):78–94. 9149143 10.1006/jmbi.1997.0951

[38] Majoros WH, Pertea M, Salzberg SL TigrScan and GlimmerHMM: two open source ab initio eukaryotic gene-finders. Bioinformatics. 2004;20(16):2878–9. 15145805 10.1093/bioinformatics/bth315

[39] Korf I Gene finding in novel genomes. BMC Bioinformatics. 2004;5:59. 15144565 10.1186/1471-2105-5-59 PMC421630

[40] Haas BJ, Salzberg SL, Zhu W Automated eukaryotic gene structure annotation using EVidenceModeler and the Program to Assemble Spliced Alignments. Genome Biol. 2008;9(1):R7. 18190707 10.1186/gb-2008-9-1-r7 PMC2395244

[41] Lowe TM, Eddy SR tRNAscan-SE: a program for improved detection of transfer RNA genes in genomic sequence. Nucleic Acids Res. 1997;25(5):955–64. 9023104 10.1093/nar/25.5.955 PMC146525

[42] Nawrocki EP, Kolbe DL, Eddy SR Infernal 1.0: inference of RNA alignments. Bioinformatics. 2009;25(10):1335–7. 19307242 10.1093/bioinformatics/btp157 PMC2732312

[43] Li Y-h, Zhou G, Ma J, De novo assembly of soybean wild relatives for pan-genome analysis of diversity and agronomic traits. Nat Biotechnol. 2014;32:1045. 25218520 10.1038/nbt.2979

[44] Apweiler R, Bairoch A, Wu CH, UniProt: the universal protein knowledgebase. Nucleic Acids Res. 2017;45(D1):D158–69. 27899622 10.1093/nar/gkw1099 PMC5210571

[45] Finn RD, Coggill P, Eberhardt RY The Pfam protein families database: towards a more sustainable future. Nucleic Acids Res. 2016;44(D1):D279–85. 26673716 10.1093/nar/gkv1344 PMC4702930

[46] The Gene Ontology Consortium. Expansion of the Gene Ontology knowledgebase and resources. Nucleic Acids Res. 2017;45(D1):D331–d338. 27899567 10.1093/nar/gkw1108 PMC5210579

[47] Kanehisa M, Goto S, Sato Y Data, information, knowledge and principle: back to metabolism in KEGG. Nucleic Acids Res. 2014;42(Database issue):D199–205. 24214961 10.1093/nar/gkt1076 PMC3965122

[48] Li L, Stoeckert CJ Jr., Roos DS OrthoMCL: identification of ortholog groups for eukaryotic genomes. Genome Res. 2003;13(9):2178–89. 12952885 10.1101/gr.1224503 PMC403725

[49] Edgar RC MUSCLE: multiple sequence alignment with high accuracy and high throughput. Nucleic Acids Res. 2004;32(5):1792–7. 15034147 10.1093/nar/gkh340 PMC390337

[50] Yang Z PAML: a program package for phylogenetic analysis by maximum likelihood. Comput Appl Biosci. 1997;13(5):555–6. 9367129 10.1093/bioinformatics/13.5.555

[51] Liu H, Liu Q, Chen Z Supporting data for “Draft genome of Glyptosternon maculatum, an endemic fish from Tibet-plateau.”. GigaScience Database. 2018 http://dx.doi.org/10.5524/100489. 10.1093/gigascience/giy104 PMC6136493 30124856

