# Supplementary material for: Draft genome of Glyptosternon maculatum, an endemic fish from Tibet Plateau
Source: Gigascience. 2018 Aug 14;7(9):giy104. doi: 10.1093/gigascience/giy104 (PMC6136493; doi:10.1093/gigascience/giy104)
Supplement: GIGA-D-18-00128_Revision_1.pdf [file giy104_giga-d-18-00128_revision_1.pdf]

GigaScience

Draft genome of Glyptosternon maculatum, an endemic fish from Tibet-plateau

--Manuscript Draft--

**Manuscript Number:**	GIGA-D-18-00128R1
**Full Title:**	Draft genome of Glyptosternon maculatum, an endemic fish from Tibet-plateau
**Article Type:**	Data Note
**Funding Information:**	the special finance of Tibet autonomous region (2017CZZX003) — Dr haiping liu<br>National Natural Science Foundation of China (31560144) — Dr haiping liu<br>National Natural Science Foundation of China (31602207) — Dr Shijun Xiao

Abstract:

Abstracts

Background

Mechanisms for high altitude adaption have arisen widespread interest to evolution biologists. Several genome wide studies have been carried out for endemic vertebrates in Tibet, including mammals, birds and amphibians. However, little information was known about the adaptive evolution of highland fishes. Glyptosternon maculatum (G. maculatum, Regan, 1905), also known as Regan or barkley, is a fish endemic to the Tibetan plateau, which belongs to Sisoridae family, Siluriformes (catfishes) order. This species live within an elevation ranging from roughly 2800 m to 4200 m. Hence, a high-quality reference genome of G. maculatum provides an opportunity to address high altitude adaption mechanisms of fishes.

Findings

To get a high-quality reference genome of G. maculatum, we combined PacBio single-molecule real-time sequencing, Illumina paired-end sequencing, 10X Genomics linked-reads and BioNano optical map techniques. In total, 603.99 Gb sequencing data were generated. The assembled genome was about 662.34 Mb with scaffold and contig N50 sizes of 20.90 Mb and 993.67 kb, respectively, which captured 83% complete and 3.9% partial vertebrate Benchmarking Universal Single-copy orthologs (BUSCO). Repetitive elements account for 35.88% of the genome, and 22,066 protein-coding genes were predicted from the genome, of which 91.7% have been functionally annotated.

Conclusions

We provide the first comprehensive de novo genome of the G. maculatum. This genetic resource is fundamental for investigating the origin of the G.maculatum and will improve our understanding of high altitude adaption of fishes. The assembled genome can also be used as reference for future population genetic studies of G. maculatum.

**Corresponding Author:**	haiping liu<br><br>CHINA
**Corresponding Author Secondary Information:**	
**Corresponding Author's Institution:**	
**Corresponding Author's Secondary Institution:**	
**First Author:**	haiping liu

Powered by Editorial Manager® and ProduXion Manager® from Aries Systems Corporation

**First Author Secondary Information:**	
**Order of Authors:**	haiping liu
	Qiyong Liu
	Zhiqiang Chen
	Yanchao Liu
	Chaowei Zhou
	Qiqi Liang
	Caixia Ma
	Jianshe Zhou
	Yingzi Pan
	Meiqun Chen
	wangjiu wangjiu
	Wenkai Jiang
	Shijun Xiao
	Zhenbo Mou
**Order of Authors Secondary Information:**	
**Response to Reviewers:**	Comments from the editor:<br>Comment: Please make sure the data is in the SRA and the accession is included in the paper before resubmission.<br>Reply: We have submit data used in this work to NCBI with SRA accession of SRR7279473-SRR7279474, SRR7268130-SRR7268162, SRR7350914-SRR7350921, SRR7351269-SRR7351265, SRR7403445-SRR7403454 under the Project accession number of PRJNA447978 for Illumina, PacBio, 10X Genomics and transcriptome sequencing data used in this work.<br><br>Comment: Please include a Fishbase ID with the NCBI taxon ID, and also please use the more up-to-date BUSCO v3 rather than BUSCO v2. We also recommend adding RRIDs for the software tools, see:<br>ftp://penguin.genomics.cn/pub/10.5524/RRID/RRIDlist.pdf<br>Reply: We have included the Fishbase ID and the NCBI taxon ID in the revised manuscript. For BUSCO version, we have double checked that the BUSCO v3 was used in our study. We are sorry for the mistake and corrected the description in our manuscript. We also have add the RRIDs of the software used in this work.<br><br>Reviewer reports:<br>Reviewer #2: This is a purely descriptive paper reporting the sequencing and genome annotation of Glyptosternon maculatum, an endemic catfish species from the Tibet plateau. This is a straightforward paper and a valuable resource, which deserves publication after minor revision.<br><br>- Was the fish individual sequenced a male or a female? Any hint of sex chromosomes in this species?<br>Reply: The fish individual used in this work was a female, and we have added the information in the manuscript. The sex determination and chromosome for the species were not identified so far, and no heterotropic chromosome was observed from the previous karyotype analysis (Wu Yunfei, Kang Bin, Men Qiang, et al. Chromosome diversity of tibetan fishes. Zoological Research, 1999, 20(4):258-264.) .<br><br>- It might be also of interest to predict long non-coding RNA genes (not presented in "all kinds" of non-coding RNA in Tab S5).<br>Reply: LncRNA is an important non-coding genes in gene expression regulation. However, the transcriptome used in this work were generated from poly-A based protocol, and it is no suitable for lncRNA prediction with the reference genome. Therefore, we did not annotate the lncRNA gene in this work. However, lncRNA

Powered by Editorial Manager® and ProduXion Manager® from Aries Systems Corporation

	regulation in high-altitude would be interesting direction, and related work will be performed in our following studies.<br><br>- To affirm that 228 genes are species-specific sounds always strange to me. More precise comparative analysis of their presence/absence in other (related) species should be performed to confirm this.<br>Reply: Thanks a lot for reviewer's suggestion. We further blast the 228 genes to NCBI NR (non-redundant) database, and found that 142 genes hit to database with e-value of 1e-5; however, there were still 86 genes failed to hit any protein sequences in the database. The function analysis of those genes is an interesting topic in our following studies.<br>We have corrected the term of "species-specific genes" to "genes without significant homologous hits" and added the additional analysis to the manuscript. The correction were highlighted by red.<br><br>- Repeats: did the authors check for the presence of MITE elements?<br>Reply: Thanks a lot for reviewer's suggestion. We checked for the presence of MITE elements using MITE-digger with default parameters. The results shown that there were 2,962 MITEs which account for 0.185 % of the whole genome. The resulted MITE sequences were attached as Supplemental_file_MITE to the revised manuscript.<br><br>- Species names should be italicized in Fig.2<br>Reply: We thanks reviewer for the reminding. Species names were italicized in Fig. 2.<br><br>- The paper should be edited for typos/grammatical errors<br>Reply: Thanks a lot for reviewer's suggestion. We have revised and corrected typos and grammatical errors through the manuscript. The corrections were highlighted by red.<br><br>- Tab S3: satellites are not interspersed repeat (correct title)<br>Reply: Thanks reviewer for the reminding. We have corrected the title in Table S3.
**Additional Information:**	
**Question**	**Response**
Are you submitting this manuscript to a special series or article collection?	No
**Experimental design and statistics**<br><br>Full details of the experimental design and statistical methods used should be given in the Methods section, as detailed in our Minimum Standards Reporting Checklist. Information essential to interpreting the data presented should be made available in the figure legends.<br><br>Have you included all the information requested in your manuscript?	Yes
**Resources**<br><br>A description of all resources used, including antibodies, cell lines, animals and software tools, with enough information to allow them to be uniquely identified, should be included in the Methods section. Authors are strongly encouraged to cite Research Resource	Yes

Identifiers (RRIDs) for antibodies, model organisms and tools, where possible.<br><br>Have you included the information requested as detailed in our Minimum Standards Reporting Checklist?	
**Availability of data and materials**<br><br>All datasets and code on which the conclusions of the paper rely must be either included in your submission or deposited in publicly available repositories (where available and ethically appropriate), referencing such data using a unique identifier in the references and in the “Availability of Data and Materials” section of your manuscript.<br><br>Have you have met the above requirement as detailed in our Minimum Standards Reporting Checklist?	Yes

Manuscript Click here to access/download;Manuscript;manuscript_r1.docx

Click here to view linked References

Draft genome of *Glyptosternon maculatum*, an endemic fish from Tibet-plateau

Haiping Liu[1,†], Qiyong Liu[1,†], Zhiqiang Chen[2,†], Yanchao Liu[1,†], Chaowei Zhou[1,†], Qiqi Liang[2], Caixia Ma[2], Jianshe Zhou[1], Yingzi Pan[1], Meiqun Chen[1], Wangjiu[1], Wenkai Jiang[2,*], Shijun Xiao[3,*], Zhenbo Mou[1,*]

[1] Institute of Fisheries Science, Tibet Academy of Agricultural and Animal Husbandry Sciences, Lhasa 850002, China

[2] Novogene Bioinformatics Institute, Beijing, China

[3] Department of Computer Science, Wuhan University of Technology, Wuhan, China

[*] Correspondence: Zhenbo Mou (mouzhenbo@163.com), Wenkai Jiang (jiangwenkai@novogene.com) and Shijun Xiao(shijun_xiao@163.com)

[†] These authors contributed equally to this work.

Abstracts

Background: Mechanisms for high altitude adaption have arisen widespread interest to evolution biologists. Several genome wide studies have been carried out for endemic vertebrates in Tibet, including mammals, birds and amphibians. However, little information was known about the adaptive evolution of highland fishes. *Glyptosternon maculatum* (*G. maculatum*, Regan, 1905), also known as Regan or barkley, is a fish endemic to the Tibetan plateau, which belongs to Sisoridae family, Siluriformes (catfishes) order. This species live within an elevation ranging from roughly 2800 m to 4200 m. Hence, a high-quality reference genome of *G. maculatum* provides an opportunity to address high altitude adaption mechanisms of fishes.

Findings: To get a high-quality reference genome of *G. maculatum,* we combined PacBio single-molecule real-time sequencing, Illumina paired-end sequencing, 10X Genomics linked-reads and BioNano optical map techniques. In total, 603.99 Gb sequencing data were generated. The assembled genome was about 662.34 Mb with scaffold and contig N50 sizes of 20.90 Mb and 993.67 kb, respectively, which captured 83% complete and 3.9% partial vertebrate Benchmarking Universal Single-copy orthologs (BUSCO). Repetitive elements account for 35.88% of the genome, and 22,066 protein-coding genes were predicted from the genome, of which 91.7% have been functionally annotated.

Conclusions: We provide the first comprehensive *de novo* genome of the *G. maculatum*. This genetic resource is fundamental for investigating the origin of the *G.maculatum* and will improve our understanding of high altitude adaption of fishes. The assembled genome can also be used as reference for future population genetic studies of *G. maculatum*.

Keywords: *Glyptosternon maculatum*; Genome assembly; Annotation; Phylogeny

Data description

Background information on *G. maculatum*

The *G. maculatum* (Regan, 1905) (NCBI Taxon ID: 175778, Figure 1a), called barkley in Tibetan language, is a species in the *Glyptosternum* genus, Sisoridae family, Siluriformes order, Teleostei infraclass. The Sisoridaes are the largest family of catfishes (Siluriformes) in China, consisting of 44 species divided into two natural groups, Glyptosternoids and non-Glyptosternoids [1, 2]. There are 8 Sisorids distributed in Yarlung Tsangpo (Brahmaputra) river. Of them, *G. maculatum* is the only one species that distributed at the middle section (Figure 1b). Specifically, it is distributed at Niyang tributary, Tangjia to Zhaxue segement of the Lhasa tributary and Xietongmen segment of Yarlung Tsangpo, which across an elevation ranging from roughly 2800 m to 4200 m [3].

The karyotype of *G. maculatum* is a debated topic. Ren and Cui [4] reported a result of 2n=48=28m+12sm+8st, NF=88, based on specimens sampled at Quxur, and speculated it to be the most specialized karyotype among all sisoridaes. Conversely, Wu et al. [5] reported a karyotype of 2n=48=22m+12sm+10st+6t, NF=80 sampled at Xigaze, while 2n=44 and 2n=42 results were also found. They compared it to other Sisorid karyotypes and concluded that the karyotype of *G. maculatum* was not the most specialized. The genome assembly of *G. maculatum* might provide a route to resolve these debates.

Glyptosternoid group fishes distributed broadly at the south and southeast drainages of Tibetan plateau, providing a good model to study the speciation process causing by the up-shift of Tibetan plateau. He *et al.*[1] conducted a cladistical analysis of Glyptosternoid group based on 60 bone features, and found Glyptosternoids formed a monophyletic group, of which the *Glyptosternum* were the most primitive clade. He *et al.*[6] further analyzed the phylogeny of Glyptosternoids using 19 species distributed in four genera by their bone features, in combination with biogeographical analysis, they postulated the rise of the Tibetan Plateau had a direct influence on the diversification of Glyptosternoids, with *Glyptosternum* (particularly *G. maculatum*) as the most primitive clade, which was consistent to the conclusion of Hora and Silas [2]. Peng *et al.*[7] sequenced mitochondrial cytochrome b (CYTB) from 13 Glyptosternoids, with results also supporting them to be a monophyletic group, of which *Glyptosternum* and *Exostoma* were relatively primitive clades. We thus choose *G. maculatum* as a represent of Glyptosternoid group fishes, its whole genome sequence would provide a foundation to explore the adaptive evolution process of highland fishes, also supplied as a starting point to study speciation mechanisms caused by rapid rising of Tibetan plateau.

G. maculatum had a specialized liver, which could be divided to two parts, one placed outside the abdominal cavity, connected to another part that located inside the cavity [8].

Several studies had also reported similar ectopic livers exist in other Sisords, suggesting that this specialized organ might be the result of adaptive evolution [9]. The genesis of livers in *G. maculatum* occurs in three stages: the ectopic liver is not present from the beginning till the end of the larva's exit from the egg envelope; a "bump" then develops, starting from day 17 till day 22; the ectopic liver appears starting from day 22 [9]. Zhang [9] pointed out the expressions of Cu-Zn SOD, Mn SOD, and CAT mRNA were all higher in the primary liver relative to the secondary liver, suggesting that the two livers have different physiological roles in *G. maculatum*. However, the molecular mechanism for the liver development and their physiological functions in adaptive evolution were not fully understand; therefore genome assembly of the species could lay a solid foundation for the following investigations.

Sample collection and sequencing

The female fish individual used for genome sequencing came from Angren, Xizang Province (Figure 1c). Total genomic DNA was extracted from muscular tissue and kept at Novogene Bioinformatics Institute.

A combination of four technologies was applied: PacBio's single-molecule real-time sequencing, Illumina's paired-end sequencing, 10X Genomics link-reads and BioNano optical maps. Two paired-end Illumina sequence libraries were constructed with an insert size of 250 bp, and sequencing was carried out on the Illumina HiSeq 4000 platform according to the manufacturer's instructions, of which 147.16 Gb (191x coverage) sequencing data were produced. In addition, one 10X Genomics linked-read library was constructed and sequencing on Illumina HiSeq 4000 platform, which produced 157 Gb (203.5x coverage) sequencing data. Raw sequence data generated by Illumina platform were filtered by these criteria: (a) filtered reads with adapters; (b) filtered reads with N bases more than 10%; (c) filtered reads with low-quality bases (<=5) more than 50%. Pacbio reads were sequenced by the Sequel platform, which gained 106.3 Gb (145.2x coverage) sequencing data. For the PacBio data, subreads were filtered with the default parameters. Finally, we obtained 106.32 Gb of long reads (polymerase reads) data. The average and the N50 length of long subreads reached 8.04 kb and 13.26 kb, respectively. An optical map was also constructed from Irys platform (BioNano Genomics), of which 191.3 Gb (248x coverage) data were generated. All these sequence data were summarized in Table 1.

De novo assembly of *G. maculatum* genome

The genome size was estimated based on the *k*-mer spectrum : $G = (K_{total} - K_{error})/D$, where K_{total} is the total count of *k*-mers, K_{error} is the total count of low-frequency (frequency ≤1) *k*-mers that were probably caused by sequencing errors, G is the genome size and D is the *k*-mer depth. Using Jellyfish [10] (v2.1.3), 17-mers were counted as 54,676,846,244

from short clean reads. The total count of error *k*-mers was 1,464,736,244 and the *k*-mer depth was 69 (Figure. S1). Therefore, the genome size of *G. maculatum* was estimated to be approximately 771.19 Mb.

The contig assembly of the *G. maculatum* genome was carried out using the FALCON assembler [11], followed by two rounds of polishing with Quiver [12]. FALCON implements a hierarchical assembly process, which include these steps: 1) subread error correction through aligning all reads to each other using daligner [13], the overlap data were then processed to generate error-corrected consensus reads; After error correction, we got 28 Gb (35x coverage) of error-corrected reads; 2) second round of overlap detection using error-corrected reads; 3) construction of a directed string graph from overlap data; 4) resolving contig path from the string graph. After FALCON assembly, the genome was polished by Quiver. Initial assembly of the PacBio data alone resulted in a contig N50 (the minimum length of contigs accounting for half of the haploid genome size) of 697.4 Kb. Then PacBio contigs were first scaffolded using optical map data, and the resulting scaffolds were further connected to super-scaffolds by 10X Genomics linked-read data using the fragScaff software [14]. Finally, we used Illumina-derived short reads to correct any remaining errors by pilon [15]. These processes yielded a final draft *G. maculatum* genome assembly with a total length of 662.34 Mb, contig N50 of 993.67 kb, and scaffold N50 of 20.90 Mb (Table 2).

To evaluate the accuracy of the genome at single base level, we mapped short sequence reads generated by Illumina platform to the *G. maculatum* genome with BWA (RRID:SCR_010910) [16] and performed variant calling with SAMtools (RRID:SCR_002105) [17]. We obtained a total of 3,632 homozygous SNPs (Table S2), reflecting a low homozygous rate (0.0007%) and a high accuracy of genome assembly at the single base level.

To assess the completeness of the assembled *G. maculatum* genome, we performed BUSCO (RRID:SCR_015008) analysis [18] by searching against the vertebrate universal benchmarking single-copy orthologs (BUSCOs, version 3.0). Overall, 83% complete and 3.9% partial of the 970 vertebrate BUSCOs were identified in the assembled genome. We also assessed the completeness of *G. maculatum* genome by CEGMA (Core Eukaryotic Genes Mapping Approach, RRID:SCR_015055) [19]. According to CEGMA, 211 (85.08%) conserved genes were identified in the *G. maculatum* genome. The muscle transcriptome *de novo* assembled by Trinity (RRID:SCR_013048) [20] were also mapped to the genome assembly using BLAT [21] with default parameters, showing that the alignment coverage of expressed sequences ranged from 75 to 99% in the genome assembly.

Annotation of repetitive sequences in *G. maculatum* genome

The repetitive sequences in *G. maculatum* genome were identified by a combination of

homology searching and *ab initio* prediction. For homology-based prediction, we used RepeatMasker (RRID:SCR_012954) [22] and RepeatProteinMask to search against Repbase. For *abinitio* prediction, we used Tandem Repeats Finder (TRF) [23], LTR_FINDER (RRID:SCR_015247) [24], PILER [25] and RepeatScout (RRID:SCR_014653) [26]with default parameters. We found that 33.96% of the *G. maculatum* assembly was composed of repetitive elements (Table S3 and Figure S2). Additionally, we predicted MITE elements through the genome using MITE-digger [27] with default parameters. As a result, we identified 2,962 MITEs accounting for 0.185% of the whole genome (Supplemental_file_MITE).

Protein coding gene prediction and ncRNA prediction

Gene prediction was conducted through a combination of homology-based prediction, *ab initio* prediction and transcriptome-based prediction methods. Protein repertoires of vertebrates including *Takifugu rubripes* (Tru, GCF_000180615.1), *Ctenopharyngodon idellus* (Cid) [28], *Cyprinus carpio* (Cca,GCF_000951615.1),*Danio rerio*(Dre, GCF_000002035.5), *Sinocyclocheilus graham* (Cav, GCF_001515645.1), channel catfish (Sas, GCF_001660625.1),*Homo sapiens* (Hom, GCF_000001405.37) and *Mus musculus* (Mmu, GCF_000001635.26) were used as queries to search against *G. maculatum* genome using TBLASTN (RRID:SCR_011822) [29]. The BLAST hits were conjoined by Solar software [30]. GeneWise (RRID:SCR_015054) [31] was used to predict the exact gene structure of the corresponding genomic region on each BLAST hit. Homology predictions were denoted as “Homology-set” (Table S4). RNA-seq data derived from 16 tissue which obtained about 77.29 Gb clean data were assembled by Trinity [20]. The Trinity assembly included 572,416 contigs with an average length of 1,075 bp. These assembled sequences were aligned against the *G. maculatum* genome by PASA (Program to Assemble Spliced Alignment). Valid transcript alignments were clustered based on genome mapping location and assembled into gene structures. Gene models created by PASA [32] were denoted as PASA-T-set (PASA Trinity set). Besides, RNA-seq reads were directly mapped to the genome using Tophat (RRID:SCR_013035) [33] to identify putative exon regions and splice junctions; Cufflinks (RRID:SCR_014597) [34] was then used to assemble the mapped reads into gene models (Cufflinks-set). Augustus (RRID:SCR_008417) [35], GeneID [36], GeneScan [37], GlimmerHMM (RRID:SCR_002654) [38], and SNAP [39] were also used to predict coding regions in the repeat-masked genome. Of these, Augustus, SNAP and GlimmerHMM were trained by PASA-H-set gene models. Gene models generated from all the methods were integrated by EvidenceModeler (EVM) [40]. Weights for each type of evidence were set as follows: PASA-T-set > Homology-set > Cufflinks set > Augustus > GeneID = SNAP = GlimmerHMM = GeneScan. The gene models were further updated by PASA2 to generate UTRs, alternative splicing variation information. In total, we haveidentified

22,066 protein coding genes with a mean of 8.5 exons per gene (Table 3). The lengths of genes, coding sequence (CDS), introns, and exons in *G. maculatum*were were comparable to those of close-related genomes (Table S4 and Figure S3). In addition, we predicted non-coding RNA genes inthe *G. maculatum* genome. The rRNA fragments were predicted by searching against Human rRNA database using BLAST with an E-value of 1E-10. The tRNA genes were identified by tRNAscan-SE (RRID:SCR_010835) software [41]. The miRNA and snRNA genes were predicted by INFERNAL (RRID:SCR_011809) [42] using Rfam database [43]. We found a total of 3,117 ribosomal RNA (rRNA), 3,512 transfer RNA (tRNA), 1,235 microRNAs (miRNA),and 781 snRNA genes in the *G. maculatum* genome (Table S5).

Functional annotation of protein-coding genes

Gene functional annotation refers to searching functional motifs, domains, and possible biological process of genes by aligning protein sequences to known databases such as SwissProt [44], Pfam [45], NR database (from NCBI), GeneOntology (GO) [46], and Kyoto Encyclopedia of Genes and Genomes (KEGG) [47]. In total, 20,234 protein-coding genes (91.7%) were successfully annotated forat least one function terms (Table S6, Figure S4).

Phylogenetic analysis and species divergence time estimation

To investigate the phylogenic position of *G. maculatum*, we retrieved nucleotide and proteindata for *Cyprinus carpio* (GCF_000951615.1), *Sinocyclocheilus rhinocerous* (GCF_001515625.1), *Sinocyclocheilus anshuiensis* (GCF_001515605.1), *Astyanax mexicanus* (GCF_000372685.2), *Pygocentrus nattereri* (GCF_001682695.1), *Sinocyclocheilus grahami* (GCF_001515645.1), *Ictalurus punctatus* (GCF_001660625.1), *Danio rerio* (GCF_000002035.6), *Amazon molly* (GCF_000485575.1), *Oreochromis niloticus* (GCF_001858045.1), *Takifugu rubripes* (GCF_000180615.1) and *Ctenopharyngodon idellus* [28] from public databases. To remove redundancy caused by alternative splicing variations, we retained only gene models at each gene locus that encoded the longest protein sequence. To exclude putative fragmented genes, genes encoding protein sequences shorter than 50 amino acids were filtered out. All-against-all BLASTP (RRID:SCR_001010) [29] was employed to identity the similarities among filtered protein sequences in these species with an E-value cut off of $1e^{-7}$. The OrthoMCL (RRID:SCR_007839) [48] method was used to cluster genes from these different species into gene families with the parameter of “-inflation 1.5”.

A total of 26,588 gene family clusters were constructed. There were101 gene families and 228 genes in *G. maculatum* without significant homologous hits to other teleosts. We further searched the 228 genes to NCBI NR database by BLASTP (RRID:SCR_001010) and found that 142 genes hit to database with e-value of 1e-5, and 86 genes still failed to hit any protein sequences in the database. The function of those genes lacking significant

homology is an interesting topic in the following studies.

Protein sequences from 247 single copy gene families were used for phylogenetic tree reconstruction. MUSCLE (RRID:SCR_011812) [49] was used to generate multiple sequence alignment for protein sequences in each single-copy family with default parameters. Then, the alignments of each family were concatenated to a super alignment matrix. The super alignment matrix was used for phylogenetic tree reconstruction through Maximum likelihood (ML) methods. Divergence time between species was estimated using MCMCtree in PAML [50] with the options ‘correlated molecular clock’ and ‘JC69’ model. A Markov Chain Monte Carlo analysis was run for 20,000 generations, using a burn-in of 1000 iterations. Divergence time for the common ancestor of *C. idellus*, *S. rhinocerous* and *P. nattereri* obtained from the TimeTree database (http://www.timetree.org/) was used as the calibrate point. These phylogenetic analyses indicated that *G. maculatum* diverged from the common ancestral of *I. punctatus* at approximately 48.3million years ago (Figure 2).

Conclusion

We have constructed a *de novo* assembly of the *G. maculatum* genome and describe its genetic attributes. To our knowledge, this is the first *de novo* genome for Glyptosternoid group fishes. The *G. maculatum* genome will support investigations concerning the origin and evolutionary history of Glyptosternoid. This resource was also important for the future conservation of this endangered plateau species. In addition, the *G. maculatum* genome supplied as a foundation to study high altitude adaption of fishes and the speciation process causing by the rising of Tibetan plateau.

Availability of supporting data

The raw sequencing and physical mapping data were deposited into The National Omics Data Encyclopedia (NODE) (http://www.biosino.org/node/index) with the project ID of OEP000007 (http://www.biosino.org/node/project/detail/OEP000007) and SRA accession of SRR7279473-SRR7279474, SRR7268130-SRR7268162, SRR7350914-SRR7350921, SRR7351269-SRR7351265, SRR7403445-SRR7403454 under the Project accession number of PRJNA447978 in NCBI. The genome, annotation and intermediate files were uploaded to GigaScience FTP server. All supplementary figures and tables are provided in Supplemental File.

Competing interests

All authors declare that they have no competing interests.

Authors’ contributions

Haiping Liu, Wenkai Jiang and Zhenbo Mou conceived the study. Haiping Liu and Wenkai Jiang designed the scientific objectives. Qiyong Liu and Zhenbo Mou managed the project; Yanchao Liu and Chaowei Zhou collected the samples and extracted the genomic DNA; Zhiqiang Chen estimated the genome size and assembled the genome; Qiqi Liang and Caixia Ma assessed the assembly quality; Jianshe Zhou and Yingzi Pan carried out the repeat annotation and gene annotation. Zhiqiang Chen carried out comparative genomics analysis, Haiping Liu, Shijun Xiao, Zhiqiang Chen and Wenkai Jiang wrote the manuscript. And all authors read, edited, and approved the final manuscript.

Acknowledgements

This work was supported by the special finance of Tibet autonomous region, Grant No. 2017CZZX003 and the National Natural Science Foundation of China (NSFC), Grant No. 31560144 and 31602207.Thanks should go to anonymous reviewers for their helpful comments and constructive suggestions.

References

1. He, S., The phylogeny of the glyptosternoid fishes (Teleostei: Siluriformes, Sisoridae). Cybium, 1996. **20**(2): p. 115-159.
2. Hora, S.L. and E.G. Silas, Evolution and distribution of Glyptosternoid fishes of the family Sisoridae (Order: Siluroidea). Proceedings of the National Institute of Sciences of India, 1952. **18**.
3. T, C.Q. and Z.B. S, Systematic Index of Fish Species in China. China Science Publishing, 1987.
4. Ren, X., The karyotype and haploidy NOR of Glyptosternum maculatum. Hereditas, 1992.
5. WU Yun-fei, K.B., MEN Qiang,WU Cui-zhen, Chromosome Diversity of Tibetan Fishes. Zoological Research, 1999. **20**(4): p. 258-264.
6. He, S., W. Cao, and Y. Chen, The uplift of Qinghai-Xizang (Tibet) Plateau and the vicariance speciation of glyptosternoid fishes (Siluriformes: Sisoridae). Science China 2001. **44**(6): p. 644.
7. Peng, Z., S. He, and Y. Zhang, Phylogenetic relationships of glyptosternoid fishes (Siluriformes: Sisoridae) inferred from mitochondrial cytochrome b gene sequences. Molecular Phylogenetics & Evolution, 2004. **31**(3): p. 979-987.
8. X, X.C., L.H. J, and L.D. P. *Special Organ in Glyptosternum maculatum:exo-celiac liver*. 2006.
9. Huijuan, Z., Genesis of Liver in Glyptosternum maculatum and Related Bioadaptive Studies, in Library of Huazhong Agricultural University. 2011, Huazhong Agricultural University: WuHan, China.
10. Marcais, G. and C. Kingsford, A fast, lock-free approach for efficient parallel counting of occurrences of k-mers. Bioinformatics, 2011. **27**(6): p. 764-70.
11. Pendleton, M., R. Sebra, A.W. Pang, et al., Assembly and diploid architecture of an individual human genome via single-molecule technologies. Nat Methods, 2015. **12**(8): p. 780-6.
12. Chin, C.S., D.H. Alexander, P. Marks, et al., Nonhybrid, finished microbial genome assemblies from long-read SMRT sequencing data. Nat Methods, 2013. **10**(6): p. 563-9.
13. Myers, G. *Efficient Local Alignment Discovery amongst Noisy Long Reads*. in *Algorithms in Bioinformatics*. 2014. Berlin, Heidelberg: Springer Berlin Heidelberg.
14. Adey, A., J.O. Kitzman, J.N. Burton, et al., In vitro, long-range sequence information for de novo

genome assembly via transposase contiguity. Genome Research, 2014. **24**(12): p. 2041-9.

15. Walker, B.J., T. Abeel, T. Shea, et al., Pilon: an integrated tool for comprehensive microbial variant detection and genome assembly improvement. PLoS One, 2014. **9**(11): p. e112963.
16. Li, H., *Aligning sequence reads, clone sequences and assembly contigs with BWA-MEM*. Vol. 1303. 2013.
17. Li, H., A statistical framework for SNP calling, mutation discovery, association mapping and population genetical parameter estimation from sequencing data. Bioinformatics, 2011. **27**(21): p. 2987-93.
18. Simao, F.A., R.M. Waterhouse, P. Ioannidis, et al., BUSCO: assessing genome assembly and annotation completeness with single-copy orthologs. Bioinformatics, 2015. **31**(19): p. 3210-2.
19. Parra, G., K. Bradnam, and I. Korf, CEGMA: a pipeline to accurately annotate core genes in eukaryotic genomes. Bioinformatics, 2007. **23**(9): p. 1061-1067.
20. Grabherr, M.G., B.J. Haas, M. Yassour, et al., Full-length transcriptome assembly from RNA-Seq data without a reference genome. Nat Biotechnol, 2011. **29**(7): p. 644-52.
21. Kent, W.J., BLAT--the BLAST-like alignment tool. Genome Res, 2002. **12**(4): p. 656-64.
22. Bergman, C.M. and H. Quesneville, Discovering and detecting transposable elements in genome sequences. Brief Bioinform, 2007. **8**(6): p. 382-92.
23. Benson, G., Tandem repeats finder: a program to analyze DNA sequences. Nucleic Acids Res, 1999. **27**(2): p. 573-80.
24. Xu, Z. and H. Wang, LTR_FINDER: an efficient tool for the prediction of full-length LTR retrotransposons. Nucleic Acids Res, 2007. **35**(Web Server issue): p. W265-8.
25. Edgar, R.C. and E.W. Myers, PILER: identification and classification of genomic repeats. Bioinformatics, 2005. **21 Suppl 1**: p. i152-8.
26. Price, A.L., N.C. Jones, and P.A. Pevzner, De novo identification of repeat families in large genomes. Bioinformatics, 2005. **21 Suppl 1**: p. i351-8.
27. Yang, G., MITE Digger, an efficient and accurate algorithm for genome wide discovery of miniature inverted repeat transposable elements. BMC Bioinformatics, 2013. **14**: p. 186.
28. Wang, Y., Y. Lu, Y. Zhang, et al., The draft genome of the grass carp (Ctenopharyngodon idellus) provides insights into its evolution and vegetarian adaptation. Nat Genet, 2015. **47**(6): p. 625-31.
29. Altschul, S.F., W. Gish, W. Miller, et al., Basic local alignment search tool. J Mol Biol, 1990. **215**(3): p. 403-10.
30. Yu, X.J., H.K. Zheng, J. Wang, et al., Detecting lineage-specific adaptive evolution of brain-expressed genes in human using rhesus macaque as outgroup. Genomics, 2006. **88**(6): p. 745-751.
31. Birney, E., M. Clamp, and R. Durbin, GeneWise and Genomewise. Genome Res, 2004. **14**(5): p. 988-95.
32. Haas, B.J., A.L. Delcher, S.M. Mount, et al., Improving the Arabidopsis genome annotation using maximal transcript alignment assemblies. Nucleic Acids Res, 2003. **31**(19): p. 5654-66.
33. Kim, D., G. Pertea, C. Trapnell, et al., TopHat2: accurate alignment of transcriptomes in the presence of insertions, deletions and gene fusions. Genome Biol, 2013. **14**(4): p. R36.
34. Trapnell, C., A. Roberts, L. Goff, et al., Differential gene and transcript expression analysis of RNA-seq experiments with TopHat and Cufflinks. Nat Protoc, 2012. **7**(3): p. 562-78.
35. Stanke, M. and S. Waack, Gene prediction with a hidden Markov model and a new intron submodel. Bioinformatics, 2003. **19 Suppl 2**: p. ii215-25.

36. Guigo, R., Assembling genes from predicted exons in linear time with dynamic programming. J Comput Biol, 1998. **5**(4): p. 681-702.
37. Burge, C. and S. Karlin, Prediction of complete gene structures in human genomic DNA. J Mol Biol, 1997. **268**(1): p. 78-94.
38. Majoros, W.H., M. Pertea, and S.L. Salzberg, TigrScan and GlimmerHMM: two open source ab initio eukaryotic gene-finders. Bioinformatics, 2004. **20**(16): p. 2878-9.
39. Korf, I., Gene finding in novel genomes. BMC Bioinformatics, 2004. **5**: p. 59.
40. Haas, B.J., S.L. Salzberg, W. Zhu, et al., Automated eukaryotic gene structure annotation using EVidenceModeler and the Program to Assemble Spliced Alignments. Genome Biol, 2008. **9**(1): p. R7.
41. Lowe, T.M. and S.R. Eddy, tRNAscan-SE: a program for improved detection of transfer RNA genes in genomic sequence. Nucleic Acids Res, 1997. **25**(5): p. 955-64.
42. Nawrocki, E.P., D.L. Kolbe, and S.R. Eddy, Infernal 1.0: inference of RNA alignments. Bioinformatics, 2009. **25**(10): p. 1335-7.
43. Li, Y.-h., G. Zhou, J. Ma, et al., De novo assembly of soybean wild relatives for pan-genome analysis of diversity and agronomic traits. Nature Biotechnology, 2014. **32**: p. 1045.
44. UniProt: the universal protein knowledgebase. Nucleic Acids Research, 2017. **45**(D1): p. D158-D169.
45. Finn, R.D., P. Coggill, R.Y. Eberhardt, et al., The Pfam protein families database: towards a more sustainable future. Nucleic Acids Res, 2016. **44**(D1): p. D279-85.
46. Expansion of the Gene Ontology knowledgebase and resources. Nucleic Acids Res, 2017. **45**(D1): p. D331-d338.
47. Kanehisa, M., S. Goto, Y. Sato, et al., Data, information, knowledge and principle: back to metabolism in KEGG. Nucleic Acids Res, 2014. **42**(Database issue): p. D199-205.
48. Li, L., C.J. Stoeckert, Jr., and D.S. Roos, OrthoMCL: identification of ortholog groups for eukaryotic genomes. Genome Res, 2003. **13**(9): p. 2178-89.
49. Edgar, R.C., MUSCLE: multiple sequence alignment with high accuracy and high throughput. Nucleic Acids Res, 2004. **32**(5): p. 1792-7.
50. Yang, Z., PAML: a program package for phylogenetic analysis by maximum likelihood. Comput Appl Biosci, 1997. **13**(5): p. 555-6.

Table 1. Sequencing data used for the *G. maculatum* genome assembly. The coverage was calculated using an estimated genome size of 771.19 Mb.

Pair-end libraries	Insert size (bp)	Raw data (Gb)	Clean data (Gb)	Read length (bp)	Sequence coverage (X)
**Illumina reads**	250 bp	148.16	147.16	150	191
**Pacbio reads**	20 Kb	106.32	106.05	11,745	145.2
**10X Genomics**	500 bp	157.21	157.02	150	203.5
**BioNano**	--	192.30	191.30	--	248
**Total**	--	603.99	601.53	--	787.7

Back to main body

Table 2. Assembly statistics of *G. maculatum*

Sample ID	Length		Number	
	Contig** (bp)	Scaffold (bp)	Contig**	Scaffold
**Total**	637,133,884	662,339,741	3,281	531
**Max**	5,772,991	47,179,384	-	-
**Number>=2000**	-	-	3,161	531
**N50**	993,673	20,902,354	161	11
**N60**	668,112	17,328,106	239	14
**N70**	418,057	12,288,896	359	19
**N80**	211,596	6,320,921	575	27
**N90**	77,392	1,017,220	1,067	50

** Contig after scaffolding

Back to main body

Table 3. General statistics of predicted protein-coding genes

Gene set		Number	Average transcript length (bp)	Average CDS length (bp)	Average exons per gene	Average exon length (bp)	Average intron length (bp)
De novo	Augustus	14,910	9,534	1,241	6.93	179	1,399
	GlimmerHMM	73,320	7,896	574	3.87	148	2,551
	SNAP	43,247	15,950	847	6.04	140	2,996
	Geneid	23,523	16,924	1,323	6.29	210	2,948
	Genscan	24,037	19,024	1,514	8.14	186	2,451
Homolog	*Cav*	32,364	6,413	1,142	5.12	223	1,279
	Cca	27,208	6,326	1,252	5.36	234	1,165
	Cid	30,336	5,615	1,048	4.87	215	1,181
	Dre	19,458	9,935	1,507	7.58	199	1,280
	Hom	16,090	10,844	1,432	7.83	183	1,379
	Tru	23,120	8,191	1,225	6.12	200	1,362
	Mmu	16,164	10,803	1,417	7.74	183	1,392
	Sas	37,610	6,704	1,155	5.22	221	1,315
RNASeq	PASA	97,309	9,419	1,201	7.09	169	1,348
	Cufflinks	92,180	19,478	4,707	10.13	465	1,618
EVM		25,365	11,517	1,323	7.66	173	1,531
PASA-update*		38,086	13,009	1,521	8.79	173	1,475
Final set*		22,066	12,913	1,458	8.48	172	1,531

back to main body

Figure 1. A picture showing about *G. maculatum*. (a) The appearance of *G. maculatum*. (b) Distributed localization (red triangle) of *G. maculatum* for sequencing. (c) The liver of *G. maculatum* was divided to two parts, one placed outside the abdominal cavity (attaching liver), connected to another part that located inside the cavity (mail liver) (Figure schematic drawings (ventral view) of *G.maculatum* (imaged from Zhang[9]).

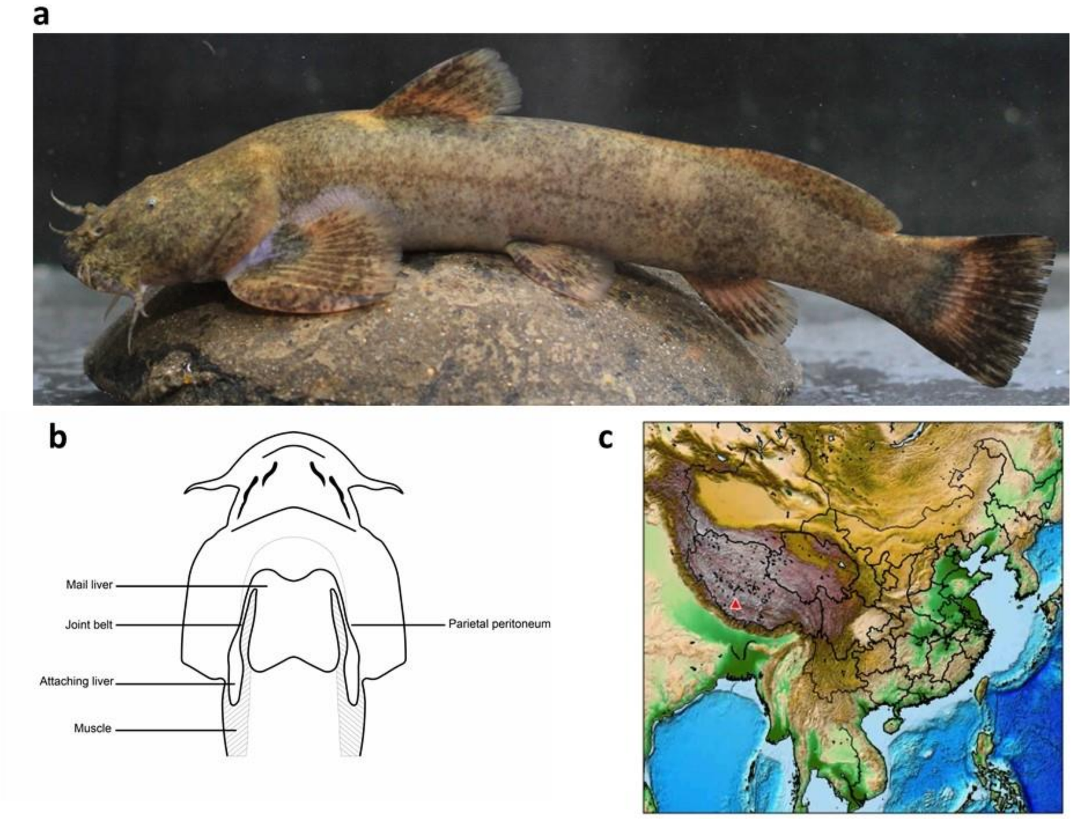

Figure 2. Divergence time estimated between *G. maculatum* and other species

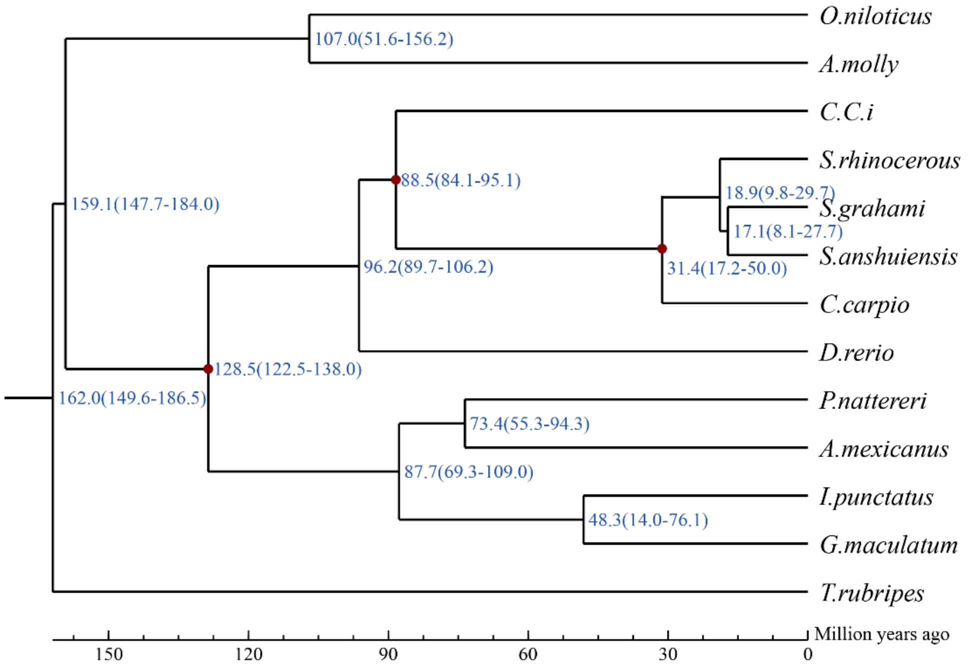

back to main body

Supplementary Figures and Tables

Click here to access/download
Supplementary Material
Supplemental_file.docx

Supplementary MITE annotation

Click here to access/download
Supplementary Material
Supplemental_file_MITE.docx
