# Supplementary material for: Draft genome of Glyptosternon maculatum, an endemic fish from Tibet Plateau
Source: Gigascience. 2018 Aug 14;7(9):giy104. doi: 10.1093/gigascience/giy104 (PMC6136493; doi:10.1093/gigascience/giy104)

Supplementary Tables and Figures for

Draft genome of *Glyptosternon maculatum*, an endemic fish from Tibet-plateau

**TableS1. Statistics of 17-mer analysis**

| **Kmer** | **Kmer_num** | **Peak depth** | **genome_size** | **used_base** | **used_read** | **X** |
| --- | --- | --- | --- | --- | --- | --- |
| 17 | 79702571328 | 25.0399 | 3155773450 | 203,652,243,867 | 2,167,240,756 | 27.7199 |

All 17-mer sequences were extracted from paired-end clean reads that passed quality control (QC) fromNext-generation sequencing libraries (250 bp), and the frequency of each 17-mer was calculated and plotted.

**TableS2. Statistics of variants calling**

|  | Number | Percentage（%） |
| --- | --- | --- |
| All SNP | 202,751 | 0.0371 |
| Heterozygosis SNP | 199,119 | 0.0364 |
| HomologySNP | 3,632 | 0.0007 |

**Table S3. Summary statistics of repeat annotation in** ***Glyptosternon maculatum***

|  | Denovo+Repbase | | TE Proteins | | Combined TEs | |
| --- | --- | --- | --- | --- | --- | --- |
|  | Length  (bp) | %in Genome | Length  (bp) | % in Genome | Length  (bp) | % in Genome |
| DNA | 22235383 | 3.36 | 7267878 | 1.10 | 27514536 | 4.15 |
| LINE | 138798262 | 20.96 | 45426510 | 6.86 | 150571072 | 22.73 |
| SINE | 3758352 | 0.57 | 0 | 0 | 3758352 | 0.57 |
| LTR | 59975525 | 9.06 | 14983444 | 2.26 | 66624256 | 10.06 |
| Satellite | 1570534 | 0.24 | 0 | 0 | 1570534 | 0.24 |
| Simple_repeat | 7178800 | 1.08 | 0 | 0 | 7178800 | 1.08 |
| Unknown | 6129271 | 0.93 | 0 | 0 | 6129271 | 0.93 |
| Total | 215848259 | 32.59 | 67515740 | 10.19 | 224921133 | 33.96 |

Note: Denovo+Repbase: the result of RepeatMasker based on Repbase, *RepeatModeler, RepeatScout and LTR_FINDER*; TE proteins: the result of RepeatProteinMask based on Repbase; Combined TEs: combine the results of Denovo+Repbase and TE proteins.

**Table S4. General statistics of for the genomes used by homolog-based method**

| Species | Number | Average transcript length (bp) | Average CDS length (bp) | Average exons per gene | Average exon length (bp) | Average intron length (bp) |
| --- | --- | --- | --- | --- | --- | --- |
| Cca | 49264 | 11719.03 | 1260.28 | 7.69 | 163.99 | 1564.5 |
| Ipu | 22996 | 17770.79 | 1759.38 | 10.29 | 170.96 | 1723.27 |
| Cid | 32811 | 10444.53 | 1384.98 | 7.65 | 180.99 | 1361.89 |
| Dre | 25619 | 25207.59 | 1642.64 | 9.42 | 174.39 | 2798.97 |
| Hom | 19926 | 50501.84 | 1725.4 | 9.89 | 174.44 | 5485.83 |
| Mmu | 22278 | 37435.55 | 1600.64 | 8.93 | 179.14 | 4516.11 |
| Sga | 45816 | 16267.5 | 1588.18 | 9.25 | 171.71 | 1779.44 |
| Tru | 18523 | 7492.75 | 1693.53 | 11.1 | 152.61 | 574.33 |

**Note:** *Takifugu rubripes*(Tru), *Ctenopharyngodon idellus* (Cid), *Cyprinus carpio* (Cca), *Danio rerio* (Dre), *Sinocyclocheilus graham* (Sga), channel catfish (Ipu), *Homo sapiens* (Hom) and *Mus musculus* (Mmu)

**Table S5. Number of all kinds of non-coding RNA**

| Type | | Copy(w*) | Average length (bp) | Total length (bp) | % of genome |
| --- | --- | --- | --- | --- | --- |
| miRNA | | 1235 | 110.715 | 136733 | 0.020644 |
| tRNA | | 3512 | 74.44562 | 261453 | 0.039474 |
| rRNA | **rRNA** | 3117 | 119.8447 | 373556 | 0.056399 |
|  | 18S | 48 | 282.1458 | 13543 | 0.002045 |
|  | 28S | 57 | 209.5965 | 11947 | 0.001804 |
|  | 5.8S | 11 | 147.1818 | 1619 | 0.000244 |
|  | 5S | 3001 | 115.4439 | 346447 | 0.052307 |
| **snRNA** | **snRNA** | 781 | 140.9334 | 110069 | 0.016618 |
|  | CD-box | 104 | 93.13462 | 9686 | 0.001462 |
|  | HACA-box | 97 | 159.4742 | 15469 | 0.002336 |
|  | splicing | 548 | 145.4945 | 79731 | 0.012038 |

Note: w*, whole genome annotation

**Table S6. Number of genes with homology or functional classification by each method**

|  | Number | Percent(%) |
| --- | --- | --- |
| NR | 20038 | 90.8 |
| Swiss-Prot | 19105 | 86.6 |
| KEGG | 17374 | 78.7 |
| InterPro | 18934 | 85.8 |
| Pfam | 17085 | 77.4 |
| GO | 14007 | 63.5 |
| Annotated | 20234 | 91.7 |
| Total | 22066 | - |

**Table S7. mRNA sequencing reads blasted to NT database.** Only the sources of the top 5 hits were shown. The number in parenthesis represented the percentage of hit numbers.

| **Sample** | **Hit1** | **Hit2** | **Hit3** | **Hit4** | **Hit5** |
| --- | --- | --- | --- | --- | --- |
| RRA61713-S | Ictalurus punctatus ribosomal (0.0038) | Ictalurus punctatus 40S (0.0028) | Ictalurus furcatus clone (0.002) | Zebrafish DNA sequence (0.0015) | Wallago attu voucher (0.0015) |
| RRA61707-S | Ictalurus punctatus ribosomal (0.0125) | Ictalurus punctatus 40S (0.0087) | Ictalurus punctatus clone (0.0043) | Glyptosternon maculatum voucher (0.0042) | Ictalurus furcatus clone (0.0041) |
| RRA61712-S | Ictalurus furcatus clone (0.0027) | Ictalurus punctatus 40S (0.0027) | Glyptosternon maculatum voucher (0.0026) | Ictalurus punctatus ribosomal (0.0026) | Ictalurus punctatus clone (0.0015) |
| RRA61708-S-W | Glyptosternon maculatum voucher (0.0151) | Glyptosternon maculatum cytochrome (0.0059) | Rita rita voucher (0.0038) | Ompok pabo voucher (0.0036) | Ompok pabda voucher (0.0034) |
| RRA61704-S-W | Ictalurus punctatus ribosomal (0.0173) | Ictalurus punctatus 40S (0.0162) | Glyptosternon maculatum voucher (0.0065) | Ictalurus furcatus clone (0.0056) | Ictalurus punctatus clone (0.0054) |
| RRA61705-S | Ictalurus punctatus ribosomal (0.0138) | Ictalurus punctatus 40S (0.0092) | Ictalurus furcatus clone (0.0041) | Glyptosternon maculatum voucher (0.004) | Ictalurus punctatus clone (0.0038) |
| RRA61706-S | Ictalurus punctatus ribosomal (0.0096) | Glyptosternon maculatum voucher (0.0082) | Ictalurus punctatus 40S (0.0073) | Ictalurus furcatus clone (0.0046) | Ictalurus punctatus clone (0.0041) |
| RRA61709-S | Ictalurus punctatus ribosomal (0.0097) | Ictalurus punctatus 40S (0.0052) | Ictalurus furcatus clone (0.0043) | Glyptosternon maculatum voucher (0.0041) | Ictalurus punctatus clone (0.0027) |
| RRA61710-S | Glyptosternon maculatum voucher (0.0066) | Ictalurus punctatus 40S (0.0052) | Ictalurus punctatus ribosomal (0.0049) | Ictalurus furcatus clone (0.0022) | Glyptosternon maculatum cytochrome (0.0017) |
| RRA61711-S | Glyptosternon maculatum voucher (0.0068) | Ictalurus punctatus ribosomal (0.0029) | Glyptosternon maculatum cytochrome (0.0024) | Glyptosternon maculatum mitochondrial (0.002) | Ictalurus punctatus 40S (0.0019) |

**Figure S1. 17-mer frequency distribution in *Glyptosternon maculatum* genomes.**


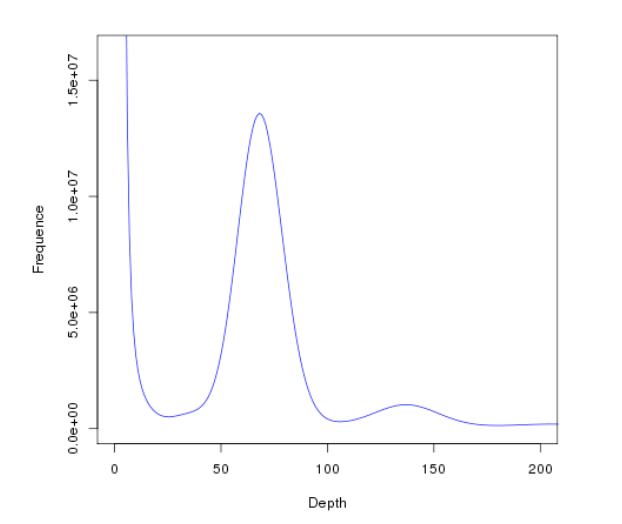


**Figure S2. Distribution of Divergence Rate of each Type of *Glyptosternon maculatum*’s TE (RepeatMasker). The divergence rate was calculated between the identified TE elements in the genome by homology-based method and the consensus sequence in the Repbase.**


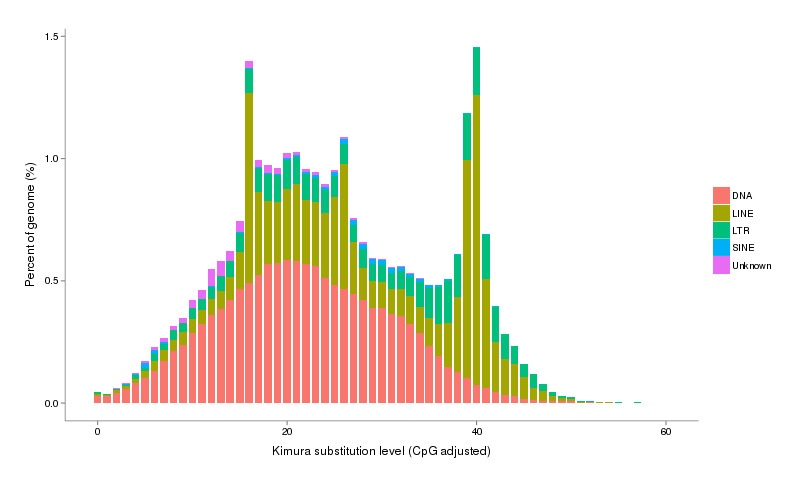


**Figure S3. Comparisons of gene parameters of the genomes used by homolog-based method**

**
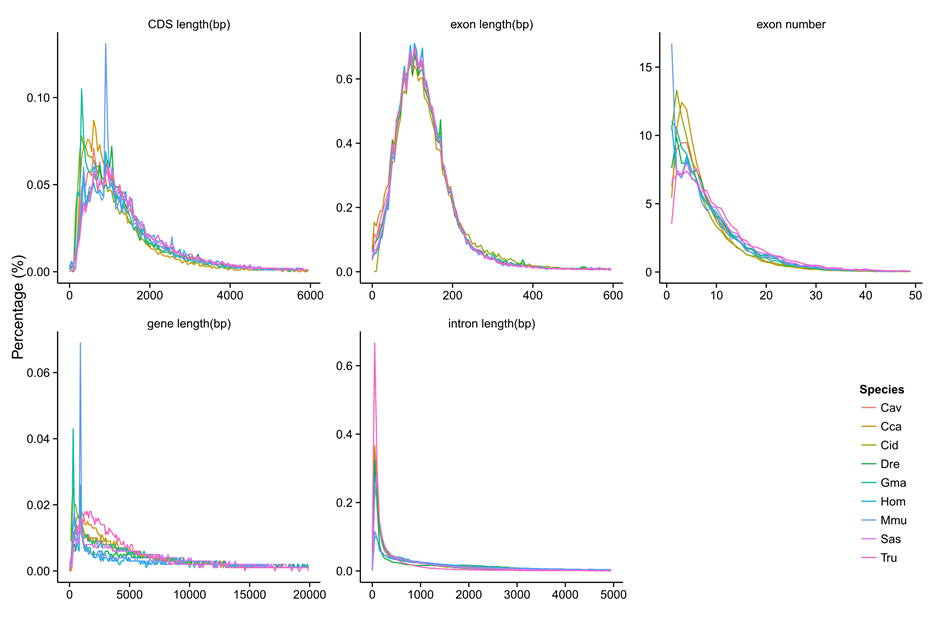
**

**Note:** *Takifugu rubripes*(Tru), *Ctenopharyngodon idellus* (Cid), *Cyprinus carpio* (Cca),*Danio rerio*(Dre), *Sinocyclocheilus graham* (Cav), channel catfish (Sas),*Homo sapiens* (Hom) and *Mus musculus* (Mmu), *Glyptosternon maculatum*(Gma)

**Figure S4. Venn diagram of number of genes with homology or functional classification by each method**


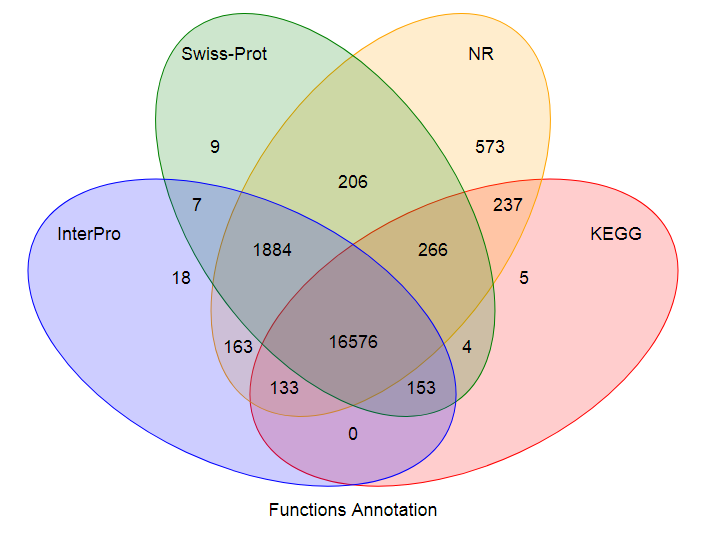

Supplement: Supplemental Files [file giy104_supplemental_files.zip › Supplemental_file.docx]
