# Supplementary material for: Draft genome of Glyptosternon maculatum, an endemic fish from Tibet Plateau
Source: Gigascience. 2018 Aug 14;7(9):giy104. doi: 10.1093/gigascience/giy104 (PMC6136493; doi:10.1093/gigascience/giy104)
Supplement: Supplemental Files [file giy104_supplemental_files.zip › Supplemental_file_MITE.docx]

>scaffold_40000-2

CTATTCGCACGGGATTAGTATTATCTGGGATTCTCTGGTTATGTGGAATAATTGTGGAGGTTGTCTGTGTGAATCGGCCATGTCTGTAATTAGTAAAGTAAACATTCCCCCGTAAATGATCTACCATATTTCTCCGAACACAGAGGTCATGTGATAATATTAATCCCGTGTGAATCGACATCTCTGTGATTTGGTCAGGATGTGTGGGGTCGTGTATCACTAATTAAGGTTTGTGTTTCCTGTGTGTGAAGAATAAGTGTGTGTTGGAGTGTTTGGGTGTATTTTGGGTGCTGCATAATCGTTATACACCAAAACCACCATACAACTTTCAGAAAGATCAAACTGACCACCAGCAGAAGAGCCAAAACTAAAAGAATGATTAGATGGAGATAAATGACGTATAACGGCATGCGGTAATGAACATTCTTCTGTTTATGATATATCATACATCTATACCCATAGAACCACATGATATTTCCTAACAATAGCCTATCCAATGCTGTGACGTCATATCCGGGAGATAAATCAGTAAATTCAAGTAAAATCACAGGCTTCGTTTATCCAGTGCGAATGCGCCACACAAAATTCAGATGTGGGGGTAATTTCTAACTCACACGAGGTCCCCAGTTAAAACTAATCCCGTGCGAATAG

>scaffold_400001-2

CTATTCGCACGGGATTAGTTTTATCTGGGGACCTCGTGTGATTTAGAACCCCCACATCTGAATTTTGTGTGGCGCATTCGCACTGGATAAACGAAGCCTGTGATTTTACTTGAATTTACTGATTTATCTCCCGGATATGACGTCACAGCATTGGATAGGCTATTGTTAGGAAATATCATGTGGTTCTATGGGTATAGATGTATGATATATCATAAACAGAAGAATGTTCATTACCGCATGCCGTTATACATGTCATTTATCTCCATCTAATCATTCTTTTAGTTTTGGCTCTTCTGCTGGTGGTCAGTTTGATCTTTCTGAAAGTTGTATGGTGGTTTTGGTGTATAACGATTATGCAGCACCCAAAATACACCCAAACACTCCAACACACACTTATTCTTCACACACAGGAAACACAAACCTTAATTAGTGATACACGACCCCACACATCCTGACCAAATCACAGAGATGTCGATTCACACGGGATTAATATTATCACATGACCTCTGTGTTCGGAGAAATATGGTAGATCATTTACGGGGGAATGTTTACTTTACTAATTACAGACATGGCCGATTCACACAGACAACCTCCACAATTATTCCACATAACCAGAGAATCCCAGATAATACTAATCCCGTGCGAATAG

>scaffold_400003-4

CTATTCGCACGGGATTAGTATTATCTGGGATTCTCTGGTTATGTGGAATAATTGTGGAGGTTGTCTGTGTGAATCGGCCATGTCTGTAATTAGTAAAGTAAACATTCCCCCGTAAATGATCTACCATATTTCTCCGAACACAGAGGTCATGTGATAATATTAATCCCGTGTGAATCGACATCTCTGTGATTTGGTCAGGATGTGTGGGGTCGTGTATCACTAATTAAGGTTTGTGTTTCCTGTGTGTGAAGAATAAGTGTGTGTTGGAGTGTTTGGGTGTATTTTGGGTGCTGCATAATCGTTATACACCAAAACCACCATACAACTTTCAGAAAGATCAAACTGACCACCAGCAGAAGAGCAAACTAAAAGAATGATTAGATGGAGATAATGACGTATAACGGCATGCGGTAATGAACATTCTTCTGTTTATGATATATCATACATCTATACCCATAGAACCACATGATATTTCCTACAATAGCCTATCCAATGCTGTGACGTCATATCCGGGAGATAAATCAGTAAATCAAGTAAAATCACAGGCGTCGTTTATCCAGTGCGAATGCGCCACACAAAATTTCAGTGTGGGGGTAATTTCTAACTCACACGAGGTCCCCAGTTAAAAACTAATCCCGTGCGAATAG

>scaffold_400003-32

TTAGGGCTGCACGATACTGGGGAAATATACAATATTATTGTTGAGTATTGCGATAACGATATTTCTTGCGATATAATATTCCCCAAGAGAAATGCTATTTTTATTAGCTATTTTAGCTGCAGGTGTTTTTCAGGCTGGTTCATCTTATCATGATTATCTCGCTATGAAAGCGTCGCAGTTTGTCGATCGTCTTGCGTCAAATCACGCGCACGCAAATCCCAACTAACATCCGCGTACCGGATATTTTACTGTCCTAAAACTTTAACCACACATTAATATTTAAAGTGTTAAATCGATCATTTATCGCAAACTATTACCATGTGCACATTTGTGATAATCCGATATATTGTGCAGCTCTAA

>scaffold_800001-1

ACTATATGGCCCAAAGTTTATGGACACCTCACCAGCACACCAACGTCAGTCTGTAATCACACGGTCGACTATAACGTCTCTGTATGTTGTATCTTTCCGTTCTTCCTTCACTGGAACTGCGAGACTCGAACCCTGTTCCAGCATGACAATGCCCCTGTGCACAAAGCGCCTGAGCTCCATGAAGACATGCTGTGTTCTGGTTGGAGTGGAAGAACTCTCATGTTCTGCACAGAGCCCTGACTCTGACTCAACCCCACTGAACCCCTTTGGGATGAACTGGAACCCCGACTGCCCCCCAGACCTCCTCCCCCAACATTAGTGTCTGATCTCATTAATACTATTGTAGCTGAATGATCAAATCCCCACAGTCACATGACCCAATCTAGTGGAAAACCTGCACAGAAGTCTGGAGGTGATTATAGGAGGAAGAGAGAGACTAACATCTGGAATAGGATGTGAAAAATATTCAGATGGATGGGATGGGCAGGTGTCCCAATACTTTGGCCATATAGT

>scaffold_900005-5

TAGGGCTGCACAATATATCGAAATTATCGAAATATCGCAAATGTGCATATGTCAATTGTTTGCGATAAATGACCGATTTAATACTTCAAATAGTAACGTGTGGTCAAAGTTTCAGAATAGTAAAATATCTGGTACGCGGATGTTAGTTGGGATTTGCGTGCGCGTGTTTTGACGCAAGACAATCGACAAACTGCGAAGCTTTCATAGCGAGCTAATCTTAATAAGATGAAGCAGCCCGAAAAACACGCGCAGCTAAAATAGCTAATAAAAATAGCATTTCTCTAGGGAAATGTTATATCGCAAGAAATATTGTTATCGTAATACTCAACAACAATATCGCATATTTCCCCCGTATCGTGCAGCCCTA

>scaffold_900006-1

TTAAAGGTAGGGTAGGAGATTTCGGAGAGGCTAGCAATAGCAAGCTAGCATTGAAAACCAAAGATCCTGCCCTCCCTGCATAATCACTCTGCAAAGCCACGCCTCCTCCAAAACACATGAACGCGCACAGGCACGCTCTTTGCTGATCTAAATCTTTTTTAGACATCTTCTTTCTAACACTGTTTTTCTCATGCGATTGTCTGTCACTTTTAGAGGCAATGTTGGGTTCTTGCTGTTTGCCTTCCATTTCCGCTCAGTCTGCATAGCGGGAACGCGCTAATGACATATTCTGTCTGCGAACAGGGTGCGTAGAGGTATGCAAATACATATTTGACAAGCAGGTAGGATAGCCTATCGTAATGTTTGGACCGAATATTTTGATTGGATGAAGTTGTTTTGGTCCTACGCCTTCCACAGACGATATAAATACATATAAATACATTTAGACCAGTAAACGTAATGAGTGCTATCGAGATGTGAAGAAACTTTCAACCCGCAGAACAAAAAATGTTTTGGAACCAAATCACCTACCCTGCCTTTAA

>scaffold_900007-9

TAGGGCTGTGTATTAGCAAGGGCCTCCCGAAACGATACATATCACGATACATATCACGATACATGGGTCACTATAGAATATATCACGATATATTGCAATACTTAAAAAAACTAAAACAAATTTAAAACAAATAGCTTGCTGTGTGCCACCTGGTGGATTCTAGTATGTGTATCACATGATATTGATTACTCAGCAAAGAAATAAAAACTGAGGGTTTTAAATAATTCAATATTAACGTAAACGAAAATCAGTTTAAAACGTCATTTAAATATGTATCAATACTAGAGGTTAGAATATCGATACACTATCGTGAAAAAAATATTGCGATAGTTAGCTGTATCGATATTTTTACACAGCCCTA

>scaffold_900008-3

CACTATATTCCCAAAAGTTTGTGGACACCTCACCATCAGATCCATATGTGGTTCCTCTCCAAAATGTTGCTACAAATTTGGAGTCACACAGTCGTCTAGAATGTCTTTGTATGTTGTAGTATTACATTATTCCTTCACTGGAACTAAGAGACCCAAACACTGTTCCAGCATGACAATGCCCCTGTGCACAAAGCACCTGAGCTCCATTAACACATGCTGTGTTCTGATTGGAGGGGAAAAACTTGAGTGTTCTGCACGAAGCCCTGACTTTAACACCACTGAACACCTTTGGGATGAACTGGAACGCCGACTGCACCCCAGACCTCCTCACCTGACGTTAGTGTCTGATCTCGCGAATACTATTGTAGCTGAATGAACACAAATCTCACATGCAAGCTCCACATTGTAGTGGAAAATCTTACTAGAAGACTGGAGCTTAATCGAACAGCAAAATGGGAGTAAATCTGGAATAAGATGTTTCAAATCACATGTTTATGATGATCAGGTGTCCACAAACTTTTGGCAATATAGTG

>scaffold_900008-11

TAGGGCTGTGTATTGGCAAGGGCCTCAAGATACGATAACATATCACGATACAATATATCCCAATATATCGCAATACTTAAAAAAAAAAAAAAAAAGGAAAACAAATAGCTAAAAATACATTTTGCTGTGTAGCACCTGGAGGATTTTAGTATGTGTATCACATGATATTGTTTACTCAGCAGTGAAATAAAAACTGAGGGTTATAATGAAATATTAACGTAAACGTAAATCAGTTTCAAACGTCATTTAAATATGTATCGATACTAGAGGTTAGAATATCGATACACTATCGTGAAAAAAATATTGCGATAGTTAGCTGTATCGATATTTTTACACAGCCCTA

>scaffold_900008-16

GAATTAGGGCTGAACAAAATATGGAAATTATCGAAATATCACAAATGAGAATATTGAGATATGCATGTTGCAACGGTTTGCGATAAATGAGCAATTTAACACTTCAAATATTAATGTGTGGTCAAAGTTTTAGGATAGTAAAATATCTGGCGCGCGGATGTTAGTTGGGATTTGCATGCGCGTGATTTGACGCAAGACGATCAACAAACTGCGACGCTTTCATAGCGAGATAATCGTGATAAGATGAACCAGCCGGAAAAACACCCGCAGCTAAAATAGCTAATAAAAATAGCATTTCTCTAGGGAAATATTATATCGCAAGAAATATCGTTATCGCAATACTCAACAACAATATCGCAACTCGCAGATTGTTCCAGTGTCGTGCAGCCCTAATTC

>scaffold_900009-11

AGCTCAGTGGTTAAGGTGTTGGCCTGCTGACTGGAAGATCATGGGTTCAATTCTCAGCACCACCAAGCTGCCACTGCTGGGCCCCTGAGCAAGGCCCTTAACCACTTAGCT

>scaffold_900009-14

TAGGGCTGTGTAAATATATCGATACAGATGACTATCGTGATATTTTTTTCACGATAATGTATCGATATTCTAACCTCTAGTATCAATACCTATTTAAATGACGTTTTAAACAGATTTACGTTTAATATTGAATTAAACCCCTCCGTTTTTATTTGTCTGCTAATCAATATCATGTGATCCACATAATAAAATCCTCCCACAGCAAGTTGTATTTTTAGCTATTTGTTTTCCTTTTTTTATTTTGAAGTATTGCAATATGTATTGTATTGCGATATATCGTGATATATTGTATCGTGACCCATGTATCGTGATATGTATCGTATCGTGAGGCCCTTGCCATATAATACACAGCCCTA

>scaffold_900009-19

TAGGGCTGTGTAAATATATTGATACAGCTAACTATCATGATATTTTGTTTCATGATAGTGTATCGATATTTTAACCTCTAGTATCGATACATATTTAAATTACGCTTTAAACTGATTTACGTTTATGTTAATATTTCATTAAAACCCTCAGTGTTTATTTGTCTGCTGAGTAAACAATATCATGTGATACACATACTAAAATTCCCCTGGTGCTACACAGCAAAATGTATTTTCAGCTATTTGTTTACCTTTTGCAATATGTATTGTATTGCGATATATCGTGATATATTGTATCGTGACCCATGGATCGTGATCTGTATCGTATCGTGAAACCCTTGCCAATACACAGCCCTA

>scaffold_9000015-28

TTAACCCCTTTTGAACGATTGTCGCAAAATCGCCTCAAACCGCTTAAGGCCTGAACGCAGCAGAGTTGGCGTACTGTATGTATCCTGCCAATGCCCTTCTTTTTTTATATAATTGTACATATTTATTTAGGCAACAAGGGGTTAA

>scaffold_1000002-4

TCAGGGCTCGCAAAATCACTAGCCCGTCGCCCCGGGGCTATTGTGTCTTCCAGTCGGGCTACCAAAACGTATCCCCGCCCTGCCTGACGGGATATCTCATAGAGAGGAAATATGTGTCAATGCTTTTGTATTCTCTCACAAATATAGTGGTGTAATTATGTTGTCTGTAATTTTCGGGCATCGGGGCGTCACTATCAGTATGTGAGGTATTGAAATTGAGTTTGAATGTGCGCTCGCTTTTTACTTTCAATCCGCGATCGCACAAACTGTGTATCGGCAGCGGCGGTACTGATTTTACACGATATTTATCGGTGACGCTGTTGCACACCAATTCCTCAGACACGTCCTGTCAGTCATTTAATGTCCTCACGTGACAAGTGAAATCTCACACAGCAAGTTTAAACATGTGATGGAGATACACGCATGCGCAGGGGAGAGAATTGGGAAAATCGCTAACATCTGGAATAGGATGTTCAGTTAGAACCTAATCTTTATATTAATAATTGTAATTTAATGATTTTATATCTACGATTGTTATTTGTGTTTTAAAAATGTTTTGGATTGATATTTTGTCTCCTTTACAATATTCTACATTAAATATTATGTCTTTTTGATATTCAGGCTATTTAAATTCATTTCGGGCTACCAAAAACTGAAGAGTGCCTGCCAGAAAGGCTACCAAGAATTTTTACATTTTGCAAGCCCTGA

>scaffold_1000003-4

ATATACACTTTATGGCCAAATGTTTGGGGACACCTTAATAGCACACCTTTAACTGTGTCTTCCCCAAACTGTTGCCACAAAGTTGGAAGCACCACACAGTTGTCTAGAATGTCTTTATAATCTGTAGCATTGTTTTTTTTCCTTCACTAAAAGACCCAAACCCTGTTCCAGCATGACAATGCCCCTGTGCACAAAGCACTGAGCTCCATGAAGACATGCTGTGTTCTGGTTGGAGTGGAAGAACTCTCATGTCCTACACAGAGCCCTGACTCTGACTCAACCCCACTGAACACCTTTGGGATGAACTGGAACCCTGACTGCACCCCAGACCTCCTCCCCCAACATTAGTGTCTGATCATTTATACTACTGTAAAAGAATTCTAGTGGAAATTCATCAAGTGGTAATCCACCATCTAGTGGAAAGCCTTTCCAGAAGTGTGGAGGTTATTACAGGAGTAAGATGGAGACGGAATCTGGAAATGGATGTTAAAAAAACACATATAGATGTATTTGTCAGGTGTCCCAATACTTTTGTGTATATAGTGTATAT

>scaffold_1000004-1

CTATTCGCACGGGATTAGTATTATCTGGGGACCTCGTGTGATTTAGAAACCCCTCGATGAATTTGGTGTGACGCATCGGCACGGGATAAGTGAAGCCTGTGATTTGACTTGAATTTACTGATTTATCTCCCGGATATGACGTCACAGCATTGGACAGGTTATTGTTAGGAAATATCTTGTGGTTCTATCGGTATAGATGTATGATAGATCATAAACAGAAGAATGTTCATTACCGCATGCCGTTATACAGTAATTTATCTCCATCTAATCATTCTTTTAGTTTTCTCTCTTCTGCTGGTGGTCAGTTTGATCTTTCTGAAAGTTGTTTGGTGTATAACGATGATGCAACACCCAAAATACACCAAACACTCCAACACAGAATTATTCTTCACACACAGGAAACACAAACCTTAACAAATGATACACGACCCCACAAATCCTGACCAAATCACAGAGATGTCCATTCACACGGGATTAATATTATCACATGACCTCGGTGTTCGGAGAAATATAGTAGATCATTTACGGGGGAATTTTTACTTTACAAATGACAGACATGGCCGATTCACACGGGATTAATATCACACACAACCTCCACAATTATTACATTAACCAGAGAATCCCCAGATAATACTAATCCCGTACGAATAG

>scaffold_1000004-2

ATATACAGAGTGGGTGAAAATGAACTAGGCAATATTTAATGGCTGTAGAACTTGTAGTATCTCTGGAGTCATGATGTAAACAGTCTGTAAATAGAAGATAGTAAATCGGATTTTAAGCCTCGCACGCTGGAGGACTTGGAGGTACGGATTCGGGAGGTTCTCTGTAATATCCCCAACCACTTCCTTCAGAACACTGCACATTCCATCTCCGGGTGGTTGAGGAAACTGGTTGACGCCGGCGGGACCTACGTTGAAATTTAAAGATTTGCTTTCATTTTCCTATGTAATAAAGTACATGTACAATTAGTTTCAATCAATTTGTTTTAGAAATATGGACTTTATTACCAATTTTTAATGCCTAGTTACTTTTCACACACCCTATATAT

>scaffold_1000005-4

TAAGCTGAGTTCACACTGCATGATATCAGCCCTGATTTTCACTCGCCGACAGGTTTTACAAAATCCAACAAATGTCCAAAATCGGAGGCAAATCGCTGCTCGTACACACGAATAACAATAACGCAGTGTGAATGATCAAACACGCGATCTATCTGCGATTCAAAATCCTGCTGTGTGAAATGAGTTCTGAGTGAAAAATACCTCTGCGATGACTTACAGCCAATGAGAGAGCAAGATACAGGACATCTGGAAGTTCGGGGAGGAGTTACAGACCATAATATCAGCAAGCATGACTTCGGTTCCGTGAAAGGCTCCTTCTCAGTCTATTTGGATCAGCGCACAGACCATTTGTATCGCAAACTTCTTGCGGGCTTCCATTTTAATAACATTTCTAGTCTCACGCGTGATTTTGTGTTATTTCCTTATTACTTCTCGTGTGCGTTTGGTTGTGAAATGTAGTTTACGGAGCAGACAGAGTTGTTGGCGATTCTTCCTATTGTAAAGTCATGCAGTGTGTAACTTCCTGTCACCGATCCATCGTACAATGGGAACACAACAGCTACTGAACGTTACACCAGATAGTCGTGTAGTGTGAAAACAACGCTGATCCAACAACTTTGAAAATCCTGCAGTGTGAACTCGGCATTA

>scaffold_1000006-1

CTATTCGCATGGGATTAGTATTATCTGGGGACCTCGTGTGATTTAGAAACCCCTCGATGAATTTGGTGTGACGCATCGGCACGGGATAAGTGAAGCCTGTGATTTGACTTGAATTTACTGATTTATCTCCCGGATATGACGTCACAGCATTGGACAGGTTATTGTTAGGAAATATCTTGTGGTTCTATCGGTATAGATGTATGATAGATCATAAACAGAAGAATGTTCATTACCGCATGCCGTTATACAGTAATTTATCTCCATCTAATCATTCTTTTAGTTTTCTCTCTTCTGCTGGTGGTCAGTTTGATCTTTCTGAAAGTTGTTTGGTGTATAACGATGATGCAACACCCAAAATACACCAAAACACTCCAACACAGAATTATTCTTCACACACAGGAAACACAAACCTTAACAAATGATACACGACCCCACAAATCCTGACCAAATCACAGAGATGTCCATTCACACGGGATTAATATTATCACATGACCTCGGTGTTCGGAGAAATATGGTAGATCATTTACGGGGGAATTTTTACTTTACAAATGACAGACATGGCCGATTCACACGGGATTAATATCACACACAACCTCCACAATTATTACATTAACCAGAGAATCCCCAGATAATACTAATCCTGTACGAATAG

>scaffold_1000008-1

TACACTATATGGCCAAAAGTTTGTGGACACCTTACCAGCACACGCATATAAGCGTGTCTTTCCCAAATTCTTACCACAAAGTTGGAAGCACACAGTTGTCTAGAACGTCTCTGTATGTTGTATCATTACATTATTTCTTCACTGGAACTAAGAGACTCGAACCCTGTTCCAGCATGACAATGCCCTTGTGCACAAAACCCCTGAGCTCCATGAAGACATGCTGTGTTCTGGTTGGAGTGGAAGAACTCTCATGTCCTGCACAGAACCCTGACTCTGACTCAACCCCACTGAACCCCTTTGGGATGAACTGGAACCCCGACTGCACCCCAGACCTCCTCCCCCAACATTAGTGTCTGATCTCACTAATACTATTGTAGCTGAAGGATCAAATCCCCACAGTCACATGACCCAATCTAGTGGAAAGTCTTTCTCGTTATTATAGGAAGAAGAATGAGACTACATCTGGAATAGGCTGCTCAAAAAACAAATAAATGTGATGGTAAAGTGTTCCAATACTTTTGGCAACATAGTGTA

>scaffold_180000-1

TATATATATACAGCTCTGGAAAGAATTAAGAGACCACTCCAATTTTTTTTTATAAAATTAGCATCTCTACATGCATGACAGCTATTCCATTCCTGTGTTTTTGAATTCTAATATTGGCACATCAGATTCTCCAGTACTGATTTTGTGATCACCTGAAATATTGTCAGCAGTTTAAAAAATAAAACAAAACTGTCCATTTTACTTAAACACATCGATAAATAGTAAACCCAACGAAATAGATCAGTTTAAGTGGTCTCTTAATTTTTTCCAGAGCTGTATATATATA

>scaffold_200000-22

TAGGGCTGTGTAAAAATATCGATACAGCTTACTATCGCGATACTTTTGTTCACGATAGTGTATCGATATTCTACCCTCTAGTATCAATACATATTTAAATGACGTTTTAAACTGATTTACATTTACATTAATATTTAATTAAAACCCTCCGTGTTTATTTGTCTGCTGAGTAATCAATATCATGTGATACACATACTAGAATTCCCCCAGGTGCTACACAGCAAGTTGTATTTTTAGCTATTTGTTTAAAAAAAATTATATATTTTTGGAAGTATTGCAATATATCGTGATATATTGTATCGTGATATGTATCGTATCGTGAGGCCCTTACCAATACACAGCCCTA

>scaffold_2100005-1

TAGGGATGCACCGATACCATTTTTTGTATGGTACAAGTACAAGTACCGATATTTTTTTCCTGGTACTCGCCGATACCGATACCTGTACTTTTTGGGATGTGGGATTATTTGTATGGCTCTCGGGTTTTCTCTGGCCATTTTCTCTCGTCTTGTAAGGGTTTTCTGCAGGGTTGTTGCCGAGTGCTAACGTTACTGCTAGCGGCTAATTTTGTGTTTTAATTCCAGATGTTTTATCAGATTACTCGTATTATAAGTACTTATTTTTGTACCTCTTGATATTTATGCGGAACAAAGTTTGAGGTCTGCCGTGCGTGGGTTCATTAATTTATTAATTTTGAAATATTTCCAGAGTCTGACATTCTGTCGCTTTTTCAGTGCTTTAATCACTGAAAGTTCTGTCAATGACGTCACAAGAGGTATCGGCCTTTGGTATTGGAGTGTTTTTACATGTACGAGTACATGAGCTCAGTATCAGACCTGATACCGATACCAGTATCAGAATCGGTGCATCCCTA

>scaffold_2100006-8

TAGGGCTGTGTATTGGCAAGGGCTTCACGATACGATACTAATCACGATACATGGGTCACGATGCAATATATCACGATATATATATTGCAATACTAAAAAAAAAAAATACGGAAACAAACAGCTAAAAATACAACTTGCTGTGTGCCACCTGGGGGATTCTAGTATGTGTATCACATGACGTTGATGACTCAGCAGACAAATAAAAACCAAGTGTTTAAATGAAATATTAACGTAAACGTATATCGGTTTAAAATGTAATTTAAATATGTATCGATACTAGAGGTTAGAATATCGATACACCATCGTGGAAAAAAATATTGCGATAGTATCTGTATCGATATTTTTTACACAGCCCTA

>scaffold_21000010-7

CATGATTAGAGATGAGAAATCCAAATCCATTTAATGATTCGAATCTTTCTGAATCACTCATTGAACATCCGAATTTGAGTCACTTGAGTCAGTAGGGATTCTGACTGCTTTTGCCATGGTATTCCACTAGAGGGCACTTAACAAATCCCTAGAATCCACTGAATCCTTAGACTCGAAAATGTCACACACACACTCGGATCCGGTTGTTTCGCTACGTGCTACAAGTCAGACGGATCCACTGAATCCCACGGTTCAGATGAATCTGAGGAATCAGTACCCGCCATTTTGAGTCTCTTATATTTCTGATCATGCAGGAAGTTTCCTAAACAACCCAGTCAGCTACTTGTTCCTACAATTACTAGCTAACAACTACTTGGAACAACTGAATGGTGTCATTTTCAGTTTGTATTTATCAATGTTGTTGTTGGCAACAAGATCATTTTGGACTCATCTGATCCGAGGAAATGAAGAGAAGGATTCGTGAATCCCGAGTCATGTAAAGAATCGGGTTATTTAACCAGGATGAATCCGGATTCGCTCATCTCTAGTCATG

>scaffold_21000010-10

CAGGGCAGGCCCAAGGCTTAAGCAAACTAAGCAGCTGCTTAAGGCCCCCGTGGCCACCAGGGGGCCCCCAAGAGTACATGAAATGACAGATTGTTTTTGTTTTTTGGGATATATTATGTCAACGGACAATGGAAATTATACAAAAATGCAATATTAAATGAAATTAACCCAACGTAATATGATGTTAACGGGATGCAGGATGCACGCACAGCCAGAGTGGGACTGATTAACGTTAACGTTATTGACGTTGATTGAAGAAGCAGCGGCACTGCAGCAAAACTCTCATTAATGTCACAAAAAAGGACACACACATCCTTCCAGTGCAGAGAAAAGAAAAAAGAAAAAAGCGAGGAAGAGAAAAAACAGCAAGATAAACGTAAGTTAAGAAAATGAATGTAAACTAAACGAGGCTAACTTTATATTGTCTGCCGGTTTATTGAGCTACCTTTCAGTACACCACATCAAGTAGGCCATTCGGTACTGCAAGACTTTAAATGTTAAAGTGCAAATGATCACCTGACCGCTCTCGTGGGTTTGCTAATGTTACAATAAAAAATGGTAGAAGGGTAAAAACATTGGTATGATGTAAACAACACAGCTACAACAAATAAAACTGATAATGGGTGTGTTCGATTTATAGTGTGCAAATAATCAAGGTTTGACAATAATTAATCATATTGGTGGCACCGAGGGTCCCCCAAATAAAATTCTGCTTAGGGCCCCCTAAAGGCTTGGGCCGGCCCTG

>scaffold_21000012-2

ATACAAGGGGGGTGTCAAAAAGTGTCGAGACATGCCAGCAGATGGCAGCACAAGGCTGAACACCCAGTTTCTTACATCAGAGCACAGCCTTGTGTGCTGCCATCTGTTGGTGTGTATCAAAACTTTTTGATACCCCCTTGTAT

>scaffold_21000012-4

CTTAGGCTATGTTTACACTGCAAGGTTTAGTGCTCAATTCCGATTTTTGCTCAGATCAGATTTTTTTGCATAGCTGTTCACATTGTTGTTTAAAATGTGGCCAATGTCAGATTTCCAGAGTGAACTGATCATGGTCCTAAACTGACCCACATGCCCAAAATAACCAATTCTACGTCTCGCGCAGCGTCCTGTCATACAGAAAAATAAACATGGAAGACACTGAAAGCAGCGTGTACGCGTTAAATGCTTACATTTAAACGGTGATGTGCAGCGGACGACAGGGAATTTATGAGCAGTTGTTGAGGAGGAAGAGGACGAGGAGAAAAAAAGGACAAATGTTTAATAATAGCATGTTGTGGATCAGTGGCTGCTACTCCAGTATGGAGGTGTGTGTGGATGCGGAGCCGGAGTCAGAAGTGGTGGGACTGTGACGTGATCAACGCCTTCAGCCAAATCGATTATACATCAACTATTTGTAGTTACGTGCTGGTGCAGAATGATGACGCATGTCCATCGAAAATGACGTAGTCGCATGATCTCCGACATAACCGTTCACACTGCGGTCGCATTGCAAAACATCTGATCTGTGTCGCATTTAATAGCATATATGGAAGTGGCACAAATAAGAATTGAAAAGATCAGTGCTGTTCACACTGTCATGAGAAAAACAGATCTGAGTCACATGTGGGCAAAAAAATCGGATTTGGGCCACATTTGCCTGCAGTGTGAACGTAGCCTAAG

>scaffold_21000013-4

ATATGGAGGGGTGAGAACCAGAAGGGGCGTAAGTGATGTTTCACCAACTTTACAGGAGGGGCCAAAAACAAAAAATGACCATAGTTTGATCTGACTTTGTGTACTGATTTCAATCTTTTATAATAAAAATGTGTACTCATATGGATGACAGCATACTTTGTCAGAAGAAAGAGCTCATCAAGAGCTTTCATTTAATATAGATATATCTCCATTTCACCCAAAATGTCACTTACGCCCTTCTGGTTCTCACCCCTCGATAT

>scaffold_21000014-2

TTAGGCTACGTTCACACTGCAGGCAAATGTGACCCAAATCCAATTTTTTTGCACACATGTGACTGAGATCTGGTTTTCCCATGACAGTGTGAGCAGCACAAACCGCTTGGAATCTGATCTTTTCAATTCCTATTTGTGCTACTTCCATATGTGGTATTAAATGCGACACAGATCAGATGTTTTGCAATGCGACTGTAGTGTGAACAGTTACGTCGGAATTCATGTGGAATTCATGCGTCTTTTACGTCATTTGCGATTGACATGCGTCATCATTCTGTACGGCCGCGTAACTACAAACAGTTGATGTATAATTGATTTGGCTGAAGGCGTAGATCACGTCACAGTCCCACCATTCCTGACTCCGGCTCTGCATCCACACACACCTCCATACTGGAGTAGCAGCCACTGATCCACAACATGCTATTATTAAACATTTGGCCCTTTTTCTCCTCGTCCTCTTCCTCCTCAACAACTGCTCATTAATTCGTCCGCTGCACATCACCGTTTAAATGTTAGCAGCTAACGTGTACACGCTGCTTTCAGTGTCGTCCGTGTTTATTTTTCTGTATTTACCGCAGTTTAGGACCGTGTTATCAATTGACACTGGGACGATTTAAAACAACCATGTGAACAGCTATACAATAAAATCTGATCTGAGCAAAAATTGGAATTGAGCACTAAGCCTCGCAATGTGAACGTAGCCTAA

>scaffold_21000014-3

CTTATGCCGAGTTCACACTGCAAAATTCTAGCCTCGATTTTCACTCGCCGACACGTTTTGCGAAATCGCCAACAAATGCCTGAAATCGGAGGCAAATCGCTGCTCGTTCACGCGAGTAACAATCACGCAGTGTGAATGATCAAAGACGCAATCTGAGAGAATCGCAGAGGCGTCGCGGGGGCAATTGAAATATTTGGCATGCTAAATATCTGGATCTGTCTGCGATTCAAAATCCTGTAGTGTGAAATGAGTTCTGACTGAAAAATACATCAGCGATGACCTCCAGCCAATGAGAGAGCAAGGAACAGCGGGAAGTTCGGGGAGGACTTATAGACCACAATATCAGCAGGCATGGCTTCGGTTCCCATCTTTTAAAATCTTTTTCGCTGCAAATCAGTGCACAGACCATTTGTATGGCAAACTTCTTGCGGGCTACCATTTTAATAACATTTCCAGTCTCGCGCGAGAACTCTGATCTCACGCCTGATCTTGCGTTATTTCCTTTATCACTTCTCGTGTGAGTTTGGTTGTGAAACCTAGTTTGCGGATCAGACAGAATTGTCGTTGATTCTTCCTATTGTAAAGTCATACAGTGTGTATCCTCGTGTCGCCATCGTGCATGTGAACACAGCAGCAACTGAATGTTTACCCAGATAATCGTGCAGCGTGAAAACAACGGCGATCCGACCACTTTGGAAATCGTGCAGTGTGAACTCGGCATAAG

>scaffold_21000014-4

CTTATGCCGAGTTCACACTGCACGATTTCCAAAGTGGTCGGATCGCCGTTGTTTTCACGCTGCACGATTATCTGGGTAAACATTCAGTTGCTGCTGTGTTCACATGCACGATGGCGACACGAGGATACACACTGTATGACTTTACAATAGGAAGAATCAACGACAATTCTGTCTGATCCGCAAACTACGTTTCACAACCAAACTCACACGAGAAGTGATAAAGGAAATAACGCAAGATCAGGCGTGAGATCAGAGTTCTCGCGCGAGACTGGAAATGTTATTAAAATGGTAGCCCGAAAGAAGTTTGCCATACAAATGGTCTGTGCACTGATTTGCAGCGAAAAAGATTTTAAAAGATGGGAACCGAAGCCATGCCTGCTGATATTGTGGTCTATAAGTCCTCCCCGAACTTCCCGCTGTTCCTTGCTCTCTCATTGGCTGGAGGTCATCGCTGATGTATTTTTCAGTCAGAACTCATTTCACACTACAGGATTTTGAATCGCAGACAGATCCAGATATTTAGCATGCCAAATATTTCAATTGCCCCCGCGACGCCTCTGCGATTCTCTCAGATTGCGTCTTTGATCATTCACACTGCGTGATTGTTACTCGCGTGAACGAGCAGCGATTTGCCTCCGATTTCAGGCATTTGTTGGCGATTTCGCAAAACGTGTCGGCGAGTGAAAATCGAGGCTAGAATTTTGCAGTGTGAACTCGGCATAAG

>scaffold_21000015-2

CTTAAGTCGGGTTTACACTGTGCGATTTTGGCCACAATTTGGTTGTCAGAGACAAATTTTAAAATCCTAAAAGATTCCTATCATCGTCCGCTAAAATCTGTTGTCTTTGATGGCTAGTTTGACGTGTTCACCGACAGATGATTAACGGCCGTTTCGATCAGTTTTTTCCTCCGATAAAATTCTGGCAGTGTCCGAAGATTTCAGCCGCTTTCCTACAGTGTGACGTGTCCTACGACGACCGTCAAACCAAGAACCAATAGGAGCACCAAACCTGATGACGCAATTAACGCTACAACTTCAAACCGTTTCAGGAAAATTTGGAAACAAGTTCGGTGGACATGAAACTTATTGAGTTTTGGCGGGAAAAGGAGTCTTTGTATGACGTGTCATCGCTGCTGTTTGTTTGCTGCTAGCTACCATTTAGCGAGAGTTACTGAACCTGTCACGGATTGAAGACGTAAAACTCCATACGAGTCTTCTTGTGTGCGTCCGTTTTTTGACGAGTTTTGACTGTTGTACAGTCTGACATTATGACAACTGACATCCTACAATGTAACATGGGGATCATGTTCTTACAGTCTGACAAGCAACAGTGACAAAACACTATTAAAAATCGTACAGTGTAAACCCAGCTTAAG

>scaffold_21000015-3

AATTAGGGCTGTGTATTGGCAAGGGCCTCACGATACGATACATATCACGATACATGGATCACGATACAATAGATCACGATATATATTGCAATACATTGTAATACTGCAGAACATTGCTTTTTTTTATATAAAACAAATAGCTAAAAATACAACTTGCTTTGTACCACCTGGGGGATTCTAGTATGTGTATCACAATATATATATATATATATTACTCAGCAGACAAATAAAAACTGAGGGTTTGAATGAAATATTAACATAATTTAAATATGTATCGATACTGGAGGTTAGAATATCGATACACTATCGTGAAAAAAAATTTCGCGATAGTTAGCAGTATCGGAATATTTTTACACAGCCCTAATT

>scaffold_21000016-21

TAGGGCTGTGTATTGGCAAGGGCCTCACGATACGATACATGTCACAATACATGGGTCACAATACAATATATCACGATATATTGCAATACAATACATGTTGCAATACTTTAAAAAAAAATCTAACAAATAGCTAAAAATACAACTTGCTCTGTAGCACCTGGGGGCTTCTAGTATGTGTATCACATTATATTGATAACTCAGCAGACAAATAAAAACTGAGGGTTTTAATGAAACATTTATGTAAACGTAAATCAGTTTAAAACAATTTAAATATGTATCGATACTAGAGGTTAGAATATCGATACACTGTCGTGAAAAAAATATTGCGATAGTTAGCTGTGTCGATATATTTACACAGCCCTA

>scaffold_21000019-3

TTTAAAGGGGTCATGAACTGAGAAATTAAAATTCCTTTGGTCTTTTGACAAATAAAAGGCCATAATTGCTATAAAAAACATCCTGTAAGTTTCAGAACTCATAACTTTGTCATTAGTATAAAAACAGCTTATATTGAAAGCAGGCGGTAAATACAACATGTTTTGGAATGTTCTACTTGATGACGTGAATAAGCGCCACCTCCGCAGAAGATCGACACCTGCTTTGACATCACTGTCCGTTTAGCCCCGCCCACCGATTTGCGCATGCATGTGATAAGTATATATATTTTTATTTTACTTCTGAGACAACAATCCGCCACGCCTATTCAAACAGAGCCTTCTGGTGGGGGTGGGGAGGGGCAAAAACAGGACAGAAAATAGCCTATTACTTGTAAATGATGATGTTTTTAGATGTAAAAATCTTGATAACTTTATAAGTGGACCTCAGAGAACAGTACAAAATAAAATACTGAGGTAATTCATGAGCCCTTTAAA

>scaffold_21000020-1

TACACTATATTGCCAAACGTTTGTGGACACCTGACCATCAGATCCATATGTGGTTCCTCTCCAAAATGCTGCTACAAATTTGGAAGCACACAGAATGTGTCTAGAACGTCTTTGTATGTTGTAACGTTACATTATTCCTTCACTGGAACTAAAAGACTCAAACACTGTTCCAGCATGACAATGCCCCTGTGCACAAAACCCCTGAGTTCCATTAAGACATGGTGTGTTATGATTAGAGTGGGAAAACTTGTGTTCTGCACAAAGCCCTAACCTTAACACCACTGAACACTTTTGAGATGAACTGGAACTCCGACTGCACCCCAGACCTCCTCACCTGACGTTGGTGACGATCTTACTAATACTATTGTTGCTGAATGAACACAAATCTCACATTCAAGCTCCAAATTGTAGTGGAAAGCCTTACTAGAAGAGTGGAGCTTAATCTAACAGTCTAAACTCAAGTTTGTGATAGTCAGGTGTCCACAAACTTTTGGCAATAAAGTGTA

>scaffold_21000020-10

ACTATATTGCTGAAATATTTGTGGACACCTCACCATCAGATCCATAGAGTATGTGGTACCTCTCAAAGTTTGCTACAAATTTGGAAGCAGACAGTCGTCTACAACGTCTTTGTATGTTGTAGCGTTACATTATTCCTTCACTGGAACTAAGAGACCCAAACACTGTTCCAGCATGACAATGCCCCTGTGCACAAAGCTCCTGAGTTCTATTAAGACATGGTGTGTTATGATTGGAGTGGGAAAACCGGAGTGTTCTGCACAAAGCTCTGATCTTAACCCCACTGAACACCTTTGGGATGAACGGGAACGCCGACTGCACCACAGACCTCCTTACCTGACGTTAGTGTCTGATCTCACTAAAACTATTATAGCTGAATGAACACACATCTCACATTCAAGCTCCAAATTGTAGTGGAAAGTCTTACTAGAAGAGTGGAGTTTAATCAAACAGCAAAATTGGGAGAAAATCTGGAATAAGAGGTTTAAAATTACATACGATTGAGATGGTCAGGTGTCCAAACTGTTGCAATATAGT

>scaffold_21000021-2

GTAATCCATCTTCCTTAAATTACTTTGTTCGGCTTGTTTTCCAGCCAACCCTGCCCTACCCACTGCTGGTTACCTGGTTCAGGTGTGTTTAGTCAATCAGAAGTTGGAAGATACCATTTCAGATGAGGGTGGAGTGGGGGAAGACCTGGTGATTTGGTATTTCCTACCCTATGATTGACTGAACCAGGTAACCAGCAGTGGGTAGGGCAAGGGTAGCTGGAAAACAAGCGGAACAAAGTAGCTTAAGGAATATGGGTTAC

>scaffold_21000021-12

TCTACAGAGTGGGTGAAAAGTAACTAGGCATGAAAAATTGGTAATAAAGTCCATATTTACAATACAAATTTATTGAAACAAATTGTACATGTACTTTATTACATAGGAAAATAAAAGCAATGTTTAAAATTTCAACGTAGGCTTCTACCAGTTTCCTCAAACGGCCAGAGATGGAATGCATAGTCTTTTGAAGGAAGTGGTTTGGGATATTGCTGAGAACCTCCTGAAGCCGTACCTCTGAGTCCTCCAGTGTGCGAGGCTTGAAATCCAATTTACTATCGTCTATTTAGAAACTGTTTTCATCAGGACGCCAGAGATACTACAAGTTCTATAGCCATTAAATAATGCCTAGATAATTTTCACCCACCCTATAGA

>scaffold_21000021-23

TAGGGCTGTGTAAAAATATTGATACAGCTTACTATCGCGATTTTTTTTTCATGATATATTAACCTCTGGTATCAATGTCTATTTAAATGACGTTTTAAACTGGTTTACGTTAATATTGAATTAAAACCCTCAGTTTTTCTTTGTCTGCTGAGTAATCAATATAAGGTGATACACATACTAGAATACTACATACAGGTGGTACACAGCAAGTATTGCAATATGTATTGTATTGCAATATATCGTGATATATTGTATCGTGACCCATGTATCGTGATATGTACCGTATCGTGAGGCTCTTGCCAATACACAGCCCTA

>scaffold_21000022-2

ATACAGCTCTAGAAAAAATTAAGAGACCACTTAAACTGATCTATTTCATTGGTTTTACTATTTATAGGTGTGTTTAAGTAAAATGGACAGTTTTGTTTTATTTTTAAAACTGCTGACAATATTTCAGGTGATCACAGAATCAGTACTGGAGAATCTGATGTGCCAATATTAGAATTCAAAAACACAGGAATGGAATAGCTGTCATACATGTAGAGATGCTGATTTTATTTTTTTTTAAATTGGAATGGTCTCTTAATTTTTTCCAGCGCTGTAT

>scaffold_21000023-4

ATGCCGAGTTCACACTGCACGATTTTCAAAGTCGTCGGATCACCGTTGTTTTCACACTGCACGACTATCTGGGGTAACATTTAGTTGCTGCTGTGTTTACATTGCACGATGGATCGGTGACAGGAGGTTACACACTGCATGACTTTACAATAGGAAGAATCACCGTCAACTCTGTCTTTTCTGCAAAATACGTTTCACAACCAAACGCACGCGAGAAGCAATAAGGAAATAACGCAAGATCCTGTGTGATATCAGAGTTCTTGCGCAACAAGTTTGCGATACAAATGGTCTGTGCGCTGATTTGCAGCGAAAGGGCGAAAAAGAAAAAAAAGATTATGGAGGAGGAAATAGTTGGGGTGAACCGAAACCATGTTTGTTGATATTGTGGTCTATAACTCCTCCCTGAGCTTCCCGCTGGCCTGTATCTTGCTCTCTCATTGGCTGTAGGACATCGCAGATGTATTTTTTTAGTCAGAACTCATTTCACACATCAGGATTTTGAATGTCCGACAGGTCCAGATATCGGCTACGCCTCTGCGATTGTCTCAGATCGCGTCTTTGATAATTCACACTGTGTGATTGAGCACCGATTTGTCTCAGATTTCAAGGCGATTTTGTAAAACCTGTCGGCGAGTGTAAATCAGGGCTAAAAACGTGCAGTGTGAACTCGGCAT

>scaffold_21000023-8

TTAAAGGGCTCATGAACTAACTCAGTATTTTATTTTGTACTGTTCCCTGAGGTCCACTTATAAGGTTATCAAGATTTTTACATAAAAAAAACATCATAATTTAGAAGTCATAGGCTATTTTCTGCCCTGTTTTTGACAACCCCCCCCAGAACGCTTTGTTTGATTAGGCATGGCGGATTGTTGTCTCAGAAGTAAACGCCCACTGCTATGATTGGCTAACAGTTTTGCATTTTACCCATCATGCGCAAATCGGTGGGCGGGGCTAAACAGACAGTGACGTTGAAGCAGGCATCGATCTTCTTCTTCTGCGGAGGCGGCGCTTATCCACACTATTACATCATCGAGTAGAACATTCCAAAACCTGTCGTTTTGGCCGCGCCTGCTTTCAATATAAGCTGTTTTTCGAGTAACGACAAAGTTTTGAGTTCTGAGACTTACAGGATGTTTTTATAGCACAACGACCTCTTATAGGTCAAAAGATCGAGGGAATTTTGATTTCTCAGTTCATGACCCCTTTAA

>scaffold_21000023-10

TTTAAAGGTAGGGTGGGTGATTTTAGAGAGGCTAGCAATAGCAAGCTAGCATTGAAAACCAAAGATCCTGCCCTTCCTGCATAATCACGCTGCAAAGCCACGCCTCCTCCAAAACACATGAACGCGCGGGCACAGGCAGACCGGATCTACACCTATATTAACTCTACTTTTATCTCTTTGCTTAACACTTTTTTAGACATCGCTCTTTCTAACACTGTTTTTCTCATGCGATGGCTTGTGCCACTTTTAGAGGCAATGTTGGGCTCTTGCTGTTTGCCTTCCATTTTCTCTCAGTCTGCATAGCAGGAATGCGCTAATGACGTGTTCTGTCTGCGCGAACAGGGGTGCACAGAGTTATGCAAATACATATTTGACAGGCAGGTAGGACAGCCTATCGTAATGTTCGGATTGAACATTTTGATTGGACGAAGTTTTTTTGGTCTTACGGCTTCCACCGAATATATAAATACATTTAGACCACTGAACTTTTAATGAGTGCTATTGAGATGTGAAGAGACTTTCAACCAGCATAACAAAACATGTTTTGAACCAAATCACCTAGCCTGCCTTTAAA

>scaffold_21000023-13

TATATCGCCAAAGGTTTGTGGACACCTCCAAATGTGGTTCCTTTCCAAAATGTTGCTACAAATTTGGAAGCACACAGTCGTCTAGAACGTCTTTGTATGTTGTAGCATTACATTATTTCTTCACTGGAACTAAGAGACCCAAACACTGTTCCAGCATGACAATGCCCCTGTGCGCAAAACCTTTGAGTTCCATTAAGAGATGGTGTGTTCTGATTGGAGTGGGAAAACTTGAGTGTTCTGCACAAAGCCCTGACCCTTAACACCAGTAAACATCTTTGGGATGAACTGGAACCCCGACTGCACCCTAGACCTCCTTACCTGACGTTAGTGTCTGATCTCACTAATACTATTGTAGCAGAATGAACACATATCTCATATTCAAGCTCCAAATTGTAGTGGAAAGTCTTACTTGATGAGTGGAACTTAATCTAACAGCAAAATGGGGAGTAAATCTGAAATAAGAGGTTTAAAATCACATGTGTTTGGGATGGTCAGGTGTCCTTAAACTTTTGGTAATATA

>scaffold_21000024-5

TGGCTCTGTCCGAAATCGCATACTCCTTAAGTACGTACTACATTTGAGTTACTACATACTTCCCAACCGTTAAAAAAGTATGTTCTATATAGTATGATTCTGCGAGTATGATCAAAATTCTGACGTACTACATCTGCCATGTTGATATTGTCACATGACATACGTCGTCACAGCAGCGTGAATTTAACGTTGGATAATGTTTACCTGGCTAACGTTAACCACGAGGAACAATGTTTTTTTTCTTCCCCTGAAGTACTTAACGCTACTTTAAGCACTTGAAAAGTGCTTAAAATTGCCGCCATTTTCGAATATGACAAGTCGTCTCTTCCGTGGACACATGGGATAGTAAAGTGTCCATCGTATAGGTACTTCAAAATCCAGGCGAAAATAGTAGGTTCATCCGAGAACTTTTCACCTGCTGGTTTTCGCATGCTAAGGATTCGGAAATACAACTCGCCTCGCAAATTCTATACAGTAATATAGTATGAAAGTATGCGATTTCGGACGGAGCCA

>scaffold_21000024-16

ACTAGAGATGAGTGAATCTGGATTCATCCGGGTTAAATAACCCGATCCTTTATATGACTCGGGATTCACGAATCCTTCTGTTCATTTACTCGGATCAGTTGAGTCCTAAGTTATCTTGTTGCTAACAATAACATAGATAAATACAAACTGAAAATTACACCTACATCTAAGTTGTTCTAAGTAGTTGTTAGCTAGTAACTGTAGGAACAAGTAGCTGATGGAGTTGTTTAGGAAACTTTCTGCATGCTCAGAAACGCAAGACACTCAAAATCGTGGGTACTGATTCATCTGAACCGTGTGAACCGTGTGATTCAGCGTATCCGGCGTAGCGCGTAGCGAAACAGACAGATCCAAGGGACTCACTGTGTCAATTTCATGAGTCCATGTATTCGGTGGATTCTAAAGATTCATTGAGTGCCCTCTACTGGAAGGACCATGGCAAAAGCAGTCAGAATCCCTACTGACTCAAGTGACTCAAAGGACTCGTGAGAGACTTAGATCCATTCAATGAGTGATTCAGAAGGATTCGAATCCGTAACTGGATTGGGATTTCTCATCTCTAGT

>scaffold_21000028-1

TTACACTGTGCGATTTTTAATAGTCCTTTGCGATTGTTACTTGTCAGACTGTACAAACATAATGTCACACTGTAGGGTCTCAGTTATCATAAAGCCAGACTGTACGACAGTCAAAACGACTCAAAAACGGACGCACACAAGAAAACTCGTCCGGTGTTTTACATCATTAATCCGTAACAGGTTCAGTAACCCGCGTTCTCTCGCTAAATGGTAGCTAGCAGCAAACAAAAAAAAATTGTGGCGTTAATTTTGCTCATGACATGTCGCAATAAGTCCCTCCTCTTGCTGTTCTGTCCACCGGACTCGTTTCGAAGGTGAATTGCGTCATCAGGTATGGCGCTCCTATTGGCTCTTGGTTTGATGGTCATCGTAGGAGACGTTACACTGCAGGAAAGAGTCTGAAATACTGATCGCAACGGCCATTAATTGGCCGTCGGTGAACATGTCAAACTAGCCATCAAAGACAACAGATTTTAGCGTAAGATTATAGGAATCTTTTAGTATTTCAAAATTCGTCTTAGATGACCAAATCGTGTCCAAAATCACACAGTGTAA

>scaffold_21000028-13

TAGGGGTGGGAATCACAGGTTAACTCACGATACGATACTGTCACGATATTTTGCCCACGATAACGATATATCACAACACCAGCGATTCTGTGACAGACGATATATTGCAAGAAAATCATTTACGATACATCACGATATCTCTAAATAAATTTAAAAATGCATCAAAAAGTCATATGATTTATTTCATTTATTAAGAGTACATCTATTTTATAAAAATCGTATAAAATTCTATAAAATCTATTTAATCACTGGGGGTACTGCCGCTCCGGTAAGCCAAAGTGGTAAACAGTTACGGTGTAGGACAGGTATGAATTTAGAGCTTTTGTATGTTTAAAAAATATCGATACTTGGTGCAAGAGTATCAATAACGTCTCACGGCCAGAAATATCGCGATAAATCGGCAAATCGATTTTTTGTCCCACCCCTA

>scaffold_21000029-1

TACAGGGTGGGTAAAAATTAACTAGGCAATATTTAATGGCTATAGTTGTACTTGTAGGATCACTGGAGTCATGATGAAAACAGTCTCTAAATAGACGGTAGTAAATAGGATTTCAAGCCTCGCACACTGGAAGACTTGGCGGCACGGATTCGGGAGGTTCTCAGCAATATCCCAAACGGTGCCCATGTTGAAATTTGAAAGATTTGCTTTCATTTTCCTATGTAATAACGTACATGTACAATTTGTTTCAATAAATGTTTATTAGAAATATGGACTTTATTACTAATTTTTAATGCCTATTTACTTTTCACCCACCCTGTA

>scaffold_21000029-5

ACTAGGGCTGCACAATATATCGAAATCATCGCAAATGTGCATATCGAGATATGCATATTGCAATGGTTTGCGATAAATGAGCGATTTAATACTTCAAATAGTAATGTGTGGTCAAAGTTTTAGGATGGTAAAATATCTGGCATGCAGATGTTAGTTGGGATGTGCGTGCACGTGCTTTGACGCAAGATGGTGAACAAGCTGCGAAGCTTTCATAGCGAGCTAATCCTGATCAGAAGAGCCAGCCCGAAAAACACCCGCAGCTAAAATAGCTAATAAACATAACATTTCTCTGGGGAAATATTATATCGCAAGAAATATTGTTATCGCAATACTCGACAACAATATCGCATATTTTCCCTGTATCGTGCAGCCCTAGT

>scaffold_21000029-6

TAGAGATGCACCGATACCATTTTTTAAAGACCGAGTACGAGTGCCGATATATTTTTTTCTTGCCGATACCGATACCGATACTTTTTGGGGATGTGGGATTATTTGTATAGCTCTTGAGTTTTCTCTTGCCCTTTTCTCTCGTCTTCCAAGAGTTTGCTGCAGGGTTGGTTCCCGAGTGCTAATGTTACTGCTTGCGGCAAATTATCCATGCTCACTTTTGTGTTTTAATTTCAGGTGTTTTATTAGATTACTCGTATTATAAGTACTTATTTTTGTACCTCTTTATATTTTTGCGGAACAAAGTTTGCAGTCTGCCATGCGTGGGTTTTTGAAATATTTCCTCACTACTAAGCCTGACATTCTGTCGCTGCTGCTAGAGTCTTTGTGCACTGAAAGTTCTGCCAATGACCTCACAAGAAGTATCGTTTTTTGGCATCGGAGTGTTTTTACGAGTATGAGTACATGAGCTCTGTATTGGGCCCAATACCAGTATCGGTGCATCCCTA

>scaffold_21000029-8

TAGGGCTGCACAATATATAGAAATGATTGAAATATGGCAAATGTGCATAGTGCAATATGCATGTCGCAAGGGTTTGCGAGATATGAGCGATTTAATACTTCAAATAGTAATGTATGTTTAGTGTATATCAAAGTTTTAGGTCGATGCAGATAGTAAAATATCTGGCACGTGGATGATAGTTGGGATATGCGTGCGCGTGCTTTGCTGCACGACAATCAACAAGCCGCAAAGCTGTCATAGTGAGCTAATCTTGATAAGATGAGCCAGCCTGAGGCACCCACAGATGCTGCCGACGATTTAGTGCCAAAACGATGAAGCACCTTAAAGATTTGACAGTATTTCGGTTTTAAAAGAGATGACCACATGCAGACTATAGTTCTATGTAGAACATGTAAATCTCGAGTGGCAACGCAAAGTGGAAACACTACAAATTTTTACATTCACCTCAAAACGAAGCACAACAATTTGTATCAGCTATGCTCGGCCGAAAAATCTTCAAACACAGGTGAATGTGTTTGAAGAAACATATGTTTCTTTTTTTTATATCGTATCGCATATAGAATATTGCATTATTATCATATCGCATTTTTCTTCAATATTGCGCAGCCCTA

>scaffold_21000030-5

TTACTAAAGCCGGGCTCACACTGTGCGTTTCTGACCACGATTTGGTCATTTGAGACAAATTTTGAAATGCTAAAAGATTCCTATAATCCTACACTAAAATCGGTTGTCTTTGATGGCTAGTTTGACATGTTCACCGACAGACCGATTAATGGTTGTTGCGATCAGTTTTTTTCCTCCGAAGAAATTCTGGCAGTGTGAGAAGATTTCAGACACTTTCCTGCAGTGTGACGTCTCCTACAACAACCGTCAAACCAAGAACCGATAGGAGCGCCGAACCTGATGACGCAATTAGCGCGACAATTTCAAACCACCTCGGGAAAATGTGGAAACGAGTCCAGTGGACAGACCAGCAAGAAGAGAAACTTATTGAGTTATTTAGGGAAAAAGGAGTGTTTATATGACGTGTCATCGCTGCTGTTTGTTTGCTGCTAGCTACCGTTTAGCGAAAGAACGCGGGTTACTGAACGTGTCACGGATCGATGACGTACAACTCCGGACGAGTCTTTTTGTGTGCGCCTGTTTTTGACGCGTCTTCACTGTCGCACAGTCTGACATTATGACAACTGAGATCCTGCAGTGTGACACGGGGCATCATGTTCATACAGTCTGATAAGCAACAATAGCTAAAGACTATTAAAAATCGCACAGTGTAAACCCGGCTTAAGTAA

>scaffold_21000031-5

GTTTACACTGTGCGATTTTTAATAACCCTTTGCGATTGTTGCTTGTCAGACTGTACGAACATGATCCCCATGTACCAGTGTAGGATCTCAGTTGTCAATAATTTCAGACTGTACGACAGTCAAGACGCATCAAAAACAGACGCACATAAGAAGACTCGTCCGGAGTTTTACATCATCAAGCCGTACAACGTTCAGTAACCTGCGTTCTCTTGCTAAACGGTAGCTAGCAGCAAACTAAAACAATCTGTAGCGTTAAATTTGCTCATGACGTGCCGCAATAAGTTTCTCCTCTTGCTGTTCTGTCCACATGACTCGTTTTGACATTTTCCTAGTACGAGAAGTCACACTGCAGGAAAGTGTCTGAAATCTTCTCACACTGCCAGAATTTCATCGGAGGAAAAAACTGATCGCAACGGTCATCAATCGTCTGTCGGCGAACATGTCAAACTAGCCATCAAAGACAACAGATTTTAGCGTCGGATTATAGGAATCTTTGAGGATTTCAAAATTTGTCTCAGACGACCAAATCGTGGCCAAAAATCTCACAGTGTAAAC

>scaffold_21000032-3

GGCTCTGTCAGAAATCACATACTTCAATACTATATAGTATGCGAAACTGAGTAAGCAAGGCGAGTAGTATGTCCGAATCGTTAGTATGCGAAATACAGTAGGTAAAAAGTTCCTGGATGATCTCCTACTTCTACCCGAATTCTTGAAGTACGCATATGATGGACCCTTTGTTAACCCATGAGCCCACGCGAGAGACGACTCATCATATTCGAAAATAGCGGAAAGTGGCGTCGCCGCATTTCTCCAAGTGATTTAAGTAGCTTTAAGTACTTCAGAAAAAAAAAAACATTGTTTCTTGTGGTTAAATTGCACTTGTTTAACATAATCTAAGTAAACAATGTCCAACTTTTAATTCTCGCTGTTGTGATGACGTTTGTTACGTGACAAATATCAACATGGCGGATGTAGTACGTCCGAATTTCATTCATACCTCCCATAATCATACAATATAAAACATACTTTTTTTAACAGTCGGTAAGTACGTACTAACTCAAATGTAGTACAGTAGTATGCAATTTCGGACAGAGCC

>scaffold_21000033-2

TACACTATATTGCCAAAAGTTTGTGGACACTTGACCATGACAAACAGATGTGATTTTAAACATCTTTATTCCAGATTTATCCCCTCTTTGTTGTTAGATTAAGCTCCACTCTTCTAGTAAGACTTTCCACTACAATTTGGAGCTTGAATGTGAGATGTGTGTTCATTCAGCTACAATAGTATTAGTGAGATCAGACACTAACGTCAGGTAAGGAGGTCTTTGGTGCAGTCGGCGTTCCAGTTCATCCCAAAGGTGTTCAGTGGTGTTGAAGTCAGAGCTTTGTTTTTCCACTCCAATCATAACACACCATGTCTTCATGGAACTCAAGGGTTTTGTGCACAGGGGCATTGTCATGCTGGAACAGTGTTTGGGTCTCTTAATTCCAGTGAAGGAAAAATGTAATGCTACAACATATAAAAGACTTCCAAATTTGTAGCAACAGTTTGGAGAGGAATCACATATGGATCTGATGGTCATGCGTCCACAAACTTTTGGCAATATAGTGTA

>scaffold_21000033-3

CTAATGTACTATTCACACAGGATTAGTATTATCTGGGAACCTGGTGTGATTTAGAAATTCTCCCCACATCTAAATTTTGTGTGGCGCATTTGTACCGGATAATTGAAGCCCGTGATTTTACTTGAATTTACTGACTTGTCTCCAGGATATGATGTCAAAGCATTGCTTAGAAATATCTTGTGGTTATGTATGGATGTATGATATATCATAAAGAGGAGAATGTTCTTTACCGCGTGCCGGTCTATACGTGTAATTTATCTCCATCTAATCATTCTTTTTGTTTTCACTCTTCTGGTGGCGTTCAGTTTGATCGTTCTGAACGTTGTAGGGTTGTTTGGTGTATAGCGATTATGCAGCACCCAAAACACTCCAACACAGCCTCATTCTTCACACACAGGAAATACAAACCTTAAAAAATTATACATGACAACACAAATCCTGGCCAAATCACACAGATGTCGATACACAGGAATAATATTATTACATGACCTCTGTATTCGGCGAAATATGGTAGATCACTTGAGATGGAATTTTTACTTTACAAATGAAAGACATGGACGATTTACACGGGATTAAGATCACAGACCACCTCCACAATTATTACTAATAACCAGACGTCCCCAGATAATACTAATCCTGTGCGAATAGGACTTTAG

>scaffold_21000035-2

ACTATATTGCCAAAAGTTTGTGGACACCTGACTATCACAAATATGTGATTTTAAACATCTTTATTCCAGATTTATCCCCTCTTTGCTGTTAGATTAAGCTCCACTCATCTCTAAGCGTTCCACTACAATTTGGAGCTTGAATGTGAGATGTGTGTTCATTCAGCTACAATAGTATTAGTGAGATCAGACACTAACGTCAGGTAAGGAGGTCTGTGGTGCAGTCGGTGTTCCAGTTCATCCCAAAGGTGCTCAGTGGTGTTAAGATCAGAGCTTTGTGCAGAACACTCAAGCTTTTCCCACTCTAATCATAACACAGCATGTCTTCATGGAGCTCAGGGGTTTTGTGCGCAGGGGCATTGTCATGCTGGAACAGTGTTTGGGTCTCTTGGTTCCGGTCAAGGAATAATGTAATGCTACCACATACAAAGACATCCTAGACACCTGTGTTTCCAAATTTGTAGCAACATTTTGGAGAGGATCCACGTATGGATGTGTGGGTGAGGTGTCCACGAACTTTTGGCAATATAGT

>scaffold_21000036-4

TTAGGGCTGCACGACACTGGGAAATTATGCGATATGCGCGATTGTTGTGGAGTATTGCGATAACGATATTTTTTGCGATATAACGTTTCCCTAGAGAAATGCTATTTTTATTAGCTACTTTCCCTGCGGGTGTTTTTCGGGCTGGCTCATCTTATAAAGATTAGCTCGCTATGAAAGCTTCGCAGTTTGTTGATTGTCTTGCGTCAAAGCACGCGCACGCAAATCCCAACTAACAGCCGCGTGCCAGATATTTTACTATCCTAAAACTTTGACCACTTATTACTTATGATGTATTAAATCACTCATTTATCGCAAACCATTGCGATATTTCTATCATTTCGATATATTGTGCAGCCCTAA

>scaffold_21000036-5

TAGGGCTGTGTATTGGCAAGGGCCTCACGATACGATACATATCACGATACATGGGTCACGATACAGTATATCATGATATATTACAATACAGTACATATTGCAATACTTTAAAAAAAAAACAAAAAAAAAAAAGGAAAACAAATAGCTACAAATACAACTTGTTGTGTCCCACCTGGGGGATTTTAGTATGTGTATCACATGATACTGATGACAAATACAAACTGAGGGTTTTAATTAAATATTAACCTAAACGTAAATCAGTTTAAAAGGTCATTTAAATATGTATCGATGCTAGAGGTTAGAATATCGATACACTATCGTGAAAAAAAATTATCGCGATAGTTAGCTGTATCGACATTTTTACACAGCCCTA

>scaffold_21000038-4

AAGCTACAGTCCGTAAAGTTTGCCTCTTTGTCGCCATCTCTGTTTGAAACCTGCAATTGCATTTATTTTCGGAATTCTCTTCTTTACGTGGGTTGTGCATCGGTACGGCTCCTCGGCGCGAATGAATCTAATGTTTTGCGGTGTGTGTGGGCTGTCAGTCATCGCACCGGTGTGAATACTACACTTTGGAATGACAGATTCTACGTCTTGAAAGTGTGATTCAAATAAAAAATTTTACCGGAGAATGTCATCTGAACAAGTAAGTAACATATCTGCCACTTTTGTTCTGACCAACTGAGGAAAAAAAGCATTACAATAAATCACGCTACCAAAGGGGATTAAATCTAACGATTGCCAATTAGCTCATATCACATCAAACTTTGCAAGTTATTATTATTGTTATACTTTGTTCTCAAATTGATCTTAACATCAGCAATGCGTGACTACGTGTTTAGTGTGTACTGGCATTAGCATTCCCGCATGCGATATAGCCGGGACTACTTCTTTATGTTTACGGACGTGACGTAATGACGCAAAGACGAACGGTGCCATGCTCTTATTTCCTGTGAAAACCTACCAGTACCACTCAAATTATAAAACATTATTACAAGATTGGCGTTGTGAATCAGGCGGCGTTAAGGAAATAGTTTTAAACACTGGCTGGGAACGTACTTGCTTAATAATTCATTTCGAATCATTTTTAACCAAAAAATGTTACGGACTGCAGCTT

>scaffold_21000039-36

TAGGGCTGCACGATACTGGGAAAATATGCGTTATGCGCTATTGTTGTTGAGTATTGCGATAACGATATTTCTTGCGATATAACGTTTCGCTAGAGAAATGCTATTTTTATTAGCTATTTTAGCTGCGGGTGTTTTTCGGGCTGGCTCATTTTATCAAGCTTAGCTCGCAATTAAAGCTTCGCAGTTTGTTGATTGTCTTGCGTCAAAGCACGCGCACGCAAATCCCAACTAACAGCCGCGTGCCAGATATTTTACTATCCTAAAACTTTGACCACACATTACTATCTGAAGTATTAAATCGCTCATTTATCGCAAACCATTGCGATATGCATATCTCCATATGCACATTTGCGATATTTCTATAATTTCGATATAATGTGCAGCCCTA

>scaffold_21000040-10

TTAGGGCTGCACAATATATCGAAATTATCGAAATATCGCAAATGTGCATAACGAGATATGCATATCGCAATTGTTTGCGATAAATGAGCGATTTAATACTTCAAATAGTAAAGTGTGGTCAAAGTTTTGGGATGGTAAAATATCTGGCACGCAGATGTTAGTTGGGATTTGCGTGCGCATGCTTTGACGCAAGACAATCAACAAGCTGCGAAGCTTTCATAGCAAACTAATCTTGATAAGATGAGCCAGCCCGCAGCTAAAATAGCTAATAAAAATAGCATTTCTCTAGGGAAACGTTATATTGCAAGAAATAGCATTATCGCAATGCTCAACAACAATAGCGCATATCGCATATTTTCCCAGTATCGTGCAGCCCTAA

>scaffold_21000042-2

TACGAGGGGGTATTAAAACGTTTCGAGACACACCAAGAGCAGCACAGGGAGCACAGCCTTGCATGCAGCCTTGTGCTGCCATCTGTTGGTGTGTCTCGAAACTTTTTGATACCCCCTCGTA

>scaffold_21000042-3

TCGAGGGATGAGAACCAGAAGGGCGTAAATAACATTTCACCAACTTTACAGGAGGGCCATAAACAAAAATGACCATAGTTTGATCTGACTTTGTGTACTGATTTCAATCTTTTATAATAAAAATGTGTACTATTATGGATGACAGCATACTTTGGCAGTAGAAATCTCCATTTCACCCAAAATGTCACTTACGCCCTTCTGGTTCTCACTCCTCGA

>scaffold_21000042-4

TACGAGGGGTTATCAAAAAGTTTTGAGACCTGCCAACAGATGGCAGCACAAGCAGCACAAGCCATCTGTTGGCGTGTCCCGAAACGTTTGATACCCCCTCGTA

>scaffold_21000042-5

GGCTACGTTCACACTGCAAGGCTTAGTGCTCAAATCCGATTTTTGCTCAGATCACATTTTTTGTACAGCTGTTTAAATTGTTGTTTTAAATGTGGCCAATATCAGATTTCCATGATGAACTGATCATGGTCCTAAACTGACCCGCATGAGCAAAAGAACAAATTCTACATCTCATGCAGCGTCCTGCCATACGGAAAAATAAACATGGAAGACACTGAATGCAGCGTTTACGTGTTAGGTGCTTACATTTATAAGGTGATGTGCAGCGGACGACAGTGAATTAATGAGCAGTCATTGAGGAGAAAAGGGCCACATATTTAATAATAGCATGTTGTGGTGCAGTGGCTGCTACTTCAGTATAGAGGTGTGTGTGGATGCGGAGCCGAAGCCAGGAGTGGTGGGACCGTGACGTGAACGCCTTCAACGAAATCGATTATATATCAACTGTTTGTAGTTACGCGCCAGTGCAGAATGATGACGCATGTCGATTGAAAATGACGTAAAAGTCGCATGAATTCTGATATAACTGTTCACACTGCGGTCGCTTTGCAAAACATCTGACCTGTCGGGTTTAATACCACATATGGAGGTGGCACAAATCGGAATTGAAAAGATCAGATTCCATGTGGTTTGTGCTGTTCACACCGTCATGAGAAAAACACATCTGAGTCACATGTGGGTGAAAAAATTGGATTTGAGCCACATTTGCCTGCCGTGTGAACGTAGCC

>scaffold_21000042-15

GTAGGGCTGTGTATTGGCAAGGGCCTCACGATACGATACACATATGTTGCAACACAATACATATTGTAAATTGTAAAAGAAATAGCTAAAAATACAACATGCTGTGTAGCACCTGGTGGATTCTAGTATGTGTATCACATTATATTGATTACTCAGCAGACAAAACTGAGGGTTTTAATTAAATATTAACGTCAACGTAAATCAGTTTAAAACATAATTTAAATATGTATCGATACTAGAGGTTAGAATATCGATCAACTATCGTGAAAAAAAATATCGCGATAGTTAGCTGTATTGATATTTTTACACAGCCCTAC

>scaffold_21000044-2

TATTCGCACTGGATTAGTATTATCTGGGGAGCTTGTGTGATTTAGAAATTACCCCCCCCCCCCCCCCCTTCTCCCATCTGAATTTCGTATGGCGTATTTGCACGGGATAATCGAAGCCTGTGATTTTACTTGAATTCACTGACCCGGATATGACGTCAAAACATTGGATAGGCTATTTTTTAGGAAATATCTTGTGGTTATATAGGTATAGATGTATAATATATCATAAACAGAAGAATGTTCCTTACCGCATGCCGCTATACATGTCATTTATCTCCATCTAATCATTCTGTTTGTTTTCGCTCTTCTGCTGGCGTTTAGTTTTATCTTTCTGAAAGTTGTATGGTTGTTTGGTGTATAGCGATTATGCAGCACCCAAAATACACCAAAACACTCCAATACAGCCTCATTCTTCACGCACGGTTAACAGAAACCTTAAAAAATGATACACGACCCAGCAAATCCTGACCAAATCACAAAGATGTCGATTCGCATGGGACTAATATCACGTGACCTCGGTGTTCGGCGAAATATGGTAGGTCATTTGTGGGAGAATTTTTACTTTACAAATTAGAGACATAGCCGATTCACACGGGATTAAGATCACAGACCACCTTCGCAATTATTACTAATAACCAGAGGTCCCCAGATAATACTTATCCCGTGCGAATA

>scaffold_21000044-10

TATCGTCTTGAGACCCCGCCCACTAACGTGTGTCTTCTGTAGTGGATATTTTGTTTTCATAAATGTTTTTAAAATGTTTTAAAAAAATTAATTTCTGCTGTAATGTACAGAGGACATCCTGGGTTTTCTAGTGATATGTTATTTGATTGGCTGGGATGCTAGGAACAAGTTCTTACACTCCATCCAAAAATGGCCGCCATAGGAACCAACACGTTTTATAAAAAGGACTGTGTTGCTGTAAAATGTAAAATCACAATCAGATTTAAAAACTTTAAATTGTAGAATGTTTTTTAACCCGAAAGGTGTAGGAAATCGCACAACACACAAAAAAATTGATGACACTTTTTTTCCCTTGGGTCTCAGGACGATA

>scaffold_21000045-2

ATACACTATATTGCCAAAAGTTTTTGGACACCTGACCATCAGATCCATGATCCATCCAAAATGTAGCTACAAATTTGGAAGCACACAGTCGTCTAGAATGTCTTTTCTATGTTGTAGAATTACATTATTCCTGCACTGGAACTAAAAGACCCAAACACTGTTCCAGCATGACAATGCCCCTGTGCACAAAGCCCCAGAGCTCCATTAAGACATGGTGTGTTATGATCGAAGTGGAAAAACATGAGTGTTCTGCACAAAGCCCTGACCTTAACACCACTGAACACTTTTGGGATGAACTGGAACACCGACTGCACCACAGACCTCCTTACCTGGCGTTAGTGTCTGATCTCACTAATACTATTGTAGCTGAATGAACACACATCTCACATTCAAGCTCCAAATTGTAGTGGAAAGTCTTACTAGAAGAGTGAAGCTTAATCCAACAGCAAAATGGGGAGGAAATCTGGAATAACATGTTTAAAATCACATATGTTTTAGGTAGTCAGGTGTCCACAAACTTTTTGACAATATAGTGTAT

>scaffold_21000045-3

GGCTACGTTCACACTGCAGGCAAATGTGGCTCAAATCCGATTTTTTCGGTCACATGTGACTCAGATGTGTTTTTCTCATGACGGTGTGAACAGCACAAACCACATGGAATCTGATCTTTTCAATTCCGATTTGTGCCACCTCCATATGTGGTATTAAACCCGACAGGTCAGATGTTTTGCAAAGCGACCGCAGTGTGAACAGTTATATCAGAATTCATGCGACTTTTACGTCATTTTCAATCGACATGCGTCATCATTCTGCACTGGCGCGTAACTACAAACAGTTGATATATAATCGATTTCGTTGAAGGCGTTCACGTCACGGTCCCACCACTTCTGGCTTCGGCTCCGCATCCACACACACCTCTTTACTGAAGTAGCAGCCACTGCACCACAACATGCTATTATTAAATATGTGGCCCTTTTCTCCTCAATGACTGCTCATTAATTCACTGTCATCCGCTGCACATCACCTTATAAATGTAAGCAACTAACACGTAAACGCTGCATTCAGTGTCTTCCATGTTTGTTTTTCCGTATGATAGGACGCTGCATGAGATGTAGAACCATGGACCATGATCAGTTCATACTGGAAATCTGATATTGGCCACATTTAACACAACAATTTAAACAGCTGTACAAAAAATCTGATCTGAGCAAAAATCTGAATTGAGCACTAAGCCTTGCAGTGTGAACGTAGCC

>scaffold_21000045-10

ATTAGGGCTGCACAATATATCAAAACTATTGAAATATCGCAAATGTGCATATCAAGATATGCATATCGCAATGGTTTGCGATAAATGAGCGATATAATAGTAGGATAGTAAAATATCTGGCACGCGGATGTTAGTTGGGAAATGCGTGCGCGTGCTTTGACGCAAGACAATCAACAAACTGCAAAGCTTTCATAGCGAACTAATCTTGATAAGATGAACCAGCCCGAAAAACACCCGCAGCTAAAATAGCTAATAAAAATTGCATTTCTCTAGAGAAACGTTATATCGCAAGAAATATCGTTATAGAAATACTTGACAACAATATTGTATATTTTCCCAGTATCGTGCAGCCCTAAT

>scaffold_21000046-2

TACACTATATGGCAAAAAGTTTATGGACACTTTAGCAGCACACCCATAAGTAAGTCGGAAGTACACAGTTGTCTAGGATGTCTCTACATGCTGCAGTTTTAAGATTTCCCTTCACTGGAACAAACACTTTCTCAAACAATGTTACAGCATGACAATGCTCCTGTGCACAAAAGCCCTGGGCTCCAAAAAGACATGGTGCTTTATGGTTGGAGTGGAAGAACTCGAGTATCCTGCAGAAAGCCTAGACCTTAACCCCACTGAACACCTTTGGGATGAACAGGTACGCTGACTGTATGTTAAAACTCAGCAACCAACATTAGTGCCTGATCTCACAAATACTATTGCAGCTGAATGAGCAAATCCCCACAGCCACACTTCACATTCTAATGAAAAGTCTTTCCAGAAGTGTGGAGGTTATTATAGGAGTAAATGTGAGACAAAATCTCAAATAAGACGTTAAAAACACACATACAGATTTGATGGTCATGTGTCTCAATACTTTTTGCTATATAGTGTA

>scaffold_21000046-13

AAGCCGGGCTTACATTATGCGATTTTCAATAGATTTTCGTGATTGTTGCTTGTCAGACTGTACGAACATGATCCCCTTGTCACACTGTAGGATATCAGTTGTCAGAATGTCAGACTGTACGACAGACAAGACGCGTCAAAAACAGACACACACAAGAAGACTCGTCCGGAGTTTTACGTCATCAATGTGTGACACGTTCAGTAACCAGCGCACTCTCGCTAAATGCTAGCTAGCAGCAAACAAACAGCAGCGACGACACGTCATGCAAACACTCCTTTTCCCTCCATAACTCATTAAGTTTCTCTTCTTGCTGTTCTGTCCACCTGAATTGTTTTGACATTTTTCCGAAATGGTTTGAAGTTGTTGCGCTAAGATTCGGCGCTCCTATTGGTTCTTGGTTTGTTGGTCGTCGTAGGAGAAGTCACACTGCAGGAAAGTGTCGGAAATCTTCTGACACTGCAGAGGAACAAACTGATCGCAAAATCGGCTGTCGGTGAACATGTCAAACTAGCCATCAAAGACAACAGAGTTTAGCGTAGAATTATAGGAATCTTTTAGGATTTCAAACTTTGTCTCAGACGACCAAATGTTTGCCAAAATCGCACAGTGGAAGTCCTGCTT

>scaffold_21000047-1

TACACTATATTGGCAAAAGTTTGTGGACACCTGACCATCACAAACATATGTGATTTTAAACATATTATTCCAGATTTCCTCCCCATTTGCTGTTGGATTAAGCTTCATTCTTCTAGTAAGACTTTACACTACAATTTGGAGCTTGAATGTGAGATGTGTGTTCATTCAGCTACAATAGTATTAGTGAGATCAGACACTAACGTCAGGTAAGGAGGTCTGTGGTGCAGTCGGTGTTCCAGTTCATCCCAAAGGTGGTGTTACTATACAGGTCAGGGCTTTGTGCAGAACACTCATGTTTTTCCACTTCGATCATAACACACCATGTCTTAATGGAGCTCAGGGTCTTTGTGCACTGGGGCATTGTCATGCTGGAACAGTGTTTGGGTATTTTAGTGCAGGAATAATGTAATGCTACAACATCCAAAGACATTCTAGACGACTGTGTGCTTCCAAATTTGTAGCAACATTTTGGAGAGGAACCACATATGGATCTGATGGTCAGGTGTCCACACACTTTTGGCAATATAGTGTA

>scaffold_21000047-2

CCAGGACTGGGTAACCCGTGTTCCTAAAGTTACTTGGTTCCGCTTGTTTTCCAGCTACCCCTGCCCTACCCACTGCTGATTACCTGCTTCAGGTGTGTTCAGTCAATCAGAAGCTGGAAGACACCATATTAGATGAGGGTGGAGTGGGGAAAGACCTGATGATTTGCTTTATCCTACCCTCTGATTGAACCAGGTAATCAGCAGTGGTAGCTGAAAAACAAGCGGAACAACGTAACTAAAGGAACATGGTTTACCCAGCCCTGG

>scaffold_21000047-27

ACTATATTGCCAAAAGTTGGCTGACAACTTACCAAGCACACTTACAATATATAAGTGTGTCTTCCCCAAACTGTTGTCACAACGTTGGAAGCACACAGTTGTTTAAGCTTTCTCTATATGCTGTAGCATTATATTTCTTTCAACTGGAACTAAGAGACCCAAAACTGTCCCAGCGTGACAATGCCCCTGTGCACAAAGCCCCAGAAATGACCAAAAGACATGGTGTGTTATGACTGAAGAACTTTAGTGTTCTACACAGAATCCCGACCTCAATCACACTCAATACTTTTAGGATGAACTGGAACACCGACTGCACCCCAGACCTCCTCACCCAACATTAGTGTCTGCTCTCACTAATACTATTCTGGCTGAATGAACACAAATCCCCAAAGTCACAATCCACAATCCAGTGGAAATAATTTCCAGATTATTACAGGAGTAAGAGTGAGATTAAATCTGGAATAGAATGTTCAATGAACACACATGGATGTAATAGTCAGGTGTCCCAATACTTTTGTGAATATAGT

>scaffold_21000048-5

TATATAACCAAAAGTTTGTGGACACCTAACCAGCACACCCACATTTGTGTGTTTTTCCAAAACTGTTGCCGCACACACTGTTGTCTAGAATGCCTTTGTATGTTGTAGCATTACATTATTCCTGCATTGGAACTGAGAGACCCAAACACTGTTCCAGCATGACAATGAAGTGTGCACAAAGTCCCTGAGCTTTATTAAGACACGGTGGGTTATGATTGAAGTGGACAAACTTGTGTATTCTGCACAAAGCGCTGACCTTAACACCACTGAACACTTTTGGGATGAACTGGAACACCGACTGCACCACAGACCTCCTTACCTGGCGTTAGTGTCTGATCTCACTAATACTATTGTAGCTGAATGAACACACATCTCACATTCAAGCTCCAAATTGTAGTGGAAAGTCTTACTAGAAGAGTGGAGCTTAATCCAACAGCAAATGGAGAAAAAATCTGGAATAAGATGTTTAAAATCACATATGATTTTGATGGTCAGGTGTCCACAAGCTTTCGGCAATATA

>scaffold_21000049-1

GGTCACACTTTACTTTAGGGACCAATTCTCACAATGAAATAACTATTAATTGCAACATCTACCCTAATAAACTTAGAATTTGCTTATTATGGTAGTTAGGTCTAGGTACGGGGTAAGATTAAAGGATATAAAATATGGTCATAGTATAAGTATAAGGCATTAATAAAGTATGTGCTTTATAATTAATGATAAACAGCCAAGATGCTGGTAATATAATAAACTAATAAGCAAATAGTTAATAGTGAGAATTAGTCCCTAAACTAAAGTGTTACC

>scaffold_21000050-1

TATTCACTATATTGCCAAAAGTTTGCTGACACCTGACCATCAGATCCATATGTGGTTCCTCTCCAAAATGTTGCTACAAATTTGGAAGCACATAGTTGTCTAAAATGTCTTTGTATGTTGTAGCATTACATTATTCCTTCACTGGAACTTCACTTGGAGAGACCCAAACACTGTTCCAGCATGACAATGCCCCTGTGCACAAAGCCCCAGAGCTTTATTAAGACATGGTGTGTTATAATTGGAGAAAATAACTTGAGTGTTCTGCACAAAGCCCTGACCTTAACACCACTGAACACTTTTGGGATGAACTGGAACGCCGACTGCACCACAGACCTCCTTACCTGACGTTAGTGTCTGATCTCACTAATACTATTGAAGCTGAATGAACACACATCTCACATTCAAGCTCCAAATTGTAGTGGAAAGTCTTACTAGAAGAGTGGAGCTTAATCCAACAGCAAAATGGAGATGAAATCTGGAATAAGATGTTTAAAATCACATAGGTTTGTGATGGTCAGGTGTCCACAAACCTTTGGCAATATTGTGTATA

>scaffold_21000053-1

TATTGGCAAAAGTTTGTGGACACCTGACCATCACAAACATATGTGATATTTTAAACATCTTATTCCAGATTTCCTCCCCATTTTGCTGTTAGATTAAGCTCCACTTAAGAAGACTTAAAAAGACTTTCCACTACAATTTGGAGCTTGAATGTGAGATATGTGTGTACATTAGTGAGATCAGACATTAATGTCAGGTAAGGAGGTCTGTGGTGCAGTCGGCGTTCCAGTTCATCCCAAAAGTGTTCAGTGGTGTTAAGGTCAGGGCTTTGTGCAGAACACTCAAGTTTTTCCACTCCAATCATAACCCACCGTGTCATAATAAAGCTCAGGGGCTTTGTGCACAGGGGCATTGTCATGCTGGAACAGTGTTTGGGTCTCTCGGTTCCAGTGCAGGAATAATGTAATGCTACAACATACAAAGACATTCTAGACAACTGTGTGCTTTCAAATTTGTAGCAACATTTTGGAGAGGAACCACATATTGATCTGATGGTCAGGTGTCAACAAACTTTTGGCAATA

>scaffold_21000057-3

CTATTCGTACGAGATTAGTATTATCTGGGGACCTTGTGTGATTTAGAACCCCCCCCCCCCACACACACACACACATCTGAATTTGGTGTGTCGCATTGGTACAGGATAAGCGAAGCCTGTGAATTGACTTGAATTTACTGACTTTATCTCCCGGATATGACGTCACAGCATTGGATAGGATATTGTTAGGAAATATCATGTGGTTCTATCGGTATAGATGTATGATAGATCATAAACAGAAGAATGTTCCTAATCGCATGCCGCTATACGTGTCATTTATCTCCATCTAATCATTCTTTTTGTTTTCTCTCTTCTGGTGGTGTTCAGTTTTATCTTTCTGAAAGTTGTATGGTTGTGTATAGCGATTATGCAACACCCAAAATGCACCAAAACACTCCAACACAGCCTCATTATTCACACACAGGAAACACGACCCCACAAATCCTGACCAAATCACAGAGATGTCCATTCACACGGGATTAATATTATCACATGACCTCGGGGTTCTGGGAAATATGGTAGCTCATTTACGGGGGAATTTTTACTTTACAAATTACAGACATGGCCGATTCACACGGGATTAAAATCACACACAACCTCCACAATTATTACACATAACCAGAGAATCCCAGATAATACTAATCCCGTACGAATAG

>scaffold_21000057-19

TAGGGCTGCACAACATATGAAAATTATCGAAATATCGCAAATGTGCATATCAAGATATGCATATGGCAAAGGTTTGCGATAAATGAGTGATTTAATACTTCAAATAGTAACGTGTGGTCAAAGTTAGGATAGTAAAATATCTGGCATGCGGATGTTAGTTGGGATTTGCGTGCGTGTGATTTGACGCAAGACAATCAAGAAGTTGCGAAGCTTTCATAGCGAGCTAATCTTGATAAGATGAACCATCCCGAAAAACACACGCAGCTAAAATAGCTAACAAAAATAGCATTTCTCTAGGGAAATTTTATAATCGCAAGAAATATCGCAATACTCAATATTGCATATTGCATACTTTCCCAGTATTGTGCAGCCCTA

>scaffold_21000060-2

TCCTATTCGCACAGGATTAGTATTATTTGGGGACCTCGTGTGATTTAGAAACCCCCCCCATCTGAATTTCATGTGGTGCATTCGCACGCGATAAGCAAAGCCTGGGATTTTACTGGAATTTACTGACTTATCTCCCGAATATATGATGTCCAAGCATTGGATAGGCTATTACTTGGAAATACATCTAGGCATAGATGTATGATATATAATAAACAGAAGAATCTCCCTTACCGAATGCCGCTATACATGTAATTTATCTCCATCTAATCATTCTTTTAGTTTTTGCTCTTCTGGTGCCGTTCAGTTTTATCTATCTGAAAGTTGTATGGTTGTTTGGTGTAAAGCGATATGCAGCACCCAAAATACACCCAAAACACTCCAACACGGCCTCATTCTTCACGCACAGGAAACACAAACCTTAAAAAATCATACACGACCCTGCAAATCCTGGCCAAATCACTGAGATGTCAATTCGCAAGGGACTAATATTATCACCGGACCTCTGTGTTTGGTGGAAAATGGTAGGTGATTTACGGGGGAATTTTTACTTTACAAATTACAGACATGGCCGATTCGCACTGGATTAAGATCACAGAAAACCTCCACAATTATTACAAATAACCAGAGGTCCCCAGATAATACTAATCCCGTGCGAATAGGA

>scaffold_21000060-11

TACACTATATGGACAGAAGCATTGGGACATCAGACCATCACATCCATATGCGTTTATTAAACATCCTTTCTTCAGATTCAGTCTCACTCTTATTCCAGTCATAACCTCCAAACTTCTGGAAAGGCTTTCCATTAGATGTGGGCGTGGCTCTGGGGATTTGTTCATTCAGTCACAATAGTATTAGTGAGTTGGGTGAGGAGGTCTGGGGTGCAGTCAATGTTCCAGTTCATCCCAAAGATGTTCAGTGGGGTTGAGTCAGAGTCAGGGATCTGTGCAGGACTCTCGAGTTCTTCCACTCCAACTAGAACACACCATGTCTTCATGGAGTTCAGGGATTTGTGCACAGGGGCATTGTCATGCTGGAACAGTGTTTGGCTCTATCAGTTTTAGTGAAGGAATAATGTAATGCTACAACATACAAAGACATTTCTAGCCATATAGTGTA

>scaffold_21000069-3

TGTACTATTTGCACGGGATTAGTATTATCTGGGGACCTCGTGGGATTTAGAAACTACCCCCCCACATCTGAATTTTATGTGGCATATTTGCACGGGACAAGTGAAGCCTGTGATTTTACTTGAAGTTACTGACTTATCTCCCGGATATGACGTCAAAGCATTGGATAGGCTATTGCTTGGAAATATCTTGTCATTATATAGGTATAGATGTATGATGCATCATAAACAGAAGAATGTTCCTTATCGCATGCCGCTATACATGTAATTTATCTCCATCTGATCATTCTTTTTGTTTCTGGTGGCGTTCAGTTTTATCTTTCTGAAAGTTGTAGCGTTGTTTGGGGTATAGCAATGTACAGCACCCAAAATACACCAAAAACACTCCAAAGCAGCCTCATTCTTCGCACACAAGAAACAGAAACCTTAAAAAAAAGATACACGACCCCACAAATCCTGGCCAAATCACAGAGATGTCGATAAGCACGGGACAAATTTTTTCACTGGACCTCGGTGTTATATGGTAGGTAATTTGGTGGGGAATTTTTACTTTACAAATTACAGACATGGCATGGCATGACATGGCATGGAATTAAGATCCCAGACAACCTCTGCAATTATTACAAATAACCAGAGGTTCCCAGAAAATACTTATCCCGTGCGAATAGTACA

>scaffold_21000080-7

ATGGCGAGTTTACACTGCACGATTTCCAAAGTCGTCGGATCACCTTTATTTTTAAACTGCACGACTATCTGGGGTAACATTCAGTTGCTGCTGTGTTCACATTGCACGATAGATAGGCAAGAGGAGGTTCCACGCTGCATAACTTTACAATAGGAAGAATCGCCAACAACTCTGTCTGGTACGCAAACTACATTTCACAACCAAACGCACGCGAGAAGTGATAAGAAAATAACACAAGGTCACGCATGAAATCAGAGTTCTCGCGTGAGACTGGAAATGTTTTTAAAATGGTACAAGTTCGCAATACAAATGGTCTGTGCGCTGATTTGCAGTGTAAATGCAAAAAGAAAAAAGGTTAAAAAAAAAGATCGGAACCAAAGCCATGCTTGCTGATATTGTGATCTATAACTCTTCCCCAAACTTCCCTCTGCCCTGTATCTTGCTCCCTCACTGGCTGTAGATCATCGCCGATGTATTTTTCAGTCAGAACTCATTTCACACAGCGGGATTTTGAATCGCTGACAGATATTTAGCATGGCAAATATTTCACAGGCGTAGGCGACACGATTCACTCAGATTGCGTCTTTGATCATTCACACTGCGTGATTGTCACTAATGTGAATGAGCACCGATTTGCCTCTGATTTCGGGCAGTTGTCGGCGATTTCGCAAAACCTGTCGGCAATTGAAAATCTGGGCTAAAATCATGCAGTGTGAACTCGGCAT

>scaffold_21000083-2

TAATATACAGCTCTGGAAAAAATTAAGAGACCACTTAAACTGATCTATTTCGTTGGTTTTACTATTTATAAGTGTGTTTAAATAAAATTGACAGTTTTGTTTTATTTTTTAAACTGCTGACAATATTTCAGGTGATCACAGAATCAGTACTAGAGAATCTGATGTGCCAATATTAGAATGAAAAAACACAGGAATGGAATAGCTGTCATACATGTAGAGATGCTGATTTTATAAAACATTGGAGTGATCTCTTAATTTTTTCCAGAGCTGTATATTA

>scaffold_21000086-1

CTTAATGCTGAGTTCACACTGCACAATTTTTCACTCGCCGACAGGTTTTTAGGAATCGCCAAGGAATGCCTGAAATCAGAGGCAACTCGCTGCTTGTTCACACGAGCGACAATCGCGCAGTGTGAATTATCAAAGACACGATCAGAGAGAATCGCCGAGGCGTCGCCAAGCCCCGTGAAATATTTGGCATGCTAAATATCTGGACTTGTCGGCGATTCAATCTATTGCTGTGTGAAATGAGTTCTGAGTGAAAAATTCACCGGTGATGACCTACAGCCAATGAGAGAGCAAGATACAGGACAGCAGGAAGTTAAGAGAGGAGTTATAGACCATAATATCAGCTAGCATGGCTACGGTTCCCATCCTTTTTTTTCTTTTTCACTCTTTCACTGTAAATCAGCGCGCAGACCATTTGAATCGCAAACTTCTTGCGGGCTGCTTTTTTAATAACATTTCCATTCTCGTGCGAGAACTCTGATCTCACGTGTTATTTCAACATCACTTCTCGCGTGCGTTTGGTTGTGAAACGTAGTTTGCGGACCAGACAGAGTTGTTGGCGATTCTTCCTATTGTAAAGTCATGCAGTGTGTAACCTCCTGTCGCCAAGCTATCGTGCAATGTGAACACAGCAGAACTGAATGTTACCCCAGATAGTCATGCAGTGTGAAAACAATGGTGATCCGACGACCTGAAAATTGTGCAGTGTGAACTCGGCATAAG

>scaffold_21000087-2

ATGCTGAGTTCACACTGCACGATTTTTCACTCGCCGACAGGTTTTTTTGAATCGCCGATCAATGCCCGAAATCGGAGGCAAATCGCTGCTCGTTCACGCGAGCGACAATAGCGCAGTGTGAATTATTAAAGACGTGATCAGACAGAAAAGCCGAGGCGTCGCCGAGCCCCGTGAAATATTTGGCATGCTAAATATCTGGACTTGTCGGCGATTCAAACTATTGCTGTGTGAAAGGAGTTCTGAGTGGAAAATTCACCGGTGATGACCTACAGCCAATGAGAGAGCAAGATACAGGACAGCAGGAAGTTAGGGGAGGAGTTATAGACCATAATATCAGCTAGCATGGCTACGGTTCCCATCCTTTTTTTTCTTTTTCACTTTTTCACTGTAAATCAACGCGCAGAACATTTGAATCGCAAACTTTTTGCGGGCTGCCATTTTAATAACATTTCCAGTCTCGTGTGAGAACTCTGATCTCACGTGTTATTTCCTATCACTTCTCGCGTGCGTTTGGTTGTGAAACATAGTTTGCGGACCAGACAGAGTTGTTGGCGATTCTTCCTATTGTAAAGTCATGCAGTGTGTAACCTCCTGTCGCCAAGCTATCGTGCAATGTGAACACAGCAGTAACTGAATGTTACCCCAGACAGTCGTGCAGTGTGAAAACAATGGTGATCCGACGACCTGAAAATCGTGCAGTATGAACTCGGCAT

>scaffold_21000089-2

TCGAGGGGTGAGAACCAGAAGGGCGTAAGTGACATTTCACCAACTTTACAGGAGGGCCAAAAACAAAAAATTACCATAGTTTGATCTGACTTTGTGTACTGATTTCAATCTTTTATAATAAAAATGTATACTCATATGGATGACAGCATATTTGGCAGTAGAAAGAGCTCATCAAGAGCTTTCATTTAATATATATATATCTCCATTTAACCCAAAATGTCACTTACGCCCTTCTGGTTCTCACTCCTCGA

>scaffold_21000091-1

TAGTTTAGGGATCAATTCTCACTATTACAGTAACTTGTTGCTTATTAGCATTTATATTAATAGCATATTGGCTGTTTATTGGTACTTATAAAGCACATATCAATGCTGTATACTTTATAACCATATTTTAGTTCCCTTAATCCTACCCCACACTTAAAACTACCTTACTAGCTATTAATATACTGCAAATTAGGTGTTTCAAAAATGCAGAATTTGTAGTTAATAGTTAGTTCATAGTCAGAATTGGTCCCTAAGCTA

>scaffold_21000092-1

AGGCTACGTTCACACTGCGAGGCTTAGGGCTCAATTCCGATTTTTGCTCAGATCAGATTTTTTTGTATAGCTGTTTACATTGTTGTTTTAAAATGTGGCCAATATCAGATTTCCAGTGTGAAATGATTATGGTCCTAAACTGACCCGCAAGCGCAAAATAACTAATACTTCGTCTCACGCAGCGTCCTGTCATACGGAAAAATAAACACGGAAAACAATGAACGCAGCATTTACGCGTTAGGTGCTTACATTTATAAGGTGATGTGCAGCAAAGGACAGCGAATTAATGGGCAGTCGTTGAGGAGGAAGAGGATGAGGAGAAAAAGGGGCCACATTTTTAATAATAGCATGTTGTGGAGCAGTGGCTGCTACTTCAGTATGGAGGTGTGTGTGGATGCGGAGCCGGAGTCAGGAGTTGTGGGACCGTGACGTGAACGCCTTCAGCGAAATCAATTATATATCAACTGTTTGTAGTACTTGCCAGTGCAGAATGATGACGCGTGTCGATCGAAAATGACGTAAAAATTGCATGAATTCCGACATAACTGTTCACACCAGGGTTGCATTGCAAAACATCTGACCTGTGTCGGATTTAATACCACATATGGAAGTGGCACAAATCGGAATTGAAACGATCAGATTCCATGTGGCTTTTGCTGTTCACACTGTCATGAGAAAAACAGATCTGAGTTACACGTGGGCAAAAAAATCTGATTTGAGCCACATTTGCCTGCAGTGTAAACGTAGCCT

>scaffold_21000097-1

TACACTATATGGCCAAAGATTTGTGGACATGTGGCCATCACAACTATGTAATGTTGAACATCCTGTTACAGATTTATCCCACTCCTTTGCTGTTTTAATAACCTACACTCTTTCGTAAAGCCTTTTCAGCTAATTTTTTAACATTGCTGTGGAATTGTGATCATTCAGCTAAGGGCAGCTAAGGGCATTATTAAGATCAGTCACTGATGTTGGGTGAGGAAGTCTGGGGTGCAATCGGTGTTCCAGTTCATTTCAAAAGTGCTCATTGGGGTTGTGGTCAGGGCTCTGTGCAGGAAATTTAAGTTCTTTAACTCCTGCCATAACAATATGGAGTCTTCATAAAGCTCACAATGTGCGAAGGGGCATTGCCATGCTGCAAAAGTGTTCAAGGTTCCTAGTATTATACTGTAATGTTACAGCACACAGTGCATTTTACACAATCAAGTGTTTCTAAATTTGTGCCCACAGATTGGAGAAGAATCACACAATGATCTGATGTGAATTGTACACAAACATTTGGCCATATAGTGTA

>scaffold_210000106-1

TATATACAGGGTGGGTGAAAAGTAACTAGGCATTAAAAATTGGTAATAAAGTCCATATTTCTAATACAAATTTATTGAAACAAATTGTACATGTACTTAATTACATAGGAAAATGAAAGCAAATCTTTAAATTTCAACGTAGGCACCGGTGGCGTGAACCAGTTTCCTCAAACAGCCAGAGATGGAATGCACAGTCGTCTGAAGAAAGTGGTTTGGGATATTGCTGAGAACCTCCCGAATCCGTGCCTTCAAGTCTTCCAGTGTGCGAGGCTTGAAATCCTATTTACTATCGTCTATTTAGAGACTGTTTTCATCATGACTCCAGTGATACTACAAGTTTTATAGCCATTAAATATTGCCTAGTTAATTTTCACCCACCCTGTATATA

>scaffold_210000113-3

AGGGTGTGTTCACAGTTGTAGTTCGGTTCATTTGGTCAGGACCAAAAAGGAAAATGATACATTTACGTTCACATTGGGATTTTTGACAGCGAACCTAAAGATACCGAACCTAAAGGCATAGTGATACGTTCACAAACTGATTGGTCGGCTTTTGTGACGTATATTTTGTGACAGAACATAACATTCGAAAACGATGCCGTGTAATAAATTAAGCACGCTCATTGTGTGCATGTATATATGGTTTTACTTTTACCAGCTGAGAACGCAAGAAGAGCTCCTAAAATATTCAAAACAGCACCAGGATGTCCTCCGTTGTATGCAGAAACAAAGATCTGCTGCAAGGAGACGGCGTTTCTGATGCCTTTTTCGACTAATAAGCAACAAAGGTCCTTTAATGACATGAACCAGGTATGCTTGAGCATTCTGTCCTTTTTTTACATCGCTCTTTTTACCATGTGACTTGTGGCGTCGCACCTTGTGACATCACATCCTGTTTTTGGTTCGATTAGATATATTTGGTCCGTGTTGCATTCATATTTCAATCGATGCGCACCAGAGTTTGTTTGGAAGCGGGCCGAGGCCCATCTTTTTAGCGGTTTCAGTCCGCTTGTTTGGTGCGCATCAGGGTTCGGATGGCAGCGTTTACATTTACTCAAATGAACAGCACTAACTAGCAATCGCTCCAGAGTTTGTTTTAATCGAACCAAACATGACAAGTGGGAACACACCCT

>scaffold_210000113-15

TAGGGCTGTGTTTTGGCAAGAGCCTCACAATACGATACGTATCACGATACATGGGTCACGATACAATATATCACTATATTGCAATACAATACATATTACAATACATTGCAATACTTCAAAAATACAATTTTGTATGTAAAACAAATAGCTAAAAAATACAACTTGCTGTGTAGCACCTGGGGGAGTCTAGTATGTGTATCACATTATATTGATTACTCAGCAGACAAATAAAAACTGAGGGTTTTAATGAAATATTAACGTAAACGTAAATCAGTTTAAAACGCAATTTAAATATATATCGATACTAGAGGTTAGAATATATTGATAAACTATCGTGAAAAAAATATTGCGATAGTTAGCTGTATTGATATTTTTACACAGCCCTA

>scaffold_210000118-2

TTAAAGCTGCAGTCCGTAACTTTTGGGGGGTTAAAATCATTCAAAATGAATTATTGAGCAAGTACATTACCAGCCAGTGTTCAAAACTATCTCCTTATCTTCACCCAATTCACAACGGTAATATTGTAATAATGTTTTATTGTTTGTGTGGTACTGGTAGGTTTTTGCGGGAAATAAGAGCATGGCACCGTTTGTCTTTGCGTCATTACATCACGTCTGTAAACAAAGTAGCAGTCCCGTCTATATCGCATGCGAGGATGTTAACGCTAACGCTAATACACACTAAACACACATAGTTACGCAATGCTGATGTTGTTAAGATTAACAATTTGAGAACAAAGTATAACAATAATAATAATTTGCATGGTTTGATGGGATATGAGCTAATTGGTGATCGTTAGATTTAATAACTATCAATAGCGCGATAAATTGCAATGCATTTTTCCCCTCAATTGTTCAAAACAAAAGTGGCAGACGTTACTTACCTGTTCAGATGACATTCTCCGGTGACAATTCTAATTTGGGTCATACTTTCAAGATTCCAAAGTGCAGTATCCTCACCGGTGTGGTGACTGACAGCCCACACACACACTGCAAAACATTACATTTACCCGCCCCGAGGAGCCGTGCCGATACACAACCCACGTAAAGATCATTCCGCAAATAACTGCAATTGCAGGTTTCAAACAGAGATGGCGACAAAGAGGCAAAACTTACGGACTGTAGCTTTAA

>scaffold_210000118-4

TTAAAGCTACAGTCCGTAAGTTTTGCCTCTTTGTCGCCATCTCTGTTTGAAACCTGCAATTGCAGTTATTTGCGAAATGATCTTTACGTGGGTTGTGTATCGACACGGCTCCTCGGGCGGGTAAATGTAATGTTTTGCAGTGTGTGTGTGTGGGCTGTCAGTCACCGCACCGTTGAGGATACTGCACTTTGGAATCACAGATTCTACATCTTGAAAGTATGACCCCAAATTAGAATTTTCACCAAAGAATTTCATCTGAACAGGTAAGTAACATGTCTGCCACTTTTGTTTTGAACAACTGAGGAGAAAATGCATTACAATAAATCGTGCTACCGATAGTGATTAAATCTAACAATTACCAACTAGCTTACAAACCGTGCAAATTATTATTATTGTTACACTTTAATCTCATCAGCATTGCATAACTGTGTATTTAGTGTGTATTAGCGTTAACATTCTCGCATGCGATATAGCCGGGACTACTACTTTATGTTTACAGACGTGACGTAATGACGCAAAGACAAATTATTTCCTGTAAAAACCTACCAGTGCCACTCAAATGATTAAAACATCTTTACAAGATTACCGTTGTGAATCAGGCAAAGATAAGGAGATAGTTTTCAACACTGGCTGGTAATGTACTTGCTCGGTAATTAATTTTGGGATCATTTTTAACAGAAAAAAGTTACGGACTGCAGCGTTAA

>scaffold_210000118-13

CTTTAACCCCTTGTTGCCTGAATTTTTACAATAAAAAAAAAGAATACGCATTAACCGGTATTGGGAAGACACATACGCCACATCAGCTGGATTCAGGCCGGAAGCGGTTTGAGGCGAATTCACAACAATTGTCTAAAAGGGGTTAAAG

>scaffold_210000120-2

ATATATCCTCCTGAGACCCAAGGAAAAAAAATGTCAGCATTTTTTTTGTTGTTTGGTGTGATTTTCTACTCCTTTTAATTTGAAAATAAGTTTCTACAATTTAGAGTTTATAAACGTTATTGTTATTTTTCATTTTACAGCATGTCCACTGTACTGGGCCACTGGACCATTTTAGTTTGAAAAGACAGTCCTTTCCATAAAGCATGCTCATTCCGATGTTGGGCATTTTGGATGCAGTATAAGAACCAGTTCCTAGCATCCCAGCCAATCAAATGACATATCACTAGAAAGCCCAGGCTGTCCTCCGTACAGTAACAGCAGAAATTGTTAGAGTAGCTCAAAAAATTCAGAAAACATTTATGAAAACAAAAGTCAATGGGCCGGGTCTCAGGAGGATTATAT

>scaffold_210000122-12

TAGGGCTGTGTATTGGCAAGGGACTTACAATATGATACATACATGGGTCACGAGACAATATATCACGATTTATTGCAATACATATTGCAAAACTTTTAAGAAGAAAAAAGAATTTTTTGTACCACTTGTGGGATTCTAGTATGTGTATCACATTATATTTATTGCTCAGCAGACAAATAAAAACTGGGGGTTTTAATTCAATATTAACATAAACGTAAATCAGTTTAAAAACGTAATTTAAATATGTATCAATACTAGAGGTCAGAATATTGATACACTTTCATGAAAAAAATATGGCGATAGTTAGCTGTATCGATATTCTTACACAGCCCTA

>scaffold_210000126-2

AGATACACTATATGGCCAAACGTTTGTGGACACCTTACGAGCGCACTCATAAATCTGTCTTCCCCAAAGTGCTGCAACAAAGTTGGAAGCACACACTGGTCTAGGAGGTCTCTATATGCTATAGCTTTAGCATTTTCCTTTATTGGAACTAAGAGGCCCAAACAATGTTATAGCATGACAACACCCTGGTGCACAAAGCCCCTGAGCTCCATGAAGACATGGTGTGTTTAATTAAAGTGAAAGTACTCAAGTGTTCTGCCTCAATTCCACTTAACACATTGGGGTGAACTGGAACACTGACTGCACCCCAGACCTCTCCACCCAACATTAGTACCTGAACTCACAAATAAATGTTGTAGCTAAATATGAAAAACAATTCTCTTTACTATTTAGTGGAAAGCATTTCCAGAAGTGTGGAGGTTATTATAGGAGTAGATGTGAGACTAAATCTGAAATAGGATGTTAAACAAACACATATGGATGTGATGGTCAGGTGTCCAGATACTTGTGGCCATTATAGTGTATCT

>scaffold_210000126-5

ACTAGGGATGCACCAATACCATTTACTGACTGAGACTGAGATACGAGTACCAATATTTTTTTTCTGGTACTCGCCGATACCAATACGATGCCAATACCTGTACTTTTTTGGGATGTGGGATTATTTGTATGGTTCTCGGGTTATCTCTTATCTCTTTTCTCTCGTCTTGCAATTGTTTGCTGCAGGGTTGGTTGCCGAGTGCTAACGTTACTGCTAGCAGCAAAGTCTCCAAAAAAGTACTTTCTTTTGTGTTTTAATTTCAGATGTTATATCAGATTACTCGTATTATAAGTGCTCATTTTTGTACCGCTTGATATTTTTGCTGAACAAAGTTTGCAGTCTGCCATGCGTGGGTTGTCATCATTAATTTAGAAATATTTCCACACCGCTGAGCCTGACATTCCGTCGTTGCTGCCAAAGTGTTTGTGCACTGAAAGTTCTGCCAATGACTTCACAAGAGGTATCGGTCTTTGGTATCAGAGTGTTTTTACGAGTACAAGTACATGAGCTCAGTATCGGGCCCAATACCGATACCAGTATTGGTATCAGTGCATCCCTAGT

>scaffold_210000128-1

ATGCCGAGTTCACACTGCAGGATTTTTAAAGTTGTTGGATCACCGTTGTTTTCACACTGCATGACTATCAGGGGTAACATTCAGTTGCTGCTGTGTTCACATGTATGATAGCTCGGCGACAGGAGGTTACACACTGCATGACTTTACAATAGGAAGAATCGACAACAACTCTGTCTGGTCCGCAAACTACGTTTCACAACCAAACGCACGCGAGAAGTGATGAGGAAATAACGCGAGATCACACGTGAGACCAGAGTTCTCGCACGAGACTGGAAATGTTATTAAAATGGTAGCCTGCAAGAAGATCGCCATACAAATGGTCTGTGCACTGATTTGCAGTGAAAGGGTGAAAAAGATTACAAAACCGGAATGGGAACCGAAGATTTCCCATAAGTCTATAACTCCTCCCCGAACCTCCTGCTGTCCTGTATCTTGCTCTCTCATTGGCTGTAGGTCATCACCGGTGAATTTTTCACTCAGAACTCATTTCACACAGCAATATTTTGAATCGCCGACAGCTCCAGATATTTAGCACGCCACATATTTCACGGGGCTCGGCGACGCCTCTGCGATTCTCTCTGATCGCGTCTTTGATAATTCACACTGCGTGATTGTCGCTCGCGTGAACGAGCAGCGATTTGCCTCCGATTTCGGACATTGCTCGGCGATTTCGCAAAACCTGTCGGCGAGTGAAAAATCGTGCAGTGTGAACTCGGCAT

>scaffold_210000133-1

ATATACAGCTCTGGAAAAAATTAAGAGACCACTGAAACTGATCTATTTCGTTGGTTTTACTATTTATATGTGTGTTTAAGTAAAATGGACAGTTTTTTGTTTTTTTTTAAACTGCTGACAATATTTCAGATGATCACAGAATCAGTACTGGAGAATCTGATGTGCCAATATTAAAATTCAAAAACACAGGAATGGAATAGGTGTCATACATGTAGAGATGCTGATTTAAAAAAAAAAAATTGGAGTGGTCTCTTAATTTTTTCCAGAGCTGTATAT

>scaffold_210000141-4

ATGCTGTGTTCACACCAAACACGGATGTAATTATTCGCGCGTGTAGATTACATACAAAGTCGATGCAAAGACGCGAATAGAGGCGAATGGCGCGAATGCTGCGAACGTGCAATTATCGCCTTAATCGCGTCTTCCGCGCAAGTTGAAAAATTGCAACTGAAGCGGAAAATCCGCATGGCGCGTTGAGAGAGAGAGAGAATAAACAAAGAATCAACAAAGAGTTTATTGACACGTCCGTTGTATCTGACAATGGAGAAGAGCCTTATTGTAGCCGTCTGCGGCCACTTCAAAATTGATGTTTTATGAATAAACACATTGCGCGAGTGACGCGAAAAACATCCCACAAGTCATCGAGAGTGAGTGACGCGATGCACATATTTGCTTTGCGTTTGGCGTGAACACAGCAT

>scaffold_210000142-3

ACTTAAGCCAGGCTTACACTGTGGGGTTTTGGCCACGGCTTGGTTGTCTGAGACAAATTTTGAAAACCCTTAAAGATTCCTATAATCCTATCCTAAAATCAGTTGTATTTGATGGCTAGTTTCAAATGTTCACCGACAGCCGATTAATGACCGTTGCGAGAATTTTTTTCCTTTGATGAAATTCTGGCAGGGTGAGAAGATTTCAGACACTTTCCTGCAGTGTGGCGTCTCCTACGACGACCGTCAAACCAAGAACCAATAGGATAACGCAATTAGTGCGACAACTTCAGACCACCTCAGGAAAATGTCAAAAAGAGTCAGGTGGACATGTACAGCAAGAAGAGAATCTTATTGAGTTATAGAGGGAAATTAAGTGTTTGTATGATGTGTTGTTGCTGCTGTTTGTTTGCTGTTAGCTACCGTTTAGCGAGAGAATGCAGGTTACAGAACGTGTCATCAATTGATGACGTAAAACTCCGGACGAATATTCTTATGTGCGTCTGTTTTTGACACGTCTTGACCTAAAGTATGACATGGAGATCAGTCTGACAAGCAACACTCGCAAAGGACTATTCAAAATTGCACAGTGTAAACCCGGCTTTAGT

>scaffold_210000144-5

TTAACCCTTGTGTGGTGTTTGTGTCTGTGGGACCCGATTTCAAAGTTTGTTTAAAGAGAAATTATGCTATTCATTATTTATTTCCATGTTGAACATAAAATAGAACATATTTTCCATAACACTTATATTTTCAAATGTGATCTAATGGGATAAATGGCTAATATTAACCATAAATTATGTCTATATTGCTTGTAAGTGAGCTAAAGTCCCTTTTAAGTGTTTTTCCATAAATTGTTATGGTGGTGTTGATTAAAAACCCAATAATGCGGTGGGTCCACCAGAACAGTGAACATTGGCTAAGTAACAAAAACATGAACACCACATGAGGGTTAA

>scaffold_210000145-1

TCTAATGTACTATTCGCACGGGATTAGTATTATCTGGGGACCTCGTGTGATTTAGAAATTACCCCCCACATCTGAATTTCGTGTGGCGCATTCACACAGGATAAGCGAAGCCTGTGATTTTACTTATGACTTATCTCTCGGATATGATGTCAAAGCATTGGATAGACTATTGCTTGGAAATATCTTGTGGCCATATAGGTATAGATGTATGATTTATCATAAACAGAAGAATGTTCCTTACCACATGCCGCTATACATGTAATTGATCTCCATCTAATCATTCTTTTTGTTTTGGCTCTTCTGGTGGCGTTCACTTTTATCTTTCTGAAAGTTGTATCGATGTTTAATGTATAGCGATATGCAGCACCCAAAATACACCCAAAACACTTTAACGCAGCCTCATTCTTAATGCACAGGATGTGTGTTTATACACGACGAAAAATGATACACGACCCCACAAGTCCTGACCAAATCTCAGAGATGTCGATTTGCACGGGACTAATATTATCACAGGACCTCGGTGTTCGGCGAAATGCGGTAGGTCATTTGCGGGGGAATTTTTACTTTACAAATGACAGACATGGCCGATTCGCACGGGATTAAGATCATAGACAAACCTCCGCAATTATTACAAATAATTAGAGGTCCCCAGATAATACTTATCCCGTGCCAATCGGACTTTAGA

>scaffold_210000147-1

TATTATTAGGGCTGCACAATATATCGAAATTATCCAAAAATCGCAAATGTGCATATTGAAATATGTATATCGCAATGGTTTGCGGTAAGTGAGCGGTTTAATACTTCAAATAGTAATGTGTGGTCAAAGTTTTAGGATAGTAAAATATCTGGCACGCGGATGTTAGTTGGGATATGCGTGCGCGTGCTTTGACGCAAGACAATCAACAAGCTGCGAACCTTTCATAGCGAGCTAATCTTAAGAGGAGCCAGCCCGAAAAACACCTGCAGCTAAAATAGCTAATAAAAATAGCATTTCTCTACGGAAATATTATATCGCAAGAAATACCGTTATCGCAATACTCAACAACAATATTGCATATTTTCCCAATATCGTGCAGCCCTAATTATA

>scaffold_210000149-1

TATATATACAGCTCTGGTAAAAATTAAGAGACCACCCCAATTTTTTTATAAAATCAGCATCTCTACATGTATGACAGCTATTCCATTCCTGTGTTTTTGAATTCTAATATTGGCACATCAGATTCTCCAGTACTGATTCTGTGATCACCTGAAATATTGTCAGCAGTTTAAAAAATAAAACAAAACTGTCCATTTTACTTAAACACACCTATAAATAGTAAAACCAACGAAATAGATCAGTTTAAGTGGTCTCTTAATTTTTTCCAGAGCTGTATATATA

>scaffold_210000151-1

TATAGACAGGGCTAGGTAACCCGTGTTCCTTAAATTACTTTGTTCTGCTTGTTTTCCTGCTACCCCTGCCCTACCCACTGCTGATTACCTGGTTCAGGTGTGTTCAGTTAATCAGAGGGTAGGAGATACCATATCACCAGGTCTTCCCCCACTCCACCCTCATCTGAAATGGTATCTTCCAGCTTCTGATTGAATAAACACACCTCAACCAGGTAATCAACAGTGGGTAGGATAGGGGTAGCTAAAGCGGACCAAAGTAACTTAAGGAACATGGGTTAACCAGCCCTGACTATA

>scaffold_210000151-13

ACTATATGGACAAAAGTTTGTGGATACCTAACTAGCACACCCATAACTGTTGCTACACATGTTAAAAGCACACAATTGTTAAGGAGGTTTCTATATGCTGTAGAGTTAATATTTCCTTTCTATGGAACTAAGAGGCCAAAACACTGTTCCAGCATGACAATGCCCATGTGCACAAAGCCCCTGAGCTCAATGCAAACGTGGTGTGTTATAGTTTGAGTGGAAGAACTCGAGTATCCTGCACAGAGCCCTGACCTCAACCTCACTGAACACTTTTGGAATTAACTGGAATGCCAACAGCACCCCAGACCTCCTCACCCAACATTAGTTCCCGATCTCACTAATACTAATGTGTCTGAATGTACAAATTTCCACAACCATGCTTTATAATCTAGAGGAAAGCATTTTCAGCAGTTTGAAGGTTATTATAGAAATAAGATTGCGACTAAATCTAAAATAGGATGTTTAAAAAAACATATGAATGTGAAAATCAGATTTCCCAATACTTCTGTTCATATAGT

>scaffold_210000156-1

AGTACAGTAAGACCTCGATTCACGAACACATTAACCCACGAACAAATTGATTCATGAACAAATATATATATAAAAATGCTGTTTGATTCACAAACTCATTTTTGAAACACAAACATGGTCCGATGGCCAAAATTTGAAAAATGCGGGCAATCGCTCATCTTACGTGGGTGCATTCGAGCCGCTATGGCGGCCGCCATCTTGTTAGTGTAGGGTACTATACATCTCCCACGCACTTCCACCCACGCTTAGTCTGTTTTTGATTTTGGTCTTGAGTAGACGCGCGTTCAAACGCTTCAAACTCCTACCCACCCTCCTCTTCTCTCGTCCGTCAAGCCAGAAGTCGTGTCAGCCAACGTAAGTGTTCACTTTTTAAATGTTTTTTTAGTTTGTTTACTATTATAAATTATATTATTAGATATATTAAGGTTTTTCACACTTTGTTTAATATTATCTGTGTAAAATAAAACAAAATACATAATTTATAATGGTTCGTTAGGGTCATGAACAAATTAATCGTATTCCCATTAAACCTTATGTGAAAATTAACTTTGGGTCACGAACATATTGGAACACAAAGTTTAGGAACGAATTGTGTTCGTGAATCCAGGTCCTACTGTACT

>scaffold_210000157-3

TCTTAAGCAGGGCTTACACTGCGATTTTGGCCATGATTTGGTTGTCTGAGAAAAATGTTGAAATCCTAAAAGATTCCAGTAATCCTAAACTAAAATCTGTTGTCTTTGATGGCTAGTTTGACATGTTTACCGGTACAGCCGATTAATGGCCGTTTCTGGCTCTGTCCGAAGATTTCAGACACTTTCCTGCGGTGTGACGTCTCCTAAGACAACCGTCAAACCAAGAACCAATAGGAGCGCCAAACCTGATGACGCAATTAGCACAAGTCTGGTGGACATAACAGCAAGAAGAGAAACTTATTGAGTTATGGAGGGAAAAGGAGTGTAGACATGTTGGCTGCTAGCTAACATTTAGTGAGAGAATGCAAGTTACTGAACGTGTCTCGGTTTGATGACGTAAAACTCTGGACTTTTTTAGTGTGCATCCGTTTTTGACGCGTCTCGAATGTCATGCAGCCTGACATTATGACAACTGAGATCCTACAGTGTGACATGGGGATCATGTTTGTACAGTCTGACAAGCTACAATCGCAAAAGACTATTATAAATCACACAGTGTAAGCCCGACTTAAGA

>scaffold_210000159-11

TAGGGATGCACCAATCCCGATACTGGTATCAGTATCAGGCCCGATACTGAGCTCATGTACTCGTACTCATAAAAACACTCTGATACCAAAGACCGATACCTCTTGTGATTTCATTGACAGAACTTTCAGTGCACAAAGACTCCGGTAGCAGCAACAGATTGTCAGGCTCAGGTGTGGAAATATTTCAATATTAATGATTGCAAACTTTATTCGGCAAAAATATCAAGATGTTCAAAAATGGGTATTTATAATACGAGTAATCTGAAAAAATATCTGAAATTAAAACAAAAAAGTGAGCACAGTGAATTTTCCGCTAGCAGTAACATTAGCACTCGGCAACCAACCCTGCAGCAAACCCCTGCAAGACGAGAGAAAGTGGCAAGAGATAACCCAAGAGCCATACAAATAATCCCACATCCCAAAAAAGTACAGGTATCGGTATCGGCGAGTACCAGAAAAAATATATCGGTACTCGTACTCGGCCTTTAAAAAATGGTATCGGTGCATCCCTA

>scaffold_210000161-7

TACCCAGCTAACAAATTTATGTTCTCAAACAGTTTTTCAAACGTTCTGTTTTGGTTTCAAGAACGTGACTTGAAATGTTCCAAAAACGTGAAAATGTCCAGTTTGCTTAACGCTGGTAGAACGTTATGTATGGTTATTTTATGGCGTTTTTCTAAGAACTATTTCTCTATACATTTTGAGAACATTCATCACATACAAATAACGGATGTTTCAAGAACATTGTTCTATAAACGTTCTCTCAACATTTTGAGAACATTCATCTAGGACGTTCCAAGAACATTGTTTCAAGAAAATTTTCTCTCAACATCACAAGAATGTACAATAACTAATTAATAACATTATGAGAACATTCCATGCACATTATTTTAATAACATTTTTCCCTAACATTTAGAAAACTTTTTAAAGAACATTAGTGAAAGTTGTGGGAACGTTCCCTGTTAGCTGGGTA

>scaffold_210000161-10

TACCCAGCTAACAAATTTATGTTCTCAAACAGTTTTTCAAACGTTCTGTTTTGGTTTCAAGAACGTGACTTGAAATGTTCCAAAAACGTGAAAATGTCCAGTTTGCTTAACGCTGGTAGAACGTTATGTATGGTTATTTTATGGCGTTTTTCTAAGAACTATTTCTCTATACATTTTGAGAACATTCATCACATACAAATAACGGATGTTTCAAGAACATTGTTCTATAAACGTTCTCTCAACATTTTGAGAACATTCATCTAGGACGTTCCAAGAACATTGTTTCAAGAAAATTTTCTCTCAACATCACAAGAATGTACAATAACTAATTAATAACATTATGAGAACATTCCATGCACATTATTTTAATAACATTTTTCCCTAACATTTAGAAAACTTTTTAAAGAACATTAGTGAAAGTTGTGGGAACGTTCCCTGTTAGCTGGGTA

>scaffold_210000162-1

TATATTGCCAAACGTTTGTGGACACCTCACCATCAGATCCATATATGGTTCCTCTCCAAAGGGTTGCCACACATTTGGAAGTACACAGTTGTTTAGAATGTCTTTCTAGACAACTGTGTTGTAGCATATCATTATACCTTCACTGAAACTAAGAGACCCAAACAGTGTTCCAGCATGACAATGCCCCTGTGCACAAAGCCCCTGAGTTCCATTAAGACATGGTGTGTTATGATTGGAGAGGAAAAACTTGAGTGTTCTGCACAAAGCCCTGACCTTAACACCACTGAACACCTTTGGGCTAAACTGGAACACCGACTGCATCACAGACCTCGTTACCTGACGTTAGTGGCTGATCGCACTAATACTATTGTAGCTGAATGAACATGTATCTCACATTCAAGCTCCAAATTGTAGTGGAAAGCCTTACTAGAAGAGTGGAGCTTAATCTAACAGCAAAATGGGAGTAAATCTGGAATAAGATGTTTCAAATCACATATGTTTGTTTGTAATGGTCAGGTGTCCACAAACTTTTGGCAATATA

>scaffold_210000163-16

GCAGGGACGGCCCTTCCATTAGGCGGAATAGGCAAATGCTAGGGGCGCCGTTCAGTCACAGGGGTGCCGAAATCCAAATAAATGAATCTAATAACACTATTATTAAACATTTTTTATTAATATTAATTTACAAAATATTCTAATTATTTGAAATATTCCAAAAAAACAAAACCCCCCACAAAAAACCTAATTACATTGCATTGCTTAATAGTCATTCGACTCAGCATCCTGGTCTGACGTGTCAATCTTCCCCATCAACGCTGCACTCGGACTTACCTGATAGTGACAGATGGCTGGATTCCTCTTTCACAACACGAAAAATTAGCATTACAACCTGTTTAAAGCTGCTTACAGTGTCAAAAAGACCAAAGGAGAAAATAATAGGAAGAAAAATGGGAAAAAGACAGAGATAATGTGATGAACGGTTTTTGTAACTACATAGTAGTTGTTAGGCAGCACTGTTATGTTAATGTTCGCTAATTTGAAATATCTAATTACAGTTAAAGTCAGTTTTACCTATGTACTGTTCACATCAACTGTACTACACTCAGGATGTTAACTCTCGTATAATGTGATTTTGTAACATAAATTACAAAATCAAGAGGCACCAGGTAAAATCTTGCCTAGGGCAGCAAATTTGTCAGGGCCGGCCCTGC

>scaffold_210000165-2

ATACACTATATGGACAAAAGTATTGCGACACTTGACCATCACATCCATATGTGTTGTTTGAACATCCTATTCCAGATGTAGTGCTACTCTTACTACTATAATAACCTCCACGCTTAGATTGTGGACCGTGGCTGTCGAAATTTGCTAATTCAGCCACAATAGTATTAGTGAGATCAGACACTAATGTTGGGTGAGGAGGTCTGGGGTGCAGTCGGTGTTCCAGTTCATCCCAAATGTGTTCAGTGGGATTGAGCCAGCTCTGTGCAGGATCACCGAGTTGTTCCACTCTAACTATAACACATCATGTTTTCATGGAGCTCAGGCTTTTTGCACAGGGGCATTGTCATGCTGGAACACTGTTTGGGTCTCTTAGTTCCAGTAAAGGAATAATGTGATGCTACAACATACAAAGACTTTCTAGACAACTGTCTGCTTCCAAATTTGTGACAACAGTTGGGGAAAGACACACTTGTGGGTGTGCTGGTAAGGTGTCCACAAACTTTTGGCCATATAGTGTAT

>scaffold_210000165-6

AATTAATAATATCCTCCTGAGACCCAAGGAAAAAAGTGTCATCAATTATTTTTGTTTTGTGTGATTTCCTACTCCTTTCGGGTTTAAAAAACATTCTACAATTATAGTTTTAAATTTAATTGTGATTTTTAATTTTACAGCATGTCCAGTAGATCACAGGACCATTTTAGTTTAAAAATAAATTTCCATAAAACTTGCTTGTTCTTATGGCGACGATTTTGGATGCAGTGTAAGAACTAGTTTCTAGCCCAGCCAATCAAATGACATATCACTAGAAAGCCCTAGATGTCCTCTGTACAGTACATCAGAAATTGATAGAGTAGCTAAAAAAAACCCAAAAAAAACATTTATGAAAACAAAAGGTCCACTACAGGGGACACAAGTCAACGGGCGGGGTCTCAGGGGAATATATAATT

>scaffold_210000167-6

TAGGGCTGCACGATACTGTGAAAATATGCAATAATGTTGTTGTGTATTGCGATTATATTTCCTGCGATATAACATTTCCCTAGAGAAATGCTATTTTTATTTTAGCTGCGGGTGTTTTTCGGGCTGGCTCATCTTATCAAGTTTAGCTCGCTATTAAAGCTTCGTGGCTTGTTGATTGTCTTGCGTCAAAGCACGCGCACGCCTATCCCAACTAACATCCGCGCGCCAGATATTTTACTATCCTAAAACATTGACTACACATTACTATTTGAAGTATTAAATCTCAATATGCACATTTGTGGTATTTAGATAATTTCGTTATATTGTGCAGCCCTA

>scaffold_210000167-23

CAGGGGCGCAGCTAGCAATTTTGGGCCCTATGAAAGAATATGAGGTTGGGCCCCCTACTACACCCATTATGCACCAAACAAACAAGACAACCTAGCTTTCCAAAAAATGTTAGTCTGCAATTTAATAATACTGACCTATTACTTTTGCTTTTGACCACTAGATATCAGTATTTAGTCATTTTTAGTATTTACTACTAATAAAACAGATCTCTGGGGGCCCCCAACGTGCGTGGGCCCTTAGAATCATCTTAACTTTCCCCCCCTTAGCGGCGCCCCTG

>scaffold_210000168-6

TTAGGGCTGTGTAAAAATATCGATATAGCTAACTATCGCGATATTTTCTAACCTCTAGTATCGACTTATTCAAATTATGTTTTAAACTGTTTACGTTTACTTTAATATTTAATTTAAACGCTCAGTTTTTATTTGAAGAATAACTAGAATACTAGAATCCTCCAGGTGGTACACAGCAAGTTGTATTTGTAGCTATTTGTTTGACTTTTTTAATAAGTATTGCAATATGTATTGTATTGCAATATATTGTGATATATTGTATCGTGACCCATGTATCGTGATATGTATTGTATCGTGAGGCCCTTGCCAATACACAGCCCTAA

>scaffold_210000172-4

TAAGGCTACGTTCACACTGCAGGCAAATGTGGCCCAAATCGGATTTTTTTTTCCTCAGATCTGTTTTTCTCATGACAGTGTGAACAGCACAAACCGCATGGAATCTGATCTTTTCAATTCTGATTTGTGCCACTTCCATATGTGGTAATAAATCCGACACAGGTCAGATGTTTTGCAATGCGACCGCAGTGTGAACAGTTATATCGGAATTCATGCGACTTTTACGTCATTTTCGATCGACATGCATCATCATTCTGCACTGGCGCATACTACAAACAGTTGATATATATATATATATATATATATATATAATCAATTTCGATGAAGGCGTTCACATCACGGTCCCACCACTCCTGGCTCCGGCGCCGCATCCACACACACACCTCCATACTGAAGTAGCAGCCACTGCTCCAGAACATGCTACTATTAAAAATGTGGCCCTTTTTCTCCTCATCCTCTTCCTCCTCAACAACTGCTCATTAATTCGCTGTCGTCCGCTGCACATCACCTTATAAACGTAAGAACCTAACGCGTAAATGCTGCCTTCAGTGTCTTCCATGTTTATTTTTCTGTATGACAGGACGCTGCATGAGACGTAGAATTTGTTCTTTTGCGCAAGCGGTTCAGTTTAGGACCATGATCAGATCACACTGGAAATCTGATATTGGCCACATTTAAAACAACAATGTAAACAGCTATACAAAAAAAATATGATCTGAGCAAAAATCTGAATTTTGCACAAAGCCTCGCAGTGTGAACGTAGTCTTA

>scaffold_210000173-2

CAGGGCTCGCAAAATGTTTTAATTCCTGGTAGCCCTTCAAGCAGGTTCTCTTCAGTTTTTGGTAGCCCGAAATTAATTTAAGTAGCCCGAATAAAAAAGACATTATTTTTAATGTAGAATATTGTAAAAGAGACAAAACTTCAATCAAAAACACATTTTTTAATACAAAATAATAAAATTACAATCATCAATACAAATATTTAGTTCTAACTGAACATCCTATTCCAGATGTCAGCGATTTTCGCAATTCCCTCCACTGCACATGCGATCTCAATCACATGTTTAATCACATGAGATTTCACTTGTCACGTGAGGATAGTAACGACTGACAGACTGATGTCAGAGGAATTGGTGCACAACAGATCGGGAGCGTTACTGACAAATATCTTGTAAAATCAGTACTGCCGCTCCCTATACACAGTTTGCACGATCGCGAAAGTAAAAAGTGAGCGAGCATTCAAACGCGATTTCATTACCACACATATTGATAGCGGCGCCCCGATGCCCGAAAATTAGAGACAACAATTACCCAACTATATTTGTGAGAGAATGCAAAAGCATTGAAATATTTTTTTCCTCCCTATGAGATAGCCCGACGGGCAGGGCAAGGATACATTTTGGTAGCCCGACTGAAAGTGACAATAGCCCCGGGACGTCGGGCTAGAGATTTTGCGAGCCCTG

>scaffold_210000175-2

TAGGGATGCACCAATACCATTTTTTAAAGATAGTTGTACAAGTATTTTTTTCCTGGTACTCGCCGATACCGATACCTGTACTTTTTGGGGATGTGGGATTAATTGTATGGCTCTCGGATTATCTCTTGCCATTTTCTCTCGTCTTGCAAGGGTTTGCTGCAGGGTTGGTTGCCGAGTGCTAACGTTACTGCTAGCGGCAAATTCTCTGTGTTTTAATTTTAGATGTTTTATCAGATTACTCGTATTACAAGTACTCATTTTTGTACCTCTTGATATTTTTGCAGCACAAAGTTTGCAGTTTGCCATGCGTGGGTTGTCGTCATTAATTTTGAAATATTTCCACACCGCTAAGCCTGACATTCGGTCACTGCTGCCGGAGTCTTTGTGCACTGAATGTTCTGTCAATGATGTCACAAAAGGTATAAGTCTTTGGTATCTGAGCGTTTTTACGAGCACGAGTACACGAGCTCAGTATTAGGTATTGCATACCGATACCAGTATCGGTGCATCCCTA

>scaffold_210000175-6

TAGGGCTGCACAATATATCGAAATTATCATAATATCTTAAATGTGCATATCAAGATAGGCATATGCAATGGTTTGCGATAAATAAGCGATTTAATATTTCAAATAGTAATGTGTGGACAACGTTTTAGGATAGTAAAATATCTGGCACGCAGATGTTAGTTGGGATGTGCGGGCGCGTGCTTTGACGCAAGACAATAAACAAGCTGCGAAGCTTTCATAGCGAGCTAAACTTTATGAGCTCGAAAAACACCTGCAGCTAAAATAGCTAATAAAAATAGCATTTCTCTAGGGAAATGTTATATCGCAAGAAATATCGTTATCGCAATACTCAACAACAATATTGCATATTTTCCCAGTATCGTGCAGCCCTA

>scaffold_210000178-1

ATTAGGGCTGCACAATATAACGAAATTATCGAAATATCGCAAATGTGCATATCGAGATATGCATATCGCAGTGGTTTGCGATAAATGAGCGATTTAGAACTTTAAATAGTAATGTATGGTCAAAGTTTTAGGATAGTAAAATATCTTACACGCGGATGTTAGTTGGGATGTGCGTGCGCGTGTTTTGATGCAAGACAATCAACAATCTGCAAAGATTTCATAGCGAGCTAAACTTGATAAGATGAACCAGCCCGAAAAACATCTGCAGCTAAAATAGATAATAAAAATAGCATTTCTCTAGGGAAATATATCGCAAGACATATCGTTATTGCAATACTCAACAACAATATCGCGTATCGCATATTTTCCCTGTATCGTGCAGCCCTAAT

>scaffold_210000184-1

GCTGCGTCCGAAATTGCATACTTCCATACTATATAGTATGTGAAACTGAGTATGCAAGGCGAGTAATATGTCCAAATCCTTAGTATGCGAAATACAGTATGCAAAAAGTTCCCGGATGACCTACTACTCCCGCCCGGATTTTGAAGTATGAATACGATATACACTTTAAAATCCCATGAGGCCACGGGAGAGACGACTCGTCATATGCGGAAGTGGCGGAAAGCGGCGACGCGCCACTTTACAAGTGCTTTAAGTAGCTTTAAGTGTAAAAAATTTTTTATAGTTCCTTGTGGTTAAATTGCACTTGATTAACATCATTCAAGTTAAACATTATCCAACTTTAAATTCGCGCCTCTGTGACGTCGTATGTCACGTGACAATATCAACATGGCGGATGTAGTACGTCAGAAATTCATTCATACTACCCGTAACCATACTATATAGAACATACTTTTTTAAACGGTCGGGAAGTGCGTACTTACTCAAATGTACATACTCAAGTAGTATGCAAATTTCGGACGCAGC

>scaffold_210000184-2

ATACACTATGTGGCCAAAAGTTTGTGGACACCTTACCAGCACGCCCATAAGTGTTCCTTCTTCAAATTGTTGCCACAAAATCTGAGTCACACTGTTGTTTAGAATGTATTTGTATGTCTTAGCATTCTATCATTTCTTCACTGGAACTAAGAGACTCAAACATTGTTCCAGCATGACAAAACCCCTGTGCACAAAACCCCTGATCTCCATGAAGACATGGTGTGTTCTGGTTGGAGTGGAAGAACTCGAGTGTCCTGTACCGAACCCTGACTCTGACTCAACCCCACTGAACACCTTTGGGATGAACTGGAACACAGACTGCACCCCATACCTCCTCACCCAACATTAGTGTCTGAACTCACTAATACTACAGTGACTAAATGAACACAAATCCCCACAGACACACGCCACAATCTAGTGGAAAGTTTATCCAGAAATGTGGAGGTTATTATAGGAGGAAGAGCGAGACTACATCTGAAATAGGATGTTCAAAAAACATGTATAGATGAGATGATCGGGTGTCCTAATACTTTTGGCAATATAGTGTAT

>scaffold_210000184-14

TAGGGCTGCACAATATATTGAAATTATTGAAATATCGCAAATGTGCATATCGAGATAAATGAGCGATTTAATACTTCAAATAGTAATGTGTGGTCAAAGTTTTAGGATAGTAAAATATCTGTCACGCGGATGTTAGTTGTTAGGGGTGCGCGTGCTTTGACGCAAGCTTTCATAACGAGCTAATCTTGATCAAATGAACCAGCCCGAAAAACACCTGCAGCTAAAATAGCTAATAAAATTAGCATTTCTCTAGGGAAATGTTACATCGCAAGAAATATCGTTATCGCAATACTCAACAAACAATATCGCATATTTTCCCAGTATCGTGCAGCCCTA

>scaffold_210000187-6

TTAGGGCTGTGTATTGGCAAGGGCCTCACGATACGATACATATTACGATACATGGGTCACAATACAATATATCACAATATATTGCAATACAATACATATTGAAATACTTAATAAATCCTTTTTTTAAAAGAAAAACAAATAATTAAAAATACAACTCGCTGTGTACCACCTGGGGGAGTCTAGTATGTGTATCACATTATATTGATTACTCAGCAGAAAAATAAAAACTGAGAGTTTTAATTGAATATTACGTCATTTATAAATCAGTATGACGTTTTAAATATCACTGTTTAAAACGTAATTGAAATATGTATCGATGCTACAGGTTAGAATATCGATACACTATCGTGAAAAAAAAAAAGCGATAATTAACTGTATCAATATTTTTACACAGCCCTAA

>scaffold_210000189-6

CAGGGGTGTCAAACATATGGCCCGCGGGCCAAATCTGGCCTGCTAGGGGGTTCAATCCGGCCCGCTGGATGAATTTTGAAAGTATAAAAAATGCATAAAAGACACAAACTCAAAATTCTTAATAAAATGCTATTCCTAAATCATCCGCTAGAGTCGCATTGTGTTAGTCAGAGTAGAGGGGTGCATTGTTTTAATTAGAGTAGAAGGAGAGGCACGACGAACAGCAGATGGTAGCAATGCTCCTTTTGCTAATTACGGTGTATGCTACTTTATTTTCTACTCACCTTCACACCCTCATTAGCCAAAATGTATTTGTCAAAAAGGAGAGAAGTGGATACCGAGTGTAGAGTTTTCCAAAAGAAATGGACTGCTTCCTATTCATTCACAGAGATGAATGGGAAACCTGTGTGCTTGGTGTGTAATATAATTGCTAACGAAATAGCATTAGCATCAAAGCCGTACACGAAAACAAAGGGAAAAAACATGGACTTGTTATTTATAGGTAATTATGTTAAGATTGTACAGGCTGTACTCTAATTAGGCTGTATGTGGCCCCTGAACCAAAATGAGATTGACACCCCTG

>scaffold_210000191-10

AAGGGCTGTACAATACTGGGAAAAAATACGATATTGTTGTTGAGTATTGCGATATCAATATTTCTTGCGATATAACATTTCCCTAGAGAAATGCTATTTTTATTAGCTATTTTAGCTGCGGGTGTTTTTCAGGCTGGCTCGAGTTAGCAAGATTAGCTCGCTAGGAAAGCTTCGCAGCTTGTTGATTGTCTTGCATCAAAGCACGCGCACGCATATCCCAACTAACATCCGCCTGCCAGATATTTTACTATCCTAAAACTTTGACCACACATTACTATTTGAAGTATTAAATCGCTCATTTATCGCAAACCATTGCGATGTTCACATTTGTGATATTTAGATAATTTCGATATATATTGTGCAGCCCTT

>scaffold_210000193-3

AGGCCGCATTTACACTGCAGGTCTTGATGCACGATTCCGATTTTTTGGACGACCCGCTTACATCTTATTTTAAAACTGACCCATATCCGATATCTGCATTTACACACGGCAAAACAACCCATGCGGTAGACGTACTGACCGGAAAAGGAAGTAAAAACGGCGCGATAATCAACGGACGAAGACGAAATGATTATTCAGTATTTTGCTTTACGCACCATATTTAAAGGTCGTCATAGTTGTTGGAACCTGGGGTCGGGAACAGTGGGTCAGCAATTTTCATATGAGACGAGAAACATTTGATATGTTGTCAAGAGGTCGCTGTTGGTGTCCACCTGAATTGACGTCATGGGGCACCGTCGCTTATTCAATGACGAACGACTCGCATTGACAGGTGAAAAATCCGATCTGTCTGCTTACATGTCAGATGCTAATGCACGTATCCGATTCATATCCGATTTATTTTCACATATGAATGAGGCCTGAAACCGATTTGAAAATCTCGGAATCAATGTGCATTTTTCCTGCTTACACGATCATATCATATCATATCGGATCTGTGCCAGATTGGAAGAACAAAATCGGAATTGGGTCACTTGATACATGCAGTGAAAATGAGGCCT

>scaffold_210000195-2

TACAGGGTGGGTGAAAATTAACTAGGCAATATTTAATGGCTATAAAACTTGTGGTATCACTAGAGTCATGATGAAAACAGTCTCTAAATAAACGATAGTAAAAAGGATTTCAAGCCTCGCACACTGGAAGACTTGGAGGCACGGATTCGTGAGGTTCTCAGCAATATCCCAAACGACTTCCTTCAGAAGACTCTCCGGCCGTTTGAGGAAACTGGTTGACACCACCGGTGCCTACGTTGAAATGTAAAGATTTGCTTTCATTTTCCTATGTGATGAAGTACATAATAAATTTGTATTAGAAATATGGACTTTATAACCAATTTTTAATGCCTAGGTACTTTTCCCCCACCCTGTA

>scaffold_210000198-3

TATATGGACAAAAGTTTGTGGACACTTTATCAGCACACTCATATAACTGTGCCTTCCCAAAATGTTGACACAAAGTTAAAAGCATACAGTTGTATAGAATATCTTTGTATGTTATAGAATTACATTATCCCTTTACAGAAACTAAGAGACCAAAACCTGTTCCAGCATGACAATGCCCCTATGCACAAAGCCCCAGAGCTCCATGAAAACATGGTTTGCCAATGTTAGCATGGAAAATTTAAGTGTTTTGCACAGAATCCTGATCTCAACCCCACTAAACACCTTTAGAATAAACTACAACGCTGACTGCGCCCCACTCCTCATCACCCAAAATCTGAGTCTGATTTCACTAGTACTGTTGCATCTATATGAGAAAATCCCCACAGCCTTTTTAAACAATCTAGTAGAAAGCCTTTTCAGAAGTCTGGAGGTTATTATAGGAGTAAAAGAAAGACTAAATCTGGAATAGGATGTTAAACAAACACATATGAATGTGATGGTCAGGTGTTCCCATACTTTTGTTCATATA

>scaffold_210000200-1

AAGCCAGGCATACACTGCGATTTTTAATAGTCAATTGTTACTTGTCAGACTGTAGGAACATGATCCCCATGTCACACTGTAGGGTCTCAGTTGTCATAATGTCAGACTGTATGACAGTAAAGATGCGTCAAAACCAGATGCACACAAGAAGACTCATCCGGAGTTTTACGTCAGCAATCCGTGACACGTTCAGTAAATGACAATCCGTGACACGTTCACTCGCTAAATGGTAGCTAGCAGCAAACAAACAGCAGCGACGACACGTCCTACAAACCTTCAATAAGTTTCTCTCTTTGCTGTACTGTCCACCTGACTCGTTTCCACATTTTCCCCAAATGGTTTGTAGTTGTTGCGCTAATTGCGTCATTAGGTTCAGCGCTTCTTGGCTTGACAGTTGTCGTAGGAGAAGTCACACTGCAGGAAAATGTCTGAAATCTTCTGACACTGCCAGAATTTCATCGGAGGAAAAAAATGATCTCAATTACTATTATTTGGCTGTCGGTGAACATGTCAATATAGTGATCAAAGACAACAGACTTTAGGCGTAGGATTATAGGTATCTTTTAGGATTACAAAATTTGTCTTGTGTCCAATCGTGGCCAAAATCGCACAGTGTAAGCCTGGCTT

>scaffold_210000200-11

ATATTAGAGATGCACCAATAAAAAAAATTTAAGACCGAGTACGAGTACCGATATTTTTTTCCTGGTACTTGTCAATACAGATACCTGTACTTTTGGGATGTGGGATTATTTGTATGGTTCTCGGGTTAACTCTTGTGATTTTCTCTCGTCTTGCAAGAGTTTGCTGCAGGGTTGGTTGCCAAGTGATAACATTACTGCTAGCGGCTCACTTTTGTGTTTAAATTGCAGTTGTTTTATCAGATTACTTGTATTACAAGTACTTATTTTTGTACCTCTTGAAATTTTGGGGGAACAAAGTTTGCAGTCTGCCATGCACGGGTTGTCGTCATTCATTTAGAAATATTTCCACACCGCTGAGCCTGACATTCTGTCGCTGCTGCCAGAGTCTTTGTGCAGTGAAAGTTCTGTCAATGACGTCACGTCAAGGGGTATCGGTCTTTGGTATCAGAGTGTTTTTACGAGTACAAGTGCGAGTACTTGAGCTCGGTATCGGTGCATCCCTATTAT

>scaffold_210000213-1

ATCAGGGCTGGGTAACCCATGTTCCTTAAGTTACTTTATTCTGCTTGTTTTCCAGCTACCTGCCCTACCCACTGCTGATTACCTGCTTCAGGTGTGTTCAGTCAATCAGAAGCTGGAAGATACCATTTCAGATGAGGGCGGAGTGGGGAAGCACCTGGTCATTTGGTATCTCCTACCCTCTTGATTGAATGAACACACCTGAAGCAGGTAATCAGCAGTGGGTAGGGCAGGGGTAGCTGAAAAACAAGCGGAACAAAGGAACTTAAGGAACATGGGTTACCCAACTCTGAT

>scaffold_210000214-2

TAGGGCTGCACAATACTGGGAAAATATGCGACATTGTTGTTGAGTATTGCGATAACGATATTTCTTGCAATATAACATTTCCCTAGAGAAATGCTATTTTTTATTAACTATTTTAGCTGTGGGTGTTTTTCAGGCTGGCTCATCTAGTCAAGATTAGTTTGCTACGAAAGCTTCACAGCTTGTTGATTGTCTTGTGTCAAAGCACGCGCACGCATATCCCAACTAACATCCGCATGCCAGATATTTTACTATCCTAAAACTTTGACCACACATTACTATTTGAAGTATTAAATCGCTCATTTATCACAAACCATTGCGATATGCATATCTCAATATACACATTTGCGATATTTCGATATATTGTGCAGCCCTA

>scaffold_210000219-3

ATGCCGAGTTCACACTGCGCGATTTTCAGGTCGTCGGATCACCGTTGTTTTCCCACTGCAAGACTATCTGGGGTAACATTCAGTTGCTGTTGTGTTCACATTGCACGATAGCTCGGCGACAGGAGGTTACACACTGCATGACTTTACAATAGGAAGAATCGCAGATAACTCTGTCTGCTCCGCAATCTAAGTTTCACAACTAAACGCACGCGAGAAGTGATAGGAAATAAGACGTGAGATCAGAGTTCTCGCACGAGACTGGAAATGTTATTAAAATGGGAGCCCGCAAGAAGTTTGTGATTCAAATGGTCTGCGCACTGATTTACAGTGAAAGAGTGAAAAAGAAAAAAAAGGATGGGAACCGTAGCCATGCTAGCTGATATTATAGTCTCTTACTCCTCCCTGAACTTCCCGCTGTCCTGTATCTTGCTCTCTCATTGGCTGTAGGTCAGCGCCGATGAATTTTTAATCAGAACTCATTTCACACAGCAGGAGTTTGAATCGCAGACAAGCTCAGATATTTAGAATGGCAAATATTTCATGGGGCTTGGCGACGCCTCTGCGATTCTCTCTGATCGCGTCTTTGATAATTCACACTGCGCGATTGTCGCTCGCGTGAACGAGCAGCGATTTGCTTCTGATTTCGAGCATTGCTCGGCGATTCCGAAAAACCTGTCGGCGAGTGAAAAATCGTGCATTGTGAACTCGGCAT

>scaffold_210000219-7

TAGGGATGCATTGATACCGATACTGAGCTCATGTGCTCGTACTCATAAAAACACTTCGATACCAAAGGCCGATACCTCTTGTGACGTCATTGACCGAGCTTTCAGTGCACAAAGTCTCCAGGGGCAGTGACAGAATGTCAGGCTCAGCAATGTGGAAATAATTCAAAATTAATGATGGCAGACTGCAAACTTTGTTCTGCAAAAATATCAAGAGGTACAAAAATGAGTACTTATTATACGAGTAATCTGATAAAACATCTGAAATGAAAACACAAAAATGAGCACGGAGCATTTGCCACTAGCAGTAACGTTAGCACTTGGCAACCAACCCTGTAGCAAACTCTTGCAAGACGAGAGAAAATGGCAAGAGATAACTCGAGAGCCATACAAATAACGCCACATCCCAAAAAGGTACAGGTATCGGGATCGGCGAGTACCAGGAGAAAAAATATCGGTACTCGTACTTGGTCTTTAAATAATGGTATCGGTGCATCCCTA

>scaffold_210000224-2

AAATACACTATATAGACAAAAGTATTGGGACAACGAATCATTACATCCATATATGTTTTTTGAACATCCTATTCCAGATTTAGTCTCAATCGTACTCCTATAATCACTTCCCCACTTCTGGAAAGACTTTCCACCAGATTGTGAAGTGTGAAGGGAAAATATTTCATTCAGCCACAACAGAATTAGTGAGTTTAGGCGCTAATGTTGGGTGAGGAGTTCTGGGGTGCAGTCGGTGTTCCAGTTCATTCCAAAGGTGTTGGGTGGGGTTGAGTCAGAGTCAGGGCTCTGTGCAGGATGCTTGAGTTCCTCCAGCCATAACACACCATATCTTCCTGGAGTTTAGGTCATTTGTGCACAGCGGCATTGTCATGCTGGAACGGTGTTTGGGTCTTTTAGTTCCAGTGAAGGAATAACGTAATGCTACAACATACAAAGACATTCTAGACAACTGTGTGCATCCGATTTTGTAGCAACAGTTTGGGGAAGACACACTTGGGTGTGCTGGTAAGGTGTTCACAAACGTTTGGCCATATAGTGTATTT

>scaffold_210000224-8

TAGGGCTGTGTAAAAATATTGATACAGCTAACTATCATAATTATTTTTTTCACGATAAAGTATCGATATTCTAACCTCTAGTATCGATACATATTTAAATGACGTTTTAAACTGATTTAAGTTTGCGTTAACATTTAATTAATATCCGCAGTATTTATTTGTCTGCTGAGTAATCAATATAACGTGATACACATACTGGAATCCCACAGGTACTACACAGCAAGATGTATTTTTAGCTATTTGTTGTACATTTTTTATTATAATTTTTATAATGTATATATTTAAAAATACACGTATTGTATTGCAATATATCGTGATATGTTGTATCGTGACCCATGTATCGTGATATGTATCGTATCGTGAGGCCCTTGCCAATACACAGCCCTA

>scaffold_210000227-3

ATACACTATATCGCCAAAAGTTGTGGACACCTGACAATTGGAATATGTCATTTTAAACATCTTATTTCAGATTTACTCCCCATTTTGCTGTTCGATTAAGATCCACTCTTCTAGTAAAACTTTCCACTACAATTTGGAGCTTGAATGTGAGATATGTGTTCATTCAGCTACAATAGTATTAGTGAGATCAGGCACTAACGTCAAGTAAGGAGATCTGTGGTGCAGCCGGTGTTCCAGTTCATCCCAAAGGTGTTCAGTGGTGTTAATGTCAGGACAAGTTTTTCCACTCCAATCATAACACACCATGTCTTAATGGAACTCAGGGGTTTTGTGCACAGTGACATTGTCATGCTGGAACGGTGTTTGGGTCTCTTAGTTCCAGTGAAGGAATAATGTAATGCAACAACATACAAAGACGTTCTAGACAACTGTGTGCTTCCAAATTTGTAGCAACATTTTGGAGAGAAACCACATATGATTGTGATGGTCAGGTGTCCATAAACTTTTGACAATATAGTGTAT

>scaffold_210000227-4

ATATACAGCTCTGGAAAAAAATTGAGACCACTCCAATTTAAATTTTTTTTAAAAATCAGCATTTCTACATGTATGACAGCTTTTCCATTCCTGTGTTTTTGAATTCTGATATTGGCCCATCAGATTCTCCAGTACTGATTCTGTGATCATCTGAAATATTGTCAGCAGTTTAAAAAATAAAACAAAACTGTCCATTTTACTTAAACACACCTATAAATAGTAAAACCAGCGAAATAGATCAGTTTAAGTGGTCTCTTAATTTTTTCCAGCGCTGTATAT

>scaffold_210000228-8

TAGGGCTGTGTAAAAATATTGATACAGCTATCGCAATATGTTTTTTAAATGATATGTATCCATATTCTAACCTCTAGTTTCGATACATAATTAAATTACGTTTTAAACTGATTTACATTTATGTTAATATTTCATTAAAACCCTGTGTTTATTTGTCTGCTGAGTAATCAATATAATGTGATACATATATACTAGAATCATCCAGGTGGTACACAGCAAGTTGTATTTTTTAGCTATTTGTTTTATATTTTTAAATATATATTTTTTGAAGTATTGCAATATGTATTGTGTTGCAATATATTGTGATATATTGCATGGTGACCCATGTATCCTGATATGTATCGTATCGTGAGGCCCTTGCCCATACACAGCCCTA

>scaffold_210000229-2

AATTAGGGCTGTGTAAAAACATTGATACAGCTAACTATCGCAATATTTTTTTTTCACGATAGTGTATCGATATTCTAACCTCGAGTATCGATAAATTAGGTTTTAAACTGATTTATGTTTACGTTAATATTTAATTAAAACCCTCAGTTTTTATTTGTCCGTGGAGTAATAAATTCACATACTAGAATCCCCCAGGTGGGTCACAGCAAGTTGTATTTTTAGCCATTTGTTTTACATTTAAAATATATATTTTTTAAATATGTATTGTATTGCAATAAATCATGATATATTGTATCATGACCCATGTGAGGCCCTTGCCAATACACAGCCCTAATT

>scaffold_210000230-2

CTACGGTGGCCGAGAGAGCTCAATTGTTCATGCTGCAATTTAAGAACCCACATGCAAATAGAAAAAACACCAACAAATTAAGAAAACATCTTCATTAGTTTGACAACACACGTCCTGCAAATTCTCACAACACGACCAAATACAGAAATACGCTGCAAAAACAGAAATGCACTGCAACTAGCACAGACCACAAGGGGACCCAAAAAAGTGACTAACTCGGCTGGGACCTGTTTATCGTTTCACAGTTAATTGTGCAATTTGTCAGATTAATTTCAGGCTAATAACATTTAAAAGACCAAGGAATCATACGATCGGGATGATAAAATGAACAAAAAACTTGCCTTTCAGTTTACCGATAACACCACTGACAAATAGCCACTGAACAATAAGCAGGTCCCTGCCAGGTTCCTCACTTTTTGGGGTCCCTTGAAACATTTCCGTTGTGGTGTTGCAGCGCGTTTCCATATTCGTTGGCATTGTGAGGATTTGCAACACGTCTGTTGTCAAACTGATGAGGATGTTTTCTTCATTTGTTGGTGTTTTTTTCTATTTGCCTGTATTTTCTTAAGTTGCAGCGCGTTGAACTCTCTTGGCCACCGTAG

>scaffold_210000230-8

CTAAAGTCGGGTTTACAATGTGTGATTTGGGCCGCAATTTGGTAGTTTGAGACAAATTATGAAATCCTAAAGGCTTCTTATAATCCTATGCTAAAATCTGTTGTCTTTGATGGCTAGTTTGACATGTTCACAGCTGATTAATGGCTGTTGCAATCAGTTTTTTCCTCCGATGAAATTCTGGCAGTGTCAGAAGTTTTCAGTCTCTTCCCTGCAGTGTCACTTCTCCTACGACGACCGTCAAACCAAGAACCAATAGGGTGCGCCGAACCTGATGATGCAATTAACATGACCACTTCAAACCATTTTGGGAAAATGTCGAAACGAGTCAGGTGGACAATACAGCAAGAAGAGGAAGTTATTAAGTTATGTAGGGAAAAGGAGTGTTTCTATGACGTGACGTCGCTGCTGTTTGTTTGCTGCTAGCTACCACTTAGCGAGATAACGCGGGTTACTGAATGTGCTATGAATTGATGACGTAAAACTCCGGACGAGTCTTATTGTGTACATCCTTTTTTGATGCTCTTTGACTGTTGTACAGTCTGACATTATGACAACTGAGATCCTACGGTGTGACATGAGGATCATGTTTGTACAGTCTGACAAGCAACAGACTATTGCAAAAGACTATTAAAAATCGCACAGTGTAAACTTGGCTTTAG

>scaffold_210000231-3

TTTACACTGCAGGTCTTGATGTCCAATTCCGATTTTGTGACTATATCCGATTTTCTTGATGACCCGTTTACATCTTCTTTTAAAACTGACCTGTATCCCATATCTGCATTTACACTATACATGGCAAAAGAACCCAAGGTAGATGTACTGACCGGAAAAGGAAGTAAAAACGATGCGGTATAATCAATGGACGCAATGGATGAAGACGAAATGATTATTCAGTATTTTGCTTTTTCCGCACCATATTTAAAGGTCGACAAAACAATGCTCTCTTTAGGATGAAAAAAAAGATGAGAGCCCTTATTGCAGCCTGGGTGAATGTAGCATTGATCGCTGCTACATCGGTTCAGAGAGACGTGTGGGTGCGGCAAAGGACTCAAGATTGGTGAGACCAAGATCTTGGAACCTGGGGTCAGGAACAGTGGGTCAGCAATTTTTGTATGAGACGAGAAACATTTGATATGTTGTCAAGAGGTCGTTGTTGGTGTCCACCTGAGTTGACGTCATGTGCCACCGTCGCTTATTCAATGACGTATGACTCGAATTGACAGGTGAAAATCCGATCTGTCTACATGGCAGACGCTAATGCATATCCGATTTATTTCCACATATGAATGAGGCCTGAAACCGATCTGAAGGTGCTTTTTTTCCTGCTTACATGATCGTGGGTCATATCCGATCTGTGCCACAATGGAGGAAAAAATCGGAATTGGGTCACTTGATACATGCAGTGTAAA

>scaffold_210000231-4

CATTAGGCTCTGTCCGAAATCGCATACTTCCATACTATATATTATGCGAAACTGAGTATGCGAAGCGAGTAGTATGTCCGAATCCTTAGTATGAGAAATACAGTACGCGAAAATTTCCCGGATGACATACTACTCCCGCCCGCATACGGATGTACACTTTACTATCCCATGAGGCCACAGGAGAGACGACTCGATCTATGAGGAAGTGGCAGAAAGCGCCGACGCAGCACTTTTCAAGTGCTTTAAGAAGCTTTAGGTACTTAAAGAAAAAAACATTGTTCCTTGTAGTTAAATTGCCTCGTTTAACATCATCCAAGTTAAACATTATCTAACTTTAAATTCGCCCTGCTGTGACGTCGTAAGTCAAGTGTCAGTATCAGCATGGCAAATGTAGTACGTCCGAATGTCATTCATTCTAGCCGTAACCATACTATATAGAACATCCTTTTTTAACGGTCGGGAAGTACGTACTTACTTTATTGTGGTACATACTTAAGTAGTATGCGATTTTGGACGGTGCCTTAATG

>scaffold_210000232-11

TATAGATAGGGCTGTGTAAAAATATCGATACTATCGCAATATTTTTTGTTCATGATTGTGTATCGATATTTTAACCTCTAGTATCGAAACATATTTAAATGACATTTTAAACTGATTACGATTACGTTTACGTTATTATTGAATTAAAACCCTCAGTTTTTATTTGTCTGCTGAGTAATCAATAGAATGTGATACACATACGAGAATCATCCAGGTGGTACACAGCAAGTTGTATTTTTAGCTATTTGTTTAACTTAAAAAAAAAAATTGAAGTACTGCAATGTATTGCAATATGTATTGTATTGCAATATATCGGGAAATATTGTATCATGACCCATGTATCGTGATATGTATCGTATCGTGAGGCCCTTGCCAATACACAGCTCTAGCTATA

>scaffold_210000233-1

TATTGCCAAAAGTTTGTGGACACCTGACCATCACAAACATATGTGATTTTAAACATCTTATTCCAGATTGTAAGGTTTTCCACTACAATTTGGAGCTTGTATGTGAGATATGTGTTCATATGTGTTCAAGTGCTTGATCGTCAGGTAAGGAGGTCTGTGGTTCAGTCGGTGTTCCAGTTCATCCCAAAGGTGTTCAGTGGTGTTAAGATCAGGGCTTTGTGCAGAACACTCAGCTTTTTCCCCTCCAATCATAACACACCATGTCCTAATGGATCTCAGGGGCTTTGTGCACAGGGGCATTGTCATGCTGGAACAGGGTTTGGGTCTCTTAGTTCCGATGAAGGAATAATGTAATGCAACAACATGCAAAGACATTCTAGACAACTGTGTGCTTCCAAATGTGTGGAAACATTTTGGAGAGAAACCACATATGATTGTGATGGTCAGGTGTCCACAAACTTTTGGCAATA

>scaffold_210000233-8

TATTCTCCTGAGACCCAAAGAAAAAGGTGTCATCAATTTTATTTGTTCTTTGGGATTTCCTATTCCATTCAAGCTAAAAAACATTCTAAAATTTAGAGTTTTTAAATTTGATTGTTATTTTTAATTTTGCAGCATGTCCACTATAGTGGACCACAGGACCATTTTAGTTTGAAAAGACATTACTTTCCATAAAACATGTTTGTACCTATAAAAAAACGGCAGCCATTTTGGATGCAGTGTAAGAACTAGTTTCTAGCATCCCAGCTAATCAAATGACATATCACTGGAAAGCCCAGGATGTTCTCTGTACAGTACAGCAGAAGTTGACAGAGTAGCTAAAAAAATTCCAAAAACATTTATGAAAACAAAATGTCCACTACAGAGGTTAATGGGCGGGGTCTCAGGAGGATA

>scaffold_210000235-3

ATAACTATATTGTCAAAAGTTTGTGGACAGCTGACCATCACAATCATATGTGGTTCCTCTCCAAAATGTTGCTACACATTTGGAAGCACGCAGTTGTCTAGAATGTCTTTGTATGTTGTTGCATTACATTATTATTATTACATTATTCCTTCACTGGAACCAAGACACCCAAACCCTGTTCCAGCATGACAATACCCCCTGTGCACAAAGCCCCTGAGATCCATTAAGACATGACGTGTTCTGATTGGAGTGGAAAAAGCCCTGATCTTAACACCACTGAACACCTTTGGGATGAACTGGAACACCGACTGCACCACAGACCTCCTTACCTGACGTAACCTTACCTTACCTTGATCTCACTAATACTATTGCAGCTGAATGAACACATATCTCACATTCCAGCTCCAAATTGTAGTGGAAAGCCTTACTATAAAAGTGGAGCTTAATCGAACAGCAAAATGGCGAGTAAATCTGGAATAAGAAGTTTAAAATCACGTATGTTTGTGATGGTAATGTGTCCACAAACTTTTGGCAATAAAGTGTAT

>scaffold_210000235-6

ATACACTATATGGCCAAAAGTTTGTGGACACATAAGTGCGTCTTTACTAAACTGTTGCTACAAGGTTGAAAGCGCACATTTGTCTACAATGTCTTTGTATGTTGTAGCATTAGATTATTTCTTCACTGGAACTTAAAGGCCCAAACCCTGTTCCAGCATGACAATGCCCCTGTGCACAAAGCCCCTGAGCTCCATAAAGACATGGTGTGTTATGGCTGTGTGAAAGAACTCAAGTGTTCTCCACAGAGCCCTGACCTTAACCTCACTGAAGACCTTTGGGATGAACTGTAACTCAGACTGCACCCCAGACCTCCTCACCCAACATTAGTGCCTGATCTCACTAATACTATTGTAGCTGAATGAGAAAATCCCCCAAATCCACAAATCTAGTAAAAAGCCTTCCCAGAAGTGTGGAGGTTATAATAGGAGTATGATTGAGACTAAATCTGGAATAGGATGTTCAAAAAACACATAAGGATCTTATGGTCAGGTGTCTCAATACTTTTGTCCATATAGTGTAT

>scaffold_210000235-8

TTTAACCCTTGTGTGGTGTTTGGCTCTGTGGGACCCGATTTCAAAGCTAGTTTCAAGAGAAATGATGCTATTAATAATTTTTGTAGCCCCAGACTCATTGGCCTTGCCTCATGTTCCATGTGGAACATAAAATAGAACATATTTTATATAACACTTATATATTCAAATGTGATCTAACGGGGTAAATGGCAAATATTAACCATAAATGATGTCTATATTGCTTGTAAGTGAGCTAAAGTAAACATCTTTACTTTAAGTGTTTTTTATAAATTGTTATGGCTGTGTTGGTTAAAAACCCAATAATGTGGTGGGTCCACCAGACCCGTGAACACTGGCTAGTTAACAAAAACATGAACACCACATAGGGGTTAAA

>scaffold_210000236-11

CATTAGGGCTATGTATTGGCATGGGCCTCACGATGCGATACATATCATGATACATGGGTCACGATACCATTTATCACGATACATTGCAATACAAAACAAATAGCTAAAAATACAACTTGCTGTGTATCACCAGGGGATTCTAGTATGTGTATCACTTTATATTGATTACTCGGCAGACAAATAAACACTTGAGGGTTTTAATTAAACATTAACAAACGTAAATCAGTTTAAAATGTCATTTTAAATATGTATGGATACTAGCGGTTAGAATATCGACCCCCTATTGGGGGAAAAAAATATTGCGATAGTTGGCTGTATCGATATTTTTACACAGCCCTAATG

>scaffold_210000237-6

AGCTATGTTTCCATCCAAAGATGCGAATTAAACTTATGCGCAAAACTGGAATATCGCATAAAACATTTGCGAATAAAGCACCATTTCCATCCAACGAGTCAAAGAGAACAAAATCGTCACTTCCTGATAAACTGGCACCAAATATCAAAAATAAAAATGGAAGTTGCTGCGGTCGGAGAAGCCACCGTGGGACTTTTTTCGTATATAATAAATTACTTGCGTCTCGGAACACGTAGACGAAACGCAGTGAACACAGTCATGTCATCTTGCTTTCGAAGGCGGGAAAAGCTCTTTGGGAACGTAGCCGGTCAAGACAGTTCTGGGAGGTAATAATACCGAACCACTTTGATGACAGACTTTGGCCAAGGCATTTTAGAATGACCAAAACGACATTTCAGATGCTGTGCAATGAGATCGGTCCGCTGGTTAGTCCATAAATGCCGTCCCATCGCACCCCAGTTCCCACAGAAAAACGCATTGCCATTGCGCTTTATAAATTGGCCATTTTTGTTATCACATGATCTGTTGATGCAAAAATCACATGACTTTTTTTATGTGCATGCTGGAATTTATTCGGTAAACGTGTTTCCATTGTAGTTTATGCACATTTTTTCTTACCGGATAAAAAGTTTATCCTAACTCAGTTGTGCGGATACTTTTTTAATGCACATTTTCAGAATTTATGCGCATCTTGCCGTTTCCATCCAGCGATTTTTAATGCAACAGTCCAAAATGCGCATAAAAATAGGTGGATGGAAACATGGCT

>scaffold_210000237-7

AAAGGGGTCATGAACTACCTCATTTTATTTTTATTTTAGACTGTTCTCTGAGATCCACTTATAAAGTTATCAAGATTTTTACATAAAAAAAAACAGTCATAATTTAGAAGTAATAGGCTATTTTCTGTCCTGTTTTTGACCCCCTAACACCCCTTGTCAGAACGCTCCAGTTTGAATAGGAGTGGCAGACTGTTGTCTCGGAAGTAAACACCCACTGCTATGATTGGCTAACAGTTTTCTGCCGCTCTTGTCATTTACCCATCACGTGCATGCGCAAAATCGGTGGGCGGGGCTAGTGATAACAGACAGTGATTTCGAACCAGGCGTCAATCTTCTTCTGCAGAGGTGGCACTTATCCACACTATTACGTCATCGAGTAGAATATTCCAAAACCTGTCATTTTGGCCGACTGCCTTCAATATAAGCTGTGTTTAGAGTCACGACAAAGTTTTGAGTTCTGATACTTGCAGGATGTTTTTATAGCACAATGACCTCTTAAATGTCAAAAGATCAAGGGAATTTTAATTTCTCAGTTTATGACCCCTTT

>scaffold_210000237-11

CACTATATGGCCAAAGGTTTGTGCACACCTACCTAAACATCTCTTCCTTAAACTGTTCCCACAAAGCTGGAAGCGCACATTGTGGTCTACAATGTCTTTGTATGTTGTAGCATTACATTATTCCTTCACTGAAACGAAGAGGCCCAAACCCTGTTCCAGCATGACAATGCCCCTGTGCACAAAACCCCTGAGCTCCATAAAGACATGGTGTGTTATGGTTGGTGTGGAAGAACTTGAGTGTTCTACACAGAGCCCTGACCTTAACACCACTAAACACCTTTGGGATGACTACACCCCAGACCTACTTACCTAACATTAGCACCTGATCTTTCTAATCCTTTTGTGACTGAGTGAGCAAATCACCACAGCCATGCTGCACAAACTAAAATCGCTTTCCAGAAGTGTGGAGGATGTTACAAACGTAAGAATGAGACAATCTGAAATAGGATGTTCAAAAACACATACAGATGTGATGGTTAGGTGACCCAATACTTTTGCCCATATAGTG

>scaffold_210000238-1

TTACACAATATTGCCAAAAGTTTGTGGACACCTCACTATCATATCCATATGTGGTTCTTCTGTGGTTGCCACAAATTTGAAAGCACACAGTTGTCTAAAATGTCTTTGTATGTTGCTGCATTACATTATTATTACATTATTCCTTTATTGGAACTAAGACACCCAAACCCTGTTCCAGCATGACAATGCCCCTGTGCACAAAGCCCCTGAGATCCATTAAGACATGACGTGTTCTGATTGGAGTGGAAAAAGCCCTGATCTTAACACCACTGAACACCTTTGGGATGAACTGGAACACCGACTGCACCACAGACCTCCTTACCTGACGTAACCTTACCTTACCTTGATCTCACTAATACTATTGCAGCTGAATGAACACATATCTCACATTCCAGCTCCAAATTGTAGAGGAAAGCCTTACTATAAAAGTGGAGCTTAATCGAACAGCAAAATGGCGAGTAAATCTGGAATAAGAAGTTTAAAATCACGTATGTTTGTGATGGTAATGTGTCCACAAACTTTTGGCAATATAGTGTAA

>scaffold_210000238-4

ATTAGTAGGGCTGTGTATTGGCAAGGGCCTCACAATACGATACATATCATGATACATGGGTCACGATACAATATATCAGGATATATTGCAATACAATACATATTGCAATACATTGCAATACTTCAAGAAAAATCAAATTAAAAAAGGCCAATTGTTGTGTACCACCTGAGGGATTCTATTATGTGTTTCGAATTATATTGATTACTCAGCAGACAAATAAAAACGGAGGGTTTTAATTAAACATTAACATAAATGTAAATCAGTTTAAAACTTTTAAATATGTATCGATACTAGAGGTTAGAATATCGATACACTGTCATTAAAAAAAAAATCGCAAAAGTTAGCCTTATCAATATTTTTACACAGCCCTATTAAT

>scaffold_210000240-1

TACTGTTGCCGAGAGAGCTCAACGTGCTGCAATGAAACTTGTGCAAAAATAAAAAACACCAACAAATTAAGAAAACGTCTTTAGCAGTTTGACAACACACGTTCTGCAAATCCTCACAACAGAGACCAAATACAGAAACTCGTTGCAAATAGCGCAGACCACAACAGAAATGCTTCAGGGGGACCCCCCAAAAAGTGACGAACCTGGCTGGGACCTGTTTATCGTTTCACAGTTAAATTGTGTAATTCATCAGATTATTTAGGCTAATAATATCCAAAAGAACCAAGGAATCATACAATTGGGATGATAAAATAAACAAAAAAATTACCTTTCAGTTTAGATAACACCACTGACTAATAGCCGCTGAACAATAAGCAGGTCCCAGCTGGTTCGTCACCTTTTGGGGTCCCTTCAAACATGTCCTTTGTGGTCATTTCCGTATTCGTTTGCGTTGTAAGGATTTGCATCGTGTTTCTGTAGCGTGTTTCCGTATTGCGGATTGTGTTGTCAAACTGATGAAGATGTTTTTTTAATTTGTTTGTGTTTTTTCTATTTGCACCTGTTTTCTTATATTACAGCGCATTGAGCTCTCTAGGCCACCGTA

>scaffold_210000240-22

TAGGGATGCACCGATACCATTTTTTAAAGACCAAGTACGGGTACCAATATTTTTTCCCTGGTACTCGCTGATACCAATACCGGTATTTTGGGGGATGTGGGATTATTTGTATGGCTCTCTGGTTACTTCTTGGCATTTTCTCTCGTCTTCCAAGGGTTTGCTGCAGGGTTGTTGCTGAATGCTAACATTACTGCTAGCGGCAAATTCTCCAAGCTCACTTTTGTGTTTTAATTTCAGATGGTTTATCAGATTACTCGTATTATAAGTACACATTTTTGTACTTCTTGATATTTTTACGGAACAAAGTTTGCAGTCTGCTATGAGTCATCATTAATTTTGAAATATTTCCACACCGCTGAGGCTGACATTCTGTCGCTGCTGCCGGAGTCTTGTGCACTGAAAGTTCTGTCCATGACATCACAAGAGTTATCGGTCTTTGGTATCGAGGTGTTTTTACGAGTACGTGTACATGAGCTCAGTATTGTGCATCCCTA

>scaffold_210000242-1

TATTTAACCCTTGTATTGTCTTCCTGTCAACCATGCAACTTTTGTCCTCCTGAATTACTGTTTATAAAGCATGATATTAAAAAATTATCACCCAATGTTAATTTCAAGCAATTACATAGAAATTAAACCATATTTCTAATATCAAAGCTTTTAAAACGGGCCAAATTTCACTTGAAGACAACAGGAGGGTTAAATA

>scaffold_210000244-2

CTAAAGTCGGGCTTACACGGTGCGATTTTGGCCACAATTTGGTTGTCGGCGACTAATTTTGATATCCTAAAAGATTCCTATAATCCTATGCTAAAATCTGTTGTCTCTGATCGCTAGTTTGACATGTTCACTGACAGTTGATTAATGGCCGTTACGATCAGTTTTTTCCTCCGATTAAATTCTGGCAGTGTCAAAAGATTTCAGACACTTTCCTGCAGCGTGACCTCTCCTACCGATATGAGCGCCGAACCTCATGAAGCAATTAGCGTGACAACTTCAAACTACCTCGGGAAAACGTCAAAACGAGTCCAGTGGACAGAACAGCAAGAGATTAAACTTATTGAGAAAAATATCGCCTTAGATTGTTATTGTTTGCTGTTAGCTACCATTTAGCGAGAGAACGCAAGTCACTGAACGTGTCACGGATTGATGTAAAACTCCGGACGAGTCTTCTTGTGAGCGTCCGTTTTTGACGCGTCTTAACTGTCGTACAGTCTGACATTATGACAACTGAGATCCAACAGTGTGACATGGGGATCATATTCGTACAGCCTGACAAGCAACAATCGCAAAAAGGACTATTAAAAATCGCACAGTGTAATCCCGGCTTTAG

>scaffold_210000244-12

TAGGGCTGTGTAAAAATATCGATACAGATAACTATCGCAAATTTTTTTTCACGATAGTGTATTTTTAACTGATTTACGTTTACGTTACTATTTAATACAAACCCTCCGTTTTTATTTGTCTGCTGAGTAATCAATAGAATGTGATAGACATACTGGAATCCTCCAGGTGGTACACGGAAAGTTGTAGTTTTAGCTATTTGTTTTACTTAAAAAACATTTTTTTGAAGTATTGCAATGTATTGCAATATGTATTGTATTGCAATATAATGTGATATATTGTATCGTGACCCATGTATCGAGATATGTATTGTATCGTGAAGCCCTTGCCAATACACAGCCCTA

>scaffold_210000245-3

ATACAGGGTTGGTGAAAATTAACTAGGCAATATTTAATGGCTGTAGAACTTGTAGTATCACTGGAGTCATGATGAAAACAGTCTCTAAATTGGCGATAGTAAATAGGATTTCAAGCCTCTCCCACTGGAGGACATGGAGGCTCGGATTCAGGAGGTTCTCAGCAATATCCCAAACGACTTCCTTCAGAATACTGTGCATTCCATCTCCATCCGTTTGAGGAAACTGGTTGACGCCACCGGTGCCTACAATGAAATTTAAAGATTTGCTTTCATTTTCCTATGTTATAAAGTACATGTACGATTTGTTTCAATCAATTTGTATTAGAAATATGTACTTTACTACCAATTTTAAATGCCTAGTTACTTTTTACCAACTCTGTAT

>scaffold_210000245-4

TCTAAAGCCGGGCTTACACTGTGGGATTTGGCCACGATTTGGTCGTCTGAGATAAACTTTCAAATCCTAAAAGATTTGTACACTAAAATCTGTTGTCTTTGATGACTAGTTTGACATGTTCAGCGACAGCCGATTAATGGCCGTTGCAATTGTTTTTTTCCTCCGATGAAATTCTAACAATGTCAGAATTTCAGACACTTTCCTGCGGTGTGACTTCTCCTACAACGACCGTAAAACCAAGAACCGATAGGAGCGCCAAACCTGATGACGCAATTAGCGCGACAACTTCAAACCATTTCGGAAAAATGTCTAAACGAGTCCGGTGGACAGTACAGCAAGAAGATAAACTTATTGAGGCATGTCATGGGCAACATTAACGCTAGAGATTGTTTTGTTTGCTGTTAGCTACCATTTAGCGAGAGAACGCGGGTTACTGAACGTGTCACAGATTGATGACAGAAAACTCCGGACGAGTCTTCTTGTGTGCGTCCGTTTTTGAGGCGTCTTGACTGTCGTACAGTCTGACAATATGACAACTGAGATTCTACAGTGTGACATGGGGATCGTGTTTGTACAGCCTGACAAGCAACAATCACAAAGGACTATTAAAAATCGCACAGTGTAATCCCAGCTTAAGA

>scaffold_210000245-9

TACAGGATGGGTGAAATTGAACTAGGCAATATTTAATGGCTGTAGAACTTGTAGTATCACTGGAGTCATGATGAAAACAGTCTCTAAATAGACGATAGTAAATAGGATTTCAAGCCTCTCACACTGGAGGACATGGAGGCTCGGATTCAGGAGGTTCTCAGCAATATCCCAAACGACTTCCTTCAGAAGACTGTGCATTCCATCTCCATCCGTTTGAGGAAACTGGTTGACGCCACCGGTGCCTACGTGGAAATTTTAAGATTTGCTTTTATTTTCCTATGTAATAAAGTACATGTACAATTAATTTCAATACATGTGTATTAGAAATATGAATTTTAGTACCAATTTTTAATGCCTAGTTACTTTTTACCCACCCTGTA

>scaffold_210000247-4

TTAAAGAGGTCATGAACTACCTCAGGTTTTTTATTTTGTACTGTTCTGGGAGGACAATTATAATGTTATCAAGATTTTTACATTTAAAAAAAACCCATCATACTTTAATAATAGTACTTTAGTAATAGGCAATCTTCTATTCTGTTTTTGACACCCCCAACCCCCCCTGTGGCGGATTGTTGTCTCGGAAGTAAACGCCCACTGCTATGATTGGCTAACAGTTTTGCATTAAAAAAAATATATACCCATCACGTGCATGCGCGAAGCGGTGGGCGGGGCTAAACAGACAGTGATCTCGAAGCAGGCGTCGATCTTCTGTGGCGGAGGCGGCGCTTATCCACACTATTACATCATCGAGTAGAACATTCCAAAAGCTGTTGTTTTGGCCTTCAATACAAGTTGGTTTTTTTAGAGTAACGATAAAGTTTTGAGTTGTAAAACTTACAGGAGGTTTTATAGCACACATTGGCCTCTTATATGTCAAAAGACCAAGGGAATTTAGATTTCTCAGTTCATGACCCCTTTAA

>scaffold_210000247-11

TAGGGCTGTGTATTGGCAAGAGCCTCACGATACGATACATATCACGATACATGGGTCATACAATACATATTGCAATACTTAAAAAAATATATATATTTATTTATATACACTTGCTGTGTACCACCTGTGGGGATTCTAGTATGTGTATCACATTCTATTGATTACTCAGCAGACAAATAAAAACGGAGGGTTTTAATGAAATAGTAACGTAAACGTAAATCAGTTAAAAAAAACGTCATTTAAATATGCATCGATACTAGAGGTTACAATATCGATACACTATCGTGAAAAAAAATATTGCGATAGTTAGCTGTATCGATATTTTTACACAGCCCTA

>scaffold_210000247-13

TAGGGCTGCACGATACTGGAAAAAATATGCGATATTGTTATTGAGTATTGCGATAACGATATTTCTTGCGATATAACATTTCCCTAGACAAATGCTATTTTTATTAGCTATTTTAGCTGAGGGTGTTTTTTCAGGCTGGCTCATCTTGTTAAGTTTAGCAGCTTGTTGATTGTCTTGCTTCAAAGCAGGCGCACGCAAATCCCAACTAACAACAGCGTGCCAGATATTTTACTATCCTAAAACTTTGACCACACATTACTATTTGGAGTATTAAATCGCTCATTTATCGCAAACCATTGCGATATGCATATCTCGACATGCGCATTTGCGATATTTCAATAATTTCGATATATTGTGCAGCCCTA

>scaffold_210000248-23

CAGGGGCGCCGCTAAGGGGGGAAAGTAGGACGATTCTAAGGGCCCACACACTTTGGGAGCCCCCAGAGATCTGTTTTATTAGTAGTAACAGACTGTTAGGGTACCGTAGAGGGCCCAACCTCATATTCTTTCATAGGGCCAAAAATTGCTAGCGGCGACCCTG

>scaffold_210000249-3

TTAAAGGGGTCATGAACTGAGAAATCTAAATTCCCTTGATCTTTTGACATAGAAGAGGCCAATGTGCTATAAAACCTCCTGTAAGTTTCAGAACTCATAACATTGTCGTTACTCTAAAAACAGCTTATATTGAAGGCAGTCGGCCCTAACAACAGGTTTTGGAATGTTCTACTCTATGACGTAATAGTGTGGATAAGCGCCGCCGCCGCCACAGAAGATCAACGCCTGCTTCGACATCACTGTCTGTTTAGCCCCGCCCACCAATTTGCGCATGCCCGTCCAAAACTGTTAGCCAATCATAGCAGTGGGTGTTTAGTTTCGAGACAACAATTCGCCACGCCTATTCAAACAGAGCGGTCTGATGAGGGAAAATAGCCAATAACTTCTAAATTTCTAAAGTTTTTCTGATGTAAAAATCTTGATAACATTATAAGTGGACCTCAGAGAACAGTACAAAATAAAAAACTGAGGTAGTTCATGACCTCTTTAA

>scaffold_210000250-1

AAGCCGGGTTCACACTGTGCAATTTTAGCCACGATTTGGTCGTCTGAGACAAATTGTGAAATACCAAAAGATTCCTATAATCCTACGCTAAAATCTGTTGTCTTTGATGGCTAGTTTGACATGTTCACCGACAGACGATTAATGGTCGTTGCGATCAGTTTTTTCCTCCGATGAAATTCTGGCAGTGTCAGAAGATTTCGGACACTTTCCTGCAGTGTGTCTTCTCATATGACGACCGTCAAACCAAGAACCAATAGGAGCGCCAAACCTGATGACGCAATTAGTGCAACAACTACAAACCACCTCAGGAAAATGTGGAAACGAGTCAGGTGGACAGAACAGAAAGAAAAGAAAGTTATTGAGGCATGTCATGAGCAAAATTACCGCTACAAATTGTTTTTGTTTGTTGCTAGCTACCATTTAGCGAGAGAATGCGGGTTACTGAACGTGTCACGGTTTAATGACGTAAAACTCCAGACGGGTCTTCTTGTGTGCGTCCGTTTTTGATGCGATTTGACTGTCGTACAGTCTGACATTATGCCAACTGAGATCCTACAGTGTGACATGGGGATCATATTCGTACAGTCTGACAAGCAACAATCGCAAAAGTCTATAAAAAATCGCACAGTGTAATCCCGGCTT

>scaffold_210000251-31

TTAACCCTCATGTGGTGTTCATGTTTTTGTTACCTAGCCAATGTTCACAGGTCTGCATTATTGGGTTTTTAATCAACACAGCCATAACAATTTATGGAAAAACACTTAAAAGATGTTTACTTTAGTTCACTTACAAGCAATATAGACATAATTTGTGCTTAATATTTACTATTTACCCCGTTAGATCACATTTGAAAATATAGGTGTTATGAAAAATATGTTCTATTTTATGTCCTACATGGAAAATTAGCCAAGACGAATGAGTCTGGGGCTAAAAAAAAATAATTAATAGCATAATTTCTCTTTAAACAAACTTTGAAATCGGATCACACAGACCCAAACGCCACACAAGGGTTAA

>scaffold_210000253-2

ATATCGAGGGGTGAGAACCAGAAGGGCGTAAGTGACATTTTGGGTGAGATGGAGATATATATATTAAAAGAAAGCTCTTGATGAGCTCTTCCAAAGTATGCTGTCATCCATAGGAGTACACATTTTTATTATAAAAGATTGAAATCAGTACACAAAGTCAGATCAAACTATGGTCATGTTTTTGTTTTTGGCCCTCCTGTAAAGTTGGTGAAATGTCACTTACGCCCTTCTGGTTCTCACCCCTTGATAT

>scaffold_210000253-3

ATACACTTTATGGCCAAAAGTTTTTGGACACCTGACCAGCACATGCACATAACTGTTATCACAAAGTTGGAAGCACACAGTTGTCTAGAATGTCTTTGAATGTTGTAACATTACATTATTCCCTTGATGGACCTAAAAGACCCAAACACTGTTCCAGCATGACAATGCCCCTGTGCACAAAACACCTGAGCTCTGTGAAAAGATGGACTGTTATGGTATGTGTGGAAGAACTCAAGTGTCCTGCACAGAGCCCTGATTCTGACTCAACTCCATTGAACACCTTTGAGCCAAACTGGAACACTGACTGAATCCCAGACCTCCTCACCCAATATTAGTGCCTGAACTCACTAATACTATTGTGAATGAATGACAAAAGATTCCACAGCCACATTCCAATTCCTAGTAGAAAGCCTTTCCAGGAGTGTGGAGGTTATTATAGGAGTAAAAGTGAGACTAAATCTGGAATAGAATGTGTCAAAAAACACATTTGGATGTGATGGTCAGCTGTCCCAATACTTTTGTTCATATAGTGTAT

>scaffold_210000254-2

TACAGTATATTGCTTAAAATCTTGTGGACACCTCACCATCAGATCCATATGTGGTTCCTCTCCAAAATGTTGCTACAAATTTGGAAGCACACGTTTGTCTAGAATGTCTTTGTATGTAGTATTACATTATTCCTTCACTGGAACTAAGAGACCCAAACACTGTTCCAGCTTGACAATGCCCCTGTGCACAAAACCCCTGAGCTCCATTAAGACATGCTGTGTTATGATTGGAGTGGAAAAACGTGAGTGTTCTGCACAGAGACCTGACCTTAACACCACTGAACACCTTTGGGATGAACTGGAACGCCGACTGCACCACAGACCTCCTTACCTGACGTTAGTGTCTGATCTCACTAATTCCAATCTAGCTGAATGAACACATATCTCACATTCAAGCTCCACATTGTAGTGGAAAGCCTTACTAGAAGAGTGGAGCTTAATTGAACAGCAAAACAGGGAGTAAATCTGGAACAATATGCTTAAAATCCCAAATGTTTGTGATGTTCAGGTGTCCACAAACTTTTGGCAATATAGTGTA

>scaffold_210000256-2

CTACGTTCACACTGCGGCGAATGTTTCCCAAATCCGATTTTTTGCCCACATGTGACTCAGATCTGTTTTTCTCATGACAGTGTAAACAGCACAAATTGCATGGAATCTGATCTGTCCAATTCCAATTTGTGCCACTTCCATATGTGGTATTAAATCCGACACAGGATTCACCGCAGTGTGAACAGTTATGTCAGAAGTCATGTGACTTTTACGTAATTTTCGATCGACATGCGTCATCATTCTGCACTGGCGCGTAACTACAAACAGTTGATATATAATCGAGTTCGTTGAAGGCGTCACGTCACCACTCCTGGCTCCGGCTCCGCATCCACACACCTCAATACTGAAGTAGCAGCCACTGCTCCACAACATGCTATTATTCAAAATGTGGCTTTTCTTCAACAACTGCTCATTAATTCGTTGTTGTTCGCTGCACATCACCTTCTAAATGTAAGCAGCTAACGCGTAAATGCTGCCTTTAGTGTCTTCCATCTTTAATTTTCTGTATGACAGGACGCTGCGCGAGTCGTAGAATTTGTTCTTTTGTGCATGCGAGTCAGTTTAGGAGCATGATCAGTTCACACTGGAAATCTGATATGGGCCACATTTAAAACAACAATGTGAACAGCTATACAAAAAAATCTGATCTGAGCAAAAGATTTGAGCATCTAAGCCTCGCAGTGTGAACGTAG

>scaffold_210000257-19

CCAGTCCTTCTCAAATAGTGGGGCGCGCCTCCCTGGGGGAACTCGGAGCGATGCCAGGGGGGGCGCGTGTGACCCCGGGGAACATGCTTTTTTTTGCCGCAGGGAGTAGGGTTTTTTTTGCACCGAACAAGAGCACACAGCACAGAGCAGGAGATATGAAGTGGAGATAACAAACCCTTAAGAGACACCATGGAAAAATATTTAACAGGGATGAAAAGAAAGGCGGAGAGAGACGGAGATAATGAGACAAACGTAAGTCTCCCGAAAGTTAAGACGAGGAAATATGACGAAGCGTATGTAGCGCTTGGCTTCACTGTGACTACGGTGGGAGACGAGGAAAGACCGGTATGTTTACTGTGTCTAAAAATGTTGGCAGCGGACAGCATGAAGCCAAATAAATGAAGGCGTCACTTAAAGACATTACACCCCAATCACGCTGATAAGCCGCTTGAGTTTTTTCAGCGAAAACGTGCCGAATATTGCCATAGTTTACAGATTAAATACGGATTGATTTTTTTTTTATTTCAGGCAAATTGATGCACTTTAAGTCTTTTCTGTTACAGACTAAAAAACAATGTTAAAGTTATTCTTTGTTGTAAGTTGATCTATATTTCTTTCTTTTTTTGTTTTCTTTAATGTTAATAAGGATACAATGTTATGCAGAGGTGTACTTATAACAATTTTATAGACAAATGTTACTATTTACAGTCGCGGCGGAGAGTTGGGGGGCGCGAAATGTTTACTTCTTCCTAGGGGGGCGTAACAGAAAATAATTGAGAAGCACTGG

>scaffold_210000258-2

TATCCTCCTGAGACGCCGCCCATTGACTTGTGTCTTCTATAGTGGACATTTTATTTTTTATTTTTGAGCTACTCTATCAATTTCTGCTGTCCTGTACAGAGGACATCCTGGGCTTTCTAGTGATATGTCATTTGATTGGTTGGGATGCTAGGCCCTAGTTCTTACACTGCATCCAAAATGGCCTCCATAAGAACAAGCATGTTTTATGGAAAGGACTGTCTTTTTATACTAAAATAGTCCTGGTATCCACTTCAGTGGACATGCTGTAAAATAAAAAATCACAATAACATTTAAAAACTATTTCAACCTGAACGGAGTAGGAAATCACACAAAGCAAAAAAAAATATGATGCCACTTTTTTCATTGGGTCTCAGGAGGATA

>scaffold_210000260-2

TATGGCTCTGTCCGAAATCGCATATTACTTAAGTATGTTCTACATTTGAGTAAGTATGTACTTCCCGACCGTTAAAAAGTGTGTTCTATACAGTGTGGTTATGCGTAGTATGAATGAAATTCGGACGTACTACATCCGCCATGTTGATATTTTCATGGGATAGATGTCGTCATAGCAGCACAAATTTAAAGTTAAATAATGTTTACTTGTTAAACAAGTGCAATTTAACCACAAGTAACAATGTTTTTTTTTAAGTAGCTACTTAAATCACTTGAAAAGTGCTGTGTCGCCGCTTTCGGCAACTTCCCCATATGACGAGTTGTCTGTCATGTGGGCTCATGGGATAGTAAAGTGTACATCGTATGCATACTTCAAAATCTGGGCAAAAGTAGTAGATCATCCGGGAACTTTTGTGTACTGTATTTCGCATACTTAGGATTCGGACATACTACTTGCCTCGCATACATAGTTTCACATACTAATAGTATAAAAGTATGCGATATCGGACGGAGCCTATA

>scaffold_210000260-7

TAGGGATGCACTGATATCATTTTTTAAAGACCGAGTACCAATATGTTTTTTCCTGGTATTCACCGATACCGATACCTGTACTTTTTTGGGATGTGGGATTATTTGTATTGGTATGGCTCTCTGGTTATCTCTAGCCATTTTCTCTGGTCTCTTTCTCTTCTTTCTCTCAGGGTTGGTTGCCGAGTGCTAACGTTACTGCTAGCTGCAAATTCTCCGTGCTCACTTTGGAGTTTTAATTTCAAATGTTTTATCAGATTACTCGTATTATAATTACTTATTTTTGTACCTCTTGATATTTTTGCGGAACAAAGTTTGCAGTCTGCCAATGCGTGGGTTGTCATCATTAAGTTTGAAATATTTCCACACCGCTGAGCCTGACATTCTGTCGCTGCTGCCGGAGCCTTTGTGCCCTGAAAGTTCTGTCAATGACGTCACAAGAAGTATCGGTCTTTGGTGTTTTTACAAGTACGAGTACATGAGCTCAGTATCGGGCCTGATACCGATAACAGTATCGGTGCATCCCTA

>scaffold_210000260-10

TAGCCCCGTTTACATGGAGCATTGTATTCCGATTACAATTAATTTAAAAGTCCAATCCGAATGAAAAAGCCTCAACGAATGTAAACACCTCAACCAGAATAAAAATGGCCAACCCTATAAAAATTTAAATCGGGTTGAAGTAGGTGGAATAAACCTTTTCACAACCCGTATAAATAAAAAAAATTCTCCATGTAAACGACTTATTCCGGTTACTTTGAATGTACGTCCTTCTGCGCATGCCTGATCTTGACGTAATTTCTCGGGAAGTGACCGGCATGTCACGCGAAAGTTTTGTTTTCATTCCATATCGCTAAATTTCCATAGCATAACTCTCACCAGTGCTTATTACGGACTTTCATTCACAGGATTCTGCTCTTTGGAGGGGGAGGGGCAGCAAATCGATGGCTCGTAGCGAAGCACTGCTACAGAAAAGCTCAGCTTCTTGCGCTCCGTCTTTTCATAAATTACTTCAGCAACAGCGCATACTAACGTACTTAAGAGCAACAAAATGCCCATAACTAGTGCGATTAAGCGGCGCAGCTCATCTTTACAGGACTGAAATACATGAAATAATAAAATTCACGCAGTGACAGACAAGCTGTGCGCATGTCACAAAATTATTCCGACTGAAAGCGTCGGCACATGTAAACACCAGTTCAGAATAGATCACGTCTAATGTAAACACTCAAATATTTAAATCGGAATTATTTCAGTTGGAATGACAAAAAAGTGTAAATGTAAACAGGGCTA

>scaffold_210000260-15

GGCTCCATCCGAAATCGCATACTTCCATACTATATAGTATGCCAAACTGAGTATATAGGCAAGTAGTATGTCCGAATCCTATAGCATGTGAAAAGCAATAGGTGAAAAGTTCCCAGATGATCTACTACTTCCGTCCGGATTTTGTAGCAGGCATACGATGGACATTTTACTATCCCATGAGGCCGCGGGAGAGACGACTCGTCATATTCGGAAGTGGCGAAAGCAGAGCCACAGTTTTCAAGTGCTTTAAGTAGCTTTAAGTACTTAAAGAAAAAACATTGTCCGTTGTGGTTAAATTGCACTTGTTTAGCATAATCTAATTAAACATTATCCAACTTTAAGTTTGCGCTGCTGTGATGATATATTACATATTACGTGACAATATCAACATGGCGCATGTAGTACGTCCGAATTTCATTCATAGTACACGTAACCATAATATATAGAACTTCCTTTTTTACGGTTGCAAAGTAAATACTTTCTTAAATGTAGTACATACTTAAAGAGAATGCAATTTCGGACGCAGCC

>scaffold_210000261-6

TTAACCCTTTTGTGGTGTTTGGACCCGATTTCAAAGTTTGTTTAAAGAGAAATTATGCTATTAATAATTTTTTTAGCCCCAGACTCATTGGCCTTGGCTCGTTTTCCATGTAGAACATAAAATAGAACATATTTCCATAACACTTATATTTTCAAATGTGATCTAACATTAGATGGCAAATATTAACCACAAATGATGTCTATATTGCATTAAGTGAGCTAAAGTAAACATTGTTTAAGAGTTTTTCCATAAATTACTGTGTTGATTAAAAACCCCAATAATGCGGTGGGTCCACCAGACCTGTGAACATTGGCTAGGTAACAAAAACATGAACACCACATGAGGGTTAA

>scaffold_210000262-3

TAAACATACACTATATGGCCAAAAGTTTGTGGACACCTCACCTTGGGATTCAGATGTGTTTTTTCTGTCTGTCACAGATTTGCAGGCACACAATTGTATAGAATGTCTTTGTATAATGTAACATTATAAGTTTCTCTTTACTGTAAGTAAAACTGCTTAAACACTGTTCCAGCTTGACAATGCTCTGTGCACAAAGCCTATGAGCTCTATGAAGACGTTGTGTTATGGTTGGCTTGGAAGAACTCGAGTGTCCCGCATGGAGCCCTGATCTCATCCCCACTGAACACCTTTGAGTAAACTAGAACACTGACTAAACCTCAGACCTCCTCACCCAACTTCAGTGCCTGATCTCTCTAATGTTCTTGTAGCTGAATGAACAAAACATTTTCACAGCCATGATCCAAATTGTAGTAAAAAACTTTCCCAGAGGAGTGGAGCTTATTGTAACAGCAACAGGTGAATAAATCTGGAATAGGATCTACAAAATAACATGTGGTTGTGATGGTCAGGTGTCCACAAACCTTTGGCTGTATAGGGTATGTCTTA

>scaffold_210000264-3

ATACACTATATTGCCAAAAGTTTGTGGACACCTCGCCATCAGATACATATGTGGTTCCTCTCCAAAATGTTGCTACAAAGTTGGAAGCACACAGTTGTCTAGAATGTCTTTGTATATTGTAGTGTTACATTAATCTTTCACTTGAACTAAGAGACCCAAACAGTGTTCCAGCATGACAATGCCCCTGTGCACAAAGCCCTCGAGTTCCATTAAGACATGCTGTGTTATGATTAGAGTGGAAACACTTGAGTATTCTGCACAAAGCGCTGATCTTAACACCACTGAACACCTTTGGGATGAACTGGAACACCGACTGCACCACAGACCTCCTTACCTGACGTTAGTGCCTGATCTCACTAATATTATTGTAGCTGAATGAACACATCTCTCACATTCAAACTCCAAATTGTAGTGGAAAGCCTTACAAGAAGAGTGGAGCTTAATCTATCAGCAAAATGGGAAGTAAATCTGGAATAAGATGTTTAAAATCACATATGTTTGTGATGGTCAGGTGTCCACAAACTTTTGGCAATATAATGTAT

>scaffold_210000264-6

GACTAGGGCTGTGTATTGGCAAGGGCCTCACGATAAGATATGATACATATCCCGATACAATACATCATGATATATTGCAATACAATACATTGCAATATTTCAAAAACTTTTTTTGATGTAAAACAAAAAAGCTAAAAACACAACTTGCTGTGTAGCACCTGGGGGATTCTAGTGTGTGTGTATCACATTATATTGATGACTCAGCAGACAAATAAAAACGGAGGGTTTTAATTCAATATTAACGTAAACATAAATCAGTTTAAAAATTATTTTAAACTTGTATCGATAATAGAGGCTAGAATATCGATACACTATTGTGAAAAAAATTATATTGCGATAGTTAGCTGTATCAATATTTTTTACACAGCCCTAGTC

>scaffold_210000266-3

TACAGTATATTGCCAAAAGTTTGTGGACACCTGACCATCACAAACATATGTGATTTTAAACATCTTATTCCAGATTTACTCCCCATTTTGCTGATAGATTTAGCTCCACTCTTCTAGTAAGACTTTCCACTACAATTTGGAGTTTGAATGTGAGAGATGTGTTCATTCATGTACAATAATATTAGTGAGATCAGACACTAACGTCAGGTAAGGAGGTCTGTGGTGCAGTCGGGGTTCCAGTTCATCCCAAAGGTGTTCAGTGGTGTTAAGATCAGCGCTTTGTGCAGAATACTCAAGTTTTTCCATTCTAATCATAACACACTATGTCTTAATGGAACTCGAGGGCTTTGTGCACAGGGGCATTATCATGCTGGAACACTGTTTGGGTCTCTTAGTTCCAGTGAAAGATTAATGTAATACTACAACATTCTAGACAACTGTGTGCTTCCAAATTTGTAGCAACATTTTGGAGATTAACCACATATGGATCTGATGGTGAGGTGTCCACAAACTTTTGGCAATATAGTGTA

>scaffold_210000270-1

CAGGGCTGGGTAACCCATGCTCCTTAAGTTACTTTGTTCCGCTTGTTTTCCAGCTCCCCCTGCCCCGCCCCACTGCTGATTACCTGATTCAGGTGTGTTCAGTCAATCAGAGGGTAGGAGATACCAAATCACCAGGACTTTCCCCACTCGACCCTCATCTGAAATGGTATCTTCCAGCTTCTGATTCACTGAACACACCTGGGGTAGCTGGAAAATAAGTGGAACAAAGTAACTTAAAGAACATGGGTTACCCAGCCCTG

>scaffold_210000270-8

TATATCCTCCTGAGACCCAAGAAAAAAGGTTTTTTTGTTTTGTGTGACCTTCTACACCTTTTGGGTTAAAAAACATTCTACAATTTTTTTTTTTTATTGTTATTTTTAATTTTACAGCATGTCCACCGTAGTGGACCACAGGACAATTTTAGTTTAAAAAGACAGTCCTTTCCATAAAACGAGCTTGTTCCTATGGCGGCCATTTTGATGCAGTGTAAGAACTAGTTCCTAGCATCCCAGCCAATCAAATGACATATCACTAGAAATCCCAGGATGGCCACTGTACAGTACAGCATACATTGATAGAGGAAAACAAAAGGTCCACTACAGAGGACACACGTCAATGGGCGGGGTCTAAGGAGGACATA

>scaffold_210000271-1

ATACACTATATTGCCAAATGTTTGTGGACACCTTACCATCAGAAACATGTGTGATTTTAAACATCTTTTTCCAGATTTACTCCCCATTTTGCTGTTCGATTAAGCTCCACTCTTCTAGTAAGGATTTCCACTACAATTTGGAGCTTGAATGTGAGATATGTGTTAATTCAGCTACAATAGTATCAGTGAGATCAGACACTAACGTCAGGTAAGGACGGAGGTGTGTGGTGCAGTCGTGGTTCCAGTTCATGTCAAAGGTGCTCAGTGGTGTACAGGTCAGGGCTTTGTGCAGAACACTCAAGTTTTTCCACTCCAATTATAACACACCATGTCTTAATGGAGAACAGGTGCTTTGTGCACAGGGGCATTGTCATGCTGAAACAGTGTTTGGGTCTCTTAGTTCCAGTGAAGGATTAATGCACTGCTACAACTTACAAAGACATTCTAGACAACTGGGTGCTTCCAAATTTGTAGCAACATTTTGGAGAGGAACCACATATGGATCTGATGGTGAGGTGTCCACAAGCTTTTAGCCATATAGTGTAT

>scaffold_210000272-3

ACTATATTGCCAAAAGTTTGTGGACACTTGATCTTATTCTAATGATCTGTCTTAGTGGAATATTGATCAGATTTACTCCCCATTTTGATGTTAGATTAAGTTTCACTCTTCTAGTAAGGATTTCCACTACAATTTGGAGCTTGAACGTGAGATTTGTGTTCATTTAGCTACAATAGTATTAGAGACTAACGTCAGGTAAGGAGGTTTGTGGTGCAGTCGGCGTTCCAGTTCATCCTAAAGGTGTTCAGTGGTGTTAAGAACACTCAGGTTTTTCCACTCCAATCATAACACACTATGTCTTAATTGAGCTCAGGGGCTTTGTGCACAGGGGCATTGTCATGCTGGAACAGTGTTTGGGTCTCTTAGTTCCAGTGAAAGAATAATGTAATTCTACAACATACAAAGACATTCTAGACAACTGTGTGCTTCCAAATTTGTAGCAACATTTTGGAGAGGAACCACATATGGATCTGATGGTGAGGTGTCCACAAACTTTTGACCATATAGT

>scaffold_210000273-3

TACAGGGTGGGTGATATAACTAGGCAATATTTAATAGCTATAGAACTTGTAGTATCATCGTAGTCATGATGAAAACAGTCTCTAAATAGACGATAGTAAATAGGATTTCAAGCCTCGCACACTGGAGCACTTGGAGGCACGGATTCGGTAGGTTCTGATCAATATCCCAAACGACTTCCTTCAGAAGACTGGGCACTCCATCTCCAGCCGTTTGAGGGAACTGGTTGACGCCACCGGTGCCCTTGTTGAAATTTAAAGATTTGCTTTCATTTTTCTATGTAATAAAGTGCATGTACAATTTGTTTTAATAAATTGATATAAGAAATATGGACTTTATTACCAATTTTTAATGCCTAGTTACTTTTCACCCACCCTGTA

>scaffold_210000274-1

ACTATAGTGTCAAAAGTTTGTGGTCACCTGACCATCAGAAACATGTGTGATTTTAAACCTCTTATTCCAGATTTACTCCCCATTTTGCTGTTAGATTAAGCTCCACTCTTCTAGTAAGACTTTCCACTACAATTTGGAGCTTGAATGTGAGATGTGTGTTCATTCAGCTACAATAGTATCAGTGAGATCAGACACTAACGTCAGGTAAGGAGTTCTGTGGTGCAGTCGGCGTTCCAGTTCATCCCAAAGGTGTTCAGTGGTGTTAAGATCAGGGCTTTGTGCAGAACAATCAAGGTTTTCCACTCCAATCATAACACACCATGTCTTAATGGAACTCAGGGGTTTTGTACACAGGGGCATTGTCATGCTGGAACAGTGATTACATTATTTATTCACTGAAACTAAAAGACCCAAAGTTCTAGACAACTGTGTGCTTCCAAATTTGTAGCAACATTTTGGAGAAGAACCACATATGGATCTGATGGTCAGGTGTCTACAAAGTTTTGGCAATATAGT

>scaffold_210000276-1

TAAACCTGGGCTTACACTATGCGATTGTGGCCACGATTTGGTCGTCTGAGGCAAATGTTGATATCCTAAAAGATTCCTATAATCCTACGCTAAAATCTGTTGTCTTTGATGGCTAGTTCGACATGTTCACTGGCAGCCGATTAATGGCCGTTGCGATCAGGTTTTCCCTCTGATGAAATTCTGGCAGCATCAGAAGGTTTCAGACACTTTCCTGCAGTGTGACTTCTCCCAAGACGACCGTCAAACCAAGAACCAATAGGAGCGACAAACATGATAAAGCAATTAGCGCGACAATTTCAAACCACCTCGGGAAAACGTGGAAGCGAGTCCGGTGGTCAGAACAGCAAGAAGAGAAACTTATTGATGGATGGAAAAGGAGTGTTTGTATGATGTGTCGCCTCTGCTGTTTGTTTGCTGCTAGCTACCATTTAACGAGAGAACACGGTTTACTGAACGTGTCACGGACTGATGAGGTAAAACTCCAGACGAGTCTTTTTGTGTGTAAATGTTTTTGACGCGTTTTGACTGTCGTACAGTCTGACATTATGATAACTGAGATTGTACAGTCTGACAAGCAACAATCACAAAAGACTATTAAAAATCGCACAGCGTAAGCCCGGCTTTA

>scaffold_210000278-3

TATAGAAGGTGGGTGAAAATTAACAAGGCAATATTTAATGGCTATAGAACTTGTAGTATCACTGAAGTCATGATGAAAACAGTCTCTAAATAGACACTAGTAAATCTGATTTTAAACCCCGCACCCTGGAGGATTTGAAGGCACGGATTTGGGAGATTCTCAGCGATATCCCAAACAACTTCCTTCAGAAGACTGTGCATTCCATCTCCAGCCGTTTGAGGAAACAGGTTGACACCACCATGTTTCACGATGTTGAAATTTAAAGAGTTGCTTTCATTTTATTATGTACAAAAAAGTACATGTACAATTTGTTTCAATAAATTTGTATTAGAAAAATGGACTTTATTACCAATTTTTAATGCCTAGTTACTTTTCACCCACCCTGTATA

>scaffold_210000278-6

TATCCCAGCTAACAGGGAATGTTCCCACAAATTTTGCTAATGTTCTTTAAAAGTTGTCTAAATGTTAAGACAAAATGTTCTTAAATAATATTCATGGAACATTCTCATAAAGTTACTAATAATTACTATACATTCTTGTGATGTTGAGAGAAAACATTCTTAGAACAATGTTCTTGAAACGTCCATCTGACGTTATTTGTATTTGATAAACATTCTCAAAATGTTTAGGGAAAACGTTCTTAGAACAGCACCATAAAATGACCATAAATTAACGTTCTACCAATGTTAAGCAACCTGGACATTTTCACGTTTTTGGAACATTTAAAATCACGTTCCCAAAACCAAAACAGAACGTTTGAAAAACATTCTTAGAACACACATTTGTTAGCTGGGATA

>scaffold_210000279-2

ATACAGTGGAACCTGGGCATATGAATTTAATTCGTTCTGGAGGCGATTTCCTAAGGCGAACATTCGTATTGCGTATAATAGGAATTTCCCCATAAGAAATAATGTATATACAGATACCAAAAATTTTACCAATTTCCAACACTATAATCATATTTTTGCATATAAAAACAATCAAATAATTTAGAAAAGACATGCAAATGAAGTAAAATATAAAATAAATAGACCCACACCACCAATATGTCAACAAAAAATGCGCGAATAATCACGTAGCTTTTGAATGCATGCCAAAGGCGACGTCGGTATCCTGGGTTGACTCTTTCGGAACAGCTTTCGCTGACTGAAAAAAATATATAAAAAAATTCGTAAACTTGCTTCGGTTGGCTTCGCTTGATTTCCCACACATGCAAACAAGTGTTGAACGGCTCCTGGACGTACACGCAACTTAGCTGGTGTTTGGTTTGGTTCAGTTTGTTTGTATGCCAAAAATGCTTTGTATGCCGATGCAAATTTCTTGCAAATATTCAATTGTTAAGGCGAAAATTCATAAGGGAGGGCATTCGTATGCCAAGGTTCCACTGTAT

>scaffold_210000280-2

TATATGGCCAAAAGTTTGTGGACACCTTACCAACACACAAACTGTTGCCATAAGTTGGAAGCACACAGTAATCTAAGATGTCTTTACATGTTGTAGCATTAAAATTTTCTTTTACTGGAACTTAAAGACTCAAACCCTGATCCAGCATGAGAATGCCCCTGTGCACAAAGCCCCTGAGCTTCATGAAGACATGCTGTGTTCTGGTTGGAGTGGAAGAACTCTCATGTCCTGCACAGAGCCCTGACTCTGACTCAACCCCACTGAACACCTTTGGGATGAACTGGAACACCGACGGCACCCCAGACCTCCTCACCCAACATTAGTGTCTGATCTCACTAATACTATTGTGACTGAATAAACACAAATCCTCACAGTCACGAAACACAAGGAGCCTTTCCAGATGTGTGGAGGTTATTATAGGAGGAAGAGTGAGACTGCATCTGGAATATGATGTTCAAAAAACACATATAGATATGATGTTCAGGTGTCCCAATACTTTTGCCCATATA

>scaffold_210000281-3

AGTAAAGCCAGGTTTACACTGTGTGATTTTGGCCACGATTTGGTCGTCTGAGACAAATGTTGAAATTCTAAAAGATTCCTATAATCCTACATTAAAATCTCTTGTGTTTGATGGCTAGTTTGACGTGTTCACCGACAGCCGATTAATGGCCGTTGCGATAAGTTTTTTCCTCTGATGAAATTCTGGCAGTTTCGGAAGATTTCAGACACTTTCCTGCAGTGTGACTTCTCCTACAACGACCATCAAACCAAGAACCAATAAAAGCGCCAAATGCAATTAGCGCGATAACTTCAAACCACCTCAGGAAAATGTGGAAACGAGTCTGGTGAACAGAACTGAATGTAATGTAACTTATTATCGTTATATTGGGAAAAGGAGGGTTTGTATGACGTGTTGTCGCTGCTGTTTGTTTGCTGTTAGCTACCATTTAGCGAGAGAACGCGGGTTACTGAACATGTTACGGATTGATGACGTAAAACTCCGGACGAGTCTTCTTGAGTGCGTTTTTGACGCGTCATAATGCGACATTATGACAACTGAGATCAAACAGTGTGACATGGGAATCGTGTTCGTACAGTGTGACAAGCAACAATCGCAAACTATTTAAAATCACACAGTTTAAGTCCGGCTTAACT

>scaffold_210000281-4

GGCTATGTTCACACTGCAAGGCTTAGTGCTCAGTTCCATTTCTGCTCAGATCAGATTTTTTTGTATAGCTGTTTACATTGTTGTTTTAAATGTGGCCAATATCAGATTCCCAGTGTGAACTGATCATGGTCCTAAACTGACCCGCATGGACAAAAGAACAAATTCTACGTCTCACACAATGTCCCGTCATACGGAAAAATAAACATGGAAGACACTGAAAGCAGAATTTACGCGTTAGCTGCTTACATTTATAAGGTGATGTGCAGCGGACAACAGTGAATTAATGGTCATTAAGGAGGAAGAGGATGAGGAAGAAAGGGGTTGAGTGTTTAATAATAGCATGTTGTGGAGCAGTGGCTGTTACTTCAGTATGGAGGTGTGTGTGGATGCAGGGCCAGAGTCAGGAGTGGTGGGACCGTGACACGAAACCTTCAGCGAAATTATATATCAACTGTTTGTAGTTACGCGCCAGTGCAGAACCCATGTCAATCAAAAATTACGTAAAAGTCGCATAAATTCTGACATAACTGTTCAAAACATCTGACTTGTGTCGGATTTAATACCATATAAGGAAGTGGCACAAATCGGAATTGAAAAGATCAGATTCCATGCGGTTTGTGCTGTTCACACTGTCATGGGAAAAACAGATCTGAGTCACATGTGGGCAAAAAAATCGGATTTGGGCCACATTTACCTGCAGTGTGAACGTAGCC

>scaffold_210000282-3

TATACATTATATGGCCAAAAGTTTGAGCACACCAACATAAGTGTGTCTTTCCCAAATTGTTCCAGTCTGAAGCACACAGTTGTCTAGAATGTCTTTGTATGTTGTAACATTACATTATTCCTTCACTGGAACTAAGAAACAAACCCTGCTCCAACATGACAATGCCCCTGTGCACAAAACCCCTGAGCTCCATGAAGACATGCTGTGTTCTGGTTGGAGTGGAAGAACTCGAGTGTCCTGCACAGAGCCCTGACTCTGACTCAACCCCACTGAACACCTTTGGGATGAACTGAAACACTGACTGCACCCCAGACCTCCTCACCCAACATTAATGTCTGACCTCACTAATACTATTGTGACTGAATAAACACAAATCCCCACAGTCACACAACAAAATCTAGTGGAAGGAATTTACAGAAGTGTGGAGATTATTATAGGAGGGAAAATGAGACTACATCTGAAATAGGATGTTTATAAAACACATATGGATGTGATGTTATGATGACCCAATACTTTTGTCCATATAGTGTATA

>scaffold_210000283-4

AGGCTACGTTCACACTGCGAGGCTTAGTGCTCAATTCCAATTTTTGTTCATAGACGTTTTTTTGGATAGCTGTTTACATTGTTGTTTTAAATGTGGCCAATATCAGATTTCCAGTGTGAACTGATCATGGTCCTAAACTGACCTGCATGCGCAAAAGAACAAATTCTACATCTCACACAACGTCCCATCATACAGAAAAATAAACACGGAAGACACTGAAGGCAGCATTTACGCATTAGCTGCTTACATGTATAAAGTGATGTGCAGCCGACGACAGAGAATTAATGAGCAGTCGTTGAAGAGGAAGAGGATGAGGAGAAAAAGGGCCACATTGTTAATAATAGCATGTTGTGGAGCAGTGGCTGCTACTTCAGTATGGAGGTGTGTGTGGATGTGGAGCCGGAGTCAGTAGTGGTGGGACCGTGATGTGAACAACGCCTTCAGCGAAATCGATTATAAATCAACTGTTTGTAGTTACGCGCCAGTGCAGAATGAGGACGCATAGCGATCGAAAATGACGTAAAAGTCGCGTGAATTCCGACATAACTGTTCACACTGAGGTCGCATTGCAAAACATCTGACCTGTGTCGCATTTAATACCATATAAGGAAGTGGCACAAACCGGACTTGAAAAGATCAGATTCCATGCGGTTTGTGCTGTTCATACTGTCATGGGAAAAACAGATCTGAGTCACATGTGACAAAATAATCTGATTTGGGCCACATTTACCTGCAGTGTGAACGTAGCCT

>scaffold_210000284-3

TTAAAGGTGCAATAGGTGATTGTCTTCAGAAACATTTTTTGTTATGCTGGTTAAAAGTCTCTTCACATCCCGACAGCAATCATTAAGTTAAGTGGTCTAAATGTATTTATATATAGTTATATACACCAAGAAATTTTCGTCCAATCAAAATGTTTGCTCATTGTGATGGGCTTTCCTACCTGTCCGTCAACATATGTATTTGCATGCCTTTGCGCATCCTGTTTGCGCAGACAGCGTTCACATACGCTGTGCAGACAGAGCGAGAATGGAAGGCAAACAGCAACAACCTGACCTTGCTTCCCAACAACCGACGTCAAGTGGCCTTGTAAAAGTATCCACCAAAAGAAAGACTTTGAAAGGGCTACTGCGAAAAAAGGCTGGATCAAAACAGATCGAAAACCCGATTTAATATTGGTGTTGCGTTTCAAGAGGCGTGGCTTTGGACTGCGATTTGCGGGGAGGGTGGGACCTACGCTTTCAGTGCTATCAGGCTACAGTTAACATTTTCCAAGATGTCCTACTGCACCTTTAA

>scaffold_210000284-11

TGTACCATGAAGGTAGCTGAACAAACTCAGTGTTATAGGATAGGTTTTGAGTTGACAAAACCAAACAGATGCAATCAGACTTTATTGGTACCATGATGCTGATCATCATCTTTCTTTGCCAACACAGGCTTTGATCCTGAGTTTGTGGAGCGCGTGCACATGAATATGTGACATCAGTGTCGAACAGCCAATCACATGCCTTGCAACACAGAGCAAGAGACTTGAACATGCATGATTCTACAGCTTATTTATAGTACATATGTATATATTATTTTATATATACATATATATATATATATATAAACATATATATATATATATATATATTAATTACATATCTGTATATATTAATATTCATTTAAAATGAATTGCTTTATTATAACTGTTAAACTAAAATTGCTTGTGTGTAATGGATTAATAATATGTCATAGAATAATTATATAAATCATATAAAATAACTAAGTAATTATCATTAAAGCCAGACAATATTTTTTGGAAGCAAGTAACTGGGGGAGTTTTTTGCATCAGACATTTGTCACTTTGCTCACATCTGATTGGTCCAATTTCAGTTTGAGATCTCTAACCCCGAACATAATCTGCCTCGAAGCAGGTTAGCCGTGGAGTGTACGTTACTAAGGCAATGAACCCAGCTAAAACCCAAGCCACTTTCATGGTAACTAAAACCCAGGATTGGTGCAAACTAAACTGAAACTTACCTGGCTAGCCAGCTAATATGGCTTCATGGTACA

>scaffold_210000285-3

TAAAGCCGGGTTTACACTGTGTGATTTTTAATAGTCTTTTGTGATTGTTGTTTGTCAGACTGTACGAACATGATCCCCGTGTCACACTGTAGGATTTCAGTTGTCATGTCAGAGTGTACGACAATCAAAACGCGTAAAAAACGGATGCACACAAGAGACTCGTCTAGAGATTTACGTCATCAATCCGCGACACTTTAATAACCGCGTTCTCTCGCTAAACGGTAGCTAGCAGCAAACAAACAGCAGCGACGACACGTCATGCAAACACTCCTTTTCCCTCCATAACTCAATAAGTTTCTCTTCTTGCTCTCTTCTCCACCGACTCGTTTCCACATTTTCCCCAGGTGGTTTAAAGTTGTGGCGCTAATTGCGTCATCAGGTTCGGCGCTCCTATTGGTTCTTGGTTTGATGGTCGTTGTAGGAGACATCACACTGCAGGAAAGTGTGTGAAATCTTCTGACACAGCCAGAATTTCATCGGAGGAAAAAACTGATCGCAACGGCCATTAATGGTCTGTCGGTGAACACGTCAAACTAGCCATCAAACACAACAGATTTTAGAGTAGGATTATAGGAATCTTTTACGATTTCAAAATTTCTCGTGGCCAAAATCACACAGTGTAAACCCGGCTTTA

>scaffold_210000285-11

TAGGGCTGCACAATATATCGAAATTATCAAAATATCGCAAATGTGCATGTCGAGATATGCATTTCGCACAGGTTTGCGATAAATAAGCGATTTAATACCTCAAATATTAATGTGTGGTCAAAGTTTTAGGATAGTAAAATATCTGCTACGTGGATGTTAGTTGGGATTTGCGTGCGCGTGCTTTGACGCAAGACAATCAACAAGCTGCGAAGCTTTCATTGTGAGCTAATCTTGATAAGATGAACCAGCCCGAAAAACACACGCAGCTAAAATAGCTAATAAAAATAGCATTTCTCTAGGGAAATGTTATATTGCAAGAAATATCGTTATCGCAATACTCAACAACAATAACGCATATCGCATATTTTCCCAGTATCGTGCAGCCCTA

>scaffold_210000287-17

CTAGGGCTGCACAATATATCGAAATATCGCAAATGTGTATATGGTGTTATGGATATCGTAAAGGTTTGTGATAAATAATTGATTTAATACTTCAAATATTAACGTGTGGTTAAAGTTTTAGGATAGTAAAATATCTGGCGCGCGGATGTTAGTTGGGATTTGCGTGCGCGTGATTTGACGCAAGACGATCGACAAACCGCGAAGCTTTCATAGCGAGTTAATCCTGATAAGATGAACCAGCCCGAAAAACACCCGCAGCTAAAATAGCTAATAAATATAGCATTTCTCTAGGGGAATATTATATCGCAAGAAATATCGTTATCGCAATAATCAACAACAATATCGCATATTTTCCCAATATCGTGCAGCTCTAG

>scaffold_210000290-1

ATGCACTATATTGTCAAAAGTTTGTGGACAATTGACCATCACAAACATGTGGTTTTAAACATCTTATTCCAGATTTACTCCCCCATTTGGCTGTTAGATTAAGCTTCACCCTTCTACTAAAGCTTACTACAATTTGGAGCTTGAATGCGAGATGTGTGTTCATCTAGCTACAATAGTATTAGTGACATCAGACACTAACGTCAGGTAAGGAGGTCTGTGGTGCAATCGGAGTTCCAGTTCATCCCAAAGGTCTTCAGTGGGGTTAAGATCAGGGCTTTGTGCAGAACATGTTTTTCTACTCCAATCAGAACACACCATGTCTTAATGGCGCTCAGGGGCTTTTGTTCCAATGAAGGAATAATGTAATGCTACAACATACAAAGACATTCTAGGCAACTGTGTGCTTTGTAGCAACATTTGTAGCAAATTTGTAGCAACATTTTGAAGAGGAACCACATATGGATCTGATGGTGAGGTCTCCACAAACTTTTTGCATTATAGTGCAT

>scaffold_210000291-3

ATACACTTTATTGCCAAAAGTTTGTGGACAGCTCACCATCAGATCCAAATGTGGTTCCTCTACAAATTTGGAAGCACTCAGTTGTCTAGAATGTCTTTGTATGTTGTAGCATTACATTATACCTTCACTGGAACTAAAAGACTCAAACCCTGTTCCAGCATGACAATGCCCCTGTGCACAAAGCCCCTGAGCTCCATGAAGACTTTGTGTGTTATGATTAGAGTGGAAGAACTCGAGTGTTCTGCACAAAGCCCTGATCTTAACCCCACTGAACACTTTTGGGATGAACTGGAACACCAACTGCACCCCAGACCTCCTTACCTGACGTTAGTGTCTGATGTCACTAATACTATTGTAGCTGAATAAACAATGTGTGACAAATCTCACATTCAAACTCCAAATTATAGTGGAAAACCTTCCTAGAAGAGTGAAGCTTAATCTAACAGAAAAATGGGAGTAAATCCGGAATAAGCTGTTTAAAACCACATATGTTTGTGATGTCAGATGTCCACAAACGTTTGGCAATATAGTGTAT

>scaffold_210000291-32

TACACTATATGGCCAAAAGTTTGTGAACACCTTACCAGAACACCCACAAGTGTGTCTTTTCTAAACTGTTGCCACAAAGTCTGAAGCGCACAGTTGTCTAGCATTCCATTATTCCTTCACTGGAACTAAGAGACTCGAACCCTGTTCCAGCATGACAATGCCCCTGTGCACAAAACCTCTGATCTCCATGAAGACATGCTGTGTTCTGGTTGGAGTGGAAGAACTCTCATGTCCTGCGCAGAACCCTGACTCTGACTCAACCCCACTGAACCCCTTTGGACTGAACTGGAACCCCGACTGCACCCCAGACCTCCTCCCCCAACATTAGTGTCTGATCTCAATAATACTATTGTAGCTGAATGATCAAATCCTCACAGTCACATGACACAATCTAGTGGAAAACCTGCACAAAAGTGTGGAGGTTATTATAGGAATAAGAGTGAGACTAACATCTGGAATAGGATGATCAAATGATGGATGATACACAGATGGATGAGATGGTCAGGTGTCCTAATACTTTTTGCCATGTAGTGTA

>scaffold_210000292-1

CTTAAAGGCAGGGTAGGTGATTTGTTTCAAAAACATTTTTTGTTCTGCTGGTTGAAAGTCTCTTCCCATCTCGATAGCACTCATTACGTCAAGTGGTCTAAATGTATTTATATGTATTTATATCGTCTGTGGAAGGCGTAGGAACAAAACAACTTCGTCCAATCAAAACGTTCGGTCCGAACATTACGATAGGTTATCCTACCTGCCCGTCAAATATGTATTTGCATACCTCTGTGCACCCTGTTCGCGCAGACAGATTACGTCGTTAGCGCGTTCCCGCTATGCAGACCGAGTAAAAATGGAAGGCAAACAGCAAGAGACCAACATTGCCTCTAAACGTGGCACAGACACTCGCATGAGAAAAACAGTGTTAGAAAGAGTTACAGTATGTCTAAAAAAGTTTTAGGGCTAAAAGTCGAGTTAATCTAGGTGTATATCCGGTCCTGCCTGTGCATGCGCGTTCATGTGTTTTAAAGGAGGCGTGGCTGTACAGAGTGATTATGCAGGGAGGGCAGGATCTTTGGTTTTCAATGCTAGCTTGCTATTGCTAGCCTCTCCAAAATCGCCTACCCTAACTTTAAG

>scaffold_210000292-4

TAGGGCTGTGTAGAAATATCGATACAGCTAACTATCGCGATAATTTTTTTCACGATAGTGTATCGATATTCTAACCTCTAGTATCGATACATAATTCAATTAAGTTTTAAACTGATTTACGTTAATATTTAATCAAAACCCTCAGTTTTTATTAGTCTGCTGAGTCATCAATATCATGTGATACACATACTAGAATCTTCCAGGTGGCACACAGCAAGTTGTATTTTTTATTAATATTTAGCTATTTGTTTTACTTTTTTATATTTTTTATTAAGTATTGCAATATGTATTGTATTGCAATATATCATGGTATATTGTATCGTGACCCATGTTTCGTGATATGTAACGTATCGTGAGGCCCTTATCAATACACAGCCCTA

>scaffold_210000293-3

ATACACTATATGGCCAAAAGTTTGTGGACACCTTACCAGCACAGACCCATGAGTATGTCTTCCCCAAACTGTTGCCACAAACTCTGAAACACACAGTCGTCTAGAACGCCTTTGTATGTTGTAGCATTCCATTATTCCTTCACTGGAACTAAGAGACTCAAACCCTGTTCCAGCATGACAATGCCCCTGTGCACAAAGCCCCTGAGCTCCATGAAGACATGCTGTGTTCTGGTTGGTGTAGAAGAACTTGAGTGTCCTGCACAGAACCCTGACTCTGACTCAACCCCACTGAACACCTTTGGGATGAACTGGAACCCCTTCTGGACCTTCTCACCCAACATTAGTGTCTGATCTCACTAATACTATTGTGACTGAATGAACAAAACCACACAGTCACATGACACAATCTAGTGTACAGACTTTCCAGAAGTGTGGAGGTTATAATAGAAGGAAGAGTGAGACTACATCTGGAATAGAATGATCAAAAAAACACATATGGATGTGATGGTCAGGTGTCCCAATACTTCTGACCATATAGTGTAT

>scaffold_210000294-7

AATACAGTGGAACCTTGGCATACAAATTTAATTTAGAGGGGAGTTCTTAAGGCGAAAATCTGTATTGCGATACAAATTTTCCCATAAGAAATAATGTAAAAGCAGATAATCCGTTCCAGCCAACACTATCATATTTTTGCATATAAAAACAATTAAACCATTAAGAAAAGACATGGAAATGAAATAAATAGACCAGCGCCACCAATCTGTCGGCAAAAAAACGTGCGAAAAATCACGTAGCTTTTAAACGCATGCTGAAGGCGACGTCGGTATCTCTTTCAAAACAGCTTTCGCTGACTGAAAAAAAAATCGTAACATTCATAAACTCGCTCCGCTCGGATTTCCACTGAAACAAATGAGTTTTGAACAGCACCCGGACGTATGACGACAGCGAGCCGGCGTTCGGTTCTTACGTATGCTGAAAATGCTTCGTACGCCGACACAAATTTCTTACAAAATTTCAATTCTTAAGGCGAAAATTCATGGGAGGACATTCGTATGCCGAGATTCTACTGTATT

>scaffold_210000294-8

AATACAGTAGAATCTCGGCATACGAATGTCCTCCCATGAATTTTCGCCTTAAGAATTGAAATTTTGTAAGAAATTTGTGTCGGCGTACGAAGCATTTTCAGCATACGTAAGAACCGAACGCCGGCTCGCTGTCGTCATACGTCCGGGTGCTGTTCAAAACTCATTTGTTTCAGTGGAAATCCGAGCGGAGCGAGTTTATGAATGTTACGATTTTTTTTTCAGTCAGCGAAAGCTGTTTTGAAAGAGATACCGACGTCGCCTTCAGCATGCGTTTAAAAGCTACGTGATTTTTCGCACGTTTTTTTGCCGACAGATTGGTGGCGCTGGTCTATTTATTTCATTTCCATGTCTTTTCTTAATGGTTTAATTGTTTTTATATGCAAAAATATGATAGTGTTGGCTGGAACGGATTATCTGCTTTTACATTATTTCTTATGGGAAAATTTGTATCGCAATACAGATTTTCGCCTTAAGAACTCCCCTCTAAATTAAATTTGTATGCCAAGGTTCCACTGTATT

>scaffold_210000294-12

TACAGGGTGGGTGAAAATGAATGGCTATAGAACGTGTAGTATCACTGGAGTCCTGATGAAAACAGTCTGTAAATAGACGATAGTAAATCGGATTTCAAGCCTCGCCCACTGGAGGACTTGGAGGCACGGATTCGGGAGGTTCTCAGCAACATCCCAAACCACTTCCATCAGAAGACATGTGCATTCCATCTCCGGCCGTTTGAGGAAACTCGTTGAATTGGTTCCTACATTGAAATTTAAAGATTTGCTTTCATTTTTCTATGTAATAAAGTACATGCACAATTTGTTTCAATACATTTGTATTAGAAATATAAACTTTATTACCAAATTTTAATGCCTAGTTACTTTTCACCCACCCGGTA

>scaffold_210000294-16

TAGGGCTGCACAATATATCTAAATGATCGAAATATTGAAAATGTGCATATTGCAATGGTTTGCGATAAATGAGCGATTTAATACTTCAAATAGTAACATGTGGTCAAAGTTTTAGGATAGTAAAATATCTGGCAAGCAGAGTTTAGTTGGGATGTGTGTGCGCGTGCTTTGATGCAAGACGATCAACAAGCTGCGAATCTTTATAAGATGAACCAGCCGGAAAAACATCCGCAACTAAAATAGCTAATAAAAATAGCATTTCTCTAGGGTATTGTTATATCGCAAGAAATATCGTTATCGTAATACTCAACATTATCGCATATTTTTCCAGTATCGTGCAGCCCTA

>scaffold_210000294-19

TAGGGCTGCACGATACTGGAAAAATATGCGATAATGTTGAGTATTACGATAACGATATTTCTTGCGATATAACAATACCCTAGAGAAATGCTATTTTTATTAGCTATTTTAGTTGCGGATGTTTTTCCGGCTGGTTCATCTTATAAAGATTCGCAGCTTGTTGATCGTCTTGCATCAAAGCACGCGCACACACATCCCAACTAAACTCTGCTTGCCAGATATTTTACTATCCTAAAACTTTGACCACATGTTACTATTTGAAGTATTAAATCGCTCATTTATCGCAAACCATTGCAATATGCACATTTTCAATATTTCGATCATTTAGATATATTGTGCAGCCCTA

>scaffold_210000296-1

AATACAGTTGAACCTCGGCATACGAATTTGATTCTCTCAGGCGGCGAGGTCTTAAGGCGAAAATTGGTATTGCAATACGAATTTTTACATAAGAAATAATGTAAATGCAGATAATCCGTTCCAACCACCCAAAAATATTACAAATATTACAAATTTCCAACACTATAACCATATTTTTGCATAGAAAAACATTTAGAAAATGAAGTAAAATTTAAAATAAATAGACCCCCGACATCAATCTGTCAAAAAAAAAACGTGTGAAATATCACGTGGCTTTCAAACACATGCCGAAGGCAACGTCGGTATTGTGGTTTGACTCTTTCAAAACAGCTTTCGCTGACTGAAAAACAAATTGTAAAATTCGTAAACTCACTTCAGTTGTTTTCGATTGGCTTTTCACTGACGCAAACAAGTTTTGAACGGCTCCCAGACGTACGCGCAACTTAGCCGACATTCGGTTTGGTTCAGTTTGGTTTGTTGGTATACCGAATATTTTTTGCAAAATTTTAACTCTTAAGGCAAAAATTTGTAAGGGATTGCATTCGTATGCCGAGGTTCCATTGTATT

>scaffold_210000296-6

ATATACTACATGGCCAAAGGTTTCTGGACACCTTACCAGTACACACACTTAAGTGTGTCTTCCACAAACGGTAGCCACAAAATTTGAAACACACAGTTGTCTGTAATGTTTTTGGATGTTGTAGCATTACATTATTCCTTTACTGGAAGTAAGAGACCCAAACCCTGTTCCAGCATGACAATACCCCTGTGCACAAAAAGCCCCTAAGCTTCATGAAGACATGCTGTGTTCTGGTTGGAGTAAAAGAACTCGAGTGTCCTGCACAGAGCCCTGACTCTCACTCAACCCCACTGAACACCTTTGGGATGAACTGGAACACCAACTGCACCCCAAACCTCCTCACCCAACGTTAGTGTCTGATCTCACTAATACTATTATGGCCTAATAAACAAATCCCCACAGTCACACAACACATTCTAGTGGAAAACCTTTCCAGAATTGTGGAGGTTATAATAAGAGTAAGAGTGAGACTAAATTTGGAATAGAATGTTTAAAAAAAAAACACATATGGATGTGATGATCAGGTGTCCCAATACCTTTGGTTATATAGTGTAT

>scaffold_210000296-16

TTAAAGGGGTCATGAATTACCTCAGTTTTTTATTTTGTACTGTTCTCTGCGGTCCACTTATAATGTTATCAAGATTTTTACATGGAAAAAACATCATAATTTAGAAGTAATAGGTGATTTTCTGTCCTGTTTTTGACCCCCCTCATCAGAACGCTCTGCTTGAATAGATTGATGTCTCGGAAGTAAACGCCCACTGCTATGATTGGTAAAAAAAAAACTTTTAGCCAATCGCATGCATGTGCGAATCGGTGGGCGGGGCTAAACAGCCAGTGATGTCGAAGCAGGCGTCGATCTTCTTCTGCGGATGCGGCGCTTATCCACACTATTACATCATAGAGTAGTCGTTTTGGCCGACTGCCTTCAATATAAGCAGATTTTAGAGTAACGACAAAGTTTTGAGTTCTGAAACTTGCAGGATGTTTTTATAGCACAACGACCTCTTATATGTCAAAGGATCAAGGGAATTTTGATTTCTCAGTTTCTCAGACCCCTTTAA

>scaffold_210000297-1

ACTTATGCCGAGTTCACACTGCATGATTTTCAAAGTCGTCAGATCACTGTGGTTTTCACACTGCACTGACTATCTGGGGTAACATTCAGCTGCTGCTGTGTTCACATTGCACGATGGATCAGCCACAAAAGGTTACACACTGCATGACTTTACAATACAAAGTATCGCCAAGAACTCTGTCTGGTACGCACACTATGTTTCACAACCAAACACACATGAGAAGTGATAAGAAAATAACTCAAGATCACGCAAGAGACTGGAAATGTTATTAAAATGTTAGCCAGCAAGAAGTTCACTATACAAATTGTTTGTGCGCTGATTTGCAACGAAAGGGCGGAAAAGGAAAAAAGATTTAAAAAGATCCAAAGCCATGCTTGCTGATATCGTGGTCTATATCTCCTTCCTGAACTTCCCGCTGTCCTGTATCTTGCTCTCTCATTGACCTCTCATGACTGGAGGTCATTTTTCAATCAGAACTCACTTCACACTACACGATTTTGAATCGCCGACAGGTCCAGATATTTAGCATCGTCAGGTCCAGATGTTTAATTTCACACGTGATTGTCACTCGCGTGAACGAGCACCGATTTGCCTCCGAATTCGCATTTGACGGCGATTTCGCACAACCTGTCGGCAAGTGAAAATCAGGGCTGAAATCGTGCAGTGTGAACTCGGCATTAGT

>scaffold_210000299-13

TAGGGCTGTGTTAAAATATCAACACAGCTAACTATCGCGATATTTTTGTTCACGATAGTGTATCGATATTCTAACCTCTAGTATCGATACATGTTTAAATTACATTTGAAACTGATTAATATTTCATTAAAACCCTCAGTGTTTATTTGTCTGCTGAGTAATCAATATAATGTGATACACATACTAGAATCCCCCAGGTGCTACACAGCAAATTTTTTTCTACTAGTTTTCCTTTTTTTTTTTAAGTTTTGCAATATGTATCGTATCGTGATATGTATCGTATCGTGAGGCCCTTGCCAATACACAGCCCTA

>scaffold_210000300-1

CTACACTATATTGCCAAAAGTTTGTAGACACCTCATCAGATCCATATGTGGTTCCTCTTCAAACTGTTGCCACAAATTTAGAAGCACACAGTTGTATAGAATGTCTTTGTATGTTGTAGCATTACATTATTCCTTCACTGTAACTAAGAGATCCAAACACTGTTCCAGCATGACAATGTCCCTGTGCACAAAACCCCTGAGCTCCATTAACACACGGTGTGTTATGATTGGAGTGGAAAAACTGGAGTGTCCTGCACAGAACACCTTTGGGATGAACTGGAACACCGACTGTACCCCAAACCTCCTTACCTGACATTAGTGTTGATCTCACTAATACTATTGTAGCTGAATGAACACAAATCTCACATTCAAGCTCCAAATTGTAGTGGAAATCCTTACTAGAAGAGTGGAGCTTAATCTAACAGCAAAATGGGGAGTAAATTTGGAATAAGATGTTTAAAATCACATATGTTTGTGATGGTCAGCTGTCCACAAACTTTTGCCAATATAGTGTAG

>scaffold_210000300-6

TACACTGTGTGATTTTTAATAGTCTTTTGCAATTGTTGTTTGTCAGACTGTACGAACATGATCCCCATGTCACACTGTAGGATCTCAGTTATCATAATGTACGACAGACTGTACGACAGTCAAGCCACTTCAAAAACAGACGCACACAAGAAGACTCGTCTTGAGTTTTACGTCATCAATCCGTGACATGTTCAGTAACCCTCTCGCTAAACGGTAGCTAGCAGCAAACAAACAGCAGCAACGACACATTATACAAACACTCCTTTTCCCTCCATAACTCAATAAGTTTCTCTTCTTGCTGTTCTGACCACCGGATTCGTTTCCACATTTTCCCGAGGTGGTTTGAAGTTGTCGCGCTAATTGCGTCATCAGGTTTGGCGCTCCTATTGGTTCTTGGTTTGACGGTCATCGTAGGAAAAGTCACACTGCAGGAAAGTGTCTGAAATTTTTTGACACTGCCAGAATTTCATCAGAGGAAAAAACTGATCGCAGCGGCCATTAATCGTCTGTCGGTGAACATGTCAAACTAGCCATCAAAGACAACAGATTTTAGTGTAGGATTATAGGAATCTTTTAGGATTTCAAAATGTGTCTCAGACGACCAAATCGTGGCCAAAATTACACAGTGTA

>scaffold_210000300-9

TAGGGCTGTGTATTGGCAAGGGCCTCACGATACAAAACAGATCACGATACATGGGTCACGATACAATATAACACGATATATTGCAATACAATACATATTGCAATACTTCAAAAATGAAATTTAAAAATGTAAAACAAATAGCTAAAAATACAACTTGCTGTGTAGCACCTGGGGGATTCTAGTATGTGTATCACATTATATTCATTACTCAGCAGACAAATAAACACTGAGGGTTTTAATTAACTATAAACGTAAATCAGTTTAAAATGTAATTTAAATATGTATCGATACTAGAGGTTAGAATATCGATACACTATCGTGAAAAAAAATATTGCGATAGTTAGCTGTATCGATATTTTTACACAGCCCTA

>scaffold_210000301-1

TACACTATATGGCCAAAAGTTTGTGGTCACCTTACCAGCACATCTACAAGTGTGTCTTCCCCAAACTGTTTGAGGCACACGGTTGTCTAGAACGTCTTTGTATGTTGTAGCGTTACGTTATTCCTTCACTGGAACTAAGAGGAACAAACCCTGTTCCAGCATGACAATGTCCCTGTGCACAAAACCCCTGAGCTCCATGAAGACATGCTGTGTTCTGGTTGGAGTGGAAGAACTCTCATGTCCTGCACAGAGCCCTGACTCTGACTCAACCCCACTGAACACCTTTGGGATGAACTGGAACACCGACTGCACCCCAGACCTCCTCACCCAACACCTCATTAGTGTCTGATTAGGATCAGAATGAACAAATCCCGACAGCCACGCTCCACAATCTAGTAAAAAGACTTTCCAGAAGTGTGGAGGTTATTATAAGAGTAAGGGTGAGACTAAATCTGGAATAGGATGTTCAAAAACACATATATGGATGTGATGGTCAGGTGACCCAATACTTTTGTCCATATAGTGTA

>scaffold_210000301-4

AGGCTACGTTCACACTGCATGCAAATGCGGCCAAAATCAGATTTTTTTTCCCCCACATGTGACTCAGATCTGTTTTTCTCATGACAGTGTGAACAGCACAAACCGCATGGAATCTGATCTTTTCAATTCCGATTTGTGCCACTTCCATATATGGCATTAAATCCGACACAGTCCAGATGTTTTGCAATGCGACCGCAGTGTGAACAGTTATGTCGGAATTCATGAGACTTTTATGCGACATTTTCGATGGACATGCGTCATCATTCTGCACTGGCGCGTAACTACAAACAGTTGATCTATAATCGATTTGGCTGAAGGCGTTGATCACGTCACGGTCCCACCACTCCTGACTCCGGCTCTGCATCCACACACACCTCCATACTGAAGTAGCAGCCACTGCTCCACAACATGCTATTGTTTAAAATTTGGCCCTTTTTCTCCTCATCCTCGTCCTCCTCAATCACTGCTCATTAATTCACTATCGTCCGCTGCACATCACCTTATAAATGTTAGCAGCTAACGTGTAAACGCTGCCTTCAGTGTCTCCCGTGTTTATTTACTCGTATGACAGGACGCTGCGTGAGACGTAGAATTAGTTCTTTTGCGCATGCGGGTCAGTTAAGGACCATGATCAGTTCACACTGCAAATCTGATATTGGCCACATTTAAAACAACAATGTGAACAGCTATACAAAAAAAATCGGATCTCAACAAAAATCGGAATTGAGCACTAAAGCCTCGCAGTGTGAACGTAGCCT

>scaffold_210000301-9

AGTAGGGCTGCACGATACTGGAAAAATATGCGATATTGTTGAGTATTGCGATAACGATATTTATTGCGATATTACATTTCCCTAGAGCTAATGCTATTTTTATTAGCTAGTTTAGCTGCGGATGTTATTCGGGCTGGTTCATCTGATCAGGATTAACTCGCTATGAAAGCGTCGCAGCTTGTCGATTGTCTTGCGTCAACGCACGCGCACGCAAATCCCAACTAACATCCGCGTACCAGATATTTTACCATCCTAAAACTTTGACCACACATTAATATTTGAAGTATTTATAATACGCTCATTTATCGCAAACTATTGCAATAGGCACATCTCGATATTTTGATAATTTCGATATATTGTGCAGCCCTACT

>scaffold_210000303-1

ATACACTATATGGCCAAAAAGTTGTGGGCACCTTACCAGCACACCTATAAGTGTGTCTAGTTACAAAGTCGGAAGTACGCAGCTGTCTCAAGTGTTTCAATATGCTGTAAAATTAAGATTTCCATTCACTAGATCTTAGAGACTTAAACACTGTTCCAGCATGGCAATGCCCCTGTGCACAAAGCTCCTGAGCTCCATGAAGACATGCTGTGTTCTGGTTGGAGTGGAAGAACTCTCATGTCCTGCACAGAGCCCTGATTCTGACTCAACCCCACTGAACACCTTTAGGATGAACTAGAACACCGACTGCACCCCAGACCTCCTTACCTGACGTTAGTGTCTGATCTCACTAATACTATTGTAGATAAATGAACACAAATCCCCACAGTCACATGACACAATCGAGTAAAAAACCTTTCCAGAAGTGTAGAGGTTTATAGGAGGAAGAGTGAGACTAAATCTGGAATAGAATGGTCGAAAAACACATATGGATGTGATGGTCAGGTGTCCCAATACTTTTGGCCATATAGTGTAT

>scaffold_210000303-7

TACACTACATGTACAAAAGTGTTGGGACACCTTATCAAAACATACTATTAGAGATGTAGTCTCATTCTTCTTCCTATAATAACCTCCACACTTCTGGAAAAGATTTACATTAGAGTGTGACTGTGGGGATTTGTGTTCATTCAGCTACAATAGTGTTAGTGAGATCAGACACTAATGTTGGGGGAGGAGGTCTGGGGTGCAGTCGGTGTTCCAGTTCATCCCAAAGGTGTTCAGTGGGGTTGAGTCAGAGTCAGGGCTCCGTGCACTCGAGTTCTTCCACTCCAATCATAACACAGCATGTCTTCATGAAGCTCAGGGGTTTTGTGCACAGGGACATTGTCATGCTGGAACAGGGTTTGTTCCTTTTAGTTCCAGTGAAGGAATAATGTAATGATACAACATACAAAGACATTCTAGACCACTGTGTTCTTCAGACTTTGCTGCAACAGTTAGGAAAAGACACATTTAGGTGGGTGTGCTGGTGAGGTGTCCACAAACTTTTGGCCACATAGTGTA

>scaffold_210000303-14

CTAGGGATGCACCAGTACCAGTATCAGTATCAAGTACGATACTGACCTCATGTACTCGTACTCATAAAAACACTTTGATATCAAAGATCGATACCTCTTGTGACGTCATTGACAGAACTTTCAGTGTACAAAGAATCCGGCAGCAGCAACAGAATGTCCGACTCAGCGGTGTGGAAATATTTCAAAATGAATGACGACAACAACCCACGCATGGCAGACTGCAAACTTTGTTCCGCAAAAATATCAAGAGGTACAAAAATCAGTGCTTATAATTACGAGTAATCTGATAAAACATCTGAAATTAAAACACAAAAGTGAGCACGGAGAATTTGCCGCTAGCAGTAACGTTAGCACTCGGCAACCAACCCTGCAGCAAAGGTAGCAAGAGAAAACCTGAGAGCCATACAAATAATCCCACATCCCAAAAATGTACAGGTATCGGTATCAGAGAGTACCAGGAAAAAAATATCAGTACTCGTAATCAGTCTTTAAAAAATGGTATCAGTGCATCCCTAG

>scaffold_210000304-2

TACACTATATAGACAAAAGTATTGGGACACCTGACCATCACATCTATGTGCTTTTTGAACATCCTATTCCAGGTGTAGTCTCACTATTCCTCCTATTATCACCTCTACACTTCTGGAAAGGTTGTCCACCAGATTGTGTCATGTGGGGATTTGTGTTCATTTATCTGCAATAGTATTAGTGAGATCAGACACTAATGTTGGGGGAGGAGGTCTGGGGTGCAGTCGGTGTTCCAGTTCATCCTAAAGGTGTTCAGTGGGGTCGAGTCAGAGTCAGGGCTCCGTGCAGGACATGAGAGTTCTTCCACTCCAACCAGAACACAGCATGTCTTCATGGAGCTCAGGGGCTTTGTGCACAGGGGCATTGCCATGGAATAATGTAAAGCTACTACATATAGACACATACTAGACAACTGTGTGCTTCAGAGTTTGTGGTAACAAACACTAAGACACTCTAATGTGGGTGTGCTGGTAAGGTGTCCACAAACTTTTGGCAATATAGTGTA

>scaffold_210000304-3

TACACTATATTGCCAAACGTTTGTGGACACCTCACCATCAGGTTCCTTTCCAAAATGTTGCTACAAATTTAAAAGCACACAGTTGTTGTCTAGAATGTCTTTGTATGTAATATTACATTATTCCTTCACTGGAACTAACAGACCCAAACACTGTTCCAGCATGACAATGTCCCTGTGCACAAAACCCCTGAGCTCCATTAAGACATGTTATGATTGGAGTGGAGTTTCTGCACAAAGCCCTGACTTTAACACCACTGAACACGTTTAGGGTGAACTGGAACACCGACTGCACCACAGACCTCCTTACCTGACGTTAGTGTCTGATCTCACTAATCCTATTGTAGCTGAATGAACACAAATCTCACATTCAAGCTCCAAATTGTAGTAAAAAGCCTTACTAGAAGAGTGGAGCTTAATCGAACAGCAAAATGTAGAGTAAATCTAGAATAAGATGTTTAAAATCACATCTGTTTGTGATGTCAGGTGTCCACAAACTTTTGCCAATCTAGTGTA

>scaffold_210000304-4

TTAAAGGTAGTGTAGGTGATTTGGTTTAATAACAGTTTTTGTTCTGCTGGTTGAAAGTCTCTTCACATCTCGATAGCACTCATTACGTTAAGTGGTCTAAATGTATTTATAAGTATTTCTATCGTCTGTGGAAGGCGTAGTACCAAAAAACCTTCGTCCAATCAAAACGTTCGGTCCAAACATTACGATAGGTTATCCTACCTGCTCGTCAAATATGTATTTGCATACCTCTGCGCACCCTGTTCGCGCAGACAGATTACGTCATTAGCGCGTTCCCGCTATGCAGACTGAGTAAAAATGGAAGGCAAACAGCAAGAGACCAACATTGCCTCTAAAAGTGGCACAGACACCCGCATGAGAAAAACAGTGTTAGAAAGAGCGATGTCTTAAAAAGTTTTAGATTAGCAAAGAGCTAAATGTCGAGTTAATCTAGGTGTACATCCGGTCCTGCCTGTGCGCGGTCATGTGTTTTGGAGGAGGCGTGGCTTTACAGAGTGATTATGCAGGGAGGGCGGGATCTTTGGTTTTCAACGCTAGCTTGCTATTGCTAGCCTCTCCGAAATCGCCTACCCTACCTTTAA

>scaffold_210000304-7

TTAGGGCTGCACGATACTGGGATTATATGCGATATTGTTGTCGAGTATTGCGATAACGATATTTATTGCGATATAATATTTCCCTAGAGAAATGCTATTTTTATTAGCTAGTTTAGCTGCGGATGTTATTCGGGCTGGTTCATCTTATCAGGATTAACTCGCTATGAAAGCTTCGCAGTTTGTCGATCGTCTTGCGTCAAATCACGCGCACGCAAATCCCAACTAACATCCGCGCGCCAGATATTTTACCATCCTAAAACTTTGACCACACGTTAATATTTGAAGTATTAAATCGCTCATTTATCGCAAACTATTGCAATACGCACATCTCGATATGCCCGTTTGCAATATTTCAATCATTTCGATATATTGTGCAGCCCTAA

>scaffold_210000304-19

TACAGTGGAATCTCGGCATATGAATGCCCCTCCCTTACAAAGTTTCACCTGAAGAATTGCAATTTTTGCAAGAAATTTGCATTGGCATAAAGTATTTTCAGCATACGAACGTCCCGAACCGAACCGAACGCCGGCTAAGTTGTTAAGTTCTCAAAACTTGTTCGCGTCAGTGGAAAGCCAAGCAAAGCGAGTTTACGAATTTTTTTTCACTCAGTGAAAGCTGTTTCGAAAGAGTCGACCCATGATCCCGACGTTGCCTTTGGCGTGCGCTCAAAAGCTACGTGATTTTTTGCATTTTTTTTTCCGTTGGCAGATTGGTGGCGTGGGTCTGTTTATTTCATGTTTTACTTCATTTACATGTCTTTTCTAAATGTTTTGATTGTGTTGGAAACTGGTAATATTGGTAATATTTTTGGGTGGCCGGAACGGATTATCCGCATTTACATAATTTTTTATGGGAAAATTCATATTGCGATATACTAAATTCTTGCCTTAAAAACTTGCATCCAGAACGAATTAGATTTGTATGCTGATGTTCCACTGTA

>scaffold_210000305-3

TATATCCTCCTGAGACCCCGCCCATTGACTTGTGTCCTCTGTAGTGGACATTTTGTTTTCATAAATGTTCTTTCCAATTTTTTTAACTACTCATCAATTTCTGCTGTACTGTACAGATGACATCCTGGGCTTTCTAGTGATACGTCAATTGATTGGCTGGGATGCTAGGAACTAGTTTTTACACTGCATCCAAAATGGCTGCCATAGGAACAAGCACATTTTATGGAAAGGACTGACTTTTCCAACTTAAATGGTCCTGTGATCCACTACAGTGGACATGCTGTAAAATTAAAAATAGCAATAAAATTTAAAAACTCTACATCGTAGAATGTTTTGTAACCCGAAAAAGGTAGGAAATCACACAAAACAAAGAAATTGATGACACTTTTTCCTTGGGTCTCAGGAGGATATA

>scaffold_210000305-13

TAGGGCTGCACAATATATTGAAATTATCAAAATATCACAAATGGGCATATCGAGATTTATGGTTTGTGATAAATGAGTGATTTAATACTTTTTAATATTAATGTGTGGTCAATGTTTTAGGATAGTAAAATATCTGGCGCGCGGATGTTAGTTGGGATTTGCGTGAGCGTGCTTTGACGAAAAACAATCAACAAACTGCGAAGCTTTCATAGCGAGTTAATCCTGATAAGATGAACCAGTCCGGAAAACTCCCGCAGATCAACTAGCTAATAAAAATAGCATTTCTCTAGGGAAATATTATATCGCAAGAAATATCGTTATCGCAATACTCAACAACAATATCGCACATAATCCCAGTATCGTGCAGCCCTA

>scaffold_210000306-3

TTATTTAAAGGGGTCATGAACTGAGAAATCAAAATTCTTTTGACATATAAGAGGTTGTTGTGCTATAAAAACATCAAACTTTGTCGTTACTCTAAAAACAGCTTATATTGAAGGCAATCGGCCAAAACTACAGGATTTGGAATGTTCTACTCTATGACGTAATAGTGTGGATAAGCGCCGCCTCTCCAGAAGACCCTACACCTGCTTCCTACAGTCTATTTAGCCCGTGATAGGTAAATGACAAGAGAGACATAAAACTGTTAGCCAATCATAGCAGTGGGCGTTTAATTTCCGAGACAACGATCCGCCACGCCTATTCAAACAGCGTTCTGATGAGGGGGTTGCCTATTCTATATATAAATTGCCTATTCTAAATGATGATGTTTTTTGATGTAAAAAAAACTTGATAACATTATAAGTGGACCTCAGAGAACAGTACAAAATAAAAAACTGAGGTAGTTCATGACCCCTTTAATAA

>scaffold_210000307-1

CTAAAGCCGGGTTTACACCGTGCAATTTTTAATAGTCTTTTGCAACTGTTGCTCGTCAGACTGTACGAACATGATCCCCATGTCACACTGTAGGATCTCCGTTATCATAATATCAGACCGTACGACAATCAAGACGCGTCAAAAACGGACACACAAGAAAACTCGTCAGGAGTTTTACATCAACAATCCGTGACACGTTCAGTAACCCGCGTTCTCTCGCTAAATGGTAGCCAACAGCAAACAAACAGCAGCGACGACACGTCATACAAACACTTCTTTTCCCTCCATAACTCAATAAGTTTCTCTTCTTGCTGCACTGACTCATTTCCACATTTCCCCGAGGTGGTCTGAAGTCGTCGCGCTAATTGCGTCATCAGGTTCGGCGCTGCTATTGGTTCTTGGTTTGACGGTCGTTGTAGAAGTCACACTGCAGGAAAGTGTCTATTCCCCCATTGGAGGAAAAAACTGATCATAACAGTCAATAATCGTCTGTCGGTGAACATGTCAAACTAGCAAGCAAATACAACAGATTTTAGTGTAGGATTATGGGAATATTTCAGGGTTTCAAAATTTGTCTCAGACGACCAAATCGTGACCAAAATCACACAGTGTAAACCCGGCTTTAG

>scaffold_210000308-1

TATTGCCAAAAGTTTGTGGACACCTGACCATCACAAACATACATGATTTTCAACATCTTATTCCAGATTTACTCCCATTTTGCTGTTAGATTAAGCTCCACTCTTCTACTAATGCTTTTTACTACAATGTGAGATTTGTGTTCATTCAGCTACAATAGTATTAGTGAGATCAGGCACTAACATCAGGTAAGGAGGTTTTTGGTGCAGTCGGTGTTCCAGTTCATCCTAAACGTGTTCAGTGGTGTTAAAGTCAGGGCTTTGTGCAGAACAAGTTTTTTCCACTCCAATCAGAACACACCATGTCTTAATAGAACTCAGGGGTTTTGTGCACAGGGGCATTGTCATGCTGGAACAGTGTTTGGGTCTCTTAGTTACAGTGAAGGAATAATGTAATGCTACAACAAAGACATTCCAGACAACTGTGTGCTTCCAAATTTGTAGCAACATTTCAGAGAGGAACCACATATGGATCTGATGGTCAGGTGTCCACAAACTTTTGGTAATA

>scaffold_210000309-1

TACACTATTTTGCCAAAAGTTTGTGGACACCTGACATCACAAACATATGTGGTTTTAAACATCTTTTTCCAGATTTGCTCCTCATTTTGCTGTTCGATTAAGCTCCACTCCTCTAGTAAGGCATTTTACTACAATTTGCAGCTTGAATGTGAGATTTGTGTTCATTCAGCTACAATAGTATTAGTGAGATCAGACACTAACGTCAGGTAAGGAGGTTTTTGGTGCAGTCGGTGTTCCAGTTCATCCCAAACGTGTTCAGTGGTGTTAAAGTCAGGGCTTTGTGCAGAACACTCAAGTTTTTCTACTCCAATCAGAACACACCATGTCTTAATGGAGCTCAGGGGTTTTGTGCACAGGGGCATTGTCATGCTGGAACAGTGTTTGGGTCTCTTAGTTACAGTGAAGGAATAATGTAATGCTATAACATACAAAGACATTCTAGACAACTGTGTGCTTCTAAATTTGTGGCAACATTTTGGAGCGGAACCACACATGGATCTGATGGTGAGGTGTCCACAAACTTTTGGCAATATAGTGTA

>scaffold_210000309-2

TATTGCCAAAAGTTTGTGGACACCTCACCATCAGATCCATATGTGGTTCCGCTCCAAAATGTTGCTACAACTTTAGAAGCACACAGTTGTCTAGAATGTCTTTGTATGTTATAGCATTACATTATTCCTTCACTGTAACTAAGAGACCCAAACACTGTTCCAGCATGACAATGCCCCTGTGCACAAAACCCCTGAGCTCCATTAAGACATGGTGTGTTCTGATTGGAGTAGAAAAACTTGAGTGTTCTGCACAAAGCCCTGACTTTAACACCACTGAACACGTTTGGGATGAACTGGAACTGGAACTGGAACTGCACCAAAAACCTCCTTACCTGACGTTAGTGTCTGATCTCACTAATACTATTGTAGCTGAATGAACACAAATCTCACATTCAAGCTCCAAATTGTAGTAGAAATTCTCTAGAGGAGTGGAGCTTAATCGAACAGCAAAATGGGGATCAAATCTGGAATAAGATGTTTAAAACCACATATGTTTGTGATGTCAGGTGTCCACAAACTTTTGGCAATA

>scaffold_210000309-4

CTATATTGCCAAAAGTTTGTGGGCACCTCACCATCAGATCCATATGTGGTTCCTCTCCAAAATGTTGCTACAACTTTAGAAGCGCACAGTTGTCTAGAATGTCTTTGTATGTTATAGCATTACATTATTCCTTCACTGTAACTAAGAGACCCAAACACTGTTCCAGCATGACAATGCCCCTGTGCACAAAACCCCTGAGCTCCATTAAGACATGGTGTGTTCTGATTGGAGTAGAAAAACTTGAGTGTTCTGCACAAAGCCCTGACTTTAACACCACTGAACACGTTTAGGATGAACTGGAACACCGACTGCACCAAAAACCTCCTTACCTGACGTTAGTGCCTGATCTCACTAATACTATTGTAGCTAAATGAACACAAATCTCACATTCAAGCTGCAAATTGTAGTAGAAATTCTCTAGAGGAGTGGAGCTTAATCGAACAGCAAAATGAAGAGCAAATCTGGAATAAGATGTTTAAAATCACATTTGTTTGTGATTGTCAGGTGTCCACAAACGTTTGGCAATATAG

>scaffold_210000309-9

TAGCTATGTTTCCATCCAAAGATGCGAATTAAACTTATGCACAAAACTGGGATATTGCATAAAGCATTTGAGAATAAAGCCCCGTTTCCATCCAAGAAGTCAAGGAGTACAAAATCATCACTTCCTGATAAACTGGCGCCAAATATCAAAAAGAAAAATGGAAGTTGCTGCGGTAGGAGAAGCCACTGTGGGCCTTTTTCCGTATATAATAAATTACTTGCGTCTCAGAACACGTAGACGAAACGCAATGAACGCAGTCGTGTCCTCTTGCTTTCGGAGGCGGGAGACTGGGAACGCAGCTGGTCAACACAGTTCTGGGAGGAGGTAATAATACTGAACCACTTTGATGACAGACTTTGGCTACGGCATTTTAGAATAACCAAAACAACATTTCAGATGTGCAATAAGATCGGTCCGCTGGTTAGTCCATTAATGACGTCCCATCGCGCCCCGTTTCCCACAGAAAAACCCATTGCTATTGCGCTGTATAAATTGGCCATTTTTGTTATCACATGATATGTTGAAGCAAAATCACGACTTTTTTGATGCGCATGCTGAAATTTACTTGGTAAAATGTCTTACCGTCGTAGTTTCTACGCATTTTTTCCTTATCGGCAAAAAAGTTTATCCGACTCAGTTGTGCGTATAATTTTTTCATGCGCATTTTCATAATTTATGCGCATCTTGGCGTTTCCATCCATGTTTTTTTTAAATATGTGATAGTCCAAAATGCGCATAATAATAGGTGGATGGAAACATGGCTA

>scaffold_210000309-10

TACGGGGTGGGTGAAAATTAACTAGGCAATATTTAATGGTTCTAGAACTTGTAGTATCACTGACAACAGTCTCTAAATAGACGATAGTAAATAGGATTTCAAGCCTCGCACACTGGAGTCGGGAGGTTCTCCGCGACTTTCTTCAAAATTTCCCTCTCTGGCCGCTTGAGCAAACTGGTGGACTCCACCTACGTTGAAATTTAAAGATTTGCTTTCATTTTCCTATAAAATAAAGTACAATTTGTTTCAATAAATTTGTATTAGAAATATGGACTTTATTTTTAATGCCTAATTACTTTCACCCACCCTGTA

>scaffold_210000310-1

CAGGGCTGGGTAACCCATGTTCCTTAAGTTCATTTGCTCCGCTTGTTTTCCAGCTCCCCCTTCCCTACCCACTGCTGATCACCTGGTTCAGGTGTGTTCAGTCAATCAGAAAGTAGGAGATACCAAATCACCAGGTCTTCCACCACTCCACCCTCATCTGAAATAGTATCTTCCAGCTTCTGATTGACTGAACACACCTGAACCAGGTAATCAGCAGTGGGTAGTGCAGGGGGAGCTGGAAAACAAGCGGAGCAAATGAACTTAAGGAACATGGGTTACCCAGCCCTG

>scaffold_210000310-7

TAGGGATGCACCAATACTGATACTGTTATCGGTATCGGGCCCAATACTGAGCTAATGTACTTGTACTCATAAAAACACTCCAATACCAAAGACCGATACCTCTCTGTTGACATAATTGGTGGAACTTTCAGTGCGCAAAGACTCCGGCAGCAGCGTCAGAATGTCAGACTCAGCGGTGTGGAAATATTTTAAAATTAATGATGACAACCCACCCACGGCAGACTTTGTTCAGCAAAAATATCAAGAGGTTCAAAATTAGTATTTATAATCCGAGTAATCTGATAAAACATCTGAAATTTAAACACAAAAGTGAGCGCGCAGAATTGGACGCTATCAGTAATGTTAGCACTCGGCAACCAACCCTGCAGCAAACTCTTGAAAGACGAGAGAAAATGGCAAGAGAAAACCCGAGAGCCATACAAATAATCCCACATCCCAAAATAGTACAGGTATTGGCGAGTACCAGGAAAAAGATATCGGTACTCGTACTCCGTCTTTAAAAAATGGAATCGGTGCATCCCTA

>scaffold_210000310-15

TAGGGCTGCACGATACTGGGAAAATATGCGATATTGTTGAGTATTGCGATAACGATATTTCTGGCGATATAACATTTCCCTAGAGAAATGCTAATTGTATTAGCTATTTTAGCTGTGGGTGTTTTTCAGGCTGGTTCATCTCATCAAGTTTAACTCGCTATGAAAGCTTTGCAGTTTGTTGATTGTCTTGCGTCAAAGCACGCGCACCAGATCCCAACTAACATCCGCGTACCAGATATTTTACTATCCTAAAACTTTGACCACACATTACTATTTAAAGTATTAAATTTATCGCAAACCATTGCAATACGCATATCTCGATATGCACATTTGCGATATATTTTGTGCAGCCCTA

>scaffold_210000312-14

TAGGGCTGCACAATATATCGAAATTATCAAAATATTGCAAATGTGCATATCGAGATATGCGTATTGCAATGGTTTGCGATAAATGTGCGATTTAATACTTTAAATAGTAATGTGTGGTCAAAGTTTTAGGATAGTAAAATATATGGCACGCGGATGTTAGTTGGGATATGTGTGCACGTGCTTAGACGCAAGACAATCAACAAACTGCAAAGCTTTCATAGCGAGCTAAACTTGATAAGATGAACCAGCCAGAAAAACACCCGCAGCTATAAAATAGCATTTCTCTAGGGAAATGTTATATCGCAAGAAATATCGCAATACTCAACAACAATATCGCATATCGCATATTTTCCCAGTATCGTGCAGCCCTA

>scaffold_210000312-28

TTAGGGATGCAACGATACCATTTTTTAAAGACCGAGTACAAGTACCAATATTTTTTTCCTGGTACTCACCAATACCGATACCTGTACTATTTTGGGATGTGGGATTATTTGTATGGTTCTCGGGTTATCTCTCTCTTATTCTCTCTTCTTCCAAGAGTTTGCTGCAGGGTTGGTTGCCAAGTGCTAACATTACTGCTAGTGGCAAATTCTCCATGCTCACTTTTGTGTTTCACTTTTTAATATTTTATCAGATTACTTGTATTATAAGTACTCATTTTTGTACCTCTTGATATTTTTGCGGAATAAAGTTTGCAGTCTGCCGTGGGTGGGTTGTCATCATTCATTTTTAAATATTTCCACACAGCTGAGTCTGACATTCTGACGCTGCTGCCGGAGTCTTTGTGCACTGAAAGTTCTGTCAGTGACGTCACAAAAGCTATCGGTCTTTGGTATCGGAGTGTTTTTACGAGCTCAGTATCGGGCCTGATATCGATACTGGTGCATCCCTAA

>scaffold_210000313-8

TTAGGGCTGCACAATACTGGGAAAATGTGCAATAAGCAATATTGTTGTTGAGTATTGCGATAACGATATTTCTTGCGCTGTAACATTTCCCTAGAGAAATGCTATTTTTATTAGCTATTTTAGCTGCAGGATTTTTTCGGGCTGGTTCATCTTATCAGGATTAACTCGCTATGAAAGCTTCGCAGCTTGTTGAATGTCTTGCGTCAAAGCACGCGCACGCAAATCCCAACTAACATCCGCGTGCCAGATATTTTACTATCCTAAAACTTTGAACACACATTACTATTTGAAGTATTAAATCGCTCATTAATCGCAAACCATTGCGATATACATATCTCGATATGCACATTTGCAATATTTCGATAATGTCGTTATATCGTGCAGCCCTAA

>scaffold_210000314-2

AATATACACTATATTGCCAAAAGTTTGTGGACACCTCACCATCAGATCCATGTGTGGTTCATCTCTAAAATGTTGCAACAAATTTGAAAGAACACAGTTGTCTAGAATGTCTTTGTATGTTGTAGCATTACATTATTCCTTCACTGGAACTAAAAGACCCAAACACTGTTCCAGCATGACAATACCCCTGTGCACAAAACCCCTGAGCTCCATTAAGACATGCTGTGTTCTGATTGGAGTGGAAAAACTTGAGTGTTCTGCACAAAGCCCTGACTTTAACACCACTGAACACGTTTAGGATGAACTGGAACACCGACTGCACCCCAGACCTCCTTACCTGACGTTAGTGTCTGATCTCACTAATACTATTGTAGCTGAATGAACACAAATCTCACATTCAAGCTGCAAATTGTAGTAAAAAGTCTTACTAGAGGAGTGGAGCTTAATCGAACAGCAAAATGGGGAGTAAATCTGGAATAAGATGTTTAAAATCACACATGTTTGTGAAGGTCAGGTGTCCACAAACTTTTGGCAATATAGTGTGTATT

>scaffold_210000315-1

ACTTAAGCTGGGTTTACACTGTGCCATTTTTAATAATCCTCTGCGATTGTTGATTGTCAGACTGTACGAACATGATCCCCATGTCACACTGTAGGATCTCAGTTGCCATAATGTCAGACTGCACGACAGTCAAAAACGGACGCACACAAGAAGACTAGTCCGGAGTTTTACATCAACAATCCATGACACGTTCAGTAACGCGCGTTTTCTATTAAATGGTAGCTAGCAGCAAACACAAACATTCTGTAGCTGTAATTTTGCTCATGACAAAACTCAATAAGTTCCTCTTCTTGCTGCACTGTCCACCGGACTCGTTTCCACATTTACCTGAGGTGGTTTGAAGTTGCCTGCGTTAATTGCGTCATCAGGTTCGGCGCTACTATTGGTTCTTGGTTTGACGGTCGTCGTAGGAGAAGTCACACCGCAGGAAAGTGTCACTGCCAGAACTTCAGTGGAGGAAAAAACTGATCGTAACGGTCATTAATCGTCTGTCGGTGAACATGTCAAACTAGCCATCAAAGACAACAGATTTTAGAGTAGGATTATAGGAATCTTTCAGGATTTCAACATTTGTCTCAGACGAACAAATCGTGGCCAAAAATCACACAGTGTAAACCCGGCTTTAGT

>scaffold_210000317-2

ACTAAAGCTGGGTTTACACTGTGTGATTTTGGCCACGATTTAGTCGCCTGAGACAAATTTGGAAATCCTAAACGTTTCCTATAATCCTACGCTAAAATCTGTAGTCTTTGATGGCTAGTTTGACGTGTTCACCGACAGCCGATTAATGACCGTTATGATCGTTTTTTTCCTCTGATGAAATTTCGGCAGTTTCAGAAGATTTTAGACTCTTTTCTGTAGTGTGACGTCTCCTAAGACGACCGTCAAACCAAGAACCAATAGGAGCGTTGAACCTGATGACGCAATTAGCGGGACAACTCCAAACCACCTTGAGAAAATATCAAAACGAGTCCGGTGGTCAGTACAGAAAGAAGAGAAACGTATTGAGTTATGGAAGGAAAAGGAGCGTTTGTATGACGTGTCGTCGCTGCTGTTTGTTTGCAGCTAGCTACCATTTAGCGAGAGAACGCGAGTTACTGAACGTGTCACGGATTGATGATGTAAAACTCCGGACGAGTCTTCTTCTGTGTGTCCGTTTTTGACGCGTCTTGACTGTTGTCTGCAGTCTGACATTATGACAACTCAGATCCTACAGAGTTACATGGGGAACATGTTCGTACAGTCTGACAAGGAACAATCCCAAAGAACTATTAAAAATCACACAGTGTAAACCCGGCTTTAGT

>scaffold_210000318-25

CTAGGGCTGCACGATATATATCGAAATTATCGAAATATCGCAAATGTGCATATCGCAACGGTTTGCGATAAATGAGCGATTTAATACTTCAAATAGTAATGTATAGTCAAAGTTTTAGGTCGATGCGGATAGTTAAATATCTGGCACGTGGATGTTAGTTGGGATTTGAGTGCGCGTGCTTTGACGCAAGACAATCAACAAGCTGCAAAGCTTTCATAGCGAGCTAATCTTGATAAGATGAACCAGCCCGAATAACACCCGCAGCTGCCACCGAAGATTTAGTGCCAAAACGAAGAAACACCTCAAAGATATGGCAGTATTTCAGTTTTAAAAGACATGCAGACATACCGACACATGCACATGTAAATCTCAAGTGGCAACGCAAAGGGAAACACTACAAATCTTCACAGTCACCTCAAAATGAAGCGTAACATTTTGTATCAGCTGTGCTCGACTGAAAAATCTTCAAACGTGAATGTGTTTGAAGAAACATATTTCGGTTATGTCATTTTTAAAAATATCGCATATTGCGTTATTTAGCATGCTATTGCATATCGCATTTTTCTTCAATATCGCACAGCCCTAG

>scaffold_210000322-5

TTAAAGGCAGGGTAGGTGATTAGGTTCAAAAATTGTTTTGTTATGCTGGTTGAAAGTCTCTTCACATCTTGACAGCACTTAAGTTAAGTGGTCTAAATGTATTTATATCGTCTGTGGAAGGCGCAGGACCAAAAAAACTTCGTCCAATCAAAATTTTCGGTCCGAACATTACGATAGGCTATCCTACCTGCCTGTCAAATATGTATTAGCAAACCTCTGCACACCTGTTCGTGCAGACAGAACATGTCATTAGCGCATTCCCGCTATGCAGACTGAGCGAAAATGGAGGGCAAACAGCAAGAAAAAAAAAGTTGCTTATGTTCGTTATTACTGTAATATTATGCACTTTGTACTGTAATGTTTACTCCCTGATGAATCAGACATGAGGTAATCTGAATCTCGTCTGTACGTTTGAGGCTCCGGTCTGCCTGTGCACGTTCATGTGTTTTGGACGAGGTGTGGCTTTGCAGGGAGTGATTATGCAGGGAGGGCAGGATTTTTAGTGTTTAATGCTAGTTTGCTATTGCTAGCCTCTCCGAAATCACCAACCCTACCTTTAA

>scaffold_210000322-9

TAGAGATGCATCGATACCGATACCGGCATTGGTATAAGGCCCGATACTGAGCTAATGTACTCGTACTAACACTCCGATGCCAAAGACCGATACCTCTTGTGAGGTCATCGACAGATCTTTAAGTGTACAAAGACAGCGACAGAATGTCAGGCTCAGCGGCGTGAAAATATTTCACAATTAATGAGGACAACCCACGCATGGCAGACTGCAAACTGTGTTCCGCAAAAATATCAAGAGTACAAAAATGAGTACTTATAATATGAGTAATCTGATAAAACATCTCAAATTAAAACACAAAAGTGGGCACGGAGAATTTGCCCAACCCTGCAGCAAAACGAGAAAAATGGCAAGAGATAACCCGAGAGCCATACAAATAATCCCACATCCCCAAAAAGTACAGGTATCGGTAGTATCGGCGAGTACCAGGAAAAAAATATCGATATTCGTACTCAGCCTTTAAAAAAATGGTATCGGTGCATCCCTA

>scaffold_210000323-2

CACTAGATTGCCAAAGGTTTGTGGACACCTGACCATCACGCAAACGTGATTTTAAAAATCTTATTTCGGATTTACTCCCCATTTTGCTGTTACATTAAGCTCCACTCTTCTAGTAAGGATTTCCACTACAATGTGGAGCTTGAATGTGAGAAATGTGTTTATTCAGCTACAATAGTATTGGTGAGATCAGGCACTAACGTCGGGTAAGGAGGTCTGTGGTGCAGTCGGTGTTCCAGTTCATCCCAAAGGGGTTCAGTGGTGTTAAAGTCAGGGCTTTGTGCAGAACACTCAAGTTTTTCCACTCCAGTCATAACACACCATGTCTACATGGAGCTGAGGGGCTTTGTACACAGGTGCATTGTCATGCTGGAACAGTGTTTGGGTCTCATAGTTCCAGTGAAGGAATAATGTAATGCTACAACATACAAAGACTTTCTAGACAACTGTGTGCTTCCAAATTTGTAGCAACATTTTGGTGAATCTGACGGTCAGGTGTCCACAACCTTTTGGCAATATAGTG

>scaffold_210000323-4

ATATCCTCCTGAGACCCAAGGAAATAAATTGTCATCATTTTCTACTCCTTTCGGTTTAAAAAACATTAAACTATTTTGAGTTTTAAAATTTTATTGTTATTTTTTTATTTTACAGCATGTCCACTGTAGTGGATCACATGACCATTTTAGTTTGAAAAATCCCCCCTTTCCATAAAACGTGCTTGTTCCTATGGCGGCCATTTTGGATGCATTGTAAAAACTAGTTCCTAGCATCCCAGCCAATCAAATGACATCACTAGAAAGCCCAGGATGTCCTCTGTACAGTACAACAGAAACTGATAAGAGTACCTCCAAAAATACATTTATGAAAACTAAATGTCCACTACAGAGGACACAAGTCAGTAGGCGGGGTCTCAGGAGGATAT

>scaffold_210000325-1

TATTGGCAAAAGTTTGTGGACACCTGACCATCAGATCTATATGTGGTTCTTCACCAAAATGTTACTATAAATTTGGAAGCGCACAGTTTGTCTAGAAAGTCTTTGTATGTTGTAGCATTACATTATTCCTTCACTGGAACTATGAGACCCAAACACTGTTCCAGCATGACAATGCCCCTGTGTACAAAGCCCCTCAGCTCCATGTAGACATGGTGTGTTATGACTGGAGTGGAAAAACTTGAGTGTTCTGCACGTAGCCCTGACTTTAACACCACTGAACATCTTTGGGATGAACTGGAACGCCGACTGCACCACAGACCTCCTTACCCGACGTTAGTGCCTGATCTCACCAATACTATTGTAGCTGAATGAACACATTTCTCACATTCAAGCTTTACATTGTAGTGGAAATCCTTACTAGAAGAGTGGAGCTTAATGTAACAGCAAAATGGGGAGTAAATCCGGAATAAGATGTTTAAAACCACCATGTTTGTGATGGTCAGGTGTCCACAAACTTTTGGCAATA

>scaffold_210000325-4

CTTAAGCCGGGCTTACACTGTGGATATTGGCCACGATTTGTTGGTCTGAGACATCCTGAGAAATCCTAAAAGATTCCGATAATCCTACACTAAAATCTGTTGTCTTTGATGGCTAGTTTGACATGTTCACCGACAGCCGATTAATGACCTTTACGATCATTTTTTTCCTCCGATGAAATTCTGCCAGTGTTTAGAAGATTTCAGACATTTACCTGCAGTTTGACTTCTCGTACGATGACCGTCAAATCAAGAACCAATAGGAGCGCCGAACCTGTTGCCGCAATTAGCGCGACAACTACAAACTATTTGGGGGAAAAGTCACAAGTCCGTTGGACAGTACAGCAAGAAGAGAAACTTATTGAGGAATGTCATGAGCAAAATTAACGCTACAAATAGTTTTTGTTTGCTGCTAGCTACCATTTAGCGAGAGAATGCCGGTTACTGAACGTGTCACGGATTGATGACGTAAAACTCCGGATGAGTTTTCTTGTGCATGTCCGTTTTTGACGTGTCTTGACTGTCGTACAGCCTGATGTTATGACAAGTGACATCCTACAGTGTGACATGACACGGTAATCATGTTCGTACAGTGTGACGAGCAACAATCGCTAAAGACTATTAAAAATCGCACATTGTAACCCGGCTTAAG

>scaffold_210000326-2

ATGCTGTGTTCACACCAAACGTGAAGAGAATATGTGCATCACGTCACTCGCTCTACATTACTCGCGGGATGTTTTTCGCCTCACTCGCGCAATGTTTTTATTTGTAAAATATCAATTTCGCGGTGCCTGCAAACGGTAACAATAATGCTCTTTTCCATGTGCTTATGCAACGGACATGTCAGTAAGCTCTTTGCTGATTGGCTTGCGTGACAATGCATTGGGCATTTTGCGCCATTCGCATCGCCCAATGCGAATTTGCCTCTATTTGCGTCTTTGTATTGACTTTGTATGTAATCTACCAGCTTAAATAATAAAATTTTCCTTTGGTGTGAACACAGCAT

>scaffold_210000328-1

ATATACAAGGTGGGTGAAAATGAACTAGGCAATATTTAATGGCTAGAGAACTTGTAGTATCACTGGAGTCATGATGAAAACAGTCTGTAAATAGACGATAGTCAATAAAATTTCAAGCCTCGCACACAGGAGGACTTGGAGGCACGGATTCGGGAAGTTCTCAGCAATATTCCAAACCACTTCCTTTAGAAGACTGTGCATTCCATCTCCGGCCGTTTCACGAAACTGGTTGACGCCACCGGTGCCTACGTTGAAATTTACATTTGCTTTCATTTTCCTATGTAAGAAAGTACATGTACAATTTGTTTCAATAAATTTGTATTACAAATATGGATTTTATTACCAATTTTTAATGCCTAGTTACTTTTCACCCACCCTGTAGTAT

>scaffold_210000328-2

TACAGCGGAACATCGGCATACGAATTTCATTTGTTCTGGAGGCGAACATTCGTATTGCGATACGAAGTTTCCCATAAGAATTACTGTGAATGCAGACAATCCGTTCCAGCCACCCAAAAATATGACCAATATTACCGATTTCAAACACTATAATCATATTTTGGCATATAAAACCAATCAAAACATTTAGAAAAGACATGTAAATGTAGTAAAATATGAAATAAATAGACCCACGCCACCAATCTGGCAACAAAAAAATGTATGAAAAAAATCACAGCTTTTGAACGCATGCCGAAGGCAACGTTGGTATCGTGGGTCGACTCTTTGGAAACAGCTTTCGCTGACTGAGAAAGACATGTGCAAAATTTGTAAACTCAGTTGGCTTTGCTTGGCTGACGCAAACAAATTTTGAACAAGTCTCGTATGTACGCGCAACTTAGCCGGCGTTCAGTTTGGTTCCCGTTTGTATGCCGAAAATTTCTTGCAAAATTTCGGTTCTTAAGGGTGAAAATTGGTAAGGCAATGCATTCGTATGCCGAGGTTCCACTGTA

>scaffold_210000328-3

TACAGTGGAACATCGGCATACGAATTCAATTCGCTCTGGAGGCGAGTTCTTAAGGCGAAAATGTGTACCGCAAAATACGAATTTTCCCATAAGAAATAATGTAAATGCAGATAATCCGTTCCAGTAACCTCAAAATATTTTTAATATTACCAATTTCCAACCCTATAATCATATTTTTGCATATACAAACAATCCAAACATTTAGAAAAGACACGTAAATGTAGTAAAATATGAAATAAATAGACCCAAACCACCAATCTGGCAACAAAAAAACGTGTGAAAAATCACAGCTTATGAAGGCATGCTGAAGGCAACGTTGGTATCGTGGGTCGACTCTTTCAAAACAGCTTTTGCTGACAATTTTTTTTTTTGTAAAATTCGCTTCGAATGGCTTCACTTGGCTTTCCACTGATGCAAACAAGTTTTGAGCGGCTCCCGGATGTACGCGTAACTTAGCCGGCACTCGGTTAGGTTCGCTCCGTTTGTATGGCCAAAATTCTTGCAATATTTCAATTCTTAAGGCGAAAATTCGTAAGGCAGGGCACTTGTATGCCGAGGTTCCACTGTA

>scaffold_210000329-1

TACGGTGGCCGAGAGAGCGGAACGCACTGCAATTTAAGAAAACATGTGCAAATAAAAAAACACCAACAAATTAAGAAAACATCTTCATCAGTTTGACAAGACACGCGCTGCAAATCCTCACAACCCTACCAAATACAAAAACTAGCACAGGCGACAACGGAAATGTTTCCAGGGGACCCCCCCAAAACGTGATGAACCCGGCTGGGACCTGCTTATTGTTTCACAGTTAATTGTGTAATTCGTCAGATTATTTAGGCTAATAATATCCAAAAGACCAAGGAATCATACGATCGGGATGATAAAATAAACAAAATAGGTACCCTTCAGTTCACCGATAACACCACTGACAAATAGCCACTGAACAATAAACAGGTCCCAGCCAGGTTCGTTACTTTTTGGGATTCCTTGAAACATTTCCGTTGTAGTCTGTGCTATTAGCAGCGCGTTTCTGTATTTGGTTGGGTTGTGAGGATTTGCAGCGCGTTTCTGTATTTGATTGGGTTGTGAGAATTTGCAGCACATGTGCTGTCTGATGAAAATGTTTTTTTTAATTGTTGGTGTTTTTTCTATTTGCATGTGTTTTCTTAAGTTGCAGCTCGTAGAGCAATCTCGGCCACCGTA

>scaffold_210000329-2

TAATCCGGGCTTACACTGTGCGATTTTGGCCACGATTTGGTCGTCTAAAAGACAAATTTTGAAATCCTAATGGATTCTTATAATCCTAGGCTAAAATCTGTTGCCTTTGATCACTACGACATGTTCGACATGTTCACCGACAGCCGATTAATGGTCGTTGCGATCAGTTTTTTACCGCCGATGAAATTCTAGCTGTGTCCGACGTTTTTAGATACTTTCCTCTGCAGTGTGACTTCTCCTACGACGACTGTCAAACAAAGAACCAATAGGAGTGCCGAACTGATGACGCAATTAGCGCGACAACTACAAATCACCTCAGGAAAATGTTGATACGAGTCCAGTGGACAGAACAGCAAGAAGAGAAACTTATTGAGTTACGGAGGGAAAAGAAGTGGTTTGTATGACGTGTCGTCGCTGCTGTTTGTTTGCTGCTTTAGCAAGACACAGCGAGTTACTGAACGTGTCACGGATTGATGATGTAAAACTCCAGACGAGTCTTCATGTGTGCGTCTGTTTTTGACGTGTTTTGACTGGCGTACATTCTGACATCTAAGATCCCACAGTGTGACGTGGGGAACATCTTCGTACAGTCTGACAAGCAACAATCGCCAAAGACTATTTAAAATCGCACAGTGTAACCCGGTTTA

>scaffold_210000329-7

TAGGGCTGTGTATTGGCAAGGGCCGCACGATACGATACATATCACGATACATGGGTCACGATACAATATATCACGATATATTGCTATACAATACATATTACAATACATTGCATTACTTAAAAATAATAATAATAATAATAATAAAAGTAAAACAAAGAGCTACAAACACAACTTGCTGTGTGCCACCTGGAGGATTCTAGTATGTGCATCACATTATATTGATTACACAGCAGACAAATAAATACTGAGGGTTTTAATGAAATATTAACGTAAACATAAATCAGTTTAAAACGTAATTTAAATATGTATCGATACTAGAGGTTAGAATATCCATACACTATAGTGAAAAAAATATATTGCGATAGTTAGCTGTATCGATATTTTTACACAGCCCTA

>scaffold_210000330-1

GTGGCTCCATCCGAAAATGCATACTTCGCTTCTATTTAGTATGCAAAAATGAGTACGCGAGGTGAGCAGTATGTCCGAATTCTTAGTATGCGAAAAGTTCCCGGATGATCTACTACTTTCGGCCTGGATTCTGAAGTACGCATACGATGGGCATGTTACTATTCCATGAGCCCACGGGAGAGACGACTCGTCATATGCGAAAACTGCGGAAAGCGGCGACGCAGCACTTTTCAAGTGTAGAATAGCTTTAAGTACTTCAGGAAAAAAACTTTTTCTGTGGTTAAATTGCACTTGTTTTATCATTTATCCAAGTAAACATCAACCAACTTTAAATTTGTGCTGTTGTGACGCTGTATGTCATGTGACAATATCAACGGATGTAGTATGTATGACATTTATATTACCCATAAACTTACTTTTTTGATGGTCAGGAAGTACGTACTAACTCAAATGTAGTAAGTGCTTAAGTAGTATGCGATTTCGGACGGAGCCAC

>scaffold_210000333-2

TTACACTGTGCGATTTTGGCCATGATTTGGTCGTCTGGGACAAATTTTGAAATCCTAAAGGATTCCTATAATCCTACGCTAAAATATGTTGTCTTTGATGGCTAGTTTGACATGTTCACCGGCAGCCAATTAACGGCTGTTGCAATCAGTTTCTTCCTCCGATAAACTTCTTGCAGTGTCAGAGGATTTCAGACGCTGTCCTGCAGTGTGACTTCTCCAACGACGACCGTAAAACCAAGAACCAATAGGAGCGCCAAACCTAATGACGCAATTAGCACGACACCTACAAATCACCTCTGGAAAATATCAAAACGAGTCTAGTGGACAGTACAGCAAGAAGAGAAACTTATTGAGGCGTGTCATGAGCAAAATTAACGCTACAGATTGCTTTTGTTTGCTGCTAGCTACCATTTAGCGAGAGAATGTGGGTTACTGAACGGGTTGTATGTCACGGATTGATGACAAAAAACTTCGGACGAGTCTTCTCGTGTGTGTCCGTTTTTGACACGCTCTGTCTGTCGTACAGTCTGACATTATGACAACTGAGATCCTTCAGTGTGACATGGGGATCATGTTCAGACAGTCTGACAAGCAACAATTGCAAAGGACTATTAAAAAATCGCACAGTGTAA

>scaffold_210000334-1

TATATTGCCATACGTTTGTGGACACCTGACCATCACAAACATATGTGATTTTAAACATCTTATTCCAGATTTACTCCCCATTTTGCGGTTAGATTAAGCTCCAATATTCTAGTAAGGCTTTTCACTACAATTTAGAGCTTGAATGTGAGAGATGTGTTCATTCATCTACAATAGTAGACTAACGTCAGGTCAGGAGGTCTGTGGTGTTCATCCCAAAGGTGTTCAGTGGTGTTAAGGTCAGGGCTCTGTGCAGAACGCTCAAGTTTTTTCACTCCAATCAGAACACACCAGGTCTTCATGGAACTCGGGAGCTTTGTGCACAGGTGCATTGTCATGCTGGAACGCTGTTTGGGCTTTTTAGTTACAGTGAAGAGAAATTTTAATGCTACAGCATATAGACACATCCTAGTCAACTGTGTCCTTCAAACTTTGTTGCAACAGTTTGTGGAAGACACACTCATAGGTGTGCTGATAAGGTGGCCACAAACTTTTGGCAATATA

>scaffold_210000334-3

TACACTATATGGAAAAAGTATTGACACCTGACCATCACATCCATATTTTTGTTTTCAGATTTAGTCTTAGTCTTTCTCCTTTAATAACCTCCACCATTCTGGAAAGCTTTCCACTAGATTGTGGAGCGTGACTGTGGGGATTTGTGTTCGTTTAGCCACAAAAGTATTCGTGAGTTCAGACACTAATGTTGGGTGAGGTCTTGGGTGCAGTGTTCCAGTTCATCCCAAAGGTGTTCAGTGGGGTTGAATCAGAGTCAGGGCTCTGTGCAGGACACTCAAGTTCTTTCACACCAACCCAAACACACCATGTCTTTATAGAGCTCAGGGGCTTTGTGCACAGGGACATTGTCATGCTGGAACAGTGTTTGGGTCTTTTAGTTCAAGTGAAGAAATGTTGTATTGCTACAACATACAAAGGCATTCTAGGCAACTGTGTGCTTCCAACTTTGTAAAAGTTCATAAAAGTTTGGGGTGGGTGTGCTGGTAAGGTGTCTACAAACGTTTGGCCATATAGTGTA

>scaffold_210000335-1

CATGCGCTATATTGCCAAAAGTTTGTGGACACCTCACCACCATATCCATAAGTGGTTCATCTCCAAAATGTTGCTACAAATTTGGAAGCACACAGTTGTCTAGAAAGTCTTTGTAAGTTGTAGCATTACATTATTCCTTCACTGGAACTAAGAGACCCAAACACTGTTTCAGCATGACAATGCACCTGTGCACAAAACCCCTGAGCTCCATTAAGACACGGTGTGTTATGATTGGAGTGGAAAAACATCTGCACAAAGCCCTGACTTTAACACCACTGAACCCCTTTGGGATGAACTGGAACGCCGACTGCACCACAGACCTCCTGACCTGACGTTAGTGCCTGATCTCACTAAAACTATTGTAGCTGAATGAACACATCTCTCACATAACAGCTCCAAATTGTAGTGGAAAACCTTACTAGAAGAGTGGAGCTTAATCTAACAGCAAAATGGGGAGCAAATCTGGAATAAGATGTTTAAAATCACATATGTTTGTGATGGTCAGGTGTCCACAAACTTTTGGCAATATAGTGCATG

>scaffold_210000335-6

TAGGGCTGTGTAAAAAAAATATCGATACAGCTAACTATCGCAATCCTTTTTTTCACAATAGTGTATGGATATTCTAACCTCTAGTATCGATACATATTTAAATAACATTTTTAACTGATTTACGTTTACGTTAATATTGAATTAAAACCCTCAGTTTTTAGTGGTCTGCTGAGGGATCAATATAATGTGATACACATACTAGAATCCTGCAGGTGCAACACAGCAAGTTGTATTTTTACCTATTTGTTTAACTTTTTTATTTTATTTTCAAGTATTGCAATATGTATTGTATTGCAATATATCGTGATCTATTGTATCGTGACCCATGTATCGTGATATGTATCGTATCGTGAGGCCCTTGCCAATACACAGCCCTA

>scaffold_210000337-1

TATACATGGTGGGTGAAAAGTAACTAGACATTAAAAATTGGTAATAAAGTCCATATTTCTAATACAAATTGATTGAAACGACTTGCACATGTACTTTATTACATGAAAGTGAAAGCAAGTCTTTAAATTTCAACGTAGGCACCGGTGACGTCAACCAGTTTCCTCGTTTGGGACATTGCTGAGAACTTCCCGAATCCGTGCCTCCAAGTCCTCCAGTGTGCAAGGCTTGAAATCCTATTTACTATCGTCTATTTAGAGACTGTTTTCATCATGACTCCAGTGATACTACAGGTTCTCTAGCCATTAAATATTGCCTAGTTCATTTTCACCCACCCTGTATA

>scaffold_210000338-3

ATATCCCCCTGAGACCCAAGGAAAAAACTGTCATCAATTTTTTTTGTTTTGTGTGACTTCTTACTCCTTTCGGGTTAAAAAAACATTCTACAATTTAGAGTTTTTAAATGTTCACTTTAGAGGACCACAGGACCTTTTTAGTTTGAAAAGACAGTCCTTTTCGTAAAACGTGCTTGTTCTAATGGTTGCCATTTTGGATGGATGCAGTGTAAAAACTAGTTCCTAGCATCTCAGCCAATCAAATGACATCATTGAAAGCCCAGGATGTCCTCTGTATAGTACTGGAGAAACTAATAAAGAGTAGCTCCAAAAATTTTATGAAAACAAAAGGTCCACTACAGAGGACACAAGTCAATGGGCGGGGTCTCAGGCGGATAT

>scaffold_210000340-1

CTATATTGCCAAAAGTTTGTGGACATCTGACCATCACATATGTGATTTTAAACATCTTATTCCAGATTTGCTGTTAGATTAAGCTCCACTCTTCTAGTAAGACGTTCCACTACAATTTGGAGCTGGAATGTGAGAGATGTGTTCATTCAGCTACAATAGTTTTAGTGAGATCAGGCACTAACGTCGGGTCAGGAGGTCTGTGGTGCAGTCGGCGTTCCAGTTCATCCCAAAGGGGTTCAGTGGTGTTAAGGTCAGGGCTTTGTGCAGATGTTTTTCCACTCCAATCATAACACACCGTGTCTTAATGGAGCTCAGGCGCTTTGTGCACAGGTGCATTGTCATGCTGAAACAGTGTTTGGGTCTCTTAGTTCCAGTGAAGGAATAATGTAATGCTACAACTTACAAAGACTTTCTAGACAACTGTGTGCTTCCCCCTTTGTAGTAACATTTTGGAGAGGAACCACTTATGAACATGATGGTCAGGTGTCCACAAACTTTTGGCAATATAG

>scaffold_210000340-4

GCTACGTTCACACTGCAGGCAAATGTGGCCCATGTGTGAGAATGTGTGATTTGTTTTTGCCCACATTTGACTCAGATCTGTTTTTCTCATGACAGTGTGAACAGCACAAACCGCATGGAATCTGATCTTTTCAATTCTGATTTGTGCCACTTCCATATGTGGTATTAAATCCGACACAGGTCACATGTTTTGCAATGCGACCGCAGTGTGAACAGTTATGTCAGAATTCATGCGACTTTTACATCATATTTGTTGTAACTACAAACAGTTGATATATAATCGATTCCGCTGAAGCGTTCACGTCACGGTCCCACCAATCCTGTCTCCGGCTCCACATCCACACACACCTCCATACTGAAGTAGCAGCCACTGCTCCACAACATGCTATTATTCAAAATTTGGCCCTTTTTCTCCTCATCCTCTTCCTCCTCAACGTCTGCTCATTAATTCGCTGTCGTCCGCTGCACATCACCTTATAAATGTAAGCAGCTAACGCGTAAATGCTGCCTTCAGTGTCTTCCATGTTTATCTTCCCGTATGACAGGATGCTGCGTGAGATGTAGAATTAGTTCTTTTGCGCATGCGGCTCAGTTTAGGATCGTGATCAGTTCACACTGGAAATCTGATATTGACCACATTTTAAACAACAACAACGTAAACAGCTACACAAAAAAATCTGATTGGAGCAAAAATCCGAATTGAGCACTAAGCCATGCAGTGTGAATGTAGC

>scaffold_210000343-9

TATATAGCCAAAAGTTTAAGGACACCTTACCAGCACACCCATAACTGTGTCTTCCCCAAACTGTTGCCACAAAGTTCCAAGCACACAGCTGTCTAGGAGATCTGTATTTGCTGTAGCATTAAGATTTCCCCTTAAAACTAAGTGACTCAAACACTGTTCCAGCATGACAATTCCACTGTGCACAAAGCGTCTGTGCTCCATGAAACCATAGTATGTTAAAGTGAAGGAAAGTATATATAGTATAGGAAGGAAAGAACCTGTGTGTTCTGCACAGAACCCTGACCTCAACCCCACTGAACACCTTTAAGATGAACTGGAATATTGACTACACCCCAGGCCTCCTCATTCAACATTAGCACCCGAACACATTAATACTATTGTGATTGAATGAACAAATCCCCACAGCCAAGCTCCAAAAGCTAATGGAAAGCCTTTTCAGAAGTGTGGACATTATTAAAGGTGCAAGAGTGAGACTAAATGTGAAGAGATGTTTAAAAAACACGTATGAATGTCATAGTCAGGTGTCCCAATACCTTTGGCCATATA

>scaffold_210000344-2

GCTACGGTGGCCGAGATTGCTCAACACGCTGCAACTTAAGAAAACACATGCAAATAGAAAGAACACCAACAATTTAAGAAAACATCTGCATCAGTTTGAAAACACACATGCTGCAAATCCTCACAACACAACCAAATACAGGAAGTATTTGCAAATAACACAGACCACAACGCAAATTTTTCAAGGAATCCCAAAAAGTGACGAACTTGGCTGGGACCTGTTTATTGTTCAGTGGTTATTGGTCAGTGGTATTATCGGTGAACTGAAAGGTAAGTTTTTTGTTTATTTTATCATCCCGATTGTATGATTCCTTTGTCTTTTGGATATTATTATCCTAAATAATCTGTTGAATTACACAATTAACTGTGAAATGATAAGCAGGTCCCGGCGGGGTTCGTAACTTTTTTGGGGAGCCCTTTGAAACATTTCTGTTGTGGTCTGTGCTGTTTGCAGCACATTTCTGTATTTGCGGTTGCATTGTTTGCAGCACGTATGTTGTCAAACTGATGAAGATGTTTTTTTAATTTGTTGGTGTTTTTTCTATTTGCATGTTTTTTCTTAAATTGCAACGCATTGAGCTCTCTCGGCCACCGTAGC

>scaffold_210000344-11

ATATCCTCCTGAGAACCCGCCCGTTGTGTCCTCTGTAGTGGACATTTTTTTAGCTACTCTATCAATTTCTGCTGTACTGTACTGATGTAGGACATCCAGGGCTTTGTAGTGATATGTCATTTGATTGGCTGAGATGCTAGGAACTAGTTCTTACACTTCATCCAAAATGGCCATAAGAACAAGCATGTTTTATAGAAAGGACTGTCTTTTCTAACTAAAAAGGTCCTGTGGTCCGCTACAGTGGACATGCTGTAAAAAATTTAAATCTCAATCAAATTTAAAAACTCTAAATTGTAAAAATGTTTTTAACCCAAAAGGAGTAGGAAATCACACAAAACAAAAACAATTGTTGACACTTTTTTTCCCTGGGACTCAGGAGGATAT

>scaffold_210000346-4

TAGGGCTGTGTATTGGCAAGGGCCTCACAATACGATACATATTAAGATACATGGGTCACGATACAATATATCATGATATATTGCAATACAATACATATTGCAACACATTGCAATACTTAAAAAAAAAAAAAAAAGGAAAACAAATAGCTAAAAATACTTGCTGTGTACCACCTGGGGGATTCTAGTACGGTATGTGTATCACATTATATTGATTACTCAGCAGACAAATAAACACTGATGGTTATAATTAAATATTAACGTAAACTTAAATCAGTTTAAAACATAATTTAAATATGTATCGATACTAGATGTTAGAATATCGATACACTATCGTGAAAAAACATCATAATTAGCTGTATAGATATTTTTACACAGCCCTA

>scaffold_210000351-2

AGGCTACGTTCACAGTGCGAGGCTTAGTGCTCAATTCCGGCTTAGTTGCTCAGCTTTTTGTATAGCTGTTTACATTGTTGTTTAAATGTTGCCAATATCAAATTTCCAGTGTGAACTGATCATGGTCCTAAACTGACCCGCATGTGCAAAAGAACTCTACGTCTCGCGCAGCGTTTTATAAGTAAATTAAAATATTATAAATTTATAAGGTGATGTGCAGCGGACAACAGCGAGTTAATGAGCAGTCGTTAAGGAGGAAGAATATGAGGATGAAAAGGGCCAAATTTTGAATAATAGCATGTTTTGGAGCAGTGGCTGCTACTTCAGTATGGAGGTGTACTGAAGCCGGAGCCAGGAGTGGTGGGGCCGTGACGTGAAATCGATTATATATCAGCTGTGTGCCAGTGCAGAATGATGATGCGTGTCGATCAAAAATTACGTAAAAATCGCATGAATTCCAACATAACTGTTCACACTGGTTGCATTGCAAAACATCTGACCTGTGTCAGATTTAATACCACATATGGAAGTGACACGAATCGGAACTGAAAAGATCAGATTCTATGCAGTTGGTGCTGTACACACTGTCATGAGAAAAACAGATCTGAGTCACATGTGGGCAGAAAAATTGGATATGAGCCACATTTGCCTGCAGTGTGAACGTAGCCT

>scaffold_210000355-1

CAGGGCTGGGTAAACCAAGTTCCATATGTTACTTTGTTCCTTTTGTTTTCCAGCTACCCCTGCCCTACCAACTGCTGCATACCTGGTTTAGGTGTGTTCAGTCAATCAGAAGCTGGAAGATACCATTTCAGATGAGGGTGGAGTGGGGAAGACCTCTGATTGACTAAACACACATAAACCAGGTAATCAGCAGAGGGTAGGGCAGGGGTAGCGGGAAAACAAACGGAACAAAGTAAATTAAAAAACATGGGTTACCGAGCCCTG

>scaffold_210000356-5

TAGGGATGCACCGATACAATTTTTTGTACTAGTACCGATATTTTTTTCCGATACCGATTCCGCTACCTGTACTTTTTTGGGATGTGGGATTATTTGTATGGCTCTCGGGTTATCTCTTGCAATTTTCTCTCGTCTTGCACAGGTTTGCTGCAGGGTTGCTTGCCGAGTGATAACGTTACTGCTAGCGGCAACTTCTCCATGCTCACTTTTTTTTTTCAGATTACTCGTATTATAAGTGCTCATTTTTGCACCTCTTGATATTTTTGCGGAACAAAGTTTGCAGTCTGCCATGTGTGGGTTGTCATCAGTAATTTATAAATATTTCCACACCACTGACCCTGACATTCTGTTGTTGCTGTCGGAGTCTTTGTGCACTGAAAGTTCTGTTAATGACATCACAAGGGATCTTTGTTATCGGAGTGTTTTTACGACTACGAGTCCGATCGGGCCCGATACCGATACCAGTTTCGGTACCGGTGCATCCCTA

>scaffold_210000358-2

ATACGAGGTGTGTCCAGAAAGTAATGAGAATGAAGTTCCTACGCTGGAGAAGGGCTCTGTGAAGGGAGAAGCTGGCCACAGTCATTTGGGGTGGGATGCCAGGAATACGTTAGAACACTGATCAGTTGCATTCCAGCCCTGTCAAAGTCAAGACAGCCTTTTTTGTGACACGCGGTTCAAGTGCCTCGTATTAAGAAAAATGCAAGCAAGTTTAGAAACGCAGTACGCAATCATTTTGTGTGAAATTGGGTAAAAGTCAGAAACTTGAAACTAAGAGTTTTCCGTTTGCGTCCTGAGATCGGCTCAACTTGGATCCTTCATCACGACAACGCACCAAGTCACAGCGATTTGGCTGTCAGAGAGTTTTGGGCTGAAAAACACATCACGGTGCTTCTCCATCCACCTTACAGCCTCGACTTGGCTCCTTGTGACTTTTTCATCTTTCCAAAGATGAAAGGGCAGCATTTTGGTAGTGTGGATAACGTCCAATGAGCCACTACGCAGGTTCTGAACGCCATCCTACTTGAAGACTTCCAGGGAGGCTGTGAAGAATGGAAAATGGCAGCGCTGTGTACACTCGCAAGGGTACATACTTTGAAGGAGACAATGATCAATTTATAAAAAATTTCAATAAAATAATTTTTTAAAATATCATTCTCATTACTTTCTGGACACACCTCGTAT

>scaffold_210000358-12

TAGGGCTGTGTAAAAATATTGGTACAGCTAACTATCGCAATGCTTTTTTTCACGATCATGTATCGATATTCTAACCTCTAGTATCGATACATATTTAAATTACATTTTAAACTGATTTACGTTTACATTTATATATAATTTAAACCCTCAGTTTTTATTTTTCTGCTGAGTAATCAATATAATGTGATACACATTCTAGAATCCTCCAGGTGGTACACAGCAAGTTGTATTTTTAGCTATTTGTTTTACATTTTTAAATTTATTTTTTTAAGTATTGCAGTATGTATTGTATTGCAATATATCGTGATATGTATCGTATCGTTAGGCCCTTGCCAATACACAGCCCTA

>scaffold_210000376-5

TACACTATATAGACAAAAGCATTGGAACAACTGACCATTACATCAATATGTGTTTTTTTTAACATCCTGTTCCAGATTTAGTCTTACTTTTACTCCAATAATAACCTCCACACTTCTGGAAAGGCTTTCCACTAGAGTGTGGAGTTTGGCTGTGGGGATTTGTGTTTATTCAGCTACAATAGTATTCAGACACTAGTCTTGGGTGAGGAGCTCTGGGGTGCAGTTTGTGTTCCAGGTCATCACAAAGGTTTTCAGTAAGGTTAAGTCAGAGTTGGGGCTCTGTGCAGGACACTCGAGTGCAGAACACACCATGTCTTTATAGAGCTCAGTGGTTGTGTGCACAGGCGCATTTCTATGCTAGAACAGTGTTTGGGTCTCTTAGTTCCAGTGAAGGAATAATGTAATGCTGCAACATATAGAGACATCCTAGACAACTGTGTGCTTCAGAGTTTGTGATAAAAAACACTACAACATTAATGTGGTTGTGCTGTTAAGGTGTACACTTTTGGCAATATAGTGTA

>scaffold_210000377-4

CAGGGGTCACCAAACTTGATCCTGGAGGGACGGTGTCCTGCAGAGTTGAGCTCCAACTTGCCTCAACACACCTGCCTGGAGGTTTCAAGTATACCCAGTAAGACCTTGATTAGCTGGTTCAGGTCTGTTTAATTAGGGTTGAAGTTAAACTCTGCAGGACATTGGCCCTCCAGGACCAAGTCTGGGGACCACTG

>scaffold_210000385-4

ATATTTCCATCCACCTGTTTTTATGAGCATTTTGGACTATCGAATAAAAAAAACGGCAAGATGCGCATAAATTTTGAAAATTTTTTTGAAAATTCTTTGCACACAACTGTGGAGGATAAACGTTTTATCCGATAAGGAAAATGCGCATAAACTACGACGGAAACACATTTACCGAATAAATTCCAGCATGCGCATTTAAAAAAAGTCATGTGATTTTGCTTCAACAGATCAGGTGATAACAAAAATGGCCATTTTATAACGCGCAATGGCAATGCGTTTTTCTGTCTGAACTGAGGCGCGATGGGATGGCATTATTGGACTAACCCCAGCAGACCGATCTCATTGCACAAAATCTGAAATATTGTTTTGGTCATTCTAAAATGCCTTAGCCAAAGTCTGTCATCAAAATGGTTTGGTATTATTACCCCCCAGAACTGTCTTGACCGACTGTGTTCCCAAAGAGCAGTCTCCTGCCTCCAAAAGCATGATGACATGACTGCGTTCATTGCGTTTCGTCTATGTGTTCTGAGACGCAAATAATTTATTATATACAAAAAAAGGCCCTGCCTACCGCAGCAACTTCCATTTTTCTTTTTGATATTTGGCAACAGTTTATCAGGAAGTGATGATTTTGTTCTCTTTGACTTGTTGGATCAAAACGGTGCTTTATTCGCAAATGTTTTATGCAATATTTCAGTTTTGCACATAAGTTTAATTGGTTTCTTTGGATGGAAATAT

>scaffold_210000386-1

TCTAAGGCTACGTTCACACTGCGAGGCTTAGTGCTTAATTCCAATTTTTGCTCAGATCAGATTTTTTTGGATAGCTGTTTACATTGTTGTTTTAAATGTGGCCAATATCAGATTTTCACCGTGAACTGATCATGGTCCTAAACTGACCTGCATGCGCATATGAACAAATTCTTCGTCGCACGCAGCGTCCTGTCATACAGAAAAATAAACACGGAAAACACTGAAGGCAGCATTTACGTGTTAGGTGCTTACATTTATAAGGTGATGTGAAGTGGACGACAGCAAATTAATGAAGTCGATGAGGAGAATATTTTTTTTTTACTAATAGCATGTTGTGGAGCAGTGGCTGCTACTTCGGTATGGAGGTGTGTGTGGATCCGGATACAGGAGTGGTGGGACCATGATGTGAAAGCCTTCAGCGAAATTGATTATATATCAACTGTTTGTAGTATGCACCAGTGCAGAATGACGACGCATGTCGATCAAAAATTACGTAAAAGTCGCATGAATTCCGACATAACTGTTCACACTGCGGTCAAATTGCAAAACATCTGACTTGTCGGATTTAATACCACATATGGAAGGGGCACAAATCGGAATTGAAAAGATCAGATTCCATGCAGTTTATGCTGTTCAAACTGTCATGAGAAAAACAGATCTGAGTTGCATATGGGCAAAAATTCGGATTTTGGCCACATTTGCCTGCAGTGTGAACGTAGCCTAAGA

>scaffold_210000387-1

CACTATATTGCCAAAAGTCTGTAGACACCTGACCATCAGATCCATTTGTGGTTCCTCTCCAAAATGTTGCTACAAATTTGGAAGCACATAGTTGTCTAGAAAGTCTTTGTATGTTGTAGCATTACATTATTCCTTCACTGGAACTAAGAGCCCCAAACATGTTCCAGTCCTGTGCACAAAGCCCATGAGTTCCATTAGGACATGGTGTGTTATGATTGGAGTGGAAAAGCCCCGAACTTAACACCACTGAAAACCTTTGGGATGAACTGGAACGCCGACTGCACCACAGACCTCCTTAACTGACGTTAGTGCCTGATCTCACTAATACTATTAAAGCTGAATGAACACAAATCTCACATTCAAGCTCCAAATTGTAGTGGAAAGCCTTACTAGAAGAATGGAGCTTAATCTAACAGAAAAATAGGGAATAAATCTGGAATAAGATGTGTAAAATCACATATGTTTGTGATGGTCAGCTGTCCACAAACTTTTGACAATATAGTG

>scaffold_210000388-1

TATGAGGGGGTATCAAAAAGTTTTGAGACACACCAACCGATGGCAGCGCAAAACCTTGTGCTGCCATCTGTTGGTGTGTCTCAAAACTTTTTGATACCCCCTCATA

>scaffold_210000395-2

ACTAGGGCTGTGTATTGGCAAGGGCCCACGATATGATACATATCACGATACATGGGTCACGATACAAAACAATCACGATATATTGCAATACAATACATATTGCAATACTTCAAAAAACAAAAAAGGAAAACAAATGGTTAAAAATACAACTTGCTGTGTACCACCTGGAGGATTCTAGTATGTGTATCACATTATATTGATAACTCGGCAGACAAATAAACACTGAGGGTTTTAATTCCATATTAACGTAAACCAAAATCTGTTTAAAACATAATTTTAATATGTATCGATACTAGAGATTAGAATATCGGTACACTATCGTGAAAAAAAATACCGTGATCGTTAGTTAGCTGTATCTGTATTTTTACACAGCCCTAGT

>scaffold_210000397-2

ATACACTATATTGCCAAATGTTTGTGGACACCTGACAATCACAAACATTTTAAACATCTTATTCCAGATTTACTCCCTATTTTGCTGTTAAATTAAGCTCCTCTCTTCAAGTAAGGTTTTCCATTACAATTTGGAGCTTGAATGTGAGATATGTGTTCATTTAGCTACAATAGTATTAGTGAGATCAGGCATTAACGTCAGGTAAGGACGTCTGTGGTGCAGTCAGCATTCCAGCTCATCCCAAAGGTGTACAGTGGTGTAAAGGTCAGGGCTTTGTGCAGAACACTCAACTTTTTCCACTCCAATCTTAACACACCGTGTCTTAATGGAGCTCAGGGGTTTTGTGCACAGTTGCATTGTCATGCTGGAACTGTGTTTGGGTCTCTTAGTTCCAGTGAAGGAATAATGCAATGCTACAACATACAAAGATATTCTAGCCAACTGTGTGCTTCCAACTTTTTAGCAACATTTTGGAGATCAACCACATATGGATCTGATGGCTAGGTGTCCACAAACTTTTGGCAATATAATGTAT

>scaffold_210000403-6

TAGGGCTGCACAATATATCGAAATTATCGCAATATCGCATATCGAGATATTCATATCGCAATGGTTTGAGATAAATGAGCGATTTAATACTTAAAATAGTAATGTGTGGTCAAAGTTTTAGGATAGTAAAATATCTGGCATGCGGATGTTGGTTGGTTGGGATATGCGTGCGTGTGCTTTGAGGCAAGACAATCAACAAGCTGCAAAGCTTTTAAAGCGAGCTCATATTGATAAGATGATCCAGCCCGAAAAAAAACACCTGCAGATAAAATAGCTACTAAAAATAGCATTTCTCAAGGGAAATGTTATATCGCAAGAAATATCGTTATCGCAATATTCAATAACAATATCACATTTTCCCAGTATCGTGCAGCCCTA

>scaffold_210000407-6

TAGGGATGCATTGATACCATTTTTTTAATGACCGAGTACGTGTACCAATTTTTTTTTCCTGGTACTCGCCGATACCGATACAGATACCTGTACTTTTTTGGATGTGGGTTTATTTGTATGGTTCTTGAGTTATCTCTTGCCATTTTCTCTCGTCTTGCAAGGGTTTGCTGCAGGGTTGGTTGCCAAGTTCTAACATTACTGCTAGTGGCAAATTCTCTGTGCTCACTTTTGTGTTTTAATTTCAGATGTTTTATCAGATTACTCATATTATAAGTACTCAGTTTTGTACCTCTTGATATTTTTGTGGAACAAAGTTTGCAGTCTGCCATATGTGTGTTGTCATCATTAATTTTAAATATTTCCACATTCTATCGCTGCTGCCGGAGTCTTTGTGCACTAAACGTTCTGTCAATGACGTTACAAAAGGTATCTGTCTTTGGTATCTGTCTGAGTGTTTTTACGAGTACATGAGCTCAGTATCGGTATCGGTGCATCCCTA

>scaffold_210000416-1

TAAAGACAGGTTTACACTGTGCGATTTTTAAATAGTCTTTTGCGATTGTTGCTTGTCAGACTGTACGAGTATGATCCCCATGTCACTCTGTAGGATCTCAGTTGTCATAATGTCAGACTGTATACGACAGTCAAAAACGGACACACACACGACGACTCTTCCGGAGTTTTACAACATCAATCCGTGACACGTTCAGTAAGCTGCGTCCTCTTGCTACATAGTAGCTAGCAGCAAACAAACAGCAGCAATGACACGTCATACAAACACTCCTTTTCCCTCTATGACTCAATATGTTTCTCTTCTTGTTGCACTGTCCACCTGACTCGTATCCACATTTTCCCATAATGATCTGTAGTTGTCGCGCTAATTCAGGTTCAGCGCTCCTATTGGTTCTTGGTTTGACAGTTGTCGTAGGAGAAGTCACACTGCAGGAAAGTGTCTGAAATCTTCTAACACTCCCAGAATGTCATCAGAGGAAAAAACTGATCGCAACGTCCATTAATTGGCTGTTGGTGAACATGTCAAACTAGCCATCAAAGACAACAGATTTTAGTGTAGGATAATAAGAATCTTTTAGGATTTCAAAATGTGTCTCAAATGACCAAATCGTAGGCAAAATCGCAGAGTGTAAGCCCGGCTTTA

>scaffold_210000422-1

ATACACTATATTGCCAAAAGTTTGTGGACACCTGACTATCACAAACATATGTGATTTTAAACATTTTATTCCAGATTCACTCCCCATTTTGCTGTTAGATTAAGCTCCACTCTTCCAGTAAGGCTTTCCACTACAATTTGGAGCTTGAATATGAGATATGTGTTCATTCAGCTACAAGAGTATTAGTGAGATCAGACACTAACGTCAGGTAAGGAGGTCTGTGGTCTGTGGTGCAGTCGGCATTCCAGTTCATCCCAAAAGTGTTCAGTGGTGTTCAGGTCAGCGCTTTGTGCAGAACACTTAAGTATTTCCACTCCAATCATAACACACCATGTCTTAATGGAACTCAGGGGTTTTGTGCACAGGGGCATCGTCATGCTGGAACAGTGTTTGGGTCTTTTAGTTCTAGTGAAGGAATAATGTAATGCTACCACATACAAAGACATTCTAGTGTGCTTCCAAACAGTGTGCTTCCAAATTTGTAGTAACATTTTGGAGAGGAACCACATATGGATCTGATGGTGAGGTGTCCACAAACTTTTGGCAATATAGTGTAT

>scaffold_210000428-2

CTAAAGCCGGGCTTACACTGTGTGATTTTGACCACGATTTGGTCGACCGAGACAAATTTTGAAATCCTTAAGGATTCCTATAATCCTACGCTAAAATCTGTTGTCTTGGACCGCTAGTTTGACATGTTCACCAACAGCAGATTAATGGCTGTTGCGATCAGTTTTTTCCTCCAATGAAATTCTGGCAGTGTCAGAACATTTAAGACATTTACCTGCAGTGTGACTTCTACGACGACCGTCAAACCAAGAACCAATAGGAGCGCCGAACGTGATGACGCAATTAGCGCGACAACTTCAAACCACCTCCGGAAAATATCTAAACGAGTCTAGTAGACAGTACAGCAAGAAAAGAAACTTATTGAGGCATGGCATGAGCAAAATTAACACATTTTTGTTTGCTGCTAGCGAGACACAGCGGGTCACTGAACGTGTCATGAATTGATGATGTAAAACTCCGGACGAGTCTTTCTTGTGTGCATCCGTTTTTGACTCATCTTGATTGTCTGACACTGACAACTGAGATCCTACAGTGTGACACAGGATCATATTCGTACAGTCTGACAAGCAACAATCGCAAAAGGCTATTATAAATCGCACAGTGTAAGCCCGGCTTTAG

>scaffold_210000430-1

CCAATCTTATCCACAAAGCGATGGTGTGGATTCAGGATTTCATTTCAATCAAGCAGGAGTCACAGCTGATTTCACCGGTTTAATCAGTTGATCTGGGCTTTTTAGTAGACTCAGATGTGACTTTTGCTTGGTTGAAATAAAAACCTGCACCCACACCGGCCCTTTTCGGATAAGATTGG

>scaffold_210000433-24

TAGTGATGCACCGATACAGATACTGGTATCAGCATCGGGCCCGATACTGAGTTCATGTACTCGTACTTGTACTTGTAAAAACACTCTGATACCAAAGACTTGTGACGTCATTGACAGAAGTGCACAAAGACTCCGGCAGCAGCGACAATGTCAGGCTCAGCGGTGTGGAAATATTTCAAAATGAATGATTACAACCCACGCATGGCAGACTGCAAACTTTGTTCCGCAAAAATATCAAGAGGTACAAAAAAGAGTACTTTATAATACGAGTAATCTGATAAAGCATCTGAAATTAAAACACAAAAGTGAGCACGGAAAATTTGCTGCTAGCAGTAACGTTAGCACTCGGCAACCAACCTGCAGCAAACCCTTGCAAGACGAGAGAAAATGGCAAGAGATAACCAAGAGCCATACAAATAATCCCACATCCCCCAAAAAAGTACAGGTATCGGTATCGGCGAATACCAGGAAAAAAATACCGGTACTCATACTCGGTCTTTAAAAAATGGTATCAGTGCATCCCTA

>scaffold_210000434-6

TATACTAGGGCTGCAGGATACCGAGAAAATATGCGATATTGTTGTTGAGTATTGCGATAACGATATTTCTTGCAATATAACATTTCCCTAGAGAAATGCTATTTTTATTAGCTATTTCGGACTAGCTCATCTTATCAACATTAGCTCGCTATGAAAGCTTCGCAGTTTGTCGATTGTCTTGCGTCAAAGCACGCACACGCAAATCCCAACTAACATCTGAGTGCCAGATATTTTACGATCCTAAAACTTTGACACACATTACTATTTGAAGTATTAAATCGCTCGTGCATATCTCGATATGCACATTTGCGATATTTCGATATATTGTGCAGCCCTAATATA

>scaffold_210000439-2

CAGGGCTGGGTAACCCATGTTTCTTAAGTTACAGTACTTTGTTCCGCTTGTTTTCCAGATACCCCTGCCCTACCCACTGCTGATTACCTGGTTCAGGTGTGTTCAGTCAATCAAAAGCTGGAAGTTACCATTTCAGATGACAGATAAGGGTGGAGTGGAGGGAGACCTGGTGATTTGGTATCTCCTAATCTCTGTATGGCTGGACACACCTAAACCAGGTAATCATCAGTGGGTAGGGCAGGGGTAGCTTGAAAACACGCGGAACAAAGTAACTTAAGGAACATGGGTTACCCAGCCCTG

>scaffold_210000439-4

TACACTATATAGCCAAAAGTTTGTGGACACCTTACCAGTACAACCACAGGAGTGTCTCACCAATTTGAAAGCACACAGTTTCTAGAAAGATAACGTTTAGAATGTCTCTGTATGTTATAGCATTACATTATTACTTCACTGGAACTAAGAGACCTAAACAGTGTTTCAGCATGACAATGAGCTTGTGCACAAAACCCCTGAGCTCTATGAAGACACGGTTTGCCAAAATGTGATGTGGAAAAAGTGAGTGTTCTTCACAGAGCCCTGATCTCAACATTACTGAACACCTTTGGGAAGAACTGGAACTCCTCACTCAACATTAGCACCTGATCTCACTAATACTATTGTGTCTAAATGGGGAAATTCCCAAAGCCATGCTCCAAAATCTAGTGGAAATCATTTCAAAAAGTGTGGAGGTTATTATAGGAGTAAGAGTCAGACTAAATCTGGAATAGGATGTTCAAAAACACATATGAATGTGATGGTCAGGTGTCCCCAATACTTTTGTCTGTATAGTGTA

>scaffold_210000440-2

AAGCCGGGCTTACACGGTGCGATTTTGGCCATGATTTGGTCGACTGAGACAAATTTTGAAATCCTAAAAGATTCCTATAATCCTACGCTAATATCTGTTGTCTTTGATCGCTAGTTTGACATGTTCACCGACAGCCCATTAATGGCAATCATTTTTTTCCTCCGGTGAAATTCTGGCAGTGTCAGAACTTTTTAGACGCTTACCTGCCTTGTGACTTCTCCTATGACGACTGTCAAACCAAGAACCAATAGGAGCGCCAAACCTGATGACGCAATTAGCACGAAAACTACAAACCGTTTCGAGAAAATGTGGAAACGAGTCGAGTGGACAGTACAGCAAGAAAACAAACTTATTGAGTCATAGAGGGAAAAAACGTTTGCATGACGTGTCGTCGCTGCTGTTTGTTTGATGCTAGCTACTATTTACCGAACTCGTCATGGATTGATGACGCAAAACTCCGGACGAGTTTTCTTGTGTGCGTCCGTTTTTGATGCATTTTGACTGTCGTACAGTCTGACATTATAACTGAGATCCTACCGTGTGACATGGAGATCATGTTCGTACAGTCTGACAAGCAACAGGACTATTAAAGGACTATTAAATATCGCACAGTGTGCGCCCGGCTT

>scaffold_210000441-1

AGATACACTATATTGCCAAAAGTTTGTGGGCACTTGACCATCACAAACATATGTGATTTTAAACATCTTATTCCAGATTTACTCCCCATTTTGCTGTTAGTTTAAGCTCCACTATTCTAGTAAGGCTTTCCACTACAATTTGTAGCTTGAATGTGAGATACGTGTTCATTCAGCTACAATAGTATTAGTGAGATCAAGCACTAATGTTGGGTAAGGAGGTCTGTGGTGCAGTCGGCGTTCCAGTTCATCCCAAAGGTGTTCAGTGGTGTTAAGATCAGGGCTTTGTGCAGAACACACAAGTTTTTACACTCCAATCACAACACACCATGTCTTAATGGAGTTCAGGGGTTTTGTGCACAGGGACATTGTCATGCTGGAACAGTGTTCGAGTCTCTTAGTTCCAGTGAAGGAATAATGTAATGCTACAACATACAAAGACCTTCTAGACAACTGTCCAAATTTGTAGCAACATTTTTCAGAGGAACCACATATGGATCTGATGGTCAGGTGTCCACAAACCTTTGGCAATATAGTGTATCT

>scaffold_210000441-2

TACACTATATTGCCAAAGGGTTGTGGAAACCTGACCATCACAAACATATGTGATTTAAACATCTTATTCCAGATTTACTCCCCATTTTGCTGTTAGTTGAAGCTCCACTATTCTAGTAAGGCTTTCCACTACAATTTGGAGCTTGAATGTGAGATACGTGTTCATTCAGCTACAATAGTATTAGTGAGATCAAGCACTAATGTTGGGTAAGGAGGTCTGTGGTGCAGTCGGCGTTCCAGTTCATCCCAAAGGTGTTCAGTGGTGTTAAGATCAGGGCTTTGTGCAGAACACACAAGTTTTTACACTCCAATCACAACACACCATGTCTTAATGGAACTCAGGGGTTTTGTGCACAGGGACATTGTCATGCTGGAACAGGGTTTAGATCTCTTAGTTCCAGTGAAGCTACAACATACAAAGACATTCTAGACAACTGTGTGCTTCCAACTTTGCGGCAGCATTTTGGATCAGATGGTGAGGTGTCCACAAACCTTTGGCAATATAGTGTA

>scaffold_210000441-10

TCAGGTCTCGCAAAAATGTAAAATTCCTGGTAGTCCTTGGGACAGGTACTGTTTGGTTTTTGGTAGCCCGAAATGAATTTAAATAGCCCGAATAAAAAAAAAAGAAGACATTTTTAATGTAGAATATTGTGAAGGAGACAAAACATCAAACAAAAACATTTTCAAAACACAAATAACAATCGTAGGCTATAAATTAACTAAATTACAATCATCAATACAAATATTTGGTTCTAACTAAACATCCTATTCCAGATGTCAGCGATTTTCACAATTCTCTCCACTGCGTATGCATGTATCTCTATCACATGTTTAAGCTTGCTGAGCGAGATTTCACTTGTCACATGAGGATAGTAATGACTGACAGGAAGACGTCAGAGGAATTGGTGAGCAACAGATCCGGAGAGTTACTGACAGATGTTGTGTAAAATCAGAAGGCTGTCTCTCCCTATACACAGTTTGCACGATCGCGGGTTGAAAGGGAAAGTAAAAAGCGAGCGCGCATTCAAATGCGATTTCAATCCACTACATTTTGATAGTGGCTTTCCGTTGCCCGAAAATTACAGACAGCATAATTACCCAACTATATTTGTAAGAAAATATGTTTTTCCTCCCTATAAGATAGCCCGACGGGCAGGGTGGGGATACATTTTGGTGGCTCACTGGAAGACACAATAGCCCCGGGACGTCGGGCTAGCGATTTTGCGAGCCCTGA

>scaffold_210000441-14

CTAGAGATGAGCAAGTCTGGATTCATCCAGGTTAAATAACCCGATTTTTTACATGACTCGGGATTCACGAATCCTTCTCCTCATTTACTCAGTTGAGTCTAATTTATTTTGTTACCAACAACAACATAGATAAATACAAACTGAAAATTACACCATTCAGTTGTTCTAAGTAGTTGTTAGATAGTAAATGTAGGAACAAGTAGCTGACTGAGTTGTTTAGGAAACTTCCTACATACTCAGAAACATAAGAGACTCAAAATTGCGGGTACTGATTCGTCGGACTCGTAGTGCGTAGCGAGTCCAAGGATTCAGTGGATTCTAGGGATTCGGTGAGTGCCATCTATTGGAATACCATGGCAAAATCAGGCCTTAAAGTGATTTAAAGGACTCGTGAGATTCGGATCCGTTCAATGAGTGATTCAAAAGGAGTTGAATCCGTAAATTGATTTGTATTTCTCATCTCTAG

>scaffold_210000442-1

ATACACTATATTGCCAAAAGTTTGTGGACACCCCCTATCAGATCCATATGTGGTTCCTCTCCAAAATGCTGCTACAAATTTGGACAGTTGTCTAGAAGGTCTTTGTATGTTGTAGCATTACATTATTCCTTTACTGGAACTAAGAGACTCGAACACTGTTCCAGCATGACAATGCCCCTGTGCACAAAACCCCTGAACTCCATTAAGACATGGTGTGTTGTGATTGGAGTGGAAAAACTTGTGTGTTCTGCACAAAGCCCTGATCTTAACACCACTGAACACCTTTAGGATGAACTGGAACGCCGACTGCACCACAGACCTCCTTACCCAACATTAGTGCTTGATCTCACTAATACTATTGTAGCTGAATGAACACGTATCTCACATTCAAGCTCCAAATTGTAGTGGAAAGCCTTACTAGAATAGTGGAGCTTCAACTAACAGCAAAATGGGGAGTAAATCTGGAATAAGATGTTTAAAATCACATATGTTTGTGATGGTCCGTTGTCCACAACCCTTTGGCAATATAGTGTAT

>scaffold_210000442-2

TACACTATATGGTCAAAAGTATGTGGACACCTTACCAACACTCTCACATTCTCCAAACTGTTTCCACGAAGCATAAATTTGTCTAGAATGTCTATGTATGTTGTAGCATTACATTATTTCTTCTCTGGAACTAAGAGACTCAAACACTGTTCCAACATGACAATGTCCCTGTGCACAAAGCCCCTGAACTCCATGAAGACATGGTGTGGAAGAACTTGAGTGTTTTGCACAGAGCACTGACCTCAACCCCACGAAACCTTTTGGGATGAACTGCACCACAGATCTCTTCACCTGACTTTAGTGCTTGATCTCACTAATATTATTGTGGCTGAATAAGAAAATCCCCACAGTCACACTCCAGAATCTAATAGAAAAGATTTCCAGAAGTGTGGAGGTTATTATAATAGTAAGAGTGAGACCAAATCTGGAATAGAATGTTTAAAAAACACATATGGATGTAATGCATAGGTGTCCCAATACTTTTGTCCATGAAGTGTA

>scaffold_210000442-4

AAATACACTATATAGACAAAAGTATTGGGACACCTGACCATCACATCTATAGGAACATCCTCTTCTTTTACTTTGACTCCTATAATAACCTCCACTCTTCTGGAAACGCTTTACACTAGATTGTGGAATGTGGCTGTGGGGGTTTGTGTTTATTCAGCTACAGTAGTATTCAGAAACTAATGTTGTGTAAGAATGTCTGGGGTGCAGTCTGTGTTCCAGTTCATCCCAAAAGTGTTCAATAGGGTCGAGTCAGACTGCAGGACACTCAAGTATTTCCACTTCAACCAGAACACACCATGTCTTAATGGAGTTCAGGGGCTTTGTGCACAGGGACATTGTCATGCTAGAACAGTGTTTAGGTCTCTTAGTTCCAGTGAAGGAATAATGTAATGCTACAACATATAGAGACATCCTAGACAACTGTGTGCTTTAGAGTTTGTGGTAACAAACACTAAGACACACTAATGTGGGTGTGCTGGTAAGTTGTCCAAAATTTGTTAGCAATATAGTGTATTT

>scaffold_210000446-11

TAGGGCTGTGTATTGGCAGGGGCCTCATGATACGATACATATCACGATACATGGGTCACGATACAATATATCACGATATATTGCAATACTTCCAAAAAAAAAGAAGGGAAAACAAACAGCTAAAAATGCAATTTGCTGTGTACCACCTGGAGGATTCTAGTATGTGTATCGCATTATATTGATCACTCAGCAGACAAATAAACACCGAGGGTTTTAATTAAATATTAACGTCAATGTAAATCAGTTTAAAACGTATTTAATTTATGCATCGATACTAGAGGTTAAAAATATCGATACACTAAGGCGATATCGCGATAGTTAGCTGTATCGATATTTTTACACAGCCCTA

>scaffold_210000450-1

CAGGACTGGGTAGCTCATGTTCCTTAAGTTACTTCATTACACTTGTGTTCCAGCTACCCCTACCATACTCACTGCTTATTACCTGGTTCAGGTGTGTTCAGTCAATCAGAAGCTAGAAGATACCATTTCAGATGAGGCTGGAGTGGGGAAAGACCTGGTGATTTGGTATCTCCTACCCTTTGATTGACTGAACACACCAGAACAAGGTAATCAGCAGTGGGTAGGGCAGGGGTAGCTGGAAAACAAGTGGAACAAAGTAACTTAAGGAACGTAAGTTACCCAGCCCTG

>scaffold_210000454-1

ATACACCATATAGCCAAAAGTTTGTGGACACCTGACCAGCACACTTACTTTGTCTTCCTGAAACCGTTGTCTCAAAGTTGTAAGGGCACAATTGTCTAATTATCTAGAATGTATTTGTTTTAGCATAACATTATTTCTTTACTGGCACTAAGAGACTCAAACACTGTTCCAGGATGAACAGTGTGCTCAAAGCCTGTGAGCTTTATGAAGCCATGGTGTGTTACGTTTGGAGTTAAAGAACTCGAGAGCCCTGACTCAACCCCACTAAACACGTTTGGGATGAACTGGAACACCGACTGCACCCCAGACCTCCTCACTCAACATTAGTGTTTGATTTCACTAATACTATTGTGGCTGAATGAACAAATCCCCACAGCCGTGCTCCACAGTCTAGTCAAAAGCCTTTTCAGAGGTGTGAAGGTTATTACAGTAGTAAAAGTAAGTCTAAATCTCAAACGGGATGTTCAAAAAACACATGTGGATGTGATGGTCAGGTGTCCCAAAACGTTTGACTCTATAGTGTAT

>scaffold_210000457-1

TTTAATATACAGGGTGGGTGAAAAGTAACTAGGCATTAAAAATTGGTAATAAAGTCCATATTTCTAATACAAATTTATTGAAACAAATTGTACATGTACTTTATTGCACAGAAAAATGAAAGCAAATCTTTAAATTTCAACGTAGGTGGCGTTAACCGGTTTTCTCAAACGGAGAGAAATGGGATGCACAGTTTTCTGAAGGAAGTCGTTTGGGATATTGCTGAGAACCTCCCGAATCCGTGTCTCCAAGTCCTCCAGTGTGCGAGGCTTGAAATCCTGTTTTTCTATCGTCTATTTACAGACTGCTTTCATCATGACTCCAGTGATACTACAAGTTCCATAGCCATCAAATATCGCCTAGTTAATTTTCACCCACGCTGTAGATAAA

>scaffold_210000461-1

ATACACTATATTGCCAAAAGTTTGTAGACACCTCCATATGTGGTTTCTCCCCAAAATGTTGCTACAAATTTGAAAGCACACAGTTGTCTAGAAAGTCTTTGTATGTTGTAACATTACATTATTTTTTCACTGGAACTAAGAGACCCAAACACTGTTCCAGCATGACAATGCCCCTGTGCACAAAGCCCCTGAACTCCATGAAGACATGGTGTGTTATGATTGGAGTGGAAAAACTTGAGTGTTCTGCACAAAGCCCTGACCTTAACACCACTGAACACCTTTGGGATGAACTGGAACACCGACTGCATCACAGACCTCCTTACCCGACGTTAGTGTCTGATCTCACTAATACTATTGTAGCAGAATGAACACATATCTCACATTCAAGCTATAAATTGTAGTGGAAAACCTTACTAGAAGAGTGGAGCTTAATCTAACAGCAAAATGGGGAGAAAATCTGAAATAAGATGTTTAAAATCACAAATGTTTGTGATGGTCAGGTGACCACACACTTTTGGCAATATAGTGTAT

>scaffold_210000461-2

ATATTGCCAAAAGTTTGTGGACACTTCACCATCAGATCCATATGTGGTTCCTCTCCAAAATGTTGCTACAAATTGGGAAGCACACAGTTGTCTAGAAAGTCTTTGTATGTTGTAACATTACATTATTTCTTCAGTGGAACTAAGAGACCCAAACACTGTTCCAGCATGACAATGCCCCTGTGCACAAAGCCCCTAAACTCCATGAAGACATGGTGTGTTATGATTGGAGTGGAAAAACTTGAGTGTTCTGTAAAAAGCCCTGACCTTAACACCACTGAACACCTTTGGGATGAACTGGAACACCGACTGCACCACAGACCTCCTTACCCGACGTTAGTGTCTGATCTCACTAATCCTATTGTAGCTGAATGAACACATATCTCACATTCAAGCTATAAATTGTAGTGGAAAACCTTACTAGAAGAGTGGAGCTTAATCTAACAGCAAAATGGGGAGAAAATCTGAAATAAGATGTTTAAAATCACATATGTTTGTGATAGTCAGGTGACCACACACTTTTGGCAATAT

>scaffold_210000461-3

TACACTATATGGACAAAAGTATTGGGACACATGACCATCACATCCATATGTGTTTTAAAAAAAATCTATTTTAGCTTTAGTCTTACTTTTACTCCTATAATAACCTTCACGTTTTTAAAAAGACTTTCCACTAGATTTTGGAGTGTGGCTGTGGGAATTTGCTTATTCAGTCACAAAAGTATTAGTGAGTTCAGACACTAATGATGGGTGAGGAGGTCTGGGGTGGAGTCGGTGTTCCAGTTCATCCCAGAAGTGTTCAGTGGGGTTGAATTCAGGGCTCTGTACAATTTTAACACATGAACCATAACACATGGTCTTAATGTAACTCAGGTTCTTTGTGCACAGGGGCATTGTCATGCTGGAACAGTGTTTGGGACTCTTAGTTTCAGTGAGGGAATAATATAATGTTACAACATACAAAGACATTCTAGACAACAGTGTGCTTTCAAATTTTTGAAAACAGTTTGGGAGGACACACATGTGGGTGTGCTGGTAAGGTGTCCACTTTTGGCTATATAGTGTA

>scaffold_210000461-5

TACCCAGCTAACAGAGTGCGTTCCCACAACTTTCCATAATGTTCTTCAAAAGTTGAAAAATTCTTAGGACAAACTGTTCTTAAAATAATGTTCATGGACTGTTCTCATAATATTATTAATAGTTAATATATTTTCTTGTGATATTGAGAGAAACTTTCTTCATGGAAAGCCATCATGAAGTTATTCATACTTGTTATTCATACTTATTCATAATATTGAAAGAACACGTTCTTAGAGCAACACTATAGAATAATGTTCTACCAACGTTAAGCAAACTGGACATTTTCATGTTTTTGGTACAATTCAAATCATGTTCCCAAAACCAAAACAGAACGTTTGAAAAAATGTTGTCAGAACATAAATTTGTTAGCTGGGTA

>scaffold_210000462-1

ACATACACTATATTGTCAAAAGTTTGTGGACACCTCACCATCAGATCCATATGTGGTTTCTCTCCAAAATGTTGCCACAAATTGGGAAGCACACAGTTGTCTAGAAAGTCTTTGTATGTTGTAACATTACATTACTCCTTCAGTGGAACTAAGAGACCCAAACACTGTTCCAGCATGACAATGCCCCTGTGCACAAAGCCCCTGAACTCCATGAAGACATGGTGTGTTATGATTGAAGTGGAAAAACTCGAGTGTTCTGCACAAAGCCCTGACCTTAACACCACTGAACACCTTTGGGATGAACTGGAACACCGACTGCACCACAGACCTCCTTACCTGACGTTAGTGTCTGATCTCACTAATACTATTGTAGCAGAATGAACACATATCTCACATTCAAGCTATAAATTGTAGTGGAAAAACTTACTAGAAGAGTGGAGCTTAATCTAACAGCAAAATGGGGAGAAAATCTGAAATAAGATTTTCAAAATCACATATGTTTGTGAAGGTCAGGTGTCCACAAACTTTTGCCAATATAGTGTATGT

>scaffold_210000462-2

TATTGCCAAAAGTTTGTGGACACTTCACCATCAGGTCCATATGTGGTCCATCTCCAAAATGTTGCTACAAATTTGGAAGCACACAGTTGTCTAGAAAGTCTTTGTATGTTGTAACATTACATTATTTCTTCACTGGAACTAAGAGACCCAAACACTGTTCCAGCATGACAATGCCCCTGTGCACAAAGCCCCTGAACTCCATGAAGACATGGTGTGTTATGATTGGAGTGGAAAAACTTGAGTGTTCTGTAAAAAGCCCTGACCTTAACACCACTGAACACCTTTGGGATGAACTGGAACACCGACTGCACCACAGACCTCCTTACCCGACGTTAGTGTCTGATCTCACTAATCCTATTGTAGCTGAATGAACACATATCTCACATTCAAGCTATAAATTGTAGTGGAAAACCTTACTAGAAGAGTGGAGCTTAATCTAACAGCAAAATGGGGAGAAAATCTGAAATAAGATATTTAAAATCACATATGTTTGTGATGGTCAGGTGACCACACACTTTTGGCAATA

>scaffold_210000462-4

TAGGGATGCACCGATAAGATTTTTTAAAGACCCAAAACCAGGAAATATTTTTTTCCTGGTTCTCGTCGATACCTTGGGGATGTGGAATTATTTGTATGGCTCTCTGATTATCTTTTGCCATTGTCTTCCAAGAGTTTGCTGCAGTGTTGGTTGCCGAGTGCTAACTTTACTGTTAGCGGCAAATTTTTCGTGCTCACTTTTGTGTTTTAATTTCAGATGTTTGATCAGATTACTCGTATTATAAATAAAAAATTTTGATATTTTTGCGGAACAAAGTTTGCAGTCTGCCATGCGTGGGTTGTCATCATTCATTTTGAAATATTTCCACATTGCTAAGCCTGACATTCTGTCGCTGCTGCCGGAGTCTTTGTGCACTGAAAGTTCTGTCAATGAAGTCACAAGAGGTATCGGTCTGTGTTTTTACTAATACGAGTACATGAGCTCAGTATCGGGCCCGATACCGTTACCAGTATCGGTGCATCCCTA

>scaffold_210000463-7

TAGGGCTGTGTAAAAATATCGATACAGCTAACTATCACAATATTTTTTTTCACGATAGTGTATCGATATTCTAACCTCTAGTATCGATACATATTTCAATTACGTTTTAAACTGATTTATGTTTACGTTAATATTTAATTAAAACACTCCGTGTTTATTTGTCTGCTGAATAATCATTATAATGTGATGCACCTACCCAAGTGCTACACAGCACGTATTTTTAGCTCTTTATTTTACTTTTTTTTTGTATTGTATTGTATTACAATCAAGTACTGTAATGTATTGCAATATGTATTGTATTGCTATATATTGTATCGTGACCCATGTATTGTGATATGTATCGTATCGTGAGGCACTTGCCAATACACAGCCCTA

>scaffold_210000465-2

TATGTACAGGGTGGGTGAAAATGAACTGGGCAATATTATATGGCTATAGAACTTGTAGTATCACTGGAGTCATGATGAAAAAAATATCTAAATAGATGATAGTAAATCGGATTTCAAGCCTCGCACACTGGAAGACTTGGAGGCACGGATTCAAGAGGTTCTCAGCAATATCCCAAACAACTTCCTTCAGAAGACTGTTCATTCCATCGTTTAAGGAAAATGGTTAACGCCACCAGTGCCTACATAAAAATGTAAAGATTTTGTTTTCCTATGTAATAAAATACATGTACAATTTGTTTCAATAAATTTGTATTAGAAATATGAACATTATTACCAATTTTTAATGCTTAGTTGCTTTTCACCCACCTTGTATATA

>scaffold_210000468-1

CTTAAACCGGGTTCACACTGTGCAATTTTAGCCACTATTTGGTTGTCTGAGACAAATTTTGAAATCCTAAAAGATTCCTATATTCCTATGTTAAAATCTGTTGTCTTTGATGGCTAGTTTGGCATGTTCACCGACAGACGATTAATGGCCGTTGCAATAATTTTTTTCCTCCGATGAAATTCTGGCAGTGTCAGAAGATTTCAAACACTTATTGTACCTGTAGTGACAACGACCGTCAAACCAAGAATCAATAGGATCGCAGAACCTGATGACGCAATTAGCACGACAACTTCAATTCACCTCGGGAAAATGTCTAAACGAGTCCGGTGGACAGAACCGTAAGAAAGTAAGCAAATTTAACGCTACAGATTGTTTTTGTTTGCTGCTAGCGAGAGAACGTGAGAGTTTAGCGAGAGAACGCGGGTTACTGAACTTGTCACGGATTGATGACGTCCGTTTTTGATGCGTTTTGACTGACAGACAGTCTGAAATTATGACAACTGAGATCCTACAGGAGGATCAGATTCGTACAGTCTGACAAGCAACAATCGCAAAAGACTATTAAAAATCGCACAGTATAAACCCGGCTTAAG

>scaffold_210000471-6

ACTAGAGATGAGCGAATCCGGATTCACCCCGGTTAAATAGCCCAATCCTTTATAGGACTCTGGATTCAGGAATTACTCGGATTAGTTGAGTCCTAAATTCACTTGTTGCCAACAACATAGCTAAATACAAATTAAAAATTACATTATTATAATAGTTGTTAGCTAGTAACTTTAAGAACAAGTAGCTGACTGAGTTGTTTAGGAAACTTCCTACATGGTCAGAAACATCAGAGACTCAAACTTGTGGATACTGATTCATCTGAACCACGGAATCCGTCAGACTCGTAGCGTGTAGTGAAACAGCCGAATACGAGGGAGTCAGTGTGTGTGTGTGTGTGTGTGTGTGTGAGAGAGATTCATGAGTCCAAGGATTTGGTGGATTCTAGGGATTCGGTGAGTGCCCTCTAGTGGAAAGACCATGCCAAAAGCAGTCAGTATCCCTACTGACTCAAGTAGCTCAAAGGACTCGTGAGACTCGGATCCGTTCAATTATTGATTCAGAAAAATTCGAATCCGTAAATGGATTCGGATTTCTCATCTCTAGT

>scaffold_210000471-9

TAGGGCTGCACGATACTGGGAAAATATGCGATATTGTTGTTGAGTCTTGCGATAATGACATTTCTTGCGATATTACATTTCCCTAAAGAAATGCTATTTTTATTAGCTATTTTAGCTGCGGGTGTTTTTCGGGCCGGCTCATCTTATCAAGATTAGCTCGCTATGAAAGCTTCTCAGCTTGTTGATTGTCTTGTGTCAAAGCACGCACACGCAAATCCCAACTAACATTCGCATGCCAGATATTTTACTTTGACCACAAATTACTATTTGAAGTATTAAATCGCTCATTTATCGCAAACCATTGCGATATGCATATCTCGATATGCACATTTGCGATATTTCGATATATTGTGCAGCCCTA

>scaffold_2200001-6

ATATACACTCTATAGCCAAAGGTTTGTGGACACCTTACCAGCACAAATGTGTCTCCACCAAAACTGTTGACACAAACTCTGTCACACAGGTGTCTAGAACATCTTTGTATGTTGTAGCGTTACATTCTTCCTTCACTGGAACTAAGAGACTTGAATCCTGTTCCAGCATGACAATGCCCCTGTGCACAAAACCCCTGAGCTCCATGAAGACATGCTGTGTTCTGGTTGGAGTGGAAGAACTCTCATGTCCTGCACAGAACCCTGACTCTGACTCAACCCCACTGAACACCTTTGGAATGAACTGGAACCCCGACTGCACCCCAGACCTCCTCCCCCAACATTAGTGCCTGATCTCACTAATACTATTGTAGCTGAATGATCAAATCCCCACAGTCACATGACCCAATCTAGTGGAAAACCTGCACAGAAGTCTGGAGGTGATTATAGGAGGAAGAGGGAGACGAACATCTGGAATAGGATTATCAAAAAACATGTGATAATCAGGTGTCCTAATATTTGTGAATATAGTGTATAT

>scaffold_2200001-8

TTAAGCCGGGTTTACTCTGTGTGATGTTTATTAGTCTTTTACGATTGTTGTTTGTCAGACTGTACGAACATGATCCCCGTGTAGGATCTCAGTTGTCATGATGTCAGACTGTACGACTGTAAAGACGTGTGACACACACGAGAAGACTCGTCCAGAGTTTTACGTCATCAATCCGTGACACGTTCAGTAACCCGCGTTCTCTCGCTAAACGGTAGCTAACAGCAAACAAACAGCAGCGACGACACGTCATACAAACTCTCCTTTTCCCTCCATAACTCAATAAGTTTCTCTTCTTGCTGTTTCTGTCCACCGGATTCGTTTACACATTTTCCAGAGGTGGTATGTAGTTGTGGTGCTAATTGTGTCATCAGGTTCGGCGCTCCTATTGGTTCTTCGTTTGACGATCGTCGTTGGAGAAGTTACACTGCAGGAAAGTGTGTGAAATCTTCTCACACTGCCAGAATTTCATCAGAGGAAAAAAGTGATCATAACGGACATTAATCGTCTGTCAGTGAACACGTCAAACTAGCCATTAAAGACGACACATTTTAGCGCAGGTTTATAGGACTCTTTTAGCTTTTTTTTTTTTTGGGCTCAGACGAACAAATCATGGCCAAAATCGCCCAGTGTGAACCCGGCATAA

>scaffold_240000-1

TCTACAGGGTGGGTGAAAATGAACTAGGCAATATTTAATGGCTATAGAACTTATATTATCACTGGAGTCATGAAAACAGTCTCTAAATAGTCAATTGGATTTCAACTTTGAGGAATTGGAGGCACGGATTCGGGAGGTTCTCAGTAATACTTTATCCCAAACGAATTCCTTCATTAGACTGTGCATTCCATCTCCAGCTGTTTGAGGAAACTGGTTGACGCTACGTTGAAATTTAAAGATTTGCTTTCATTTTCCTATGTAATAAAGTACATGTACAATGCGTTTCAATAAATTTGTATTAGAAATATGGACTTTATTACTATAATTTTTTATGCCTAGTTACTTTTCACCCACCCTGTAGA

>scaffold_2500004-1

TAGGGATGCACCGATACATTTTTTTAAAGACAGAGTACGAATACCGATATTTTTTCCCCGGTACATACCGATACCTGTACTTTTTTGGGATGTGGGATTATTTGTATGGTTCTCTGGTTTTCTCTTGCCATTTTCTCTCGTCTTGCACGGGTTTACTGCAGGGTCGGTTACCGAGTGCTAACTTTATTGCTAGCGACAAATTCTTCATGCTCACTTTTGTGTTTTAATTTCAGATGTCTTATCAGATTACTCGTATTATAAGTACTTATTTTTGTACCTCTTGATGCGGAACAAAGTTTGCAGTCTGTCGTGCGTTGGTTCATCATTAATTTAGAAATATTTCCACACTGCTGAGTCTGACATTCTGTCTCTGCTGCCGGAGTCTTTGCGCACAAAGTTTTGTAAGAGATATCGGTCTTTGATATCGGAGTGTTTATACGAGTACGAGTACATGAGCTCAGTATCGGTATCGGTGCATCCCTA

>scaffold_2500007-6

TAGGGATGCACCAGTATTGGTATTGGGCCCGATACCGAGCTCATGTACTTGTATTTGTGAAAATACTCCGATACCAAAGACCGATACCCCTTGTGACGTGATTGGCAGAACTTTCAGTGCACAAAGACAGAATGTCAGACTCAGCGGTGTGGAAATATTTCAACATTAATGATGAACCCACACATGGCAGACTGTAAACTTTGTTCCGCATCAAGCGGTACAAAAATGAGTACTTATAATACGAGTAATCTGATAAAACATCTGAAATTAAAACACAAATGTGAGCACTAGCAATTAGCTGCTAACAGTAACGTTAGAATTTGCCAACCCAAACCCGGGCAAGATGAGAGAAAATGGCAAGAGAAAAAACGAGAACCATATAAATAATCCCACATCCCAAAAAATACAGGTATCGGCATGTACCGGGAAAAAAAGATCGGTATTCATACTCGGTCTTTAAAAAATGGTATCGGTGCATCCCTA

>scaffold_2500007-8

TAGGGATGCACCAGTATTGGTATTGGGCCCGATACCGAGCTCATGTACTTGTACTTGTGAAAATACTCCGATACCAAAGACCGATACCCCTTGTGACGTGATTGGCAGAACTTTCAGTGCACAAAGACAGAATGTCAGACTCAGCGGTGTGGAAATATTTCAACATTAATGATGAACCCACACATGGCAGACTGTAAACTTTGTTCCGCATCAAGCGGTACAAAAATGAGTACTTATAATACGAGTAATCTGATAAAACATCTGAAATTAAAACACAAATGTGAGCACTAGCAATTAGCTGCTAACAGTAACGTTAGAATTTGCCAACCCAAACCCGGGCAAGATGAGAGAAAATGGCAAGAGAAAAAACGAGAACCATATAAATAATCCCACATCCCAAAAAATACAGGTATCGGCATGTACCGGGAAAAAAAGATCGGTATTCATACTCGGTCTTTAAAAAATGGTATCGGTGCATCCCTA

>scaffold_2500009-45

TAGGGCTGCATGATACTGGGAAAATATGCGATATTGTTGTTGAGTAATGCGATAACGATATTTCTTGCGATATAACATTTTTCTAGAGAAATGCTATTTTTATTAGCTATTTTAGCTGCGGGTGTTTTTCAGGTTGGTTCATCTTATCAAGATTATCTCGCTATGAAAGCATCGTGGTTTGTTGATCGTCTTGTGTCAAATCACGCGCACGCAAATCCCAACTAACATCCGCGCCAGATATTTTACTATCCTAAAACACACATTAATATTTGAAGTATTAAATCGATCATTTATCATTAACCATTGCTATATGCACATTTGCGATATTTCGATAATTTCGATATCCTGTGCAGCCCTA

>scaffold_270000-16

TTAAGCCATGTTTACACTGTGTGATAATCTTTTAAGATTGTTGTTTGTCCGACTGTACGAACATGATCCCCGTGTCACACTGTAGGATCTCAGGGGTCATAATGTCAGACCAAACACGTCAAAAAGAAGACGACTCGTCCGGAGTTTTACATCATCAATCTGTGACACGTTCAGTAACCCGCGTTCTCTCGCTAAACGGTAGCTAACAGCTAACAAACAGCAGCGATGACACGTCATATAAACACTCCTTTTCCCTCTATAACTCAATAAGTTTCTCTTCTTGCTGTTCTGTCCACCGGACTCGTTTCCACAATTTCCCGAAATGATTTGAAGTCACGCTAATTGCGTCATCGGGTTCGGCGCTCCTGTTGGTTCTTGGTGTGACGGTCGTCGTAGGAGACGTCACACTGCAGGAAAGTGTCTGAAATCTTCTCACACTGACAGAATTTCATCGGAAGAAAAAGCTGTTTGCAACGGTCATGAATCGTTTGTCGGTAAACACTCGCCATCAAACACCACAGATTTTAGTGCAGGATTATAGGAATATTTTAGGAATCAAAATTTGTCCCAGACGAACAAATCATGGCCAAAATTGCAGAGTGTAAACCCGGCTTAA

>scaffold_270000-37

TAGAGATGAGAAATTTGAATCCATTTACAGATTCTAATTTTTCTGAATCACTCATTGAACGGATCCGAGTCTCACGAGTCCGTTGCGTCACTTGGAGTCAGTAGGGATTCTGACTGCTTTTTCCATGGTATTCCACTAGAGGGCACTCAACAAATCCTTGGAATCCACCGAATCCTTGGACTCATGAATCTCTAACACACTGAGTCGCTCGGATCCGGCTGTTTTGCTACGCGCTACGAGCCCGAAGGATCCGCTAAATTCTGTACCGAATCAGTACTCGTGATTTTGAGTCTCTTATGAGCATGCAGGAAGTTCCCTAAACAACTCAGTCAGCTACTTGTTCCTACAGTTACTAGCTAACAACTACTTAGAACAACGGAATGGCGTCGTTTTCAGTTTGTATTTATCCATGTTGTTGTTAGCAACAAGATAACTTAGGACACAACTGATCCGAGTAAATGAAGAGAAGGATTCGTGAATCCCGAGTCATATAAAGGATCGAGTTATTTAACCCGGATGAATCCGGATTCGCTCATCTCTA

>scaffold_270000-71

TAGAGATGAGAAATTTGAATCCATTTACAGATTCTAATTTTTCTGAATCACTCATTGAACGGATCCGAGTCTCACGAGTCCGTTGCGTCACTTGGAGTCAGTAGGGATTCTGACTGCTTTTTCCATGGTATTCCACTAGAGGGCACTCAACAAATCCTTGGAATCCACCGAATCCTTGGACTCATGAATCTCTAACACACTGAGTCGCTCGGATCCGGCTGTTTTGCTACGCGCTACGAGCCCGAAGGATCCGCTAAATTCTGTACCGAATCAGTACTCGTGATTTTGAGTCTCTTATGAGCATGCAGGAAGTTCCCTAAACAACTCAGTCAGCTACTTGTTCCTACAGTTACTAGCTAACAACTACTTAGAACAACGGAATGGCGTCGTTTTCAGTTTGTATTTATCCATGTTGTTGTTAGCAACAAGATAACTTAGGACACAACTGATCCGAGTAAATGAAGAGAAGGATTCGTGAATCCCGAGTCATATAAAGGATCGAGTTATTTAACCCGGATGAATCCGGATTCGCTCATCTCTA

>scaffold_300000-22

ACAGGGAGGGTGAAAAGTAACCAGACATTAAAAATGGGTAATAAAGTCCATATTTCTAATACAAATTTATTGAAACAAATTGTACTTTATTCCATAGAATCTTTAAATTTTCAACGTAGGTGTCAACCAGTTTCCTCAAACGGGCCGGAGACGGAATGCACATCCTTCTGAAGGAAGTCGTCTGGGAAATTGCTGAGAAGAGAACCTCCTGAATCCGTGCCTCCGAGTCCTCCAGTGGGCGAGGCTTGAAATCTTATTAACCATCATCTATTTACACACTGTTTTCATCATGACTCCAGTGATACTACAAGTTCTACAGCCATTAAATATGACCTAGTTCATGTTCACCCACCCTGT

>scaffold_3000002-12

AATAAACAATTACATTGATAGTCGATTAATTATCGATTATTAGAGCGATTAATCGATTAATCAGCAATTATTTAATCTCGTGCAGTCAGGTAAATTATAGTTATTCCTATTGTAACTGATGAATAAAGCAAGGAAATTACTTCAACATTTAAAAATGTCTTTTTATTAGGCCTGTAATTATTTATT

>scaffold_3000005-3

GCTAAAGCCGGGTTTACACTGTGTGATTTTTAATAGTCTTGCGATTGTTGTTTGTCAGACTGTACGAACATGATCCCCGTGTCACACTGTAGGATCTCAGTTGTCATAATGTCAGACTGTACGACAGTCAAGACGCGTCATAAACGGACACACAAAAAATCTCGTCTGGAATTTTACATCATCAATCCGTGACACGTTCAGTAACCCATGTTCTCTCTCTAAACGGTAGCTAACAGCTAACAAACAGCAGCGACGACACGTCATAAAAACCCTCCTTTTCCCTCCATAACTCAATAAGTTTCTCTTCTTGCTGTTCTGTCCACCAGACTCGTTTCCACATTTTCCCCAGGTGGTTTGGAGTTGTAGCTCTAATTGCGTCATCAGGTTCGGCGCTCCTGTTGGTTCTTGGTTTGGCGGTTGTAGTAGGAGACGTCACACTGCAGGAAAGTGTCTGAAATCTTCTCACACTGCCAGAATTTCATCAGGGGAAAAAACTGATCGTGACGGTCATTAATCGTCTGTCGGTGAACATGTTAAACTCGCCATCAAACACAACAGATTTTAGCGCAGGATTATAGGAATCTTTTAGGAATTCAAAATTTGTCTCAGGCAACCAAATCGTGACCAAAATCACCCAGTGTGAGCCCGTGATTTAGC

>scaffold_3000005-4

GCTAAAGCCGGGTTTACACTGTGTGATTTTTAATAGTCTTGCGATTGTTGTTTGTCAGACTGTACGAACATGATCCCCGTGTCACACTGTAGGATCTCAGTTGTCATAATGTCAGACTGTACGACAGTCAAGACGCGTCATAAACGGACACACAAAAAATCTCGTCTGGAATTTTACATCATCAATCCGTGACACGTTCAGTAACCCATGTTCTCTCTCTAAACGGTAGCTAACAGCTAACAAACAGCAGCGACGACACGTCATAAAAACCCTCCTTTTCCCTCCATAACTCAATAAGTTTCTCTTCTTGCTGTTCTGTCCACCAGACTCGTTTCCACATTTTCCCCAGGTGGTTTGGAGTTGTAGCTCTAATTGCGTCATCAGGTTCGGCGCTCCTGTTGGTTCTTGGTTTGGCGGTTGTAGTAGGAGACGTCACACTGCAGGAAAGTGTCTGAAATCTTCTCACACTGCCAGAATTTCATCAGGGGAAAAAAACTGATCGTGACGGTCATTAATCGTCTGTCGGTGAACATGTTAAACTCGCCATCAAACACAACAGATTTTAGCGCAGGATTATAGGAATCTTTTAGGAATTCAAAATTTGTCTCAGGCAACCAAATCGTGACCAAAATCACCCAGTGTGAGCCCGTGATTTAGC

>scaffold_3000006-14

TTACACTGTGCAATTTTTAATAATCTTTTGCGATTGTTGTTTGTCAGACTGTACGAACATGATCCTCATGTCACACTGTAGGATCTCAGTTGTCCTAATGTCAGACTGTACGACAGTCAAGATGCATCAAAAACGGTCACGCACACAAGAAAACTCGTCCGGAGTTTTACATCATCAATCCGTGACACGTTCGGTAATCCGCGTTCTCTCTCTAAACGGTAGCTAGCAGCAAACAAAAACAATCTGTAGCGTTAATGTTGACATACATGACATACATCTCTTTTCTTGCTGTTTCTGTCCAATGGACTCGTTTCCACATTTTTCCGAGGTGGTTTGAAGTCGTCGCGCTAATTGCGTCATCAGGTTCTGCGCTTCTATTGGTTCTTGGTTTGACGGTCATCTTAGGAGACGTCACACTGCAGGAAAGTGTCTGAAATCTTCTCATATTGCCAGAATTTCATCAGAGGAAAAAACTTATTGCAACGGCCATTATGAAACATGTGAATCATGTGAAACTAGAATCTTTTAGGATTTCAACATTTGTCTCAGATGACCAAATCAAGGCCAAAATCACACAGTGTAA

>scaffold_3000006-16

TTACACTGTGCAATTTTTAATAATCTTTTGCGATTGTTGTTTGTCAGACTGTACGAACATGATCCTCATGTCACACTGTAGGATCTCAGTTGTCCTAATGTCAGACTGTACGACAGTCAAGATGCATCAAAAACGGTCACGCACACAAGAAAACTCGTCCGGAGTTTTACATCATCAATCCGTGACACGTTCGGTAATCCGCGTTCTCTCTCTAAACGGTAGCTAGCAGCAAACAAAAACAATCTGTAGCGTTAATGTTGACATACATGACATACATCTCTTTTCTTGCTGTTTCTGTCCAATGGACTCGTTTCCACATTTTTCCGAGGTGGTTTGAAGTCGTCGCGCTAATTGCGTCATCAGGTTCTGCGCTTCTATTGGTTCTTGGTTTGACGGTCATCTTAGGAGACGTCACACTGCAGGAAAGTGTCTGAAATCTTCTCATATTGCCAGAATTTCATCAGAGGAAAAAACTTATTGCAACGGCCATTATGAAACATGTGAATCATGTGAAACTAGAATCTTTTAGGATTTCAACATTTGTCTCAGATGACCAAATCAAGGCCAAAATCACACAGTGTAA

>scaffold_3000007-5

TAGGGCTGTGTAAAAATATCAAAACAGCGAACAATTTTTTTTTCACGATAGTGTATCGATATTCTAACCTCTAGTATCGTTACATCTTTAAATTACGTTTTAAACTGATTTACGATAATATTTAATTAAAATATTTGTATGATGAGTAATCAATATAATGTGATACACATACTAGAATCCTCCAGGTGGCACACAGAAAGTTGTAGTATTTTTAGCTATTTGTTTGACTTTTTTTAATGTATTGCAATATATACTGTGATATATTGTATCATGATATGTATCGTATGGTGAGGCCCTTGCCAATACACAGCCCTA

>scaffold_3000007-8

TATTAGAGCTGCACGATACTGGAAAATATGCGATATTGTTGTTGAGTATTGCGATAACGACATTTCTTGCGATATAATATTTCCCTAGAGAAATGCTATTATTATTAGCTATTTTAGCTGCTGGTGTTTTTCAGGCTGGTTCATCTTATCAGGATTAACTCGCTATGAAAGCGTCGCAGTTTGTTGATCGTCTTGCGTCAAATCACGCGCACGCAAATCCCAACTAACATCCGCGCGCCAGATATTTTACTATCCTAAAACTTTAACCACACGTTAATATCTAAAGTATTAAATCGCTCATTTATCGCAAACCATTTGCGATATTTATATATCGATATGCACGTCGTTCGCGATATATTTTTCGATAATTTCAATATATTGTGCAGCCCTAATA

>scaffold_3000009-1

TACGGTGGCAGAGAGAGCTCAACGCGCTGCAATTTAAGAAAACACATAAAAATAGAAAAAAACACCAACAAATTAAGAAAACAATTGACAACACACGTGCTGCAAATCCTCACAACACAACCAAATGCAGGAACGCGCTGCAAATAGCACAGATCACAACAGAAATGTTTCATGGGACCCCAAAAAGTGAGGAACCCGGCTGGGACCTGTTTATTGTTCAGTGGCTATTTGTTGATTGTGTTAACGGTGAACTGAAAGGCAAGTTTTTTGTTTATTTTATCATCCCGATTGTATGATTCCTTGGTCTTTTGGATATTATTAGCCTAAAAAATCTGACGAATTACACAGTTAACTGTGAAACGATAAACAGGTCGACAGGTCCCAGCCGGGTTAGTCACTTTTTTGGGGGTCCCTTGAAACATTAATGTTGTGGTCTGTGCTATTTGCAGCGCGTTTCCGTATTTGGTCGTGATGTGCGGATTTGCAGCGCGTTTCCGTATTTGGTCGTGATGTGCGGATTTGCGGATGTGTGTTGTCAAACTGATGATGTTTTCTTAATTTGTTGGTATTTGCATGCGTTTCCATAAATTGCAGCGCGTTTAGCTCTCTGTCGGCCACCGTA

>scaffold_30000010-7

TAGGGCTGTGTATTGGCAAGGGCCTCACGATACGATACATATCACGATAAATCGGTCACGATACAATATATCACAATATATCGCAATACATGGAAAAGAAAAGAAAAAAGGCAAACAAATGGATAAAAATACAACTTGCTGTGTGTCACCTGGGGGATTCTAGTATGTGTATCACATGATATTGATTACTCCACAGACAAATAAACACTGAGGGTTTTAATTAATTAACGTAAGTAAGTTTAAAACTATTTAAATACGTATCGATACCAGAGGTTAGAATATCGATACTCTATCGTGAAAAAAAATATCGTGGTAGTTAGCCGTATCGATATTTTTACACAGCCCTA

>scaffold_30000011-2

TATGGGCAAAAGTTTGTGGACACCTTACCAGCACACCAATATAGGTGTGTCTTTTCCAAACTGTTCCACAAAATCTGAAGCACACAGTTGTCTAGAACGTCTTTGTATGTTGTAGCGTTACATTATTCCTTCACTGGAACTAAGAGACTCGAACCCTGTTCCAGCATGACAATGCCCCTGTGCACAAAGCCCCTGAGCTCCATGAAGACCTGCTGTGTTCTGGTTGGAGTGGAAGAACTCTCATGTCCTGCACAGAGCCCTGACTCTGACTCAACCCCACTGAACACCTTTGGGATGAACTGGAACCCCGACTGCACCCCAGACCTCCTCCCCCAACATTAGTGTCTGATCTCACTAATACTATTGTAGCTGAACGATCAAATCCCCACATGACCCAATCTAGTGGAAAACCTGCACAGAAGTCTGGAGGTTTAATAGGAGGAAGAGTGAGACTAACATCTGGAATAGGATAATCAAAACCACACATATGGATGTGATGGTCAGGTGTCCTGATAATTTTGTCCATA

>scaffold_30000014-4

TACAGTGAAATCTCTGCAAACGAATGTCCTCCCTATCAAATTTTCGCCTTAAGAATTGAAATTTTTCAAGAAATTTGCATCGGCATACGAACCATTTTTGGCAATACGAACGAACCGCACCGCACCGCACGCCGGCTAAGTTGCGCGTACGCAAACATTTTTGTGTCAGTGGAAAAGCCAAGCCAAGCGAGTTTACAAATTTTTCAGGTTATTTTTAGTCAGAAAAAGCTGTTTCAAAATAGTTACCCACGATACCGACGTTGCCTTTGGCATACGTTCAAAAGCTATGTGATTTTTTGCTCGTTTATCCTTTGCCAGATTGGTGGCGTGGATCTATTTATTTCATATTTTACTTAATTTCCACGTCTTTTCTAAAAAAAATTTTTTTTTTATTTGCAAAAAATATGATTATAGTGTTGGAAATTGGTAACAATGGTAATATTTTTGGGTGCCTGGAACGGATCATCTGCATTTATGTGATTTCGTATGGGAAAATTCGTATCGCAATACCAATTTTCGCCTTAAGAACTTGCCTCCAGAATGGACTCAATTCATATGCAGAGGTTCCACTGTA

>scaffold_30000014-18

TAGGGCTGCACAACATATCGAAATTATCGAAATATCGCAAATGTGCGTATCAAGATATGCATATTGCAATGGTTTGCGATAAATGAGCGATTTAATACTTGAAATATTAATGTGTGGATAAAGTTTAAGGATAGTAAAATATCTGGCGCGCGGATGTTAGTTGGGATTTGCGTGCGCGTGATTTGACGCAAGACGATCAACAAACTGCGAAGCTTTTATAGCGAGTTAATCTTGATAAGATGAACCAGCCTGAAAAACACCAGCAGCTAAAATAGCTAATAAAAATAGCATTTCTCTAGGGAAATGTTATATCGCAAGAAATATCGTTATCGCAATACTCAACAACAATATCGCATATTTTCCCAGTATCGTGCAGCCCTA

>scaffold_30000015-2

ATATAGTACTATTCGCACAGAATGAGTATTATCTGAGGACCTTGTGTGATTTACAAATTACCACTCCACATCTGAATTTCGTGTGCCGCATTCGCACGGGATAAGCGAAGCCTGTGAATTTACTGACTTATCTCCCGGAAATGATGCAAAGCATTGGATAGGCTATTGCTTGGAAATATCTTGTGGTTATATCGGTATAGATGTATGATATATCATAAACAGAAGAATGTTCCTTACCGCATATACACTATACATGTAATTTATCTCCATCTAATCATTCTTTATGTTTTCTCTCTTCTGGTGGTGTTCAGTTTGATCTTTCTGAAAGTTGTATGGTTGTTTGGTGTATAGCGATTATGCAGCACCCAAAATACACGCAAAACACTCCAACGCAGCCTTATTGTCACACACAGGAAACAGAAACCTTAAAAAATATACACGACCCTGCAAATCCTGGCTAAATCGCAGAGATGTCGATTCGCACGGGACTAATATTATCACAGGACCTCAGTGTTCGGCAAAATATGGTAGGTCATTTATGGGGGAAATTTTACTTTACAAATTACAGACATGGACAATTCGCATGGGACTAAGATCACAGACAACCTCCGCAATTATTACAAATGACCAGAGGTCCCCAGATAATACTAATCCCGTGCGAATAGGACTTAT

>scaffold_30000015-11

TAGGGCTGTGTAAAAATATCGATACAGCTAACTATCGCGATATTTTTTTTCACACGTGTATCGATATTCTAACCTCTAGTATCAATACATATTTAAATTAGGTTTTAAACTGATTTACGTTAATATTTAATTAAAACCCTTAGTTTTTATTTGTCTGTTGAGTAATCAATATAATGTGATACACATACTAGAATTCTCCAGGTGACACACAGCGAGTTGTATTTTCAGCTATTTGTTTACAAATTTTTTTGTTGTTGAAGTATTGGAATGTATTGCAATATGTATTGTATTGTAATATATCGTGGTATATTGTATCGTGACCCATGTATCGTGATATGTATCGTATCTTGAGGCCCTTGCCAATACACAGCCCTA

>scaffold_30000015-13

GCATTTACACTGCAGGTCTTGATGCCCAATTTCTAACTACATCCAATTTTTTTGACAACCTGCTTACATCTTTTAAAACTGACCTGTATCCAATATCTGCATTTACACTATACACGGCAAAACAACCCAAGGCAGACGTACTGACTAGAAAAGGAAGTAAAAACGGTGCTATAATCAATGAACGCATTGGACGAAGACGAAATTATTATTCAGTATTTTGCTCTCCGCACTATATTTCTTGGAACCATGGGGTCAGGAACAGTGGGTCAGCAATTTTTGTATGAGATGAGAAACATTTATGTTGTCAAGAGGTCGCTGTTGGTGTCCACCTGACTTGACGTCATGTGCCACCGTCGCTTATTCAATGACGTACGACTCGCATTAACAGGTGAAAATCCGATCTGTCTGCTTACACTATGGCAGACGCTAATGCACGTTTCCGATTCATATCAGATTTATTTCCACACATGAATGAGGCCTGAAACCTATCTGAAAATATCGGAATCCATGTGCTTTTTTCCTGCTTACACGATCGTGGGTCATATCCCATCTGTTCCACATTGGAGGAAAAAATTGGAATTGGGTCACTTGATACATGCAGTTTAAATGC

>scaffold_30000016-2

TAAAGCCGGGTTTACACACTGAGATTTTGGCCTTGATTTGGTTGTCTAAGACAAATTTTGAAATCCTAAAAGATTCCTATAATCCTACACTAAAATCTGTTGTCTTTCAGTTTAACATGTTCACCGACACACGATTAATGACCGTTAGGATCATTTTTTTCCTCCGATGAAATTCTGTCCGTGTCAGAAGATTTTAGACATTTTCCTGCATTGTGACGTCTCCTACAATGATCGTCAAACCAAGAACCAATAGGAGCGCCGAACCTGATGACGCAATAAGCGCGACAACTTCAAACCACCTCGGGAAAATGTGGAAAGGAGTCCGGTGGACAGAACAGCAAGAAGAGAAACTTATTAAGTCATGGAGGGAAAAGGAGAGTTTGTATGACGTGTCGTCGCTGCTGTTTGTTTGCTGCTAGCTACCATTTAGCGAGAGAACGCGGGTTACTGAACGTGTCATGGATTGATGACGTAAAACTCCGGACGAGTCTTCTTGTGTGCGTCCGTTTTTGACGCGTCTCGACTGTCGTACAGTCTGACATAATGGTCAGTGAGATCCTACAGTGTGACACGGGGATCATGTTCGTACAGTCCGACAAACAACAATCGCAAAAGACTATTAATAATCGCTCAGTGTGTAAACCCGGCTTTA

>scaffold_30000016-4

AAGCCGGGTTTACACGGTGCGATTTTTAATAGTCTTTTGCGATTGTTGTTTGTCAGACTGTACGAACATGATCCCCATGTCACACTGTAGGATGTCAGTTGTCATAATGTCAGACTGTACGACAGTCGAGACGCGTCAAAAACGGACGCACACAAGAAGACTCGTCCGAAGTTTTACGTCATCAATGCGTGACATGTACAGTAACCCGCGTTCTCTCGCTAAATGGTAGCTAGCAGCAAACAAACAGCAGCGACGACACGTCATACAAACTCTTTTTTTCTCCATAACTCAATAAGTTTCTCTTCTTGCTGTTCTGTCCACCCGACTCGTTTCCACATTTTCCCAAAGGGGTTTAAAGTTGTTGTGCTAATTGCGTTGTTCCTATTGGTTCTTGGTTTGACGGTCGTCGTAGGAAAAGTCACACTGCAGGAAAGTGTCTGAAATCTTTTGACACTGCCAGGATTTCATCGGAGGAAAAAACTGATCGCAACGGTCAATAATCGTTTGTCGGTGAACACGTCAAACTAGCCATCAAACACAACAGGTTTTAGCGTAGGATTATAGGAATCTTTTAGGATTTCAAAATTTGTCTCAGACGAACAAATCGTGGCTAAAATCTCACAGTGTAAACCCGGCTT

>scaffold_30000016-8

TAGGGCTGCACAATACTGGGAACATATGCAATATTGTTGTTGAGTATTGCGATAACGATATTTCTTGCAATATAATATTTCCCTAGAGAAATGCTATTTTTATTAGCTATTTTAGCTGCAGGTGTTTTTCAGGCTGGTTCATCTTATCATGATTAACTCACTATGAAAGCTTTGCAGCTTGTTGATTGTCTTGCGTCAAAACACGCGCACGCAAATCCCAACTAACATCCGCGTGCCAGATATTTTACTATCCTAAAACTTTAACCACACATTAATATTTGAAGTATTAAATCGCTTATTTATCGCAAACCATTGTGATATGCACATTTGCGATATTTCGATATATTGTGCAGCCCTA

>scaffold_30000017-19

TTTAAAGGTACAATAGGTGATTGTCTTCAGAAACATTTTTTGTTATGGCCTGAAATTGAAATTTATGGTTGAAATTCTCTTCACATCCAGTTAGCAATCATTAAGTTAAGAGGTCTAAATGTATTTATATGTATTTATATATTCTGTGGAAGGCGTAAGAGCAAAAAATCTTCGTCCAATCAAAATGTTCGTTCCGAACATTATGATAGGCTTTCCTACCTGCCTGTCAACATATGTATTCGCATGCCTCTGCGCACCCTGTTCGTGCAGACAGGATACGTCATCAGCACGTTCACATACGCTGTGCAGACTGTGTGAGAATAGCAGGCAAACAGCAACAACCTGACCTTACTTCCCAACAACCGTCGCCAAGGGGCCTTTTAAAAGTGTCTACCAAAAGAAAGACAGTCTTTGAAAGGGCTACTGCGAAAAAAAGGCTGGATCAAAACAGAGCGAAAACAATTAATATCAGTTTTGTTTTTCCACGTTCAGGAGTTGTGGCTTTGGACAGCAATTTGCAGGGAGGGTGGGACGTATGCTTTCAGTGCTATCAGGCTACCGTTAGCATTTTCCAAGATGTCCTACTGCACCTTTAAA

>scaffold_30000018-12

TAGGGCTGCACAATATATATGAGACGTGCATGTCGCAATGGCTTGTGATAAATGAGCGATTTAATACTTCAAATATTAATGTGTGGTTAAAGTTTTAGAATAGTAAAATATCTGGCGCGCGGATGTTAGTTGGGATTTACGTGCGCGTGATTTGACGCAAGACAATCAACAAACTGCGAAGCTTTCATAGCGAGTTAATCATGATAAGATGAACCAGCCTGAAAAACACCCACAGCTAAAATAGCTAATAAAAATAGCATTTCTCTAGGGAAAAGTTATATCGCAAGAAATATCGTTATCGCAATACTCAACAATATTGCATATTTTCTCAGTATCGTGCAGCCCTA

>scaffold_30000018-13

TAGGGCTGTGTATTGGCAAGGGCCTCTCGATTCGATACATATCACGATACAATACATCACAACATATTGCAATACTTAAAAAAATAAATAAAAACATGTAAAACAAATAGCTAAACACACAACTTGCTGTGTACCACCAGGGGGATTCTAGTTTGTGCATCACATGTGTAATTAAATATTAACGTAAATGTAAATCAGTTTAAAACAATTCAAATACGTATCGATAATAGAGATTAGAATATCGATACACTATCGTGAGAAAAAAATATTGCGATAGTTAGCTGTATCGATGTTTTTACACAGCCCTA

>scaffold_30000021-5

ATATACAGAAAATCCTCGACTTCGACGGTGTTACGTTCTGGAAAACCCATCGGTAGTTGAAATATCGTAAGTCAATGTAAGTATACCCCCTATGATGCGTTCTTAAGTAGCCCACGGTAGCTTATAAGAGTAGTCACGAGGTTGCTGTAGATGCGTGTTTCAATAAAATCGCGCTGTTTTCTTTAGCCGCGCGAAATTGAAAAAAAATTTTTCAACATACTTTTTGGGTCACGCAGTCGTACCATAACTCCGAAAAATCACAAGTCGATATATCGTAAGTCGAGGGTTGTCTGTATAT

>scaffold_30000022-2

TATATTGCCAAAAGTTTGTGGACACCTCACCATCAGATCCATATGTGGTTCCTCTCCAAAATGTTGCTACAACTTTGGAAGCACACAACTGTCTAGAATGTCTTTGTAGGTTGTAGCATTACATTATTCCATTACTGGAACTAAGAGACCCAAACACTGTTCCAGCATGACAATGCCCCTGTGCACAAAACCCCTGAGCTCCATGAAGACATGCTGTGTTCTGGTTGGAGTGGAAAAACTTGAGTGTTCTTCCCAGAGCACCTTAACACCACTAAACACCTTTGGGATGAACTGGAACACAAACTGCACCACAGACCTCCTTACCCGACATTAGTGCCTGATCTCACTAATACTATTGTAGCTGAATGAACACATATCTCACATTCAAGCTCTAAATGGTAGCGGAAAGCCTTACTAGAAGAGTGAAGCTTAATCGAACAGCAAAATGGGGAGAAAATCTGGAATAAGATGTTTAAAATCACATATGTTTTTGATGGTCAGGGGTCCACAAACTTTTGCCGATATA

>scaffold_30000022-12

TTACAGGGGTCATGAACAACCTCAGTTTTTATTTTGTACTGTTCTCTGAGGTCCCCTTCTAATGTTATCAAGATTTTTACATCAAAAACATCATCATTTAGAAGAAATAGGCCATTTTCTGTCCTGTTTTTGACCCTCTTATCAGAACGCTCTGTTTGAATAGGCGTGGCGGATTATTGTCTATGTAGTAAACGCCCACTGCTATGATTGGCTAACAGTTTAATGCCGCTTGTCATTTGTCCATCACGTGCATGCGCGAATCGGTGGGCGGGGCTAAACAGACACTGATGCCGAAGCAGGTGTCAATCTTCTTCTGTGGAGGCGGAGCTTATCCACACTATTACGTCATCGAGTAGAACATTCCAAAACCTGTCGTTTTGGCCGACTGCCTTCAATATAAGCTGTTTTTAGGGAAACGACAAAGTTTTGAGTTCTGAAACTTACAGGATGTTTTTATAGCACAATGACCTCTTATATGTCAAAAGATCAAAGGAATTTGGATTTGTCAGTTCAAGACCCCTTTAA

>scaffold_30000024-2

TTTACACTGTGCGATTTTGGCCACGACTTGGTCATCTGTGGCAAATTTAGAAAGCCTATAAGATTCCTATAATCCTAGGCTAAAATCTGTTCTCTTTGATGGCTAGTTTGACATGTTCACCGACAGCCGATTAATGACATTGCTATCCGTTTTTCCACCGATGAAATTCTGGCACTTTCCTGCAGTGTGACTTCCCCTACGGCGACCATCAAACCTAGAACCAATAGGAGCGCCAAACCTGATGACACAACTTCAAACCACCTCGGGAAAATGTGGAAACGAGTCCGGTGAACAGAACAGCAAGAAAAGAAACTTATTGAGTTATGAAGGGAAAATAAGTGTTTGTATGACGTGTCGTCGCTGCTGTTTGTTTGCTGCTAGCTACCATTTAGCGAGAGGACGCGGGTTACTGAACGTGTCACGGATTGATGACGTAAAACTCCAGACGATTCTTCTTGTGTGCGTCCGTCTTTGAGGCGTCTTGACTGTTGTACAGTCTGACATTATGACAACTGACCTCCTACAGTGTGACATGGATATCATGTTCAGACAGTCTGACAAACAACAATCGCAAAAGACTAATAAGAATCGCACCGTGTAAA

>scaffold_30000026-2

CACTATATTGCCAAAAGTTTGTGGACACCTCACCATCAGATCCATATGTGGTTCCTCTCCAAAATGTTGCTACAAATTTGGCTGCACACAGTTGTCTAGAATGTCTATGTATGTTGTAACATTACATTATTTCTTCACTGGAACTAAGAGACCCAAACACTGTTCCAGCATGACAATGCCCCTGTACACAAAACCCCTGAGCTCCATGAAGACATGGTGTGTTATGATTGGAGTGGAAAAACTTGAGTGTTCTGCACAGAGCCCTGACCTTAACACCACTAAACACCTTTGGGATGAACTGGAAAGGCGGAACCACAGACCTCCTTACCCAACGTTAGTGCCTGATCTCATTAATACCATAGTAGCTGAATGAACACATATCTCACATTCAAAATTCAAATTGTAGTGGAAAGCCTTACTAGAAGAATGGAGCAAAATGGGGAGTAAATCTGGAATAAGATGTTTAAAATCACATAAGTTTGTGATGGCCAGGTGTCCACAAACTTTTGGCAAGATAGTG

>scaffold_30000026-3

TATACAGGGTGGGTGAAAAGTAACTAGGCATCAAAAATTGGTAATAAAGTCCATATTTCTAATACAAATTTATTGAAACAAATTTTACATGTACTTTATTACATAGGAAAATGAAAGCAAATCTTTAGATTTCAACATAGCATCAACCAGTTTCCTCAAACGGCCGGAGATGGAAAGTACAGTATTCTAAAGGAAGTTGTTTGGGATATTGCTGAGAACCTCCAGAATCCGTGCCTCCAAGTCCTCCAGTGTGCGAGGCTTGAAATCCGATTTACTATCGTCTATTTAGAGACTGTTTTCATCATGACTCCAGTGATACTACACGTTCTATAGTTACGAAATATTGCCTAGTTCATTTTCACCCACCCTTTATA

>scaffold_30000026-4

TTGGTAACACTTTAGTTTAGGGACCAATTCTCACTATTAACTAGTTTTTTTATTAGCATTTATATTGCTAGCATATTGGCTGTTTATTAGTACTTATAAAGCACATATTAATTTCTTTATACAATACTCCATGACCATATTGTGGATCCCTTAATCCTATCTCATACCTTAATTTAACAACCACAATGCTAACTATTAATAAACAGCAAATTAGGGGTTTATTAAGGCAGAAGTTGTAGTTACTAGTAAGTTCATAGTAAGAATCAGTCCATAAAGTAAAGTGTGACCAA

>scaffold_30000026-7

TTAAAGGGGTCATGAACTAACCCAGTTTTTATTTTTGTTCTCTGAGGTCCACTTATAATGTTATCAAGATTTTTACATCAAAAAACATAATTTAGAAGTAATATTCGATTTTCTGTCCGGTTTTTGACCCCCTCATCAGAACGCTCGGTTTGAATAGGCGTGGTGGATTGTTGTCTATGTAGTAAACGGCCACTGCTATGATTGGCTAACAGTTTTATGCCGCTCTTGTCATTTACCCATCATGTGCATGCGTGAATCGGTGGGCGGGGCTAAACAGACAGTGATGTCGAAGCAGATGTCGATCTTCTTCTGCAGAGGCAGCGCTTATACACACTATTACGTCATCGAGTAGAACATTCCAAAACCTGTCGTTTTGGCCGACTGCCTTCAATATAAGCTGTTTTTAGAGAAACGACAAAGTTTTGAGTTCTGAAACTTACAGGATGTTTTTATAGCACAACTGACCTCTTATATGTCAAAAGATCAAAGGAATTTGGATTTCTCAGTTCATGACCCCTTTAA

>scaffold_30000026-8

AAGCCGGGTTTACACTGTGCAAATCTGGTGACAATTTGCTCGTCTGAGACAAATTTAGAAATACTAACAAATTCCTATAATCCTACGCTAAAATCTGTTGTGTTTGATGGCTAGTTTGACGTGTTCACCGACAGACGATTAATAACGTTGCGAAAAGTTTTTTCTCCAATTAAATTCTGGCAGTTTCAGAAGATTTCAAACACTTTCCTGCAGTGTGACGTCTCCTACGGCGACCATCAAACCAAGAAGCAATCTGATCACGCAATTAACGTGACAACTTTAAGCTACCTCGAGAAAATGTGGAAACGAGTCCGGTGGACAGAACAGCAAGAAGAGAAACTTATTGAGTTATGGAGGGTAAAGGAGTGTTTGTATGATGTGTCGTCGCTGCTGTTTGTTTGCTGCTAGCTACCGTTTAGCGAGAGAACGCGGGTTACTGAACGTGTCACGGATTGATGACGTAAAACTCAAGACGAGTTTTCTTCTGTGCATCCGTTTTTGATGTGTTTTGACTGTCGTACAGTCTGACATAACAACTAAGATTCTACAGTGTGACATGAGGATCATGTTTGTACAGTCTGACAAGCAACAATCACAAAACACTCTTACAAATCACACAGTGTAAACCCGACTT

>scaffold_30000026-9

TAGCGATGCACCGATACCATTTTTTAAAGACCGAGTACGAGTACGGATATTTTTTTCCTGGTACTCGCCAATACTGATACCGATACCTGTACTTTTTGGGGATATGGGATTATTTGTATGGTTCTCGGGTTTTCTCTTGCCATTTTCTCTCGTCTGCTGCAGGGTCGGTTGCCGAGTGCTAACGTTACTGCTAGCGGCAAATTCTCCGTGCTCACTTTTGTGTTATAATTTCAGATTTTTTATCAGAGTACTCGTATTCTAAGTACTCATTATTGTACCTCTTGATATTTTTGCGGCACAAAGTTTACAGTCTGCCATGCGTGGGTTGTCCTCATTAATTTAGAAATATTTCCAAACCGCTGAGTCGGACATTCTGACGCTGCTGCCGGAGTCTTTGTGCACTGAAAGTTCTGTCAATGACGTCACAAGAGGTATCGGTCTATGGTATCGGAGTGTTAGTGCGAGTACATGAGCTCAGTATCGGGGCCCGATACCAGTATCGGTGCATCTCTA

>scaffold_30000026-17

CTAGAGATGAGCGAATCCGGATTCATCCGGGTTAAATAACCCGATCCTTTCTATGACTCGGGATTCACAAATCCTTCTCTTCATTTACTCGGATCAGTTGACTCCTGAGTTATCATAAATAAATACAAACTGAAAGTTACACCATTCAGTTGTTCTAAGTAGTTGTTAGCTAGGAACTGTAGGAACATGTAGCTGCCTGAGTTGTTTAGGAACAACAGGAACTTCCTGCATGGGCAGAAACATAAGAGACTCAAAATCGCAAGTACTGATTCATCTCATTCATCCGAATCGTGGGATTTAGAGGATCCATCGGACTTGTAGCACTGAGGGACTCAGTGTGTGTGTGAGAGATTCATGAGTCCAAGGATTCAGTGGATTCTAAGGATTCGTTGAGTGCCCTCTAGTGAAAGGATCATGGCAAAAGCAGTCAGAATCCCTACTGAGTCAAGTGACTCAAAGGACTTGTGAGCCTCGGATCCGTTCAATGAGCGACTCAGAAGGATTCGGATATCTCATCTCTAG

>scaffold_30000027-6

CTAAGGCTACATTCACAAGGCTGCGAGGCTTATTGCTCAATTCCGATTTTTGCTCAGATCAGATTATTTTTGTATCGCTGTTTACATTGTTTTAAATGTGGCCAATATCAGATTTGCAGTGTGAACTGATCGTGATCCTAAACTGACCTGCTTGCGCATAAAAGAACAAATTCTACGTCTCACGTAGCGTCCTGTCATACGGAAAGATAAACACGGAACACACTGAAGGCAGGATTTACGCGTTAGCTGCTTACATTTATAGGGTGATGTGCAGCGGACGACAGTAAATTAATGAGCAGTCGTTGAGGACGAAGAGAAAAAGGGCCACATTTTGAATAATAGCATGTTGTGGAGCAGTGGCTGCTACTTCAGTATGGAGGTGTGTGTGGATGCGGAGTTGGAGTCAGGAGTGGTGGAACCGTCACGTGATCAACGCCTTCAGCCAAATCGATTATACATCAACTGTTTGTGGTTACGCGCCAGTGCAGAATGATGACGCACGTCGATCGAAAATGACGTAAAAGTCACATGAATTCCGACATAACTGTTCACACTGCGGTGGCATTGAGAAGAAATCTGACCTGTGTCCGTTTTCATACCATATATGGAAGTGGCACAAATTGGAATGGAAAAGATCGGATTCCCTGCGGTTTGTGCTGTTTACACTGTCATGAGAAAAACAGATCCGAGTCACATGTGGGCAAAAAAATCGGATTTGGGTCACATTTTCCTGCAGTGTGAACGTAGCCTTAG

>scaffold_30000027-15

TATGGCTCTGTCCGAAATCGCATACTTCCATACTATATAGTATGCGAAACCGAGTATGCCCGAGTCGAATAGTATGTCCGAATCCTTAGTATGCAAAAAACAGTACACGAAAAGTTCCCGGATGATCTACTACTTCTGAAGTATGCATACGATGTACACTTTACTATCCCATGAGTCCACATCTTATGCGAAAGTGGTGGAAAGCAGAGACGCAACACTTAAAAAAAAATTAAGTGTTAAGTACTAAAAGAAACATTGTTGGTTTTGGGTAAATTACACTTGTTTAACATCATCCACGTAAACATTATCCGACTTTAAATTTGTGCTGTTGTGACGCTGTATGTCACGTGACAATATCAACATGGCGGATGTAGTACGTCGAAATTTCATTCATACTATATAACCATACTATATAGAACATACTTTTTTAACGGTTGGGAAGTATGTACTAACTCGAATGTAGTACATACTCAAATAGTCTGCGATTTTGGACGGAACACATA

>scaffold_30000028-1

AAGCCGGGTTTACACTGCAATTTTTGGCCAGGGTTTGGTCGTCTCAGACAAAATCAGAAATCCTTAAAGATTCCTATAATCCTACGCTAAAATCTGTTGTGTTTGATCTCTAGTTTAACATGTTCACCAACAGACGATTAATGACCATTTTTTCCTCCGATGGAAATTCTGGCAGTGTCAGCAGATTTCAGACACTTTCCTGCACTGTGCCTTCTCCTAGGACGACCGTCAAACCAAGAACCAATAGGAGCGCCGAACCTGATGATGCAATTAGCGCGACAAGTTCAAACCACCTTAGGAAAATAGAGCAAGAAGAGAAACTTATTGAGTTATGGAGGGAAAAGGAGTGTTTGTATGATGTGACGTTGCTTCGGTTCGTTTCCTGCTAGCTACCGTTTAGCGAGAGGACGCCGGTTCCTGAACGTGTCGCGGATTTACGATGTAAAACTCCGTGGACAAGTTTTCTTGTGTGCGTCCGTTGTTCATGCGTCTTGACTGTCGTACAGTCTGACATTATGACAACTGAGATCCTACAGTGTGACATGGGGATCATGTTCGTACAGTCTGACAAGCAACAATCGCAAAAGACTATTAAAAATCGCACAGTGTAAACCCGGCTT

>scaffold_30000028-16

ACTAGAGATGATCGAATCCGGATTCATCTGAGTTAAATAACCCGATCCTTTATAGATCTCGGGATTCACGAATCCTTCTCTTCATTTATTCGGATCAGTTGAGTCCTAAGTTATCTTGTTGCTAACAACAACATGGATAAATACAAACTGAAAGTTACACCATTCAGTTGTTCTAAGTAGTTGTTAGCTAGGAACTGTAGGAACATGTAGCTGCCTGAGTTGTTTAGGAAACTTCCTGCATGATCAGAAACATTATAGATTCAAAATCGCAGGTACTGATTCATCTGATTCATCCGAATCGTGGGATTCAGTGGATCGGCACGTAGTGAAACAGCCGGATCCAAGGAACTTGTAGTCCAAGGATTCGGAGGATTCCAAGGATTCAATGAGTACCCTCTAGTGGAATACCATAGCAAAAGCAGTCAGAATCCCTACTGACTCAAGTGCCTCAAAGGACTCGTGAGACTCGATGTCCGTTCAATGAGCGATTCAAAAGGATTTGAATCCGTCAATGGATTTGGATTTCTCATCTCTAGT

>scaffold_30000028-19

TACACTATATATATAGCCAAAGGTTTGTGGACACCTTACCAGCACAAATGTGTCTCCCGCAAACTGTTGCCACAAAGTCTGAAGAACACAGTTCTTTAAAATGTCTTTGTATGTTGAAGCATTACATTATCCCTTCACTGAAACTAAGAGACTCAAACCCTGTTCCAGCATGACAATGCCCCTGTGCACAAAACCCCTGAGCTCCATGATGTCATGGTGTGTGATGGTTGGAGTGGAAGAACCCGAGTGTCCTGCACAGAACCCTGACTCTGACTCAACCCCACTGAACACCTTTGGGATGAACTGGAACACAGACTGCACCCCAGACCTCCTCACCCAACATTAGTGTCTGATCTCACTAATACTATTGTGTCTGAATGATCAAATCCCCACAGTCACATGACACAACCTAGTGGAAAATCTGCACAGAAGTGTGGAGGTTTTTATAGGACGAAGAGTGAGACTAAATCTGGAAAAGGATGTTAAAAAAAAACACATAGATGTGATGATCACGTGTCCCAATACTTTTGTCTATATAGTGTA

>scaffold_30000029-4

TACCCAGCTAACAGGGAACGTTTCCACACCTTTCGCTAAACGTTCTTTAAAAGTTGTCTAAATGTTAAGACAAAACACTCTTTAAATAATGTTCACGGAACGTTCTCATAGCGTTATTAATAGTTAGTCTACGTTCTTGTAATGTTGAGAGAAAATGTTCTTAGAACAGTGGTCTTGGAACGTCCTTATGACGTTATTCGTTCTCGATGAATGTTCTCAAAATGTTTAGAGAGAAAGTTCTTAGAATACCACCATAAAATAACGTTCTACCAACGTTAAGCTCACTGGACATTTTCACGTTTTTGGAACATTTCAAATCCTGTTCCCAAAACCCCAAAAGAACATTCGAAAAACCTTCTCCGAATATACATTTGTTAGCTGGGTA

>scaffold_30000032-2

ACTATATTGCCAAAAGTTTGTGGACACCTGGCCATCACAAACATATGTGATTTTAAACATCTTATTCCAGATTTACTCCCTATTTTGCTGTTAGATTAAGCTTCACTTTTGTAGGCTTTCCACTACAATTTGGAGCTTGAATGTGAGATATGTGTTCATTTATCTACAATAGCATTAGTGAGATCAGACACTAACGTCAGGTAAGGAGGTCTGTGGTGTAACTGGCGTTCCAGTTCATCCCAAAGGTGTTCAGTGGTGTTAAAATCAGGGCTTTGTGTAGAACACTGAAGTTTTTCCACTCCAATCAGAACACAGCATGTCTTAATGGAACTCAGGGGCTTTGTCATGCTGGAACAGTGTTTGGGTCTCTTAGTTCCAGTGAAGAAATAATGTAATGCTACAAAGTACATTAGACATTCTAGACAACTGTGTGCAGACAAATTTGTAGCAACATTTTGGAGATGAACCACATATGGATCCGTTGGTGGTGTCCACAAAGTTTTTGCAATATAGT

>scaffold_30000033-1

TACACTATATTGCCAAAAGTTTGTGGACACCTGACCATCACAAACATATGTGATTTTAAACATCTTATTCCAGATTTACTTCCATTTTTCTGTTAGATTAAGCTCCATTCTTCTAGTAAGGCTTTCCTACAATAGCATTAGTGAGATCAGACACTAACATTAGGTAAGGAGGTCTGTGATGCAGTTGGCGTTCCAGTTCATCCCAAAGGTGTTCAGTGGTGTTAAAATCAGGGCTTTGTGTAGAGCACTCAGGTTTTTCTACTACAATCAGAACACACCATGTCTTAATGGAACTCAGGGGCTTTGTGCACAGAGGCATTGTCATGCTGGAACAGTGTTTGGGTCTTGTAGTTCCAGTGAAGAAATAATGTAATGCTACAACTTACAAAGACATTCTAGACAACGTAATGCAGCCAAATTTGTGGCAACCGTTTGGAGATGAACCACATATCGATATAATAGTGAGGTGTCCACAAACTTTTGGCAATATAGTGTA

>scaffold_30000034-1

ATACACTATATGGCCAAAAGTTTGCAGACACCTTACCAGGACACCCAAATAACCGTGTCTACCCCAAACTGTTGCCACAAAGGTGGAAGCACACAGTCATCTAGAATGTCTCTGTTCGTTCTAGCATTACATTATTCCTTCACTGGAACTAAGAGACTTAAACACTGTTCCAGCATGACAATGCCCCTGTGCACAAAGCCCCTGAGCTCCATGAACACATGCTGTGTTATGGTTGGTGTGGAAGAACTCGAGTGTCCTGCACAGAACCCTGACTCTGACTCAACCCCACTGAACACCTTTGGGATGAACTGGAACACCGACTGCACCCCAGACCTCCTCACCCAACATTAGTGTCTGATCTCATTAATACTATTGTGGCTGAATGAACAAATGCCCACAGTCACGCAACACAATCTAGTAAAAAGCATTTCCAGAAGTGTGGAGGTTCTTATATATGTGGAGTAAGAGCGAGACTACATCTGGAATAGGATGTTCAAAAAAACACAGATGGATTTGATAGTGAGCTGTCCTTTTGTCCATATAGTGTAT

>scaffold_30000035-1

TCTTAAAGGCAGGGTAGGTGATTTGGTTCAAAAACATTTTTTGTTATGCTAGTTGAGATTCTCTTCACATCTTGATAGCACTCATTAAGTTAAGTGGTCTGAATGTATTTATATGTATTTATATTGTCTGTGGAAACGTAGGAGCAAAAAAAACCTCGTCCAATCGAAATGTTCGATCCGAACATTACGATAGGCTATCCTACCTGCCTGTCAAATATGTATTTGCATACCTTGTTTGCGCAGACAGAATACGCCATTAGCGCGATCCCGCTATGCAGACTGAGCGAAAATTGCCTCTGAAAGTCATACAGGCAATCGCATGAGAAAAACAGTGTTAGAAAGAGTTATGTCTAAAAAATGTTCAGATCAGCAATGAGCTAAAAGTCGAGGTAATATAGGTGTATATCCGGCCTGCCTGTGCCCGTTCATGTGTTTTGGAGGAGGCGTGGCTTTGCAGAGTGATTATGCAGGAAGGGCAGGATCTTTGGATTTCAATGCTAGCTTGCTATTGCTAGTCTCTCTGAAATCGCCTACCCTACCTTTAAGA

>scaffold_30000035-6

TACACTGTATGGCCAAAAGTTTGTGGACACCTTACCAGCACACCAAACTGCCTTCCCTAAACTGTTTTCACAAAGTTGTCTATAATGTTTTTGTAAGTTGTAGAATTACATTATTTCTTCAGTGGAACTAAGAGACAAACACTGTTCCAGCATGACAATGCCCCTGTGCACAAAACCCCTGAGCTCCATGAACACATGGTGTGTGATGACTGGTGTAGACGAACTTGAGTGTCCTGCACAGAGCCCTGACTCTGACTCAACCCCACTAAACACATTTGGGATGAACTGGAACACTGATTGCACCCCAGACCTCCTCACCCAACATTAGTGTCTGATCTCATTAATACTATTGTGTCTGAATGATCAAATCCCCACAGTCACATGACACAACCTAGTGGAAGTCTTTCTAGAAGTTTGGAGGTTATTAGGAGGAAGAGTGAGACTAAATCTGGAACAGGATGTTTAATAAACACATATGGATGTGACGTCAGGTGTGCCATACTTTTGTCCATATGGTGTA

>scaffold_30000035-10

TAGGGCTGTGTAAAAATATATCGATACAGCTAACTATCGCAATATTTTTTTTTCTACCCTCTAGTATCGATACATATTTAAATTACGTTTTAAACTGATTTACGTTTACATTTAATTAAAACCTCAGTTTTTATTTGTCTGCTGAGTAATTAATATAATGGGATACATATACTAGAATCTCCCCAGGTGGCACACAGCAAGTTGTATTTTTAGCTATTTGTTTTACTTTTTTTTTGTTGTTGTTGAAGTATTGCAATATGTATTGTATTGCAATATATCATGACATATTGTATCGTAAACCAATGTATCATGATTTATCGTATCGTGAAGCTCTTGCCAATACACAGCCCTA

>scaffold_30000037-2

TCTACGGGGTGGGTGAAAAGTAACTAGGCATTAAAAATTGGTAATAAAGTCCATATTTCTAATACAAATTGTGCATGTATTTGATTACATAGGAAAATGAAAACAAATCTATAAATTTCAACACACCGGTGTTGAACCAGTGTTTTCTCAAACAGCCGGAGATGGAATGTCTTCTAAAGGAAGTCGTTTAGGATATTGCTGAGAATCTCCCGAATCCGTGCCTCCATGGCCTCCAGTGTGCGAGGCTTGAAATCCGATTTACTATCGTTTATTTAGAGACTGTTTTCATCATGACTGCAGTGATACTACAAGTTCTACAGCCATGAAATATTGCCTAGTTCATTTTCCCCAACCCTGTAGA

>scaffold_30000038-7

TACAGGGTGAGTGAAAAGTAACTAGGCATTAAAAATTGGTTTAAAAAAAGGGCCATATTTCTAATACAAATTTATTGAAACAAATTGTGCATGTACTTTACACACCCACAAAAAAGGAAATCTTTCAATTTCAACGTAGGCACCGGTGGTCATGGAATGCTAGAATGCTTCTAAAGGAAGTCGTTTAGGATATTGCTGAAAATCTCCCGAATCCGTGCCTCCATGGCCTCCAGTGTGCGAGGCTTGAAATCCGATTTACTATCGTTTATTTACAGCCTGTTTTCATCATGACTGCAGTGATACTACAAGTTCTACAGCCATGAAATATTGCCTAGTTCATTTTCCCCAACCCTGTA

>scaffold_30000038-10

TAGGGCTGTGTATTGGCAAGGGCCTCACGATACAATACATATCAGGATACATAGGTCACGATACATTGCAATACGTAAAACAAAATGTGAAACAAATAGCTAAAAAATACATCTTGCTGTGTACCACATGGGGAATTCTAGTATGTGTATCACATTATATTGATTACTCAATAGACAAATAAAAACGGAGGGTTTTAATTAAATATTAACGTAAACGTAAATCAGTTTATAACACAATTTAAATGTTTCGATACTAGAGGTTAAAATATTGATACATTATGGTGAAAAAAAATTGCAATAGTTAGCTGTATCGATATTTTTACACAGCCCTA

>scaffold_30000039-3

CTTATAAGTAGGGCTGCACGATACTGGAAAAATCTGCGATATTGTTGTTGAGTATTGCAATAACGATATTTCTTGTTCTATAACATTTCCCTAGAGAAATGATATTTTTATTAGCTATCTTAGCTAATGCGGGTGTTTTTCCGGCTGGTTCATCTCATCAAGATTAGCTCGCTATGAAAGCTTTGCGGCTCGTTGATTGCATCAAAGCACGCGCAGGCATATCCCAACTAACATCTGCGTGCCAGATATTTTACTATCCTAAAACTTTGATCACACATTACTATTTGAAGTATTAAATCGCTCATTCATCGCAAACCATTGCGATATGCATATCTCGATATTCCTATAATTTCGATATATTGTGCAGCTCTACTTATAAG

>scaffold_30000040-2

ATACACTATCTTGCCAAAAATTTGTGGACACCTGACCATCAAAAACACCTGATTTTAAACATCTTATTCCAGATTTACTCCCCATTTTGCTGGTAGATTAATCTCCACTCTTCTAGTAAGGATTTGCACTACAATTTGGAGCGTGAATGTGAGATATGTGTTCATTCAGCTACAATAGTATTAGTGAGATCAGACACTAACGTCAGGTAAGGAGTTCTGTGGTGCAGTCGGCGTTCCAGTTCATCCCAAAGGTGTTCAGTGGAGTTAATGTCAGGGCTTTATCTCCAATCATAACACAGCATGTGTTCATGGAGCTCAGGGGCTTTGTGCACAGGCGCATTGTCATGCTGGAACAGGTCGAAATAATGTAATGCTACAACATGCAAAGACGTTCTAGACACCTGTGTGCTTCCAAATTTGTAGCAACACTTTGATCGGATAGTGAGGTGCCCACAAACTTTTGTCAATATAGTGTAT

>scaffold_30000041-2

ATACACTATATTGCCTAAAGTTTGTGGATATATATATATATATGTGATTTTAAACATCTTATTCCAGATTTACTCCCTATTTTGCTGGTAGATTAAGCTCCACTCTTCTAATAAGGCTTTTTACTACAGTTTAGAGCGTAAATGTGAGATTTGTGTTCATTCAGCTACAGTAGTATTAATGAGATCAGACACTAATGTCGGGTGAGGAGGTCTGTGGTGCAGTCGGCATTCCGGTTCATCCCAAAGGTGTTCAGTGGTGTTAAGGTCAGGGCTTTGTCTCCAATCATAACACAGCATGTGTTCATGGAGCTCAGGGATTTTGTGCACAGGCGCATTGTCATGCTGGAACAGTGTTTGTGTCTCTTAGTTCCAGTGAAGGAATAATGTAATGCTACATTACATACAACAACATTCTAGGCAACTGTTTGCTTCCAAATGTGTGGCAACCGTTTGGAGATGAACCACATATGGTTATGATGGTGAGGTGTCCACAAACTTTTGGCAATATAGTGTAT

>scaffold_30000042-3

TATACAGGGTGGGTGAAAAGTAACTAGGCATTAAAAATTGGTAATAAAGTCCATATTTCTAATATTAATTTATAAAAACAAATTGTGCATGTATTTTATTACATAGGAAAATTAAAACAAATCTTTAAATTTCAACGTAGGCACCGGTGGTGAACCAGTTTCCTCAAACAGCCGGAGATGGAATGTCTTCTAAAGGAAGTCGTTTAGGATATTGCTGAGAATCTCCCGAATCCGTGCCTCCATGGCCTCCAGTGTGCAAGGCTTGAAATCCGATTTACTATCGTTTATTTAGAGACTGTTTTCATCATGACTGCAGTGATACTACAAGTTCTACAGCCATGAAATATTGCCTAGTTCATTTTCCCCAACCCTGTATA

>scaffold_30000043-1

TACACTATATTGCCAAAAGTTTGTGGACGCCTGACCATGACAAACACGTGATTTTTAACATCTTATTCCAGATTTACTTCCATTTTGCAGGTAGATTAAGCTCCACTCTTCTAGGCTTTTCACTACAATTTGGAGCGTGAATGTGTGAGATGTGATCATTCAGCTCCAATAGTATTAGTGAGATCAGACACTCACGTCAGGTAAGGAGGTCTGTGGTGCAGTCGGCGTTCCGGTTCATCCCAAAGGTGTTCGGTGGTGTTAAGGTCAGAACACTTAGGTTTTCCACTCCAATCAGAACACAGCATGTCTTGATGGAACTCAGGAGCTTTCTGCACAGGTGCATTGTCATGCTGGAACAGTGTTTGGGTCTCTTAGTTCCAGAGAAGGAATAATGTAATGCTACAACATACAACGACATTTTTTTCAAATTTGGGGCAACCGTTTGAAGATGAACCACATATGGATCTGATGGTGAGGTGTCCACATACTTTTGGAAATATAGTGTA

>scaffold_30000048-1

ACATACTCTATATTGCCAAAAGTGTGTGGACACCTGACCATCACAAACATATGTGATTTTAAACATCTTATTCCAGATTTACTCCACATTTTGCTGTTAGATTAAGCTCCACTCTTCTAGTAAGGCTTTCATTACAATTTGGAGCTTGAATGTGAGAGATGTGTTCATTCAGCTACAATAGTATTGGTGAGATCAGACACTAATGTTGGGTAATGAGGTCTGTGGTGCAGTCGGCGTGTCAGTTAATCCCAAAGGGGTTCAGTGGTGTTAAGGTCAAGGCTTTGTCTCCACCCATAACACACCATGTTTTAACGGAAATCAGGGGTTTTGTGCACAGGGGCATTGTTATGCTGGAACAGTGTTTGGGCCTTTTAATTCCAGTGAAGGAATAATGTAATGCTACAACATACAAAGACGTTCTAGACACCTGTGTGCTTCCAAATTTGTGGCAACCATTTGGAGATGAACCACATATGGATATGATGGTGAGGTGTCCACAAACTTTTGGCAATATAATGTATGT

>scaffold_30000049-5

TAGGGCTGTGTATTGACAAGGGCCTCACGATACAATACATATCACGATACATGGGTCACCATGCAATATATCACAATATATTGTAAAGCAATACATTGCAATACTTCAAAATAAAAAAGCTAAAAATACAACTTGCTGTGTAGCACCTGGGGGATTCTAGTATGTGTATCACATTCTATTGATTACTCAGCAGACAAATAAAAACTGAGGGTTTTAATTACATATTAATGTAAATAAGTTTAAAACGTAAATTAAATATGTATCGATACTAGAGGTTAGAATTACGATACACTATCGTGAAAAAAAATATTGCGATAGTTGGCTGTATCGATATTTTTACACAGCCCTA

>scaffold_30000049-7

TAGGGCTGCACAATATATCGAAATATCGCAGTGGTTTATTATAAATGATCGATTTAATACTTTAAATATTAATGTGTGGTCAAAGTTTTAGGATAGTAAAATATCTGGCACGCGGATGTTAGTTGGGATATACGTGCGCGTGCTTTGACGCAAGACAATCAACAAGCTGCGAAGCTTTCATAGCGAGCTAATCTTGATAATATGAACCAGCTCGAAAAACACCCGCAGCTAAAATAGCTAATAAAAATAGCATTTCTCTAGGGAAATGTGATATCGCAAGAAATGTCGTTATCGCAATATTCAACATTATCGCATATTTTCCCAGTATCGTGCAGCCCTA

>scaffold_30000050-1

ATATTACAGGGTGGGTGAAAATTAACTAGGCAATATTTCATGGCTATAGAACTTGTAGTATTGCTGGAGTCATGATGAAAACAGTCTGTAAATAGTCGATAGTAAATAGGATTTCAAGTCTCGCACACTGGACGACTTGGAGGCACGGATTCGGGAGGTTCTCAGCAATATCCCAAACGACTTCCTTCAGAACACTGTGCATTCCATCTCCGGCCGTTTGAGAAAACTGGTTGACGCCACCGGTGTCTTTGTTGAAATGTATAGATTTGCTTTTATTTTCCTATGTAAGTACATGTGCAATTTGGTTTTTATAAATTTGTATTAGAAATATGGACTTTATTACCACTTTTTAATGCCTAGTTACTTTTCACCCACTCTGTATAT

>scaffold_30000050-4

ATTAGGGCTGTGTAAAAATATCGATACAGCTACCTATCGTGATATTCTTTTCTCACGATAGTGTATCGATATTCTAACCTCTAGTATCGATACATATTTACATTATGTTTTAAACTAATTTACGTTTACTATTAATATTTCATTAAAACCCTCAGTTTTTATTTGTCTGTTGAGTAATCAATATAATGTAATGTACATGCTAGAATCCTCCAGGTGCTACACAGCAAATTGTATTTTAGCTATTTGTTTTATTTATTTATTTATTTATTTAAGTATTGCAATGTATCGTAATGCAATTATATCGTGATATATTGTATCGTGACCCATGTATCGTGATATGTATTGTATCGCGAGGCCCTTGCCAATACACAGCCCTAAT

>scaffold_30000051-1

ATATATATATCGAGGGGTGAGAACCAGAAGGGCGTAAGTGACATTTTACCAACTTTACAGGAGGGTCAAAAACAAAAAATTACCATAGTTTGATCTGACTTTGTGTACTGATTTCAATCTTTTATAATAAAAATGTGTACTCATATAGATGACAGCATACTTTAGCAGTAGAAAGAGCTCATCAAGAGTTTTCATTTAATATATATATCTCCATTTCACCCAAAATGTCACTTACGCCCTTCTGGTTCTCACCCCTCGATATATACTAT

>scaffold_30000051-7

TTACATTGTGCGATTTTGGCCACGATTTGTACGTCCGAGACAAATTTTGGAATCCTAAAAGCTTCCTATAATCCTATGCTAAAATCTGTTGTTTTTAATCGCTAGTTTAACATGTTTACCGACAATTGATTAATAGCCATCGTGATCCGTTTTTTCCTCCAATGAAATTCTGGCAGTGTCAGAAGATTCCAATCACTTTCCTGCAGTGTGACTTCTCCGACGACGACCGTCAAACCAATTGGAGCGCCGAACCCAATGACGCAATTTGCGCCACAACTTAAAACTATCTCGGAAAAATGTCGAAGCGAGTTCGGTGGACAGAACAGCAAGAAATGAAACTTGAGTTATGTCCTGAGCAAAATTACCGCTACAGATTGTTTTTGTTTGCTGCTAGCTACCATTTAACGAGAGAATGCGGGTTACTGAACGTGTCACGGATTGATGACGTAAAACTCCGGACTAGTCTTCTTGTGTGTGTCCGATTTCGATGCGTTTTAACTGTCGTACATTATGACAACTGAGATCCTACAGTGTGACATGGGGATCATGTTTGTGCAGTCTGACAAGCAACAATCGCAAAGGATTATTACAAATCGCACAGTGTAA

>scaffold_30000051-10

TAGGGCTGCACAATATATTGAAATTTTCGAAATATCAACAAATGTACATATTGAGATATGCATATCGCAATGGTTTAATACTTTAAATATTAATGTGTGGTCAAATATCTGGCACGCGGATGTTAGTTGGGATATACGTGCGCGTGCTTTGACGCAAGACAATCAACAAGCTGCAAAGCTTTCATAGCGAGCTAATCTTGATAATACGAACCAGCCCGAAAAACACCCGCAGCTAAAATAGCTAATAAAAATAGCATTTCTCTAGGGAAATGTGATATCGCAAGAAATGTCGTTATCGCAATATTCAACATTATCGCATATTTTCCCAGTATCGTGCAGCCCTA

>scaffold_30000054-4

ATACACTATATTGCCAAACATTTGTGGACACCTGACCATCACAATCCTATGTGCTTTTAAACATCTTATTCCAGATTTACTGACAGATTACCCATTTTGCTGTTAGATTAAGCACCACTCTTCTAGTAAGGCTTTCCAACTTTTACAATTTTGAGCTTGAATGTGAGATATATGTTCATTCAGATGCAATAGCATGAGTGAGATCGGACACTAATGTCAGGTAAGGAGGTCTGTGGCGCAGTCGGCGTTCCAGTTCATCCCAAAGGTGTTCAGTAGTGTAAAGGTCAGGGCTTTGGGCAGAACACTCAGGTTCTTCCACCCAACCATACCACACCACGTCTTAATGGAACTTTGTGCACAGGGGCATTGTCATACTGGAACATTGTTTGGGCCTTTTGGTTCCAGTGAAGGAATAATGTAATGCTACTACATACAAAGACATTTTAGACAACTGTGTGCTTCCAAATTTGTAGCAACATTTTGGAGAGGAACCACATATGGATCTGATGGTGTCCACAAACTTTTGGCAATATAGTGTAT

>scaffold_30000054-20

ATGCTGTGTTCACTGCAAACGCGAATTGAATTATTTGCGTGAGTAGATTACATACAAAGTCAATGCAAAGACGCCAATAGAGGCAAATTCGCGGAAGACCCGATTAAGGAGAATATAGCGTGTTTCTCGGCAGACGCGCTATATTCTCCTCAGTTTCAACTCGAGTGGAAAATTCGCATGACGCGTTGTTTCACAAGCCAGTCAGCGAAGAGTGTTTTATCAGACAATGGAGAAGAGCATTATTGTAGCCGTCTGCGGGCACCCCTAAAATGTATGTTTTATGAATAAAAGCATGGAGCGAGTGACGCGAAAAACATTCTGCGAGTAATCTAGAGCGAGTGACACGATGCACATATTCGCTTTGTGTTTGGTGTAAACACAGCAT

>scaffold_30000056-4

AGTAGGGCTGTGTATTGGCAAGGGCCTCACGATATGATATATATCACGATACATGAGCCACGATACAATATATCATGATATATTGCAATACATTTTTTTATGTAACTTGCAAACATGCTGTGTACCACCTGGGGGATTCTAGTATGTGGAGCACATTATATTGATTACTCAGCAGACTAAAGCTTTTAATGAAATATTAACGTAAATGTAAATCAGTTTAAAACGTAATTTAAATATGTTTCGATACTAGAAGTTAGAATATTGATACACAATCGTGAAAAAAAATATTGTGATAGTTAACTGTATCAATATTTTTACACAGCCCTACT

>scaffold_30000056-8

CTATACAGCGAAAAGTTTGTGGACACCTCACCAACACATCAACATGAGTGTGTCTACCCTAAAATGTTGCCACAAAGTTGGAAGCACACAGTTGTCTAGAATGTCTTTGTATTTTGTAGCATTACATTATTCCTTCGCTGAAACTAAGAAGCTCAAACACTGTTCCAGCATGACAATGTCTCTGTGCACAAAGCCCCTAACCTCTTTAAAGACATGGTGTGTTATGATTGGAGTGGAAGTACTTGAGTGTCCTGCACTGAGCTCTGACCTCAACCTCACTGATCACCGTTGGGATGGACTGGAACACAAACTGCACCCCAAACTTTCTCACCCAATCTTAGTGCTAATCTCACTAATACTATTGTGGCTGAAAGATAAAATCCCCACAGCCACACTCCAAAATCTAGTGAAAAACCTTTCCAAAAGGTTGTAGGTTATTAAAGTATTATAGTAAGAGTGAGACTAAATTGAAAGTAGGATGTTCAAAAAAACATATGAAAGTGATGGTCTGGTGTCCCAAAACTTTTGACTATATAG

>scaffold_30000058-1

ATACACTATATGGACAGAATTATTAGGACACCTGGCCAACCCATTCATGTGTTTTTGAACAACCTATTCCAGATGTATTCTCACTCTTACTCCTATAATAACCTTTATACTTCTGAAAAGGCTTTTGCTAGACTTTGAAGCTTGTCTGTGGGGATTTGTTCATTAAGCCATAATAGTATTATTGAGTTTAGGCACTAATGTTGGGTGAGGAGGTCTGGGGTGCAGTCGGTGTTCCAGATCATTATAAAGGTGTTCAGTGGGGTTGAGTCAGAGTCAGGACTGTGCAGAACACTCAATGTCTTTTACTCCAACCAGAACATACGATGTCTTGATGCAGCTCAGGAGCTTTGTGCACAGAGACATTGTCATGCTGGAACAGTGTCTGGGTCTCTTAGTTCAAGTGAAGAAATAATATAATGCTACAACACACAAAGACATCCTAGACAACTGTGTGCTTCCAACTTGGTAGCAACAGTTTGGGGAAAACACTTATGTGGGTGTGCTGGTAAGGTGTCTACAAACTTTTGGCCATATAGTGTAT

>scaffold_30000059-3

TACACTATATGACTAAAAGTTTGTGGACACCTTGCCAGCACACCTGTAAATGTGCCTTTCCCAAACTGTCACAAAGTTGGAAGCGCACAGTAGTCTAGGATATCTTTAGATGCTGTTGCATTATATTATTACTTCACCAGAACTAAGAGCCCGAATGCAGTTCCAGCATGACAATGCCCCTGTGCACAAAGCCCCTGAGCTCTATGAAGACATGGTGTGTAGAACTCAAGTGTTCTGCACAAAGCCCTGACCTTAACCCCACTGAATACCTTTGGGATGAACTGGAACACTGACTGCACCCCAGACCTCCTCACCCAACATCAGTGTCTGATCTCACTAATGCTATTGTGGCTAAATGAGCAAATCTTCATCGCCAAGCTCCAAAACTTTAGCTAAAAGCATTTTTAGAAGTGTGAAGGTTATTATAGGAGTAATAGTGAGACTATATCTGGAGTAGGATGTTTAAAAAACATATGTGGATGTGAGGGTCAGGTGTCCCAATACTTTTGTCCATATAGTGTA

>scaffold_30000060-14

AGTATGGCTGCACAATATATCGAAATTATCGAAATATCGCAAATGTGCATATCGAGATATGCATATTGCAATGGTTTGCGATAAATGAGCGATTTAATACTTCAAATAATAATGTGTGCTCAGAGTTTTAGGATAGTAAAATATCTGGCACGCGGATGTTAGTTGGGATTTGCGTGTGCATGCTTTGGCGCAAGACAATCAACAAGCTGCGAAGCTTTCATCGCGAGCTAATCTTGATAAGATGAACCAGCCCGAAAAATACCCGCATTTAGGGAAATGTTATATCGCAAGAAATATCGTTATCGCAATAAACAACAACAATATCGCATATTTTCCCAGTATCGTGCAGCCCTACT

>scaffold_30000060-15

TTAACCCTCATGGGGTGTTTATGTTTTTGTTTCCTAGCCAATGTTCACGGGTCTGGTGGACCCGCCGCATTATTGGGGTTTTTTAATTAACACAGTCATAACAATTTATAGAAAAACACTTAAAAGCTGTTTACTTTAGCTCACTTACAAGCAATATAGACATCATTTATGGTTAGTATTTGCCATTTACTCCGTTAGATCACATTGGAAAATAGAAGTGTTATGGAAAATATGTTCTATTTTATGTTCTATATGAAAAATGAGCCACGGCCAATGAGTCTGGGGTAAAAAAAATTATTAATAGCATAATTTCTGTTTAAACAAGCGTTGAAATCCCACAGACCCAAACACCACACAAGGGTTAA

>scaffold_30000061-2

ATATACACTATATGGCTAAAAGTATATAGACACCTTACCAGTCCACCCAAGTGAGTCAACCCCAAACTGTTGCTACAAAGTTGGAAGCACAGTTTCCCAGAAGATCTTTGCATGTTGTCGCATTACATTATTCCTTTACTGGAGCTAAAAAGCCCAAACCTGTTTCATCAGGGCAATGCCCCTGTGCACAAAGCCCCCGAGTTCCATGAAGACATGGTGTGTTCTGATTAGTATCGGAGAACTCGCGTGTCCTAAACAGAGCTCTGACCTCAACCCGACTGTACAGCTTTGGGATGAACTGGAACACCAACTGTACCTCAGACCTCCTCCCCCAATATTAGTGCCTGAATCCACTAATACTTTTGTGATGGATGCCCCCAAAATCTAGTGGAAAGCCATTCCAGAAGCGTGGAGGTTATAATAAGAGTAAAAGTGAGACAAAATCTGGAATAGGATGTTAAAAAAAAACACAGATGGATGTGATGGTCAGGTATCCCCATACTTTTCACCATATAGTGTATAT

>scaffold_30000062-1

TACGGTGGCCAAGAGAGCTCAATGCACTGCAAATTAAGAAATTACATGATAATAGAAAAAACACCAACAAATTAAGAAAACATCTCCACAAATCCTCACAATGCAAGCAAATGCAAAAAGGCGCTGCAAATTACAGAAACACGCTGCAAATCCTCACAACACAACCAAATACAGGAACGCTCTACAAATGGCACAGACGGAAATGTTTCAACGGACCCCAAAAGGTGACGAACCCGGCTGGCACCTGCTTATTGTTCAGTGGTGTTATCGGTGAACTGAAAGGTACATTTTTTGTTTATTTCATCATCCCGATCGTATGATTCCTTGGTCTTTTGGATATTATTAGCCTAAATAATCTGATGAATTACACATTTAACTGTGAAACGATAAACAGGTCCCAGACGAGTTCGTCGCTTTTTTGGGATCCCCTTGAAACATTTCCGTTGTGGTCTGTGCTAGTTGCAGCGCGTTTCTGTATTTGTGGTTGCATTGGGAGGATTTGCAGCACGTCTATTGTCAAACTGATGAAGATGTTTTCTTAATTTGTTGGCATTTTTTCTATTTGAACGTGTTTTCTTAAATTGTAGTGCGTTGAGCTCTCTCGGCCACCGTA

>scaffold_30000062-2

TATCCCACATGAAAAGATACCATAGTATACTTTAGGATTTAGTATACAGTAAGATGTAGTATAGTGTAGTATATTACAGTGTTTACTACATCTTACTGCAGCATACTATAGTATTAACTCTAGTGAATTGATAAACGGTAGTAAATACTGTAGTATACTTTACACGTTACTAGAGTAAACTACAAGACTGTAATAAACTGTATCATATTATAGTATTTACTCAAGTATTTAGTTTATTATAGTATTTACACTTGGTTAATGTAGATGCCATACTATAGAAAGAACTGTAGTGAACTGATCAACAATATTTGCTATATGGGAAACAGTTGCTAATGGGGAGCAATGTGTTCCAGTGTGAAAGGCCAGATTCCCATTTCTAATGGGCAGCAATGTCAGATCATTGATTCAGCAAAAGTTAAACAATGCAGCAAAGATTTTCAACAAAAATGAGCAATAAAAAAATGAATGGTTTTATTCATTTTAGACAATTTGTGTGCATTGCATTGATACACTTTTTGCTCTGATCTGTGCTGTGAAGGTTCTCACACATACAGTATAATATAAAAAAATTACTATAGTAATTAATGTAGTCATGTGTTTATTTTACTATAGTAGAGTTACTAAAACAAGTGTAGTAGAATTACCACAACAAATGACCACATCATTGCAGTTTACTATGGAATATTTACTATAGTAACGTTCAAAAACACTACAGTATTTACCACAGGGAATTACTCCATGTTCTATAGTAAAGTTACTCTACAATACTATAGTCTTTTTCATATGGGATA

>scaffold_30000062-21

TTAACCCTTGTGTGGTGTTTGGGTCTGTGGGATTTCAACGCTTGTTTAAACAGAAATTATGCTATTAATAATTTTTCTTACCCCAGACTCATTGGCCGTGGCTCATTTTCCATATAGAACATAATATAGAACATATTTTCCATAACACTTCTATTTTCCAATGTGATCTAATGGGGTAAATGGCAAATATTAACCATAAATGATGTCTATATTGCTTGTAAGTGAGCTAAAGTAAACAGCTTTTAAGTGTTTTTCCATAAATTGTTATGACTGTTTTAATTAAAAAACCCCAATAATGCGGCGGGTCCACCAGACTCGTGAACATGGGCTAGGTAACAAAAACATAAACACCCCATGAGGGTTAA

>scaffold_30000064-2

ACTATATAGCCAAAAGTTTGCAGACACCTTACCAAGCACACTTACATAAGTGTATCTCCCCGAAATTGTTGTTGTCACAACGTTGGAAGCACACAGTTGTCTAAGATGTCTCTATATGCTGTAGCATTATATTTCTTTTAACTGGAACTAAGAGGCCCAAAGTCTGTTCTAGCATGACAATATCCCTGTGCACAAAGCCCCTGAGCTCCATGAAGACATGGTGTGTTGTGATTGAAGAACTTTAGTGTTCCACACAGCCTCACTCAACATTTTTAGGATGAACTGGAACACCGACTGAACCCCAGACCTTTTCACCCAACATTAGTGTCGGATATCACTAATACTTTTCTGGCTGAATGAACACAAATCCCCACAGTCACACTCCAAAATCTAGTGGAAATAAGTTCCAGAAGTGTGGAGATCATTACAGGAGTAAGAGTGAGATTAAATCTGGAATAGGATGTTCAAAAAACACACATGGACGTAATAGTCAGGTGTCCCAATACTTTTGTCAATATAGT

>scaffold_30000066-11

TTAAAGGGGTTATGAGCTGAGAAATTTAAATTTCCTTGATCTTTTGACATATAAGAGGCCATTGTGCTATAAAAACATCCTGTAAGTTTCAGAACTCAAAACTTTGTCGTTACTCTAAAAACAGCTTATATTTAAGGCAGTCGGCCAAAACAACAGGTTTTGGAATGTTCTACTCTATGACGTAATAATGTGGATAAGCGCCACCTCCGCAGAAGAAGATCAACACCTGCTTCGACGTCACGGTCTGTTTAGGCCCGCCCACTGATTCGCGCATGCATGTGATTGGTAAATCACAAGAGCGGCATAAAACTGTTAGCCAATCATAGCAGTGAGCGGTTACTCCCTAGACAACAATCCGCCACGCCTATTTAAACAGACGAGGGGGTCAAAAACAGGACAGAAAATAATTTAGTACTTCTAAATTATGATGTTTTTTGAGGTAAAAATCTTATTAACATTATAAGTGGGCCTCAGAGAACAGTACAAAATAAACTGAGGTAGTTCATGACCCCTTTAA

>scaffold_30000068-1

TACATTATATTGGCAAAAGTTTGTGGACACCTCACCATCAGATCCATATGTGGTTCCTCTCCAAAATGTTGCTACAAATTTGGAAGCACACAGTTGTCTAGAATGTCTTTGTATGTTGTAGCATTACATTTCTTCACTGTAACTAAGAGACCCAAACACTGTTCCAGCATGACAGTGCCCCTGTGCACAAAGCTCTTGAGACATGGTGTGTTATGATTGGATTGGAAAAACTTAAGCTCTGACCTTAACACCACTGAACTTCCAAATGCCAAATGCACCACAGACCTCGTTACCTGACGTTAATGTTTGATCTCACTAATACTATTGTAGCTGAATGAACACATATCACACATTTAAGCTCCAAATTGTAGTGGAAATCCTTACTAGAAGAGTGGAGCTTAATCTAACAGCATAATGGGGAGTAAATCTGGAATAAGATCACATATGTTTGTTTTGGTCAGGTGTCCACAAACCTTTGGCAATATAGTGTA

>scaffold_30000068-7

TTACACTATATAGACAAATGTTTGTGGTCACTTGACAAGCACAACCACATAAATGTGTCTTCAACAAGCCGTTGCCACAAAGTTGGAAGCAAACAGTTGTCTAGAATGTCTTTGTATTTTGTAGCATTACATTATTCCTTCAATGGATAATAAGAAGCCCAAACCTGTTTCAGCATGACAATGCCCCATGCACAAATTAAGCTCCATGAAGACATGGTGTGTTATGGTTGGTGTGGAATAACTCGAGTAACCTGCACAGAGCCCTGACCTCAACCCTTCTGAAAATCCTTGGGTATGAACTGGAACACTAACTGAACCCAGAACTCCTCACCAGATATAAGTGCCTGAACTCACTAATACTATTGTTGCTGAATTAGAAAATTCCCACAGCTACACTCGAAAAGTTAGCGTAAAGTATTTCCAGAAGTGTATAGGTTATGATAGAAGTTAGAATTAGCCTAAATTTGGAATAGGATGTTCAAAAAACACATATGGATATGTCCCAATGGATATGGTGTCCCAATACTTATTGCCCATATAGTGTAA

>scaffold_30000069-9

TACACTATATGGCCAAAAGTATTAGAACATCATATTTATATGTTTTTTTGAGCATTCTATTCCAGATTTAGTTTCACTCTTACTCCTATAAAAATTTTACATCAAAATTTTTTTTAACTAGATTGTAAAGTGTGGCTGATGGGACTTGTAATCATTTAGCCTGAAAATGATTAGTGAGATCAGGCATTAATGTTGGGTGAAGATGTCTGGAGCGTAATCGATGTTCTAGTTCATTCCAAAGGTGTTCAGTGTGGTTGAGGTCAAAGCTCTGGGTAGAAAACTCGAGTTCTTCCACTCCATCCACCACACACCATGTCTTCATGGAGCTCAGGGCCTTTGTGCACAGGGGCATTGTCATGCTGGAACAGTGTTTGTTTTCAAGTTTTAGTTCAAGTGAAGGAAAATCTTAATGCTACAGCATGTACAGATATCCTAGACAAAAGTTGTGTTTTCAATGTTGTGACAAGAGTTTGGGGAAGACTCATTTATGGGTGTGTTGATAAGATGTCCACAAACCTTTGACTATATAGTGTA

>scaffold_30000070-2

CAGGGCTGGCTAACCCATGTTCCTTAAGTTCCGCTTGTTTTCCAGCTATTCCTGCCCTACCCACTGCTGATTACTTGGTTCAGATGTGTTCAGTCAATCAGAAGCTGGAAGATACCATTTTAGATAAGGGTGGAATGGGGGAAGACCTGGTTATTTGGTATCTCCGACCCTCTGACTGACTGAACCACCTGAACCAGGTAAAATAGGGCAGGGGTAGCTGGAAAACAAGTGGAACAAAGTAACTTAAGGAACATGGGTTACCCAGCCCTG

>scaffold_30000070-3

TTAACCCTTGTGTGGGGTTTGGGTCTATGGGACCCGATTTTGTTTAAAGAGAAATTATGCTACTAATGAATGTTTTTAGCCCCAGACTCATTGGCCTTGGCTCATTTTCCATGGAGAACATAAAATAGAACATATTTTCCATAACACTTACAGTACATTTTCAAATGTAATCTAACAGAGTAAATGACAAATATTAACCATAAATGATGGTTTTATTGCTTGTAAGTGAGCTAAAGTAAACATCTTTTATGTGTTTTTCCATGAATTGTTATGGCTGTGTTGATTAAAAACCCAATAATGCGGTGGGTCCACCAAACCCGTGAACACTGGCTAGGTAACAAAAACATGAACACCACACAAGGGTTAA

>scaffold_30000074-1

TATATATACACTACATTGCCAAAAGTTTGTGGACACCTTACCATCACAAACACATGTGGTTTTAAACATCTCTTTACTCTTCATTTTGCTGTTCGATTAAGCTTTAGTCTTCCAGTAAGGCTTTCCACTACAATTGAATGTGAGAGATGTGTTCATTCAGCTACAATAGTATTAGTGAGATAAGGCACTAACGTCAGGTAAGGAGGTCTGTGGTGCAGTCGGCGTTCCAGTTCATCCCAAAGGTGTTCAGTGGTGTTAAGGTCAGGACACTCAAGTTTTTCCACTCCAATCCTAACACACCATGTCTTAAAGGAACTCAGGGCTTTGTGCACAGGGGCATTGTCATGCTGGAACAGTGTTTGGGTCTCTTAGTTCCAGTGAAGGAATAATGTAATGTTACAAACTACAAAGACATTCTAGACAACTGTGTGCTTCCAAATTTGTAGCAACCTTTTGAAGAGGAACCACATATGGATCTGATGTGCAGGTGTCCACAAACTTTTGGCAGTATAGGGTATATATA

>scaffold_30000076-3

AAACTACAGTGGAATCTCGGCATACAGATTTAATTAGTTCTGGAGTCGTGTTCTTAAGGCAAAAATTTGTATTGCGAAACGTAAAAATATTGTATTGCAGATAATCCGATAAAGCCACCCAAAACTATTACCAATATGACCAATTTCCAACACTATAATCACATTTTTGCATATAGAAACAATCAAAACATTTAGAAAAGACATGTAAATGAAGTAAAACATGAAATAAATAGACCCACGCCACCAGTCTGTCAACAAAAAAACGTGCAAAACATCATGTAGCTTTTGAATGCAGGCCGAAGGCAACATTGGCATCATGGATTTCAAAACAGCTTTCGCTGACAAAAAAAATAAATATTTCATTCATAATCTCACTTCGATTGGCTTCGCTTGGCTTTCCACTGACGCAAACAAGTTTTTAACGGCTCCCGGACATACGCGCAACTCAGCGTTCGGTGCGGTTCGTGCCGATGCAAATTTCTTGCAAAATTTAAATTTTTAAGGCGAAAATTCGTAAGGGAGGGCATGGGTATGCCGAGGTTCCACTATACTTT

>scaffold_30000077-2

ATACTGCCAAAAGTTTGTGGACACCTGCACATCAGATCCATATGTGGTTCCTCTTCAAAAGGTTGCTACAAATTTGGAAGCACACAGTTGTCTAGAATGTCTTTGTAGTTTGTAACATTACATTATTCCTTCACTGGAACTAAGAGACCCAAACACTGTTCCAGCATGACAATGCCCCTGTGCACAAAGCCCTGAGTTCCTTTAAGACATGGTGTGTTAGGATTGGAGTGGAAAAACTTGAGTGTCCTGACCTTAACACCACTGAACACCTTTGGGATGAACTGGAACGCCGACTGCACCACAGACCTCCTTACCTGACGTTAGTGCCTTATCTCACTAATACTATTGTAGCTGAATGAACACATCTCTCACATTCAATTGTAGTGGAAAGCCTTACTGGAAGACTGGAGCTTAATCAAACAGCAAAATGAAGAGTAAATCTGGAATAAGATGTTTAAAATCACATGTGTTTGTGATGGTCAGGTGTCCACAAACTTTTGGCAATAT

>scaffold_30000079-4

CACTATATAGACAAAAGTATTGGGACACCTGACTATTGCATCCATGTGGGTTTTTTGAACATTCTATTCCAGATTTAACCTGTAATAATCTCCACACTTCTGAAAATTATTTCCACTAGATTTTGGAGTGTGGCTATGGTGGGATTTATGTTCATTCAGCCAGAATAGTATTAGTGAGATCAGACACTAATGTTGGGTGAGGAAGTCTGGAAGTCTGAAGGTGTTGAGTGAGGTTGAGGTCAGGACTGTGTAGAACACTAAAGTTCTTTAACCAGAACACACCATATCTTTGTGGAGCTCAGAGCCTTTGTGCACAGGGGCATTTTCATTCTGGAACAGTCTTTGGGCAACTTAGTTCCAGTAAAAATAAATAGAATGGTACAGTATGATGAGACATCTTAGACAACTGTGTACTTTCTGACAACAGTTTGGGTAAGACACTTATGTAAGTGGGCTTGGTAAGGTGTCCGCACACATTTGGCTATATAGTG

>scaffold_30000079-12

TACCCAGCTAACAGGCAAAAATTGTTCCCACAATATTCGCTAAGGTTCTTTAAAAGTTGTCTGAATATTTTAACAAAACGTTCTTAAAATATTGTCCAAAGAACGTTCTCATAACGTTTGTAATAGTTAATATACATTCTTGTGATGTTAAGAGAGAACGTTCTTAGAACAATGTCATTGGAACGTCCTTATGATGTTATTCGCACTTGATGAACGTTCTCAAAATGTTTCGAGAAAACGTTCTTAGAACAACACCATAAAATAACCATAAACAGAAATAATGTTCTACCAATGTTAAGCAAACTGGACATTTTCACATTTTTGTAACATTTAAAATCACGTTCCCAAAACCAAAACAGAATGTTTTAAAAACGTTCTTAGAACATAAATGTGTTAGCTGGGTA

>scaffold_30000083-1

TGGCTACGTTCACACTGCAGGCAAATGTGGCCCAAATCCGATTTTTTTGCCCGCATGTGACTCAGATCTGGTTTTCTCATGACAGTGTGAACAGCATAAACCGCATGGAATCTGAGCTTTTCAATTCCAATTTGTGCCACTTCCATATGCAGTATTAAATCCGACACAGGGCAGATGTTTTGCAATGCGATCGCAGTGTGAACAGTTATGTCGGAATTCATGCGACTTTTACGTCATTTTCGATCGACATGCGTCATCATGCTGCACTGGCGTGTACTACAAACAGTTGATATATAATCGATTTTGTTGAAGGCGTTCACGTCACAGTCCCACCACTCCTGGCTCCGGCTCCGCATCCACACACACCTCCATACTGAAGTAGCAGTCAGTGCTCCACAACATGCTTTTAATCAAAATAAAATGGATCTTTTTCTCCTCATCCTCTTCCTCCTCAACGGCTGCTCATTAATTTGCTGTCGTCCGCTGCACATCACCTTATAAATGTAAGCACGTAACGCGTAAATGCTGCCTTCAGTGTCTTCCGTGTTTATTTTTCCGCATGACAGGACGCTGTGTGAGACGTAGAATTTGTTCATGCGGGTCAGTTTAGGACTATGATAAGTTCACACTGGAAATCTGATTTTGGCCACGTTTAAAACAACAATGTAAACAGCTATACAAAAAAACCTGATCTGAGCAAAAATTGAGAATTGAGCACTAAGCCTTGCAGTGTGAGCGTAGCCA

>scaffold_30000083-2

CTAAAGCCGGGTTTACACTGTGCGGTTTTTAATGGTCTTTTGTGATTGTTGCTTGTCAGACTGTATGAACATGATCTCTATATCACACTGTAGGATCTCAGTTGTCATAATGTCAGATCGTATGACAGTCAAAACGCATCAAAAACGGACGCACACAAGACGACTTGTCAAGAGTTTTACATCATCAATCCATGACACGTTCAGTAACCCGCGTTCTTTCGCTAAATGGCAGCTAGCAGAAAACAAACACCAGCGACGACGCGTCATACAAACACTCCTTTTCCCTCCATAATTCAATAAGTTTCTCGTCTTGCTGTACTGTCCACCTGACTCGTTTCGACATTTTCCCCAAAATAGTTTGTAGTTGACATGCTAATTGCATCATCAGGTTCGGCGCTCCTATTGGTTTTTGGTATGACGGTCGTCGGAGAAGTCACACTGCAGGTAAGTGTCTGAAACCTTCTGACACTGCCAGCATTTCATCAGAGGAAAAAACGATCGCAATGGTCATTAATCGGCTGTCGGTGAACATGCAAACTAGCAATTAAAGACAACAGATTTTAGTGTAGGATTATAGGAATCTTTTAGGGTTTATTTCAGACAACCAATTCATGGCCAAAATCCCACAGTGGAAGGCCGGCTTTAG

>scaffold_30000083-3

ATACACTATACTGCCAAACGTTTGCGGACACCTTCCCAGCACACCCACATTAGTGTGTCTTAGTGTTTGTTACTACAAACTCTGAAGCACACAGTTGTCTAGGATGTCTCTAAATGTTGTAGCATTACATTTTTCCTTCACTGGAACTAAGAGGCCCAAACACTGTTCCAGCATGACGATGCCCCTGTGCACAAAGCCCTTGAGCTATATGAAGACATGGTGTGTTCTGGTTGGAGTGCTAGAGTGTCCTGGACAGAGCCCTGACTCTGACACAACCCCACTGAACACCTTTGGGATGAACTGGAACACAGACTGCACCCAAGACCTCCTCACCCAACATTAGTGTCTGAATACTATTGTAGCTGAATAAACACAAATCCCCACAGTCACACTCCACAATCTAGAGGAAAGCTTTTCCAGATATCTGGAGGTTATTATAGGAGTAACTGCGAGACTATATCTGGAATAGGACGTTCAAAATACACATATACATGGTGATGGTCAGGTGTCCCAATAATTTTGTCTATACAGTATAGTGTAT

>scaffold_30000084-1

GGCCACATTTACACTGCAGGTGTTGATGCCCAGATCCGATTTTGTGACTGTCAGATTTTTTTGATGTCCTGCTTGCATCTGCTTTTAAAACTGACCCGTATCCGATATCTGCATTTACACTATACACGGCAAAACAACCCAAGGTAGGTTCAACGTACTGACTGGAAAAGGAAGTAAAAACGACGCAATGGACAAAGACCAAATGATTATTCAGTATTTTGCTTTCCGCACCATATTTAAAGGTCGGCAAAACAATGCTCTCTTTAGGGGGTGAGAAAAGAGGAGAGCCCTTGTTGCAGCCTGGGAATATGTAGCATTGCTCGCTACATATAGGTCGCCGTTGGTGTTCACCTGAGTTGACGTCGTGTGCCTCCGTCACTTATTCAATGACGTACGACTTGCATTAACCGGTGAAAAACCAAAACTGTCTGCTTACATGGCATACGCTAATGCAAGGATCCGATTTATTTCCACATATGAATGAGGCTTGAATCCATGTGCTTTTTTCCTGCTTACACGATTGTGGATCATATCCGATCTGTGCCACATTGGAGGAAAAATTGGGTCACTCAATACATGCAGTGTAAATGTGGCC

>scaffold_30000084-12

TTAACCCTTTTGTGGTGTTTGGATATGTGTAAACCGATTTCAAAGTTTGTTTGTTATGTTTTTTAGCCCCAGACTCATTGGCCTTGGCTCATTTCCCATGTAGAGTATAAAATAGAACATATGTTCCATAACACTTATATTTTCAAATGTGATCTAACGGGGCAAATATTAACCATAAATGATGTCTATATTGCTTGTAGGTGAGCTAAAGTAGCCATCTTTTAGGTGTTTTTCCATAAATTGTTATGGCTGGGTTGATTTAAAACCCAATAATGCAATGGGTCCACCAGACCCGTGAACATTGACTAGGTAACAAAAACATGAACACCACATGAGGGTTAA

>scaffold_30000086-1

GCAGGGCTGGGTAACCCATGTTCCTTAAGTTACTTTCGCCTGTTTTCCAGCTACCCCTGCCCTACCGACTGAACTGAACACACCTGAACCAGGTAATCAGCAGTGGGTAGGGCAGGGGTAGCTGGAAAACAAGCAGAACAAAGTAACTTAAGGAACATGGGTTACCCAGCCCTGC

>scaffold_30000086-4

CAGTGTTGCCAACTTAGCGACTTTGTTGCTAGATTTAGTAACTTTTCAGACCCCTTTAGCAATTATTTTTGAAAAATCTAGCGACTTTTGGACAAACCTTAGCAACATTCCAAATGTTGCCAGTACTGTGCTGGGAGCGCGAGGTGTTGCTTTCTCGGTACGGTTACACCTCTCCCTGTGTCTGCTCTGATCAATGAGCGCTAGCTCCGTCTGAAAGGAACAGCACGTTCCAGTGACCGCACGAATGCAAATACGATGTAATTACGGCATTAATATGCAAATTTGCGCATGACGTCATTTAGCGACATTTGGCGACTTTTTGAGCAGACTTTAGCCCCTTTCCATTGAAATTAGTTGGCAACACTG

>scaffold_30000087-5

CGGAGTGGAGCGGCTGCGATTTTGCTCAGAGTTTGGAGCAAGTTTTTTAAAAGTTGTAGCGTTGTGGTTTTCTGTCGCTCTGAGCATGCTCCATCACGAGCTCAACTCTTGTTAACGGCCCATAACCCAACTGCATAGAGCATGAATGAATTCACCACTGACAGACTCAATAAAATATGGATTTCCTTAAATACTATCTATCTATCTATCTATCTATCTATCTATCTATCTATCTATCTATAATAAAATGTTTACAGTGGAGCGTTAATAAGGCACAATTTTTTCCATGGAGAAACATAGAACATAGAGCAAGGAGTAAGTGAGGAGGGAGCATGGAGTGAGCGAAAAGCGGGGAAATGAAAATCCGGAGCGGAGCTGTAGTGGAGCGATTATAAAATGCTCTCAGCGCGGAGCGGAAATTTTTGCCGCTCCACTCCG

>scaffold_30000088-7

TAGGGCTGCACGATACTGGGAAAATATGCTATATGCGATATTGTTGCTGAGTATTGCAATAAGGATATTTCTTGCAATATAACGTTTCCCTAGAGAAATGCTATTTTCATTAGCTATTTTACCTACAGGTGTTTTTCGGGCTGGTTCATCTTATCAAGATTAGCTCGCTATGAAAGCTTCGCAGCTTGTTGATTGTCTTGCATCAAAGCACACGCACGCATATCCCAACTAACATCCGCGTGCCAGATATTTTACTGTTTAAATATTAATGTGTGGTCAAAGATTTAGAAGTATTAAATAGCTAATTTATCACAAACCATTGCAATAGGCATATCTCATTTGCGATATTTCGATATATTGTGCAGCCCTA

>scaffold_30000089-2

CACTATATTGCCAAAGGTTTGTGGACACCTTACCAGCACACCCACATTAGTAGGTCTTGATGTTTGTTACCACAAACTCTGGAGCACACAGTTGTCTGGGATGTCTCTATGTCTTGTAGCATTACATTATTCCTTCACTGGTACTAAGAGACTCAAACACTGTTCCAGCATGATGATGCCCCTGTGCACAAAGCCCCTGAGCTCTATGAAGACATGGTGTGTTCTGGTTTGAGTGAAAAGACTCGAGTGTCCTAAACAGAGCCCTGACTCTGACTCAACCCCACTGAACACCTTTGGGATGAACTGGAACACAAGACCTCCTCACCAAACATCAGTGTCTGAATACTATTGTAGATGAATAAACACAAATCCCCACAGTCACACTCCACAATCTAGTGGAAATCCTTTCTAGAAGTGTGGAGGTTATTATAGGAGTAACTGTGAGACTAAATCTGGAATAGGACGTTCAAAAACACATATAGATGTGATGGTCAGGTGTCCCAATACTTTTGTCTTTATAGTG

>scaffold_30000091-2

TACACTATATAGCCAAAAGTTTCAGGACACCTCACCATCAGATCCATATGTGGTTCCTCTCCAAAATTTTGCTACAAATTTGGAAGCACACTGTTGTCTAGAATGTCTTTGTATGTTGTTGCATTACATTATTCTTTCACTGAAACTAAGAGACCCAAACACTGTTCCAGCATAACAATGCCCCTGTGCACAAAACCCCTGAGTTCCATTAAGACATGGTGTGTTATGATTGGAGTGGAAAAACTATTCTGTACAAAGCCCTGATTTTAACACCACTGAACACCTTTGGAATGAACTGGAACGCCAACTGCAACACAGACCTCCTTACCTGATGTCTGATCTTACTAATACTATTGTAGCTGAATGAACACATATCTCACATTCAAACTCCAAATTGTAGTGGAAAGCCTTACTAGAAGAGTGGAGCTTAATCTAACAGCAAAATTAAATTTAAACATCTGGAATTAGATGTTTAAAAATCACATACTGTATGTTTGTGATGGTCAGGTGTCCACAAACATTTGGCAATATAGTGTA

>scaffold_30000091-3

ATAAATACGCTACACTGCCAAAAGTTTGTGGACACCTCACCATCAGATCCATACGTGGTTCCGCTCCAAAATGTTGCTACAAATTTGGAAGCACACAGTTGTCTAGAATGTCTTTGTATGTTCTTGCATTACATTATTCTTTCACTGGAACTAAGAGACCCAAACACTGTTCCAGCATAACAATGCCCCTGTGCACAAAACCCCTGAGCTCCATTAAGACATGGTGTGTTATGATTGGAGTGGAAAAACTTGAGTATTCTGCACAAAGTCCTGATCTTAACACCACTGAACACCTTTGGAATGAACTGGAACACAGACTGCACCACACGCCTCTTTACCTGAGGTTAGTGTCTGATCTTACTAATACTATTGTAGCTGAACGAACACATATCTCACATTCAAGCTCCTAATTGTAGTGGAAAGCCTTACTAGAAGAGTGGAGCTTAATCCAACAGCAAAATGGGGAGTAAATCTAGAATAAGATGTTTAAAATAACATGTTTGTGATGGTCAGGTGTCTACAAACCTTTGGCAATATAGTGTATATAT

>scaffold_30000091-4

TAGGGATGCACCGATACTGATACTGGTAAAAACATTCTGATACTTGTAAAAACATTCTGATACCAAAGGCCGATACCTCTTGTGACGTCATTGACAGAACTTTCAGTGCACAAAGACTCCGGCAGCAGCGACAGATTGTCAGGCTCAGCAGTGTGGAAATTAATGATGACAACCCACGCATGGCAGACTGCAAACTTTGTTCCGCAAAAATATCAAGAGGTACAAAAATGAGTACTTATAATACAAGTAATCTGATTAAAAATCTGAAATTAAAACACAAAAGTGAGCACAGAGAATTTTCCGCTAGCAGTAACGTTAGAACTCGGCAACCAACCCTGCAGCAAACCCTTGCAAGACGTGAGAAAATGGCAAGAGAAAACCCGAGAGCCATATAAATAATCCCACATCCCAAAAAAGTACAAGTATCAGTATCGGCGAGTACAAGGAAAAAAATATCGATACTCATACTCTGTCTTTAAAAATAGTATCGGTGCATCCCTA

>scaffold_30000092-2

ATATCCTCCTGAGACCCAAGGACAAAGTGTTTTTGTTTTGTTTTGTGTGATTTCCTACTCCTTTCGGGTTAAAAAACATTTTATGATTTAGAGTTTTTAAACATTATTGTTATTTAAAATTTTACAGCATGTCCACTGTAGTAGACCACAAGACCATTTTAGTTTAGAAAGACTTTCCAGACCTTACGTCTTTCTATAAAATGTAATTGTTCTTATGGCGGCCATGTTGGATACAGTGTAAGAACTAGTTCCTAGCATCCTAGCCAATCTAATGACATATCACTAGAAAGTTTACAATACAGCTGAAATTAATAGAGTAGCTCAAAACATTTATAAAAAACGAAATGTCCACTACAGAGGACACAAGTCAATGGGCCAGGTCTCAGGAGGATAT

>scaffold_30000093-2

ATACACTTTATGGCCAAATGTTTATGGACACCTCACCATTGACTTAGGATTCTTCTCCAATCTGTTGTTATAAATTTAGGAAGCAATTGTATAGAATGTATTTGCATACTGTAGCTATACAATTTCCCTTTACTGGAACTAACAGACCCAAACACTGTTCCAGCATGACAATACACAAAGCGCCTGAGCTCCATAAAGACATGGTTTGCCAATGTTGGAGTGGAAGAACTCAAGTCTTCTGCAAAGAGCCCTGACTGCAACCCCACTAAACACTGTTGGGATGAAATTAAACAATGACCCCTGCACCCCAGTCTTCCTTACCCAACATTAGTACCTAATCTTGCTAATGCTGAATGGACACAATGTCTGACAGCCACGATTTAAATTGTGGTGGGAAGCCTTCCCAAATGATTGAAGCTTATTATAACAGCAAAAGAGGGAAGAAATCTAGAATAGGATGTTTAAAATCACATATGGTTGTAATGGCCCACAATGGTGTCCAGAAACTTTTGGCTATATAGAGTAT

>scaffold_30000095-1

CAGGGCTGGGTAACCCATGTTCCTTAAGTTCCTTTGTTCCGCTTGTTTTCCAGTTACCCCTGCCCTACCCACTGCTGATTACCTGCTTCAGGTGTGTTCAGTCAATCAAAGGGTAGGAGATACCAAATCACCAGGTCTTCCCCCACTCCTCCATCATCTGAAATGGTATCTTCCGGAAGCAGGTAATCAGCAGTGGGTAGGGCAAGGGTAGCTGGAAAACAAGCGGAATTAAGTAACTTAAGAAACATGGGTTACCCAGCCCTG

>scaffold_30000095-5

ACTAAAGTGGGGCTTACACTGTGCGATTTTGGCCACGATTTGTTCGTCTGAGACAAATTTTGAAATCCTAAAAGATTCTTATAATCCTACACTAAAATCTGTTGTCTTTGATCGCTAGTTTGGCATAGACATTAGTTCACCAACAGCCTATAAATGGCCGCTGTGATCATTTTTTTCCTCCAATGAAATTCTGGCAGTGTCAGAAGATTTCAGACACTTACCTGAAGTGTGACTTCTCCTACGATGACCGTTAAACCAAGAACCAATAGGATCGCCGAACCTGATGACGCATTTAGCGCTGCAACTTCACCACCACCTCGGGAAACTGTCAAAATGAGTTTGGTGGACAGTACAGCAAGAAGAGAAACATTGAATCATGAAGGGAGAAAGAGTGTTTTTATGACGTGTATGACTAGCTACCATTTAGCGAGAGAAAGCGGGTTACTAAACGTGTCACGGATTGATAACGTAAAACTCCGGACGAGTCTTCTTGTGTGCGTCCGTTTTTGACGTGTTTTGACTCTCGTACAGTCCGACATTATGGCAACTGAGATCCTACAGTGTAACATGGGGATCAGGTTCGTACAGTCTGACAAGCAACAATCGCAAAGGACTATTAAAAAATTCGCACAGTGTAAATCCGGCTTTAGT

>scaffold_30000096-1

AGCTTTAGTGCCACGTTGAAAAAAATGTATAAATAAGATTACGAGAATAAAGTCAAAATACTTCGAGAATAAAGTCGAAATACTTCGAGAATAAAGTCGAAGCAATACGAGATTAATAATAGTAATATTAATAGTAATATTTTCAGAAAAAAATCAAAGAATTGTGAGAAACGTTTCTCTGTTTTGTATAAAATGGATGTGGAGGATTTTGTTAAATGCTACTTTGGGTAAGGATTTATAGCAATAAGGAAATTATTTGTCTTTTGGCGCATCAGCACGGAGTTATAATTAGTAAACGGACACTTTGGCGGTTATGTAGAAAACTTAATCCTAACTTAAAGTAAGACAAACAATTCCTCCTGTTAAAAAGGTCATTAATCTGTTAGTGTGTTAATGAGTGATGACCACGTCTCTCTCCTCTGCTGAGAAAAATAGATGTACAACAACCCCGCACGGCAGCCAATCAGCATTCCCGATTTACATACAGCTACAACTTTCTCATAATTTTGACTTTATTCTCAAAATATTATGATTTTAATGTTGTATTGCTTCGACTTTATTCTCAAAGTATTTCACCTTTAATCTTTGTAATCTTATTTATTTATTTTTTCAACGTGGCACTAAAGCT

>scaffold_30000096-2

ATACACTATATTGCCAAAAGTTTGTGGACACCTGACCATCACAAACATGTGATTTTAAACATCTTATTCCAGATTTACTCCCCATTTTGCTGTTAGATTAAGCTCCACTCTTCTAGTAAGTCTTTCCACTACAATTAGGAGCTTGAATGTGAGATATGTGTTCGTTCAGCTACAATAGTATTAGTAAGATCAGACACTAACCTCAGGTAAAGAGGCGTGTGGTGCAGTCAGTGTTCCAGTTCATCCCAAAGGTGTTCAGTGGTGTTAAGATCAGGACTTTGTGCAGAATACTCAAGTTTTTCCACACCAATCATAACACACCATGTCTTAATGGAGCTCAGGGGTTTTGTGCACAGGGGCATTGTTATGCTGGAACAGTGTTTGGGTCTCTTAGTTCCAGTAAAGGTATAATATAAAGCTACAACATACAAAGACATTCTAGACAACTGTGTGCTTCCAAATTTGTAGCAACATTTTGGAGAGGAACCACATATGCATCTGATGGTCAGGTGTCCACAAACTTGGCAATATAGTGTAT

>scaffold_30000096-3

ATACACTATATTGCCAAAGGTTTGTGGACATCAGATGCATATGTGGTTCCTCTCCAAAATGTTGCTACAAATTTGGAAGCACACAGTTGTCTAGAATGTCTTTGTATGTTGTAGCTTTATATTATACCTTTACTGGAACTAAGAGACCCAAACACTGTTCCAGCATAACAATGCCCCTGTGCACAAAACCCCTGAGCTCCATTAAGACATGGTGTGTTATGATTGGTGTGGAAAAACTTGAGTATTCTGCACAAAGTCCTGATCTTAACACCACTGAACACCTTTGGAATGAACTGGAACACTGACTGCACCACACGCCTCTTTACCTGAGGTTAGTGTCTGATCTTACTAATACTATTGTAGCTGAACGAACACATATCTCACATTCAAGCTCCTAATTGTAGTGGAAAGCCTTACTAGAAGAGTGGAGCTTAATCCAACAGCAAAATGGGGAGTAAATCTGGAATAAGATGTTTAAAATCACATGTTTGTGATGGTCAGGTGTCCACAAACTTTTGGCAATATAGTGTAT

>scaffold_30000098-8

TAGGGCTGTGTAAAAATATCGATACAGCTAACTATCGTGATATTTAGTGTATCGATATTCTAACCTCTAGTATCGATACATATTTAAATTACGTTTTAAACTGATTTAGGTTTACATTAAAACCCTCAGTGTTTATTTGTCTGCTGAGTAATCATGTGATACACATACTAGAATGTTTTTTTTAAGTATTGCAATGTATTGCAATATGTATTGTATTGTGTTATGTTGTTATATATATTGTGATATATTGTATGGTGATATGTATCGTATCGTGAGGCTCTTGCCAATACACAGCCCTA

>scaffold_300000102-2

TTAGGGCTGCACAATATATAGGAATTATCGAAATATCGCAAATGTGCATATCGAGATATGCATATCGCAATGGTTTGCGATAAATGAAAGATTTACAGTAATACTTTGAATAATAATGTGTGGTCAAAGTTTTAGGAAAGTAAAATATCTGGCATGCGCATGTCAGTTGGAATATGCGTGCGCGTGCTTTGACGCAAGACAATCAACAAGCTGCGAAGCTTTCATAGCGAGCTAATCTTGATAAGATGAACCAGCCCAAAAAACACCCGCAGCTAAAATATCTAATAAAAAATAGCATCTATGGAAATGTTATCGTAATAAATGTTATCCCAATACTCAACAACAATATTGCATATTTTCCCAGTATTGTGCAGCCCTAA

>scaffold_300000106-3

AGCTCCGTCCAAAATTGCATACTTTCATACTAGATAGTATGCGAAACTAAGTATGTGAGGCGAGTAGTATGTCCAAATCCTTTGTATGCAAAATACAGTATGTGAAAAGTTCCCGGATGATTTACTACTTCTGCCCGGATTTTGAAGTATGCATATGATGAACATTTTACTATCTCATGAGCCCACGGGAGAGACGACTCGTCATATGCGGAAATAGCAGAAAGCAACGATGCGGCACTTTTCAAGTGATTTAAGTACTTCAGGAAAAAAAACTGTTTCTTGTGGTTAAATTGCACTTGTTTATCATTATTCATATAATTATCCAACTTTAAATTCACGCTGCTGTGATGACGTATGTCATGTGACGAAATCAACATGGCGGATGTAGTACGGCCGAATTTCATTCATACTACCCGTAACCATACTATATAGAATGTACTTTTTTAATGCTCAGGAAGTACGTACTTACTTAAATGTAGTATGTACTTAAGTTGTATGCGATTTTGGACAGAGCT

>scaffold_300000106-4

GTACACTATATGGCCTAAAGTATTGGGACGCCTGACCATTACATCCATATTTGCTTTTTGAGCATTCTATTCCAGATTTAGTCTTACTCTTCCTCCTATAATAACCTCCACACTTCTAAAAAGGTTTTTCACTAGATTGTGAGGATTTTCTCATTCAGCCACAAAATGATGAGTGAGATCAGAAACTGATGAGAGGTGAGGAGGTCTGGAGTGCTGTCTGCGTTCCATTTCATCCCAAAGGTGTTCAGTGGGGTTGAGGTCAGGGCTGTGTGCAGAACTCTTGAGTTTTTCCAAACCAACCAAAACGCACCATGTCTTCATGGAGTTCAGGGGCTTTGTGCACAGGGGCATTGTCATGCTAAAACAGTGTTTGAGACTCTTAGTTCCAGTGAAAAATGTTTAATGTAACAGCATAAAGACACATCCTAGATAGGTGTGTGTGCTTCCAAATTTGTGGCAACAGTTTGGAGAAGATGCACTTTTGGACATACTGGTAAACTGTCCACCAACTTTTGGTCATATAGTGTAC

>scaffold_300000109-4

TATCTACAGGGTGGGTGAAAATGGCAATATTTAATGGCTATAGAACTTGTAGCATCACTGGAGTCATGATAAAAACAGTCTGTAAATAGGATTTCAAGCCTCGCACACTGGAGGACTTGCAGGCGTGGATTCGGGAGGTTCTCAGCAATATCCCAAATGACTTCCTTCAGAAGACCGTGCATTCCACCTCTGGCCGTTTGAGGAAACTGGTGGACGTTGAAATTTAAAGATTTGCTTTCATTTTCCTATGTATTAAAGTACATGTACAATTTGTTTCAATAAGTTTGTATTCGAAATATGGACTTTATCAATTATTAATGCCTAATTACTTTTCCCCCACCCTGTATATA

>scaffold_300000111-2

TACACAATATGGCCAAAAGTTTGTGGACACCTTACCAGCACACCCATAACAGTGTTTCCGAAACTGTTGCCAAAAGTTGAAGCTAAGTTGCCTAGGGTGTCTCTGTATGCTGTAGCTTTAAGATTTCCCTTTATAGGAACTAAGAAACCCAAACACTGTTCCAGCATGACAATGCTCCTGTGCACAAAACCCCTGAACTCAATGAAGACATGGTGTGTTATGGTTAGTTTGGAAGAACTCAAGTGCTCTGCAACCCTGACCTCAACCCCACTGAACACCTTTGGGATGAACTGGAACCTCCTCACCAAAAATTTGTGTCTAAACTTACCAATCATTTTGTGGCTAAATGAGCAAATCCTCCAAACCATGCCCCAAAATCTATTGGAAAGCTTTTCAGTAAGTGTGGAGATTATTACAGAAGTAAAAGTGAGACTAAATCAGGAGTAGAATGTTGAAAAAACACAAATGAATGTGATGGTCAGGTCTCCCAATACTTTTTGGCCATATGGTGTA

>scaffold_300000112-11

TATCCTCCTGAGACCAAGAAAAAAAAAGGTGTTATTTTGGGTTTTGTTTTGTGTGATTTTTTACTCCTGTCAGGTTAAAAACATTCTACAATTTAGAGATTTTACTGTTATTTTTTATTTTATAGCTCAACTATAGTAGACCACAGGACCATTTTAGTTAGAAAAAACAGTCATTTCCATAAAACAAGCTTGTTCCTATATGGTGGCCATATTGGATGCAGTGTAAGAACTAATGTAGTTCCTAGCATCCCAGCCAGTCAAATGGCATATCACTAGAAAGCCCAGAATGCACAGGTCCTTTGTACAGTAGAGCAGAAATTGTTAGAGTAGCTAAAAAAAAATCAAAAAACATTTATGAAAACAAAATGTCCACTACAGGGGACATAAGTCAATGAGCTGGGTATCAGGAGGATA

>scaffold_300000115-2

ATATTGCCAAAAGTTGACCTGACCATCAGATCCATATGTGGCTCCTCTCTAAAAATGTTGCTACAAATTTGGAAGCACGCAGTTGTCTAGAATGTCTTTGTATGTTGTAGCATTACATTATTCCTTTACTGGAATACCCCATGACAATACCCCAGTGCAAAAGCCCCTGAGTTCCATTAGGACATGGTGTGTTATGGTTGAAGAGGAAAAACTTGAATGTTCTGCACAGAGCTTACAGATCTTACACCATTGAACACCTTTGGGATAAACTGGAATGCCGACTGCACCACAGACTTCTTTAACTGACGTTAGTGCCTGATCTCTCTAATACTTTTATTAGCTGAATGAACACATATCTCACATTCAAGCTCCAAATTGTAGTGGAAAGTCTTACTAGAAGAGTGGAGCTTAATCAAACACCAAAATGGGGAGTAAATCTGGATAAGATGTTTAAAATCACATATGTTTGTGATGGTCAGGTGTCCACAAACTTTTGGCAATAT

>scaffold_300000116-19

TAGGGATGCACCGATACCATTTGTAAAAGGCCGAGTACGACTACCGATTTTCTTTCTTGGTACTCATCGATACCGATACTTGTACTTTTTGGGGATGTGGGATTATTTGTATGGCTCTCTATGGGTTATCTCTTGCCATTTCCTCTCGTCTTGCACAGGTTTGCTGCAGGGTTGGTTGCCGAGTGCTAACGTTACTGCTAGCGGCAAATTCTCAGTGCTCACTTTTGTGATTTAATTTCAGATGTTTCATCAGATTACTCGTATTATAAGTAATCGTTTTGTACCTCTTGATATTTTTGCAGAACAAAGTTTGCAGTCTGCTATGAGTGGGTTGTCATCATAAATTTTAAAATATTTTTACACTGCTGAGCCTGACATTCTGTCGCTGCTTCTGTCTTTGAGCACTGAAAGTTCTGTCTGTGACATCACAAGAGGTCTTTAGTATCGGAGTGTTTTTAGGAGTACGAGTACATGAGCTCAGTATCGGGCCCGTGCATCCCTA

>scaffold_300000119-23

ACTATATGGCAAAAAGATTTTGGACACCTAACTAGTGCACCCATAAGTATGTCTTCTACAAACTGTTGATACAAAGTTAAATTGAGTTTTCTAGAATTTCTTTGTATGTTGTGGCATTACATTATTTCCCCAACTAAAAGACCCAAACACTGTTACAGCATGACAATGCCCCTGTGCACAAAGCTCTGAGCTCCATGAAGACATGATGTGTAATGGTTGGTGTGAAAGAACTCGAGTGTCCTGCACAAAGCCCTTATGTTGATTTAACCCCATTGAACACCTTTGGGATAATTGGAACACTGACCTCCTCACCCTGCATTAGTGCCTGAACTCTCTAATCATTTTGTGGGTGACTGAGAAAATCCCCACAGCCATGCTCTGCAATTACAAGCATTTCCAGAAGTGTGAAGGTTATTACAGGAGTAAGAGTGAGACTAAATCTTCAATAGAATGTTTAAAAAACATAGAATGTGATGGTCAGGTGTCCCAATACTTTTGTCCACATAGT

>scaffold_300000130-1

CACTATATTGACAAAAGTATTGGGACACCTGACTTTAACATGTGTTTTTTGAACACCCTCTTACTCCTCTTACTCTTACTACTTTTCTGGAAATTCTTTCCACTAGAACTTGGAGTGTGACTGTGGGGATTTGCATTCATTCAGTTACAATAATATTAGTGAGATCAGACACTAATGTTGGGTGAGGAGGTCTGAGGTGCAGTCCGTGTTCCAGTTCATACTAAAGGTATTGAGTGAGGTCGAATTCAGGACTCTGTGTAGAACACTAAAGCTCTTCAATCATAACCCACCATGTCTTCATGGAGCTCAGGGGTTTTGTGCACAGGGGCATTGTCATTCTGGAACAGCCTTTGGGCCTCTTAAAAAGAAATATATTTCTACAGCATATTGAGACATCTTAGACAACTGTGTGTTTCCAACGTTGTGACAACAGTTGGGGAAGACACACTTATGTAAGTGTGCTTGGTAAGGTGTCCGAAAACATTTGGCTATATAGTG

>scaffold_300000130-8

GCTTACACTGTGTGATTTTTAATAGTCTATTGCGATTGTTACTTTTCAGACTGTACAAACATGATCCCCATGTCACACTAAGATCTCAGTTGTCATAATGTCCGACTGTACAACAGTAAAGACGCGCAAAAAACAGACGCACACAAGAAGACTTGTCCGGAGTTTTACATCATCAATCTGTGACACGTTTAGTAACTTTCGTTCTCTCGTTAAATGGTAACTAGCAGCAAACAAACAGCATTGACAACACGTCATACAAACACTTCTTTTTGCTCCATAACTCAATAAGTTTCTCTTCTTGCTGTCCTGTCCACCTGACACGTTTCGACATTTTCCCGAAATGGTTTATAGCTAAGTGAGTCATCAGGTTCGGCGCTCCTCTTGGCTCTTGGTTTGTCGTAGGAGACGTCACACTGCCGGAAAGTGTCTGAAATCTTCTGACACTGCCAGAATTTCATCAGAGAAAAAACGGATTGCAACGGTTATTAATCAGCTGTTGGTGAACATGTCAAACTAGAGATTAAAGACAACAGATTTTAGAATAGGATTATAGGAATCCTTCAAGATTTCAAAATTTGCCTCAGACAACCAAATCGTAGCCAAAATGCACAGTGTAAGC

>scaffold_300000142-1

AAGCCGGGCTTACACTGTGCGATTTTTAATAGCCTTTTGCGATTGTTGCTTGTCAGACTGTACGAACATGATCCCCATGTCACACTGTAGGATCTGTTGTCATAATGTTACTGTACGACAGTCAAAACGCATCAAAAATGGACGCACACAAGAAGACTCGTCCGGAGTTTTAAGTCCTCAATCCGTGACGCGTACAGTAACCCACGTTCTTGCTAAATGGTAGCTAGCAGCAAACAAACACAGTACATATGTAGCGGTAATTTTGCTCATGATATACGTCAAGTTTAATTTCTTGCTGTACTGTCCATCGGACTTGTTTCGTCATTTTCCCGAGGTGGTTAAAAGTTCGGCTATTGGTTCTTGGTTTGACGTCGTCGAAGTCACACTGCCGGTAAGTGTCTGAAATCTTCTGACACTGCCAGAAGTTCATCGGAGGAAAAAACTGATCGCAACAGCCATTAATCAGCTGCTAGTAAAACACGTCAAACAAGTGGTCAATGACAACAGATTTTAGCTTAGGATTATAGGAATCCTTTCAAATTTAAAAATTTGTCTCGGGACGACCAAATCGTGGCTAAAATCGCGCAGTGTAAGCCCGGCTT

>scaffold_300000153-2

AATATTCAATTTTATTTGTACAGCGTTTTAACAATAGAAATTGCCTCAAAGCAGCTTTACAGAAAAACTGAATTTAAATAAAAAATACAAATAAAAAATAAAATTGATATT

>scaffold_300000156-11

TTCATTTTTAACAGTAAATTGACAATATTGTCTTTATTGTGCCATAGCAACAACACCATTTATGTATTTCATTAAATATAAAGTTGTACAAATATTTCATTAAACAATGCAATTAAAAATGAA

>scaffold_300000174-8

TAGGGCTGCACGATACCGGGAAAATCTGCGATGTTGTTGAGTATTGCGATTACGATATATCTTGCGATATAAAATTTCCCTAGAGAAATGCTATTTTTTATTTGCTATTTTAGCTGTGGGTGTTTTTCAGGCTGGCTCATCTTATCAAGATTAGCTCGCTATGAAAACTTTGCAGCTTGTTGATTGTCTTGCGTCAGAGCACGCGCACGCAAATCCCAACTAACATCCGCGTGCCAGATATTTTACTATCCTAAAACTTTGACCGCACATTACAATTTAAAGTATTAAATCCCTCATTTATCGCAAACCATTGCGATATGCATATCTCAATAGGCACATATATCGATATATTGTGCAGCCCTA

>scaffold_300000175-21

TACGCTATATGGACAAAAAGTATTAAGACACTTGACCATCACATCCATATGTTTTTTCAACATCCTATTCAAGATTTAGTCTCACTCTTACTCCAATAATAACTTCTACACTTCTGAAAAGACTTTCTACTATATTGTGGAGAGTGGCTGTCTGGATTTGCTCATTTAGCCACAATAGTATTCATAAGATCCGACACTAATGTTGAGTGATAAGGTCTGAAGTGCAGTTGGTGTTCCAGTTCATTCCAAAGGTGTTCAGTGGGGTTAAGGTCAGGGCTCTGTGTGCAGGACACTTAAGTTCTTCCAGCTTTGGCAAACCATGTCTTAATAGAGCTCAGGGGCTTTGTGCACAGGGGCATTGTCATGCTGGAACAGTGGTTGAGCCTCTTAGTTCAAGTGAAGGAATAATGTAATGCTACAACATACAAAGACATTTGTGACATTTGTGTCTTTCTAACTTTGTAGCAACATTTTGGGAAAGACACACTAATGTGGGTGTGCTGGTAAGGTGTTCACAAACGTTTGGCCATATAGAGTA

>scaffold_300000176-1

AAGCCGGGCTTACACTGTGCGGTTTTAGACACGATTTGGTTGTCTGAGACACATTTTTAAATCCTTAAGGATTCCTATAATCCTACGCTAAAATCAGTTGTCATTTTTTTCCTCCAATGAACTTCTGGTAGAAGATTTCAGACACACCTGCACCTTCTCCAACAACGATCATCAAACCAAAAACCAATAGGAGCGCTGATGATGCAATTAGCGCGACTTTGACCCACCTCTGGAAAATGTCGAAATGAATCCGGTGGACAATGCAGCAAGAAATAAAACGTATTAAATTATGTCATGCGCAAAATTACCGCTACAGATTGTTTTTGTTTGCTACTAGCTACCATTTAGCGAGAGAACGCGGGTTACTGAACGTGTCACGGATTGATGATGTAAAACTCCAAACAAGTTTTCGTGTGTGCATCTGCTTTTGATGCGTTTTGACTGTCGTACAGTCTGACATTATGACAACTGAGATCCTACAGTGTGACATGGGGATCATGTTCGTACAGTCTGACAAGCAACAATCGCAAAAGACTATTAAAAATCGCACAGTGTAAGCCTGGCTT

>scaffold_300000176-2

TAGGGCTGCACAATATATCGAAATTATCGAAATATCGCAAATGTGCATATTGAGATATGCATATCGCAATGGTTTGCAATAAATAAGTGATTTAATACTTCAAATAGTAATGTGTGGTCAAAGTTTTAGGATAGTAAAATATCTGGCGGATGTTAGTTGATATTTGCGTGCACGTACTTTGACGCAAGACAATCAACAAGCTGCGAAGCTTTCAAAGCGAGCTAATCTTGATAAGATGAGCCAGCCCGAAAAACACCCTCAGCTAAAATAGCTAATGAAAATAGTATTTCTCTAGGGAAATGTTATATCGCAAGAAATATCATTATCGCAATACTCAACAGCAATATTGCATATCGCATATTTTTCCAGTATTGTGCAGCCCTA

>scaffold_300000178-2

TAGGCTACGTTCACACAGCAGGCCCATGTAAATGTGTTTTGCCCACATATGACTCATCTGTTTTTCCCATGACAGTGTGAACAGTACAAACCGCTTGGAATATGATCTTTTCAACTCCGATATGTGCCACTTCCATATGAGATATGAAATCCGACACAGGTCAGATGTTTTGCAATGAGATCGCATTGTGAACAGTTATGTCGGAATTCATGCGACTTTTACGTCATTTTCGATCGCCATGCGTCATCATTCTGCACTGGCGCGTAGGCTACTATTAATATATCGATATATAATCGAGGCGTTCACATAACGGTCCCACCACTTCTAACTCCGCCTCTGCATCCACACACACCTCCATACTGAAGTAGCAGCCACTGATCCACAACATGCTATTATTAAAAATATGTCCCTTTTTCTCCTCATCCTCTTCCTCCTCAACGACTGCTCATTAATTCTCTGTCGTCTGCTGCACATCACCTTATAAATGTAAGCAGCTAACGCGTAAATGCTGCCTTCAGTGTCTTCCATGTGTTTTTTTATGTATGACAGAAGGCTGCGTGAGACGTAGAATTTATTATTTTGCATGCGGGTCAGTTTAGGACCATGATCAGTTTACAATGGAAATCTGATATTGGCCACATTTAAAACAACAATGTAAACAGCTTGACAAAAAAATCTGATCTGAGCAAAAATCGGGAATTGAGCACTAAGCCTCGCAGTGTGAACGTAGCCTA

>scaffold_300000181-10

TAGGGCTGCACGATACGGGGAAAATATGCGATATTGTTGTTGAGTATTGCGACTACGATATTTCTTGCGCTATAACATTTCCCTAGAGAAATGTTATTTTTATTAGCTATTTTAGCTGCGGGTGTTTTTCAGGCTGGTTCATCTTATCAAGATTAGCTCGCTATGAAAGCTTCGCAGCTTGTTGATTGTCTTGGGTCAAAGCACGCGCACGCAAATCCCAACTAACATCCGCGTGCCAGATATTTTACTATCCTAAAACTTTGACCACACATTATTATTTGAAGTATTAAATCGCTCATTTATTGCAAACCATTGCGATATGCATATCTCAATATACACATTTGCGATATTTCGATATATTGTGCAGCCCTA

>scaffold_300000182-1

TACGGTGGCTGAGAGAGCTCAATGCGCTGCAATTTAAGAAAACACGTGCAAATAGAAAAAACACTAACAAATTAAGAAAGCATCTTCATCAGAAACGTGCTGCAAATTCTCACAACACAATAAAATACAGAAATGTGCTGAAAATTTTCACAACACAACCAATTACAGAAACGCGCTACAACTAGCACAGACCACAACGGGAATCAAGGGGACCACCAAAAAAGGGACAAATCTGGCTGGGACCTGCTTATCATTTCACAGTTAATTGTGTAATTCGTCAGATTATTTAGGCTAATAATATCCAAAAGACCAAGGAATCATACAATTGGGGTGATAAAAACTTACCATTCAGTTTGACTATAACACCACTGACAAATAGCCACTAAACAATAAGCAGGTCGTCACTTTTTGGGGTCCCTTGAAACATTTCCGTGGTGGTCTGTGCTATTTGCAGCGTGTTTCCGTATTTGTTTGCGTTGGTAGGATTTGCAGCACGTGTGTTGTCAAACTAATGAAGATGTTTTCTTAATTTGTTGGTGTTTTTTGTATTTGCATATGTTTATTTTTAAATGTGGAGCGTTGACCTCTCTTAACCACCGTA

>scaffold_300000183-2

GCTACGTTCACACTGCGAGGCTTAGTGCGAGGTCAATTCAGATTTTTACTCATAGCTGATTTTGTGTCTAGCTGTTTACATTGTTGTTTTAAATGGGGCCAATATCAGATTTCCAGTTTGAACAGATCATGGTCCTAAACTGATCCACGTGCACAAAAGAATCAATTCTACGTCTCACGCAGCCTCCTGTCATACGGGAAAAAACACATGGAAGACACTGAAGGCAGCATTTACGCGTTAGGTGCTTACATTTATAAGGTGATGTGCAGCAGACGACAGAGAATTAATGAGCAGTCATTGAGGAGGAAGAGGATGAGGAGAAAAAGGGACAAATGTTTAATAATAGCATGTTGTGGAGCAGTGGCTGCTACTTCAGTATGGAGGTGTGTGTGGATGTGGAGCCGGAGTCAGGAGTGGTGGGACCGTTACGTGATCAACGCCTTCAGCCCTCAACTGCTGGTATATCAAATGTTAATAGTAGCCTACGCGCCAGTGCAGAATGATGACGCATGTCGATCGAAAATGACGTAAAAGTCGCATGAATTCTGACATAACTGTTCACACTGCGGTCTCATTGCAAAACATCTGACCTGTGTCGGATTTAATAGCGCATATGGAAGTGGCACATATCGGAATTGAACAGATCAGATTCCATGCGGTTTGTGCTGTTCACACTGTCATGGAAAAAACAGATCGGAGTCACATGTGGGCAAAAAAATCGGATTTGGGCCACATTTGCCTGCAGTGTGAACGTAGC

>scaffold_300000183-4

ACTATATGGCCAAATGTTTGTGGACACCCTACCAGCACACCTGTAATTGTGTCTTCCCCAAACTACTGCCACGGAGTTGGAAGCACACAATTGTCTATGATGTCTATATGCTGTATTTTCCTTCACCTTCCTTAAGAGGCTTAAACACTGTTCCAGCATGACCATGTCCCTGTGCACAAAAGCCTCCAAGCGTCCTAAAGACATGGTGTGTTATGGTTGGTGTGGAAGAACTCAAATGTCCTGCACCGTTCCCTGACCTCAACCCTACTGAACACCTTTGGGATGAACTGGAACACCGACTGCACCCCAGAAATCATCACCCAGCATTAGCACCCGATCTCACTAGTCATTTCATGGATGAATGAACGAACCCCCCATAGCCACAAAGCCTTTCCAGACATGTGGAGGGGTTATTCTAGGAGTAAAAGTGAGACTAAATTGGAAATGGATGTTTAAGTAACACATATGAATGTGATGGTCGTGTGTTTAAAAATGTTTGGCTATATAGT

>scaffold_300000184-6

TAAATAAATTATATGGCCAAAAGTTTGTGGACACCTTACCAGCACACCCATAAGTCTTTCTTCCCCAAACTGCTGCCACAAAGTCTAAAGCACACAGTTGTCCAGAATGTCTTTGTATGTTGTAACTTTATATTATTCCTTTACTGGAACAAAGAGGCTGAATCACTGTTCCAGCATGACAATGCCCCTGTGCACAAAGCCCCTGATCTCCATGAAGAAATGCTGTGTTCTGGTTTCAGTGGAAGAACTTGAGTGTCCTGCAAAAAGCCCTGACTCTGACTCAACATTACTGACCACCTTTGGGATGAACTGGAACACCGACTGCACCACAGACCTCCTCACCAGACATAAGTGTGTGATCTCACTAATACTATTGTGTCGGAATAAAATAATCCCCACAGCCATGCTCTAAAATCTAGTGAAAATTCTTTTCAGAAGTGTGAAGGTTATTGTAGGAGTAAGAGTGAGAAAAAATCTGGAATAAGATATTCAAAAACACATCAATTTTATCATCAGGTGCCTCAATACTTTTGGCCAAATTGTGTATTTA

>scaffold_300000185-12

TATACAGGGTGTGTGAAAAGTAATAATTAGGCATTAATAATTGGTAATAAAAATTCTAATACAAATGTACATGCACTTTATTACATAGCAAAATGAAAGCAAATCTTTAAATTTCAACGTAGTTTAGTTTCCCCAAACTGCCGGAGATGCAAGGAAGGAAGCCGTCTGGGATATTGCTGAGAATCCATGCCTCCAAGTCCTCCGGTGTGCGAGGCTTGAAATCCTATTTACTATCGTCTATTTACACACTGTTTTCACTCATTCGGTGATGCTGCAAGTTCTACAGCCATTAAAGATTGCCTAGTTAATTTCCCCCACCCTGTATA

>scaffold_300000185-13

TTACTCTGTGCGATTTTGGCCACGATTTGGTTGTCTGAGACAAATTTTGAAATCCTAAAAGATTCCTATAATCCTATGCTAAAATCTGTTGTCTTTGATGGCTAGTTTGACATTTTCACCGACCGGCGATTAATGGCCGTTGTGATCAGTTTTTTCCTCCGATGAAATTCTGAAAGTCAGAAGATTTCAGACACTTTCCTGCAGTGTGACTTCTCCTACGACAACCGTCAAAACCAAGAACCAATAGGAGCACCGAACCTAGCGCGACAACTTAAAACAACCTCTGGAAAGTGTTGAAACGAGTCCGGTGTACAGTACAGCAAAAAAAGAAAAACTTATTCAGGGAGGTCATGAGCAAAACTATCACTACAGACACAGTTTTTGTTTTTTGCTGCTATCTATTAACCCAACCAGAATGTGGGCTACTGAATGTGTCACGGATTGATGATGTAAAAGTCCTCTTGTGTGCGTCCGTTTTTGACGTGTCTTGACTGTCGTACAGTCTGACATTATGACAACAGAGATCATACAGTGTGACATGGGGATCATGTTCGTACAGTCTGACAAGCAACAATCGCAAAGGATTATTAAAAATCGAACAGTGTAA

>scaffold_300000186-1

AAATACACTATATTGCCACAAGTTTGTGGACACCTGACCATCACAAACATATGTGATTTTAAACATCTTATTCCAGATTTACTCCCATTTTGCTGTTAGATTAAGCTCCACTCTTCTAGTAAGGTTTTCCACTACAGTTTGGAGCTTGAATGTGAGATACGTGTTCATTCAGCTACAATAGTATTATAGAGATCAGACACTAACGTCAGGTAAGGAGGTCTGTGGTGCAGTCGGCGTTCCAGTTCATCCCAAAGGTGTTCAGTGGTGTTAAGATCAGTGCTTTGTGCAGAACACTCAAGTTTTTCCACTCCAATCATAATACACCATGTCTTAATGGACCTCAGGGGCTTTGTGTACAGGGGCATTGTCATGCTGGAACAGTGTTTGGGTCTCTTAGTTCCAGTGAAGAAATAATGTTAAAGCAATAATGCTACACTGTACAAATACATTCTAGACAACTGTGTGCTTCCAACTTTGTGGCAGTTTGAAGAAGACACAATTATATTGGTGTGCTGGTAAGGTGTCCACAAACTTTTGGCCATATAGTGTATTT

>scaffold_300000188-6

TTAGGGCTGTGTATTGGCAAGGGCCTCACGATACGATACATGGGTCACAATACAATATATTGCAATACAATACATATTTAAATACATTGCAATACTTAATTTAAAAATGTCAAACAAATAGCTAAAAATACAACTGGCTGTGTAGCACATTATATTGATTAATCAGCAGACAAATAAAAACTGAGGGTTTTGATTAAATATGAACATAAACGTAAATCAGTTTAAAACGTAATTTAAATATGTATCGATACTAGAGGTTAGAATAGTGATACACTATCGTGAAAAAAAATATCGCGATAGTTAGCTGTATCGATATAGTTACACAGCCCTAA

>scaffold_300000188-19

TACACTACATAGCCAAAAGTTTGCGGACACCTTACCAAGCACACTTACATAAGTGTGTCTTCCCCAAACTGTTGTCACAACGTTGGAAGCACACAGTTGTCTAAAATGTCTCTATATGCTGTAGCATTATATTTCTTTTTACTGGAACTAAGAGGCCCAAACACTGTCCCAGCATGACAATGCCCCTGTGCACAAAGTCCCTGAGCTCCATCAAGACATGGTGTGTTATGATCAAAGAACAGAGTCCTGACAACAACCTCACTCAATACCTTTAGGATGAACTGGAACACCGACTGCACCCTAGACCTCCTCACCCAACATTAGTGTCTGATCTCACTAATACTATTCTTACTGAATGAACACAAATCCCCACAGCCAAACTCCAAAATCTAGTGGAAAGTATTTCCAGAAGTGTGGAGATTATTACAGGAATAAGACCGAGATTAAATCTGGAATATGAGGTTAAAAAAAACACACATGGATGTAATAGTCAGGTGCCCAAATACTTTTGTCAATATAGTGTA

>scaffold_300000189-1

AATACACTATATTGCCGAAAGGTTGTGGACACCTGACCATCACAAACATATGTGATTTTTAACATCTTATTCCAGATTTACTTCCTATTTTGCTGTTAGATTAAGCTCCACTCTTCTAGTAAGGCTTTCCACTATAATTTGGAGCTTGGATGTGAGATATGTGTTCATTCAGCTACAATAGTATTAGTGAGATCAGACACTAACATCAGGTAAGGAGGTCTGTGGTGCAGTCGGCGTTCCAGTTCATCCCAAAGATGAACTTAAAGTCAGGGCTTTAGGCAGAACACACTAGTTTTTCTACTCCAATCATAACACACCATGTCTTAATGGAGCTCAGGGGCTTTGTGTACAGGGGCATTGTCATGCTGGAACAGTGTTTGGTTCTCTCAGTTCCAGTGAAGGAATAATGTAATGCTACAACATACAAAGACATTCTAGACAACTGTGTGCTTCCAACTTTGTAGCAACATTTTGGCGAGGAACGACATATGGATCTGATGGTCAGGTGTCCACAAACTTTTGGCAAAATAGTGTATT

>scaffold_300000189-16

ATACGCTATATGCCCAAAAGTTTGTGGACACTTCCCAGCACAACCACCTAGGTGTGTATCCCTTCAAACTGTTGCCACAAAGTTGGAAGCACACAGTTGTGTAGAATGTTTTTGTGTTTTGTAGCATTACGTTATTTCTTCACCAGAACTAAGAGACCCAAAAACTGTTTCATCATGACAATGCCCCTGTGCACAAAACCCTTAAGCTTCATGGTGTGTTATGGTTGGTGTAGAAAAACCCAAATGTCCTGCACAGAGCCCTGAATCTGACTCAACCCCACTGAATACCTTTGGAATAAACTGGAACACCAACTGCACCCCAGACCTCCTCACCCAACATTAGTACCTCAACTCACTAGCATTGTGGCTAAAAGGAAAAAAAATGCCCACGTAGCCTTACTCCAGAATGTAGTGGAAAGCCTTTCCAGAAGTGTGGAGGTTATTATAGGAGCAAAAGGGAGACTAAATCTGAAATAGCTTTTTAAAACACACACGTGATGGTCAGGTGTCTTAATACTTTTGTCCATATAGTGTAT

>scaffold_300000190-1

AATTAGGGCTGCACAATACTGGAGAATTATCGATTTGTGATATTGTTGTTGAGTATTACAATAAAGATATTTCTTGCGATATAACATTTCCCTAGAGAAATGCTATTTTTATTAGCTATTTTAGCTGCGGGTGTTTTTCGGGCTGGTTCAGCTTATCAAGATTAGCTCGCTATGAAAGCTCCGCAGCTTGTTGATTGTCTTGCATCAAAGCACGCGCACGCAAATCCCAACTAACACCCAGATATTTTACTATCCTAAAACTTTGACCACACATAACTATTTGAAGTATTAAATTGCTCATTTATCGCATACCATTGCGATATGCATATCTCGATGTGCACATTTGCGATATTTCGATAATTTCGATATATTGTGCAGCCCTAATT

>scaffold_300000194-6

CACTACATGGCCAAAAGTTTGTGGACACCTTACCAGCACACTCACATAATTGTCTTTCCAAATGTATTTCCATAAATTTGAAAGCACATAGTTGGCTAGAATGTCTGTGTTTTAACATTACATTATTCCTTCACTGGAGCTGAGAGGCTCAAACACTGTTCCAGCATGACCAGGCTACTGTACAAAAACCGAGCTTCATACAGACATGGTGTGTTCTGGGCGGTGTGGAAGAACTCGAGTGTCCTGCACAGAGTTCTGACCACATCCCCACTGGACACCTTTGACAAAACTGGAACACTGACTGCACCCCAGATCTTCTCACCTGACAATGTCTGATCTCACTAATACTATTGTGGCTGAATTTGCAAATAACTAAATATAGTGCTATAACTAATCTTCACTATCTAGTGAAAATCATTTCCAGAAGTGTGGAGTTTATTATAGGAGTAGGAGTGACACTAAATGTGGAATAGGATGTTAAAAACACATAAATGTGATGGACCGGTGTCACAAAACTTTTGTCTATATAGTG

>scaffold_300000194-9

TACACTATATTGATAAAAGTATTGGAACACCTGACTATAACATCCATGTGTGTTTTTTGAACATCCTATTCCAGATTTAATCTCACTCTTACTACTGTGATAATCTCCACACTTCTGGAAATACTTTTCACTAGATTTTGGAGTTTGGCTGTGGGGATTTGTGTTCATTCAGCCAGAATAGTTTTAGTGAGATCAGACACTAATGTTGGGTGAAAAGCTCTGGGATGCAGTCGGCGTTCCAGTTCATCCTAAAGCTGTTGAGTGAGGTTGAGGTCAAGACTTTGTGTAGAACACTAATGTTCTTCAATCATAACACACCATGTCTTCATGGAGCTCAGGGGCTTTGTGGACAGGGGCATTGTCATACTGGGACAGTCTTTAGAAAGAAATATAATGCATATAGAGACATCTTAGACAACTTTGTGCTTCCAACATTGTGACAACAGTTTGGGGAACACACTTATATAAGTGTGCATAGTACGGTGTTCGCAAACTTTAGGCTATATAGTGTA

>scaffold_300000194-10

CAGGGCCGGCCCTGACCAATTTGCTGCCCTAGGCAAGATTTTACCTGGTGTGCCTTGATTTTGTAATTTGTTACAAAATCACATTTTACCATAGTTTACATCCTAAGTGTAGTACAGTTTGTGAACAGTACATTAGGGTGAATTTACATTAACTCTAGTTAGCTAGCTATTTCAAATTAGCAAACGTTAACAGTGCTGTCTAACAACTACTACATGGTTACATAAACCGTTCATCACATGACCTCTGTCTTTTTCCAGTTTTTCTTCTTCTTTTCTCCTTTTTCTTCCCTGGACACCAGAGGGCTTTGGTCTTTGACATTGTAAGCAGCTTTGACAGGTTGTAATGCAAATTTTCGTGTTGTGAATGATGAATCCAGCCATCTGTCTCTATCAGGTAAGTCAGAGTGCGGTGTTGAGGGGGAAGATTGACTCGTTGGACCAGGATGATGAGTCGAATGACAACTAATGCAATGTAATTAGGGTTTTTTTGTGGGGAATGGGTTCGCTTTTTTGTAATATTTCAAATAATTATAATATTTTTCAAAATAGTATTAGAAAAAAAAAATTCAATATTAAAAATTAGATTCATTCATTTGGATTTGGGTGCCCCTGTGGGTGGGCGGCACCCCTAGCATTTTCCTATTCTGCCTAATGGAAGGGCCGGCCCTG

>scaffold_300000194-14

TGGCTCCGTCCGAATTCGCATACTACTTAAGTATGTACTACATTTGAATAAGTACGTACCTCCCGCCCGTTAAAAAAGTATGTTCTATATAATATGCTTACGGGTAGTATGAATGAACTTCGGACATACTACATTGATATTGTCACGTGACCTATGTCATCACAGTAGCGCAAAAATTTAAGTTTATATAATGTTAACTTGAATGATAAAGTTCTTAAAGCTCTTTTAAACACTTGAAAAGTGCAGCGTCTTTGGTTTTTGTCACTTCCGCATATGACGAGTCGTCTCTCCCGTGGTCTCATGGGATAGTAAAATGTACATCGTATGCATACTTCAAAATCCGGGCGGAAGTAGTAGGTCATCCGGGAACTTTTCGCATACTGTATTTTGCATACTAAGGATTCGGACATACTACTCGCCTCGCATACTCAGTTTTGCATACTATATAGTATTAAAGTATGCCATTTCTGATGGAGCCA

>scaffold_300000195-13

GCTACGTTCACACTGCAGGAAAATGTGGCCCAAATCCGATTTTTTTGCCCACATGTGCCTCTGATCTGTTTTTCTCATGACAATGTAAACAGCACAAATCGCATGGAATCTGATCTTTTCAATTCTGATTTTTGCCATTTCTGTATGTGCTATTAAATCCGACACAGGTCAGATGTTTTGCAATGCGACCGCAGTATTGGAATTCATGCGAATTTTACGTCATTTCCGATCGGCATGCATCATCATTCTGCACTGGCGCGTAACTACAAACAGTTGATATATAATCGATTTCACTGAAGGCGTTAATCACGTCACGGTCCCACCACTCCTGGCTCCGGCTCCTCATCCACACACACCTCCATACTGAAGTAGCAGCCACTGCTCCACAACATGCTATTATTAAAAATTTGGCCCTTTTTCTCCTCATCCTCTTCCTCCTCAACGACTTTTCATTAATTTGCTGTCGTCCGTGCACATCACCTTATAAATGTAAGCAGCTGCTTTCAGTGTCTTCCGTGTTTATTTTTCCGTATGACAGAATGGTGCGTGTGACGTAGAATTTGTTCTTTTGCAACTGCGGCTCAGTTTAGGACCATGATCAGGTCACACTGTAAATCTGATATCGGCCACATTTAAAACAACAATGTAAACAGCTATACAAAACAATTCTGAGAAAAAATCAGAATTGAGCACTAAGCCTCACAGTGTGAACATAGC

>scaffold_300000196-5

ACTAAGGCTACGTTCACACTGCAGGAAAATGTGGCCCAAATCCGAATGTTTTTGCCCACTTGTCACTCAGGTATGTTTTTTTCATGACAGTGTGAACAGCACAAACCGCATGGAATCTGATCTTTTCAATTCTGATTTGTGACACTTCCATATGAGATATTAAACCCAACACAGGTCAGATTTTTGCAATGCGACCGCAGTGTGAACAGTTATGTCGGAATTCGTACAACGTTTACGTCATTTTCGATCGACATGCGTCATCATTCTGCACTGGCGAGTAACTACAAACAGTTGATATATAATCGATTTTGCTGAAGGCGTTAATCACGTCACGGTCCTACCACTTCTGGCTCCGGCTCCTCATCCACACACACCTCCATACTGAAGTAGCAGCCACTGGTCCACAACATGCTATTATTAAAAATTTGGCACTTTTTCTCCTTATCCTCTTCCTCCTCAACGACTGCTCATTAATTTACACACCGCTGCACATCACCATTTAAATGTAAGCAGCTGCCTTCAGTGTCTTCCGTGTTTATTTTTGCGTATGACAGGACACTGCGTGAGAGGTAGAATTTGTTCTTTTGCGCATGCGGGTCAGTTTAGGACCATGATCAGGTCACACTGGAAATCTGATATTGGCCACATTTAAAACAACAATGTGAACAGCTATACAAAAAAATCTGATCTCTGAAATCAGAATTGAGCACTAAGCCTTGCAGTGTGAACGTAGCCTAAGT

>scaffold_300000197-24

TTTAACCCTCATATGGTGTTAATGTTTTTGTTACCTAGCCAATGTTCACGGGTCTGGTGGACCCACCGCATTATTTGGTCTTTAATCAACCCAGCCTTAACAATTTATAGAAAAACACCTAAAAGAGGTTTACTTTATACTTTATAGACATCATTTATGGTTAATATTTGCTATTTACCCCGTTAGATCACATTTGAAAATATAAGTGTTATGGAAAATATGTTCTATTTTATGTTCTACATGGAAAATGAGCCAAGGCAGTGAGTCTGGGGCTAAAAAAAAATTATAGCGTAATTTCTCTTTAAACAAACTTTGAAATCGGGTCCCACAGACCCAAACACCACACAAGGGTTAAA

>scaffold_300000198-1

AGGCTACGTTCACACTGCAAGGCTTAGTGCTCAATTCCGATTTTTGCTCAGATCAGATTTGTTTTTATAGCTGTTCACATTGTTGTTTTAAATGTGGCCAATATCAGATTTCCAGTGTGAACTGATCATGGTCCTAAACTGATCCACATGTGCAAAAGAACAAATTCTACCTCACACACAGCGTCCTGTTATGCAGAAAAATAAACACGGAACACACTAAAGGCAGCATTTACACGTTAGGTGCTTACATTTATAAGGTGATGTGCAGCGGACGACAGAAAATTAATGAGCAGTCGTTGAGGAGGAAAAGGATGAGGAGAAAAAGGGCCAAATTTTGTATAATAGCATGTTGTGGAGAAGTGGCTGCTACTTCAGTATGGATGTGTGTGTGGATGCGGAGCCGGAGCCATGAGTGGTGGGACCGTGACGTGATTAACGCCTTCAGCAAAATCGATTATATATCAACTGTTAATAGTAGCCTATGCGCCAGTGCAGAATGATGACACATGTCGATTGAAAATGACGTAAAAGGCACATGAATTCTGACAATTCTGACTGTTCACACTGCGGTCGCATTGCAAAACATCTGACCTGTGTCGCATTTAATACCACATATGGAAGTGGCACATTCACACTGTCACTTGTCACACAGATCTCAGTCACATGTGGGCAAAAAAATCGAATTTGGGCTACATTTGCCTGCAGTGTGAATGTAGCCT

>scaffold_300000199-28

TAGGGATGCACCGATACTGAGCTCATGTACTCGTACTCGTAAAAACACTCCACCTCTTGTGACGTCATTGACAGTGTCAGTGTACAAAGACTCTGGCAGCAGCGACAGAATGTCAGGCTCAGCGGTATGGAAATATTTCAAAATCAATAATGACAACCCACGCATGGCAGACTGCAAACTTTGTTTGACAAAAATATTAAGAGGAAAAAAAATTGAATGCATAAAATATGAGTAATCTGATAAAACATCTCAAATTAAAACACAAGTGAGCAAGGAGAATTTTCCACTAGCAGTAACTTTAGCACTCAACAACCAACCCTGCAGCAAACCCTTGCAAGATGAGAGAAAATGGCAAGAGAAAACTAGAGAGCCATACAAATAATCCCACATCCCAAATAGTACAGGTATCGGTATTGGTGAGTACCAGGAAAAAAATATCGGTACTCGTACTCTGTCTTTTAAAAATGGTATTGGTGAATCCCTA

>scaffold_300000200-2

ATATACACTATATTGCCAAATGTTTATGGACACCTGACCATCACAAACATATGTGGTTTTAAACCTCTTATTCCAGATTTACTCCCCATTTTTCTGTTAGATTAAGCTCCACTCTTCTTGTAAGACTTTCCACTACAATTTGGAGCTTGAATGTGAGATATGTGTTCATTCAGCTACAATAGTATTAGTGAGATCAGACACGAACGTCAGGTAAGGAGGTCTGTGGTGCAGTCGGTGTTCCAGTTCATCCCAAAGGTATTTAGTCGTGTTAAAGTCAGGGCTTTGTGCAGAACACTCAAGTTTTTCCACTCCAATCATAACACAGCATGTCTTAATGGAACTCAGAGGTTTTGTGCACAGGGGCATTGTCATGCTGGAACAGTGTTTGGGTTTCTCAGTTCCAGTGTATAACATACAAAGACATTCTAGACAACTGTGTGCTTCCAAATTTGTGGCAACATTTTGGAGAGGAACAACATATGGATCTGATGGTGAGGTGTCTCACAAACTTTTGGCAATATAGTGTATAT

>scaffold_300000200-3

CACTATATAGCCAAAAAATTTTGGACACATGATCATCACAATCATATGTGTTTTTTAAACATCTATTTCCCATTTAGCCTCACTTTTACTCTTATAATAACCCCTCCACATTTTTGGAAAAGCTTTTTACTCAAATATGGTGTATGGCTATGAGGATTTGTTCATTCAGCCATAAAATAGGAAATACTAGTGAGAACGGGTGCTAATGCTGGTTGACGAGTTCTGGGGTCAAGTTGGTGTTTCCGTTCATCCCAAAGGTGTTCGGTGGTGTTGAGGTCAAGGAACTGTGCAGGGCATTTGAGTTCTCCAACACCGACCATGATATATCGTGTCTTAGGACGCTTAGAGGCTTTGTGCACAGATGCATTGTCATGCTGGAACAATGTTTAAGCCTCTTAGTTCCAGCAATGGTAAATCTTAATGCTACAGCATATAGAGACATTATAGACAACTGTGTGCTTCCATCTCTGTGGCAGTAGTTTTGGAAAAACACAATTACGAGTGTGCTGGTATGGTGTCCACAAACTTTTGGCCATATAGTG

>scaffold_300000200-8

GGCTCCATCCAAAATTGCATACTTCCATGCTATATAATATGCGAAACTGAGTATGTGAAGCGAGTAGTACAGCCGAATCCTTAGTATGCAAAATACAGTACGCGAAAAGTTCTGGATGACCTACTTATTCCGTCCGGATTTTGAAGTATGCATACGATGTACACTTTACTATCCCATGAGCCCACAGGAGAGGCGACTCTTCATATGCGTAAGTGCCGAAAAGTGGCAACGCAGCACTTTTCAAGTGCTTTAAGTAAGAAAAAAACATTGTTCCTTGTAGTTAAATTGCACTCGTTTAACATCATTCAAGTTAAACATTACCCAACTTTAAATTCGCGCTGCTGTGACGTCGTAAGTCACGTGTCAATATCAACATGGCGGATGTAGTACGTCCGAATTTCATTTCATTACTACACGTGTCATACTATATAGAATATACTTTTTTAACGGTCGGGAAGTACGTACTTATTTAAATATAGTACATACTTAAGTAGTATGCGATTTCGGACGGAGCC

>scaffold_300000201-1

CACTATATTGCCAAAAGTTTGTGGACACCTGACCATCAGATCCATATGTGGTTCCTCTCCAAAATGTTGCTACAAATTTGGAAGCACACAGTTATTTAGAAAGTTTTTGTATGTTGTAGTATTACATTATTCCTTCACTGGAACTGAGAGACCCAAACACTGTTCCAGCATGACAATGCCCCTGTGCACAAAACCTCTGAGTTCCATTAAGACTTGGGGTGTTATGATTGGAGAGGAATAACTTGAGTGTTCTGCACAAAGCTCTGACATTAACACCACTGAACACCTTTGGGATGAACTGGAACACCGACTGCACCACAGACCTCCTTACCTGACGTTAGTGTGTGATCTCACTAATACTATTGTAGCTGAATGAACACATATCTCACATTCAAGCTCCAAATTGTAGTGGAAAGAGTTACTAGAAGAGTGGAGCTTAATCGAACAGCAAAATCTGGAATAAGATGTTTAAAATCACATTTTTATTTTATTTCAGGTGTCCATCAACTTTTGGCAATATAGTG

>scaffold_300000201-4

ATACACTATATAGACAAAAGTATTGGGACACTTGACCATCACTTGTTTTTTGAACATCCTATTTCAGATTTAGTCTCACTCTTCCTCCTATAATAACCTCCCCACCTCTGAAAAGGCTTTCCATTAGATTGTGGAGTGTGACTGTGGGGATTTGTGTTTATTTATCGACAATAGTATTCAGACACTAATGTTGGGTGAGGAGGTCTTGAGTGAGAAGGTCTGGGGTGCAGTCGGTGTTCCAGTTCAAAGGTGTTGAGTGGGGTTGAGTCAGAGTCAGAATCAGGGCTCTGTGTAGGATACTTGAGTTCTTCCACTCCAATCATAACACACCATGTGTTCATGGAGCTCAGGGGTTTTGTGCACAGGGGCATTGTCATGCTGGAACAGTGTTTGGGTCTCTAAGTTCCAGTGAATTAAGAATGTAATGCTACAAAATATAGAGACATTATAGACAACTGTGTGCTTCAGAGTTTGTGGTAACAAACACTAAGACGCACTAATGTGGGTGTGTTGGTAAGGTGTCCCCAAACGTTTGGCAATATAGTGTAT

>scaffold_300000202-2

TACACTATATTGCCAAACGTTTGTGGACACCTGACCTTCAGATCCATATGTTGTTCCTCTCCAAAATGTTGCTACATTTGGAAGCACACAGTTGTCTAGAATGTCTTTGTATGTTGCAGCATTACATTATTCCTTCACTGGAACTGAGAGACCCAAACACTGTTCCAGCATGACAATGCCCCTGTGCACAAAACCCCTGAGCTCCATTAAGACATGCTGTGTTCTGATTGGAGTGGAAAAACTCGAGTGTTCTGCACAAAGCTCTGACCTGAACACCACTGAACACCTTTGGGATGAACTGGAACACCGACTGCACCACAGACCTCCTTACCTGACGTTAGTGTCTGATCTCACTAATACTACTGTAGCTGAATGAACACAAATCTCACATTCAAGCTCCAAATTGTAATGGAAAGAGTTACTAGAAGAGTGGTGCTTAATCGAACAGCAAAATGGGGAGTAAATCTGGAATAAGATGTTTCAAATCACACATGTTTGGGATGGTCAGGTGTCCACCAACGTTTGGCAATATAGTGTA

>scaffold_300000202-11

CTATATCTTCCTGAAACCCAAGGGAAAAAAGCGTCATTATTTTTTTTTATGGTTTTGTGTGATTTTCTACTTCTTTTGGGCTAAAAAAACATTCTACAATTTAGTGTTTTTCAATTTTATTGTGATTTTTTATTTAACAGCATGTCCACTGTAGTGGATCACAGGAACATTTTTGTTTGAAAAGATAGTCCTTTCCATAAAATGTGCTTGTTCCTATGGCGGCCATTTTGGATGCAGTGTAAGAACTAGTTCCTAGCATTCCAACCAATCAAATGACATATCACTAGAAATCCCAGGATGTCCTCTGTACAGTACAGCAGAAATTGATAGAGTTGCTCAAAAAAATTCAAAAAACATTTATGAAAACCAAATGTCCACTACAGAGGACACAAGTCAGTGGGCGGGGTCTCAGGAGGATATAG

>scaffold_300000202-12

TTAACCCTCATGTGGTGTTCATGTTTTTGTTACCTAGCCGACGTTCACGAGTCTGGTGGACCCACCGCATTATGTTTTTAATCAACTCAGCAATAACAATGTATGGAAGAACACTTAAAAAAATGTTTACTTACAAGCAATATAGACATCATTTATGGTTACTATTTGCCATTTACCCCATTAGATCACATTTGAAAATATAAGTGTTGTGAAAAATATGTTCTATTTTATGTTCTACATGGAAAATGAACCAAAGCCAATGAGTCTGGGGCTAAAAAAAATGAATAGCCTAATTTCTTTTTAAACAAACTTTAAGGGAAGCGGGTCCCACAGACCCAAACACCACACAAGGGTTAA

>scaffold_300000204-4

AGTAGGGATGCACCGATACGGATACTAGTATCGGTTTTGGGCCCGATACTGAGCTCATATACTCATACTCATAAAACACTTCGATACCAAAGACTGATACCTCCTGTGACATCATTGATAGAACTTTCGGTGCACAAAGACTCCGGCAGCAGCGACAGAATGTCAGGCTCAGCGGTGGGAAAATATTTCAGAATGAATGAGGACAACCCACACATGGCAGACTGCAAACTTTGTTCTGCAAAAATATCAAGAGGTACAAAAATGAGTACTTACAAAACGAGTAATCTGATAAAACATCTGAAATTAAAACACAAAAGTGAGCACGGAGAATTTTCCGCTAGCAGTAACGTTTGGCAACCAACCCTGCAGCAAACCCTTACAAGACGAGAGAAAATGGCAAGAAAAAACCTGAGAGCCATACAAATAATCCCACATCCCTAAAAAAGGACAGTTATCGGTATCGGCATGTACCAGGAAAAAAATATCGGTACTCGTACTCGGTCTTTAAAAAATGGTATCGGTGCATCCTTACT

>scaffold_300000205-13

TAGGGCTGTGTGAAAATATCGATACACTAACTATCGTGATCATTTTTCTCACAATAGTGTATCGATATTCTAACCTCTAGTATCGATACATATTTAAATTATGTTTTAAATTGATTTACGTTTACGTTAAATTTTAATTAAAACCCTCAGTGTTTATTTGTCTGCTGAGTAATCAATATAATGGGATACACATACTAGAATCCCCCAGGTGATACACAGCAAGCTGTATTTTTAGCTATTTGTTTTACATTAAAAATTTGTTTTTTTAAGTATTTCAATGTATCGTGATATGTATCGTATCGTGAGACCCTTGCCAATACACAGCCCTA

>scaffold_300000206-2

TACATTATATTGCCAAAAGTTTGTGGACACCTCACCATCAGATCCATATGTGGTTCCTCTCGGACAGTTGTCACAAATTTGAAAGCACACAGTTGTCTAGAATGTCTTTGTAGGTTGTAGCGTTACATTATTCCTTCACTGGAACTAAGAGACCCAAACACTGTTCCAGCATGACAATGCCCCTGTGCACAAAACCCCTTAGCTCCATGAAGACATGCTGTGTTATGATTGGAGTGGAAGAACTCGAGTATCCTACACAAAGCTCTGAACTGAACACCACTGAACACCTTTGGAACGCCGACTGCACCCCAGACCTCCTTACCTGACCTCACTAATACTATTGTAGCTGAATGAACACACATCTTACATTTAAGCTCCAAATTGTAGTGGAAAGTCTTACTAGAAGAGTGGAGCTTAATCGAACAGCAACATGGGAGTAAATCTGGAATAAGAGGTTTAAAATCACATGTTTGTCATGGTCAGTTGTCCACCAACTTTTGTCAATATAGTGTA

>scaffold_300000207-3

AACCCTTATGTGGTGTTCATGTTTTTGTTACCTAGCTAACATTCACGGGTCTGGTGGACCCACCGCAAGTATTGGGTTTTTAATTAACATTGCCAAAACAATTTACGGAAAAAAACTTAAAAGATGTTTACTTTAGCTCACTTATAAGCAATATAGACATCATTTATGATTAATATTTGCCATTTACCCCGTTAGATCACAATTGAAAATATAAGTTATGGAAAATATGATTATTTTATGTTCAAGATGGAAAATGAGCCACGGCCCATGAGGCTAAAATAATCATTAATAGCATAATTTCTCTTTAAACAAACTTGAAATCGGGTCCCACAGACCCAAACACCACACAAGGGTT

>scaffold_300000207-7

ATTAGAGATGAGAAATCCAAATATATTTATGGATTAAATTCTGAATCGCTCATTCAACGGATCCGAGTCTCACAAGTTCTTTGAGTCACTTGAGTCAGTAGGGATTCCGAGGGCTTTTGCCATAGTATTCCACCAGAGGGCACACACCAAATCCCTAGAATCCACTGAATCCTTGTACTCATGAATGTCACACACGGAGTCCCTCGGATCCGGCTGTTTCGCTACGCTACGGTTCAGATGAATCAGATGAATCAGTACCTGCGATTTTGAGTCTTTTATGTTTCTGACCATGCAAGAAGTTTCCTTATTCCTACAGTTACTAGTTAACAACTACTTAGAACAACTGAATGGTGTCATTTTCAGTTTGTATTTATCTATGTTGTTGTTAGCTATGTTTACTGATTCGAGTAAATGAAGAGAAGGATTCATGAATCCCGTGTCATACAAAGGATCGGGTTATTTAACCAGGATGAATCCGGATTCGCTCATCTCTAAT

>scaffold_300000207-11

TAGGGCTGTGTAAAAATATCGATACAGCTAACTATAGCTATAATGTTTTTCAAGATGGTGTATCAATATTCTAACCTCTAGTATCGATACATATTTAAATGGCGTTTTAAACTGATTTCCGTTTCCGTTAATATTTAATTAAAACCCTCAGTGTTTATTTGTCTGATGAGTAATCAATATAATGGGATACACATACTAGAATCCCCCAGGTGATACACAGCAAGCTGTATTTTTAGCAATTTGTTTTACATTTACATTTTTTTATAGTATTGCAATGTATTGTAATATGTATTGTATTGCAATATATCGTGATATATTGTATCGTGACCCATGTAACGTGATATGTATCGTATCGTAAGGCCCTTGCCAATACACAGCCCTA

>scaffold_300000207-13

TACACTGTAAACCCTAATGTTGTAAACCCTTACTTAAATGATCAAGTTATGTTGATTAAACATTTGCATATTGGACCCGTAACTCCAAAAAATAAGTTATTGTCACTTATTAACCTTTAAAGTACATGAACTTTCTGTATGATTCATTAAATTAGCTTACGCTGGTCTTCCTTTAATGTGATTGGAAAGCAAAAGAACTAATGCGCTGATTGGTGGATTATTACAAAAGGCGGAGCTTTATGTCTTGTCTGTTACTGTGAAGGAGAGGACGGGAGTTTGTCATTGTTTTAAGTGAACTTAATAGTTTAAGTACATTATATAAACACTAAATTAGATAAACTTGAAAATTTAAGTACAGTCTACATGAGCACCAAGTTATTTGAACTTAAATATCTAAGTTTTTGACCCATTACTCAAGTTACTGAAGGAACGGGTTTACTCAAAACAATTGAGTTCATTCAACTTATTAGGGTTTTTAGTGTA

>scaffold_300000208-3

TCTAAATTAGGGCTGCACAATATATCAAAATTATTGAAATATCGCGAATGTGCATATCGAGATGGTTTACGATAAATGAGCGATTTAATACTTTAAATAATAACGTGTGGTTAAAGTTTTAGGATAGTAAAATATCCGGTACGCGGATGTTAGTTGGGATTTGCGTGCGCATGATTTGACGCAAGACGATCAACAAACTGCAAAGTTTTCATAGCGAGATAATCATGATAAGATGATAAGCCCGAAAAACACCCGCAGCTAAAATAGCTAATACAAATAGCATTTCTCTAGGGAAATGTTTTATCGTTAGAATATCGTTATCGAAATACTCAACAACAATATCGCATATTTTCCCAGTATCGTGCAGCCCTACTCTAGA

>scaffold_300000209-1

AAGCCGGGTTTATACTGTGTGATTTTTAATAGTCTTTTGCGATTGTTGCTTGTCAGACTGATGATCCCCGTGTCACGCTGTAGGATCTCAGCTGTCATAATGTCAGACTGTACGACATTCAAGACGCGTCAAAAACAGACGCACACAAGAGACCCGGAGTTTTACTTCACCAATCTGAGACACGTTCAGTAACCCGCTTTCTCTCGTTAAACGGTAGCTAGCAGCAGCGACGACACGTCATACACACACTCCTTTTCCCTATATAACTCAATAAGTTTCTCTTCTTGCGGTTCTGTCCACCGGACCAGTTTCCACATTTTCCCGAGGTGGTTTGAAGTTGTCGTGCTAATTGCGTCATCAGGTTCGGCGCTCCTATCGGTTCTTTGTTTGACGGTCGTCGTAGGAGACGTCACACTGCAGGAAAGGGTCTGAAATCTTCAGCCAGAATTTCATCAGAGGAAAAACTGATCGCAACGGCCATTAATCGTCTGTCGGTGAACACGTCAAACTAAAATGATCAAAGACAACAGATTTTAACGTAGAATTATAGGAATCTTTTCATAATTTGTCTCAGACGAACAATTGTGGCCAAAATCGCACAGTATAAGCCCGGCTT

>scaffold_300000209-2

GCTACGTTCACACTGCAGGTAAATGAGGCCCAAATCCGATTTTTTTTGCCCACACGTGATTCAGATCTGTTTTTTTTATCATGACAGTGTGAACAGCACAAACCGTATGGAATCTGATCTTTTCAATTCCGATTTGTGCCACTTCCATATATGGTATTAAATGCGATACAGGTCAGATGTTTTGCGATGCGACCGCAGTGTGAACGGTTATGTCAGAATTCATGCGACTTTTACGTCATTTTTGATGGACGTGCGTCATCATTCTTCACCGGCGCGTAACTACAAACAGTTGATATATAATCGATTTGGCTGAAGGCGTTGATCACGTGACGGTTCCACCACTCCTGACTCCGGCTCCACATCCACACACACACCTCCATACTGAAGTAGCATCCACTGCTCCACAACATGCTATTATTAAACATTTAACCCTTTTTCTCCTCTTCCTCCTCAACGACTGCTCATTAATTTACTGTCGTCTGCTGCACATCACCGTATAAATATAAGCAGCTAACGCGTAAACGCCGCCTTCAGTGTGTTCCGTGTTTATTTTTCCGTAGGACAGGCTGTGTGAGGCGTAGAATTAGTTCTTTTGCACATGCGGGTCAGTTTAGGATCGTGATCAGTTCACACTGCAAATCTGATATCGGACACATTTAAAACAATGTGAACAGCTATACAAAACAATACTGATCGGAATTGAGCACTAAACCTCGCAGTGTGAACGTAGC

>scaffold_300000209-3

CTTTAAAGGGTCATGAACTTCCTCAGTTTTTTTATTTTGTACTGTTCTCTGAGGTCCACTTATAATGTTATCAAGATTTTCACATAAAAAAAAATCATCATTAAAAAGTAATAGAAAACGTGCTGTTTTTGACCCCCTCATCAGAACACTCTGTTTGAATAGGCGTGGCAGATTGTTGTCTCGGAAGGAAACGCCCACTTCTATGATTGGCTAACGGTTTTATGCCGCTCTTGTCATTTACCTATCACGTGCATGCGTGAATCGGTGGGCGGGGCTAAACAGACAGTGACGTCAAAGCAGGCGTCGATCTTCTTCTGTGGAGGCGGAGCTTATCCACACTATTACATCATCGAGTAGAACATTCCAAAACCTGTCGTTTTAACCGACTGCCTTCAATATGACCTGTTTTTAGAGTAACAACAAAGTTTTGAGTTCTGGTTTTTATATCACAACGGCCTCTTATATGTCAAAATAATTTAGATTTCTCACTTCATGACCCCTTTAAAG

>scaffold_300000210-3

ACTAGTGATGCACCGATACCGATACTGAGCTCATGTACTCGTACTCGTAAAAACACTCCGATAGCAAAGACCGATACCTCTTGAGACGTCATTGACAGAAGTTTCAGTGCACAAAGACTCCAGCAGCAGCGACAGAATGTCCGACTCAGCGGTGTGGAAATATTTCAAAATTAATGATGAACCCACGCATGGCAAACTGCAAACTTTCCACAAAAATATCAAGCGGTACAAAAATGAGTACTTATAATATGAGTAATCTAATAAAACATCTGAAATTAAAACACTAAAGTGAGCACGGAGAATTAGCCGCTAGCAGTAACGTTAGCACTCGGCAACCGACCCTGCAGCAAACCTGTGCAAGACAAGAGAAAATGGCAAGAGAAAACCAGAGAGCCATACAAATAATCCCACATCCCAAAAAAGTACAGGTATTGGTATCGGCGAGTACCAGGAAAAAAATATCGGTACTCGTACTCGGTCTTTAATAAATGGTATCGGTGCATCTCTAGT

>scaffold_300000212-2

TATATTGCCAAAAGTTTGTGGACACCTGACCATCAGATCCATATGTGGTTCCTCTCCAAACTACTGCTACAAATTTGGATGCACACAGTCGTCTAGAACGTTTTTGTATATGTTGTAGCGTTACATTATTCCTTCACTGGAACTAAGAGACTCAAACCCTGTTCCAGCATGACAATGCCCCTGTGCACAAAACCCCTGAACTCCATGAAGACATGCTGTGTTCTGATTGGAGTGGAAAAACTTGAGTGTTCTGCACAAAGCCCTGATTTTAACACCACTGAACACTTTTTTGAATGAACTGGAACACCGACTGCACCCCAGACCTCCTCACCTGACGTTAGTGTCTGATCTCACTAATACTATTGTAGCTGAATGAACACACATCTCACATTCAAGCTCCACATTGTAGTGGAAAGTCTTACTAGAAGAGTGGAGCTTAATCGAACAGCAACATGGGAGTAAATCTGGAATAAAATGGTCAGGTTTCCACAATCTTTTGGCAATATA

>scaffold_300000212-4

GTTTAAACTGTGCGATTTTTAATAGTCTTTTGCGATTGTTGTTTGTCAGACTGTACAAACATGATCCACATGTCACGCTGTAGGATCTCAGTTATCATAATGTCAGACTGTACGACAGTCAAGACGCGTCAAAAACGAATGCAAACAAGAAAACTCATCTGGAGTTTTAAGTCATCAATCCGTGACACGTTCAGTAACCTGCGTTCTCTCGCTAAACGGTAGCAAGCAGCTAACAGACAGCAGCGACAACACATTATGCAAACACTCCTTTTTCCTCCATAACTCAATACGTTTCCCTTCTTGCGGTTCTGTCCACCAGACTCATTTAGACATTTTCCCCAGGTGGTTGAATTTTTTTCGTGCTAATTGCGTCATCAGGTTCGGCGCTCCTGTTGGTTCTTGGTTTGACGGTCGTCGAAGGAGACGTCACACTGCAGGAAAGTGTCTGAAATCTTTTGCCACTGCCAGAATTTCATCGGAGGAAAAAACTGATCGCAAGGGCCATTAATCGGCTGTCGGTGAACACGTCAAACTAGCCATCAAAGACAACAGATTTTAGTGTAGGATTATAGGAATTTTTTAGGATTTCAAAAATTGTCCCAGACGAACAAATTGTGGCCAAAATCGCACAGTGTAAAC

>scaffold_300000212-9

ACTACCCAGCTAACACATTCATGTTGTGATTACGTTTCTCAAACGTTCTGTTTTGGTTTTGGGAACGTGATTCGAAATGTTCGAAAAACGTAAAAAAAATGTCCAGTTTGCTTAACTTTGGTAGAACGTTATTTTATGGTTATTTTATGGTAACGTTTTCTCTAAACATTTTGAGAACATTTATCAAGTACGAACGACGCCATAAGGACGTTCCAAGAACATTGTTCTAAGAACGTTTTCTCTCAACATTGCAAAAACATAGCTTAACTATTAATAACGTTATGCGAACGCTCCATGAACATTACTTTAGGAACGTTTTGTGCTAACATTTGAACAACTTTTAAAGAGCGTTAGCGAAAGTTGTGGGAACGTTCCCTGTTAGCTGGATAGT

>scaffold_300000215-4

ATATCCTCCTGAGACCCCACCCATTGACTGCTGTCCTCTGTAGTGGACATTTTGTTGTCATGAATGTTTTCTGAATTTTTTGAGCTAGTCTATCAATTTCTGCTGTAATGTACAAAGGACATCCTGGGATTTTGTGTGATATGTCATTTGATTGGCTGGGATTCTAAAAACTAGTTCTTACTCTGCATCCAAAATGGCCGCCATAGGAACAAGCACGTTTTATGGAAAGGTCTGTCTTTTCAAACTAAAATGGTTGTCTGATCCACTACAGTCGGTAAAATTAAAATGAACAATGACATTTTAAAACTCTAAATTGTAGAATGTTTTTGAACCCGAAAGGCGTAGGAAATTACACAAAACAAATGATGACACTTTTCTTTTTTCTCAGGAGGATAT

>scaffold_300000216-7

AAGCCGGGTTTACATTGTATGATTTTAGCCACGATTTGTTCGTCTGAGACAAATTTTGAAATCCTAAAAGATTCCTATAATCCTACACTAAAATCTGTTGTCTTTAATCGCTAGTTTGACGTGTTCACCGACAGATTAATGACCGTTGCGATCAGTTTTTTCCTCCAATGAACTTCTGGCAGAAGATTTCAGACACTTTCCTGCAGTGTAACGTCTCCTACGACGACCATCAAACCAAGAACCAATAGGAGCGCCGAACCTGATGACGCAATTAGCACGACAACTTCAAACCACCTCGGGAAAATGTGGAAACGAGTCCGGTGGACAGAACAGCGAGAAGAGAAACTTATTGAGTTCTGGAGGAAAAAGGAGTGTTTGTATGACGTGTCGTCGCTGCTGTTTGTTTGCTGCTAGCTACCATTTAACTCCGTATGAGTCTTCTTGTGTGCGTCCGTTTTTGATGCATCTTGACTGTCGTACAGTCTGACATTATGACAACTGAGATCCTACAGTGTGACACTGGGATTATGTTCGTACAGTCTGATAAGCAACTATCGCAAAACACTTTAAAAAATCACACAGTGTAAACCCGGCTT

>scaffold_300000216-8

TATATCCTCCTGAGACCCAAGGAAAGAAGCGTCATCACTTTTTTTTGTTTTGTGGGATTTCCTAAGCCTTACACGTTAAAAAACATTCTACAATTTAGAGTTTTTAAACTTTATTGCTATTTTTAATTTTACAACATGTCCACTGTAGTGGATCACATGACCAATTTAGTTTGAAAAGACAGTCCTTTCCATAAAACGTGCTTGATCCTATGGCGGCCATTTTGGATGCAGTGTAAGAACTAGTTCCTAGCATCCCAGCCAATCAAAGCCCGGGATGTCCTCTGAACAGTACAGCAGAAATTGATAGAGTAGCTCAAAAAAATTCCAGAGGACACCAGTCAATGGGCGGGGTCTCAGGAGGATATA

>scaffold_300000216-9

TAAAGCTACGTTCACACTGCAGGCAAATGTGGCCTAAATCCGATTTTTTTTGCCCACATGAGACTCAGATCTGTTTTTCTCATGACAGTGTGAACAGCACAAACCGCATGGAATCTGATCTTTTCAATTCCCATTTCCAGATGTGCTATTAAATGCGACACAGGTCAGATGTTCTGCGATGCGACCGCAGTGGGAACAGTTATGTCGGAATTCATGCGACTTTTACGTCATTTTTGATGGACATGCGTCATCATTCTGCACCGGCGCGTAACTACAAACAGTTGATATATAATCGATTTCACTGAAGGCGTTGATCACGTGACGGTTCCACCACTCCTGACTCCGGCTCCACATCCACACACACCTCCATACTGAAGTAACAGCCACTGCTCTACAACATGCTATTATTAAAACTTTAACCCTTTTTCTCCTCTTCCTCCTCAACGACTGCTCATTAATTCACTGTCGTCTGCTGCACATCACCTTATAAATATAAGCAGCTAACACGTAAACGCCGCCTTCAGTGTCATCCGTGTTTATTTTTCCGTATGACCGGCTGTGTGAGGCGTAGAATTAGTTATTTTGCGCATGCAGGTCAGTTTAGGATCGTGATCAGTTCACACTGCAAATCTGATATCGGACACATTTAAAATAACAATGTAAACAATTATACAAAAAAATCGGATTTAAGCAAAAATCTGAATTGAGCACTAAACCTTGCAGTGTGAACGTAGCATTA

>scaffold_300000216-17

TTTACACTGTGCGATTTTGGCCACGATTTGGTCGTCTGAATCATATTTTGAAATCCTAAAACATTCCTTTAATCCTACACTAAAATCTGTTGTCTTTGATATTTTTAGTTAGACTTGTTCACTGACAGACGATTAATGACCGTTGTGATCATTTTTTACCGCCGATGAAATTCTGGCAGAAGATTTCAGACACTTTCCTGCAGTGTGACGTCTCCTACGACGACCGTCAAACCAAGAACCAACAGGAGCGCCGAACCTGATGACGCAATTAGCACAACAACTTCAAACCACCTCGAGAAAATGTAGAAATGAGTCCGGTGGACAGAACAGCAAGATGAGAAACTTATTGAGTTAAGTCATGAGCAAAATTAACGATACAGATTGTTTTTCTTTGCTGTTAGCTACCGTTTAGCGAGAGAACGCTGGTTACTGAACATGTCACGTGATGTAAAACTCCGGACGAGTCTTCTTGTGTGCGTCCGTTTTTGACGCGTCTTGACTGTCGTACAGTCTGACATTATGATAACTGACATCCTACAGTGTGACACGGGGATCATGTTCGTACAGTCTGACAAACAACAATCGCTAAAGACTATTAAACATCACACAGTGTAAA

>scaffold_300000217-2

ATACACTATATTTCTCAAAAGTTTGTGGACACCTGACCATCACAAACATATGTGATTTTAAATATCTTATTCCAGATTTATTCCCATTTTGCTGTGCGATTAAGCTCCACTGTTCTAGTAAAACTTTCCACTACAATTTGGAGCGTAAAAATGTGAGATTTGTGTTCATTCAGCTACAATAGTATTAGTGAGATCAGACACTAACGTCAGGTGAGGAGGTCTGGGGTGCAGTCGGCGTTCCAGTTCATCCCAAAGGTGTTCAGGGGTGTTAAAGTCAGGGTTTTGTGCAGAACACTCAAGTTTTTCCCCACCAACCAGAACACAGCATGTCTTCATGGAGCTCAGGGGCTTTGTGCACAGGGGCATTGTCATGCTGGAACAGGGTTTGAGTCTCTTAGTTCCAGTGAAGGAATAATGTAATTTTACAACATACAAAGACTTTCTAGACAATAGTTTGGAGATGAACCACATATGGAACTGATGGTCAGGTGTTCACAAACGTTTGGCAATAAAGTGTAT

>scaffold_300000217-3

AAGCCAGGGGTCACCAAACTCCTGGAGAGCCGGTGTCCTGCAGAGCTTATCTCCAACCCTAATACCCCAAACACACCTGAACTAGCTAATCAAGGTCTTGCTAGGTATACTTGAAACTTACAGGCAGGTGTGTTGAGGCAAATTGGAGTTAAACTCTGCGGACATCGGCCCTCCAGGACCGAGTCTGGTGACCCCTGCCTT

>scaffold_300000218-2

TATAATATACACATCATATTGTTACAAGTTTGTGGACACCTCACCATCAGATCCATATGTGGTTCCTCTCCAAAATGTTGCTACAAATTTGGAAGCACACTGTTGTCTAGAAAGTCTTTGTATGTTGTATCATTACATTATTCCTTAACTGGAACTGCGAGACTCGAACCCTGTTCCAGCATGACAATGCCCCTGTGCACAAAGCCCCTGAGCTCCATGAAGACATGCTGTGTTCTGGTTGGAGTGAAGAACTCTCATGTCCTGCACAGAGCCCTGACTCTGACTCAACCCCACTGAACCCCTTTGGGATGAACTGGAACCCCGACTGCACCCCAGACCTCCTCCCCCAACATTAGTGTCTGATCTCATTAATACTATTGTAGCTGAATAAACACACATCTCACATTCAAGCTCTAAATTGTAGTGTATACCTTACTAGAACAGTGGAGCTTAATCGCACAAAAAAATGGGAGTAAATCTGGAATAAGATATTTAAAATCACATATGTTTGTGATGGTCAGGTGTCCACAAACTTTTGGCAATATAGTGTATACTATA

>scaffold_300000218-24

TAGGGCTGTGTGTTGGCAAGAGCCTCACAATACGATACATATCACGATACATGGGTCATGATATATTGCAATACTTAAAAAAAATTTGTTAAGTAAAACAAATAGCTAAAATTACAACTTGCTGTGGGATTCTAGTATGTGTATCACATTATATTGATTACTCAGCAGACAAATAAAAACTGAGGGTTTTAATTAAATATTAATGTAAATGTAAATCAGTTTAAAATGTAATTTAAATATGTATCGACACTAGAGTTTAGAATTTCGATACACTATCATGGAAAAAAAAAAATCACGATAGCTAGCTATATCGATATTTCTACACAGCCCTA

>scaffold_300000219-1

AATAAGGCTACGTTCACACTGCAGGCAAATGTTCCCTAAATCCGATTTTTTTTGCCTACATGTGACTCAGATCTGTTTTCTCATGACAGTGTGAACAGCACAAACCGCATGGAATCTGATCTTTTCAATTCCGATTTCCATATGTGCTATTAAATGCGACACAGGTCAGATGTTCTGCGATGCGACCGCAGTGGGAACAGTTATGTCGGAATTCATGCGACTTTTACGTCATTTTTCGATGGACATGCGTCATCATTCTGCACCGGCGCGTAACTACAAACAGTTGATATATAATCGATTTGGCTGAAGGCGTTGATCACGTGACGGTTCCACCACTCCTGACTCCGGCTCCACATCCACACACACCTCCATACTGAAGTAACATCCACTGCTCCACAACATGCTATTATTAAAACTTTAACCCTTTTTCTCCTCTTCCTCCTCAACGACTGCTCATTAATTCACTGTCGTCTGCTGCACATCACCTTGTAAATATAAGCAGCTAACGCGTAAACACCGCCTTCAGTGTTTATTTTGCCGTATGACAGGTCAGTTTTGCGCATGCGGGTCAGTTTAAGGATCGTGATCAGTTCAGACTGGAAATCTGATATCGGCCACATTTAAAACAACAATGTAAACAATTATACAAAAAAATCTGATCTGAGCACTAAACCTCGCAGTGTGAACGTAGCCTTATT

>scaffold_300000219-4

AAATTCAATTTTATTTGTTTAGCACTTTTAACAATAGATATCGTCTCAAAACAGCTTTACAGAAATATAAAAATTCAGATTGAAAAATGCAAATTTTAAAATTGAATTT

>scaffold_300000219-19

TAGGGGTGGGACCAAAAAATCGATTTGCCGATTTATCACGATATTTTTGGCCGTGAGACGTTATCGATACTCTTGCACCAAGTATCGATATTTTTTAAACATACAAAAGCTCTAAATTCATACCTGTCCTACACCGTGGCTGTTTACCACTTTGGCTTACTGGAGCGGCAGTACCCCCAGTGTCTTGTGAAGTGATAAAATAGATTTTATAAAAAAGATTTTTATAAAATAGATGTACTCTTAATAAATGAAATACATCATATGACTTTTTGATGCATTTTTTTTTATTTAGAGATATCGTGATGTATCGTAAAGGATTTTCTTGCAATATATCGTTTGTCACAGAATCGCTGGTGTTGTGATATATCGTTAGCGTGGGCAAAATATCGTGACAGTATCGTATCGTGAGTTAACCTGTAATTCCCACCCCTA

>scaffold_300000221-3

GCTACGTTCACACCGCAAAGTTTAGTGCTCAATTCCGATTTTTGCTCAGATCAGATTTTTTTGTATAATTGTTTACATTGCTGTTTTAAATGTGGCCTACATCAGATTTCCAGTGTGAACGGATCACGATCCTAAACTGATCCGCAGGCGCAAAAGAACTAATTCTACGTCTCACACACGTCCTGTCATACGGAAAAATAAACATGGATGACACTGAACTCGGCGTTTACACGTTAGCTGCTTATATTTGATGTGCAGCGGACGACAGTGAATTAATGAGCAGTCGTTGAGGAGGAAGAGGAGAAAAAGGGTTAAAGTTTTAATAATAGCATGTTGTGGAGCAGTGGCTGTTACTTCAGTATGGAGGTGTGTGTGGATGTGGAGCCGGAGTCAGGAGTGGTGGAACCGTCACGTGATCAACGCCTTCAGCCAAATCGATTATATATCAACTGTTTGTAGTTACGCGCCGGTGCAGAATGATGACGCATGTCCATCGAAAAATGACGTAAAAGTCGCATGAATTCCGACATAACTGTTCCCACTGCGGTCGCATCGCAGAACATCTGACCTGTGTCGCATTTAATAGCGCATATGGAAATCGGAATTAAAAAGATCACATTCCATGCGGTTTGTGTTGTTCACACTGTCATGAGAAAACAGATCTGAGCCACATGTAGGCAAAACAATTTGGATTTGGGCCGCATTTGCCTGCAGTGTGAACGTAGC

>scaffold_300000223-3

ATACAGTGGAACCTCAGCATACAAATGTCCTCCATTAATAATTTTTGCCTTAAGAATTGTAATTTTTCAAGAATTTTGCATCGGCAAATGAAGTATTTTCGGCATACAAACGAACGAACTGAACAGAATGCCGAGTTAAATTGCGCATACGTTTGGGAGCTGTTCAAAACTTGTTTGCGTCGGTGGAAAGCCAAGAAAAGCCAACCGAAGTGAGTTTACAAATTTTACAAAAAATATTTTCAGTCAGCAAAAGCTGTTTTAAAAGACTCAACCCACGATACCGACGTCGCCTTCGGCATGCGTTCAAAAGCTACGTGATTTTATGTGCGTTTTTTTGTTGTTGACAGATTGGTCTATTTATTTCATATTTTACTTCATTTTCATGTCTTTTCTAAATGTTTTGATTGTTTTTACATGCAAAAATATGATTATTGTGTCTGAATATGGTAATATTGGCCATATTTTTGAGTGGCTGGCACAGATTATCTGCATTTACATTATTTCTTATAGGTAGTACAAATTTTGGCCTTAAGAACTCTCCTTAAGAACGAATTAAATACGTATGCCGAGGTTCCACTGTAT

>scaffold_300000223-18

TTACACTGTGTGATGTTTAATAGTCTTTAGCGATGGTTGTGTGTCAGACTGTACGAACACGATCCCCGTGTCACGCTGTAGGATTTCAGTTATCATAATGTCAGACTGTACGACAGTCAAGACACGTCAAAAACGGACGCACACAAGAAGGCTCGTCTATAGTTTTACGTCATCAATCCGTAATCTAATCTCTCGCTAAATGGTAGCTAGCAGCAAAGAAAAACAATCTGTAGCGTTAATTTTGCTCATGACATGCCTCAATACGTTTCTCTGCTTGCTGTTCTGTCCACCGGATTTGTTTCCACATTTTCCCGAGGTGGTCTGTAGTTGTCGTGCTAATTGCGTCATCAGGTTGTTGGTTTGACGGTCGTCGTAGGAAAAGTTACACTGCAGGTACGTGTCTGAAATCTTCTGAAACTGCCAGAATTTCATTGGAGGAAAAAAATGATTGCAACGGTCATTAATCGTCTGTCAGTAAACACGTCAAACTAGCCATCAAACACAACAGATTTTAGAGTAGGATTATAGGAATCTTTTAGGATTTAAAAATTTGTCTCAGATGAACAAATCGTGGCCAAAATCGCACAGTGTAA

>scaffold_300000224-4

TAATGCCGTGTTCACACTGTACGATTCTCAAAGCCGTCGGATCACCGTTGTTTTCACACTGCACGACTATCTGGTGTAACATTCGGTAGCTGTTGTGTTCACATGGACGATGGATCGGTGACAGGATGTTACACACCACATGACTTTACAGTAGGAAGAATCGCCATCGACTCTGTCTGATCCACAAACTACGTTTCACAACCAAACGTACACGAGAAGCGCTGAGGAAATAACGCATAAAGATCACGAGTGAGATCAGAGTTCTCACATGAGACTGGAAATGTTATTAAAATGGTAGCCGGCAAGAAGTTCGCCATACAAACGGTCTGTGCACTGATTTGCAGTGAAGGAGTGAAAAATAAAAACAGATAAAAAAATCAAATAAAAAATTATGGTCTGTAACTCCTCCCCGAACTTCTCGCTGCTCTGTATCTTACTCTCTCATTGGCTGTAAGTCATCGCCGAGGTATTTTTCACTCTGAACCCCCGACAGGTCCAGATATTTAGCATGCCAAATATTTCAGTCCTCAGCGATTCTCTCAGGTCGCGTCTTTGATAATTTGACGAGCACCGATTTGCCTCCGATTTCAAGCATTTGTCTGCGATTTCGCAAAACCTGTCGGCGTGTGAAGATCGGGGTTAAAACCGTGCAGTGTGAACTCGGCATTA

>scaffold_300000224-7

TTAGGGCTGCACGATACTGGGAAAATATGCAATATTGTTGTTGAGTATTGCGATAACGATATTTCTTGCAATTTCCCTAGAGAAATGCTATTTTTATTAGCTATTTTAGCTGCAGGTGTTTTTCAGGCTGGTTCATCTGATCATGATTATCTCGCTATAAAAATTTTGCAGTTTGTTGATCGTCTTGCGTCAAATCACGCGCACGCAAATCCCAACTAACATCCGCGTACCAGATATTTTACTATCCTAAAACTTTAACCACACGTTAATATTTGAAGTATTAAATCTCTCAATTATCGCAAACCATCTCGATCTGCATATTTGCGATATTTTGATAAGTTCGAATATATTGTGCAGCCCTAA

>scaffold_300000226-2

TGGCTCCGTCCGAAATCGCATACTTCCATACTGTATAGTATGAAACTGTATAAACTGAGTATGTGAGGCGAATAGTATGTTTGAATCCATAGTATGCGAAATACAGTACGTGAAAAGTTCCCGGATGACCTACATGTACTATTCCCGCCCAGATTTTGAAGTATGCATACGATGTACTTGTACACGTTACTATCCCATGAGGATGTGGCGGAAAGCGGCGACGCAACACTTTTCACGTTTTACTTAAAGAAAAAATCATTGTTCCTTGTTGTTCAATTACACTCGTTTAACATCATTCAAGTTAAACATTATCCAATTTGAAATTCACATTGCTGTGACGTCGTATGTGATGCGGATTTAGTACATCCGAATTTCATTTATACTACCCGTAATCATACTATACAGAACATACTTTTTTTTATGGTCGAGAAGTACGTACTAACTCAAATGTAGTACATACTTAAGTAGTATGCGATTTCGGAAGGAGCCA

>scaffold_300000229-9

TTAAAGGGGTTATGAACTACCTCAGTTTTTCATTTTGTACTGTTCTCTGAGGTCCACTTATAATGTTATCAAGAGTTTTACATCAAAAAACATAATCGTTTAGAAGTAATAGGTTATTTTCTGTCCTGTTTTTAACCCCATCATCAGAACGCTCTGTTTGAATAGGCGTGGCAGATTGTTGTCTCAGAAGTAAACGCCCACTTCTATTATTGGCTAACAGTTTATGCCACTCTTGTCATTTACCTATCACGTGCATGCGTGAATCGGTGGGCGGGGCTAAACAGACAGTGATGTCGATTTAAGCGTCAATCTTCTTCTGCGGAGGCGGTGCTTATCCACACTATTACATCATCGAGTAGAACATTCCAAAACCGTCGTTTTGGCCGACTGCCTTCAATATAAACTGTTTTTAGAGAAACGACAATGTTTTGAGTTCTGAAACTTACAGCATGTTTTTATAGCACAGTGACGTTGTATATGTCAAAAGATCAAAGGAATTTAGATTTCTTAGTTCATGACCCCTTTAA

>scaffold_300000229-17

TTATTAAGACTTTGGGTTGTTGCTTAGAGGATTGAAGTTATAGCCCCACCGCCGCCCAGCTGCCACTTTTGGACCCTTGAGCAAGGGTCTTAACCCTCTCCCTGCTCCTACGGTGCTGTATCATGGCTGACCATTCTGAACCAATCTTCCTAAGATTATGAGGTATGCAAAGGAGATGATTTTGCTGTTCAGTAATGTACAGGTGACAAATAAAGTCTTAATAA

>scaffold_300000229-31

ACACTGGAACCACGGCATACGAACTGAATTTGTTCTCTAGGCGAGTTCTTATGGTGAAAATTAATATTTGCGATACAAATGTTCCCATAAGAAATAATGTAAATGCTAATGATAATTCGTTCCAGCCACCCAAAAATATTCACAATATTACAAATTTCCAACACTATAATCATATTATTGCATATAAATCAATCAAAACATTTAGAAAAGATGTGTAAATGAAGTAAAATATGAAATAAACCCCAGACCCACGCCACCAATCTTTCAACAAAAAACTCACATAGTTTTTGAACGCATGTTGAAGGCGACATCGGTATCGTGGGTCGACTCTTTAGAAACAGCTTTCGCTGATTGACATTTTCTTTTTTTAATACGTAAACTTGCTTAGCTTGGCTTTCCCCTGACGCAAACAAGTTTTAAACCGCTCCCGGACGTACGTGCAACTTATCCGGCGTTCGGTTCGGTTCGTTCATATGCCGAAACATGCTTCGTATTTTGATGCAAATTCTTGCAAAATTTCAATCCTTAAGGCGAAAATTCTAATGGAGGACATTCGTATGCTGAGATTCCACTGT

>scaffold_300000230-1

TTATGCTGTGTTCACACCAAACGCTAAGCGAATATGCGCATCGCGTCACTCGCTCCAGATTACTCGCGGAATGTTTCTCGTGTCACTCACGCAACGTTTTTATTCGTAAAACATAAATTTTGCGGTGCCCGCAGACGGCTACAAAAATGCTCTCCTCCATTGTCTAATACAATGGACGTGTAGCTCTTTGCTGATTGGATGGTACAACACCGTGTCAGTGAATTTTCCGCTCAAGCTGAAAGTTTTTTAACTAGCGTGGAGATGCGTTTGATGCGAATATTGCATGTTTGCGGCAAACGCTCTCCGCAGTTCGCCTTTAATCGCGTCTTTGCATTGACTGTGTATGTAATCTACTCGCGCGAATAATTAAATTCGTGTTCGGTGTGAACACAGCATAA

>scaffold_300000237-3

TACAGCCAACCCTCGACTTACGATGGTTTTACGTTTTGGAAAACCCATCGGAAGTGGAAATACCGTTAGTGGATATAAGTATACCCCTTTATGATGCGTTCTGAAGTAGCCTGCGGTAGCTAATAAGTGTTCTGTATGCAAGGTTATAGAGTATGTAATGTTACTTTAAAACAAAAAGCATATCAATATAGCAAAATATGCCATGGAAAAGTTGCAACAAAGGTCATTTAATGAAACAATGTTAACACTAAGCAACCAAGTGTGCGCTTGAGTGAACAGCAACCACCAGGTAGCAGTAGATGCGTGTTCCAATAATATCATGCTGTTGTCACGTTTTCTTTAGCCGCACAAAATTTTACAAATATTTTTCAACATATTTTTTGGGTCACGCAGTTGTATCGTAACTCGTAAGTCGACATATCCTATGTCGAGGGTTGTCTGTA

>scaffold_300000237-4

TACAGACAACCCTCGACATAGGATATGTCGACTTACGAGTTACGATACAACTGCGTGACCCAAAAAATATGTTGAAAAATATTTGTAAAATTTTGTGCGGCTAAAGAAAACGTGACAACAGCATGATATTATTGGAACACGCATCTACTGCTACCTGGTGGTTGCTGTTCACTCAAGCGCACACTTGGTTGCTTAGTGTTAACATTGTTTCATTAAATGACCTTTGTTGCAACTTTTCCATGGCATATTTTGCTATATTGATATGCTTTTTGTTTTAAAGTAACATTACATACTCTATAACCTTGCATACAGAACACTTATTAGCTACCGCAGGCTACTTCAGAACGCATCATAAAGGGGTATACTTATATCCACTAACGGTATTTCCACTTCCGATGGGTTTTCCAAAACGTAAAACCATCGTAAGTCGAGGGTTGGCTGTA

>scaffold_3100003-8

TAGGGCTGTGTAAAAATATTGATGCAGCTAACTATCGCGATATTTTTTCGCGATACTGTATCAATATTCTAACCTCTAGTATCGATACATATTTAAATGACGTTTTAAACTGATTTACGTTTATATTTAATTCAAACCCTCAGTTTTTATTTGTTTGCTGAGTAATCAATATAACGTGATACACATACTAGAAGTTGTATTTTTGGCTATTTGTTTCACTTTTTTTTTTAAGTATTGCAATATATCGTGATATATTGTATCGTGACCCATGTATCGTGATATGTATCGTATCGTGAGGCCCTTGCCAATACACAGCCCTA

>scaffold_3100009-2

TAGGGCTGCACAATAAATCGAAATTATCGAAATATCGCAAATGTGCATATGGCAACGATTTGCGATAAATGATCGATTTAATACTTCAAATAGTAACGTGTGGTTAAAGTTTTAGGATAGTAAAATATCCGGTACGCGGATGTTAGTTGGGATTTGCGTGCGCGTGTTTTGACGCAAGACGATCGACAAACTGCGACGCTTTCATAGCGAGATAATCTTGATAAGACGAACCAGCCTGAAAAACACCTGCAGCTAAAATAGCTAATAAAAATAGCATTTCTCTAGGGAAACGTTACATCGGAAGAAATATCGTTATCGCAATACTCAACAACAATATCGCATATTTTCCCAGTATCGTGCAGCCCTA

>scaffold_31000011-9

TAGGGCTGCACAATATATTCAAATAATCGAAATATCGCAAACGTGCATATAGAGATATGAATATCGCAACGGTTTGCGACAAATGATCGATTTAACACTTCAAATAGTAACGTGTGGTTAAAGTTTTAGGATAGTAAAATATCTGGCGCGCGGATGTTAGTTGGGATTTGCGTGCGCGTGATTTGACGCAAGACGATCAACAAACCGCGACGCTTTCATAGCGAGTTAATCTTGATAAGACGAACCAGCTTGAAAAACACCCGCAGCTAAAATAGCTAATAAAAATAGCATTTCTCTAGGGAAACGTTATATCGCAAGAAATATCGTTATCGCAATACTCAACAATATCGCATATCGCGTATTTTCCCAGTATCGTGCAGCCCTA

>scaffold_31000011-11

TAGGGCTGCACGATACTGGGAAAATACGCGATATGCGATATTGTTGAGTATTGCGATAACGATATTTCTTGCGATATAACGTTTCCCTAGAGAAATGCTATTTTTATTAGCTATTTTAGCTGCGGGTGTTTTTCAAGCTGGTTCGTCTTATCAAGATTAACTCGCTATGAAAGCGTCGCGGTTTGTTGATCGTCTTGCGTCAAATCACGCGCACGCAAATCCCAACTAACATCCGCGCGCCAGATATTTTACTATCCTAAAACTTTAACCACACGTTACTATTTGAAGTGTTAAATCGATCATTTGTCGCAAACCGTTGCGATATTCATATCTCTATATGCACGTTTGCGATATTTCGATTATTTGAATATATTGTGCAGCCCTA

>scaffold_31000017-1

TACAAGGGGGTATCAAAAAGTTTGGCGACACACCAACAGATGGCAGTGTGCAGCCTTGTGCGGCCATCTGCTGGCGTGTCTCAAAACTTTTTGATACCCCCTTGTA

>scaffold_31000017-10

TAGGGCTGTGTAAAAATATCGATACAGCTAGGTATCGCGAGTCTCGTGTATGCTGAGTTTCACGATAGTGTATCGATATTCCAACCTCTAGTATCAATACATATTTAAATTACGGTTTAAACTGATTTACGTTAATATTTAATTAAAACCCTCTGTTTTTATTTGTCTGCTGAGTCATCAATATAATGTGATACACATACTAGAATACCCCCGGTGGTACACAGCAAGTTGTATATATTTTTTTGAAGTATTGCAATATCTATTGTATTGCAATATACCGTGATATGTTGTATCGTGACCCATGTATCGTATCTTAAGGCCCTTGCAAATACACAGCCCTA

>scaffold_31000019-12

GTAGGGATGCAACGATACTGGTATCGGGCCCGATACTGAGCTCATGTACTCGTAAAAACACTCCGATACCAAAGACCGATACCTCTTGTGACGTCATTGACAAAACTTTCAGTGCACAACGATTCCGGCGGCAGCGACAGAATGTCAGACTCAGCGGTGTGGAAATATTTTTCAAAATTAATAATGACAACCCACGCACGGCAGACTGCAAAGTTTGTTTTGCAAAAATATCAAGAGGTACAAAAATGAGTACTTATAATACGAGTAATCTGATAAAACATCTGAAATTAAAACACAAAAAAGTGAGCACAGACAATTTGCCGTTAGCACTCGGCAACCGACCCTGCAGCAAACCCTTGCAAGACGAGAGAAAATGGCAAGAGAAAACCCGAGAGCCATACAAATAATCCCCCATCCCAAAAAAGTGCAGGTATCGGTATCGGCGAGTACCAGGAATATCGGTACTCGTACTCGGTCTTTAAAAAACGGTATCGGGGCATCCCTAC

>scaffold_31000022-3

AGGCCGCATTTACACTGCAGGTCTTGATGCCCAATTCCGATTTCGTGAATATATCCGATTTTTTTTCACGACTCGCGTACATCTAGTTATAAAACTGACCCGTATCCGATGTCTGCATTTACACTATACACGGCAAAACAAGCCAAGATAAACGTACTGACCGGAAAAGGAAGTAAAAACTGTGTGATCATCAATGCACGCGATGGACGAAGACGAAATGATCATTCAGTATTTTGCTTTCCGCACCATTTTTTCGGTCGGCTTAGTTCTTGGAACCCGGGGTCAGGAACAGTGGGTCAGCAATTTTCGTATGAGACGATAGACATTTGATACGTTGTCAAGAGGTCGCTGTTGGTGTCCACCTGAGTTGACGTCATGCGCCACCGTCGCTTATTCAATAACGTATGACTCGCATTGACGAGTGAAAATCCGATCTGTTTGCTTACATGGCAGACGCTATTGCACGTATCCGATTCATATCGTATTTATTTCCACATATGAATGAGGCTTGAAACCCATCTGAAAATAACGCAATCCATGTGTTTTTTTTTCCCTACTTACACGATCGTGGGTCACATCCCATCTGAGCCACATTGGAGGAAAAAATCGGAATTGGGTCACTTGATACGTGCAGCGTAAATGTGGCCT

>scaffold_31000022-5

ATTAGGGCTGCACAATATATCGAAATTATCGAAATATTGCAAATGTGTTAAATGAGCGATTTAATACTTCAAATAGTAACGTGTGGTCAAAGTTGTAGGATAGTAAAATATCTGGCACGCGGATGTTAGTTGGGATTTGCGTGCGCGTGCTTGGACGCAAGACAATCAACCAGCCGCAAAGCTTTCAAAGCGAGCTAATCTTGATAAGATGAACCAGCCCGAAAAACACCCGCAACTAAAATAGCTAATAAAAATAGCATTTCGCTAGGGCAAAAATAGCATTTGCTCGTTATATCGCAAGAAATATCTTTATCGCAATACTCAACAACAATATTGCCTATTTTCCCAGTATCGTGCAGCCCTAAT

>scaffold_31000023-8

TGGCTGCGTCCGAAATTGCAAACTTCCATACTATACAGTGTGCGAAACTGAGCATGCGAGGCGATTAATTAGTATGTCCGAATCCTTAGTATGCGTAATACAGTACGCGAAAAGTTCCCGAATGTCCTACTACTCCCGGCCGGATTTTGAAGTATGCATACCATGTACACTTTACTATCCCATGAGGCCGCGGGAGACACGAGTCATCATATGCGGAAGTGGCGGAAAGCGGCGACGCGGCACTTTTCAAGTGCTTTAAGTACTTAAAGACAAAACATTGTTCCTTGTAGTTAAATTGCACTCGTTTAACATCATTCAAGTTATCCAACTTTAAATTCGCACTGCTGTGACGTCGTATGTCACGTGACATTATCAACATGTAGTGCATCCAAATACTTTTTTAACGGTCGGGAAGTACGTACTTACTTAAATGTAGTACATACTTAAGTAGTATGCGATTTCGGACAGAGCCA

>scaffold_31000024-2

ATAATAATAATAATAAAACAATCACAACAGCATTATTACTATTAATTATATTGTTATTATTATTATTGTTAATGTTATAATAATAATAATAATAAAACAAAATCACAACAGCATTATTATTATTATTATTATTATTATTATTATTATTAT

>scaffold_31000024-7

TAGGGCTGCACAATATATCGAAATTATCGAAATATCGCAAATGTGCATATCATGATATACATAATAGCAATGCTTTGCGATAAATGAGCGATTTAATACTTCAAATAATAATGTGTGGTCAAAGTTGTAGGATAGTAAAATATCTGGCACGCGGACGTTAGTTGGGATTTGCGTGCGCGTGACGCAAGACAATCAACAATTTGCGAAGCTTTCATAGCGAGCTAATCTTGATAAGATGAGCCAGCCCGGAAAACACCCGCAGCTAAAATAGCTAATAAAAATAGCATTTCTCTAGGGAAAGGTTATATCGCAAGAAATATCGTTATCGCAATGGACAACAACAATATCGCATATCGCATGTTTTCCCAGTATCGTGCAGCCCTA

>scaffold_31000025-2

CAGGGGTGGGCAACCCATTCTCCTTAAGTTCTTTTGTCCTGCTTGGTTTCCAACTATCCCTGTCCTACCTACTGCTGATTCCCTGGAACAGGTGTGTTTAGTCAATCAGAAGCTGGAAGAGACCATCTTGGATGAGGGTGCAGTGGGAGGATACCAAGTGATTTTGTATCTCCTAGCGTCTGATTGATTGACTAAACACAGGGAATTTTCAGTGAGTAGCACAGGGGTAGTTGTAAAACAAGCAGAACAAAGGAACTTGAGAAACATGAATTGCCCACCTCTG

>scaffold_31000025-9

TACAGAGTGAGTAAAAAGTAACTAGGCATTAAAAATTGGTAATAAAGCACATATTTCTAAAACAAATTTATTGAAACAAATTGTACATGTACTTTATTACATAGGATAATGAAAGCAAATCTTTACATTTCAATGTAGGCACCAGTAGCGTCAACCGGTTTCCTTAAACGGCCGGAGATGGAATGCACAGTCTTCTTAAGGAAGTCGTTTGGAATATTGCTAAGAACCTCCTGAATCCGTGCCTCCAAGTCGTCCAGAGTGCGAGCCTTGAAATCCTATTTACTATCGTCTATTTACGGACAGTTTTCATCAGGACTCCATTGATACTACAAGTTCTAGAGCCATTAAATATTGCCTAGTTCATTTTCACCCACCCTGTA

>scaffold_31000025-26

ACTAGGGCTGCACAATATATCTGCATGCATATATATGCATATGGCAATGCTTTGCGATAAACGAGCGATTTAATATTTCAAATATTAATGTGTGGTCAAAGTTTTAGGATAGTAAAATATCTGGCACGCGGATGTTAGTTGGGATTTGCTTGCGCGTGCTTTGACGCAAGACAATCAACAAGCTGATTGGGACTAATCTTGATAAGATGAACCAGCCTGAAAAACACCCGCAGCTAAAATAGCTAATAAAAATATCATTTCTCTAGGGAAAGGTTATATCGCAAGAAATATCGTTATTGCAATACTCAACAACAATATTGAATATTTTCCCGGTATCGTTCAGCCCTAGT

>scaffold_31000031-1

CAGGGGTCACCAGACTCGGTCCTGGAGGGCCAATGTCCAGCAGAGTTTAGCTCCAACTTGCCTCAACACACCTGCCTGTAAGTTTCTAGCAAGACCTCGATTAACTAGTTCAGGTGTGTTTGATTAGGGTTGGAGATAAACTCTGCAGGACACCGACCCTCCAGGACCAAGTTTGAGGAACCCTG

>scaffold_31000034-10

ATATACTATATGGCCAAAGGTTTGTGGACACCTTACCAGCACACCTACATACTATAAGTTGGAAGTACACAGTTGTCTAGAACGTCTTTATATTTTGTAACATTACATTATTCCTTCACTAGAACTAAGAGATCCAAGCACTGTTCCAGCATGACAATGCCCCTGTGCACAAAGCCTCTGGGCTCCATGAAGACATGCTGTGTTCTGGTTGGTGTGGAAGAACTCGAGTGTTCTGCACAGAGCCCTGACTCTGGCTCAACCCCACTGAACACCTTTGGGATGAACTGGCACACAGACTGACTGAACCCCAGACCTCCTCACCCAACACTAGTGCCTGATCTCACTAACACTATTGTGACTGAATGAACAAATCCACATAGCCACGCTCTACAATCTAGTGGAAATCCTTTCCAGAAGTGTGAAGGTTATTATAGGAGTAAGAGTGAGAATAAATCTAGGAGTAGGATGTTTGAAAAACGCAAATGGATGTAATGGTCAGGTGTCCTAATATTTTTGTCTATATAGTGTAT

>scaffold_31000035-22

CTAGGGCTGTGCGAAAATATCGATACAGCTAACTATCATGATATTTTTTTCAATAGTGTTTCGATACTCTAACGTCTAGTATCGATACATATTTAAATGACGTTTTTAAACTGGTTTACGTTAATATTGAATTAAAACCTTCAGTGTTTATTTGTCTGCTGAGTAATCAATATCATGTAATACACATACTAGAGTCACCCAGGTGATACGCAGCAAGATGTATTTTTGCTGTGTGCTTAAATTTTTTTTTTTTTTAAGAATTGCAATATGTATTGTATCGTGAGGCCCTTGCCAATACACAGCCCTAG

>scaffold_31000036-5

TAGGGCTGTGTATTGGCAAGGGCCTCACGATACAATACATACTGTAACATGATACATGGGTCACGATACAATATAGCATGACATATTTCAATACTTAAAAAAAAAAGTAAAACAAATAGCTAAAAATACATCTTGCTGTGTGCCACCTGGGTGACTCTAGTATGTGTATCACATGATATTGATTACTCAGCCGACAAATAAACACTGAGGGTTTTAATGAAATATTAACGTAAACGTAAATCAGTTTAATATGTCATTTAAATGTGCATCGATACTAGAGGTTAGAATATTGCGATAAATATTGCGATAGTTAGCTGTATTGATATTTTTACACAGCCCTA

>scaffold_31000037-1

TAATGTACTATTTGCACAGGATTGATATTATCTGGGGACCTCGTGTGATTTAGAAATTACCCCCCACGTGTGAATTTCATGTGCACGGGATAAGCGAAGCCTGTGATTTTACTTGAATTTACTGACTTATCTCCTGGAAATATCTTGTCATTATATAGGTATAGATGTATGATATATCATAAACAGAAGAATGATCCTTACCGCATGCCGCTATAAATGTCATTTATCTCCATCTAATCATTCTTTTAGTTTTCGCTCTTCTGGTGGCGTTCTGTTTGATCTTTCTGAAAGTTTTATGGTTGTTTGGCGTATAGCGATATGCAGCACCCAAAATACACCCCAAACACTCCAACACAGCCTCATTCTTCACACACAAGAAAACTGAAACCTTAAAAAATGATACACGACCCCGCAAATCTTGGCCAAATCACAGAGATGTCGATTCGCACAGGACTAATATTATCACAGGACGTCTGTGTTCGACAAAATATGGTAGGTCATTTGCAGGGGGAATATTTACTTTACAAATCACAGACATGGTTGATTCGCACGGGATTAAGATCACAGACAACCTCCACAATTATTACAAATAACCAGAGGTCCCCAGATAATACTTATCCCGTGCAAATAGTACTTA

>scaffold_31000038-4

TTAGGGTTGCAAAGAGGCGGGGAAATGTTTGGTAAATTTCCAGAAACTTTTCATGTTTACTTCATTTTTGGAATTTTGGAAAAGAAATTCCAAGTTGGACACTTAACAGGAATTTATAGTAACTTACAGGAATTTAAGGGAAATTTATTGAATTATTTTAAAATCTGGGGAAACTGTTTATACAAACAAATATAAACATTTTGTTTGGTCATAAGCTGACATGCAAGCAAACTAATCCCCACTTTAAGAATGACGTTATCGGTGTGTGTTCTGGAGGAACGCACAGTGCATGCGGAGGTGTGGCCTCGATAGTCCTGCAGTGGTGTGTGCATGGGATTAAGGAATATGCAGGGTAGAAATTCAACTGAACTTGCATTAAATCTGGTTGTTTTAACCAAGATCTTATGCTGATTTTTTTTCAACTACATTTAAGCTCCCTGTTATAGGCTAACCTGCAATTTTGCAAATTCCCAGTTTATTCCCATTATATTCCCATGGAAAGTTTCCAGCCTTGAAAATTCCCAGAATTTTGCAACCCTAA

>scaffold_31000039-2

TATTAGGGCTGCACAATATATAGAAATTATCGAAATATCGCGAATATGCATATCGCAATGGTTTGCGATAAATGAGTGATTTAATACTTTAAATAGTAACGTGTGGTCAAAGTTTTAGGATAGTAAAATATCTGGCACGCGGATGTTAGTTGGGATTTGCGTGCGCCTGCTTTGACTCGTGACCATGAACAAGCTGCGAAGCTTTCATAGCGATCTAATCTTGATAAGATGAACCAGCCCGAAAAACACCCGCAGCTAAAGTAGCTAATAAAAATAGCATTTCTCTAGGGAAATGTTATATTGCAAGAAATATCGTTATCGCAATACTCGACAACAATATTGCATATTTCCCCAGTATCGTGCAGCCCTAATA

>scaffold_31000039-3

TAGGGCTGCACAATATATTGGAATATATCGAAATATCGCAAATGTGCATCTTGAGATATGCATATCGCAATGGTTTGCGATAAATGAGTGATTTAATACTTTAAATAGTAACGTGTGGTCAAAGTTTTAGGATAGTAAAATATCTGGCACGCGGATGTTAGATGGGATTTGCGTGCGCCTGCTTTGACTCGTGACCATGAACAAGCTGCGAAGCTTTCATAGCGATCTAATCTTGATAAGATGAACCAGCCCGAAAAACACCCGCAGATAAAGTAGCTAATAAAAATAGCATTTCTCTAGGGAAATGTTATATTGCAAGAAATATCGTTATCGCAATACTCGACAACAATATTGCATATTTCCCCAGTATCGTGCAGCCCTA

>scaffold_31000043-1

TACACTATATTGCCAAAGAGTTTGTGGACACCTCGCCATCAGGTCCATATGTGGTTCATCTCCAAAATGCTGGAAGCACACAGTTGTCTAGAATGTCTTTGTATGTTGTAGCATTACGTTATTCCTTCACTAGAACTAAGAGACCCAAACACTGTTCCAGCATGACAATGCTCCTGTGCACAAAACCCCTGAGCTCCATTAACACATGCTGCGTTATGATTGGAGTGGAAAAACTTGAGTGTTCTGCATAAATCCCTGACCTTAACACACCACTGAACACCTTTGGGATGAACTGGAACGCAGACTGCACCACAGACCTCCTTACCTGATGTTAGTGTCTGATCTCACTAATACTATTGTAGCTGAATGAACACATCTCTCACATTCAAGCTCCAAATTGTAGTGGAAAGCCTTACTAGAAGAGTGGAGCTTAATCCAACAGCAAAATTGGAAGTAAATCTGGATTAAGATATTTAACATCACACATGTTTGTGATGGTCATGTGTCCACAAACGTTTGGCAATATAGTGTA

>scaffold_31000043-6

TATCCCCCTGAGACCCAAGGAAAAAAGTGTCATCAATGTATTTTTTGTGTGTGTGTGATTTCTTTTCGAGTTAAAAAAACATTTTACAATTTACAACCCTTCAATTTAGAGTTTTAGAATTTTACTGTTATTTTAAAATTTTCAGTATGTTAACTGTCGTGGACCACAGGAATATTAAAGTTTGAAAAGGTGGTCCTTTTCATAAAAAGTGCTTGTTCTTATGGCAGCCATTTTGGATGCAGTGTAAGAACTAGTTCCGAGCATCCCAGCCAATCAAATGACATATCACTAGAAAGTCTTGATAGATTAGCTCAAAAATTTCCAAAAACATTTATGAAAACAAAATGTCCAATACAGAGGACACAAGTCAATGGGCCGGGTCTCAGGAGGATA

>scaffold_31000043-10

CATTAGCTAAGTTTCTATCCAAAGATGTGAATTAAACGCAAAACTGGAATATCGCATAAAACATTTGCAAATAAAGCACCGTTTCCTTCCAATGAGAACAAAATGGTCACTTCCTGATAAACTGCTGGCAAATATCAAAAAGAATAATGGAAGTTGCTGCGGTAGGAGAAGCCACTGTGGCCCTTTTTTGGTATATAATAAATTACTTGCGTCTCAGAACATGTAGACGAAACGCAGTCATGTCATCTTACTTTCGGGGGCAGGAAACGCAGCTGGCCAAGACAGTTCTGGGAGGTAATAATACCAAACCACTTTAATGACCGACTTTGGCATTTTAGAATGACCAAAACAACATTTCAGATGTTGTGTGATGAGATCGGTTTGCTTGTTAGTCCATAAATGGCGTCCCATCGCGCCCCAGTTCCGACAGAAAAACGCATCGCCATTGCCCTGGTTAAATTTGGCCATTTTTATCAATCACATGATCTGTTGAAGCTAAATCACAAGACTTTTTTGCTGCGCATGCTGGAATTGATTCGGTAAATGCGTTTCCATCATAGTTTATGCACATTCTTATCGGTTAAAAAGTTGATCCTACTCAGTTGTGCGCATAATTTAATGCGCATTTTCCGAATTCATGCACATATTGGCGTTTCCATCCAGCGTTTTTTATGCGATAGTCCAAAATGCGCATACAAATAGGTGGATGGAAACATGGCTAATG

>scaffold_31000048-2

GGCTACATTCACACTGCAAGGCTCGGTGCTCATTTCCAATTTTTGGTCAGATGAAATTTTTTTGTGTAGCTGTTTACATTGTTGTTTCAAATGTAGCCAATATCAGATTACCAGTGTGAACTGATCATGTTGCTAAACTGACCCGCATGCGCAAAAAAACTAATTATACGTCTCACGCAGCATCCTGTCATATGGAGAGAAAAAAACACGGAAGACACTGAAGGCAGAATTTACGGGTTAGGTGCTTACATTTATAAGGTGATGTGCAGCGGACGACAGCTAATTAATGAGCAGTCGTTGAGGAGGAAGAGGAGAAAAAAGGCCACATTTTTAATAATAGCATGTTGTGGAGCAGTGGCTGCTACTTCAATATGGAGGTGTGTGTGGATGTAAGCCGGAGCCAGGAGTGGTGGGGCCGAGACATGAACGCTTTCAGCGAAATCGATATCAACTGTTTGTATTACGTGCCGGTGAAGAATAATGACTTATGTCGATCGAAAATGACGTAAAAGTTGCATGAATTTCGACATAACTGTTCACACTGCGGTCGCATTGCAAAACATCTGACCTTTGTCGGATTTAATACTTGTGGCACAAATCGGAATAGAAAAGATCAGATTCCATGCAGTTTGTGCTGTTCACACTGTCATGAGAAAAACAGATCTGAGTCACATGTTGGCCAAAAAATCGGATTTGGGCCACATTTACCTGCAGTGTGAATGTAGCC

>scaffold_31000049-4

TTACACTGTGCGATTTGGTCCACGATTTTGTCGTCTGAGACAAATTTTGAAATCCTAAAAGTTTCCTAAAATCCTACGCTAAAATCTGTAGTCTTTGATCGCTAGTTTGACATGTTCACCGACAGAGGATTAATGGTGGTTGCGATCAGTTTTTTCCTCTGATGAAATTCTGGCAGTGTCAGACGTTTTCAGACACTTACGTGCAGTGTGACTTCTCCTACGACGACCGTCAATACAAGAATAGACGCAATTATAACCTGATGACGCAATTAGCATGACAACTACAAACCACCTCGGGGAAATGTCAAAACTAGTCTGGTGGACAGTACAGCAAGAAGAGAAAGTTATTGAGTTATGTCATGAGCAATCTAAATGCTACAGATAACGCTGTTTTTGTTTGCTGCTAGCTATTATTTAGCGAGAGAATGCAGGTTACTGAACGTGTCACGGAGTGATGATGTAAAACTCCAGACGAGTCTTCTTGTGTGCGTCCGTTTTTGACGCGCCTTGCCTGTCGGACAGTCTGACATTATGACAACTGAGATCCTACAGTGTGACATGGGGATCACGTTCGTACAGTCTGACAAGCAACAATCGCAAAAGACTATTAAAAATCACACAGTGTAA

>scaffold_31000049-5

TGTAACCCTCAGGTGGTGTTTGTATTTTTGGTACCTAGCCAATGTTTACGGATCTGGTGGACCCACCGCATTATTGGGTTTTTAATCAACACAGCCATACCAATTTATGGAAAAATACTTAAAGGATGTTTACTTCATTAACAAGCAATATAGACATCATTTATGGTTAATATTAGCCATTTACCCTGTTAGATCAAATTGGAAAATATAAGTGTTATGGAAAATATGTTGTATTTTCCATTGTTCTACATGGAAAATGAGCCAAGGCCAATGAGTCTGGGGCTGAAAAAATTAGTAGCAACATTTCTCTTTAAACAAACTCAGGTCTCACATTCGCAAACACCACACAAGGGTTACA

>scaffold_31000052-7

TAGGGCTGCACAATATATAGAAATTATCGAAACATCGCAAATGTGCATATCGAGATATAAATATCGCAGTGGTTTGCAATAAAAAATTTTTTTAATACTTCAAATAATAAATGTGTGGTCAAAGTTTTAGGATAGTAGTAAAATATCTGGCTCGTGGATGTTAGTTGGGATATGCGTGCGCGTGCTTCGACGCAAGACAATCAACAAGCTGCGAAGCTTTTATAGCGAGCTAATCTTGATAAGATGAACAAGACCGAAAAACACCCGCAGCTACAATAGCTCATAAAAATAGCATTTCTCTAGGGAAATGTTATATCGCAAGAAATATCGTTATCGCAATACTCCACAACAATATCGCATAATTTTCCAGTATCGTGCAGCCCTA

>scaffold_31000054-35

TACACTATATTGTCAAAAGTTTGTGGACACCTGACTATCCGATCTAGATGTGATTCCTCTCCAGAAGGTTGCACACAATTGTCTAGAATGTCTTTGTATGTTGTAGCGTTACATTATTCCTTCACATGCCTCCAGCATGACAATGCCCCTGTGCACAAAACCCCTGAGATCCATTAAGACATGGTGTGTTATGATTGGATTGGATAATCTTGAGTGTTCTGCACAAAGTCCTGACCTTAAAACACCACTGAGCACCTTTGGGATGGACTGCACCACAGACCTCTTTTACCTGATGTTAGTTCCTGATCTCACTAATACTATTGTAACTGAGTGAACACACATCTCACATTCAAGCTCCAAATTGTAGCGGAAAACCTTTCTAGAAGAGTGGAGCTTCATCGAACAGCAAAATGGGGAGTAAATCTGGAATAAGATCTTTAAAATCACATGTGTGTGTAATTGTCAGGTGTGCACAAACTTTTGGCAATATAGTGTA

>scaffold_31000054-160

TTAAAGGGGTCACGAACTCCCTCTGTTTTTTATATTGTATCTCTGAGGTCCACTTATACTGTTAAGATTTTTACATCAAAACAAAGCGATTTCCTGTCCTGTTTTTGACCCCCTTATCAGAATGCACTGTTTGAATAGGCGTGGCAGATTGTTGTCTCGGAAGTAAACGCCCACTGCCATGATTGGCTAACAGTTTTATGTGCGACATTCCGAGACATTCCAATCCGAGACATTTACCAATCACGTGCATGTGCGAATCGGTGGGCGGGGCTAAACAGACAGTGACGTCGAAGCAGGCGTCGCGCTTCTTCTGTGGAGGCGGAGCTTATCCACACTATTACGTCATCGAGCAGAACATTCCAAACCTGTCATTTTAGCCGACTGCCTTCAATAAAAACCGTTTTTGAAGAAACAACAAAGTTTTGAGTTCTGAAACTTACAGGATGTTTTTATAGCACAACGACCTCTTATATATCAAAAGATCAAAGGAATTTTCATTTCTTAGTTCATGACCCCTTTAA

>scaffold_31000056-18

CAGGGCTTGCAAAAAATTTATTCCTGGTAGCCCTTCGGGCAGGCACTCTACAGTTTTTGGTAGCCTGAAATGAATTTAAGTAGCCCGAATAAAAAAAGACATTATTTTTAATGTAGAAGATTGTAAAGGAGACAAAACATCATGAAAAACATTTTCAAAACACAAATAACAATTGTAGGCTATAAAATAATCAAATTACACTCATCAATACAAATATGGTTCTAACTGAACATCCTATTCCAGATGTCAGCAATTTTGGCAATTCTCTCCACTGCGCATCCGACCTCTATCACAAGTTTAAGCTTGCTGCACGAGATTACATTGTAACGTGAGGACAGTAATGACTGAGAGGACGAAGTCAGAGGAATTTGGTACGCAACAGATCAGAAGCGTCACTGACAAATATTGTGTAAAATCAGTACTGCTGCTCCCTACACAGTTTGCGTGATCGCAGATTAAAAGGGAAAATAAAAACCAAGAAAGCATTCAAATGTGATTTCAATACCCCACATATTTACATACATAATTACCAAACTATATTTGTGAGAGAATGCAAAAGCAATGAAATATATTTTCTTCCCTATAAGATAGCAGGGATACATTTTGGTAGCTCAACTGGAAGACAAAATAGCCCCGGGACATCGTGCTAGCAATTTTGCGAGCCCTG

>scaffold_31000058-5

TAGGGCTGTGTAAAAATATCGATACAGTTAACTATCGTGATATTTGTGTTCACGTTAGTGTATCGAAATTCTAACCTGTAGTATCGATACGTATTTAAATGATGTTTTAAACTGATTTACGTTCATATTTAATTAAAACCCTCAGTGTTTATTTGTTTGCTGAGTAATCGATATCATGTGATGCACACACTTTATTTTACTTAAAACATTTCTTTTTCAAGTATTGCGATCTGTTGTATTGAAATATATTGTGATTTATTGTATCGTATCGTATCGTGAGGCCCTTGCCAATACACAGCCCTA

>scaffold_31000058-6

TAGGGCTGTGTATGGGCAAGCGTCGAACGATACGATACATATCACGATACATGGGGCACGATACAATACATCACGATATATTGCAATACTATACATCTTCAAAAACATTTTTTTATGTAAAACAAATAGCTAACAATACAACTTGCTGTGTAGCACCTGGGGGATTTTAGTATGTGTGTCACATTATATTGATTACTCAGCAGACAATGGTAGGGTTTTAATTCAATGTTAACGTAAATGTAAATCGGTTTAAAAAATAATTTAAATATGTATCAATACTACAGGTTAGAATATCGATACACTATCGTAAACAAAAATAATGCGATAGGTAACTGTATCAATATTTTTACACAGCCCTA

>scaffold_31000059-1

TACGGTGGCCGAGAGTGCTCAACGCGCTGCAATTTAAGAAAACGTGCAATTAGAAAAAACACCAACAAATTAAGAAAACGTCTTCATCAGTTTGACAACAGATGCGCTGCAAATCCTCACAACGCAAACGAATACAGAAACGCGCTGCAACTAGCACAGACCACAACGGAAATGTTTCAAGGGGACCCCCCAAAAAAGTGACGAACCCAGCTGGGATTTGTTTATCGTTTCACAGTTAGCTAATTGTGTAATTCATCAGATTATTTAGGCTAATAATTTCCAAAAGACCAAGGAATCATACGATCAGGATGATAAAATAAACCAAAAACTGACCTGTCTGTTCACCGATAACACCACTGACAAATAGCCACTGAACAATAAGCAGGTCCCAGCCGAGACCGTCACTTTTTGTGGTCCCCTTGAAACATTTCCGTTGTGGTTTGTGCTATTTGCTGCGCGTTTCTGTATTTGGTCGTGTTGTGAGAATTTGCAGCGCGTTTCCGTATTCGTTTGCGTTGTGAGAATTTGCAGCACGTCTGTTGTCAAACTGATGCTGTTTTCTTAATTTGTTGGTGTTTTTTCTATTTGCATGTGTTTTCTTAAGTTGCAGCACGTTGAGCTCTCTCGGCCACCGTA

>scaffold_31000059-8

TAGGGCTGTGTATTGGCAAAAGCCTCACGATACGATACATATCACAATATTTTGCAATACAATACATTGCAATACTTCATAAAAAAAAAAAAGGAAAATAAATAGCTAAAACTACAACGTGCTGTGTACCTCCTGGGGGATTCTAGTGTGTGTATCACATGATATTGATTACTCAGCAGACAAATAAACACTGAGGGTTTTAATGAAATGTTAACGTAAATCAGTTTAAAACGTCATTTAAATACGTATCGATACTACAGGTTAGACTATCGATACACTATCGTGAACACAAATATCGCGATAGTTACTGTATCGATATTTTTACACAGCCCTA

>scaffold_31000060-5

ATTAGGGCTGCACGATACTGAGAAATTATGCGATATTGTTGTGGAGTATTGCGATAACGATATTTCTTGCGATATAACGTTTCCCTAGAGAAATGCTATTTTTATGAGCTATTTTAGCTGCGGGTGTTTTTTGGTGCTGGTTCATCTTATCCAGATTAGCTCGCTATGAAAGCTTCGCAGCTTGTTGATTGTCTTGCGTCGAATCACGCGCACGCAAATCCCAACTGACATCCGCGTGCCAGATATTTTAATATCCTAGAACTTTGACCACACATTATTATTTGAAGTATTAAATCGCTCATTTATCGCAAATTATTGGGATATTTATATCTCGACATGCACATTTGCGATATTTCGATATATTGTGCAGCCCTAAT

>scaffold_31000061-2

ATACACTATATGGACAAGTATTGGGACACCTGACCATTACATCCATATGTGTTTTTTATAAGAGCATATTCCAGATTTAGTTCCACTTTTACTTCAATAATAATCTCCACACTTTTGGAAAGGCTTTCCACTAGATTGTGGATGGTGATTTGCTCATTCAGCCTCAAAATAATTAGTGAGTTTAGGTGCTAATGTTGGGTGAGGAGGTCAGGAGTGTTGTAGGAGTTCCAGTTCCTCTTAAAGGTGTTCAGCAGGGTTGAGGTCAGAGCTCTATGCAAGACACTTGAGTACTTCCACTTCAACTATAACACACCATGTTTTATGGAACTTAGGGACTTTGGACACAAGGGCATTGTCGTGCTGGAACAGTGTTTCGGTCTCTTAGTTTTAGTAAAGGTCAAATGTAATGCTACAACATACAAAGACATTATGTGTGTCTTTCCAACTTTGTGTTAACAATTTGGGGAAGACACACTTATATTGGTGTGATTGTAAGGTGTCCACAAACTTTTGCCCATACAGTGTAT

>scaffold_31000062-7

TAGGGCTGTGTATTGGCAAGGGCCTCACGATACGATACGTATCACGATACATGGGTCACGATACAATATATCATGATATATTGCAATACTTCAAAAAAAGCCTAATAGCTATAAATACAACTTGCTGTGTAGCACCTGGCGGATTTTAGTATGTGCGTCACATATTATATTGACATTATATTGATTACTCAGCAGACAAATAAAAACTGAGGGTTTTAATTCAATATTAATGTAAATCAGTTTAAAACGTAATTTAAATATGTATCGAAACTACAGGTTAGAATATCGATACACTATTGTGAACAAAAAATTTTGCGATTAGTTAGCTGTATCGATATTTTTACACAGCCCTA

>scaffold_31000064-1

CTAATGCCGAGTTCACACTGCACGATTTTCAGGTCGTTGGATCACCGTTGTTTTCACACTGCACGATTATCTGGGGTAACATTCAGTTGCTGCTGTGTTCACATGCACGATGAATCGGCGACAGGAGGTTACACACTGCATGACTTTACAATAGGAAGAATCGCCAACAACTCTGTCTGGTCCGCAAACTACGTTTCACAACCAAACGCACGCGAGAAGTGATAGGAAATAACACGTGAGATCAGAGTTCTCGCACAAGACTGGAAATGTTATTAAAATGGTAGCCCGCAAGAAGTTTGCGATTCAAATGGTCTGCGTGCTGATTTACAGTGAAAGAGTGAAACAGAAAAAAAGGATGGGAACCGTAGCCATGCTAGCTGATATTATGGTCTATAACTCCTCCCCTAACTTTCTGCTGTCCTGTATCTTGCTCTCTCATTGGCTGTAGGTCATCACCGGTGATTTTTTCCACTCAGAACTCATTTCACACAGCAGGAGTTTGAATCGCCGACAAGTCCAGATATTTAGCATGCCAAATATTTCACGGGCTCGGCGACGCCTCGGCGATTCTCTCTGATCGCGTCTTTGATCATTCAAACTGCGCAATTGTCGCTCGTGTGAATGAGCAGCGATTTGCCTCTGATTTCGGGCATTCCTCAGCGATTCCGAAAAACCTGTCGGCGAGTGAAAAATCGTGCAGTGTGAACTCGGCATTAG

>scaffold_31000070-11

TTATCCAGAGTGACTTACATTTATCTCATTTTATACAACTGAGCAATTGGGGGTTAAGGCCCTTGCTCAGGGGCCCAGCAGTGGTAACTTGGTGCTGCTGGAAATCGAACCCACGATCTTCTGGTTCGCAGTCCAACACCTTAACCACTACACTTCCACTTCCCCCACTCTGGATAA

>scaffold_31000071-7

TAGGGCTGTGTAAAAATATAGATACAGCTAACTATCGGGATTTTTTTTTTCACGATAGTGTATCGATTTTCTAACCTCTGGTATCGATACACATTTATATTTATTTATTAAGTTTTAAACTGATTTAAATTTACATTAATATTTCAATAAAACTCTCAGTTTTTATTTGTCTGCTGTGTAATCAATATAATGTGATACACATACTAGAATCTCCCAGGTGGTACACAGCAAGTAGTATTTTTAGCAATTTTTTTGTATTGTATTGTAAATATATCGGGATATATTGTATCGTGACCCATGTATCGTGATAAGTATCGTATCGTGAGGCCCTTGCCAATACACAGCCCTA

>scaffold_31000073-1

ACTATATTGCCAAAAGTTTGTGGACACCTGACCATCACAAACGTGTTTCATCTCCAAACGTTTGCCACAAATTTGGAAACACACAGTTGTCTAGAATGTCTTTGTATGTTGTAGCGTTACATTATTCCTTCACTGGAACTATGAGACCCAAACACTGTTCCAGCATGATAGTGCCCCTGTGCACAAAGCCCTGAGTTCCATTAAGACATGGTGTGTTATGATTGGAGTGGAAAAACTTGAGTGTTCTGCATAAAGCCCTGACCTCAATATCACTCAACACCTTTGGAATGAACTGGAACACCAACTTCACCACAGACCTCCTTACCTGACATTAGTGCCTGATCTCACTAATACTATTGTAGCTGAATGAATACATATCTCCCATTCAAGCTCCAGATTGTAGTGTCTAGTCTTACTAGAAAAGTGGAGTAAATCTGGAATAAGATGTTTAAAATCAAATGTTTATGATTGTTAGGTGTCCACAAACTTTTAGCAAAATAGT

>scaffold_31000073-2

ATACACTATATTGTCAAAAGTTTGTGGAGACCTGACCATCAGATCCATTTGTGGTTTCTCTCAAAAATGTTGCTACAAATTTGGAATCACACAGTTGTCTAGAAAATCTATGTATGTTGTAGCATTACATTATTCCTTCACTGGAACTAAGAGACCCAAACACTGTTCCAGCATGACAACGCCCCTGTGCACAAAGCCCCTGAGTTCCATTAAGACATGGTGTGTTATGATTGGAGTGGAAAAACTTGAGTGTTCTGCATAAAGCCCTGACCTCAACACCACTCAACACCTTTGGAATGAACTGGAAACCCGACTTCACCACAGACCTCCTTACCTAACGTTAGTGCCTGATCTTACTAATACTATTGTAGCTGAATGAATACATATCTCCCATTCAAGCTCCAAATTGCAGTGGAAAGCCTTACTAGAAAAGTGGAGCTTAAACTAACAGCAAAATGGGAGGAAATCTGGAATAAGATGTTTAAAATCAAATATGTTTGTGATGGTCAGGTGTCCACAAATTTTTGGTAGTACAGTGTAT

>scaffold_3600001-8

TACAGTAGAACCTTAGCATACAAATTTAATTTGTTCTGGAGGCGAGTTTTCAAGTAAAAATTTGTATTGTGATACGAATGTTTTCATAAGAAATTATGTAAATGCAGCTAATCAGTTCCAGCCGCTCAAAATTATTACGAATATTACCAATTTCCAACATTAGAATAATATTTTTGAATAAAAAACCAATCCAAACATTTAGAAAAGACATGAAAGTGAAGTAAAATATGAAATAAATCGACCTCCGCCACCAATCTGGGGAAAAAAAATGTGCAAAAAATCACCTAGCTTTTGAACTCGTGCCGACGTTTGTATTGTGTGTCAACTCTTTGGAACCAGCTTTCGCTGACTGAAAAAAGAAATCGTAAAAGACGTAAACTCTCTTCGGTTGGCTTCGCTCAGCTTTCCACTCACGCAAACAAGTTTTAAAGAGCTCCCGAACGTATGCGCAACTTAGCTGTAACTGTTAAAATAGTTCGTTCGTATGCCGACAATGCTTCGAACTGTGATGCAAATCTTGGCCAAATTTCAATTCGTAAGGCAAAAATTCATAAGAGAGGGTTTTGTATGCCGAGGTGCCACTGTA

>scaffold_390000-5

ATTAGGGCTGTGTATTGGCAAGGGCCTCACGATACGATACACATCACGATACAATATATCACGATATATTGCAATACTTGTTACGTGCATCACATGATATTGATTACTCAGCAGACAAATAAACACTGAGGGTTTTAATGAAATATTAACGTAAATCAGTTTAAAACGTCATTTAAATATGTATCGATACTAGAGGTTAGAATATCGATACACTATCGTGAAAGAAAATATTGCGATAGTTAGCTGTATCGATATTTTTACACAGCCCTAAT

>scaffold_390000-13

ATTAGGGCTGCACGATACTGGGAAAATCTGCGATATTGTTGAGTATTGCGATAACGATATTTCTTGCGATATAACATTTCCCTACAGAAATGCTATTTTTATTAGCTATTTTAGCTGCGGGTGTTTTTCAGGCTGGTTCATCTTATCAAGATTATCTCGCTATGAAAGCTTCGCGGTTTGTCGATCGTCTTGCGTCAAATCACGCGCACGCAAATCCCAACTAACATCCGCGTACCAGATATTTTACTATCCTAAAACTTTAACCACACGTTAATATTTAAAGTATTAAATCGCTCATTTATCGCAAACCGTTACCATATGCACATTTGCGATATTTTGATCATTTCGATATATTGAGCAGCCCTAAT

>scaffold_3900001-9

TACACTATATAGACAAACGTATTGGGACACCTGACCATGACATCCATATGTGTTTTTTAAACATCTTATTCCAGCTTTAGTCTCACTTTTACTCCTATAAGAACCTCCACACTTCTGGAAAGGCTTTTTATTAGGTTGTGTCATGTGACTGTGGGGATTTGATCATTCAGCTACAATAGTATTAGTGAGATCGGACTCTAATGTTGGGGGAGGAGGTCTGGGGTGCAGTCAGGGTTCCAGTTCATCCCAAAGGGGTTCAGTGGGGTTGAGTCAGAGTCAGGGTTCTGTGCAGGACATGAGAGTTCTTCCACTCCAACCAGAACACAGCATGTCCTCATGGAGCTCAGGGGTTTTGTGCACAGGGGTATTGTTATGCTGGAACAGGGTTCGAGTCTCTTAGTTCCAGTGAAGGATTAATGTTAATGCTACAACATATAGAGACGTCCTAGAAAACTGTGTGCTTTAGAATTTGTGGTAGCAAACACTAAGACACACTAATGTGGGTGTTCTGGTAAGGTGTCCACAAACTTTAGCCAATTTAGTGTA

>scaffold_3900002-1

CTACACTATATTGGCAAAAGTTTGTGGGCACCTGACCATCAGATCCATATGTGGTTCCTCTCCAAAATGTTGCCACAAATCTGAGTCACACAGGTGTCTAGAACGTCTTTGTATGGTGTAGCGTTACGTTATTCCTTCTCTGGAACTAAGAGACTCGAACCCTGTTCCAGCATGACAATGCCCCTGTGCACAAAACCCCTGAGCTCCATGAAGACATGAGTGTCCTGCACAGAGCCCTGACCTTAACACCACTGAAAACAGCATTGGGATGAACTGGAACGCCGAATGCACCCCAGACCTCCTCACCCGACGATCTCACTACTACTATTGTAGCTGAATGAACACACATCTCACATTCAAGCTCCATATTGTAGTGGAACGCCTTACTAGAAGAGTGGAGCTTTAATCGAACAGCAAAATGGGGAGTAAATCCGGAGGTTTAAAATCACATATGTTTGTGCTGGTCAGGTGTCCACAAACTTTTGGCCATATAGTGTAG

>scaffold_470000-4

ACTAGGGCTGTACAATATATTGAAATATCACAAATATGCATATTTCGGCAATGGTTTGTGATAAATGAGTGATTTAATACTTTAAATATTAATGTGTGGATAAAGTTTTAGGATAGTAAAATATCTGGTACGCGGATGTTAGTTGGGATTTGCGTGCGCGTGATTTGACGCAAGACAATCAACAAACTGTGAAGCTTTCATAGCGAGATAATCATGATAACATGAACCAGCCAGAAAAACACCTGCAGCTAAAATAGCTAATAAAAATAGCATTTCTCTAGCGAAATATTATATCACAAGAAATATCGTTATCGCAATACTCAACAATAATAATATTGCATATTGCATATTTTCCCAGTATTGTGCAGCCCTAGT

>scaffold_4700004-7

TTAGGGCTGTGTATCGGCAAGGGCCTCACGATACAATACATGGGTCACGATATAATATATCACGATATATTGCAATACAATACAAATTGCAATACCTGCTGTGTACCACCAGGGGGATTCTAGTATGTGTATCACATTATATCGATTACTCAGCAGACAAATAAACACTGAGGGTTTTAATTAACGTAAACGTAATTTAAATATAAACGTAATTTAAATATGTATCGACACTACAGGTTAGCATATCGATACACTATCGTGAAAAAAAATATTGCGATAGTTATCGATATTTTTACACAGCCCTAA

>scaffold_4700006-3

TGGCTCCGTCCGAAATCGCATACTTCCATACTATATAGTGTGCAAAACAGAGTATGCGAGGCGAGTAGTATGTCCGAATCCTTAGTATGTGAAAAACAATAGGCGAAAAGTTCCCGGATGATCTACTACTTCCGCCTGAATTATGAAGTACGCATACGATGGACACTTTACTATCCCATGAGGCCGCGGGAGAGACGACTCGTCATATGCGGAAGTGATGGAAAGCAGGCACTTTTCAAGTTCTTTAAGTACTTAAAGAAAAAAATATTGTTCTTTTTGGTTAAATTGCACTCGTTTAAAATCATCCAAGTTAAACATTATCCAACTTTAAATTTGCGCTGCTGTGACGTCGTATGTCACGTGAACGTATCAACATGGAGGATGTAGTACGTCCCAATTTCATTCATACTCCCCCATAATCATACTATATAGAATGTACTTTTTTAACGGTCGGGAAGTACGTACTCACTTAAATGTAGTGCATAATTAAGTAGTGTACGATTTCGGACAGAGCCA

>scaffold_4700007-1

CTAAAGCCGGACTTACACTGTGCGATTTTGGCCAAGATTTGTTTGTCTGTGACAAATAATTTTGAAATCCTAAAAGATTCTTATAATCCTACGCTAAAACCTGTTGTGTTTGATGGCTAGTTTGACATGTTCACCGACAGCTGATTAACGGCCGTTGCGATCAGTTTTTTCCTCCTATGAAATTCTGGCAGTGTGAGAAGATTTCAGACACTTTCCTGCAGTGTGACGTCTCCTACGATGACGTCAAACCAAGAACCAATAGCAGCGCCGAACCTGATGACGCAATTTCAAACCACTTCGGGAAAATGTGGAAACGAGTCCAGTGGACAGAACAGCAAGAAAGGAAACTTATTAATGCATGTCATAAACAAAATTACGCTACAGATTGTTTTTGTTTGCTGCTAGCTACCATTTGGCGCAAGAACGCGGGTTACTGAACGTGTCACGGATCGATGATGTAAAACTCCGGACGAGTCTTCTTGTGTGCGTCTGTTTTTGACGCGTCTTGACTGTCGTACAGTCTAACATTATAACAACTGAGATCCTACAGTGTGACATGGGGATCATGTTCGTACAGTCTGACAAACAACAATCGTAAAAGACTATTAAAAATCGAAGTGTAAGCCCGGCTTTAG

>scaffold_4700008-2

ATATCCTCCTGAGACCCCGCCCATTGACTTTTGTCCTCTGTAGTGGACATTTTGTTTTCATAAATGTTTTTGGAATTTTTGGAGCTACTCTATTAATTTGCTGTACTGTACAGAGGACATCCTGGGCTTTCTAGTGATGTCATTTGATTGGCTGGGATGCAAGAAACTAGTTCTTACACTACATCCAAAATGGCCGCCATAAGAACAAGCACAGTTTATTGTTTTTATTTATGAAGTCTAGAGTGGACATGCTGTAAAATTTAAAATGATGATAAAATTTAAAAACTGTAAATTGTAAAATGTTTTTTAACCTTAAAGAAGTAGAAAAATCACACAAAACAAAAATGATTACCCTTTTTTTCCTTGGGTCTCAGGAGGATAT

>scaffold_4700008-4

GGCTCCGTCCGAAATCGTATACTTCGATACTATATATGTGAAACTGAGTATGCGAGGTGAGTAGTTTGTCCGAATCCTTAGCATGCAAATTAAAGTATGCGAAAAGTTCCTCAATGACCTACTACTTCCGCCCGGATTTTGAAGAACGCATGCGATGTACACTTTACTATCCCATGATCCGGCGGGAGAGATGACTCGTCATATTGGCGACGCGGCACTTTACAAGTGCTTTAAGTAGCTTTAAGTACTTAAAGAAAAAAAACATTGTTCCTTGTGGTTAAATTGCATTTGTTTAACATCATTCAAGTTAAACATTATCCAACTTTAAATTCATGCTGCTGTGACGTCGTATGTCACGTGTCAATATAAACATGGCGAATGTAGTACATCCGAATTTCATTAATACTACCGTAATCATACTATATAGAACAGACTTTTTTAACGGTCGGGAAGTGTGCACTTACTTAAATGTAGTAATATGGGATTTCGGACGGAGCC

>scaffold_4700008-7

TAAAGCCGGATTTACACTGCGATTTTGGTCACGATTTGGTCGACAAATTTTGAAATCCTAAAGATTCCTATAATCTTACACTAAAATCTGTAGTCTTTGATGGCTAGTCTGACATGTTGACCGACAGCCAATTGATGTAATCAGTTTTTTCCTCCAATGAAATTCTGGCAGTGTGAGAAGATTTCAGACACTTTCCTGCAGTGTGACGTCTCCTACGACGACCGCCAAACCAAGAACCAATAGGAGCGCCGAACCTGATAACTCAATTAGCACGACAACTTCAAACCACTTCAGGAAAATGTGGAAACGAGTCCGGTGGACAGAAAAGCAAGAAGAGAAACTAATTGAGTTATGGAGGAAAAGGAGAGTTTGTGTGACGTGTCGTCGCTGCTGTTCATTTAGCGAGAGAACGCGGGTTACTGAATGTGTCACGGATTGATGATGTAAAACTCCGGACGAGTCTTCTTGTAGGTGTCCGTATTTGATGGACAGTTTGTTGTACATTTGTCGTACAGTCTGACATTATGACAACTGAGATCCTACAGTGTGACATGGGGATCATGTTCGTACAGTCTGACAAACAACAATCGCAAAAGACTATTAAACATCACACGTGTAAACCCGGATTTA

>scaffold_4700009-4

CTTAAACCAGGTTTACACTGTACGAATTTGGTCAAAATTTGGTTGTCTGTGACAAATTTTGAAATCCTAAAAGATTCCTAAAGTCCTACTCTAAAATCTGTAGTCTTTGATGGCTAGTTTGACGTGTTCACCGACAGACGATTTATGACTGTTGCGATCAGTTTTTTCCTCCGATGAAATTCTGGCAGTGTCGGATGATATCAGATACTTTCCTGCAGTGTGACGTCTCCTACGATGACCGCCAAACCAGCCGAACCTGACGACGCAATTAGCGCGACAACTTCAAACCACCTCGGGCAAATGTTTAAACAAATCCAGTGGACAGAACAGCAAGAAGAGAAACTTATTGAGTCGTGTCATGGACAAAATTAATGCCACAGATTGTTTATTTGCTGCTAGCTACCATTTAGCGAGTGAACGCGGTTTACTGAACGTGTCATGTATAGATGACCTAAAACTCCGGATGAGTCTTCTTGTGTCCGTCTGTTTGTGATGCGTCTTGACTGTCGTACAGTCTGACATTATGACAACTGAGATCCTACAGTGTGACACGGAGATCATGTTCGTACAGTCTGACAAACAACAATCACTAAAGACTATTAAACATCGCACAGTGTAAACCCGGCTTAAG

>scaffold_4700009-5

GTAGGGCTGCACGATACTGGGAAAATATGCAATATGCGATAACAATATTTTTTGTTATATAATATTTCCCTAGAGAAATGCTATTTTTATTAGCTATTTTAGCTGCAGGTGTTTTTCAGGCTGGTTCATCTTAGCAAGATTAGCTCGCTATGAAAGCTTCGCAGTTTGTTGATTGTCTTGCGTCAAAACACGCGCACGCAAATCCCAACTAACATCCGCGTACCAGATATTTTACTATCCTAAAACTTTAACCACACATTAATATTAAAGTATTAAATCGCTCATTTATCGCAAACCATTACAATGTGCACATTTGTGATATTTCTATAATTTCGATATATTGTGCAGCCCTAC

>scaffold_4700009-13

ATTAGGGCTGTGTATTGGCAAGCGCCTCACAATACAATACACATCACGATACATGGGTCACGATACAATATATCACGATATATTTCAATACAATACATATTGCAATACTAAATAAAAAAATGCAACTTGCTGTGTAGCACCTGGGGGATTCTAGTATATGTATCACATTATATTGATTACTCAGCAGACAAATAATCACTGAGGATTCTAATTAAATATTAACATAAATCAGTTTAAAACGTAATTTAAATATGTAGCGATACTAGAAGTTAGAATATCAATACACTATCGTGATTTTTTTTTGCGATAGTTGTCTGTATCGATATTTTTACACAGCGCTAAT

>scaffold_47000010-4

TATACAGGGTGGGTGAAAATGAACTAGGCAATATTTCATGGCTGTAGAACTTGTAGTATCGCTGGAGTCCTGATGAAAACTCTAAACGGGATTTCAAGCTTCGCACACTTGCGGACTTGGAGGCACGGATTCGGAAGGTTCTCTGCAATATCCCAAACCACTTTCTTCCTTCCATCTCCGGCCGTTTAAGGAAACTAGTTGACGGCACCGGTGCCTACATTAAAATTGAAAGATTTGCTTTCCTTTTTCTATGTAATGAAGTACATCATACATACATCATTGTTTCAATACATTAGTATTAGAAATATGGATTTTATTACCAATTTTTAATGCCTAGTTACTTTTCACCCACTGTATA

>scaffold_47000010-14

TTACAGGGGTCACGAACTGAAAACTCAAACTTCCTTTGATCTTTTGACATATCCGTTGTGCTATAAAAACCTCCTGGAAGTTTCAGAACTCAAAACTTTGTCGTTTCTCTAAAAACAGCTTGTATTGAAGGCAGTCGGACAAAATGACAGGTTTTGGAATGTTTTATTCGATGATTTAATAGTATGGATAAGCCCCGCTTACGTTGAAGAAGATCGACACCTGCTTCGACATCACTGTCTGTTTAGCCTTGCCCACAGATTCGCGCATGCACGTGATGGGTAAATGTTTTTAAATGCAAAACGGTTAGCCAATCATAGCAGTGGGCGTTTACTTCCGAGACAACAATCCAAAGCCACGCCTATTCATACAGAGCGTCCTGATGAGGGGGACAGAAAACAGGACAGAAAATAACCTATTACTTCCACATTAATTTATTTTTTGATGTAACAACCTTTATAACATTATTATAAGTGCACCTCAGCGAACAGTACAAAATTAAAAAAAACTGAGGTAGTTCATGACCCCTTTAA

>scaffold_47000011-2

ATGCCGAGGTCACACTGCACGATTTTCAAAGTCGTTGGATCGCCGTTGTTTTCACACTGCATGACTATCTGGTGTAACATTCAGTTGCTGCTTTGGTCACATGTACGATGGATCGGCGACAGGAGGTTACAAACCGCATGACTTTACAATAGGGAGAATCTCAGACAACTCTGTCTGGTCCGCAAACTACGTTTCACAACCAAACACACGTGAGAAGTGATAAAGAAATAACGCAAGATCACGTGTGAGATCAGAGTTCATGTGCGAGAAGTTCCCGATACACTTAGGTCTGTGTGCTGATTTGCAGCGAAAGGGCGAAAAAGAAAAGAAGATTAAAAAAAGATGGGAACCGAAGTCATGCTTTCTGATATTGTGGTCTATAACTCCTCCATGAACTTCCTGCTGTCCTGTATCTTGCTCTCTCATTGGCTGTAGGTCAGCGCCGAGGTATTTTTCAGTCAGAACTCATTTCACACTGCAGGATTTTGATTCGCCGACAGGTCCAGATATTTAGCATGACAAATATTTCACGGGCCTCTGAGATTTTCTCAGATCGCGTCATTGATAATTTGATAATTCTTTGATAATTTCGGGCATTTGATGGCGATTTCGAAAAACCTGTCGGCGAGTGAAAATCAGGGCTAAAATCGTGCAGTGTAAACTCGGCAT

>scaffold_47000011-4

TTAACCCTTGTGTGGTGTTTGGGTATGTGGGATCCGATTTCAAATTTTGTTTAAAGAGAAATTATGCTATTAATTATTTTTTTAGCCCCAGACTCATTGGCCTTGGCTCATTTTTCATGTAGAACATAAAATATTTTCCATAACACTTATATTTTCTAATGTGATCTAATGGGGTAAATGGCAAATGTTAACCATAAATGATGTCTATATTGCTTGTAAGTGAGCTAAAGTTTACACATTTTAAGTGTTTTTTCCATAAATTGTTATAGCTGCGTTGATTGAAAACCCAATAACGCGGTGGGTCCACCAGACCCGTGAACATTGGCTTGGTAACAAAAACATAAACACCACACGAGGGTTAA

>scaffold_47000011-6

TAGGGACGCACCGATACCATTTTTTAAAGACAGAGTACGAGTACGGATAGTTTTTTCCTGGTACTTGCCGATAGCGATACCTGTACTTTTTTGGGATGTGGGATTATTTGTATGGCTCTCGAGTTTTCTCTTGCCATTTTCTCTCGTCGTTACTGCTAGCGGAAAATTCTCCGTGCTCACTTTAGTGTTTTAATTTCAGATGTTTTATCAGATTGCTCGTATTATAAGTACTTATTTTTGAAGCTCTTGATATTTTTGCGGATAAGTTTGCAGTCTGACATGCGTGGGTTCATCATTCATTTTGAAATATTTCTACACCGCTGAGTCTGACATTCTGTCGCTGCTGCCGGAGTCTTTGTACACTGAAAGTTCTGTCAGTGACGTCACAAAAGATATCGGTCTTTGGTATTGAAGTTTTTTTTTTTTTTACGAGTATGAGTACATGAGCTCAGTATCGGGCCCGATACTGATACCCGTATCGGTGCATCCCTA

>scaffold_47000013-13

TAGGGCTGTGTATTTTGGCAAGGGCCTCACGATACAATACATCACGATATATTGCAATACTTACATAAATAGCTAAAAATACAACTTGCCGTGTAGCACCTGGGGGATTCTAGTATGTGTACCACATTATATTAATTACTCAGCAGGCAAATAAACACAGGGTTTTAATTAAGTATTAATGTAAATCAGTTTAAAACGTCATTAAATATGTATCGAAACTAGAGGTTAGATTATCGATACACTTTCATGAAAGATAATATCGCGATAGTTAGCTGTATGGATATTTTTTACACAGCCCTA

>scaffold_47000017-4

AACTAGGGCTGCACGATACTGGGAAAATATGTGATATGCGATATTGTTGTTGAGTATTGCGATAACGATATTTCTTGCGATATAATATTTCCCTAGAGAAATGCTATTTTTATTAGCTATTTTAGCTGCAGCTGTTTTTCCGGCTGGTTCATCTTATCATAATTAGCTCGCTATGAAAACTTTCATAGCGTTTGATTTGTCTTGAGTTTGTTGATTGTCTTGCGTCAAATCACACGCACGCAAATCCCAACTAACTATTTTACTAAATATCTAAAACTTTATCCACACATTATTAATTGAAGTATTCAATCGCTTATTTATCACAAACCATTGCCATATGCACATCTCGATATGCACATTTGCGATATTTCGATAATTTCGATATATTGTGCAGCCCTAGTT

>scaffold_47000019-16

TAGGGCTGTGTAAAATTATCGATACTGCATTGTTTTTTCTCACGATAGTGTATCGATATTCTAACCTCTCGTATCGATACATATTTAAATAACGTTTTAAACTGATTTACGTTAATAATTCATTAAAACCCTCGGTGTATATTTGTCTGCTGAGTAATCAATATAATGGGATACACATACTAGAATCCCTCAGGTGGCACACAGCAAGTTATATTTTTAGCTATTTGTTTTGTACAATTTGTATTGCATTGCAATATATTGTGATTTATTGTATCGTGACCCATGTATCGTATCGAGCGGCCCTTGCCAATACACAGCCCTA

>scaffold_47000021-2

TAGGGATGCACCGATACTGATACAGGTATCGGTATTGGGCCCGATACTGAGCTCATGTACTCATACTCGTAAAAACACTCCAATACCAAAGACCGATACCTATTGTGACGTCATTGACAGAACTTTCAGTGCACAAAGACTCCGGCAGCAGCAACAGAATGTTTGACTCAGCGGTGTGGAAATATTTAAACATGAATGATGACAACTCACGCATGTCAGACTGCAAACTTTGTTTCTCAAAAATATCAAGAGGAACAAAAATGAGTACTTATAATACGAGTAATCTGATAAAACATCTGAAATTAAAACACAAAAGTGAGCACGGAGAATTTGCTGCTAGCAGTAACGTTAACACTCGGGAACAAAACCTGCAGCAAACTCTTACAAGACGAGAGAAAATGGCAAGAGAAAACCCGAGAGCCATACAAATAATCCCACATCCCAAATAAGTACAGGTATCGGCGAGTACCAGGGAAAAAATATCGGTACTCGTACTCGGCCTTTAAAAAATGATATCGGTGCATTCCTA

>scaffold_47000021-3

CAGGGCTTGCAAAATTTTTGAATTCCTGGTAGCCCTTCGGACAGGCACTCTTTAGTTTTTGGTAGCCCGAAATGAATTTAAGTAGCCCGAATAAAAAAAAGACAGTATTTTATATATAGAATATTGTAAAGGAGACAAAACATCAATCAAAAACATTTTCAAAACACAATCATATAAAATAATCTAATTATAATTATCAATACAAATATTTGGTACTAACTGTTCCAGATGTCAGCGATTTTCGCAATTCTCTCCACTGCAAATACGATCTCTGTCACATGATTAAGCTTTCTGCGCGAGATTGCACTTGTCATGTGACGATGGTAATGATTGACAGGATGATGTCGGAGGAATTGGTGCGCAACAGATCCGGCACGTCACTGACAAATATTGTGTATAATCAGTAACGCCGCTTCCGATACGCAGTTTACGCAATTGCAAATTGAAAGTGAAAGTAAAAAGCGAGCGCGCATTCAAACACCATTTCAATACACAACATATTGACAGTGGCTCGGAAATTGCAGACAACATAATTACCCAACTATATTTGTGAGAGAATGCAAAAGCATTGAAATATATTTTTCCTCCCTGTGAGATAGTCCGATGGGCAGGGCGGGGATACATTTTGGTAGCCCAACAGGACACAATAGACCCAGGACATCGGGCTAGCGATTTTGCGAGCCCTG

>scaffold_47000023-8

TAGAGCTGCACGTTACTTGGAAAATCTGCGATATTGTTGTTGAGTATTGCAATGACGATATTTCTTGCGCTATAATATTTCCCTAGAGAAATGCTATTTTTATTAGCTATTTTAGCTGCAGGTGTTTTTCGAGCTGGTTCGTCTGATCAAGATTAGCTCACTATAAAAACTTCGCAGTTTGTTGATCGTCTTGCGTCAAATCACGCGCACGCAAATCCCAACTAACATCCGCGTACCAGATATTTTACTATCCTAAAACTTTAAATACACATTATTATTTGAAGTATTAAATCGCTCATTTATCACAAACCATTACCATATGCACATGTGCTAATTCGGTAATTTCGATATATTGTGCAGCTCTA

>scaffold_47000024-2

ACATACATTATATTGCCAAAAGTTTGTGGAAATCTGAACGTCAGATCCATATGTGGTTCCTCTCCAAAATGTTGCTTGAAAGCACACAGTTGTCTAGAATGTCTTTGCATGTTGTAACCAGTTACCTGTTCCAGCATGACAATGCCGCTGTGCACAAAACCCCTGAGCTCCATGAAGACATGCTGTGTTCTGGTTGGAGTGGAAGAACTCTCATGTCCTGCACCTTAACACCACTGAACCCCTTTGGGATGAACTGGAACGTCGACTGCACCCCAGACCTCCTTACCTGACGTTAGTGTCTGATCTCATTAATACTATTGTAGCTGAATGATCACATCTCTCACATTCAAGCTCCAAATTGTAGTGAAACGTCTTACTAGAAGAGTGCAGCTTAATCTAACAGCAAAATGGGGAGTAAATCTGGAATAAGATGTTTAAAATCACATATGTTTGCGAAGGTCAGGTGTCCATAAACTTTTGACAATATAGTGTATGT

>scaffold_47000024-3

ACATACACTATATTGTCAAAAGTTTATGGACACCTGACCTTCGCAAACATATGTGATTTTAAACATCTTATTCCAGATTTACTCCCCATTTTGCTGTTAGATTAAGCTGCACTCTTCTAGTAAGACGTTTCACTACAATTTGGAGCTTGAATGTGAGAGATGTGATCATTCAGCTACAATAGTATTAATGAGATCAGACACTAACGTCAGGTAAGGAGGTCTGGGGTGCAGTCGACGTTCCAGTTCATCCCAAAGGGGTTCAGTGGTGTTAAGGTGCAGGACATGAGAGTTCTTCCACTCCAACCAGAACACAGCATGTCTTCATGGAGCTCAGGGGTTTTGTGCACAGCGGCATTGTCATGCTGGAACAGGTAACTGGTTACAACATGCAAAGACATTCTAGACAACTGTGTGCTTTCAAGCAACATTTTGGAGAGGAACCACATATGGATCTGACGTTCAGATTTCCACAAACTTTTGGCAATATAATGTATGT

>scaffold_47000025-2

ACATACATTATATTGCCAAAAGTTTGTGGAAATCTGAACGTCAGATCCATATGTGGTTTCCTCTCCAAAATGTTGCTTGAAAGCACACAGTTGTCTAGAATGTCTTTGCATGTTGTAACCAGTTACCTGTTCCAGCATGACAATGCCGCTGTGCACAAAACCCCTGAGCTCCATGAAGACATGCTGTGTTCTGGTTGGAGTGGAAGAACTCTCATGTCCTGCACCTTAACACCACTGAACCCCTTTGGGATGAACTGGAACGTCGACTGCACCCCAGACCTCCTTACCTGACGTTAGTGTCTGATCTCATTAATACTATTGTAGCTGAATGATCACATCTCTCACATTCAAGCTCCAAATTGTAGTGAAACGTCTTACTAGAAGAGTGCAGCTTAATCTAACAGCAAAATGGGGAGTAAATCTGGAATAAGATGTTTAAAATCACATATGTTTGCGAAGGTCAGGTGTCCATAAACTTTTGACAATATAGTGTATGT

>scaffold_5100001-11

ATATCCTCCTGAAACCCAAGGGCAAAAAAAGTGTCTTAATTTATTTTGTGTTGTGTGATTTCCTACTCCTTTTGGGTAAAAAAGAAAAATTCTACAATTTAGTTTTTAAATTTTATTATTTTTACAGCACTGTAGTGGACCACAGGATTATTTTAGTTTGAAAAGACAGTCATTTCCATAAAATGTGCTTGTCCTTATGGCAGCCATATTGGATGCAGTGCAAGAACTAGTTCCTAGCATCCCAGCCAATCAAAGGACATTCCCCTAAAACTCCCAGGATGTCCTCTGTACAGTACAGCAGAAATTGATAGAGTAGCTAAAAAAATTCAAGAAACATTTATAAAAACAAAAGGTCCACTAAAGAGGACAAGTCAATGGGCGGGGTCTTAGGAGGATAT

>scaffold_5100002-1

TACAGGGTGGGCGAAAATTAACTAGGCAATATTTCATGGCTATAGAACTTGTATGGAGTCATGATGAAAACAGTCTGTAAATAGACGATAGTAAATAGGATTTCAAGCCTCGCACACTGGAGAATTTAGAGGCACGGATTCGGGAGGTTCTCAGCAATATCCCATCACCGGCCGTTTGAGGAAACTGGTTCAAAATTTACAAGATTTTCTTTCATTTTCCTATGTAATAAATGACATGTACAAATTGTTTCAATAAATTTGCATTAGAAATATGGACCTTATTACCAATTTTTAATGCCTAGTTACTTTTCACCCACCCTGTA

>scaffold_5100002-2

TACACTATATAGACAAAAGTTTTGGGACACCAGACCATCACATCCATCTGTGTTTTTGAACACCCTATTCCAGATGTAGTCTCACTCTTCCTCCTATAATAACCTCCACATTCTGGAAAGGGGCAATGAGGATTTGATCATTCAGTCACAACAGTGTTAGTGAGATCAGACACTAATGTTGGGTGAGGAGGTCTGGGGTGCAGTCGGTGTTCCAGTTCATCCCAAAGGTGTTCAGTGGGGTTGAGACAGAGTCGGTGCTCTGTTCAGGACACTTGAGTTTTTCCACTCCAACCAGAACACGCCATGTCCTCATGGAGCTCGGGGGTTTTGTGCACAGGAACATTGTCATGCTGGAACAGTGTTTGAGTCTCTTAGTTCCAGTGAAAGAATAATATAATGCTACAACCTACAGAGACATTCTAGACAACTGTGTGCTTTTAACTTTGTGGCAACAGTGAGGGGAAGACACACATGTGGGCATGCTGGTGAGGTGTCCACAAACTTTTGGCCTTATAGTGTA

>scaffold_5100004-1

TAACATACACTATATTGCCAAAAGTTGGTGGACACCACACCATCAGATCCATATGTGGTTCCTCTCCAAGATGGTGCTACAAATTTGGAAGCGCACAGTTGTTTAGAATGTCTTTGTATGTTGCAGCATTACATTATTACTTCACTGGAACTAAGAGACCCAAACACTGTTCCAGCATGACCATGCCCCTGTGCACAAAACCCCTGAGCTCCATGAAGACATGGTGTGTTATGATTGGAGTAGAAAAAATTGAGTGTTCTGCACAAAGTCCTGAACTTAACACCACTGAACACCTTTGGGATGAACTGGAACGCCGACTGCAGCACAGACCTCCTTACCTGATCTCACTAATACTATTGTAGCTGAATGAACACATCTCTCACATTCAAGCTCCAAATTGTGGTGGAAAGCCTTACTAGAAGAGTGGAGCTTAATCTAACAGCAAAATGGGAGTCAATCTGGAATAGGAGGTTTAAAATCACATATGTTTGTGATGGTCAAGTGTCCACAAACTTTTGGCAATCTAGTGTATCTTA

>scaffold_5100004-13

CTAAAGCCTGGCACACACTGTGCAATTTTTTGTATTTTGCGATTGTTTCTTGTCAGACTGTACAAACTTGATTCCCATGTCACACTGTAGGATATCAGTTGTCATAAGGTCAGACTGTACGACAGTCAAGACACGTCAAAAACAGACACACACAAGAAGACTCGTCCGGAGTTTTACGTCATCAGTCCGTGACACGTTCGGATGTGTTCTCTCGCTAAACGGTAGCTAGCAGCAAACAAACATTAGTGATGACAAGTCATACAAACACTCCTTTTCCCCTCCATAACTCGGCAATTTATCTTCTTGCTGTACTGGCCAAATGACTCGTTTTGACATTTTCCCGAAATAGTTTGAAGTTGTCGCATTAATTGCGTCATCAGGTTCGGCGCTCCTATTGGTTCTTGGTTTGACGATCGTTGTAGGAGAAGTCGCGCTGCAGGTCAGTGTCTGAAATCTTATGAAACTGCCAGAATTTAATCGGAGAAAAAAACTGATCGCAACGGCCATTAATCAGCTGTCGGTCAACATGTCAAACTAGCCATCAAAGACAACAGATTTTAGCGTAGCATTACAGGAATCTTTTAATATTTCAAAGTTTGTCTCAGACGACCAAATCATGGCCAAAATCGCACAGTTTAAGGCCGGCTTTAG

>scaffold_5100008-1

ATATTGCCTAAAGTTTGTGGACACCTAAAAATCATATCACAAACATATGATTTTAGACATCTTATTCCAGATTTACTACCATTTTGCTGTTCGATTAAGCTCCACTCTTCTTCACTACAATTTGACGCTTGAATGTGAGAAGAGTTCGTTCAGTTCAGTATTAGTGAGATCAGACACTAATGTCAGGTGTTGCAGTCAGCGTTCCAGTTTATCCCAAAGGTGTTTAGTGTTCTGCACAAAGCCCTGACCTTACCACCACTGCAGTTTTTCCACTCCAGTCATAACACACCATGTCTTAATGGAACTCAGGGGTTTTGTGCACAGGGACATTGTCATGCTGGAACAGTGTTTGTGTCTCTTAGTTCCAGTGAAGGAATAATGTAATGCTACATCATACATAAACATTCTAGACAACTGTGTGCTTCCAAATTTGTAGCAACATTTTGGAGAGGAACGAAATATGGATCCGATTGTGAGGTGTCCACCAACTTTTGGCATTAT

>scaffold_5100009-24

AGGGCTGCACGATACTGGGAAAATATGCGATGTTGTTGTTGAGTATTGTGATAACGAAATTTCTTGCGATATAACATTTCCCTAGAGATATGCTATTTTTATTAGCTATTTTAGCTGCGGGTGTTTTTCAGGCTAACTCATGTTATCAAGATTAGCTTGCTATGAAAGCTTCGCAGCTTGTTGATTGTCGCGTCAAAGCACACGCACACATATCCCAGCTAACATCCAGATATTTTACCATCCTAAAACTTTGACCACACATTACTACTTGAAGTATTACATCGCTCATTTATTGCAAACCATTGCGATCTGCATATCTCGATATGCACATTTGCAATAGTTCGATAATTTCGATATATTGTGCAGCCCT

>scaffold_51000010-1

ATATACACTATATTGCCAATAGTTTGTGGACACCTGACCATCAGATCATAATGTGGTTCCTCTCCAAAATGTTGCTACAAATTTGGAAGCACACAGTGACTTTGTATGTTGTAGCATTACATTATTCCTTAACTGGAACTAAGAGACACAAACACTGTTCCAGCATGACAATGTCCCTGTGCACAAAACCCCTGAGCTCCATGAAGACATGGTGTGTTATGGTTGGAGTGTTTTGCACAAAGCCCTGATCTGAACGTCACTGAACACCTTTGGGATGAACTGGAACGCCGACTGCACCACAGACCTCCTTACCTGACGTTAGTGTCTGATCTCACTAATACTATTGTAGCTGAATGAACACATCTCTCACATTCAAGCTCCAAATTGTAGTGGAAAGCCTTACTAGAAGAGTGGAGCTTAATCTAACAGCAACATGGGAGTAAATCTGGAATAAGATGTTTAAAATCCCATATGTTTATGATGGTCAGGTGTCCACAAACTTTTGGCAATATAGTGTATAT

>scaffold_51000010-2

TATATTGCCAAATGTTTGTGGACACCTGACCATCAGATCCAGATGCGGATCCTCTCCAAGATGGTGCTACAAATTTGGAAGCACACAGTTGTCTAGAATGTCTATGTGTGATGTAGCATTACATTGTTTCTTCACTGGAACTAACAGACCCAAACACTGTTCCAGCATGACAATGTCCCTGTGCACAAAACCCCTGAGCTCCATTAGGACAGGGTGTGTTATGATTGGAGTGGAGTGGACAAAGCCCTGATCTGAACATCACTGAACACCTTAGGGATGAACTGGAACGCCGACTGCACCACAGACCTCCTTACCTGACGTTAGTGTCTGATCTCACTAATACTATTGTAGCTGAATGAACACATCTCTCACATTCAAGCTCCAAATTGTAGTGGAAAGCCTTACTGGAAGAGTGGAGCTTAATCTAACAGCAACATGGGAGTAAATCTGGAATAAGATGTTTAAAATCCCATATGTTTATGATGGTCAGGTGTCCACAAACTTTTGGCAACATA

>scaffold_51000010-3

TACACTATATGGCCAAAAGTTTGTGGACACCTTACCAGCACACCCACATAAGCGTGTCTTCCCAAACTGTTGCAACAAAGTTAAAAGCACACACTTGTCTAGAATGTCTTTGTATGTTGTAGCATTACATTATTCCTTCACTAAAAGGTCCAAAAACACTGTTCCAGCATGACAATACCCCTGTGCACAAAACCCCTGAGCTCCATGAAGACATGCTGTGTTATGGTTGGAGTGGAATGACGAGAGCGTTCTGTACAGAGCCCTGACTCTGACTCAACCCCACTGAACACCTTTGGGATGAACTGGAACACAGACCTCCTCACCTGACATTAGTGTCTTAACTCACTAATACTATTGTGACTGAATGAGAAAATCCTGGTAGCCATGTTCCAAAATTGAGTAAAAAGCCTTTCCAGAAGTGTGGAGGTTATTATTGGAGTAAGAGTCAGACTAAATCTGGAATAAACATATGCGGATGTGATGGTCTGGTGTCCCAATACTTTTGTCTATTTAGTGTA

>scaffold_51000010-4

TTAAAGGGGTCATGAACTACCTCAGTTTTTTATTTTGTACTGTTCTCGGAGGTCCACTGATAATGTTATCAAGATTTTTACATCAAAAAACATCATAATTTAGAAGTAATAGGCACTTTTCTGTCCTGTTTTTCACCCCCCCTCATCAGAACGCTCTGTTTGAATAGGCGTGGCAGATTGTTGTCTCGAAAGTAAACACCCACTGCTATGATTGGCTAACAGTTTTATGCCGCTCTTGTCATTTACCTATCATGTGCATGCGCGAATCGGTGGGCGGGGCTAAACAGAGAGGTGTCGTTCTTCTTCTGCGGAGGCGGAGCTTATCCATAATATTACGTCATAGAGTAGAACATTCCAAAACCTGTCGTTATGGCCGACTGTCTTTAATACAAGTTGTTTTTAGAGTAAAGAAAGTTTTGAGTTCTGAAACCCACAGGATGTTTTTCTAGCACAATGGCCTCTTATATGTCAAAAGGTCAAGGGAATTTTAATTTCTCAGTTCATGACCCCTTTAA

>scaffold_51000011-2

ATAAACTATATTGCCAAAAGTTTGTGGAAATCTGACCATCACAAACATATGTGATTTTAAACATCTTATTCCAGATTTACTCCCATGTTGCTGTTAGATTAAGCTGCACACTTCTAGTAAGGCTTTCCACTACATTTTGGAGCTTGAATGTGAGAGATGTGTTCATTCAGCTACAATAGTATTAGTGAGATCAGACGCTAACGTCAGGTAAGGAGGTCTGTGGTGCAGTCGACGTTCCAGTTCATCCCAAAGGTGTTCAGTGACGTTCAGATCAGGGCTTTGTGCAGAAAAACAAGTTTTCCCATTCCAATCATAACACACCATGTCTTCATGGAGATCAGGGGTTTTGTGCACAGGGACATTGTCATGCTGGAACAGTGTTTATGTCTCTTAGTTCCAGTGAAGGAATAATATAATGCTACAACTTACAAAGACATTCTAGCCAAATGTGTGCTTCCAAATTTGTAGCAACATTTTAGAGAGGAACCACATATTAATCTGATGGTCAGGTGTCCACAAACTATTGGCAATAAAGTGTAT

>scaffold_51000012-1

CAGGGCTGGGTAACCCATGTTCCTTAAGTTACTTTGTTCTGCTTGTTTTCCAGCTACCCCCGCCCTACCCACTGCTGATTACCTGGTTCAGGTGTGTTCAGTCAATCAGAAGCTGGAAGATACCATTTCAGATGAGGGTGAAGTGGGGGAAAACCTGGTGATTTGGTATCTCCTACCTTCTGATTGACTGAACCCACCTGGAAAACATGCAGAACAAAGTAACTTAAGGAACATGGGTTACCCAGCCCTG

>scaffold_51000014-2

TATAGGGGTGGGAATCACAGGTTAACTCACGATACGATACTGTCACGATATTTTGCCCACAAAAACGATATATCACAACACCAGCGATTCTGTGACAGACGATATATTGCAAGAAAATCATTTACGATACATCACGATATCGCTAAATAAATAAAAAATGCATCAAAAAGTCATATGATGTATTTCATTTCTTAAGAGTACATCTATTTTTATAAAAATCTATTTTATCACTTCACAAGACACTGGGGCTTCTGCCGCTCCGGTAAGCCAAAGTGGTAAACAGCCACGATGTAGGACAGGTATGAATTTAGAGCTTTTGTATGTTTAAAAAATATCAATACATGGTGCAAGAGTATCGATAACGCCTCACGGCCGGAAATATCGCGATAAATCGGCAAATCGATTTTTTGTCCCACCCCTATA

>scaffold_51000022-4

TACACTGTATGGGCAAAAGTTTATGGACACCTTACCAGCACTTCTACATAAGCGCGTCTTCCCCAAACTGTTGCCGCAAAGTTTGAAGCACACAATTTTCTAGAATGTCTTTGTATGTTGTAGCATTACATTATTCCTTCACTGGAACTAAGAGAACCAAACAGTGTTCCAGCATGACAGTGCCCCTGTGCACAAAACCCCTGAGCTCCATGAAGACATAGTGTGTTCTGACTCAACCCCACTGAACACCTTTAGGATGAACTAGAAACACTGACTGCACCCCAGACTTCCTCACCCAACATTAATACCTGATCTCACTAATACTATACTAACAAATCCGCACAGTCACGCAACACAATCTAGTGGAAAACCTGCACAGAAGTGTGGAGGTGATTATAGGAGGAAGAGTGAGAATACATTTGGAATAGGATGTTCAAAAAACACATATGGATGTGATGGTCAGCTGTCCCAATACTTTTGTCTATATAGTGTA

>scaffold_51000023-1

ATACACTATATTGCCAAAAGTTTGTGGACACCTGACCATCACAAACATGTGATTTTAATCCAGGATTTTATTCCCCATTTTGATTTACTCCCATTTTGCTGTTAGATTAAGCTGCACACTTCTAGTAAGGCTTTCCACTACATTTTGGAGCTTGAATGTGAGATGTGTGTTCATTCAGCTACAATAGTATTAGTGAGATCAGACACTAACGTCAGGTAAGGAGGTCTGTGGTGCAGTCGGCGTTCCAGTTCATCCCAAAGGTGTTCAGTGATGTTAAGATCAGGGCTTTGTGCAAAACACTCAAGTTTTCCCACTCCAATCATTACACATCATGTCTTCATGGAGCTCAGGGGTTTTGTGCACAGAGGCATTGTCATGCTAGAACACTGTTTGGGTCTCTTAGTTCCAGTAAAGGAATAATGTAATGATACAACAAACAAAAACATTCTAGACAACTGTGTGCTTCCAAATTGTTTAGCAACATTTGGAGAGGAACCACATATGGATCTGATGGTGAGGTGTTTACAAACTTTTGGCAATAATATAGTGTAT

>scaffold_5500006-2

TACGGGGTGTGTGAAAAGTAACTAGGCATTAAAAATTGGTAATAAAGTCCATATTTCAAATACAAATTTATTGAAACAAATTGTACATGTACTTTATTACACAGGAAAATTAAAGCAAATCTTTACATTTTAACGTAGGCACCGGTGGCGTCAACCAGTTTCCTCAAACAGCGGAGATGGAATGTGCAGTCTTCTGAAGGAAGTGGTTTGGGATACTGCTGAGAATCTCCCAAATCCGTGCCTCCGAGTCCTCCAGTGTGCGAGGCTTGAAATCTTATTTACTATCGTCTATTTACAGACTGTTTTCATCAGGACTCCAGTGATACTACAAGTTCTACAGCCATGAAATATTGCCTAGATCATTTTCACCCACCCCGTA

>scaffold_5500006-9

TAGGGCTGCACAATATATCAAAATTATTAAAATATCGCAAATGTGCATATCAAGATATACATATCGTAATGGTTTGTGATAAATGAGCGATTTAATACTTCAAATATTAATGTGTGGTTAAAGTTTTAGGATAGTAAAATATCTGGTACGCGGATGTTAGTTGGGATTTGCGTGCGCGTGATTTGACGCAAGACAATAAACAAACTGTGAAGTTTTCATAGCGAGTTAATCATGATAAGATGAACCAGCCCGAAAAACACCTGCAGCTAAAGTAGCTAATAAAAATTGCATTTCTCTAGGGAAATATTATATCACAAGAAATATCGTTATCGCGATACTCAACAACAACATTGCATATTGCATATTCTGCTAGTATCGTGCAGCCCTA

>scaffold_55000012-26

TTAAAGGGGTTAAGAACTGAGAAATTCCTTTGATCTTTTGACATATAAGAGGTCATTGTGCTATAAAAACATCCTGTAAGTTTCAGAACTCAAAACTTTGTCTTTACTCTAAAAACAGTTGGCAGTCGGCCAAAACACAGGTTTTGGAATGTTCCACTTGATGATGTAATAGTGTGGATAAGCTCCGCCTCTGCAGAAGAAGATCGACACCTGCTTCGACATCACTGTCTGTTTAGCCCCGCCCACCAATTCGCGTATGCCCGTGATAGGTAAATGACAAGAGCGGCATAAAACTGTTAGCCAATCATAGCAGTGGGCGTTTACTTCCGAGACTATAATCTGCCACGCCTATTCAAACAGAGGGTTCTGATGAGGGGGTCAAAAACAGGACATAAAATCGCCTATTACTTCTAAATGATGATGTTTTTTGATGTAAAAATCTTGATAACCTTATAAGTGGACCTCAGAGAACAGACCAAAATAACAAACTGAGCTAACATGACCCCTTTAA

>scaffold_55000012-35

TTAAAGGGGTCATGTTAGCTCAGTTTGTTATTTTGGTCTGTTCTCTGAGGTCCACTTATAAGGTTATCAAGATTTTTACATCAAAAAACATCATCATTTAGAAGTAATAGGCGATTTTATGTCCTGTTTTTGACCCCCTCATCAGAACCCTCTGTTTGAATAGGCGTGGCAGATTATAGTCTCGGAAGTAAACGCCCACTGCTATGATTGGCTAACAGTTTTATGCCGCTCTTGTCATTTACCTATCACGGGCATACGCGAATTGGTGGGCGGGGCTAAACAGACAGTGATGTCGAAGCAGGTGTCGATCTTCTTCTGCAGAGGCGGAGCTTATCCACACTATTACATCATCAAGTGGAACATTCCAAAACCTGTGTTTTGGCCGACTGCCAACTGTTTTTAGAGTAAAGACAAAGTTTTGAGTTCTGAAACTTACAGGATGTTTTTATAGCACAATGACCTCTTATATGTCAAAAGATCAAAGGAATTTCTCAGTTCTTAACCCCTTTAA

>scaffold_55000021-1

ACTATATTGCCAAAAGTTTGTGGACACCTGACCATCACAAACATGTGATTTTAAACATCTTATTCCAGATTTACTCCCCATTTTGCTGTTCGATTAAGCTCCACTCTTCTAGTAAGACTTTCCACTACAATTTGGAGCTTGAATGTGAGATTTGTGTTCATTCAGCTACAATAGTATTAATGAGATCAGACACTAATGTTGGGGGAGGAGGTCTGGGGTGCAGTCGGTGTTCCAGTTCATCCCAAAGGTGTTCAGTGGGGTTGAGTCAGAGTCAGGGTTCTGTGCAGGACATGAGAGTTCTTCCACTAACCAGAACACAGCATGTCTTCGTGGAGCTCAGGGGTTTTGTGCACAGGGGCATTGTCATGCTGGAACAGGGTTCGAGTCTCTTAGTTCCAGTGAAGGAATAATGTAATGCTACGACATACAAAGACGTTCTAGACAACTGTGTGCTTCCAAATTTGTGGCAACCTTTTGGAGAGGAACCACATATGGATTTGATGGTGAGGTGTCCACAAACTTTTGGCAATATAGT

>scaffold_55000021-2

TACACTATATTGCCAAAATGTTGTGTACACCTGACCATCAGAGCCATACAGTATGTGGTTCATCTCCAAATGGTTGCTACAAATTTGGAAGCACACAGTCGTCTAGAACGTCTCTGTATGTTGTAGCATTACATTCTTCCTTCACTGGAACTAAGAGACTCGAACCCTGTTCCAGCATGACAATGCCCCTGTGCACAAAGCCCCTGAACTCCATGAAGACATGCTGTGTTCTGGTTGGAGTGGAAGAACTCTCATGTCCTGCACAGAACCCTGACTCTGAATCAACCCCACTGAACCCCTTTGGGATGAACTGGAACCCCGACTGCACCCCAGACCTCCTCCCCCAACATTAGTGTCTGATCTCATTAATACTATTGTAGCTGAATGAACACAAATCTCCCATTCAAGCTCCAAATTGTAGTGGAAAGTCTTACTAGAAGAGTGGAGCTTAATCGAACAGCAAAATGGGGAGTAAATCTGGAATAAGATGTTTAAAATCACGTGTTTGTGATGGTCAGGTGTCCACAAACTTTTGGCAATATAATGTA

>scaffold_55000021-5

AAGCCGGTTTTACACTGTGAGATTTTAGCCACGATTTGTTCGTCTGAGACAAATGTTGAAATCCTAAAACATTCCTATGATCCGACGCTAAAATCTGGTGTGTTTGATGGCGAGTTTGACGTGTTCACCGACAGACGATTAATGACCGTTACAATCAGTTTTTACCTCCGATGAAATTCTGGCAGTGTGAGAAAATTTCAGACACTTTCCTGCAGTGTGACGTCTCCTACGACGACCGTCAAACCAAGAACCAATCAGAGCGCCGAACCTGATGACGCAATTAGTGCGAGAACTGCAAACCACCTCGGGAAAATGTGTAAACGAGTCCGGTGGACAGAACAGCAAGAAGAGAAACTTATTGAGTTATAGAGGGAAAAGGAGTGTTTGTATGACGTGTCGTCGCTGCTGTTTGTTTGCTGTTAGCTACCGTTTAGCGAGAGAACGCGGGTTACTGAACGTGTCACGGATTGATGATGTAAAACTCTTTTTCTTGACAGTCGTACAGTCTGACATTATGACAACTGAGATCCTACAGTGTGACACGGGGATCATGTTCGTACAGTCTGACAAACAACAATCGCTAAAGACTATTAAACATCACACAGTGTAAACCCGGCTT

>scaffold_55000021-7

GGCTACGTTCACACTGCAGGCAAATGAGGCCCAAATCCAAATTTTTTTGCCCACATGTGATTCGGGTCTGTTTTTCTCATGACAGTGTGAACAGCACAAATTCCGTTTGGAATCTATTCAATTCCGATTTGTGCCACTTCCATATATGGTATTAAATCCGACACAGGTCAGATGTTATGCAATGCGACCGCAGTGTGAACAGTTATGTCGGAATTCACGCGACTTTTACGTCATTTTAGATGGACATGCGTCATCATTCTGCACTGTCGCGTAACTACAAACAGTTGATATATAATCGATTTCTCTGAAGGCGTTGATTACGTCACGGTCCCACCACTCCTGACTCCGGCTCCACATCCACACACACATACTGAAGTAGCAGCCACCGCTCCACAACATGCTATAATTACAAAACATTTAACCCTTTTTCTCCTCATCCTCTTCCTCCTCAACGACAATAATTCGCTGTCGTCCGCTACACATCACCTTATAAATGTAAGCAGCTAACGCCGTCAGTGAGTTCTGTGTTTATTTTTCCGTATGACAGGACGCGGTGTGAGACGTAGAATTAGTTCCTTTGTGCATGCGGATCAGTTTAGGACCAGGATCAGTTCACACTGGAAATCTGATATTGGCCACATTTTAAAACAACAATGTAAACAGCTCTACAAAAAAATCTGATCTGAGCAATAATCGAAATTGAGCACTACACCTCGCAGTGTGAACGTAGCC

>scaffold_55000021-10

TTACCCAGCTAACAGGGAACGTTCCCACAACTTCTGTTGTTGTTCTTTAAAAGTTTAAAATAACGTTTGTGGAACATTCTCATAAGGTTATTAACCGTTAATATACATTCTTGCGATGTTGAGAGAAAACGTTCTTAGAACAATGTTCTTAAAACATCCTTAAGAGGTTATTCCTACTTGATGAATATTCTCAAAATGTTTAGAGAAAACGTTCTTAGAACAACACCATGAAATAACCATAAAAAAATTCTACCAACGTTAAGCAAACTGGACAGTTTTACGTTTATGGAATATTTAAAATCATGTTCCCAAAACAGAGCGTTTGAAAAACGTTCTCAGAACATAAATTTGTTAGCTGGGTAA

>scaffold_55000021-22

TTTAACCCTTGTGTGGTGTTTGGGTCTGTGGGACACGATATCAATGTTTGTTTAAAGAGAAATGATGCTATTAATAATTTTTTTAGCCCCAGACTTATTGACCTTTGCTCATTTTCCATGTAGAACATAAAATAGAACATATTTTCAAATGTGATCTAACGGGGTAAATGGCGAATATTATCCATGAATGATGTCTATATTGCTTGTAAATGAGCTAAAGTAAACATCTTTAAGTGTTTTTCCATCAATTGTTATGGCTGTGTTGATTAAAAACCCAATAATGCGGTGGGTCCACCAGACCTGTGAACATTGGCTAGGTATTAAAAACACGAACACCACATGGGGGTTAAA

>scaffold_55000022-2

AAGCCGGGTTTACACTGTGAGATTTTGGCCACGATTTGTTCGTCTGAGAAATTTTGAAATCCTAAAACATTCCTATGATCCGACGCTAAAATCTGGTGTGTTTGATGGCGAGTTTGACGTGTTCACCGACAGACGATTAATGACCGTTACAATCAGTTTTTACCTCTGATGAAATTCTGGCAGTGTGAGAAGATTTCAGACACTTTCCTGCAGTGTGACGTCTCCTACGACGACCGTCAAACCAAGAACCAATCAGAGCGCCGAACCTGATGACGCAATTAGTGCAACAACTTTAAACCACCTGGGGAAAATGTGTAAACGAGTCCGGTGGACAGAACAGCAAGAAGAGAAACTTATTGAGTTACAGAGGGAAAAGGAGTGTTTGTATGACGTGTCGTCGCTGCTGTTTGTTTGCTGTTAGCTACCGTTTAGCTCCGGACGAGTTCTTGTGTGCATCCGTTTTTGACACGTTTTGACTGTCGTACAGTCTGAAATTATGATAACTGAGATCCTACAGTGGACACGGGGATCATGTTCGTACAGTCTGACAAACAACAATCGCAAAAGACTATTAAACATCGCACAGTGTAAACCCGGCTT

>scaffold_55000022-3

AAGCCGGGTTTACACTGTGCTGTTTTGGCCGCGATTTGTTCGTCTGAGACAAATTTAGAAATCCTAAAAGATTCCTATAATCCAACACTAAAATCTGGTGTGTTTGATGGCGAGTTTGACGATTAATGACCGTTATAATCAGTTTTTAACTCAGATGAAATTCTGGCAGTGTGAGAAGATTTCAGACACTTTCCTGCAGTGTGACGTCTCCTACGACGACCGTCAAACCAACAACCAATCAGAGCGCCGAACCAGATGACGCAATTAGCACGACGACTTCAAACCACCTGGGGAAAATGTGTAAACGAGTCCGGTGGACAGAACAGCAAGAAGAGAAACTTATTGAGTTATAGAGGGAAAAGGAGTGTTTGTATGACGTGTCGTCGCTGCTGTTTGTTTGCTGTTAGCTACCGTTTAGCGAGAGAACGCGGGTTACTGAACGTGTCTACGGATTGATGATGTAAAACTCCGGACGAGTTCTTGTGTGCGTCCGTTTTTGACACGTTTTGACTGTCGTACAGTCTGAAATTATGATAACTGAGATCCTACAGTGTGACACGGGGATCATGTTCGTACAGTCTGACAAACAACAATCGCAAAAGATTATTAAACATCGCACAGTGTAAACCCGGCTT

>scaffold_55000022-5

ATACAGTGGACCCTCGGTTTTCCTGATTAATCCGTTCGAAAAAGTCTGATGAAAACCGAATCGTACGAAAACTGAAGCAATATTTCCCATAAGAAATAATGTAAATCCAATTAATCCGTTCGGCACCCAAAAATATTAACATAAAATACATTTTATAGAGAATAACTATATTTTTACATACAGAAAACAATGAGAAATAAATATAAATGACTAAAGAAATGGATAAATGAACATTTAACATCACTTTTACCTTTATTGAAGATTCTTGTTGGCGTATTTCGTACTTTGCTACGAGTTCTTTCTTGAATTCTATCGTGTTTCTCGCCTGCTTTATCAAACGGCTGGCACTCTGGACTTTCTTTGGCCCCATGGTGGCTTATTTAGCAGTCGCACTCAATAAACATTGAGGAAATGCGGAAATGCTCGATTCCGAGTCACGCGAGCAGGTGGACGAGTTTGGTACGGACCATTTTCAACTGTACGAAAACTGGGACAAAATTTTTACGAAAAAAGTTGTTGAAACCCGAATTGTACGAAAACAAGTTCATACGAAAACCGAGGTTCCACTGTAT

>scaffold_55000022-6

CTTAGGCTACGTTCACACTGCGAGGTGTAGTGCTCAATTCCGATTATTGCTCAGATCAGATTTTTTTGTAGAGCTGTTTACATTGTTGTTTTCAAATGTGGCCAATATCAGATTTCCAGTGTGAACTGATCCTGGTCCTAAACTGACCCGCATGCACAAAATAACTAATTCTACGTCTCGTGCAGCGTCCCGTCATACGGAAAAATAAACACAGAACTCACTGACGGCATTTACGCGTTAGCTGCTTACATTTAGAAGGTGATGTGCAGCGGACGACAGCTAATTAATGATCAGTCATTGAGGAGGAAGAGGAAGAGGAGAAAAGGGATAAATGTTTAATAATAATAGCATGTTGTGGAGCGGTGGCTGCTACTTCAGTATGGAGGTGTGTGTGGATGTGGAGCCGGAGTCAGGAGTGGTGGGACCGTGACGTGATCAACGCCTTCAGCCAAATCGATTATATATCAACTGTTTGTAGTTACGCGCCAGTGCAGAATGATGACATGTCCATCGAAAATGACGTAAAAGTCGCGTGAATTCCGACATTACTGTTCACACTGCGGTCGCATTGCATAACATCTGATCTGTGTCAGATTTAATACCATATATGGAAGTGGCACAAATCGGAAATTGAATAGATCAGATTCCATGCGGTTTGTGCTGTTCACACTGTCATGAGAAAAAACAGATTCACATGTGGGTAAAAAAAATTTTGAGCCACATGGGATTTGACCCACATTTGCCTGCAGTTTGAACGTAGCCTAAG

>scaffold_55000022-8

TATCCTCCTGAGACCCAAGGGAAAAAGTGTCATCACTTTGTTTTGTTTTGTGTGATCTCCTACTCTTTTTAGGTTAAAAAACATTCTACAATTTAGAGTTTTTAAATGTTATTGTTATTGTTATTTTTAATTTTATAGCATGTCCACTGCAGTGGACCACATGACCAATTTAGTTCTTTCCATAAAACATGCTTGTTTCTATGGTGGCCATATTGGATGTAGTGTAAGGTCTAGTTCCTAGCATCCCAGCCAATCTAATGCCACGTCACTAGAAATCCAGAAGTCCAGGATGTCTTCTGATAGAGTAACTCAAAAAATTCAAAAAACATTTATTAGAACAAAATGAGGACGCAAGTCAGTGGGTGGGGTCTCAGGAGGATA

>scaffold_55000022-9

AATTAGGGCTGCACGATACTAGGAAAATATGCAATCTGCGATATTGTTGAGTATTGCGCTAACGATATTTCTTGCGATATAATATTTCCCTTGAGAAATGCTGTTTTTATTAGCTATTTTAGCTGCGGGTGTTTTTCAAGCTATCTCGTCTTATCAAGATTAGCTCGCTATGAAAGCTAACATCCGCATGCCAGATGTTTTACCATACTAAAACTTTGATCACACACATTACTCTTTGAAGTATTAAATCGCTCATTTATTGCTAACCATTGCTATATACATATCTCAATATACACATTTGCGATATTTTGATAATTTCGAGATATTGTGCAGCCCTAATT

>scaffold_55000022-11

AAGTCGGGTTTACACTGTGTGGTTTTGGCCACGATTTGTTCGACTGAGACAAATTTTGAAATCCTAAAAGATTCCTGTAATGCTACGCTAAAATCTGTTGTCTTTGATGGCGAGTTTGACGTGTTCACCGATTAATGACCGTTACAATCAGTTTTTACCTCTGATGAAATTCTGGCAGTGTGAGAAGATTTCAGACACTTTCCTGCAGTGTCACGTCTCCTACGACGACCGTCAAACCAAGAACCAATCAGAGCGCCGAACCTGATGACGCAATTAGCACGACGACTTCAGATCATCTCAGGAAAATGTGTAAACAAGTCCGGTGGACAGAACAGCAAGAAGAGAAACTTATTGAGTTATAGAGGGAAAAGGAGTGTTTGTATGACGTGTCGTCGCTGCTGTTTGTTTGCTGTTAGCTACCGTTTAGCGAGAGAACGCGGGTTACTGAACGTGTCACGGATTGATGATGTAAAACTCCGGACGAGTTCTTGTGTGCGTCCGTTTTTGACGCGTCTTGACTGTCGTACAGTCTGAAATTTTGACCACTGAGATCCTACAGTGTGACACGGGGATCATGTTCGTACAGTCTGACAAACAACAATCGCAAAAGACTATTAAACATCAGTGTAAACCCGGCTT

>scaffold_55000025-6

GGCTCCGTCCGAAACACATACTACTTAAGTAGGTACTACATTTAAGTTAGTACGCACTTCCTGACTGTTAAAAAAGTACGTTCTATATAGTATGGTTACGGCTAGTATGAATGAAATTGGGACTTACTACATCCGCCATGTTGATATTGTCACGTGACATACGACGTCACAGCAGCGTGAATTTAAAGTTGGAAAATGTTTAACTTGATGATGATAAACGAGTGTTTTAACTACACGGAACAATGTTTTTCCTTAAGTACTTAAAGCACTTGTAAAGTTCCGCGTCGCCGCTTTACGCCACGAGTCGTCTCTCCCGCGGCCTCATGGGATAGTGTAAATGTATACATCGTATGTATACTTCAAAATCCAGGCGGGAGTAGTAGGTCATCTGGGAACTTTTCACGTACTGCATTTCGCATACTAAGAATTTGGACATACTACTCGCCTTGCGTACTCAGTTTCGCATACTATATAGTATGGAAGTATGCGATTTCGGACGGAGCC

>scaffold_55000025-7

TAAAGGCTCCGTCAGAAATCGCATACTTGCATACTATATAGTGTGTGAAACTTAGTATGTGAGGCGAGTAGTATGTCTGAATCCTTAGTATGCGAAATACAGTACGCGAAAAGTTCCCGGATGACCTACTACTCCCGCCCGGATTTTGAAGTATGCACACGATGTACACTTTACTATCCCATGATCCCGCGGGAGAGACGACTCGTCATATGCGGAAGTGGTGGAAAGCGGTGACGCGGCACATTTCAAGTGCTTTAAGTACTTAAAGAAAAGAACATTGTTCCTTGTGGTTAAATTGCTCTCGTTTAACATCATTCAAGTTAAATATTATCCAACTTAAAATTTGCGCTGCTGTGACGTCGTATGTCATGTGTCAATATCAACATGGCGGATGTATTGCGTCCGAATTTCATTCATACTAGCCGAACCATACTATATAGAACGTACTTTTTTAATGGTCGGGAAATACTTACTCACTTAAATGTAGTACATACTTAAGTAGTATACAATTTCGGACGGAGCCATTA

>scaffold_55000027-1

TTAACCCTTGTGTGGTGTTTGGGTCTGTGGGACCTAATTTTAAAGTTTGTTTAAAGAGAAATTATGCTATTAATATTTTTAAGCCCCAGACTCATTGACCTTGGCTCATTTTCCATGTACAACTGAAAATAGAACATTTTCCATAACACTTATATTTTCTAATGTGATCTAACGGGGTAAATGGCGAATATTAACCATAAATGATGACTACATCGTTTGTAAGTGAGCTAAAGTAAACATCTTTTAAGTGTTTTTCCAAAGTTGTTATGGCTGTGTTGATTAAAAACCCAATAATGCGGCGGGTCCACCAGACCCATGAACGCTGGCTAGTTAACAAAAACATGAACACCACATGAGGGTTAA

>scaffold_55000028-2

AAGCCGGGCTTACTTTGTGCGATTTTGGCCACGATTTGTTCGTCTGAGACAAATTTTGAAATCCTAAAACATTCCTATAATCCTACACTAAAATCTGTTGTCTTTGATAACATGTTTACAGAAAGCCGATTTATGGCTGTCGTGATCAATTTTTTCCTCAGATGAAATTCTGTCAGTGTCAGAATTTTTCAGACACTTTCCTGCAGTGTGATGTCTCCAACGACGACCGTCAAACCAAGAACCAATAGGAGCGCCAAACCTGATGAGGCAATAAGCACGACAACTTGAAACCACCTCGGGAAAATGTGGAAACGAGTCCACTGGACAGAACAGCAAGGAGAGAAACTTATTGAGTTATAGGAGGGAAAAGGAGTGTTTGTATGACGTGTCGTCGCTGCTGTTTGTTTGCTGCTAGCTGCCGTTTAGCGAGAGAACGCGGGTTACTGAACGTGTCACGGATTGATGACGTAAAACTCCGGACGAGTCTTCTTGTGTGCGTCTTGACTGTCGTACAGTCTGACATTATGACAACTGAGATCCTACAGTGTGACATGGGAATCATGTTCGTACAGTCTGACAAGCAACAATCGCAAAAAATCGCACAGTGTAAGCCCGGCTT

>scaffold_55000029-3

CAGGGGTTAAATTGGGATTTGTTATGGGGGGGGCTCTATGGTGTTGGGCTTTTAAGGGGTGCACATGTGTATGCAAAATCATTGAGTCGTTCATTCAAACGATTCGTTCAGACGGCTGATTCATCAGATGTTTATGCATGGGCCAATGAAACTCTGACACTGAATCATTCAGCAGTGAATGGGATGCGCTGTGGTTCTGCTGGGTCTTTCTTTGGAATTATTTTCACTGTTGAAATAAAACAAAAGCCATCTGCAATACTGCTCCTAAAATATAAGTGACCTAATATTATTGTACTTGCTTATTAAACTGTTTATTGAACTATTGAACAGTCGTGATGGTGAAAACGTCGTGTGATGTTGCTTAATTAACTGTAATTTAAATATAAAAACTTTTCATTTGGAATTTTGACCTCAGAATGCCGAGTTTGTTTGTGTTGCGCGTTTTGATTTCTCCTGGAGAGACTGTGTGATCCTCGCCTCAGCAGCGCCACCTACTGTCAATATGCGTTATATACAGTAGCTATATCCACTATTCGCCGCGCACTCTAAATGTAATAAAATCACAATAATACTTGCGTTTTTTGGCGATTGCCTAGTTATTTTTGGTTATTGATGTTTTAATCAGGTTACTTGTCTAGCGTTAATAAAGCTTTAATGTCGAGCCCTGGAGTCTATGTGCCGGGGCTCAGCCCCGGACGTCCCCAGCCCAATTTAAACCCTG

>scaffold_55000029-7

TTAAAGCTGCAGTTCGGAACTTTTTTGGGGTTAAAAATGACCTGAAATGAATTATTGAGCAAGGACATTACAAACCAGTGTTCAAAACCATCTCCTTATCGTAGCCCGATTCACAATGGTAAGCTTGTAATAATGTTTTATAATTGAGGTGATACTGGTAGGTTTTCGTGGGAAATAAGAGCATGGCGCCGTTCGTCTTTGCGTCATAACGTCACGTTCGTAAAGATAAAGAAGTAGTCCCAACTATATCCCATGCAAGGATGCTAACGCTAATGCTAATACACACTAAATACACGTAGTCACACAATGCTGATGTTGTTAAAATGTATAATTTGAGAACAAATTATAACAATGATAATTTGCACGGTTTGATGTGATATGAGCTCATTAGCGATTGTTAGATTAAATCTGCATTGTTAGCGTGATTTATTGTAATGCTTTTTTTTTCCTCAGTTAGTCAGAACAAAAGTGGCAGTTACTTGCTCAGATGACATTCTCTAGTGAAAATCCTTATTTGGGTCAAACTTTCCACCAAAGTACAGTATCCACACCAGTACGGTGACTGACAGCCCACATACACACCGAAAAACATCAGATTCATCCGCGCTGAGGAGCCGTGCCGATGTATAACCCATATAAAGATTTATATACAGAATTTCACAAATAACTGCAATTGCAGGTTTCAAACAGAGATGGCGACCAAGAGGCAACACTTACGGACTGCAGCTTTAA

>scaffold_55000030-1

TACACTGTATTGTCAAAAGTTTGTGGACACCTCACCATCAGATCCATATGTGGTTCTTCTCCAAAATGTTGCTACAAATTTGAAGCACACAGTCGTCTAGAATGTTTTTGTATGTTGTAGCGTTACGTTATTCCTTCACTGGAACTAAGAGACCCAAACACTGTTCCAGCATGACAATGCCCCTGTGCACAAAACCCCTGAGCTCTATTAACACATGGTGTGTTATGACTGGAGTGGAAAAAACTTGAGTGTTCTGCACAAAGCCCTGACTTTAACACCACTGAACACGTTTAGGATGAACTGGAACGCCGACTGCACCACAGACCTCCTTACCTGACGTTAGTGTCTGATCTCACTAATACTATTGTAGCTGAATAAACACACGTCTCACATTCAAGCTCCAAATTGTAGTGGAAAGCTTTACTAGAAGAGTGGAGCTTAATCGAACAGCAAAATGGGGAGAAGATCTGAAATAAGATGTTTAAAATCACATATGGTTCTGATGGTCAGGTGTCCACAGACTTTTGGCAATATAGTGTA

>scaffold_55000030-2

ATACATTATATTGCCAAAAGTTTGTGGACACCTCACCATCAGATCCATACGAGGTTCCTCTCCAAAATGTTGCTACAAATTTGAAGCACACAGTCGTCTAGAATGTTTTTGTATGTTGTAGCGTTACGTTATTCCTTCACTGGAACTAAGAGACTCAAACACTGTTCCAGCATGACAATGCCCCTGTGCACAAAACCCCTGAGCTCTATTAACACATGGTGTGTTCTGACTGGAGTGGAAAACTTGAGTGTTCTGCACAAAGCCCTGACTTTAACACCACTGAACACGTTTAGGATGAACTGGAACGCCGACTGCACCACAGACCTCCTTACCTGACGTTAGTGTCTGATCTCACTAATATTATTGTAGCTGAATGATCACACATCTCACATTCAAGCTCCAAATTGTAGTGGAAAGCTTTACTAGAAGAGTGGAGCTTAATCGAACAGCAAAATGGGGAGAAGATCTGAAATAAGATGTTTAAAATCACATATGTTTCTGATGGTCAGGTGTCCACAAAGTTTGGGCAATATAGTGTAT

>scaffold_55000030-6

TGGCTCTGTCTGAAATCGCATACCTCCATACTATATAGTATGCAAAACTGAGTATACGAGGCGAGCTGTATGTCGGCATTAATAAGTATGCGAAATACAGTACGCGGAAAGTTCCCGGATGATCCACTTCTCCCACCAGGATTTTTAAGTATGAATACGACGGACACTTTACTATCCCATGAGCCCGCGGGACAGATGACTCGTCATATGCGGAAATAGCGGAAGGCGGCGACTTTACAAGTGATCTAAGTACTTCAGGGGAAAAAACATTGTTCCTTGCAGTTAAATTGCACCCTTTTAACATCATCAAGGTAAAAACATTATCCAACTTTAAATTCGTGCTGCTGTGACGTCGTATGTCACGTGACAATATCAACATGGCGGGTGTAGTACATCCAAATTTCATTCATACTACACATAATCATACTATATAGAATACTTTTTTAATGGTCGGGAGTACATACTAACTCAAATGTAGTACATACCTAAGTAGTATGCGATTTAGGACGGAGCCA

>scaffold_55000031-2

ATGCCGAGTTCACGCTGCACGATATTCAAAGTCGTTGGATCACCGTTGTTTTCACACTGCACGACTATCTGGTGTAACATTCAGTTGCTGCTGTGTTCACATTGCACGATGGATCGGCGACAGGAGGTTACACACTGCATGACTTTACAATTGGAAGAATCGCCAACAACTCTGTCTGCTCCGCAAACTACGTTTCGTGACCAAACGCACGCGAGAAGTGATGAGGAAATAACAGACGATCACGCGTGAGATCAGAGTTTTCACGCGTGACTGGAAATGTTATTAAAATGGTGGCCCGCAAGAAATTCGCAATACAAATAGTATGTGAGCTAATTTGCAGCGAAAGAGCGAGAAAGGAAAAAAAGATGAAAAAAAACAACAACAAGGAACCGAAGCCATGCTTGCTGATATCGTGATCTATAACTCCTCCATAAACGTCCCACTGTCCTGTATCTCGCTCTCTCATTGGCTGTCGGTCATCGTCCATGTATTTTTCAGTCAGAACTCATTTCACACAGCAGGATTTTGAATCGCCGACAAGTCCAGATATTTAGCATGCCAGATATTTCACGGGCCTCGGCGACGCCTTGGCGATTCTCTCAGATCGTGTGAACGAGCACCGATTTGGGACATTTCTCTGCGATTTCGCAAAACGTGTCTGCGAGTGAAAAACGGGGCTAAAATCGTGCAGTGTGAACTCGGCAT

>scaffold_55000031-3

TTAAGCCGGGTTAACACTGTGTGATTTTGGCAGCGATTTGGTGGTCAAATTTTGAAATTCTAAAAGATTCCTATAATCCTACGCTAAAATCTGTTATCTTTGATGGCTAGTTTGACATGTTCACCCACAGCCGATTAATGATCGTCACGAGCAGTTTTTTCCTCCAATGAAATTCTGGCAGTGTAAGAAGATTGCAGACACTTTCCTGCCATGTGACTTCTCCTACGACGACCGTCAAACCAAGAACCAATACGAGCGCCAAACCTGATGACGCAATTAGCGCGACAACTTCAAACCATCTCAGGAAAATGTGGGAAAATGTGGAGTTATGGAGGGAAAAGGAGTGTTTGTATGACGTGTCGTCGCTGCTGTTTGTTTGCTGTTAGCTACCGTTTAGCGAGAGAACGCGGGTTACTGAATGTGTCACGAATTGATGACGTAAAACTCCGGAAAACGAGTCTTCTTGTGTGCGTCCGTTTTTGGCACATCTTGACTGTCGTACAGTCTGACATTATAACAACTGAGATCCTACAGTGTGATTATGTTCATACAGTCTGACAAGCAACAATCACAAAAGACTATTAAACATCACACAGTTTAAGCCCGGCTTAA

>scaffold_55000031-5

GCTTACACTGTGCGATTTTGGCCACGATTTGGTCGTCTGAGAGCAATTTTGAAATCCTAAAAGATTCCTATAATCCTACGCTAAAATCTGTTGTGTTTGATGGCGAGTTTGACGTGTTCACCGACAGATGATTAATGGCCGCTGGGTTCAGTTTTTTCCTCTGATGAAATTCTGGCTGTTGCTGGAGATTTCAGACACTTTCCTGCAGTGTGACTTCAACTACGACGACCGTCAAACCAAGAACCAATCAGAGCGCCGAACCTGATGACGCAATTAGCGCGACAACTTCAAATCACCTTGGGAAAATGTGTAAACGAGTCCGGTGGACAGAACAGCAAGAAGAGAAACTTATTGAGTTATGGAGGGAAAAGGAGTGTTTGTATGACGTGTCGTCGTTGCTAATTGTTTGCTGATAAATACTATTTAGCGAGAGAACGCAGGTTACTGAACGTGTCACGGATTGATGACGTAAAACTCCAGACAAGTCTTCTTGTGTGCGTCTGTTTTTGAAGCGTCTTGACTGTCGTACAGTCTGACATTATGACCACTGAGATCCTACAGTGTGACACGGGGATCATGTTCGTACAGTCTGACAAGCAACAATCACAAAAGACTATTAAACATCGCACAGTGTAAGC

>scaffold_55000032-2

TACACTATCTTGCCAAAAGTTTGTGGACACCTCACCATCAGATCCATACGAGGTTCCTCTCCAAAATGTTGCTACAAATTTGAAGCACACAGTCGTCTAGAATGTTTTTGTATGTTGTAGCGTTACGTTATTCCTTCACTGGAACTAAGAGACTCAAACACTGTTCCAGCATGACAATGCCCCTGTGCACAAAACCCCTGAGCTCTATTAACACATGGTGTGTTCTGACTGGAGTGGAAAACTTGAGTGTTCTGCACAAAGCCCTGACTTTAACACCACTGAACACGTTTAGGATGAACTGGAACGCCGACTGCACCACAGACCTCCTTACCTGACGTTAGTGTCTGATCTCACTAATACTATTGTAGCTGAATGATCACACATCTCACATTCAAGCTCCAAATTGTAGTGGAAAGCTTTACTAGAAGAGTGGAGCTTAATCGAACAGCAAAATGGGGAGAAGATCTGGATTAAGATGTTTAAAATCACATGTGTTTGTGATGGTCAGGTGTCCACAAACTTTTGGCAATATAGTGTA

>scaffold_55000032-5

TTAAAGGGGTCATGAACTGAGAGATCAAAATTCCCTTGATCTCTTGACATATAACAGGTCTTTGTGATATAAAATCATCATCCTGTACGTTTCATAACTCAGAACTGTGTCGTTATTTTAAAAACAGCTTATATTTCAGGCAGGCGGCCAAAATGACGGGTGTTGGAATGTTCTACTCGATGACGTAATAGTGTGGATAAGCCCCGCCTCCACAGAAGAAGATCGACACCTGCTTCAACATCACTGTCTGTTTTGCCCCGCCCACCGATTCAGGCATGCATGTGATAGGTAAAATGCAAAACTGTTAGCCAATCATAGCAGTAGGCGATTACTTCCGAGACAACAATCCGCCACGCCTATTCAAACAGAGCGTTCTGATGAGGGGGTCAAAAACAGGACAGAAAACAGCCTAGTACTTCTAAATTATTATGGTTTTTTTTATGTAAAAATCTTGATAACATTATAAGTGGACCTCAGAGAACAGTACAAAATAAAAAACTGAGGTAGTTCATGGCCCCTTTAA

>scaffold_55000033-3

TAAATACTATTCGCACGGGATGAGTATTATCTGGGGACCTCGTGTAATTTAGAAATCCCCCTCCCATCTGAATTTCGTGTGCGAATTCGCACCGGATAAGCAAAGCCTGTGATTTTACTCGAATTTACTGACTTACCTCCCGGATATGACGTCACAGCATTGGATAGGCTATTGCTTGGATTATTGCTATATCTTGTGGTTATTATAGGTATAGATGTATGATATACATAAACAGAAGAATGTTCCACACCGCATGCCGCTATACATGTAATTTATCTCCATCTAATCATTCTTTGTGTTTTGGCTCTTCTGGTGGTGTTCAGTTTGATCTTTCTGAAAGTTGTATGGTTGTTTGGTGTATATCGACATGCAGCACACAAAATACACCCAAAACACAGCCTCATTCTACACACACAGGAAACAGAAACCTTAAAAAATGATACACGACCCTGCAAATCCTGCTCAAATCACAGAGATGTCGATTCGCACGGGACTAATATTATCACAGGACTTCGGTGTTCAGCTAAATATGGTAGGTCATTTGTAGGGGAATTTTTACTTTACAAATTACAGACATGGCCGATTCACACGGGATTAAGATCACAGACAAACTCCACAATTATTACAAATAACCAGAGGTCCCCAGATAATACTTATCCCGTGCGAATAGGACTTTA

>scaffold_55000034-1

TACGGTGGCCGAGAGAGCTCAACACGCTGCAATTTAAGCAAACCCGTGCAAATGGAAAAAAACACCAACAAATTACGAAAACATCGTCATTAGTTTGACAACACACATGCTGCAAATCCTCCCAATACAACCAAATACAGAAGCAAATGCAATGCAAATAGCACAGACAACAACGGAAATGTTTCAAGGGTTTCCCAAAACAGTGACGAACCTGGCTGGGACCTGTTTATTGTTTCACAGTTAATTGTGTAATTTGTCAGATTATTTAGGCTAATAATATCCAAAAGACCAAGGAATCATATGAACGGGATGATAAAATAAACAGAAAACTTAGATTTCAGTTCCCCGATAACACCACTAACAAACAGCCACCGAACAATAAACGAGTCCCAGCCGGGTTCCTCCCCTTTTGTGGTACATTGAAACATTTCCGTCTGTGCTAGCTGCAACGCGTTTCTGTATTTGGTTGTGTTGTGAGGATTTGCAGCACGTGTGTTGTCAAACTAATGACGATGTTTTCTTAAATTGTTGGTGGTTTTTTTTTTCTATTTGCATGTGTTTACTTAAGTTGCAGCGCGTTGAGCTCTCTCGGCCACCGTA

>scaffold_55000034-3

TTAAAGGTAGGGTAGGGGATTTCAGAGAGGCTAGCAATAGCAAGCTAGCATTGAAAACCAAAGATCCTGCCCTTTCCTCATAATCGCTCTGCAAAGCCACGCGTCCTACAAAACACATGAACGCGCACAGGCATTCTGGAGCCTCAAACGTCAAGATGAAAGCCAGATTACCTCATGTCTGATTCACCAGTGAGTAAACATTACAGTACAAAGTGCCACTTTTAGAGGCAATGTTGGGCTCTTGCGGTTTGCTTTCCATTTTTGCTCAGTCTGCATAGCGGGAACGCGCTAATGACGTATTCTGTCTGCGTGAACAGGGTGCGCAGAGGTATGCAAATACAAATTTGACAGGCAGGTAGGACAGCCTATCGGACCGAACATTTTGATTGGACCAAGTTTTTTTGGTCCTACGCCTTCCACAGAATATATCAATACATATAAATACATGTAGACCACTTAACTTAATGAGTGTTTGTGATGCTGGTTGAAAGTCTCTTCACATCGTGATAGCAATGTTTTTGAAACAAATCACCTACCCTGCCTTTAA

>scaffold_55000034-6

TAGGGCTGCACGATAATGGGAAAATATACAATATTGTTGTTGAGTATTTCAATAACGATATTTCTTGCGATATAATATTTCCTTAGAGAAATGCTATTGTTATTCACCATTTTAGCTGTGAGTGTTTTTCGGGCTGGCTCATCTTAACAGGATTAGCTCGCTATGAAATTAGCTTTGCAGCTTGTTGACTGTCATGCGGCAAAGCACGCGCACGCAAATCCCAACTAACATCCACGTGCCAGATATTTTACTATCCTAAAACTGTGACCACACATTATTATTTGAAGTGTTAAATCGCTCATTTATCGCAAACCATTGCGATATGCACATCTCGATAGGCATATTTGTGATATTTTGATCATTTCGATTTATTGTGCAGCCCTA

>scaffold_55000035-26

TAGGGCTGTGTATCTGCAAGGGCCTCACGATACGATACATATCACGCCACGTGGGTCACGATACAATATATCATGATGTATTGCAGTACAATACATATTGCAATACTTCAAAAGAAACATGTAAAACAAATAGCTAAAAATACCACTTGCTGTGTACCACCTGGAGGACTCTAGTATGTGTATCACATGATATTGTTTACTCAGCAAACAACTAAAAACTGAGGGTTTTAATTAAATATTAACGTGAACATAAAACAGTTTAAAGCGTAATTTAAATATGTATCGATACTAGAGTTTAGAATATCGATACAATACCGTGAAAAAAAATATCGCGATAGTATCGATATTTTTACACAGCCCTA

>scaffold_55000036-1

CACTATACTGCCAAAAGTTTGTGGACACCTGACCATCAGATCCATATGTGGTTCCTCTCCAAAATGTTGCTACAAATTTGGAAGCACACAGTTGTCTAGAATGTTTTTGTATGTTTGTAGCATTACATTATTCCTTCACTGGAACTAAGAGACCCAAACACTGTTCCCGCATGACAATGACCCTGTGCACAAAGCTCCTTTGTTCCATTAAGGAGTTATGATTGGAGTGGAAAAACTTGAGTGTTCTGCACAAAGCCCTGACATTTACACCACTGAACACTTTTGGGATGAACTGGAACGCTGACTGCACCACATACTTCCTTACCTGACCTTAGTGCCTGATCTCACTAATACTATTGTAGCTGAATAAACACACATCTCACATTCAAGCTCCAAATTGTAGTGAAAAGCCTTACTAGAAGAGTGGAGCTTAATCTAACAGCAAAATGGGGAATAAATCTGGAATAAGATGTTTAAAAACACATATGTTTGTAATGGTCAGATGTCCAAAAACTTTTGGCAATATAGTG

>scaffold_55000036-5

TAGGGATGCACCGATACTGTTATCGTTATCGGGCCCGATACTGAGCTCATGGGCTTGTACTCATACTCATAAAAACACTTCGATACCAAAGGCTGATACCTCTTGTAACTTTATTGACAAGTTATTGATAAGTCTCCGGCAGCAGCGACAGAATGTCCGGAAATATTTGAAAATTAATGATGACAATCCACGCATGGCAGACTGCAAACTTTGTTCCGCAATAATATCAAGAGCTACAAAAATGAGTACTTATAATATGAGTAATCTGATAAAACATCTGAAATGAAAACACAAAAGTGAGCACGGCGAATTTGCCGCTAGCAATAACGTTAGCACTCGGCAACCAACCCTGTAGCAAACTCTTGCAAGACGAGAGAAAATGGCAAGAGATAACCCGAGAGCTATACAAATTATCCCACATCCCCAAAAATACAGGTATTGGTATCGGCGAGTTCCAGGAAAAAAATATCGGTATGGTAAAAATGGTATCGGTGCATCCCTA

>scaffold_55000037-5

AGGCTACGTTCACACTGCGAGGCTTAGTGCAAATTTTTTTTTTATAGCTGTTTACATTTTTTGTTTTAATCAATATCAGATTTCCAGTGTGAACTGATCATGGTCCTAAACTGACCCGCATGTGCAAAAGAACAAATTCTACGTCTCATGCAGCGTCCTGTTATACGGAAAAAATAAACACGGAAGACACTGAAGGCAGCATTTACGCGTTAGCTGCTTACATTTATAAGGTGATGTGCAGCAGACGAGAGCAAATTAATGAGCAGTTGTTGAGGAGGAAGAAGATGAGGAGAAAAAGGGGCAAATTTAGAATAATAGCATGTTGTGGAGCAGTGGCGGCTACTTCAGTATGGAGGAGTGTGTGGATGCAGAGCCGGAGCCAGGAGTGGTGGGACCGTGACGTGATCAACGCCTTCAGAGAAATCGATTATATATCAATGGTTTGTAGTTACGCGCCAGTGCAGAATGATTACGCATGTCCATCGAAAATGACATAAAAGTCGCATGAATTCTGACATAACTGTTCACACTGCGGTCGCATTGCAAAACATCTGACCCGTGTCGCATTTAATAGCACATATGGAAATGGCACAAATCGGAATTGAAAAGAACAGATTCCATGCGGTTTGTGCTGTTCACACTGTCATGGGAAAAACAGATCTGTTTGGGCAGATCTCTCATGTGGGCGGAAAATTGGGATTTTGGCCACATTTGCCTACAGTGTGAACGTAGCCT

>scaffold_55000037-19

TTAACCCTTGTGTGGTGTTTGGGTATGAGAGACCCGATTTCAAAGATCGTTTAAAGAGTTATTGAAAAACATTTTTAGCCGCAGACTCATTGGCATTGGCTCATTTTCTATGTAGAACATAAAATAGAACATATTTTCCATAACACTTATATTTTCAAATGTGATCTAACCGGGTAAATGACAAATATTAAGTGAAAATTATGTCTATATTGCTTGTAAGTAAGCTCAAGTAAATATGTTTTAAGTGTTTTTCAATAAATTGTTATAGCTGTGTTGATTAAAAACACAATAATGCGGTGGGTCCACCAGACCTGTGAACACTGGCTTGGTAACACAAACAAACACCACATGAGGTTAA

>scaffold_55000039-1

TTAAGCCGGGTTTACACTGTGCGATTTTGGCCACGATTTGGTCGTCTGAGACAAATTTTGAAATCCTAAAAGATTCCTATAATCCTAGGCTAAAATCTGTTGTGTTTGACGGCTAGTTTGACATGTTCAATAACAGCCGATTAATGGCTGTTACAATAATTTATTTCCTCCGATGAAATTCTGTCAGAGTGAGAAGATTTCAGACACTTTACCTGCAGTGTGACTTCTCATACGACGACCTACAAACCAAGAACCAATAAGAGCACCGAACCTGATGATGCAATTTGCGTGACAACTTCAAACCACGTTGGGGAAAATGTGGAAACGAGTGCGGTGGACAGAACAGCAAGAAGAGAAACAAAATGAACGCTACAGATTGTTTTTGTTTGCTGCTAGCTACCATTCAGCGAGAGAACCCGAGTTACTGAACGTGTCACGGATTGATGACGTAAAACTCCAGACGAATCTTCTTTTTTGCGTCCATTTTTGACAGGTCTTGTAACTGTTGTACAGTCTGACATGATGACAACTGAGATCCAACAGTGTGACGAGCAACAATCGCAAAAGACTATTAAAAATAGTACAGTGTAAGCCCGGCTTAA

>scaffold_55000039-2

TAGGGATGCACCGATACCATTTTTTTAAGACAGAGTACGAGTACCGATATTTTATTTCTGGTACTCACCGATACCTGGACTTTTTTGGGATGTGGGATTATTTGTATGGCCCTCGGGTTTTCTCTTGCTATTTTCTCTCGTCTTGTAAGAGTTTGCTGCAGGGTCGGTTGCTGAGTGCTGCTAGCGGAAAATTCTCCGTGCTCACTTTAGTGTTTTAATTTCAGATGTTTTATCAGATTACTCGCATTACAAGTACTCATTTTTGTACCTCTTGATATTTTTGCGGAATAAAGTTTGCAGTCTGCCATGCGTGGGTTGTCATCATTAATTTTGAGTCTTTGTGCACTGAAAGTTCTGGCAATGACGTCACAAGATATCGGTCTTTGGTATCGGAGTGTTTTTACGAGTATGAGAGCTCAGTATCGGGCCCGATACCGATACCAGTATCGGTGCATCCCTA

>scaffold_55000041-2

TTAACCCTTGTGTGGTGTTTGGGTCTGTGGGACCTGATTTCAAAGTTTGTTTAAAGAGAAATTCTGCTGTTAATTATTTTTTAGCCCAGACTCATTGGCCTTGGCTCATTTTTCCATGTAGAACATAAAATAGAACATATTTTCCATAACACTTATATTTTTTAATGTGATCTAACAGGGTAAATGGCAAATATTAACCATAAATGATGTTTATATTGCTTGTAAGTGAGCTAAACATCTTTTAAGTGTTTTCCATGAATTGTTATAGCTGTGTTGATTAAAAATCCAAATAATGCAGTGGGTCCACCAGACCTGTGAACATTAGGTAACAAAAACATGAACACCACATGAGGGTTAA

>scaffold_55000041-11

GGCTCTGTACGAAATCGCATACTTCCATATTATATAGTATGCAAAACTGAGCATGCGAGACGAGTAGTATGTCCGAATCCTCGTTTGCAAAAAGTCCCCGGATGACCTAATACTACCGCCCAGATTTTGAAGTATGCATACGATGGACACTTTACTATCCCATGAGGCTGCGGGAGAGACGACTCGTCATATGCGGAAGTGGCGGAAAGGCGCAACGCGGCTCTTTACAAATGCTTTAAGTACTTAAGGAAAAAAACATTGTTCCTTGTAGTTAAATTGCACTTGTTTAACATTATTCAAGGTAAACATTATCCAACTTTCAATTTGAGCTGCTGTGACATCTGTATGTTACGCGTCAATATCAACATGACGAATGTATTACGCCCGAATTTCATTCATACTAACCGTAACCATACTATATAGAACATACTTTTTTAACCGTTCGGGAAGTATGTACTTACTTAAATTTAGTACATATTTTAGTATGCTAATTCGGACGGAGCC

>scaffold_55000042-3

TTAGGGTGTGTTCACACTTGTAGTTCGGTTCGTTTGGTTCATTTGGTCCGGACCAAAAATGACAATGATACATTTGGTCCTGGTCCGCTTAGCGTTCACACTGACATTTTTGACAGTGAACCTAAAAATATCGAACATAAAGGCAAAGTCATTTGACCAATCAGGTTTTCAACCTGATTGGTCGGCTTTTGTGACGTATATTTCGCGATGGAACTTACCATCCGAAACGATTCTGTGTACTGGATTAAGTGCGCTCATCGTGTGCATGTGTATTTGGTTTTACTTTTACCAGCTGAGAACTCACGAAGAGCTCCTAAAATGTTCAAAACAGCACCAGGATTTCCTCTGGTGTATGCAGAAACGAAGATCTGCTGCAAGGAGACGGCGTTTTTTCGACGCCTTTTTCGACTATTGGGCAACATAGGTCCTTTGATGAGACGAACCAGGTATGCTTGAGCATTCTGTCCTTTGCAATCAAAGAATCATTTAACTTTTTTCACCATGTGACTTGTGGCGTCGCACCTTGTGACATCACATCCTGTTTTTAGTCCGATTAGATATATTTGGTCGGTGTTGCGTTTATATTTTAATCGAACCGCACCAGAGTTTGTTTGGAAGCGAACCGAGACCCTTCTTTTTAGTGGTCTCGGCCCGCTTGTTTGGTGCACACCATGGTTTGGATTTACACTTACTCAAATGAACCCCACTAACCAATCAATCGCACCAGACTTCTTTTTAATCGAACCAAACATGACAAGTGTGAACACACCCTAA

>scaffold_55000042-5

ATACACTAAATTGCCAAAGTCTGTAGACACAGATCCGTATGTGGTTCTTCTCCAAAATTTTGCTGAAAATTTGGAAGCACACAGTTGTCTAGAATGTCTTTGTATGTTGTAACATTACATTATTCCTTCACTGAAACTAAGAGACCCAAACACTGTTCCAGCATGACAATGCCCCTGTGCACAAAGACATTAAGACTTCCATTAAGACATGGTGTGTTATGATTAGAGTGGAAAAACTTGAGTTTTCTGCACAAAGCCCTGACTTTAACACCACTGAACACCTTTGGGATGAACTGGAACGCCGACTGCACCACAGACCTCCTTACCTGACGTTAGTGTCTGATCTCACTAATACTATTGTATCTGAATGACACATATCTCACATTCAAGTGGAAAACCTTACTAGAAGAGTGGAGCTTAATCGAACAGCAAAATGGGGCGTAAATCTGCAATAAGATGTTTAAAATCACATGATTGTGATGGTCAGGTGTCCACAAACCTTTGGAAATATAGTGTAT

>scaffold_55000042-10

TAGCCCCGTTTACATGTAACATTGTATTCCGATTACAATTAGTTTAAAAGTCCAATCCGAATGAAAATGCTCCATGTAAACATCTCAATCGGAATAAAAATGCCCAACCTGATTAAAATTTCAATCGGGTTGAGAGAGGTGAGATAAACCTTTTTACAACCCAATCAAAGGAAATATTCCTCTATGTAAACGACTCATTCTGGTTACTTTGAGTGTACATCCTGTCGTGCACTGTCGTCCTGTTTTGCGTGTCACGCGAAAGTTTTGTGTCCATTCCAGGTTGCAAAATTTCTGTAGCACAACTCTAACCAGTCCTTACTGTCGACTTTTATTCACAGGATTCTGCTCTTCGGAGGAGGGAGAGGCAGGAAATCGATGGCTCGTAGCGAAGCACTGCTACAGAAAAGCTCCGAATCTTGCGCTCTGTCTTTTCATAAAAGATTTCAGCAACGGCGCGTACTCACGCCCTTAAGAGCAACACAATGCCAATAACTAGTGTGATTAAGCGACGCGGCTCATCTTTACAGGACTGAAAACACTGGAATAATAAAATCCATGAAGTGACAGACGAGCTTTGCGCGTGTCACTAAATTATTCTGACTGAAAGCGTCGGCACATGGAAACACCAGTTCAGAATAGATATCGCTCATATCGTCATGTAAACATTCAATCGGAATATTTCAATCGGAATGATCTCAGTCTGAATGACAAAAAAGTGTGCATGAAAACGGGGCTA

>scaffold_55000043-4

TATATTCCCAAAAGTTTGTGAACACCTGACCATCAGATCCGTATGTGGTTCCTCTCCAAAATGTTGCTACAAATTTGGAAGCACACAGTTGTCTCGAATGACTTTGTATGTTGTAGAATTACAATATTTCTTTGCTGGAACTACGAGACCGATCCAAACACTGTTCCAGCATGACAATGCCCCTGTGCACAAAGACATTAAGACTTCCATTAAGACATGGTGTGTTATGATTAGAGTGGAAAAACTTGAGTGTTCTGCACAAAGCCCTGACTTTAACACCACTGAACACCTTTGGGATGAACTGGAATGCAGAATGCAACACAGGCCTCCTTACCTGATGGCTACTATTGTAGCTAAATGATCACATAATCTCACATTCAACCTCCAAATTGTAGTGGAAAGATGAACTAGAAAAGTGGAGCTTAATATAACAGCAAAATGGGAAGTAAATCTGGAATAAGATGTTTAAACTTACGTGTCCACTAACTTTTTGCAATATA

>scaffold_55000044-1

ATACACTATATTGCCAATGGTTTGTGGACACCTCACCATCAGATCCATACGTGGTTCCTCTCCAAAATGTTGCTACAAATTTGGAAGCACAGTTTTTTTATGTTGTAACATTACATTATTCCTTTACTGGAACTAAGAGACCCTAACACTGTTCCAGCATGACAATGCCCCTGTGCACAAAGACATTAAGACTTCCATTAAGACATGGTGTGTTATGATTGGAGTGGAAAAACTTGAGTGTTCTGCACAAAGCCCTAAACTTAACACCACCGAACACCTTTGGGATGAACTGGAACGCCGCCTGCATCACAGGCCTCCTTACCTGACGTTAGTGCCTGATCTTATTAATACTATTGTAGCTGAATGAACACATATCTCACTTTCAAGCTCTAAATTGTAGTGGAAAACCTTACTAGAAGAGTGGAGCTTAATCTAACAGCAAAATAGGGAGTAAATCTGGAATAAGATGTTTAAAATCACATATGGTTGTAATGGTCAGGTGTCCAAAAGCTTTTGGCAATATAGTATAT

>scaffold_55000046-1

TATATTGGCAAAAGTTTGTGGACACGTGATCATCAGATCCGTATGTGGTTCCTCTCCAAAATGTTGCTACAAATTTGGAAGCACACAGTTGTCTAAAATGTCTTTGTATGTTTTAGCATTACATTATTCCTTCACTGGAACTAAGAGACCCAAACACTGTTCCAGCATGACAATGCCCCTGTGCACAAAGCCCCTGAGTTCCATTAAGACATGGTGTGTTATGATTGGAGTGGAAAAACTTGAGTTTTCTGCACAAAGCCCTGACTTTAACACCACTGAACACCTTTGGGATGAACTGGAACGCCGACTGCACCACAGACCTCCTTACCTGACGTTAGTGCCTGATCTCACTAATACTATTGTAGCTGAATGACACATATCTCACATTCAAGCTCCAAATTGTAGTGGAAAACCTTACTAGAAGAGTGGAGTTGAATCTAACAGCAAAATGTGGAGAAAATCTAGAATAAGATGTTTAAAATCACATATATTTGTGATGGTCAGGTGTCCACAAACTTCTGTCAATATA

>scaffold_55000046-4

CTACGTTCACACTGCGAAGCCTAGTGCTCAATTCAGATTTTTTGCTCAGATCAGATTTTTTTGTATAGCTGTTTACATTGTTGTTTTAAATGTGGCTAATATCAGATTTCCAGTGTGAACTAAATGGTCCTAAACTTACCCGCATGCGCCAAAGAACAAATTATTTGTCTCATGCAGCGTCCTGTCATACGGAAAAATAAACATGGAAGACACTGAAAGCAGAATTTACGCATTAGGTGCTTACATTTATTAAGGTGATGTGCAGCGGACAACAGCAAATTAATGAGCAGTTGTTGAGGAGGAGGAAGAGAATGAGTTGAAAAATGGCCACAGTTTTAATAATAGCATGTTGTGGAGCAGTGGCTGTTGCTTCAGTATGGAGGTGTGTGTGGATGCAGAGCTGGAGCCAGGAGTGGTGGGACCGTGACATGAACGCCTTCAGCGAAATCGATTATATATCAACTGTTTGTAGTTACGCGCCAGTGCAGAATGATGACGCATGTCGATCGAAAATGACATAAAAGTCGCATGAATTCCGATATAACTGTTCACACTGCGGTCGCATTGAATATCTGACCTGTGTCGGATTTAATACCATATATGGAAGTGCCACAAATCGGTATGGAAAAGATCACATTCCATGCGGTTTGTGCTGTTCACACTGTAATGAGAAAAACAGATCTGAGTCACATGTGGGCTAAAAATCGGATTTGGCCACTTTTGCCTGCAGTGTGAACGTAG

>scaffold_55000046-7

TTAACCCTCATGTGGTGTTCATGTTTTTGTTACCTCACCAATGTTCACGGGTCTGGTGGACCCACCACGTTATTGGGTTTTTAATCCACAGCTATAACAATTTATGGAAAAACACTTAAAAGATGTTTACTTTAGCTCACTTACAAGCAATATAGACATTATTTATGGTTAATATTTGCCATTTACCCCATTAGATCACATTTGAAAATATAAGTGTTATGGAAAAGATGTTCTAGTTTATGTTCTACATGGAAAATGAGCCAGGGCCAATGAGTCTGGGGCTAAAACAAAATTAATAGCATAATTTCTCTTTAAACAAACTTTGAAATCAAGTCCCACAAACCCAAACACCACACAAGGGTTAA

>scaffold_55000047-1

AAATACACTAAATTGGCAAAAGTTTGTGGACACCTGACCATCACAAAACACGTGATTTTAAACATCTTATTCCAGATTTTCTCCACATTCTGCTGTTAGATTAAGCTCCACTCTTCTAATAAGGTTTTCCACTACAATTTGGAGCTTGAATGTGAGATATGTGTCATTCAGCTACAATAGTATTAGTGAGATCAGGCACTAATGTCAGGTCAGGAGGTCTGTATTGCAGTCGGCGTTCCAGTTCATCCCAAAGGTGTTCAGTGGTGTTAAAGTCAAGGCTTTATGCAGAATACAAGTTTTTCCGCTCCAATCATAACACACCATGTCCTAATGGAACTCAGGGGCTTTGTGCACAGGGGCATTGTCATGCTAGAACACTGTTTGCGTCTCTTAGTTCCAGTGAACAATACAAAGACATTCTAGACAACTGTGTGCTTCCAAATTTGTAGCAACCTTTTGGAGAGGAACCACACATGGATCTGATGGTGAGGTGTCCACAAATCTTTTGTCAATATAGTGTATTT

>scaffold_55000047-4

TAAAGCCGGGCTTACACAGTGCGATTTTAGCCATGATTTGGTCGTCTGAGACCAATTTTGAAATCCTAAACGTTTCCTATAATCCTACGCAAAAATCTGTTGTCTTTGATGGCTAGTTTGACATGTTCACTGACAGCCAAATAATGGCAGCTGCAATCAGGTTGTTGTTGTTTTTTTGATGAAATTCTGGCAGTGTCAGAAGGTTTCAGACACTTTCCTGCAGTGTGACTTCTCCTACGACGACCAAGAACCAATAGGAACACCGAGCCGGATGACTCAGTTAGCGCGACAACTTCAAACCACCTTGGGAAAATGTTGAAATGAGTCAGGTGGACAGAACAGAAAGAAGAGAAACCTACTGAGGCATCTCATGACCAAAATTAACGCTACAGATTTTTTTACAGATTGTTTTTGTTTGCTGCTAGCTACCATTTAGCAAGAGAACGCGGATTACTGAACGTGTCACGGATTGATGAAGTAAAACTCCGGACGAGTTTTCTGTGTGCATCCGGTTTTGACGCGTCTTGACTGTTGTACAGTCTGACATTATGACAACTGAGTGACTACAGTGTGACAGGGTCACATGTTCGTATAGTCTGACAAGCAACAATCGCAAAAGACTATTAAAAATCGCAAAGTGTGAGCCCGGCTTTA

>scaffold_55000047-6

AGTAGGGCTGTGTAAAAATATCAATACAGCTAACTATCGCGATATTTTTTTTTTACGATAGTGTATCGATATTCTAACCTCTAGTATCGATACATATTTAAATGACGTTTAATAAACTGCTTTACGTTTATGTTAATATTTCATTTAAACCCTCAGTATGTATTTGTCTGCTGAGTAATCAATATGATACACATACTAGAATCCCCCAGGTTGCAACATACGACAGCAAGTTGTATTTTTAGCAATTTTTTTTTACATAATTATTATTTTTTATTAAGTATTGCAATGTATATATCGTGATATATTGTATCGCGACCCATGTATCGTGATATGTATCGTATCGTGAGGTCCTTGCCAATACACAGCCCTACT

>scaffold_55000048-2

ACTATATCGCCAAAAGTTTGTGGACACCTGACCATCACAAAATATGTAATTGTAAACATCTTATTCCAGATTTACTCCCGTTTTGCTGTTAGATTAAGCTCCACTCTTCTAGTAAGGCTTTCCACTACAATTTGGAGCTTGAATGTGAGATATGTGATTATTCAGCTACAACAGTATTAGTGAGATCAGGCACTAACGTCAGGTAAGGAGTTCTGTGGTGAAGTCGGCGTTCCAGTTCATCCCAAAGGTGTTCAGTGGTGTCAAAGTCATGGCTTTGTGCAGAACACAAAGAAGTATTTTCACTCCAATCATAACACAACATGTCTTAATGGAGCTCAGGGGCTTTGTGCACAGGGGCATTGTCATGCTGGAACAGTATTTGGGTCTCTTAGTTCCAGTGTAAGAATACACTGGACAAACATACAAAGACCTTCTAGACAATTGTGTGCTTCAAAATTTGTAGTAACATTTTGGTGAGGAAACACATGTGGAACTGATGGTCAGGTGTTCACAAACGTTTGGCAATATAGT

>scaffold_55000048-4

TAGCCCCGTTTACATGGAGCATTGTATTCCGATTACAATTAGTTTAAAAGTCTAATCCGAATGAAAATGCTTCATGTTAACACCTCAACCAGAATAAAAATACCCAACCCGATTAAAATTTCAATCGGATTAAGAGAAACCTTTTCACAACCCGATTAAATGAAAAATTCCTCCATGTATACAACTCGTTCGGGTTACTTTGTGTGTACGTCCTTCCGCGCATGCCTGGTCTTGACGTAATTTCTCTGGAAATGATGTCAATTCAAAGTTTTGTTTTCATTCCATGTTGCTAAATTTCCGTAGCACAACTCTCACCAGTCCTTACTACGGACTTTCATTCACAGGCTTCTGCTCTTTGGAGGGGGGGCTGGAAATCGATAGCTTGTAGTAAAGCTTGTAAAAAAGCTCGGCTTTTCGCTTTTCATGAAAGATTTCAGAAACAGCGTGTACTCACCCACTCACGTGCTTAAGAGCAACACAATGCCCATTAATAGCATGATTAATCAACTTTACAGGACTGAAAACATGAGAAATAATAAAATTCATGGAGTGACAGACGAGCTGTGCGCATGTCACAAAATTATTCTGATTGAAAGCATTGGCACATGGAAACACCAGTTCGGAATAAATAACGTCTCATGTATGATAATGTCACTCAAACTGAATATTTCAATCAGCATGATCTCAGAGTGGAATGACAAAAAAGTGTGCATGTAAACGGGGCTA

>scaffold_55000048-5

TAACCCTTGTGTGGTGTTTGGGTTTGTGGGACTCGCTTTCAAAGTTTGTTTAAAGAGAAATTATGCTATTAATTATGTTTTTTAGCTTCAGACTCATTGGCCCTGGCTCATTTTCCATGTAGAATATAAAATAGAACATATTTTCCATAACACATATTTTCAATTGTGATCTAACGGGGTAAATGGCAAATATTAACCATAAATAATGTCTATATTGCTTGTAAGTGAGCTAAAGTAAACATCTTTTAATTGTTTTTCCATAAATTGTGATAGCTGTGTGGATTTAAAATCCAATAACGCAATGGGTCCACCAGACCCGGGAACACTGGCTAGGTAAAACGTGAACACCACATAAGGGTTA

>scaffold_55000049-1

TAAAGCCGGGCTTACACTGTGTGATTTTAGCCACGATTTGGTCACCTGAGATAAAGTTTGAAATCCTAAAAGATTCCTTTAATCCTATGCTAAAATATGTTGTCTTTGATGGCTAGTTTGACATGTTCACCACCTGCCGATTATTGGCCATTGGGATCAGTTTTTTCCTCCAATGAACTTCTGTCAGTGTCAGAAGCTTTCAGACACTTTCCTGCAGTGTGACCTCCTACGACGACCGTCAAACCAAGAACCAGTAGGAGCGCCAAACCTGATGACGCAGTTAGCACGACAACTTCAAACCACCTCAGGAACATGTCAAAACGAGTCAGGTGGACAATACAGCAAGAAATGAAACTTATTGAGGCATGTCATGGGCAAATTTACTTTTTTTTTTCAAATTGTTTTTATTTGCTGCTAGCTACCATTTAGCTGAGAATGCAGGTTACTGAACGTGTCACTGATTGATGATGTAATACTTGTCATACAGTCTGAAATGATGACAACTGAGAACCCACAGTGTTACATGGGAATCCCGTTTGTACAGTCTTACAAGCAACAATCACAAAAGACTATTAAAAATCGCACAGTGTGAGCCTGGCTTTA

>scaffold_55000050-1

TATATTACCAAAAGTTTGTGGACACCTGACCATCACAAACATATGTGATTTTAAACATCTTATTCCAGATTTACTTCCCATTTTGCTGATAAATTAAGCTCCACTCTTCTAGTAAGGCTTTCCACTACAATTTGGAGCTTGAATGTGAGATATGTGATCATTCAGCTACAATAGTATTCGTGAGATCAGGCACTAACGTCAGGTAAGGAGGTCTGTGATGCAGTCGGCGTTCCAGTTCATCCCAAAGGTGTTCAGTAGTGTTAAAGTCAGGGCTTTGTGCAGAACACTCAAGTTTTTCCACTCCAATCAAAACACACCATGTCTTAATGGAGCTCAGAGGCTTTGTGCGCAGGGGCATTGTCATGCTGGAACACTGTTTGGGTCTCTTAGTTCCAGTGAAGGAATAATTTACAACATACAAAGACATTTTAGACAACTGTGTGCTTCCAAATTTGTAGCAACATTTTGGAGAGGAACCACATACGGATCTGATGGTCAGGTGTCCACAAACGTTTGGCAATATA

>scaffold_55000051-1

CAGGGCAGGGTAACCCATGTTCCTTAAGTTACTTTGTTCTGCTTGTTTTCCAGCTTCCCCCGTCCTACCCACTACTGATTACCTGGTTCTGGTGTGTTCAGTCAATCAGAAGCTGGAAGATACCATTTCAGATGAGGGGGGAGTGGAGGAAGACCTGCACAATGATTTGGTATCTCCTACCCTGTGATTGAGTGAACACACCTGACCCAGCTAATCAGCAGTGGGTAGGGCAGGGGTAGCTGGAAAACAAGCAGAACAAATGAACTTAAGGAACATGGGTTACCCTGCCCTG

>scaffold_55000052-1

CTATTCGCATGGGATAAATATTATCTGGGGACCTTGTGTGATTTAGTGATTTAGAAATTATCCCCCCCCCCCCATCTGAATTTGGTGTGGCGCATTCGCACATGATAAGCGAAGCCTGTGATTTTCCTTGAATTTATTGACTTGTCTCCCAGATGTGATGTTAACCATTGGATAGGCTATTGCTTGGAAATATCTTGTGGTTCTATAGGTATAGATGTATGATATATCATAAACAGAAGAATGTTCCTTACCGCATGCCGCTATACATTTATCTCCATCTAATCATTGTTTTTGTTTTCGCTCTTCTGGTGGCCTTCCGTTTTATCTTTCTGAAAGTTGTATGGTTGTTTGGTGTATAACGATATGCAGCACCCAAAACACTCCAACGCAGCCTCATTCTTCACACACAGGAAACCGAAACCTTAACAAATGATACACGACCCCACAAATCCTGGCCAAATCATGGAGATGGAGATGTCGATTCGCACGGGAATAATAGTATCACAGGACCTCGGTGTTCTGTGAAATATGGTAGGTCATTAGTGGGGGAATTTTTACTTTACAAATGACAGACATGGCCGATTCGCACGGGATTAAGATCACAAACAACCATATAACAAATAACCAGAGGTGCCCAGATAATACTTATCCCATGCGAATAG

>scaffold_55000052-3

CTTAAGCCGGGCTCATACTGTGTGATTTTTAATAGTCTTCTGCGATTGTTGCTTGTCCGACTGTACGAACATGATCCCCATGTCACACTGTAGGATCTCAGTTGTCATAATGTCAGACTGTCAAGGCGCATCAAAAACGGGCGCACACAAGAACACTCGTCTGGAGTCAATCCGTGACACGTTCAGTAACCCGCGTTCTCTCGCTAAATGGTAGCTAGCAGAAAACAAACAGCAACGACGACACGTCATACAAACACTCCTTTTCCCTCCATAACTCAATAAGTTTCATTTCTTGCTGTTCTGTCCATCGGACTCGTTTCAACATTTTCCCGAGGGGGTTTGAAGTTGTCGCGTTAATTGCGTCATCAGGTTCGGCGCTCCTATTGGTTCTTGGTTTGGCGATCTGCAAAAAAAAAACTGCAGGAAAGTGTCTGAAATCTTCTGACACTGCTTGATATTCATCAGAGGAAAAAACTGATCGTAATGGCCATTAATCGGCTGTCGGTGAACATGTCAAACTAGCCATCAAAGACAACAGATTTTAGCGTAGGATTATAGGAATCTTTTAGGATTTTAAAATTTGTCTCAGACGACCAAATCATGGCCAAAATCGCACAGTGTAAGCCCGGCTTTAAG

>scaffold_55000052-5

TGTACACTATATGGACAAATGTATTGGGACACTTGACCGTCACATCGAACATTATGTTCGTATTGAACATCCTTTTTCAGATTTAGTCTCATTCTTCCTTCTATAATAACCTCCACACTTCTGAAAAGTGTACCAGTGGGGATGTGTTTATTCAGCTACAATAGTATTAGTGAGATCAGATAGTAATGTTGGGGGAGGATTTCTGGGGTGCAGTCAGTGTTCCAGTTTATCCCAAAGGTTTTCAGTGGAGGGCTCTGTGCAAGTTCTTCCACAGCAGCCTTGGCAAACCATGTCTTCATGGAGCTCAGGGGCTTTGTGCACAGGGGTTTTGTCATGCTGAAACAGTGATTGGACCTCTTAGTTCTAGTAAAGGAAAATGTTAATGCTAAAGCTTATAGAGACATGCTAGACAACTGTATGCGTTTAACTTCATGGCAACAGATTAAGGAAGTCACACTTAAGGGTTTGCTGGTAAGATGACCACAAATTTTGGTCCATATAGTGTACA

>scaffold_55000052-10

TAGAGCTGCACAATATATCGTTTCAGCACCGACATCGCAATGTGTGCATCTGCAATAGTCGCATTGCAGGATGTGCAATGTCAAGTCAGGATTATAGTTGACTATGCACCGCATGTGATTGGTGGATGAATTGCTAAGTTTAATAATGAGTGAAAGTTTAGCATTTGCACATTGTTTTAAAGCCCGTTACTGTGTAAACCTTTCAAGCAAGTTTAAATCATTCAGGCGCAAGGAACAATAACTTTTGTAGCTTTAACAAAGATTAATTGTACTTCATTTATACGGTGAAGACTATGCAGTGTGATTTTACATCTGATTATTCAATTTCTGTACCTGAATCCCCATAAAAACATAAAGCACTGTTTATTTCATTTGCATCTTTGTTCTATTGTTGTTATCTTTGTTTATAATTTGTCTATTATATTTTACGCAGATGCACTCCCTTACAAAATCACCCCAATCATTCTAAAATAATGAATTCTTTCCAAATATTATATTATATCTAAGCTATTCAAGTCATCGGGTGAAATTTTTTCACATTGCAATATATATATAGCAGAAAAACAAAATATCGCAATGTCCGTTTTTTCCAATATTGTGCAGCCCTA

>scaffold_55000053-2

TACAGTGGAACCTCGGCATATGAATGCCCTTCCTTACAAATTTTCGCTTTGAAATTTGAAAGAATTGAAATTTTGAACCAAACGAACCAAACCGAACGCGGCTAAGTTGCGCTTACGCCGTTCAAATATTGTTCCACTGTCAGTGGAAAGCCAAGCCAAGCGAGTTTACGAATTTTATGCAGTTTGTTTTCAGTCAGCGAAAACTGTTTTGAAAGAGTCGACCCGTGATCCCAACGAACGGTGCCTTCGGCATGCGTTCAAAAGCTACGTGAATTTTTTGCGCATTGTTTAGTTGACAGATTGGTGGCATAGGGCTATTTATTTCATATTTTACTGAATTGACATGTCTTTTCTAAATGTTTTGATTGTTTTTATATGCAAATATGATTAGAGTGTTTGGAAATTGGTAATAATGGTATTCTTTTTGGGTGGCTGAAATGGATTATCTGCATTTACATTATTTCTTATGGGAAAAGTCGTACCGCAATACAACTTTTCACCTTAAGAAGTTGCCTCCAGAAGGAATTAAATTCGTAGGCCGAGGTTCCACTGTA

>scaffold_55000057-3

AAGCCGGACTTGCACTGTGTGATTTTTAATAGTCCTTTGCGATTGTTGCTTGTCAGACTGTACGAACATGATCCCCAGGTAACACTGTAGGATCACAGTTGTCATAATGTCAGACAGTCAAAACTCGTCAAAAAACAGACGCACACAAGAAGACTCGTCCGGAGTTTTACGTAATTTGTCTGTGACACGTTCAGTAACCTGCGTTCTCTCACTTATCGATAGCTAGCAGCAAACAAAAACAATCTGTAGCGTTAATTGTGTTCATTACATGCCTCTTCTTGCTGTTCTGTCCACTGGACTCGTTTCAATATTTTCCTAAAATGGTTTGTACTTGTTGCGCTAATTGCGTCATCAGGTTCGGCGCTCCTATTGGTTCTTGGTTTGACTGTCATCGTAGGTGAAATCACACTGCTGGAAAGTGTCTGAAATCTTCTAACACTGCCAGAATTTAATCGGAGGAAAAAAACTGATTGCAACGGCCATTAATCAGCTGTTGGTGACTATGTCAAACTAGCGATCAAAGACAACAGATTTTAGCGTAGGATTATAGGAATATTAGGATTTTCTCAATTTGTCTCAGACGACCAAATTGTGGCCAAAATCCCACAGTGTAAACCCGGCTT

>scaffold_55000058-2

TACACTATATTGCCAAAAGTTTGTGGACACCTGACCATCACAATCATATGTGATTTTAAACATCTTATTCCAGATTTACTCCCCATTTTGCTGTTAGATTAAGCTCCACTCTTCTAGTAAGACTTTCCACTACAATTTGGAGCTTGAATGTGAGATATGTGTTCATTCAGCTACAATAGTATTAATGAGATCAGACACTAACATCAGGTAAGGAGGTCTGTGGTGCAGTCGGCATTCCAGTTCATCCCAAAGGTGTTTAGTGGTGTTAAATTCAGGACTTTGTGCAGAACATTCAAGTTTTTCCACTCCAATCACAACACACCATGTCTTAATGGAGCTCAAAGGCTTTGTGCACAGGGGCATTGTCATGCTGGAACAGTGTTTGGGTCTTTTAGTTCCAGTGAAGAAATAATGTAATGCAACATCATACAAAGACATTCAAGATAACTGTGGGCTTCCAATTTTGTAGCAACATTTTGGAGAGGAGGGTGAGGTGTCCACAATGGATCTGGTGGTGAGGTGTCCACAAACTTTTGGCAATATAGTGTA

>scaffold_55000059-4

CCCCGTTTACATGCACTTTTTTGTCATTCCGACTGAAATATTCTGGTTTATTGTTTACATAAGACGTGGTCTATTTCGAACTGGTGTTTCCATGTGCCGATGTTTTCAGTCGGAATATTTTTGTGACATGCGCACAGCTTGTCTGTCACTCCATGAATTAAAATTTTTCCATTGTTTACAATCCTGTAAAGATGGAGATGCATTGCTTAATCGTGCTAGTTATGGGCATTGTGTTGCTCTTAAGCACGCGAGTGTGTGAGTACACGCTGTTCCTAAAATGTTTTAGGAAAAGACGGAGCGTGGAGTGCTTTACTACGAGCCATTGATTTCCGGCCCCTTCCTCCTCCGAAGAACAGAAGACTGTGAATGAAAGTCTGTTTTTAATGACTAGTGAGAGTTGTGTTATGGAAATTTAGCAACCTGGAATGTTAACAAAACTTTCGCGTGACACGTCGTTCATTTAGTGGGAAATTGTCATTATTTTCCGAGAAATTACATCAAGACCAGGCATGTGCAGAAGGACGAACACTCAAAGTAACCGGAATAAGGGGTTTACATGGGGGAATTTTTTTGTTTGGTCAGATTGTGAAAAAGTTAATTCCACCCCTCTCAACCCGATTGAAATTTTAATCGGGTGGGGAATTTGTATTTTGGTTGAGATGTTTCCATAGAGCATTTTCTTTCTGATTGGACTTTTAAACTAGATGTAATCAGAATACAATGCTCCATGTAAACGGGG

>scaffold_55000060-1

TACACTATATTGCCAAAAGTTTGTGGACACCTGACCATCACAAACATATGTGATTTTAAACATCTTATTATACCAGATTTACTCCCCATTTTACTGTTAGATTAAGCTCCACTCTTCTAGAAAGGTTTTCCACTACAATTTGGAGCTTGAATGTGAGATACGTGTTCATTCAGCTACAATAGTATTAACGAGATCAGACACTAACGTCAGGTCAGGAGGTCTGTGGTGCAGTCGGCGTTCCAGTTCATCCCAAAGGTGTTCAGTGGTGTTAGAACTTTGTGCAGAACACTCGAGTTTTTCCACTCCCATCATAACGCACCATGTGTTAATGGAGCTCAGGGGCTTTGTGCACAGGGGCATTGTCATGCTGGAACACTGTTTGGGTCTCTTAGTTCCAGTGAAGGTATAATGTAAAGCTACAACATACAAAGACTTTTTAGACAACTGTGTACTGTCCAAATTTGTAGCAACACTTTGGAGAGAAACCAGATATGGATCTGATCATCAAATGTCCACAAACTTTTGGCAAAATAGTGTA

>scaffold_55000062-6

TAGGGCTGCACAATATATCGAAATATCGCAAATGTGCATAACGAGATAAATGAGTGCAATAAATGAGTGATTTAATACTACAAATAATAACGTGTGGTCAATGTTTTAGGATGGTAAAATATCTGGCACGCGGATGTTAGTCGGGATTTGCGTGCGCGCGCTTTAACGCAAGACAATCAACAAGCTGCGAAGCTTTCATAGCGAGCTAATCTGGATAAGATGAGCCAGCCTGAAAAACACTCGCAGCTAAAATAGCTAATAAAAATAGCATTTTTCTAGGGAAATGTTATATCGCAAGGAAATATTGTTATTGCAATACTCAAGAATATCGCATATTGCATATTTTCCCAGTATCGTGCAGCCCTA

>scaffold_55000063-2

ATACAGGGTGGGTGAAAATGAACTAGGCAATATTTAATGGCTATAGAACTTGTAGTATCACTGGAGTCATGATGAAAACAGACAATAGTAAATAGGATTTCAAGCCTTGCACACTGGAGGACTTGGAGGCACGGATTCGGGAGGTTCTCAGCAATATCCCAAACGACTCCCTTCAGAAGACTGTACATTTCATCTCCGGGTGTTTGAGGACACTGGTTGACGCCACCGGTGCCTACGTTAAAATTTAAAGATGTTTGCTTTCATTTTCCTATGTAATAAAGTACATGTACTTTATTGTATTAGAAATATGGACATTATTACCAATTTTTAATGCCTAATTACTTTTCACCCAACCTGTAT

>scaffold_55000063-5

TTTATGCCGAGTTCACACGATTTTCAGGTCGTTGGATCGCCGTTGTTTTCACACTGCACGACTATCTGGGGTAACATTCAGTTGCTGCTGTGTTCACATTGCATGATGGATGGGCGACAGGAGGTGACGCACTGCATGACTTTACAATAGGAAGAATCGCCAACAACTCTGTCTGCTCCGCAAACTACTTTTCACAACCAAACGCTGGCGAGAAGTGATAAGGTAATAACACGTGAGGTCAGAGTTATTAAAATGGTAGCCTGCAAGAAGTTTGCGATACAAATGGTCTGCGTGCTGATTCACAGTGAAAGAGTGAAAAAGAAAAAAGTCTATAACTGTAGCCATGCTAGCTGATATTATGGTCTATAACTCCTCCCCTAACTTCCTGCTGTCCTGTATCTTGCTCTCTCATTGGCTGTAGGTCATCACCGGTTAATTTTTCACTCCAAACTCATTTCACACCGCAGGAGTTTGAATCGCCGACAAGTCCAAATATTTAGCATGCCAAATATTTCATGGGGCTCGGCGACGCCTCTGCGATTCTCTCTTTGATCATTCACACTGCGCGATTGTCGCTCGCGTGAACGAGCAGCGATTTTTGCCTCCGATTTCGGGCATTGCTCGGCGATTCCGAATAACCTGTCAGTGGGTGAAAAATCGTGCAGTGTGAACTCGGCATTAAA

>scaffold_55000064-7

TTAGGGCTGTGTATTGGCAAGGGCCTCACAATACAATACATATCACGATACATGTGTCACGATACAATAGATCACGATATATTGCAACACAATACATATTGCAATACAAAAAAATAAAAAAAAATATAAATAAATAGCTAACAATACAATTTGCTGTGTACCACCTGGGGGATTCTAGTATGTGTATCACATTGCATTGATTACTCAGCAGACAAATAAACACTGAGGGTTTTAATTAAACATTAGTGTAAATGTAAATCAGTTTAAAAACGTAATTTAAATATGTATCGATACTAGAGGTTAGAATATCGATACACTATGATGAAAAAAAAATATCACAATAGTTAGCTGTATCGATATTTTTACACAGCCCTAA

>scaffold_55000064-12

TTTACACTGTGCGGTTTTGGCCATGATTTGTTTGTCTAAGGCAAATTTAGAAATCCTAAAAGATTCCTAAAATCCTACACTAAAATATGTTGTCTTTGATCACTAGTTTGACATGTTCACCAACATTAATGGCCGTTGCAATCAGTTTTTTCCTCTGATGAAATTCTAGCAGTGTGAGAAGATTTCAGACACTTCCTTGCAGTGTGACTTCTCCTACAACGACCTTCAAACCAAGAACCAATAGGAGCGTTAAACCTGATGACGCAATTAGTGCGACATCTTCAAACCACCTCGATAAAATGTGGAAACGAGTCCGGTGGACAGAACAGTAAGAAGAGAAACTTATTGAGTTATGGAGGGAAAATAAGTGTTTGTATGACGTGTCGTCGCTGCTGTTTGTTTGCTGCCAGCTACCATTTAGCGAGAGAACGCGGGTTACTGAACGTCTCACGGATTGATGACGTAAAACGTCTCCGGACGAGTCTTTTTGTGTGTCTGTTTTTGACGCGTCTTGACTGTCGTACAGTCTGACATTATGACAACTGAGATCCTACAGTGTGACATGGGGATCATGTTCGTACAGTCTGACAAACAATAATCGCAAAAGACTATTAAAAATTGCACAGTGTAAA

>scaffold_55000069-8

TAGCTATGTTTCCGTCCAAAGATGCGAATTAAACTTGTGCGCAGAACTGGAATATTGCATAAAACATTAGCGAATAAAGCACCAGTTCCATCCAACGAATCAAAGAGAACAAAATCATCACTGCTTGATAAACTGGAGCCAAATATCAAAAAGATAAATGGAAGTTGCTGCAGTAGGAGAAGCCACTGTGGGCTTTTTTTCAATAAATTACTTGCGTCTCAGAACACGTGGACAAAGCGGAATGAACGCAGTCATGTCATCTTGCTTTCGGAGGCGGGAGACTACTCTTTGGGAACGCAGCCGGTCAAGACAGTTCTGGGAGGTAATAATACCAAACCACTTTAATGACAGACTTTGGCTAAAGCATTTTTGAATGACCAAAACAACATTTCAGATGTTGTGTAATGAGATCGTCCGCTGGATAGTCCATTAATGCCGTCCCATAGCGCCCCAGTTTCAACAGAGAAACGCATTGCTATTGCGCTGTATAAATTGGCCATTTTTGTTATCACATGATCTGTTGAAGCCAAACTACATGACTTTTTTGATGCGCATGCTGGAATTTATTCGTTGAATGGGTTTTCATCGTAGTCATTTTTTTTTTTGGATAAAATGTTTATCCTACTCATTTGTGCGCATAAGTTTTTTATGCGCATCTTGGCGTTTCCATCCAGTGTTTTTTTATGCGATAGTCCAAAATGCACATAAAAATAGGTGGATGGAAACATGGCTA

>scaffold_55000081-1

ATACGAGGGAGTATCAAGTTTTGAGATACACCCCCAGATGGCAGCACAACTTGCATGCAGCCTTGTGCTGCCATCTCTTGTTGTATCTCAAAACATTTTGATACCCCCTCGTAT

>scaffold_55000083-6

TCAGTGTTCCCAACTTAGCGACTTTGTCGCTAGATCTAGCGACTTTTCAGACCCCTTTACCGACTTTTTTTTTTTTTTAAGCGCCTAGTGACTTTTGGACAAACCTTAGGAAGTTTCCAAATGTCGCCAGTACTGTGCTGCGAGTGCGAGGTGTTGCTTTCCCGGTACGGTTACACCTCTCCCTGTGCCTACTCTGATCAGTGAGTGCTAGCTCCGGCTGAGAGGAGCAGGACATTCCATTGACCGCACGAATGCAAATACGACATAATTACGTCATTAATATGCAAATTTGTGCATGACGTCATTTAGCAACATTTGGCGACTTTTCTAGCAGACTTTAGCTACTTTTCATTGAAAATAGTTGGCAACACTGA

>scaffold_55000086-1

CAGGGCTCGCAAAATGTTTCAATTCCTGGTAGCTCTTCGGGCAGGTACTCTTCAGTTTTTGGTAGCCCAAAATGAATTTAAGTAGCTTGAACAAAAACAACATTATTTTTAATGTAGAATATTGTAAAGGAGACATCAATCAAAAACATTTTTAAAACACAAATTACAATAGTATAAAATAATCAACTTACAATCATCAATACAAATATTTGGTTCTAACTGAACATCATATTCCAGATGTCAGCGATTTTCACTATTCTCTCCACTGCGCATGAATGTATTTCTATCACATGTTTAAGCTTGCTGCGCTAGATTTCACTTGTCACGTGGGGATAGTAATGACTGACAGGAGGAATTGGTGTGCAACAAATCCGAAGCTTCACTGACAAATATTAGTAATACTGCCGCTCCCTATACACAGTTTCCAAATTTAAAGTGAATATTTTCAATACCCCACATATTGATAGTGGCTCCCCAGATACCCGAAAATTACAGACAACATAATTGCCCAACTATATTTGTGAGACAATGCAAAAACATTGAAATAATTTTTCCTCTTTATAAGATAGCCCGACAGGCAGGGCGGGGATACATTTTGGTAGCCCGACTGTAAGACACAATATCCCCGGAACGTCGGGCTAGCGATTTTGCGAGCCCTG

>scaffold_55000088-1

CAGGGCTCGCAAAATCGCTAGCCCGACGTTCCGGGGATATTGTGTCTTACAGTCGGGCTACCAAAATGTATCCCCGCCCTGCCTGTCGGGCTATCTTATAAAGAGGAAAAATTATTTCAATGTTTTTGCATTGTCTCACAAATATAGTTGGGCAATTATGTTGTCTGTAATTTTCGGGTATCTGGGGAGCCACTATCAATATGTGGGGTATTGAAAATATTCACTTTAAATTTGGAAACTGTGTATAGGGAGCGGCAGTATTACTAATATTTGTCAGTGAAGCTTCGGATTTGTTGCACACCAATTCCTCCTGTCAGTCATTACTATCCCCACGTGACAAGTGAAATCTAGCGCAGCAAGCTTAAACATGTGATAGAAATACATTCATGCGCAGTGGAGAGAATAGTGAAAATCGCTGACATCTGGAATATGATGTTCAGTTAGAACCAAATATTTGTATTGATGATTGTAAGTTGATTATTTTATACTATTGTAATTTGTGTTTTAAAAATGTTTTTGATTGATGTCTCCTTTACAATATTCTACATTAAAAATAATGTTGTTTTTGTTCAAGCTACTTAAATTCATTTTGGGCTACCAAAAACTGAAGAGTACCTGCCCGAAGAGCTACCAGGAATTGAAACATTTTGCGAGCCCTG

>scaffold_55000088-2

TTAAGTCGAGCTTACACTGTGCGAATTGGGCCACCATTTGGTCGTCTGAGACAAATATTGAAATCCTAAAAGATTCCTATAATCCTACGCTAAAACCGGTTGTCTTTGACGGCTAGTTTAACATGTTCACCGACAGCCGTTTACCGGCCGATGCGATCAGTTTATTCCTCCGTTGAAATTCTGGCAGTGTTGGAACATTTCAGACGCTTTCCTGCAGTGTCACGTCTCCTACGACGATCGTCAAACCAAGAACCAATAGGAGCGCTGAACCTGATGACGCAATTAGCGCCACAACTACAAACCACATCAGGAACATGTGGAAACGAGTCGGTGGACAGAACAGCAAGAAAAGAAACTTATTGAGGCATGTCATGAGCAAAATTAATGCTACAGGTGTTTTTGTTTGCTGCTAGCTAGCATTTAGCGAGAGAACGCAGGTTACTGAACGTGTCACGGATTGATGAGGTAAAACTCGAGTTTTCTTGTGTGCGTCCGTTTTTGACGCGTCTTGACTGTCGTACAATCTGACCTCATGACAGCTGAGATCCTACAGTGTGACACGGAGATCATGTTCGTACAGCCTGACAAGCCACAATCGCAAAAGACTATTAAAAAGCGCACAGTGTAAACCCGGCTTAA

>scaffold_55000089-9

TTACACTGTGCGAATTGGGCCACCATTTGGTCGTCTGAGACAAATATTGAAATCCTAAAAGATTCCTATAATCCTACGCTAAAACCGGTTGTCTTTGACGGCTAGTTTAACATGTTCACCGACAGCCGTTTACCGGCCGATGCGATCAGTTTATTCCTCCGTTGAAATTTTGGGCAGTGTTGGAACATTTCAGACGCTTTCCTGCAGTGTCACGTCTCCTACGACGATCGTCAAACCAAGAACCAATAGGAGCGCTGAACCTGATGACGCAATTAGCGCCACAACTACAAACCACATCAGGAACATGTGGAAACGAGTCGGTGGACAGAACAGCAAGAAAAGAAACTTATTGAGTTCTATAGAGAAAAGAAGTGTTTGTATGACGTGTCGTCACTGTTTGTTTGCTGCTAGCTAGCATTTAGCGAGAGAACGCAGGTTACTGAACGTGTCACGGATTGATGAGGTAAAAATCTGGATGAGTTTTCTTGTGTGCGTCCGTTTTTGACGCGTCTTGACTGTCGTACAATCTGACCTCATGACAGCTGAGATCCTACAGTGTGACACGGAGATCATGTTCGTACAGCCTGACAAGCAACAATCGCAAAAGACTATTAAAAATCGCACAGTGTAA

>scaffold_55000092-18

AGTAGGGCTGTGTATTGGCAAGGCCCTCACAATATGATACATATCACAATACATGGGTCATGATACAATACATCACGATATATTGCAATACTTAAAAAATGTTTAAATAAAAAGAAAACTTGCTGTGTAACACCTGGGGGATTCTAGTATGTGAATCACATTATTTTGATTACTCAGCAGAAAAATAAAAATAATCAGTTTAATTCAATAAAAACTGACGGTTTAATTAAATACTAACATAACCGTAACTCAGTTTAAAATTTAATTTAAATATGTATCAATGCTAGAGGTTAGAATATCGATACACTCGTGAACATTTTTTTTTTACTTAGCTGTATCAATATTTTTACACAGTCCTACT

>scaffold_55000093-1

AGGGTGTTTTCACACCTAGTTCTTTTGAGCCCTCCAAACAAGCCCTCTCGGAGCGGATCAGCGTGTATATGTGAACAAACCAAGTGATCACAGACCCTGCTAAAAATACCCAAAAGCGAACTGTACTGTGTTCGTTTGTGGGTCCGTTTGTGGTTCGATATCTTCGTGATATGTGAAAGCAATGAGATTTCAGTCTGCTTTGCGTTTCGTTTCAAATGTGTACCGGGAGGCTTGCCATACCTGCAACCATGTTACTGCTGTAACTTTTCACCATGGCAGGTCGCGGGGTTGTGTGGTCTCAGGAGGAGAGCTGTGCACTTATTGTAATTTGGAAAGAGGATTACATTAAACACCAACTATCCTCAACACATAAAAACATAAAAGTGTTTGCTTTTGTTTGCTCAAAAAATTATGGAAAGGAATATAACAGAGCCGCGCTACAATGCTGCGCTAAAGTAAAAAAACTTTGCCAGAAGTACATGAGCACCCATGACAAAATTCGCCAAAATGCACAGAAAGCTGGGGTCTGAGTTCTTATGAAGTTGTGGTGTGAACAAGAAAGGGTCTGAGATCACTTCATTGCTGAAGAGGACCAAAAAATGGACTGGGTTCTCTTTTGTGACCACTATATTGTGAACACCAAAAGGACTGAGATCACTTTTGGCTGGTTCCCTTTAGGTGAACTGGGTTTGGTTATTTTAAAACGAACTATATGTGAAAACACCCT

>scaffold_55000093-3

ATACAGACAACCCTTGACTTTCGAATGGGGTTACCTTCCCCAAAACCCATTGGACGTCGAAATATCATGTCGATATAGGTATACCCCTATGATGCGTTCTGAAGTAGCCCACGGTAGCTTGTAAGAGTAGCCGCAGGTGGCTATAGATACCTGTTCCAATAATATCACACTGTTGTCACGTTTTCTTTAGCCATGCGAAATTTGAAAAATATCTTTCAACATACTTTTTGGGTCACGCAGTCGTATTGTAACTCCAAAAAATCGTAAGTTGATATATTGTAAGCCGAGGGTTGTCTGTAT

>scaffold_55000093-10

TAGGGCTGTGTAAAAATATTGATACAGCTAACTATCACAATAATTTTTTTCACGATAGTGTATCTATTTTCTAATCTTTAGTATCGATACATATTTAAATTACGTTTTAAACTGATTTACGTTAGTCAGTTTTTATTTGTCTGCTGAGTAATCAATATAATGTGATGCACATACTAGAATCCCTCAGGTGGTACACAGCAAGTTGTATTTTTATCTATTTGTTTTCCTAATTTTTTTTTTTAAGTATTGCAATGTATTGCAATATGTATTGTATTGCAATATATTGTGATATATTGTATCGTGACCCATGTATCGTAATATGTATCGTATCGTGAGGCCCTTGCCAATACACAGCCCTA

>scaffold_55000098-25

TAGGGCTGTGTAAAAAAAAATCGATACAGCGAACTATCGCAATATTTTTTTCTTCATGGTAGTGTATCGATATTCAAACTTTTAGCATCGATATATATTTAAATTACGTTTTAACCTGATTGTGTTTACGTTAATATTTAATTAAAACCCTCAGTTTTTAATTGTCTGCTGAGTAATTAATATAATGTGATACACATACTAGAATCCCCCAGGTGCTACACAGCAAGTTTTATTTTCAGCCATTTGTTTTACATTTTTTTATTTTTAAGTATTGCATTGTATTGCAATATATCGTGATATGTTGTATCGTGACCCATTTATCATGATCTGTATCGTATCGTGAGGCCCTTGCCAAAACACAGCCCTA

>scaffold_550000102-5

ACTAGGGCTGTGTTTTGGCAAGGGCCTCACGATACAATATATCACGATATATTGCAATACAATACATATTGCAATACTTAAAAATAAAAGTAAAACAAATGGCTAAAAATACAACTTGCTGAGGGATTCGTGTATGTGTATCACGTTATATTAATTACTCAGCAGACAAATAAAAACTGAGGGTTTTAATTAAATATTAACGTAAATGTAAATCAGTTTAAAACATAATTTAAATATGTATCGATACTAGAGTTTAGAATATCGATACACTATCGTGAAAAAAATTGCGATAGTTAGCTGTATCGCAAATTTTTACACAGCCCTAGT

>scaffold_550000113-2

GTACATTAGGGCTGCACAATATATTGAAATTATCTAAATATCGCAAATGTGCAGATCAAGATATGCATATCGCATTGGTTTGCGATCAATGAGCGATTTAATACTTCAAATAGTAATGTGTGGTCAAAGTTTTAGGATAGTAAAATATCTGGCACGCGGATGTTAGTTGGGATAAGCGTGCGCGTGCTTTGACGCAAGACAATCAACAAGCTGCAAAGCTTTCTTAGTGAGCTAATCTTGATAAGATGAGCCAGCCTGAAAAACAACTGCAGCAAAAATAATTAATGAAAATAGCATTTCTGTAGGGAAATGTTATAATGCAAGAAATATCATTATCGCAATGCTCCACAACAATATTGCATATCTCATATTTTCCGAGTATCGTGCAGCCCTAGTGTAC

>scaffold_550000119-2

TACAGTAGGACCTCGATTCACGAACACATTAACCCACGAACAAATTGATTCACGAACAAAAAAATATATAAAAATGCTGTTTGATTCATGAACTCATTTTTGAAACACGAACATGGTCCGATGGCCAAAATTTGAAAAATGCGGGCAATCGCTCATCTTACGTGGGTGCATCCGAGCCGTTATGGTGGCCGCCATCTTGTTATTGTAGGGTATTGTACATCTCCCACGCACTTCCACCCACGCTTAGTCTGTTTTTGATTTCGGTCTTGAGTAGACGCACGTTCAAACGCTTTAAACTGCCACCCACCCTCCTCTTCTCTCGTCCGTCAAGCCAGAAGTCTTGTCAGCCAACGTAAGTGTTCACTTTTTAAATGTTTTTTTAGTTTTAGTTTACTATTATAAATTATATTATCAGATATATTAAGGTTTTTCACACTTTGTTTAATATTATCTGTGTAAAATAAAACATAAAATACGTAATTTTTTAATGGTTCGTTAGGGTCATGAACAAATTAATCGTATTCCCATTAAACCTTATGGGAAAATTAACTTTGGGTCACGAACATATTGGAACATGAACAAAGTTTAGGAACGAATTGTGTTCGTGAATCAAGGTCCTACTGTA

>scaffold_550000123-4

TATACTGTAGGACCTCGATTCACAAACACATTAACCCACGAACAAATTGATTCACGAACAAAAATATATATAAAAATGCTGTTTGATTCACGAACTCATTTTTGAAACACGAACATGGTCCGATGGCCAAAATGTGAAAAATGCGGCCAATCGCTCATCTCACGTGGATGCATCTGAGCCGCTATGGGGGCCGCCATTTTGTTATTGTAGGGTACTGTACACCACCCACGCACTTTTACCCACGCTTAGTCTGTTTTTGATTTCAGTCTTGAGTAGACGCGCGTTCATTCTCCTTCCCTCTACGCAATAATCTCCTCCCCACTCGTCCGTCAAGCCGGAAGTCGTGTCAGCCAACGTAAGTGTTCACTTTTTAAATGTTTTTTTTAGTTTTAGTTTACTATTCTAAATTATATTATTAGATATATTAAGGTTTATCACACTTTGTTTAATATTATCTGTGTAAAATAAAACATAAAATACGTAATTTATAATGGTTCGTAGGGGTCATGAACAAATTAATCGTATTCCCATTAAACCTTATGGGGAAATTAACTTTGGGTCACGAACATATTGGAACACGAACAAAGTTTAGGAACGAATTGTGTTCGTGAATCGAGGTCCTACTGTATA

>scaffold_550000123-5

ATTTACACTGCAGGTCTTGATGCCCAATTCCGATTTTGTGACTATATCCGAATATTTTGATGGCCCACTTACATCTTTTAAAACTGACCCATATCCGATATCTGCATTTACACTATACACAGCAAAACAACCCAGGTAGACGTACTGACCGAAAAAAGAAATTAAAACGGTGCGATAATTAATATACGCAAGATGAAATGATTATTCTATATTTTGCTTTTGCACCATATTTAAAGGTTGGCATAGTTCTCGGAACCTGGGGTCAGCAATTTCTGTATGAGACGAGAAACATTTGATATGTTGTCAAGAGGTTGCTGTTGGTGTCCACCTGAGTTGCCGTCATGCTCCACCGTTGCTTATTCAATGACGTACGACTTGCATTGACGGGTGAAAATTCGATCTGGCTTCTTACATGGCAGACGCTAATGCACGTATCCAATTCATATCAGATTTATTTCCACATATGAATGAGGCCTGAAACCAATCTGAAAAAATCGGAATCCATGTGCTTTTTTCCTGCTTACACGATCGTGAGTCATATCCGATCTGTGCCACATTGGAGGAAAAAATCGGAATTGGGTCACTTGATACATGCAGTGTAAAT

>scaffold_550000124-7

TGCAGGTTTTTTATTTCATTCAAGCAAGAGACGCACCTGATTCCACCTGTTTAATCACTTAATCTTTGATTTCAGTAGACTCAGGTGTGGCTTTTGCATGGTTGGAATAAAAATCTGCA

>scaffold_550000134-12

AGGCTCCATCCGAAATCGCATACTACTTAAGTAGGTACTACATTTGAGTGAGTAGGCACTTCCTAACCGTTAAATAAGTACGTTCTATATAGTATGATTATTGGGGAGTATGAATGAAATTGGGACGTACTACATCCGTTGATATGGTCACATGACATACGACGTCACAACAGCGTGAATTTAAAGTGGGATAATGTTGAAGTTGGATGATGATAAACGAGTGTAATTTAACTACAAGGAACAATGTTTTTATCCTGACGTATTTAAAGCTACTTTAAGATCTTGTAAAGTGCCGCGTCGCCGCTTTCAGCCATTTTGGAATATGACGAGTCGTCTCTCCCGCGGGATCATGGGATAGTAAAGTGTCCATCGTATGTGTAGTTCAGAATCCGAGCGGAAGTAGTAGATCATCCGGGAACTTTTCACGTAATGTTTCTCGCATACTAAGGATTTTGACGCATACTCACATACTCGTTTTTTGCTACGATATATTATGGAAGTATGCGATTTCGTACGAAGCCT

>scaffold_550000145-9

TAGGGCTGCACGATACTGGCAAAATATGCGATATGCGATATTGTTGAGTATCGCGATAACGATATTTCTTGCGATATAATATTTCCCAAGAGAAATGCAATTTTTAGTAGCTATTTTAGCTGCAGGTGTTTTTCGGGCTGGTTCGTCTCATCAGGATTATCTCGCTATGAAACCTTCGCAGTTTGTTGATCGTCTTGCGTCAAATCACGCGCACGCAAATCCCAACTAACATCCGCGTACCAGATATTTTACTATCCTAAAACTTTGACCACACATTATTATTTGAAATATTAAATCGCTAATTTATCGCAAACCATTATGATACGCACATCTCCATATGCACATTTACGATATTTTGATCATTTTTATATATTGTGCAGCCCTA

>scaffold_550000147-2

CTAGGGCTGCACGATACTGGGAAAATAGGCGATATTGTTGTTGAGTATTGCGATAACGATATTTCTTGCGATATAACATTTCCCCTAGAGAAATGCTATTTTTAGTAGCTATTTTAGCTGCGGGTGTTTTTCCGGCTGGTTCGTCTTATCAGGATTAGCTCGCTATGAAAGCTTCGCTTGCTTGTTGATCGTCTTGCGTCAAATCACGCGCACGCAAATCCCAACTAACATCCGCGCGCCAGATATTTTACTATCCTAAAACTTTCACCACACGTTAATATTTGAAGTATTAAATCACTCATTTATCGCAAACCATTACCATACGCACATCTCGATGTGCACATTTGCGATATTTTTGATAATTTCGATTTATTGTGCAGCCCTAG

>scaffold_550000147-10

ACTAGGGCTGTGTAAAATAATCGGTACAGCTAACTATCGTGATATTTTTTTCACGATAGTGTATCGATATTCTAACCTCTAGTATCGAAACATATTTCAAATTACGTTTTAAACCGATTGACGTTTACGTTAGTATTTAATTCAAAGTTTTTATTTTTCTGCTGAGTAATCAATATAAATGTGATACACATACTAGAATCCCCCAGGTGCTACACAGCAAGTATTGCAATGTATTGCATTATGTATTGTATTGCAATATATCGTCATATATTGTATCGTGAGCCATGTATTGTGATATGTATCGTATCGTGACCCCCTTGCCAATACACAGCCTTAGT

>scaffold_550000150-1

CTATTCGCACTGGATTAGTTTTATCTGGGGACCTCGTGTGATTTAGAAATTACCCCCCCATCTGAATTTCATGTGGCGCATTCGCACGGGATAAGCGAAGCCTGTGATTTCACTTGAATTGGATACAGCATTAGATATAGCTATTGTTAGGAAATATCATGTGGTTCTATCGGTATAGATGTATGATATATCATAAACAGAAGAATGTACCTTACCGCATGCAGCTATACATGTCATTTATCTCCATCTAATCATTCTTTTAGTTTTCACTCTTCTGGTGGCCTTCAGTTTGATCTTTCTGAAAGTTGTATGGTTGTTTGGTGTATAGCGATTATGCAGCACCCAAAATACACCCAAACACAGCCTCATTCTTCACACACAGGAAACAGAAACCTTAAAAAATGATACACGACCCCACAAATCCTGACCAAATCACAGAGATGTCGATTCACACAATATAGGACTAATATCACATGACCTCTGTGTTCGGCGAAATATGGTAGAGCATTTACGGGTAATTTTTACTTTACAAATTACAGACATGGCCGATTCACGCGAGATGAAGACCATAGACAACCTCTACAATTATTACAAATAACCAGAGGTCATCAGATAATACTAATCCTGTACGAATAG

>scaffold_550000153-1

ACTTAGGCTCTGTCCGAAATCGCATACCTCCATACTATATAGTATACGAAACTGCGAGGCGAGTAGTATGTCCGAATCCTTAGTATGCGAAATACAGTACGCAGAAAGTTCCCCGGATGACCTACTACTCCCGCCCGGACGGATTTTGAATACGATGGACACTTTACTATCCCATGAGGCCGCGGGAGAGACGACGCGTCATATGCGGAAGTGGCGGAAAGCGGCGACGCTGCACTTTACAAGTGCTTAAAGTACTTAAAGAAAACAACATTATTCATTGTAGTTAATTTGCACTCGTTTAACATCATTCAAGTTAAACATCAACCAACTTTAAATTAGCGCTGCTGTGACGTCGTATGTCACGTGACAATATCAACATGGCGGATGTAGTATGTCCAAAATTCATTCATACTAGCTGTAACTAAATAGAACGTACTTGTTTAACGGTCAGGAAGTGCGTACTTAAATGTAGTACCTACTTAAGTAGTATGCGATTTCGGACCGAGCCTCAGT

>scaffold_550000154-8

TAGGGCTGCACAATATATCGAAATATCGCAAATGTGCACATCGAGATATGCGTATGGTAATGGTTTGCGATAAATGAGCGATTTAATACTTCAAATAATAATGTGTGGTCAAAGTTTTAGGATAGTAAAATATCTGGCGCGCGGAATGTTAGCTGGGATTTGCGTGCGCGTGATTTGACGCAAGACGATCAACAAACTGCGAAGCTTTCATAGCGAGCTAATCTTGATAAGACGAACCAGCCCGAAAAAAACACGCAGCTAAAATAGCTAATAAAAATAGCATTTCTCTAGGGAAATGTTATATCGCAAGAAATATCGTTATCTCGATACTCAACAATAATATCGCATATTTTCCCAGTATCGTGCAGCCCTA

>scaffold_550000156-2

ATGCACCGATACCATTTTTTACAGACCGAGTACCGATCCTTTTTTTTTCTTTCTGGTACTCGCCGATATCGATACCGATACCTGTACTTTTTTTAAGACGTGGCATTATTTGTATGGCTCTCGGTGTTTTCTCTTGCCATTTTCTCTCGTCTTGCACGGGGTTTGCTGCAGGGTCGGTTGCCGAGTGCCAACGTTACTGCTAGCTGCAAATTCTCCGTGCTCACTTTCGTGTTTTAATTTCAGATGTTTTATCAGATTACTGAACAGAAGTACTCCTTTTTGTACCTTTTGATATTTTTGCGGAACAAAGTTTGCAGTCTGCCTGCCATGCGTGGGTCGTCGTCATTAATTTAGAAATATTTCCACACCGCCGAGTCTGACATTCTGTCGCTGCTGCCGGAGTCTTTGTGCACTGTAATTTCTGTCAATGACGTCACAAGAGGGTATCGGAGTGTTTTTACGAGTACATGCGCTCCGTATCGGGCCCGATTCCGATACCAGTATCGTATCGGTGCAT

>scaffold_550000160-2

CTATTCGCACGGGACTAGTATTATCTGGGGACCTAGTGTGATTTAGAAATGACATCTGAATTTCGTGACGGGATAAGCGAAGCCTGTGATTTTACTTGAATTTACTGACTTCTCTCCCGGATATGACGTCAAAGCATTAGACAGGCTATTGTTTGTAAATATCTTGTGGTTCTAAAGGTATAGATGTACGATACATCATAAACAGAAGAATGTTCCTTACCGTATGCCGCTATACATGTAATTTATCTCCATCTAATCGTTCGTTTTGTTTTTGCTCTTCGGGTGGCGTTCAGTTTTATCTTTCTGAAAGTTGTATGGTGGTTTGGTGTATAGCGATTATGCAGCACCCAAAATACACCAAAACACTCCAACGCAGCCTCTTTCTTCACGAATAGGAATCAGAAACCTTAAAAACATGCTACACGACCCCGCAAATTCTGGCCAAATCACAGAGATATCGATTCGTACGGGACTAATATTATCACAGGACCTCGGCGAAATATGGTGGGACATTTGCAGGGGAATTTTTACAGACATGGCCGATTCGTATGGGATCAAGATCACAGACAAGCGCCGCAATTATTACCAAATAACCAGCGGTACCCAGTTAATACCAATCCCGTGCGAATAG

>scaffold_550000160-3

ACTTATGCTGCGTTCCCACCAAACGCGAAGCGAATACGCGCATCGCGTCACTCGCTTTAGATCCGTCGCGCAATGTTTTATTTGTAAAACATTCATTTTGGGGTGCCCGCAGACGGTTACAATAATGCTCTCCTCCATTGACTGATACAACGGACGTGTCAATAAGCTCTTCGCTAATTGAATTGCGCGACAACGCAGCATGCGGGTTTTCCGCTTAAGTCGAATTTTTCAATTTGCGCAAAAGAAAAAGCGTTTGAGGCGAAAATCGCACGTTCGCAAACAAACATTTCGCGTGCATTTCGCATTCCGACGTGAATTCGCCTCTATCCGCGTCTTTGCGTTGAATTTGTATGTAATCTACTTGCGTGAATAATTACATTTGCGTTTGGTGTGAACACAGCATTAGT

>scaffold_550000161-6

TAGGGCTGCACAATATATCCAAATATGGTTTGCGATAAATGAGCGATTTAATACTTCAAATATTAACGTGTGGTCAAAGTTTTAGGATGGTAAAATATCTGGCGCGCGGATGTTAGTTGGGATTTGCGTGCGCGTGATTTGACGCAAAACGATCAACAAACTGCAAAGCTTTCATAGCGAGCTAATCTTGATAAGACGAACCAGCCTGAAAAACACCCGCAGCTAAAATAGCTAATAAAAATAGCCTTTCTCTAGGGAAATTTTATATCGCAAGAAATATCGTTATCGCGATACTCAACAACAATATCGCATATCTTCCCAGTATCGTGCAGCCCTA

>scaffold_550000161-9

TAGGGCTGTGTATTGGCAAGGGCCTCACGATACGATACATATCACGATACATGCAATACAATACATATTAAAAGTAAGACAAAAAAAGTTAACAAAAATAGCTAAAAACACAACTTGCTGTGTGCCACCTGGGGGATTCTAGTGTGTGTATCACATGATATTGATTACTCAGCAGACAAAAACACTGAGGGTTTAAATGAAATATTAACGTAAATCAGTTTAAAACAATTTAAATAGGTTTCGATACTAGGGGTTAGAATATCGATACACTATCGTGAAAAAAATATCGCGATAGTTAGCTTTATGGATATTTTTACACAGCCCTA

>scaffold_550000165-3

AGGCCCGGTCCCAATACCCGCACTTGCACCCATGTGCCCCTCATTTGCGTGTTCCCGCTGAAGGGTGCAAGGGGGGTAGGGTGTCCCAATTCGCTGGTGAGCTTCTCCATCCCCGCCTACTTGTGCCCTTAATGCACACTTTCTGTAAGCACGGGTCCAAGCGGACTTCGCTTAAAGAATTCATCTGGTGGTGTGACGTTTACCAATGAAGCAAAAAAAGGCGGAAAAGCGAAGCCGTTTTGGCGGACTTTTGAGAAAAGCATGACAAAATAGACAAGGATAGGAAAATTAAGTGTAAAATTGTAAACAATTCTCTGCTGGTGATTTATTATAATTTTTAGCAGCAATACTGCGTACAGCGCGCGCCTTCCAGCACCAAGATTTTTTTGGTCTTGAGGGGTGTCGAGACGATGACGTAGCAATGACGTTTGACGCAGATGGGTGACGTGCCTTAAACCGTCCCAATTCTGCAAGTCAGTGCACTTGTTCCCTTGTGCACTAATCATCTAAAGTGTGCACTTACAAAATTGCACACTTCGTGGAAGACCTAGGCGTAGTGTGGGTATTGGGACAGGGCCT

>scaffold_550000165-14

TAGGGCTGCACGATACTGAGAAAATATGCGATGTTGTTGTTGAGTATCGCGATAACGATATTTCTTGCGATATAACATTTCCCCAGAAAAAGGCTAGTTTTATTAGCTATTTTAGCTGCGGGTGTTTTTCGGGCTGGATCATCTCATCAAGATTAGCTCGCTATGAAAGATTCGCGGCTTGTTGATCGTCTTGCGTCAAAGCGCGCGCACGCAAATCCCAACTAACATCCGCAGATATTTTACCATCCTAAAACTTTGACCACACATTACTATTTGAAGTATTAAATTGCTCGTTTATCGCAAACCATTGCGATATGCGTATTTGCGATATTTCGATATATTGTGCAGCCCTA

>scaffold_550000169-1

TTAAAGGGGTCATGAACCGAGAAATCAAAACTCCTTTGATGTTTTGACATATAAGCAGTCGTTGTGCTCTAAAAACAGCTCGTATTGAAGGCAGTCGGCCAAAACGACAGGTTTTGGAATGTTCTACTCGATGACGTAATAGTGTGGATAAGCGCCGCTTCTGCAGAAGATCGACGCCTGCTTCGACATCACTGTTTAGCCCCGCCCACTGATCTGCGCTTGCACGCGATGGGCACATTTTTTAATTGCAAAACTGTTAGCCAATCATAGCAGTGGGCGTTTACTTCCTAGACAACAATCCGCCACGCCTATTTAAACAGAGCGTTCTGATGAAGGGGGTCAAAAACAGGACAGAATATAGCCTATTACTTCCAGATGATGATGTTTTTCGATGTAAAAATCTCGATAACATTATAAGTGGACCTCAGAGAACAGTCCAAAATAAAAAACTGAGGTAGTTCATGACCCCTTTAA

>scaffold_550000169-2

TAGGGATGCACAGATACCGATACTGGTATCGGTATCAGGCCCGATGCTGAGCGCATGTACTCGTAAACACACTCCGATACCAAAGACAGATACCTCTAGTGACGTCATTGACAGAACTTTCAGTGCCTCGGGCGGCAGCGACAGAATGTCAGACTCGGCGGTGTGGAAATATTTCTAAATTAACGATGACAACTCACGCATGTCAGACTGCAAAGTTTGTTCCGCAAAAAATATCAAGAGGTACAAAAACGAGTACTTGGAATACGAGTAATCTGATAGAACATCTGAAATTAAAACAAGTGAGCACGGAGAATTTGCCGCCAGCAGCAACGTTAGCACTTGGCAACCGACTCTGCAGCAAATGTCAAGAGAAAACTCGAGAGCCATACAAATAATCCCACATCCCCAAAAAAGTACAGGTATCGGCGAGTACCAAGAACAAAAAATATCGGTACTCGTACTCTGTCTTTAAAAAAATGGTATCGGTGCATCCCTA

>scaffold_550000173-1

CTTAAAGCCGGGCTTACACTGTGTGATTTTTAATAGTCTTTTGCGATTGCTGCTTGTCAGATTGTACGAAAATGATCCCCATGCCACATTGTAGGATCTCAGTTGTCATAATGCCAGACTGTACGAGACGCGTCAAAAACGGACGCAAACAAGATTACTCATTTACCTCATCAATCAGTGACACGTTCAGTAACCTGCGTTGTCTCGTTAAATGTTAGCTAGCAGCAAACAAACAGCAGCAACGACACATCATACAAACACTCCTTTTCCCTCCATAACTAAATATGTTTCTCTTTGCGTTGTCGCGCTAATTGCGTCACAGGTTCGGCGCTCCTATTGGTTGTTGGTTTGACGGTCGTCGTAGGAGAAGTCACACTGTAGGTAAGTGTCTGAAATCTTCTGACACTGCCAGAATTTCATCAGAGGAAAAAACTGATCACAACGGCCATTAATCGGCTGTCGGTGAACATGTCAAACTAGCAATCAAAGACAACAGATTTTAGCGTAGGATTATAGGAATCTTTTAGGATTTAAAAATTTGTCTCAGGCGACCAAATCGTGGCCAAAATCGCACCGTGTAAGCCCGGCTTAAG

>scaffold_550000176-4

CTAGGGCTGTGTAAAAATATCGAAACAGCTAACTATCGCGATATTTGTTTTCACGATAGTGTATCGATATTTTAACCTCTAGTATCGATACATATTTTTAAATTACATTTTAAACTGATTTTTTCAGTTTATTTGTCTGCCGAGTAATCAATATAATGTGATACACATACTAGAATCCCCCAGATGGTACAAAGCAAGTTGTATTTTTAGCTATTGGTTTAAGAAATGTATTTTGCATTTTTATAAGTACTGCAATATATTGCAATATGTATTGTATTGCAATATATCATGATATATTGTATCGTGACCCATGTATCGTATCGTGAGGCCCTTGCCAATACACAGCCCTAG

>scaffold_550000181-4

GTAGGGCTGCACAATATATAGAAATGATTGAAATATCGCAAATGTGCATATCATGAAATGAGCGATTTAATACATGTAATAGTAATGGTCAAAGTTTTAGGATAGTAAAATATCTGGCACGTGGATGTTAGTTGGGATTTGCGTGCGCGTGCTTTGACGCAAGACCATCAACAAGCTGCAAAGCTTTCATAGCGAGATAATCTTGATCAGATGAACCAGCCCGAAAAACACCCGCAGCTAAAATAGCTAATAAAAATTGCATTTCTCTAGGGAAACGTTATATCGCAAGAAATATCACAATACTCAACAATATATTTTCCCAGTATCGTGCAGCCCTAC

>scaffold_550000181-5

TTAAAGGGGTCATGAAGTGAGAAATCAACATTCCCTTGATCTTTTGACATATAAGAGGCCATTGTGCTATAAAAACATCCTGTAAGTTTCAGAACTCAAAACTTTGTCGTTTCTCTAAAAACAGCTTATATTGAAGGCAGTCTGCCAAAACGACAGGTTTTGGAATGTTCTACTCTATGACGTAATAGTGTGGAAAAGCGCCGCCTGTCGAAACATCACTGTCTAGCTCCGCCCACCAATTCTCGCATGCACGTAATGGGTAAAATGCAAAACCGTTAGCCAATCAGAAGCAGTGGGCGTCTACTTCTGGGACAACAATCCGCCACGCCTATTCAAACAGAGTGTTCTGATGAAGGGGGTCAATAACAGGACAGAAAATAGCCTATTACTTCTAAATGATGATGTATTTTGATGTAAAAATCTTGATAACATTATAAGTGGACCTCCGAGAACAGTACAAAATAAAAAAGTGAGGTAGTTCGTGACCCCTTTAA

>scaffold_550000185-6

TAGGGCTGTGTATTGGCAAGGGCCTCACGATACGATACTTATCGCGATACAATATATCACGATATATTGCAATACATTGCAAATCTTCAACAACAAAAAAATTACAAATAGCTGAAAATACAACTTGCTGTGTGCCACCTGGGGGATTCTAGTATGTGCATCACATTATATTAATTACTCAGCACAAATAAAAACTGAGGGTTTTAATTAAATATTAGCGTAAACCAGTTTAAAACGTAATTTAAATATGTATCGATACTAGTGGTTAGAATATCAATATACTATCGTGAACAAAAATATCGTGATAGTTAGCTGTATCGATATTTTTACACAGCCCTA

>scaffold_550000187-17

TTAGGGCTGTGAAATAATATATCGATACAGCTAACTATTGCGATATTTTTTTTCATATATTGATATATAGATATTCTAACCTCTAGTATGGAGACATATTTAAATTACATTTTAAACTGATTTACGTTTACGTTAATATTTCATTAAAACCCTCAGTTTTAATTTGTGCTGAGCAATCAATATAATGTGATACACATACTAGAATCCCCCAGGTGGTACACAGCAAGTTGTATTTTTAGCTATTTGTTTTCCTTTTTTTTTTTTTATTTTGCAATATATTGTGATATATTGTATCGTGACCCATGTATCGTGATAAGTATCGTATCGAAAGGCCCTTGCCAATACACAGCCCTAA

>scaffold_550000189-7

TAAAGCTGCAGTCCGTAACTTTTTTGGTTAAAAATGATCCGAAATGAATTGTTGAGCAAGTACATTACCATCCAGTGTTCAAAACTATTTTCTTATTTTAGCCTGATTCACAACGGTAATCTTGTAATAATGTTTTATAATTTGAGGGGTACTGGTAAGTTTTCGCAGAAAATAAGAGCATGGCGCCGTTAGTCTTTGCGTCATTGTCACGTCCGTATACATAAAGTGGTCCTGGCTATATCGCATGCTAGGATGCTAACGCTAACACACACTAAACACGTAGTCACGCGATGCTGATGTTGTTAATTTGGGGACAAAGTATAACAATAATAATTTGCTGATGTGATATGAGCTAATTGGCGATCTATATAAATAATAGATTTAATCACCATTGGTAGCATGATTTATTGTAGTGCTTTTTTTTTCAGTTGGTCAGACATGTTACCTGTTTAGATGACGTTCTCTGGTGAAAATTCTTATTTGGTTCATACTTTCAAGATGTAGAGAATCCAAAGTTCAGTATCCACACCGGTTCAGTGACTGACACATACACCGTAAAATATTAGATTTATCCGCAGTGAGGAGCCGTGCCGATGAACAACCCAAGTAAAGAAGATGATTACGCAAATAACTGCAATTGCAGATTTCAAACAGAGATGGCGACAAAGAGGATAAACTTTCGGACTGTAGCTTTA

>scaffold_550000192-8

AGTAGAGCTGCACAATATATTGAAATATCGCAAATGTGGATATTAAGATATGCATATGGCAATGGTTTGCGATAAATGAGAAATTTAATACTTCAAATAGTAATGTGTGGTCAAAGTTTTAGGATTGAAAAATATCTGGCATGCTAATGTTAGTTGGGATATGCATGCGCGTGCTTTGACGCAAGAAAATCAACAAGCTGTGAAGCTTTCATAGCAAGCTAATCTTGATAAGATAAGACAGCCCGAAAAACACCCGCATCTAAAATAGCTAATAAAAATAGCATTTCTCTAGAAACTGATATATCGCAAGAAATATAGTTATGGCAATACTCAAGAACAATATCGCATACCGCATATTTTCCCGGTATCGTGCAGCCCTACT

>scaffold_550000195-3

ATACAATAGGACCTTGATTCACGAACACAATTCGTTCCTAAACTTTGTTCGTGTTCCAATATGTTCGTGACCCAAAGTTCATTTTCCCATAAGGTTTAATGGGAATACGATTAATTTGTTCATGACCATAACGAACCATTATAAATTACGTATTTTGTTTTATTTTACACAGATAATATTAAACAAAGTGTGAAAAACCTTAATATATCTAATATAATTTATAATAGTAAACTAAAACAAAAAAAAACATTAAAAAAGTGAACACTTACGTTGGCTGACACGACTTCTGGCTTGACGGACGAGAGAAGAGGAGGGTGGGTGGGAGTTTGAAGCGCTTGAACGCCCGTCTACTCAAGACCAAAATCAAAAACAGACTAAGCGTGGGTGGAAGTGCATGGGAGATGTACAGTACCCTACAATAACAAGATGGCGGGTGCCTTAGCGGCTCGGATGCACCCACGTAAGATGAGCGATTGCCCGCATTTTTTTTATTTTGGCCATCGGACCATGTTCGTGTTTCAAAAATGAGTTCGTGAATCAAACAGCATTTTTATATATATTTTTGTTCGTGAATCAATTTGTTCGTGGGTTAATGTGTTCGTGAATCGAGGTCCTACTGTAT

>scaffold_550000195-4

TATACAAGGTGAGTGAAAATTAACTAGGCAACATTTAATGGCTATAGAATTTGTAGTATCTCTGGAGTCATGATGAAAACAGTCTCTAAATAGACGATAGTAAATAGGATTTCAAGCCTTGCACACTGGAGGACTTTCACTGGAAGCACGGATTCTGGAGGTTCTCAGCAATATTCCAAACAACGTACTTTACAAGACTGTGAATTCTATCTCCGGCCGTTTGAAAAAACTGGTTGACGCCACTGGTGCCTACATTGAACTGTAAAGATTTGCTTTAATTTTCCTATGTAATAAAGTACATGTACAATTTGTTACAATAAATTTGTATTAGAAATATGGACTTTATTTCCAATTTGAATGCCTAGTTACTTTTCACCCACCCTGTATA

>scaffold_550000205-2

TACAGTAGGACCTCGATTCACGAACACATTAACCCACGAACAAATTGATTCACGAACAAAAATATATATATAAATGCTGTTTGATTCACGAACTCATTTTTGAAACACGAACATGGTCCTATGGCCAAAATGTGAAAAAATGCGGCCAATCGCTCATGCATCCGAGCCGCTATGGGGGCCGCCATCTTGTTATTGTAGGGTACTGTACATCACCCACGCACTTTTACCCATGCTTAGTCTGTTTTTGATTTCAGTCTTGAGTAGACGCGCGTTCATTCTCTTTCCCTCTGCGCAATAATCTTCTCTTCTCTCGTCCGTCAAACCGGAAATCGTGTCAGCCAACGTAAGTGTTCACTTTTTAAATGTTTTTTTGTTTGTTTTAGTTTACTATTATAAATTATATTATTAGATATATTAAGGTTTTTCACACTTTGTTTAATATTATCTGTTTAAAATAAAACATAAAATACGTAATTTATAATGGTTCGTTAGGGTCATGAACAAATTAATCGTATTCCCATTAAACCTTATGGGGAAATTAACTTTGGGTCACAAACAAAGTTTAGGAACGAATTGTATTCGTGAATCGAGGTCCTACTGTA

>scaffold_550000207-4

GTAGGGCTGTGTAAAAATATTGATACAGCTAACTATCATGATTTTTTTTTTTACAATAGTGTATCGGTATTCTAACCTCTAGTATCGATACATATTTAAATTACATTTTAAACTGATTCACGTTTATCTTAATATTTAATTAAAACCCTCAGTTTTTATTTGTCTGCTGAATAATCAATATAATGTGATACACATTCTAGAATCCCCCAGGTGGTAAACAGCAAGATGTATTTTTAGCTATTTGTTTTACTTTTTTATTGTATTGCAATATATTGTGATATGTTGTATCGTGACCCAAGTATCGTGATATGTATCGTATCGTGAGGCCCTTGCCAACACACAGCCCTAC

>scaffold_550000224-5

GGATGCACCGATACCATTTTTTAAAGACTGAGTACGAGTACCAATATTTTTTTTCCTGGTACTTGCCAATACTGATACCAATACCTGTACTTTTTTGGGATGTGGGATTATTTGTGTGGCTCTCAGGTTATTTCTTGCCATTTTCTCTCGTCTTGCAAGGGTTTGCTGCAGGGTTGGTTGCCGAGTGCTAACATTACAGCTAGCGGCAAATTCTCCATGCTCGCTTTTGTGTTTTAATTTCAGATGTTTTATCAGATTACTCATATTATAAGTACTCATTTTTGTACCGCTTGATATTTTTGCGGAACAAAGTTTGCAGTCTGCCATGCGTGGGATGTCATCATTAATTTTGAAATATTTCCACATTCTGAGTCTTTCTGCACTGAAGGTTTTGTCAATGACGTCACAAGAGGTATCGGTCTTTGGTATTGGAGTATTTTTACGAGTACGAGTACAAGTACATAAGCTCAGTATCGGTGCATCC

>scaffold_550000230-13

AGTTGGGCTGTGTAAAAATTTCGACACGGTTAACTATCGCAAAAAATGTGTATCGGTATTCTAACCTCTAGTATCGATACATATTTAAATTACGTTTTAAACTGATTTACGCTTACGTTAACATTTCATTAAAACCCTCAGTGTTTATTTGTCTGCTGAGTAATCAATATAATGTGATACTAGAATCCCCCAGGTGCTACACAGCAAGTTGTATTTTTAACTATTTGTTTTCCTTTTTTTTTTTTTTTGAAGCATTGCAATATATCGTGATATATTGTAACGTGACCCATGTATCATGATGTGTATTGTATCGTGAGGCCCTTGCCAATACACAGCCCTACT

>scaffold_550000232-8

TAGGGCTGTGTATTGGCAAGGTCCTCACGATACAATACATATCATGATACATGGGTCACGTTACAATATATCACCATATATTGCAATACAATACATATTGCAATACATTGTAATACTTAAAAAAAAAAAAAGTTAAACAAATAGTTAAAAATACAACTTGTTGTTTACCACCTGGGGGATTCTAGTATGTGTATCCCATTATATTGATTACTCAGCAGACAAATATAAACTGAGTATTTTAATTAAATATTAACGTAAACGTAAATCAGTTTAAAATGTAATTTAAATATATATCGATACTAGAGGTTAGAATATCAATTCACTATCGTGAAAAAAATTATTGCGATAGTTGAAATTTTTACACAGCCCTA

>scaffold_550000234-2

TACAGTGGAACCTCGGTTTTCGTGATTAATCCGGTCGAAAAAGTCTGATGAAAACCGAATCGTACGAAAACTGAAGCAATATTTTCCATAAGAAATAATGTAAATCCAATTAATCCGTTCCAGACACCCAAAAATATTAACAAAAAATACATTTGATAGAGAATAACTATATTTTTTACATACAGAAAACAATGAGAAAAAAATATAAATGCCTAATGAAATGGATAAATGAACATTTAACATCACTTTTACCTTTATTGAAGACTCTTGTTGGCGTACCTTCGTACTTTGCTACGAGTTCTTTCTTGAATTCTATTGTGTTTCTCGCCTTCTTTATCAACGGGCTGGCACTCTGAACTTTCTTTGGCCCCATGGTGGCTTATTTAGCAGTCGCACTTAATAAACATTGAGGAAATGCGGAAATGCTCGACTCCGAGTCACGCGAGCAGGCGGACGAGTTTGGTACAGACCATTTTCAACTGTACGAAAACCGAGAGGATGTACGAAAACTGGGACAAAATTTCGACAAAAAAAGTTGTCGAAAACCGAATTGTACGAAAACGAGGGCATACGAAAACCGAGGTTCCACTGTA

>scaffold_550000237-4

TAGGGATCCACCGATACCATTTTTTAAAGACCGAGCACAAGTTCCAATATTTTTCCTGGATTCGCGGATACTGATACCAATACCTGTACTTTTTTGTGATGTGATGTGATGTGGGATTATTTGTATGGTTCTGAAGTTATCTCTTGCCATTTTTCTCATATGCTAGGGTTTGCTGTAGGGTTGGTTGGTGAGTGCTAATGTTACTGCTAGCTGCAATTTCTCCATGCTCACTTTTGTGTTTCACTTTCAGATGTTTTATCAGATTACTCGTATTATAAGTACAAATTTTTGGACCTCTTGATATTTTTGCGGAACAAAGTTTGCAGTCTGCCATGCGTGGGTTGTCATCATTAATTTTCAAATTTTTCCACTCCGCTGAGCCTGATATTCTGTCGCTGCTGTCAGAAACTTTGTGCACTGAAAGTTCTGTCAATGACGTCACAAAAGGTATTGGTCTTTGGTATTGGAGTGTTTTTACATGAGCTCAGTATAGGGCCCAATACCGATACCAGTATCGGTATTGGTGCATCCCTA

>scaffold_550000239-11

TCCTCTTGAGACCCAAGGGAAAAAGTGTCATCAATTTTTGTTTTGTTTTGTGTGATTCCCTACTCCTTTCGGGTTCAAAAACATTCTACAATAAAAAAATGTTTAATTGTGATTTGTACAGCAATTTTACAGCATGTCCTCTGTAGTGGACCACAAGACCATTTTAGTTTGAAAAGACAGCCCTTTCCATAAAACATGCTTGTTCTTATGGCGGCCAGATTGTATGCAGTGTAAGAACTAGTTGCTAGCATCTCAGCCAATCAAATAACATATCACTAGAAAGCCCAGGATGTCCTCTTTACAGGACAGCAGAAATGGAAAGATTAGCTCAAAAAATTCAACAAACATTTATGAAAACAAAAGGTCCCCCACAGAGGACACTAGTCAATGGGCAGGGTCTCAGGAGGA

>scaffold_550000243-1

TAGTCAACATTTGAAGTGGATCAAAAAAGTTAATCAAAGTTGTCCTAAGACAAGAACAGGCATTGTTTTGGTTTTAGGACAACTTTAATGAAATGTTTTGATCCACTTCAAATGTTGACTA

>scaffold_550000246-3

ACTAGGGCTGCACAATACATCGAAATTATCAAAATCTCACAAATGTGCATACCGAGATATGCATAACGCAATGGTTTGCGATAAATGACTGATTTAATACTTCAAATAGTAATGTGTGGTCAAAGTTTTAGAATAATAAAATATCTGGCACGCGGATGTTAGTTGGGTTATGTGTGCGCGTGCTTTAACGCTAGACAATCAACAAGCTGCGAAGCTTTCATAGCGAGCTAATCTTGATAAGATGAGCCAGCCTGAAAAACCCCATTAGCAAATTAGCATTGCTCTAGGGAAATGTTATATCTCAAGAAATATCGTTATGGCAACACTCAACAATATTGCATATTTTCCCAGTATCGTGCAGCCCTAGT

>scaffold_550000246-10

TAGGGCTGTGTAAAAATATCGAAACAGCTAACTATCGCAATATTTTTTTCCACGATAGTGTATCGATATATTAAACCTCTAGTATCGAATATTTAAATGACTTTTTAAACTGATTTACTTTTTGTTAATATTCAATTAAAACCCTCAGTTTTTATTTGTCTGCTGAGTAATCAATATAATGTGATTCACATACTAGAATCCCCCAGGTGGTGCACAGCAATTTGTATTTTTAGCTATTTGTTTTAAATAACTAAAAAATTATTTAAGCATTGCAATGTATTGCAATGTGTATTGTATTGCAATATATCGTGATATATTGTATCGTCACCCATGTATCGTGATATGTATCGTATCGCGAGGCCCTTGCCAATACACAGCCCTA

>scaffold_550000246-17

TAACCCTTGTGTGGTGTTTGGGTCTGTAGGACCCGATTTCAAAATTTGTTTAATGAGAAATTATGCTATTCATTATTTTTTTTAGCCCCAGACTCACTGGTCTTGGCTCATTTTTCATGTAGAACATAAAATAGAACATATTTTCCATAACACATCTTTTCAAATGTGATATAACGGGGTAAATGGCAAATATTAACTATAAATGGTGTTTTTTCATAATTTGTTATTGCTGTGATTAAAAACCCAATAATGTGGTGGGACCTGTGAACATTGGCTAGGTAACAAAAACATGAACACCACATGAGGGTTA

>scaffold_550000254-20

TAGGGCTGTGTATTGGCCAGGGCCTCACGATACGATATATGGGTCACGATACAATATATCATGATATATTGCAAATATTGCAATACAATACATATTGCAATACATTTCAATACTTCAAAATAAAACAAGTAAAACAAATAGCTAAAAATACAACTTGCTGTGTAGCACCTGGGAAATTCTAGTATGTGCATCACATTCTATTGATTACTCAGCAGACAAATAAAAAATGAGGGTTTTAATGATTTATTAACGTAAACGTAAATCAGTTTAAAACGTAGTTTAAATATGCATCGATACTAGAGGTTAGAATATCGATACACTATCGTGAAAAAAATATATCGTGATAGTTAGCTGTATCGATATTTTAACACAGCCCTA

>scaffold_550000257-9

AAAGTCATAATTTCTCATTTTTATCTCGTAATTATGACTTCTAAAGTCATAATTTAGACTTTTTTATCTTGTAACTATGAATTAAGTATGTCATAATTATGACTTT

>scaffold_550000259-3

CTACGTTCACACTGTGAGGCTTAGTGCTCAATTCAGATTTTTGCTCAGATCAGTTTTTTTTGTATAGCTGTTTACACTGTTGTTTTAAATGTAACCAATATCAGATTTCCAGTGTGAACTGTTCATGGTCCTAAATTGACCCGCTTGCGCAAAAGAACAAATTCTACGTCTCACGCAGCGTCCTGTCATACAGAAAAATAAACACGGAAGACCCCGAAAGCAGCATTTACTCGTTATGTGCTTAAATTGATACGGTGATGTCCAGAAGACGACAGCGAATTAATGAGTAATCTTACGGAGAAAGAGGATGAAGAGAAAAAGGACCAAACGTTTAATAATAGCATGTTACGGAGCAGTGGCTGCTATTTCAATATGGAGGTGTGTGTGGATGCAGAGCCGGAGCCAGGAGTGGTGGAACCGTGACGTGAACGCCTTTAGCCAAATCGATTATACATCAACTGTTTGTAGTTATGCGCCAGTGAAGAATGACGCATGTCGATCGAAAATGACATAAAAGTCACTAATTACGACATAACTGTTCACACTGCGGTCGCATTGCAAAAACATCTGCCCTGTGTCGGATTTAAATAACACAAAGAAGTGTCACGAATCGGAATTGAAAACATCTGATTCCATGCGGTTTGTGCTGTTCACACCGTCACAAGAAAACAGACCTGAGTCACATGCAAGCAAAAAAAATCCGATTTGGGCCACATTTTCCTGCAGTGTGAACGTAG

>scaffold_550000261-4

TACACTATATGGCCAAACGTCTGCAAACATTTCATTAGCACACCCATAACTGTGTCTTCACCAAACTGTTGTCACAACAATGGGAAGCATTAATTATCTAGGATGTCTCTATATGCTGTAGCATTACGTTTTCCCTACAAGGGAACCAAGGGGCTAAAACAGTGTTCCAGCATGACCATGCCCCTGAGCACAAAGCCCCTGAGCTCCATTAAGACATGGTGTGTTATGGTTGAAGTGGTAGAACTTCAGTGTCCTGTACAGTGGCCTCAACCTCACTGAAAACCTTTAAATGAGAACTGTAACCCTAACTCCACCCCAGACCTTACCTGACAATTGTGTCTGATCTCACTAACACTATTGTAGCTGAATGAGCAAATCACCACAGCCATGCTCAACAATCTAGCACAAAACATTTCCTGAAGTGTGAAGGTTATCAGGTGTAAGAGTGAGACTAAATCTGGAATAGGATGTTTAAAAAACACATATGGTTGTGATAATCAGGTGTCTAAAATCTTTTGGCCATATAGTGTA

>scaffold_5600005-2

TATTCGCATGGGATTAGTATTATCTGGGGACCTCGTGTGATTTAGAAATCCCCCCCCGACATGAATTTGGTGTGGCGCATTTGCACAGATAAGTGAAGCCTGTGATTTTACATACATTTACATTTACATACATCTCCCGGATATGATGTCAAAGCATTGGATAGGCTATTGTTAGGAAATATCTTGTGGTTCTATCATAAACGTAAGAATGTTCCTTACCGCATGCCGCTATACAGTAATTTATCTCCATCTAATCATTCTTTTTGTTTTGGCTCTTCTGGTGGTGTTTAGCTTTCTGAAAGTTGTATGGTTGTTTGGTGTATATCAATTATGCAGCACCCAAAATACACCAAAACACTCCAACACAGCCTCATTCTTCCCACACAGGAAACAGAAACCATAAAAAATGATACACGACACCACAAATCCTGTCACAGAGATGTTGATTCGCACTGGACTAATATTATCACATGACCTCGGTGTTTGGATAAATATGGTAGATCATTTATGGGGGAATTTTTACTTTACAAATTACAGACATGGCCGATTCACACGGGATTGAAATCACAGACAAGCTCCGCAATTATTACACATAACCAGACGACCCCAGATAATACTAATCCCGTGCGAATA

>scaffold_5600009-1

ATTAAAGGTAGGGTAGGTGATTTCGGAGAGGCTAGCAATCGCAAGCTAGCATTGAAAACCAAAGATCCTGCCCTTCCTGCATGATCACTCTGCAAAGCCACGCCTCCTCCATAACACATGACACGCTTGCACAGGCAAACCGGATATTCACCTATATTAACTCGACTTTTAGCTCTTTGCTGATCTAAAACTTTAAGATGTTTTTCTCATGCGATGGCCTGTGCTACATTTAGAGGCAATGTTGGGCTCTTGCTGTTTGCCTTTTTCGCTCAGTCTGCATAGCGGGAACGCGTTAATGACGTATTCTGTCTGCGCGAACAGGGTGCGCAGAGGTTTGCAAATATATATTTGACTGGCAGGTAGGATAGCCTATCGTAATGTTCGGACCGAAGTTTTTTTGGTCCTACGCCTTCCACAGTCCATATAAATACATTTAGACCACTTAACTTAATGAGTGCGATCGAGATGTGAAGAGACTTTCAACCAGCAGAACAAAAAATGTTTTTGAACCACATCACCTACCCTGCCTTTAAT

>scaffold_5600009-2

TTAAAGGTGCAGTAGGACATGCTATGGAAATGCAAACTGTAGCCTGATAGCACTGAAAGCGTACGTCCCACCCTCCCTGCAAATCGCCTTCCAAAGCCACGCCTCCTGAACGTGGAAACGCATCACTGATATTAATTTGGGTTTTCGCTCCAGCCTTTTTTCGCAATAACCCTTTCAAAGACTTTCTTTTGGTGGATACTATTACAAGGTCACTTGACGTGGGTTGTTGGGAAGGAAGGTCAGGTTGTTGCTGTTTGCCTGCTATTCTCGCTCTGTCTGCACAGTGTACGTAAACGCACTGATGACATATCCTGTCTGCGCAAACAGGGTGCGCAGAGGTATGCAAATCCATATGTTGACAGGCAGGTAGGAAAGCCTATCATAATGTTCAGACCAAACATTTTGATTGGACAAACATTTCTTGGTCCTACGCCTTCCACAGAATATATAAATACATTTAGACCACTTAACTTAATGAGCGTTATCGAGACGTGAAAAGACTTTCAACCAGCAGAACAAAAAATGTTTCTGAAGACAATCACCTACTGCACCTTTAA

>scaffold_56000010-1

CTATTCGCATGGGATTAGTATTATCTGGGGACCTCGTGTGATTTAGAGACCCCCCCCCCCCCTGAATTTCGTGTGGCGCATTCGCACAGGATAGGCGAAGCCTGTGATTTTACTCGAATTTACTAACTTATCTCCCGGATATGATGTTAAAGCATTGGATAGGCTTTAGCTTGGAAATATCTTGTGGTTATATCGGTATAGATGTATAATATATCATAAACAGAAGAATGTCCCTTACCGCATGCTGCTATACATGTCATTTATCTCCATCTAATCATTCTTTTAGATTTGGCTCTTCTGGTGGCGTTTAGTTTTATCTTTCTGAAAGTTGTATGGTTGTTTGGTGTATAGCGATTATGCAGCACCCAAAATACACCCAAACACTCCAACACAGCCTCATTCCTCACACACAGGAAACACAAACCTTAAAAACAAATGATACACAACCCCACAAATCCTGGCCAAATCACAGAGATGTCGATTCGCACGGGACTAATATTATCACATGACCTTGGTGTTCGGCGAAATATGGTAGGTCATTTTTACTTTACAAATTACAGACATGGCCGATTCGCACAGGATTAAGATCACAGACAACCGCGGCCATTATTACAAATAACCAGACGTCCCCAGTTAATACTAATCCCGTGCGAATAG

>scaffold_56000010-5

ATTAAAGGTAGGGTAGGTGATTTCGGAGAGGCTAGCAATCGCAAGCTAGCATTGAAAACCAAAGATCCTGCCCTTCCTGCATGATCACTCTGCAAAGCCACGCCTCCTCCATAACACATGACACGCTTGCACAGGCAAACCGGATATTCACCTATATTAACTCGACTTTTAGCTCTTTGCTGATCTAAAACTTTAAGATGTTTTTCTCATGCGATGGCCTGTGCTACATTTAGAGGCAATGTTGGGCTCTTGCTGTTTGCCTTTTTCGCTCAGTCTGCATAGCGGGAACGCGTTAATGACGTATTCTGTCTGCGCGAACAGGGTGCGCAGAGGTTTGCAAATATATATTTGACTGGCAGGTAGGATAGCCTATCGTAATGTTCGGACCGAAGTTTTTTTGGTCCTACGCCTTCCACAGTCCATATAAATACATTTAGACCACTTAACTTAATGAGTGCGATCGAGATGTGAAGAGACTTTCAACCAGCAGAACAAAAAATGTTTTTGAACCACATCACCTACCCTGCCTTTAAT

>scaffold_56000010-6

TTAAAGGTGCAGTAGGACATGCTATGGAAATGCAAACTGTAGCCTGATAGCACTGAAAGCGTACGTCCCACCCTCCCTGCAAATCGCCTTCCAAAGCCACGCCTCCTGAACGTGGAAACGCATCACTGATATTAATTTGGGTTTTCGCTCCAGCCTTTTTTCGCAATAACCCTTTCAAAGACTTTCTTTTGGTGGATACTATTACAAGGTCACTTGACGTGGGTTGTTGGGAAGGAAGGTCAGGTTGTTGCTGTTTGCCTGCTATTCTCGCTCTGTCTGCACAGTGTACGTAAACGCACTGATGACATATCCTGTCTGCGCAAACAGGGTGCGCAGAGGTATGCAAATCCATATGTTGACAGGCAGGTAGGAAAGCCTATCATAATGTTCAGACCAAACATTTTGATTGGACAAACATTTCTTGGTCCTACGCCTTCCACAGAATATATAAATACATTTAGACCACTTAACTTAATGAGCGTTATCGAGACGTGAAAAGACTTTCAACCAGCAGAACAAAAAATGTTTCTGAAGACAATCACCTACTGCACCTTTAA

>scaffold_5700002-1

TATCAAAAAGTTTTGAGACACGCCAACAGATGGCAGCACAAGGCTGCAGGCTGCACGCACAGTCACAGGGAGCACAGCCTTGAGTGCAGCCAGTGTGTGTGTGTGCTGCTTTCTGTTGGCGTGTCTCAAAACTTTTTGATA

>scaffold_5700003-1

GATATGAGGGGTATCAAAAAGTTTCGAGACAAGCCAAGAGATGGCATCACAAGGCTTCACGCACAGCATTGCATGCAGCCTTGTGCTGCCATCTGTTGGCAAGTCTCGAAACTTTTTGATACCCCTCATATC

>scaffold_5700006-26
[truncated: 1,074,224 more chars]
