# Supplementary material for: Draft genome of Glyptosternon maculatum, an endemic fish from Tibet Plateau
Source: Gigascience. 2018 Aug 14;7(9):giy104. doi: 10.1093/gigascience/giy104 (PMC6136493; doi:10.1093/gigascience/giy104)
Supplement: Response_to_Reviewer_Comments_Original_Submission.pdf [file giy104_response_to_reviewer_comments_original_submission.pdf]

Author's Response To Reviewer Comments

Close

Comments from the editor:
Comment: Please make sure the data is in the SRA and the accession is included in the paper before resubmission.
Reply: We have submit data used in this work to NCBI with SRA accession of SRR7279473-SRR7279474, SRR7268130-SRR7268162, SRR7350914-SRR7350921, SRR7351269-SRR7351265, SRR7403445-SRR7403454 under the Project accession number of PRJNA447978 for Illumina, PacBio, 10X Genomics and transcriptome sequencing data used in this work.

Comment: Please include a Fishbase ID with the NCBI taxon ID, and also please use the more up-to-date BUSCO v3 rather than BUSCO v2. We also recommend adding RRIDs for the software tools, see: ftp://penguin.genomics.cn/pub/10.5524/RRID/RRIDlist.pdf
Reply: We have included the Fishbase ID and the NCBI taxon ID in the revised manuscript. For BUSCO version, we have double checked that the BUSCO v3 was used in our study. We are sorry for the mistake and corrected the description in our manuscript. We also have add the RRIDs of the software used in this work.

Reviewer reports:
Reviewer #2: This is a purely descriptive paper reporting the sequencing and genome annotation of Glyptosternon maculatum, an endemic catfish species from the Tibet plateau. This is a straightforward paper and a valuable resource, which deserves publication after minor revision.

- Was the fish individual sequenced a male or a female? Any hint of sex chromosomes in this species?
Reply: The fish individual used in this work was a female, and we have added the information in the manuscript. The sex determination and chromosome for the species were not identified so far, and no heterotropic chromosome was observed from the previous karyotype analysis (Wu Yunfei, Kang Bin, Men Qiang, et al. Chromosome diversity of tibetan fishes. Zoological Research, 1999, 20(4):258-264.) .

- It might be also of interest to predict long non-coding RNA genes (not presented in "all kinds" of non-coding RNA in Tab S5).
Reply: LncRNA is an important non-coding genes in gene expression regulation. However, the transcriptome used in this work were generated from poly-A based protocol, and it is no suitable for lncRNA prediction with the reference genome. Therefore, we did not annotate the lncRNA gene in this work. However, lncRNA regulation in high-altitude would be interesting direction, and related work will be performed in our following studies.

- To affirm that 228 genes are species-specific sounds always strange to me. More precise comparative analysis of their presence/absence in other (related) species should be performed to confirm this.
Reply: Thanks a lot for reviewer’s suggestion. We further blast the 228 genes to NCBI NR (non-redundant) database, and found that 142 genes hit to database with e-value of 1e-5; however, there were still 86 genes failed to hit any protein sequences in the database. The function analysis of those genes is an interesting topic in our following studies.
We have corrected the term of “species-specific genes” to “genes without significant homologous hits” and added the additional analysis to the manuscript. The correction were highlighted by red.

- Repeats: did the authors check for the presence of MITE elements?
Reply: Thanks a lot for reviewer's suggestion. We checked for the presence of MITE elements using MITE-digger with default parameters. The results shown that there were 2,962 MITEs which account for 0.185 % of the whole genome. The resulted MITE sequences were attached as Supplemental_file_MITE to the revised manuscript.

- Species names should be italicized in Fig.2
Reply: We thanks reviewer for the reminding. Species names were italicized in Fig. 2.

- The paper should be edited for typos/grammatical errors
Reply: Thanks a lot for reviewer's suggestion. We have revised and corrected typos and grammatical errors through the manuscript. The corrections were highlighted by red.

- Tab S3: satellites are not interspersed repeat (correct title)
Reply: Thanks reviewer for the reminding. We have corrected the title in Table S3.

Close
