# Supplementary material for: Draft genome of Glyptosternon maculatum, an endemic fish from Tibet Plateau
Source: Gigascience. 2018 Aug 14;7(9):giy104. doi: 10.1093/gigascience/giy104 (PMC6136493; doi:10.1093/gigascience/giy104)
Supplement: Response_to_Reviewer_Comments_Revision_1.pdf [file giy104_response_to_reviewer_comments_revision_1.pdf]

Author's Response To Reviewer Comments

Close

Comments from the editor:
Comment: Please make sure the data is in the SRA and the accession is included in the paper before resubmission.
Reply: We have submit data used in this work to NCBI with SRA accession of SRR7279473-SRR7279474, SRR7268130-SRR7268162, SRR7350914-SRR7350921, SRR7351269-SRR7351265, SRR7403445-SRR7403454 under the Project accession number of PRJNA447978 for Illumina, PacBio, 10X Genomics and transcriptome sequencing data used in this work.

Comment: Please include a Fishbase ID with the NCBI taxon ID, and also please use the more up-to-date BUSCO v3 rather than BUSCO v2. We also recommend adding RRIDs for the software tools, see: ftp://penguin.genomics.cn/pub/10.5524/RRID/RRIDlist.pdf
Reply: We have included the Fishbase ID and the NCBI taxon ID in the revised manuscript. For BUSCO version, we have double checked that the BUSCO v3 was used in our study. We are sorry for the mistake and corrected the description in our manuscript. We also have add the RRIDs of the software used in this work.

Reviewer reports:
Reviewer 1 comments:
This manuscript describes a genome assembly for Glyptosternon maculatum, a Siluriform catfish endemic to the Tibetan plateau. The authors present sufficient data on sequence inputs and assembly methods, and also provide assembly data and analyses of assembly quality.
The manuscript states that only kmers that occurred once were removed for the kmer-based estimation of genome size. Usually the kmer distribution from Illumina sequencing of vertebrate genomes is minimized somewhere beyond a single occurrence - perhaps 3 or 4 occurrences in the data (based on depth of coverage). If the authors are only removing single-occurrence kmers, they may be including untrusted kmers with low occurrence (two or three, perhaps?). This would lead to an overestimation of genome size. If so, then their assembly would be even closer to the estimated genome size.
Reply: We thanks for the reviewer's comment. According to the suggestion, we removed all kmers with less than 3 occurrences and re-analyzed Kmer distribution data. Indeed, we observed that the estimated genome size was 763 Mb, slightly smaller than that (771 Mb) of our previous analysis. We have added the information into our manuscript, which was highlighted by red.

How many iterations of Quiver and Pilon were performed? Current recommendations are to use Quiver to correct SNPs and indels in PacBio assemblies, then to use Pilon to only correct indels since short Illumina reads may be misaligned in repetitive regions.
Reply: The reviewer's question is quite important. We have performed one round of Quiver and Pilon correction using pacbio and NGS data, respectively. In Pilon correction process, because we have observed effects of indel correction using Pilon in our previous analysis (below figure); therefore both snp and indel were corrected in our analysis.

Please state whether the Illumina reads that were mapped with BWA were from the reference individual or another individual.
Reply: Thanks for the reminding. We used Illumina sequencing reads from the reference individual. We stated the detail in our revised manuscript.

Please provide discussion as to why some Trinity contigs only aligned at low coverages (75-85%).
Reply: Thanks for reviewer's suggestion. We have searched our mRNA sequencing reads to NT database and found that the top 5 hits ware all from the closely related fish species, such as Ictalurus punctatus and Zebrafish. Therefore, the probability for external contamination was ruled out (SI Table 7).
We attribute the low coverage of some trinity contig to two fold reasons: 1) the potential chimeric transcript genereated during the transcriptome assembly using trinity, especially for genes with various alternative splicing models; 2) the fragments of genomic contig sequences was also one reason for the low coverage alignment of some assembled transcripts. We have discussed the reason for the low coverage in our manuscript and the revision were highlighted by red.

There is confusion in the text when describing Figure 1b and Figure 1c. See first paragraph of Background information, First line of Sample collection and sequencing. Please clarify. Also, can the map be magnified to better locate the location of the reference sample?
Reply: Thanks for the reviewer's suggestion. Figure 1a and 1b described the G. maculatum and Figure 1c described the location of the sample collection. We have magnified the map in Figure 1c to Tibet-plateau according to the reviewer's suggestion.

Please provide more justification as to why the species in Figure 2 were chosen. Hopefully it is more than just because the data was available. If the purpose is to focus on the divergence between the two Siluriform catfish then are all the other species necessary?
Reply: The reviewer is correct. The species divergence analysis between G. maculatum and I. punctatus was the main purpose for phylogenetic analysis. The analysis could provide us useful information regarding to the species divergence time and relative relationships among fish species. For this purpose, we used other 12 fish genome to construct the phylogenetic tree, not only due to the availability of genome data of those evolutionarily close species, but also because more species (typically 10 or more species in previous studies) are needed to recalibrate the phylogenetic relationships and species divergence time.

Minor corrections:
Line 36 under Background Information - It is unclear what "causing by the unshift of Tibetan plateau" means. Please reword to clarify.
Reply: Thanks for the reviewer's suggestion. We have re-write this part to clarify the sentence. The revision were highlighted by red.

Line 52 under Background Information - Suggest "We thus chose G. manulatum to represent Glyptosternoid group fishes ..."
Reply: Thanks for the reviewer's suggestion. The revision were highlighted by red.

On Line 16 under Protein Coding Gene Prediction, Sinocyclocheilus graham should have the abbreviation Sga, and channel catfish should be listed as Ictalurus punctatus (Ipu).
Reply: Thanks for the reviewer's suggestion. The revision were highlighted by red.

On Line 7 under Functional annotation: "refers"
Reply: Thanks for the reviewer's reminding. We have corrected the sentence and the revision were highlighted by red in the manuscript.

On Line 20: "protein data"
Reply: We have corrected according to the reviewer's comment. The revision were highlighted by red in the manuscript.

Lines 43 and 44: "were" is used twice in the sentence and should only be used once
Reply: We have deleted “were” in the sentence. The revision were highlighted by red in the manuscript.

On Line 13 under Conclusion: "Glyptosternoids".
Reply: Thanks for reviewer’s correction. The revision were highlighted by red in the manuscript.

Reviewer #2: This is a purely descriptive paper reporting the sequencing and genome annotation of Glyptosternon maculatum, an endemic catfish species from the Tibet plateau. This is a straightforward paper and a valuable resource, which deserves publication after minor revision.
- Was the fish individual sequenced a male or a female? Any hint of sex chromosomes in this species?
Reply: The fish individual used in this work was a female, and we have added the information in the manuscript. The sex determination and chromosome for the species were not identified so far, and no heterotropic chromosome was observed from the previous karyotype analysis (Wu Yunfei, Kang Bin, Men Qiang, et al. Chromosome diversity of tibetan fishes. Zoological Research, 1999, 20(4):258-264.) .

- It might be also of interest to predict long non-coding RNA genes (not presented in "all kinds" of non-coding RNA in Tab S5).
Reply: LncRNA is an important non-coding genes in gene expression regulation. However, the transcriptome used in this work were generated from the enrichment by oligo(dT), and it is not suitable for lncRNA prediction with a reference genome. Therefore, we did not annotate the lncRNA gene in this work. However, lncRNA regulation in high-altitude would be interesting direction, and related work will be performed in our following studies.

- To affirm that 228 genes are species-specific sounds always strange to me. More precise comparative analysis of their presence/absence in other (related) species should be performed to confirm this.
Reply: Thanks a lot for reviewer’s suggestion. We further blast the 228 genes to NCBI NR (non-redundant) database, and found that 142 genes hit to database with e-value of 1e-5; however, there were still 86 genes failed to hit any protein sequences in the database. The function analysis of those genes is an interesting topic in our following studies.
We have corrected the term of “species-specific genes” to “genes without significant homologous hits” and added the additional analysis to the manuscript. The correction were highlighted by red.

- Repeats: did the authors check for the presence of MITE elements?
Reply: Thanks a lot for reviewer’s suggestion. We checked for the presence of MITE elements using MITE-digger with default parameters. The results shown that there were 2,962 MITEs which account for 0.185 % of the whole genome. The resulted MITE sequences were attached as Supplemental_file_MITE to the revised manuscript.

- Species names should be italicized in Fig.2
Reply: We thanks reviewer for the reminding. Species names were italicized in Fig. 2.

- The paper should be edited for typos/grammatical errors
Reply: Thanks a lot for reviewer’s suggestion. We have revised and corrected typos and grammatical errors through the manuscript. The corrections were highlighted by red.

- Tab S3: satellites are not interspersed repeat (correct title)
Reply: Thanks reviewer for the reminding. We have corrected the title in Table S3.

Close
