# Supplementary material for: Draft genome of Glyptosternon maculatum, an endemic fish from Tibet Plateau
Source: Gigascience. 2018 Aug 14;7(9):giy104. doi: 10.1093/gigascience/giy104 (PMC6136493; doi:10.1093/gigascience/giy104)
Supplement: Response_to_Reviewer_Comments_Revision_2.pdf [file giy104_response_to_reviewer_comments_revision_2.pdf]

Author's Response To Reviewer Comments

Close

Dear editor,

We appreciate for your decision as“potentially acceptable for publication in GigaScience” for our recent manuscript. To prepare the manuscript for publication, we have revised our manuscript to address the editorial issues you mentioned in your last email:
1) Number of co-first and co-corresponding authors:
We have change the authorship of the manuscript. After negotiating with all authors, three equally contributing first author and two co-corresponding authors were decided and reflected in the revised manuscript.
2) the citation to your GigaDB dataset
We have added the citation of the GigaDB dataset. However, we did not get the doi for the citation, please help us to fill it afterward.
3) Please also ensure that your revised manuscript conforms to the journal style, which can be found in the Instructions for Authors on the journal homepage. Please check again for minor typos and grammatical mistakes, and please remove any highlighting /tracking that was made for the purpose of peer review.
We have revised the manuscript according to the Instructions for Authors and a section for List of abbreviations was added to the manuscript according to the instruction. Minor typos and grammatical mistakes were corrected and tracking was removed in this revision.

Thanks again for your help. We are looking forward for you reply.

Sincerely yours,

Dr. Haiping Liu
Institute of Fisheries Science
Tibet Academy of Agricultural and Animal Husbandry Sciences
Lhasa, P.R. China

Close
